# Use of Genetic Variants Related to Antihypertensive Drugs to Inform on Efficacy and Side Effects

**DOI:** 10.1161/CIRCULATIONAHA.118.038814

**Published:** 2019-06-25

**Authors:** Dipender Gill, Marios K. Georgakis, Fotios Koskeridis, Lan Jiang, Qiping Feng, Wei-Qi Wei, Evropi Theodoratou, Paul Elliott, Joshua C. Denny, Rainer Malik, Evangelos Evangelou, Abbas Dehghan, Martin Dichgans, Ioanna Tzoulaki

**Affiliations:** 1 Department of Epidemiology and Biostatistics, School of Public Health, Imperial College London, United Kingdom (D.G., P.E., E.E., A.D., I.T.).; 2 Institute for Stroke and Dementia Research, University Hospital (M.K.G., R.M., M.D.), Ludwig-Maximilians-Universität LMU, Munich, Germany.; 3 Graduate School for Systemic Neurosciences (M.K.G.), Ludwig-Maximilians-Universität LMU, Munich, Germany.; 4 Department of Hygiene and Epidemiology, University of Ioannina Medical School, Greece (F.K., E.E., I.T.).; 5 Division of Clinical Pharmacology, Department of Medicine (L.J., Q.F.), Vanderbilt University Medical Center, Nashville, TN.; 6 Department of Biomedical Informatics (W.-Q.W., J.C.D.), Vanderbilt University Medical Center, Nashville, TN.; 7 Centre for Global Health Research, Usher Institute of Population Health Sciences and Informatics, University of Edinburgh, United Kingdom (E.T.).; 8 Medical Research Council-Public Health England Centre for Environment, School of Public Health, Imperial College London, United Kingdom (P.E., A.D., I.T.).; 9 Imperial Biomedical Research Centre, Imperial College London and Imperial College NHS Healthcare Trust, UK (P.E.).; 10 UK Dementia Research Institute at Imperial College London, UK (P.E., A.D., I.T.).; 11 Health Data Research UK-London (P.E.).; 12 Munich Cluster for Systems Neurology (SyNergy), Germany (M.D.).; 13 German Center for Neurodegenerative Diseases (DZNE, Munich), Germany (M.D.).

**Keywords:** antihypertensive drugs, Mendelian randomization analysis

## Abstract

**Background:**

Drug effects can be investigated through natural variation in the genes for their protein targets. The present study aimed to use this approach to explore the potential side effects and repurposing potential of antihypertensive drugs, which are among the most commonly used medications worldwide.

**Methods:**

Genetic proxies for the effect of antihypertensive drug classes were identified as variants in the genes for the corresponding targets that associated with systolic blood pressure at genome-wide significance. Mendelian randomization estimates for drug effects on coronary heart disease and stroke risk were compared with randomized, controlled trial results. A phenome-wide association study in the UK Biobank was performed to identify potential side effects and repurposing opportunities, with findings investigated in the Vanderbilt University biobank (BioVU) and in observational analysis of the UK Biobank.

**Results:**

Suitable genetic proxies for angiotensin-converting enzyme inhibitors, β-blockers, and calcium channel blockers (CCBs) were identified. Mendelian randomization estimates for their effect on coronary heart disease and stroke risk, respectively, were comparable to results from randomized, controlled trials against placebo. A phenome-wide association study in the UK Biobank identified an association of the CCB standardized genetic risk score with increased risk of diverticulosis (odds ratio, 1.02 per standard deviation increase; 95% CI, 1.01–1.04), with a consistent estimate found in BioVU (odds ratio, 1.01; 95% CI, 1.00–1.02). Cox regression analysis of drug use in the UK Biobank suggested that this association was specific to nondihydropyridine CCBs (hazard ratio 1.49 considering thiazide diuretic agents as a comparator; 95% CI, 1.04–2.14) but not dihydropyridine CCBs (hazard ratio, 1.04; 95% CI, 0.83–1.32).

**Conclusions:**

Genetic variants can be used to explore the efficacy and side effects of antihypertensive medications. The identified potential effect of nondihydropyridine CCBs on diverticulosis risk could have clinical implications and warrants further investigation.

## Abstract 2

Supplemental Digital Content is available in the text.

**Clinical Perspective**

**What Is New?**

- This work identifies genetic variants that serve as proxies for the effect of angiotensin-converting enzyme inhibitor, β-blocker, and calcium channel blocker antihypertensive drugs.
- Mendelian randomization using the genetic proxies for each respective drug class provides estimates consistent with those of randomized, controlled trials against placebo for effects on risk of coronary heart disease and stroke.
- Phenome-wide association study identifies diverticulosis as a previously unreported possible side effect of calcium channel blockers, with observational analysis further supporting an association between nondihydropyridine calcium channel blocker use and increased risk of diverticulosis.

**What Are the Clinical Implications?**

- Any increase in the risk of diverticulosis related to use of nondihydropyridine calcium channel blockers could have notable consequences and warrants further study.
- No other potential side effects of angiotensin-converting enzyme inhibitors, β-blockers, or calcium channel blockers were identified.

In 2015, the 874 million adults worldwide estimated to have a systolic blood pressure (SBP) of ≥140 mm Hg accounted for 106 deaths per 100 000 and loss of 143 million disability-adjusted life-years,^1^ making hypertension a leading cause of mortality and morbidity. Blood pressure lowering through lifestyle modification or pharmacological treatment can significantly decrease cardiovascular risk, with every 10 mm Hg reduction estimated to decrease risk of all-cause mortality by 13%.^2^

The pharmacological treatment of hypertension is founded on strong evidence, underpinned by a large number of outcome-based randomized, controlled trials (RCTs) that have identified several drug classes to be effective for lowering blood pressure.^3^ However, RCTs based on clinical outcomes have limitations^4^; they are largely restricted to older or high-risk patients and have a relatively short duration of follow-up, rarely beyond 5 years.^5^ Therefore, recommendations for treatment are often based on extrapolation of the available evidence, with known side effects frequently limited to relatively common outcomes captured in RCTs.^6^ At the same time, particular drug treatments for hypertension may have beneficial effects beyond their blood pressure–lowering properties,^6^ thus offering potential for repurposing. However, observational research used to study such opportunities suffers from well-characterized biases, including confounding by indication.^7^

With the growing availability of genome-wide association study (GWAS) meta-analyses, it is becoming increasingly feasible to study drug effects by investigating genetic variants in the genes of their protein targets, as has previously been applied to lipid-lowering drugs.^7^ In this study, human genetic variants within genes corresponding to the targets of common pharmacological agents for hypertension were first identified to serve as a proxy for the effects of these treatments. Second, the validity of this approach for studying the effects of these drugs was investigated by exploring consistency in mendelian randomization (MR) estimates for their effect on coronary heart disease (CHD) and stroke risk with corresponding RCT findings. Finally, to offer insight into their adverse effect profiles and repurposing potential, phenome-wide association study (PheWAS) analyses were undertaken with replication in an external dataset, as well as further investigation in observational analysis of drug use.

## Methods

All supporting data are available within the article, the online-only Data Supplement, and the web links provided. UK Biobank data were accessed through application 236. Relevant ethical approval and participant consent were already obtained in all studies that contributed data to this work. Statistical analysis was undertaken with R version 3.4.1 (The R Foundation for Statistical Computing) and Stata 14.2 (StataCorp LP).

### Genetic Variant Selection

Common antihypertensive drugs were selected for study on the basis of recent consensus guidelines^6^: angiotensin-converting enzyme (ACE) inhibitors, angiotensin receptor blockers, β-blockers (BB), calcium channel blockers (CCB) and thiazide diuretic agents. Genes encoding the targets of these drugs related to effects on blood pressure were identified using the DrugBank database,^8^ with promoter and enhancer regions identified using the GeneHancer database in the GeneCards online platform (version 4.7).^9^ Genetic association estimates for SBP were obtained from a GWAS meta-analysis of 757 601 individuals with European ancestry drawn from the UK Biobank and the International Consortium of Blood Pressure GWAS meta-analysis,^10^ where correction was made for antihypertensive medication use by adding 15 mm Hg to the SBP of participants receiving medication, with further adjustment for body mass index.^10^ In sensitivity analyses, a GWAS of SBP on ≈337 000 white British individuals in the UK Biobank was also used, without correction for medication use or adjustment for body mass index.^11^ Genetic variants to serve as proxies (ie, instruments) for the effect of lower SBP through antihypertensive drug targets were selected as single-nucleotide polymorphisms (SNPs) in corresponding genes, promoter regions, or enhancers that were associated with SBP at genome-wide significance (*P*<5×10^–8^) and clumped to a linkage disequilibrium (LD) threshold of *r*^2^<0.1 using the 1000G European reference panel. This approach does not distinguish between selection of loss-of-function variants or those related to gene expression. The *R*^2^ and F statistics were used to estimate the variance in SBP explained and the strength of each SNP, respectively.^12^

### Statistical Analysis

#### Mendelian Randomization

MR uses randomly allocated genetic variants related to an exposure of interest to study the effect of that exposure on a given outcome. In this study, the exposure of interest was SBP lowering through a particular antihypertensive drug class. All antihypertensive drug classes for which SNPs were identified as proxies using the larger SBP GWAS were taken forward to MR analysis investigating their effect on CHD and stroke risk. Genetic association estimates for CHD were obtained from the CARDIoGRAMplusC4D (Coronary Artery Disease Genome-wide Replication and Meta-analysis [CARDIOGRAM] plus the Coronary Artery Disease [C4D] Genetics) Consortium’s 1000 Genomes–based transethnic meta-analysis of 60 801 case subjects and 123 504 control subjects.^13^ Estimates for stroke risk were obtained from the MEGASTROKE Consortium’s transethnic meta-analysis of 67 162 cases of any stroke and 454 450 control subjects.^14^ Details for the MR analyses are provided in the online-only Data Supplement Methods. To allow comparison with RCT results, all MR estimates were scaled to the SBP-lowering effect of their respective drug class as measured in these RCTs.^3^

### Investigation of Genetic Pleiotropy Unrelated to Drug Effect

The MR estimates can be biased if any of the genetic variants used affect the outcome under consideration through a pleiotropic pathway that is independent of the drug effect for which they serve as proxies. The PhenoScanner curated database of publicly available SNP-phenotype associations (accessed on March 30, 2018) was used to explore whether any of the selected SNPs or proxies with LD *r*^2^>0.8 (using a 1000G reference panel) were also associated at genome-wide significance (*P*<5×10^−^^8^) with traits that may potentially be exerting such pleiotropy,^15^ and any such SNPs were excluded in sensitivity analyses. PhenoScanner includes SNP-phenotype associations identified in analysis of UK Biobank data.^11^ Statistical evaluations of pleiotropy were also incorporated where multiple genetic variants were available to serve as proxies for the drug effect^16^ and are detailed in the Methods in the online-only Data Supplement.

### Phenome-Wide Association Study

The UK Biobank, a prospective study comprising approximately half a million middle-aged individuals,^17^ served as the cohort for the PheWAS investigating drug side effects and repurposing opportunities. The participants provided self-reported information, with blood samples collected for biochemical tests and genotyping and physical measurements performed as described previously.^17^ Individuals were linked retrospectively and prospectively to the National Health Service’s Hospital Episode Statistics database.

PheWAS was restricted to participants of self-reported European descent, with random exclusion of 1 participant from each pair of relatives based on a kinship coefficient >0.0884. For antihypertensive drugs for which genetic variants were identified to serve as proxies, PLINK was used to construct a genetic risk score (GRS) for each individual, weighted for the SBP-lowering effect of each participating SNP,^18^ and standardized to have a mean of 0 and an SD of 1 across all individuals. The 9th and 10th revisions of the *International Classification of Diseases* were used to define cases based on inpatient Hospital Episode Statistics data. The phecode grouping system was used to align diagnoses used in clinical practice with genomic analysis.^19^ A series of case-control groups were generated for each phecode, with control subjects identified as individuals with no record of the respective outcome and its related phecodes.^19^ Analysis was performed with logistic regression after adjustment for age, sex, and first 4 genetic principal components. Only outcomes that had a minimum of 200 cases were considered, to maintain sufficient statistical power to identify associations with common variants.^20^ A 5% threshold with the false-discovery rate method was used in ascertaining the statistical significance of associations, to correct for multiple testing of correlated phenotypes. As for the MR analysis, sensitivity analyses were performed using genetic association estimates derived from the SBP GWAS that did not correct for medication use or adjust for body mass index, and after the exclusion of any SNPs with potentially pleiotropic associations at genome-wide significance that were identified with PhenoScanner.^15^

PheWAS associations for noncardiovascular conditions were investigated for relation to SBP more generally using a permutation-based approach that repeated association analyses 1000 times, with the standardized GRS created on each instance using a matched number of randomly sampled SBP-related SNPs from throughout the genome (ie, associated with SBP at genome-wide significance and clumped to LD *r*^2^<0.001; Table I in the online-only Data Supplement). Compared with the investigation of antihypertensive drug targets, a more stringent LD threshold was used, because variants for SBP were selected from throughout the genome rather than any particular locus. The proportion of such permutation analyses that have a consistent direction of effect and *P* value lower than in the main PheWAS analysis would serve as an adjusted *P* value of the null hypothesis. Further study of any PheWAS associations significant at a false-discovery rate threshold of 5% for noncardiovascular conditions was also undertaken in the Vanderbilt University Biobank (BioVU), for which genetic data on ≈50 000 individuals are linked to a deidentified electronic health record system.^21^ Similar to the main PheWAS, a standardized GRS was constructed, and logistic regression with the outcome was performed after adjustment for age, sex, and first 3 principal components. The analysis was restricted to individuals identified as white, with control subjects based on the same exclusions as the main PheWAS. Results between the UK Biobank and BioVU analysis were pooled by use of a fixed-effects meta-analysis model.

### Observational Analysis of Drug Use

PheWAS associations significant at a 5% false-discovery rate for noncardiovascular conditions related to any antihypertensive class were further explored in observational analysis of drug use among individuals in the UK Biobank. This additionally allowed for investigation of the dihydropyridine and nondihydropyridine CCB subclasses, which was not possible when using genetic proxies because of overlap in the genes for their corresponding protein targets. Cox regression analysis was used to compare time to first incident outcome between individuals orally taking different antihypertensive drug classes at baseline. Individuals who died during the follow-up period before a relevant diagnosis were censored. The categories of antihypertensive drug treatment considered were ACE inhibitors alone, angiotensin receptor blockers alone, BBs alone, dihydropyridine CCBs alone, nondihydropyridine CCBs alone, thiazide diuretic agents alone, a combination of medications from any 2 antihypertensive classes, and a combination of medications from 3 or more antihypertensive classes. In a separate model, individuals who were taking any subclass of CCBs were pooled into a single category. Adjustment was made for age, sex, body mass index, Townsend Deprivation Index, smoking status, previous cancer diagnosis, number of noncancer diagnoses, and number of previous surgical operations. Individuals with a diagnosis of the condition under consideration before recruitment were excluded.

## Results

### Genetic Variant Selection

The genes and enhancer and promoter regions corresponding to the targets of each antihypertensive drug class are shown in Table II in the online-only Data Supplement. There was 1 gene identified for each drug target for ACE inhibitors (*ACE*), angiotensin receptor blockers (*AGTR1*), BBs (*ADRB1*), and thiazide diuretic agents (*SLC12A3*), and 11 genes for CCBs (*CACNA1D*, *CACNA1F*, *CACNA2D1*, *CACNA2D2*, *CACNA1S*, *CACNB1*, *CACNB2*, *CACNB3*, *CACNB4*, *CACNG1*, and *CACNA1C*) encoding the different calcium channel subunits related to effects on blood pressure. The *CACNA1F* gene is located on the X chromosome, and SNPs corresponding to this region were not available. Using the predefined selection criteria, there was 1 SNP identified for ACE inhibitors, 6 for BBs, and 24 for CCBs (Tables III through V in the online-only Data Supplement). The larger number of SNPs and correspondingly greater proportion of variation in SBP explained for CCBs was related to the availability of more genes from which to identify variants. The F statistic for SNPs ranged from 54 to 534 (Tables III through V in the online-only Data Supplement), consistent with a low risk of weak instrument bias.^12^

### Mendelian Randomization

**Figure 1.**
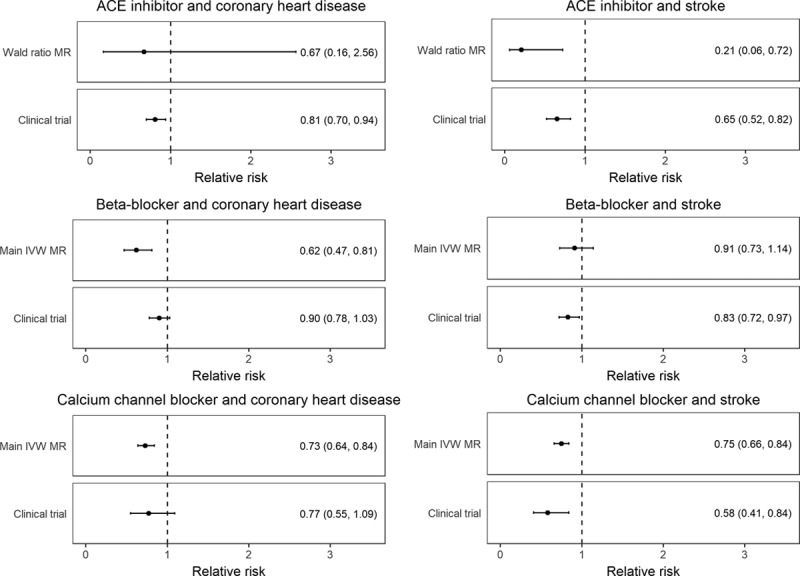
**MR estimates for the effect of genetically lower systolic blood pressure through the ACE inhibitor, β-blocker, and calcium channel blocker variants, respectively, on risk of coronary heart disease and stroke, compared with randomized, controlled trial meta-analysis results.^3^** ACE indicates angiotensin-converting enzyme; IVW, inverse variance weighted; and MR, Mendelian randomization.

To allow comparison with RCT meta-analysis effect estimates, MR results for each drug class were scaled to their respective SBP-lowering effect in these studies. Thus, for ACE inhibitors, MR estimates are given per 21.14 mm Hg decrease, for BBs per 9.51 mm Hg decrease, and for CCBs per 8.90 mm Hg decrease.^3^ MR analysis using the single genetic variant identified for ACE inhibitors showed a protective effect on stroke (relative risk [RR], 0.21; 95% CI, 0.06–0.72; *P*=0.01) but not CHD risk (RR, 0.67; 95% CI, 0.16–2.56; *P*=0.58). The main MR analysis using the 6 variants for BBs identified a protective effect on CHD risk (RR, 0.62; 95% CI, 0.47–0.81; *P*=4×10^−^^4^) but not stroke risk (RR, 0.91; 95% CI, 0.73–1.14; *P*=0.41). For CCBs, the main MR analysis using the 24 SNPs identified a protective effect on both CHD risk (RR, 0.73; 95% CI, 0.64–0.84; *P*=6×10^−^^6^) and stroke risk (RR, 0.75; 95% CI, 0.66–0.84; *P*=1×10^−^^6^). Similar results for all drug classes were obtained when the incidence of CHD and stroke was considered to be 1%, 5%, and 10% (Table VI in the online-only Data Supplement). The MR estimates had overlapping 95% CIs to those from RCTs of these drugs versus placebo^3^ (Figure 1). Individual MR estimates for each BB and CCB SNP are given in Figures I through IV in the online-only Data Supplement. Consistent MR results were found in sensitivity analyses, as detailed in the online-only Data Supplement (Results, Tables VII through IX, and Figures V through VIII).

### Phenome-Wide Association Study

**Figure 2.**
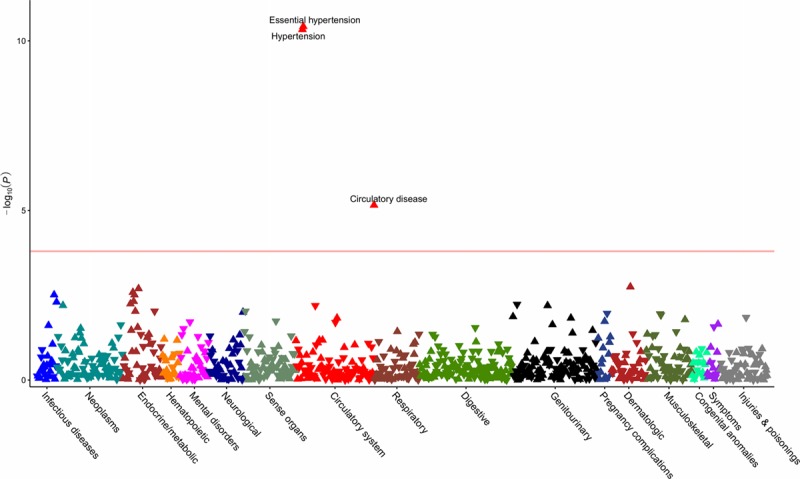
**Phenome-wide association study of the standardized genetic risk score for angiotensin-converting enzyme inhibitors.** The horizontal line depicts the 5% false-discovery rate threshold.

**Figure 3.**
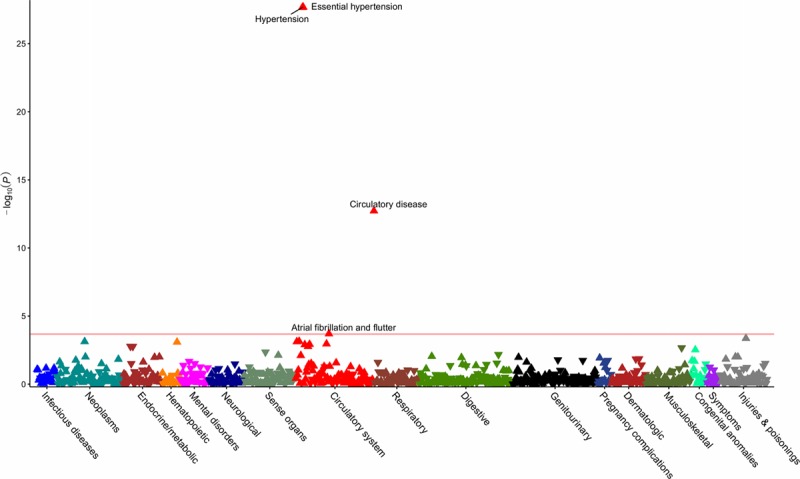
**Phenome-wide association study of the standardized genetic risk score for β-blockers.** The horizontal line depicts the 5% false-discovery rate threshold.

**Figure 4.**
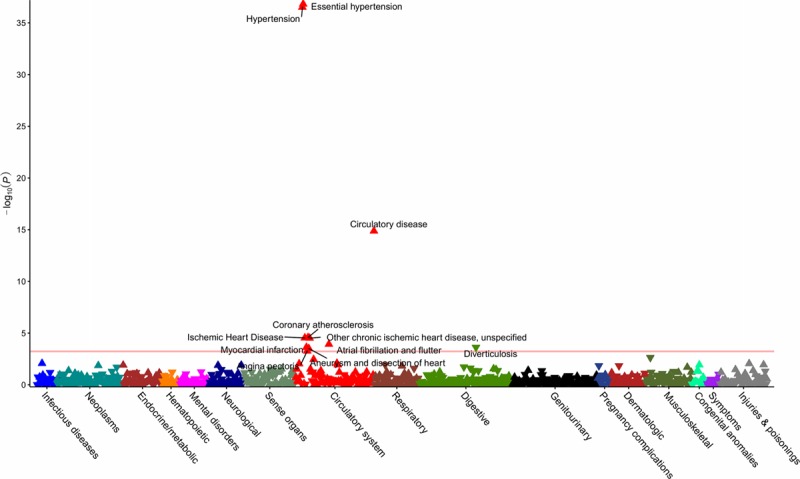
**Phenome-wide association study of the standardized genetic risk score for calcium channel blockers.** The horizontal line depicts the 5% false-discovery rate threshold.

After quality control and mapping of *International Classification of Diseases, 9th Revision* and *10th Revision*, to phecodes, data for 424 439 individuals across 909 distinct phenotypes were available for PheWAS analysis. Details of the number of phenotypes and cases per disease category are provided in the Table, with the number of cases and controls for each outcome in Tables X through XVI in the online-only Data Supplement. Using the ACE inhibitor, BB, and CCB standardized GRS, the respective PheWAS analyses revealed associations with hypertension and related cardiovascular disease (Figures 2–4 and Tables X through XII in the online-only Data Supplement). CCBs additionally showed an association with higher risk of diverticulosis (odds ratio per SD increase in standardized GRS, 1.02; 95% CI, 1.01–1.04, *P*=2×10^−^^4^). Similar results were obtained in PheWAS sensitivity analyses (Tables XIII through XVI in the online-only Data Supplement). Random sampling of 24 SBP SNPs from throughout the genome (Table I in the online-only Data Supplement) to create standardized GRSs and measurement of associations with diverticulosis risk in permutation analyses (N=1000) showed effect estimates centered close to the null (mean odds ratio per SD increase in standardized GRS, 1.00; 95% CI, 0.98–1.02, *P*=0.79; Figure IX in the online-only Data Supplement). Of the 1000 permutation analyses, only 10 had a consistent direction of effect and *P* value lower than that observed for the association of the standardized CCB GRS with diverticulosis in PheWAS, thus generating an adjusted *P* value=0.01.

**Table.**
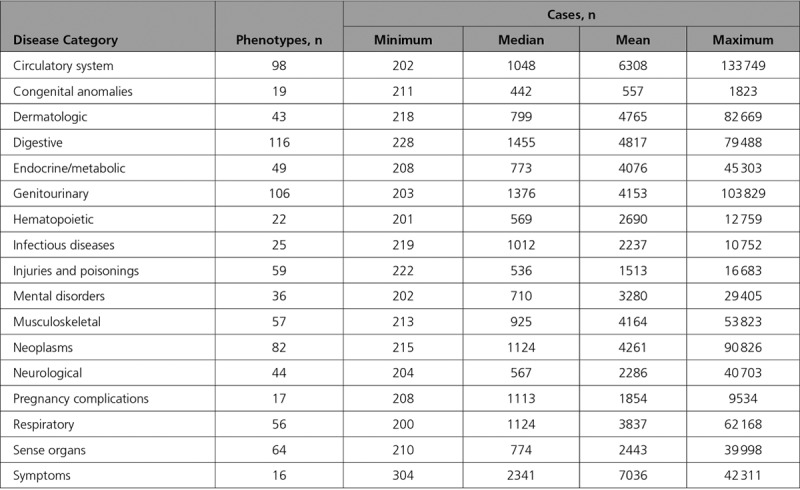
Number of Phenotypes and Cases per Disease Category in the UK Biobank Phenome-Wide Association Study Analysis

**Figure 5.**
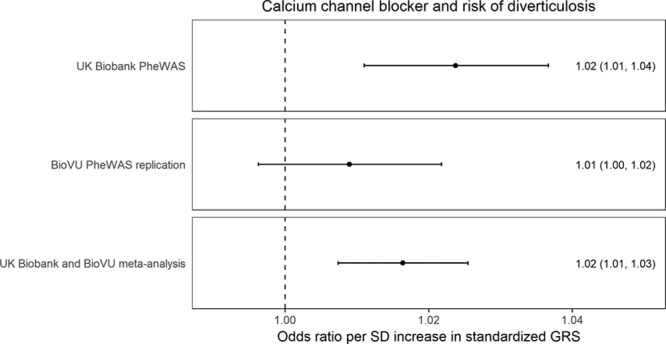
**Estimates for genetic association between calcium channel blockers and diverticulosis risk derived from PheWAS analyses in the UK Biobank and BioVU, respectively, and their fixed-effects pooled estimate.** BioVU indicates Vanderbilt University Biobank; GRS, genetic risk score; and PheWAS, phenome-wide association study.

Data for 45 517 individuals were available in BioVU to further investigate novel PheWAS findings for traits unrelated to hypertension. General cohort characteristics for the considered populations from the UK Biobank and BioVU are detailed in Table XVII in the online-only Data Supplement. The prevalence of diverticulosis in BioVU was 12%, comparable to the 10% observed in the UK Biobank. In BioVU, the CCB standardized GRS association with diverticulosis had an odds ratio per SD increase of 1.01 (95% CI, 1.00–1.02; *P*=0.17). The meta-analyses of UK Biobank and BioVU estimates had an odds ratio of 1.02 (95% CI, 1.01–1.03; *P*=3×10^−^^4^; Figure 5).

### Observational Analysis of Drug Use

For the observational analysis of antihypertensive drug use in the UK Biobank, there were 1408 incident diverticulosis diagnoses up to February 13, 2016, in the 54 612 individuals taking any of the considered antihypertensive drug classes at recruitment (March 13, 2006, to October 1, 2010), with a mean follow-up of 2538 days. In adjusted Cox regression (with use of thiazide diuretic antihypertensive medications alone as the reference category), there was no evidence for an association between use of any CCB and risk of diverticulosis (hazard ratio, 1.10; 95% CI, 0.88–1.35; *P*=0.43). Considering CCB subclasses, there was evidence for an association with risk of diverticulosis for nondihydropyridine CCB use (hazard ratio, 1.49; 95% CI, 1.03–2.14; *P*=0.03) but not dihydropyridine CCB use (hazard ratio, 1.01; 95% CI, 0.80–1.28; *P*=0.91) or any other antihypertensive drug class (Table XVIII in the online-only Data Supplement).

## Discussion

This work leveraged large-scale GWAS data from >750 000 individuals and generated genetic proxies for the effect of ACE inhibitors, BBs, and CCBs, 3 of the most commonly used medications worldwide. The MR estimates for risk of CHD and stroke were comparable to those observed in RCTs against placebo, which supports the validity of the approach. PheWAS on 909 outcomes corroborated the known efficacy of these agents in preventing hypertension and related vascular diseases, thus further supporting the robustness of the genetic variants used.

The PheWAS investigation also revealed an increased risk of diverticulosis associated with the standardized GRS for CCBs. No significant association with diverticulosis risk was identified when SBP SNPs were explored more generally, which makes effects through systemic SBP lowering unlikely to account for this. A consistent association between the standardized CCB GRS and diverticulosis risk was found in BioVU, which contained fewer cases and had a correspondingly wider CI that crossed the null. The finding was further supported by observational analysis suggesting that nondihydropyridine CCB treatment at baseline in the UK Biobank was associated with increased risk of diverticulosis. Dihydropyridine and nondihydropyridine CCBs have different pharmacological effects, and it also follows that their side effect profiles vary.^22^ In terms of a possible mechanism, constipation is an established side effect of nondihydropyridine CCBs, related to their role in reducing bowel contractility,^23^ and it may be through a similar process that the risk of diverticulosis is increased. Alternatively, there may be specific effects on the vasa recta vessels that penetrate the muscle layer of the colon, thus giving rise to weak points where diverticulae consequently form.^24^ Complications related to diverticulosis are a common reason for hospital admission^25^ and have a rising incidence.^26^ Given that more than one-tenth of the world’s adults have hypertension, and CCBs are recommended as a first-line pharmacological agent, with nondihydropyridine drugs in particular recommended for individuals with concurrent atrial fibrillation,^1,6^ the clinical implications of these findings merit consideration. For example, individuals with or at increased risk of developing diverticulosis might benefit from alternative pharmacological treatments for hypertension. The genetic proxies for ACE inhibitors, BBs, and CCBs did not show detrimental associations with any of the other traits examined in PheWAS. Although absence of evidence is not evidence of absence, this does provide some assurance that long-term pharmacological inhibition of these drug targets is generally safe, with other side effects that require hospitalization being smaller or rarer.

A major strength of our work is that it uses genetic variants to investigate the effect of antihypertensive drugs using existing data obtained from large-scale studies, thus avoiding the time and resource constraints associated with such study through RCTs^4^ and overcoming the limitations of potential confounding and reverse causation from use of standard observational methods.^7^ A range of sensitivity analyses supported the robustness of this approach, with PheWAS allowing rapid investigation of hundreds of clinically relevant traits across the phenome. Additionally, observational analysis allowed for consideration of CCB subclasses and further replication of novel findings.

Concerning the limitations of the study, the MR and PheWAS results estimate the cumulative effect of lifelong exposure to genetic variants, rather than the consequence of a clinical intervention. Furthermore, there may be unknown pleiotropic effects of the genetic variants that bias the association estimates.^16^ Although less stringent criteria for selecting instruments (such as a more relaxed *P* value threshold for association with SBP, or a more lenient LD criterion for clumping) might have increased the number of variants available, this could also have reduced the sensitivity and specificity of the analysis because of the introduction of weak instrument bias and invalid instruments, respectively. Similarly, information on gene expression was not incorporated in this work, and although such an approach could offer an additional strategy for identifying genetic variants that serve as proxies for drug effects,^7^ this would be restricted to the cells or tissues in which gene expression was measured, limiting applicability for exploration of general side effects or repurposing opportunities. Although the PheWAS analysis was performed to explore clinically relevant outcomes identified using harmonized Hospital Episode Statistics data in UK Biobank participants, there is also the potential to extend this approach to other cohorts and summary-level genetic data.^15^ Finally, although the observational analysis of drug use in the UK Biobank did support an association between nondihydropyridine CCB use and risk of diverticulosis, it is not clear whether this finding may in part relate to ascertainment bias or residual confounding. Diverticulosis can be incidental in asymptomatic individuals, and as such, increased interaction with healthcare services could lead to a greater chance of diagnosis.

In conclusion, this work has identified genetic variants that serve as proxies for the effect of the ACE inhibitor, BB, and CCB classes of antihypertensive medication. In MR and PheWAS, our instrumental variable approaches corroborated the established associations of these agents with a range of traits related to hypertension. Additionally, this study identified an apparent, previously unreported detrimental effect of nondihydropyridine CCBs on risk of diverticulosis, a finding that requires further replication before it should alter clinical practice. No other potential side effects of any drug class were identified to suggest a lack of long-term safety. This study demonstrates that the use of genetic variants offers a powerful complement to existing RCT and observational approaches for investigating the efficacy, side effects, and repurposing potential of antihypertensive agents.

## Acknowledgments

D.G., M.K.G., M.D., and I.T. designed the study. D.G., M.K.G., F.K., and L.J. collectively had full access to the data and performed the analysis. All authors interpreted the results. D.G. and I.T. drafted the manuscript. All authors critically revised the manuscript for intellectual content. All authors approved the submitted version and are accountable for the integrity of the work.

## Sources of Funding

This work was funded by the Wellcome 4i Clinical PhD Program at Imperial College London (to D.G.); scholarships from the Deutscher Akademischer Austauschdienst and the Onassis Foundation (to M.K.G.); National Institute of Health grants R01 HL133786, R01 GM120523 and R01 LM010685 (to Q.F., W.-Q.W., and J.C.D.); Cancer Research UK grants C31250/A22804 (to E.T.); the Medical Research Council and Public Health England (grant MR/L01341X/1) (to P.E., as part of the Medical Research Council-Public Health England Centre for Environment and Health); the National Institute for Health Research Imperial Biomedical Research Centre in collaboration with Imperial College Healthcare National Health Service Trust (to P.E.); the UK Medical Research Council, Alzheimer’s Society and Alzheimer’s Research UK (to P.E., as part of the UK Dementia Research Institute); a consortium led by the UK Medical Research Council (to P.E., as associate director of Health Data Research UK); the European Union Horizon 2020 research and innovation programme “Small vessel diseases in a mechanistic perspective: Targets for Intervention” (SVDs@target, No 666881; to M.D.); the German Research Foundation (DFG) as part of the “Munich Cluster for Systems Neurology” (SyNergy, EXC 1010) and the Collaborative Research Center 1123 (B3) (to M.D.).

## Disclosures

None.

## Supplementary Material

## References

[1] Forouzanfar MH, Liu P, Roth GA, Ng M, Biryukov S, Marczak L, Alexander L, Estep K, Hassen Abate K, Akinyemiju TF (2017). Global burden of hypertension and systolic blood pressure of at least 110 to 115 mm Hg, 1990-2015.. JAMA.

[2] Ettehad D, Emdin CA, Kiran A, Anderson SG, Callender T, Emberson J, Chalmers J, Rodgers A, Rahimi K (2016). Blood pressure lowering for prevention of cardiovascular disease and death: a systematic review and meta-analysis.. Lancet.

[3] Wright JM, Musini VM, Gill R (2018). First-line drugs for hypertension.. Cochrane Database Syst Rev.

[4] Frieden TR (2017). Evidence for health decision making - beyond randomized, controlled trials.. N Engl J Med.

[5] Tsang R, Colley L, Lynd LD (2009). Inadequate statistical power to detect clinically significant differences in adverse event rates in randomized controlled trials.. J Clin Epidemiol.

[6] Williams B, Mancia G, Spiering W, Agabiti Rosei E, Azizi M, Burnier M, Clement DL, Coca A, de Simone G, Dominiczak A, Authors/Task Force Members (2018). 2018 ESC/ESH guidelines for the management of arterial hypertension: the Task Force for the management of arterial hypertension of the European Society of Cardiology and the European Society of Hypertension.. J Hypertens.

[7] Walker VM, Davey Smith G, Davies NM, Martin RM (2017). Mendelian randomization: a novel approach for the prediction of adverse drug events and drug repurposing opportunities.. Int J Epidemiol.

[8] Wishart DS, Knox C, Guo AC, Shrivastava S, Hassanali M, Stothard P, Chang Z, Woolsey J (2006). DrugBank: a comprehensive resource for in silico drug discovery and exploration.. Nucleic Acids Res.

[9] Fishilevich S, Nudel R, Rappaport N, Hadar R, Plaschkes I, Iny Stein T, Rosen N, Kohn A, Twik M, Safran M (2017). GeneHancer: genome-wide integration of enhancers and target genes in GeneCards.. Database.

[10] Evangelou E, Warren HR, Mosen-Ansorena D, Mifsud B, Pazoki R, Gao H, Ntritsos G, Dimou N, Cabrera CP, Karaman I, Million Veteran Program (2018). Genetic analysis of over 1 million people identifies 535 new loci associated with blood pressure traits.. Nat Genet.

[11] Churchhouse C, Neale B Rapid GWAS of thousands of phenotypes for 337,000 samples in the UK Biobank.. http://www.nealelab.is/blog/2017/7/19/rapid-gwas-of-thousands-of-phenotypes-for-337000-samples-in-the-uk-biobank.

[12] Palmer TM, Lawlor DA, Harbord RM, Sheehan NA, Tobias JH, Timpson NJ, Davey Smith G, Sterne JA (2012). Using multiple genetic variants as instrumental variables for modifiable risk factors.. Stat Methods Med Res.

[13] Nikpay M, Goel A, Won HH, Hall LM, Willenborg C, Kanoni S, Saleheen D, Kyriakou T, Nelson CP, Hopewell JC (2015). A comprehensive 1,000 Genomes-based genome-wide association meta-analysis of coronary artery disease.. Nat Genet.

[14] Malik R, Chauhan G, Traylor M, Sargurupremraj M, Okada Y, Mishra A, Rutten-Jacobs L, Giese AK, van der Laan SW, Gretarsdottir S, AFGen Consortium; Cohorts for Heart and Aging Research in Genomic Epidemiology (CHARGE) Consortium; International Genomics of Blood Pressure (iGEN-BP) Consortium; INVENT Consortium; STARNET; BioBank Japan Cooperative Hospital Group; COMPASS Consortium; EPIC-CVD Consortium; EPIC-InterAct Consortium; International Stroke Genetics Consortium (ISGC); METASTROKE Consortium; Neurology Working Group of the CHARGE Consortium; NINDS Stroke Genetics Network (SiGN); UK Young Lacunar DNA Study; MEGASTROKE Consortium; MEGASTROKE Consortium: (2018). Multiancestry genome-wide association study of 520,000 subjects identifies 32 loci associated with stroke and stroke subtypes.. Nat Genet.

[15] Staley JR, Blackshaw J, Kamat MA, Ellis S, Surendran P, Sun BB, Paul DS, Freitag D, Burgess S, Danesh J (2016). PhenoScanner: a database of human genotype-phenotype associations.. Bioinformatics.

[16] Burgess S, Bowden J, Fall T, Ingelsson E, Thompson SG (2017). Sensitivity analyses for robust causal inference from Mendelian randomization analyses with multiple genetic variants.. Epidemiology.

[17] Sudlow C, Gallacher J, Allen N, Beral V, Burton P, Danesh J, Downey P, Elliott P, Green J, Landray M (2015). UK Biobank: an open access resource for identifying the causes of a wide range of complex diseases of middle and old age.. PLoS Med.

[18] Purcell S, Neale B, Todd-Brown K, Thomas L, Ferreira MA, Bender D, Maller J, Sklar P, de Bakker PI, Daly MJ (2007). PLINK: a tool set for whole-genome association and population-based linkage analyses.. Am J Hum Genet.

[19] Li X, Meng X, Spiliopoulou A, Timofeeva M, Wei WQ, Gifford A, Shen X, He Y, Varley T, McKeigue P (2018). MR-PheWAS: exploring the causal effect of SUA level on multiple disease outcomes by using genetic instruments in UK Biobank.. Ann Rheum Dis.

[20] Verma A, Bradford Y, Dudek S, Lucas AM, Verma SS, Pendergrass SA, Ritchie MD (2018). A simulation study investigating power estimates in phenome-wide association studies.. BMC Bioinformatics.

[21] Denny JC, Bastarache L, Ritchie MD, Carroll RJ, Zink R, Mosley JD, Field JR, Pulley JM, Ramirez AH, Bowton E (2013). Systematic comparison of phenome-wide association study of electronic medical record data and genome-wide association study data.. Nat Biotechnol.

[22] Frishman WH (2007). Calcium channel blockers: differences between subclasses.. Am J Cardiovasc Drugs.

[23] Morris CR, Harvey IM, Stebbings WS, Speakman CT, Kennedy HJ, Hart AR (2003). Do calcium channel blockers and antimuscarinics protect against perforated colonic diverticular disease? A case control study.. Gut.

[24] Brian West A (2006). The pathology of diverticulosis: classical concepts and mucosal changes in diverticula.. J Clin Gastroenterol.

[25] Everhart JE, Ruhl CE (2009). Burden of digestive diseases in the United States part II: lower gastrointestinal diseases.. Gastroenterology.

[26] Etzioni DA, Mack TM, Beart RW, Kaiser AM (2009). Diverticulitis in the United States: 1998-2005: changing patterns of disease and treatment.. Ann Surg.

