# Supplementary material for: Use of Genetic Variants Related to Antihypertensive Drugs to Inform on Efficacy and Side Effects
Source: Circulation. 2019 Jun 25;140(4):270–9. doi: 10.1161/CIRCULATIONAHA.118.038814 (PMC6687408; doi:10.1161/CIRCULATIONAHA.118.038814)
Supplement: Supplementary file 1 [file cir-140-270-s001.pdf]

# SUPPLEMENTAL MATERIAL

# Supplemental Methods

## Mendelian randomization (MR)

In the main MR analysis, estimates for each single-nucleotide polymorphism (SNP) were derived using the Wald ratio method, with standard errors estimated using second order weights to allow for measurement error in both the exposure and outcome estimates [1]. For drug targets with more than one related SNP, overall MR estimates were calculated by pooling individual MR estimates for each SNP using fixed-effects inverse-variance weighted (IVW) meta-analysis [1], and were scaled to the estimated effect of the corresponding drug target on systolic blood pressure (SBP) in randomized controlled trials (RCTs) [2], in order to reflect drug effect. After conversion of odds ratio estimates to relative risk (RR) using baseline incidences of CHD and stroke of 0.042 and 0.041 respectively from a systematic review of 613,815 participants enrolled in blood pressure lowering trials [3], MR results were compared with estimates from a recent Cochrane systematic review and meta-analysis of RCTs that investigated the effect of common antihypertensive drugs against placebo [2]. Sensitivity analyses were also performed using MR RR estimates derived from baseline CHD and stroke incidences of 1%, 5% and 10%.

## Investigation of pleiotropy

Heterogeneity in the MR estimates generated by different SNPs can be used to indicate such pleiotropy [4], which was identified through a significant Cochran's Q test ($P$<0.05) or an $I^2$ measure of heterogeneity >30%. MR statistical sensitivity analyses that are more robust to the inclusion of pleiotropic variants were also performed. Firstly, the weighted median estimator was used, which obtains an overall MR estimate by ordering individual SNP MR estimates by their magnitude weighted for their precision, and is reliable when more than half the information for the analysis comes from valid instruments [5]. Secondly, the MR-Egger technique was performed, which regresses the SNP-outcome estimates against the SNP-exposure estimates, weighted for the precision of the

SNP-outcome estimates to give a reliable MR estimate and test for the presence of directional pleiotropy in scenarios where any pleiotropic effect of the genetic variants is independent of their association with the exposure [6]. Finally, MR-PRESSO was conducted, which performs a zero-intercept regression of the SNP-outcome estimates against the SNP-exposure estimates to test, using residual errors, whether there are outlier SNPs ($P<0.05$), and whether removing these changes the MR estimates generated [7]. MR-PRESSO generally requires that at least half of the genetic variants used do not relate to the outcome independently of the exposure [7]. Statistical sensitivity analyses in MR suffer from low power [4], and as such no formal statistical significance threshold was set for these.

# Supplemental Results

## Mendelian randomization

The main variants used to proxy drug class effect were based on genetic association estimates that corrected for antihypertensive medication use and adjusted for body mass index (BMI) [8]. To avoid possible bias related to medication non-compliance or introduction of collider effects respectively, sensitivity analyses were performed using the UK Biobank SBP GWAS that did not correct for medication use or adjust for BMI [9]. No suitable variants were identified for ACEI, two SNPs were identified as variants for BB (Supplementary Table 7), and six SNPs as variants for CCB (Supplementary Table 8). IVW MR produced estimates that were comparable to the main analysis, but with wider confidence intervals (Supplementary Figures 5-8). Searching PhenoScanner [10], possible pleiotropic effects were identified for one BB SNP and five CCB SNPs (details are provided in Supplementary Table 9). Repeating the IVW MR analysis after excluding these SNPs also produced similar estimates to the main analysis (Supplementary Figures 5-8).

There was only evidence of heterogeneity, suggesting possible bias related to pleiotropic SNPs, in the MR analysis of BBs on stroke risk ($I^2$ 59%, Cochran's Q $P$=0.03). The MR-Egger intercept was not significant for directional pleiotropy for either BBs (CHD $P$=0.87 and stroke $P$=0.89) or CCBs (CHD $P$=0.89 and stroke $P$=0.51). MR-PRESSO only detected outlier SNPs in the analysis of BBs on stroke risk (2 outliers), with MR-PRESSO estimates that excluded these SNPs consistent with the main analysis results (Supplementary Figure 6). Estimates using MR-Egger regression, the weighted median approach and MR-PRESSO also produced similar estimates to the main IVW MR analyses (Supplementary Figures 5-8).

# Supplemental Tables

Supplementary Table 1. Single-nucleotide polymorphisms (SNPs) related to systolic blood pressure at genome-wide significance, clumped to linkage disequilibrium $r^2 < 0.001$ using a 1000G European reference panel.

| SNP | Chr | Pos | EA | OA | EAF | Effect | SE | P value | Total sample size | $R^2$ | F statistic |
|---|---|---|---|---|---|---|---|---|---|---|---|
| rs7796 | 1 | 1684169 | c | g | 0.5114 | 0.3385 | 0.0314 | 5.00E-27 | 726899 | 4.60E-04 | 338 |
| rs263532 | 1 | 2164116 | t | c | 0.5755 | 0.1798 | 0.0307 | 4.72E-09 | 735052 | 2.40E-04 | 178 |
| rs2493296 | 1 | 3327032 | t | c | 0.1425 | 0.4183 | 0.0442 | 3.14E-21 | 724085 | 2.80E-04 | 204 |
| rs10779795 | 1 | 6677064 | a | g | 0.6613 | 0.2191 | 0.032 | 7.44E-12 | 729906 | 2.70E-04 | 197 |
| rs488834 | 1 | 10767902 | t | c | 0.7645 | -0.3799 | 0.0365 | 2.35E-25 | 724655 | 3.80E-04 | 273 |
| rs6699618 | 1 | 11881441 | c | g | 0.8401 | 0.9115 | 0.041 | 1.68E-109 | 738170 | 6.70E-04 | 497 |
| rs75461554 | 1 | 15810172 | t | c | 0.2007 | -0.3016 | 0.0377 | 1.18E-15 | 738168 | 2.70E-04 | 196 |
| rs1889785 | 1 | 16348729 | a | g | 0.4552 | 0.1782 | 0.0304 | 4.35E-09 | 738170 | 2.40E-04 | 179 |
| rs404100 | 1 | 25366987 | t | c | 0.4513 | 0.1935 | 0.0303 | 1.68E-10 | 737164 | 2.60E-04 | 194 |
| rs34079867 | 1 | 27407850 | t | c | 0.266 | 0.1992 | 0.0354 | 1.78E-08 | 737163 | 2.10E-04 | 158 |
| rs4908348 | 1 | 28706949 | t | g | 0.6944 | 0.2366 | 0.033 | 8.07E-13 | 737164 | 2.80E-04 | 204 |
| rs11210029 | 1 | 41865293 | a | g | 0.6322 | -0.203 | 0.0313 | 8.92E-11 | 737056 | 2.60E-04 | 191 |
| rs1408945 | 1 | 42364877 | t | g | 0.4243 | -0.3196 | 0.0304 | 8.33E-26 | 737056 | 4.30E-04 | 316 |
| rs1209384 | 1 | 43765089 | a | g | 0.3878 | 0.2558 | 0.0313 | 2.85E-16 | 733288 | 3.30E-04 | 245 |
| rs778124 | 1 | 56606206 | a | g | 0.3736 | 0.2965 | 0.0311 | 1.45E-21 | 738170 | 3.80E-04 | 282 |
| rs61772592 | 1 | 56979681 | a | g | 0.8745 | -0.3181 | 0.0455 | 2.86E-12 | 738170 | 1.90E-04 | 142 |
| rs12063372 | 1 | 59621911 | a | g | 0.3846 | 0.1989 | 0.0318 | 3.86E-10 | 737165 | 2.60E-04 | 191 |
| rs12136922 | 1 | 67007389 | a | g | 0.4949 | 0.2027 | 0.0304 | 2.69E-11 | 719076 | 2.80E-04 | 200 |
| rs658780 | 1 | 78555928 | t | g | 0.7447 | -0.2028 | 0.0347 | 5.29E-09 | 737164 | 2.10E-04 | 156 |
| rs786923 | 1 | 89242954 | t | c | 0.6239 | -0.3082 | 0.031 | 2.83E-23 | 737055 | 4.00E-04 | 293 |
| rs7514579 | 1 | 94051350 | a | c | 0.7712 | 0.2243 | 0.0361 | 5.45E-10 | 738168 | 2.20E-04 | 161 |
| rs10776752 | 1 | 113044328 | t | g | 0.0809 | 0.8211 | 0.0576 | 4.61E-46 | 738168 | 3.40E-04 | 248 |
| rs59980837 | 1 | 115827266 | t | g | 0.0178 | 1.0997 | 0.1163 | 3.32E-21 | 734855 | 1.10E-04 | 78 |
| rs11585169 | 1 | 150572037 | a | t | 0.5773 | 0.1796 | 0.0308 | 5.34E-09 | 728445 | 2.40E-04 | 176 |
| rs76719272 | 1 | 156129796 | t | c | 0.1312 | -0.2738 | 0.0461 | 2.97E-09 | 737164 | 1.70E-04 | 127 |
| rs12731646 | 1 | 169090660 | t | c | 0.409 | -0.189 | 0.0307 | 7.21E-10 | 737225 | 2.50E-04 | 185 |
| rs1043069 | 1 | 180859368 | t | g | 0.6156 | 0.234 | 0.0311 | 5.26E-14 | 738169 | 3.00E-04 | 225 |
| rs4651224 | 1 | 184585182 | t | c | 0.4474 | 0.1986 | 0.0306 | 9.00E-11 | 737054 | 2.70E-04 | 199 |
| rs12042924 | 1 | 197297417 | t | c | 0.5284 | -0.1807 | 0.0303 | 2.62E-09 | 738170 | 2.50E-04 | 183 |
| rs11120093 | 1 | 207211326 | t | c | 0.4082 | -0.1792 | 0.0307 | 5.13E-09 | 738170 | 2.40E-04 | 176 |
| rs2724377 | 1 | 207974818 | a | g | 0.5303 | 0.1938 | 0.0301 | 1.29E-10 | 738170 | 2.70E-04 | 196 |
| rs7555285 | 1 | 209970355 | c | g | 0.8011 | 0.2294 | 0.0376 | 1.05E-09 | 738168 | 2.00E-04 | 148 |
| rs68085857 | 1 | 217737629 | t | c | 0.234 | 0.274 | 0.0357 | 1.68E-14 | 738167 | 2.70E-04 | 199 |
| rs72742507 | 1 | 221265336 | t | c | 0.2999 | -0.2053 | 0.0328 | 3.80E-10 | 738170 | 2.40E-04 | 175 |
| rs708117 | 1 | 228199902 | a | g | 0.5203 | 0.2874 | 0.0302 | 1.59E-21 | 738170 | 3.90E-04 | 291 |
| rs699 | 1 | 230845794 | a | g | 0.5928 | -0.3748 | 0.0308 | 5.59E-34 | 721189 | 5.00E-04 | 359 |
| rs1565440 | 1 | 243387788 | a | g | 0.3752 | 0.1746 | 0.0311 | 1.94E-08 | 738169 | 2.20E-04 | 166 |
| rs4926499 | 1 | 249155909 | c | g | 0.8263 | 0.2965 | 0.0438 | 1.33E-11 | 711084 | 2.30E-04 | 166 |
| rs17760259 | 2 | 19744462 | t | c | 0.5724 | -0.2654 | 0.0304 | 2.25E-18 | 738170 | 3.60E-04 | 264 |
| rs2384063 | 2 | 25187115 | t | c | 0.7607 | 0.3266 | 0.0357 | 6.33E-20 | 737164 | 3.30E-04 | 241 |
| rs1275988 | 2 | 26914364 | t | c | 0.6112 | -0.541 | 0.0308 | 4.42E-69 | 738170 | 7.10E-04 | 522 |

| | | | | | | | | | | | |
|---|---|---|---|---|---|---|---|---|---|---|---|
| rs13420463 | 2 | 37517566 | a | g | 0.7734 | 0.3143 | 0.036 | 2.72E-18 | 729450 | 3.00E-04 | 221 |
| rs4952609 | 2 | 40555733 | a | g | 0.7439 | 0.2124 | 0.0347 | 9.60E-10 | 737163 | 2.20E-04 | 164 |
| rs115262049 | 2 | 43196694 | a | t | 0.9132 | 0.5893 | 0.0552 | 1.29E-26 | 738168 | 2.60E-04 | 190 |
| rs12464602 | 2 | 43397614 | a | g | 0.6208 | -0.2437 | 0.0315 | 1.02E-14 | 738170 | 3.20E-04 | 233 |
| rs13016772 | 2 | 55779476 | t | c | 0.7651 | 0.2522 | 0.0355 | 1.23E-12 | 738168 | 2.50E-04 | 184 |
| rs2249105 | 2 | 65287896 | a | g | 0.6321 | 0.2927 | 0.0313 | 7.63E-21 | 729908 | 3.70E-04 | 273 |
| rs10188003 | 2 | 66773469 | t | c | 0.393 | 0.1883 | 0.0307 | 8.80E-10 | 737056 | 2.50E-04 | 182 |
| rs6731373 | 2 | 68503044 | a | g | 0.3492 | 0.1913 | 0.0326 | 4.18E-09 | 737164 | 2.40E-04 | 176 |
| rs6732123 | 2 | 69534650 | c | g | 0.4174 | -0.1737 | 0.0307 | 1.52E-08 | 738169 | 2.30E-04 | 171 |
| rs4577304 | 2 | 73403040 | t | c | 0.5233 | -0.1767 | 0.0302 | 4.99E-09 | 738170 | 2.40E-04 | 179 |
| rs72847885 | 2 | 86326717 | a | g | 0.663 | 0.2413 | 0.0318 | 3.08E-14 | 737055 | 3.00E-04 | 219 |
| rs10207726 | 2 | 112744260 | t | c | 0.296 | -0.2142 | 0.033 | 8.06E-11 | 738169 | 2.50E-04 | 181 |
| rs6737318 | 2 | 114083120 | a | g | 0.7782 | 0.2348 | 0.0364 | 1.13E-10 | 738168 | 2.20E-04 | 165 |
| rs2580350 | 2 | 121996007 | a | g | 0.5609 | 0.1769 | 0.0307 | 8.39E-09 | 737163 | 2.40E-04 | 177 |
| rs17257081 | 2 | 135630498 | a | g | 0.8065 | 0.2274 | 0.0392 | 6.35E-09 | 722234 | 2.00E-04 | 141 |
| rs55944332 | 2 | 145726621 | a | g | 0.7632 | -0.2613 | 0.0355 | 1.79E-13 | 738168 | 2.60E-04 | 192 |
| rs62170470 | 2 | 146989797 | t | c | 0.6017 | 0.1972 | 0.0321 | 7.69E-10 | 728446 | 2.60E-04 | 189 |
| rs62187653 | 2 | 162469128 | t | c | 0.9029 | 0.3286 | 0.0511 | 1.23E-10 | 738168 | 1.60E-04 | 117 |
| rs4667454 | 2 | 164867726 | a | g | 0.6705 | 0.2636 | 0.0322 | 2.63E-16 | 738169 | 3.20E-04 | 236 |
| rs73029563 | 2 | 165008166 | c | g | 0.4549 | -0.514 | 0.0304 | 4.20E-64 | 737165 | 7.00E-04 | 517 |
| rs11694601 | 2 | 174949358 | a | g | 0.5968 | -0.1909 | 0.0309 | 6.41E-10 | 729908 | 2.50E-04 | 184 |
| rs34727427 | 2 | 177016728 | t | c | 0.6832 | -0.2353 | 0.0324 | 4.02E-13 | 738169 | 2.80E-04 | 207 |
| rs1882212 | 2 | 182981968 | a | g | 0.7793 | 0.2753 | 0.0363 | 3.34E-14 | 737054 | 2.60E-04 | 192 |
| rs13412750 | 2 | 191634958 | a | g | 0.2708 | -0.2889 | 0.0341 | 2.33E-17 | 729907 | 3.10E-04 | 229 |
| rs12693982 | 2 | 204085635 | t | c | 0.4024 | 0.2575 | 0.0309 | 7.49E-17 | 735106 | 3.40E-04 | 250 |
| rs3845811 | 2 | 208521512 | c | g | 0.5661 | -0.2942 | 0.0309 | 1.88E-21 | 737165 | 4.00E-04 | 293 |
| rs12694277 | 2 | 213188795 | t | c | 0.2946 | -0.2018 | 0.0335 | 1.80E-09 | 738169 | 2.30E-04 | 170 |
| rs2161967 | 2 | 218680529 | t | g | 0.4279 | 0.2836 | 0.0307 | 2.87E-20 | 738169 | 3.80E-04 | 282 |
| rs3828282 | 2 | 218779144 | c | g | 0.4279 | 0.1857 | 0.0318 | 5.29E-09 | 737165 | 2.50E-04 | 184 |
| rs10804330 | 2 | 227185749 | t | c | 0.5668 | 0.2351 | 0.0306 | 1.62E-14 | 736111 | 3.20E-04 | 234 |
| rs1044822 | 2 | 230629138 | t | c | 0.1482 | -0.248 | 0.0424 | 5.16E-09 | 738169 | 1.70E-04 | 127 |
| rs28365916 | 2 | 231280791 | t | c | 0.4145 | -0.1713 | 0.0306 | 2.24E-08 | 737163 | 2.30E-04 | 169 |
| rs139354822 | 2 | 242344695 | t | c | 0.9704 | 0.6115 | 0.0975 | 3.51E-10 | 720286 | 9.70E-05 | 70 |
| rs9848170 | 3 | 11495983 | c | g | 0.597 | 0.3231 | 0.0307 | 7.01E-26 | 738170 | 4.30E-04 | 316 |
| rs11925504 | 3 | 14943965 | a | g | 0.5721 | -0.2901 | 0.0305 | 1.78E-21 | 737164 | 3.90E-04 | 288 |
| rs189267552 | 3 | 20073193 | a | t | 0.0132 | -0.8664 | 0.139 | 4.55E-10 | 735474 | 6.20E-05 | 46 |
| rs2643826 | 3 | 27562988 | t | c | 0.4505 | 0.4473 | 0.0306 | 1.74E-48 | 738169 | 6.10E-04 | 450 |
| rs68115553 | 3 | 27704702 | a | g | 0.9801 | -0.6445 | 0.1143 | 1.74E-08 | 727329 | 6.90E-05 | 50 |
| rs743395 | 3 | 37598382 | t | c | 0.3834 | 0.2597 | 0.0317 | 2.55E-16 | 737165 | 3.40E-04 | 249 |
| rs6788984 | 3 | 41107173 | a | g | 0.8563 | 0.2999 | 0.0432 | 3.81E-12 | 738169 | 2.00E-04 | 150 |
| rs1052501 | 3 | 41925398 | t | c | 0.8329 | 0.2262 | 0.0412 | 4.14E-08 | 729908 | 1.70E-04 | 126 |
| rs6771917 | 3 | 48108442 | t | c | 0.2477 | -0.3793 | 0.0355 | 1.39E-26 | 738168 | 3.90E-04 | 287 |
| rs7615099 | 3 | 53143901 | a | g | 0.6675 | 0.1891 | 0.0321 | 3.90E-09 | 737163 | 2.30E-04 | 170 |
| rs6445583 | 3 | 53562894 | a | g | 0.7465 | 0.2774 | 0.0349 | 1.90E-15 | 738167 | 2.90E-04 | 213 |
| rs3772219 | 3 | 56771251 | a | c | 0.6824 | 0.2733 | 0.0324 | 3.10E-17 | 738170 | 3.30E-04 | 240 |
| rs7618284 | 3 | 66422246 | c | g | 0.3394 | -0.1891 | 0.0331 | 1.10E-08 | 737164 | 2.30E-04 | 172 |

| | | | | | | | | | | | | |
|---|---|---|---|---|---|---|---|---|---|---|---|---|
| rs4499560 | 3 | 70920485 | a | t | 0.3171 | -0.2199 | 0.0326 | 1.46E-11 | 737162 | 2.60E-04 | 193 |
| rs9857362 | 3 | 74710462 | a | c | 0.5291 | 0.1727 | 0.0306 | 1.62E-08 | 721189 | 2.40E-04 | 171 |
| rs1375564 | 3 | 85656311 | t | c | 0.6395 | 0.2579 | 0.0315 | 2.84E-16 | 736109 | 3.30E-04 | 241 |
| rs12637573 | 3 | 121682388 | a | g | 0.4718 | -0.1731 | 0.0302 | 9.95E-09 | 737164 | 2.40E-04 | 175 |
| rs6438857 | 3 | 124557643 | t | c | 0.5774 | 0.2736 | 0.0305 | 3.13E-19 | 738170 | 3.70E-04 | 271 |
| rs9880098 | 3 | 133949366 | a | g | 0.3946 | 0.3081 | 0.0308 | 1.59E-23 | 738169 | 4.00E-04 | 299 |
| rs1199330 | 3 | 138101529 | a | g | 0.8824 | -0.2654 | 0.047 | 1.65E-08 | 729906 | 1.50E-04 | 111 |
| rs9876694 | 3 | 141152017 | t | c | 0.0584 | 0.4713 | 0.0651 | 4.64E-13 | 737055 | 1.40E-04 | 105 |
| rs4408839 | 3 | 153729768 | a | g | 0.7433 | -0.2301 | 0.0345 | 2.43E-11 | 738168 | 2.40E-04 | 178 |
| rs79539362 | 3 | 154680449 | t | c | 0.8992 | 0.4003 | 0.0504 | 2.09E-15 | 738166 | 2.00E-04 | 147 |
| rs17684859 | 3 | 158213841 | t | c | 0.7335 | -0.2241 | 0.034 | 4.24E-11 | 738170 | 2.40E-04 | 178 |
| rs3980686 | 3 | 168697602 | t | g | 0.1075 | -0.4998 | 0.0487 | 1.03E-24 | 738167 | 2.60E-04 | 195 |
| rs1290784 | 3 | 169096900 | t | c | 0.4483 | 0.4124 | 0.0303 | 2.97E-42 | 736111 | 5.60E-04 | 413 |
| rs2111557 | 3 | 169325621 | t | c | 0.4675 | 0.1764 | 0.0302 | 5.22E-09 | 738170 | 2.40E-04 | 178 |
| rs4955575 | 3 | 169534538 | a | c | 0.7461 | 0.2158 | 0.0348 | 5.63E-10 | 738169 | 2.20E-04 | 166 |
| rs262986 | 3 | 183435713 | a | g | 0.4704 | -0.2371 | 0.0305 | 7.67E-15 | 738170 | 3.20E-04 | 240 |
| rs13091418 | 3 | 185329756 | c | g | 0.6659 | -0.2234 | 0.0325 | 6.15E-12 | 737163 | 2.70E-04 | 201 |
| rs9869437 | 3 | 196228360 | a | c | 0.3523 | -0.2001 | 0.0318 | 3.22E-10 | 737165 | 2.50E-04 | 185 |
| rs34535756 | 4 | 2246927 | t | c | 0.0394 | 0.478 | 0.0786 | 1.18E-09 | 737053 | 9.90E-05 | 73 |
| rs1290933 | 4 | 2668217 | a | c | 0.6919 | -0.2847 | 0.0327 | 3.17E-18 | 738170 | 3.30E-04 | 246 |
| rs2498323 | 4 | 3451109 | a | g | 0.098 | 0.3171 | 0.0517 | 8.52E-10 | 736057 | 1.50E-04 | 113 |
| rs2610990 | 4 | 18008232 | a | g | 0.2641 | -0.2903 | 0.0343 | 2.86E-17 | 735152 | 3.10E-04 | 228 |
| rs55924432 | 4 | 26812737 | t | c | 0.401 | 0.2651 | 0.0317 | 5.70E-17 | 734146 | 3.50E-04 | 257 |
| rs2291434 | 4 | 38387244 | t | g | 0.5335 | -0.2622 | 0.0303 | 5.10E-18 | 735151 | 3.60E-04 | 264 |
| rs12511987 | 4 | 46595623 | t | g | 0.8226 | -0.2329 | 0.0399 | 5.39E-09 | 738169 | 1.90E-04 | 138 |
| rs62309747 | 4 | 48713862 | a | g | 0.4734 | -0.2244 | 0.0304 | 1.59E-13 | 737165 | 3.10E-04 | 227 |
| rs60991988 | 4 | 54801228 | t | g | 0.8931 | 0.3789 | 0.0498 | 2.82E-14 | 729449 | 2.00E-04 | 145 |
| rs13107261 | 4 | 63768826 | a | g | 0.3687 | -0.1778 | 0.0314 | 1.57E-08 | 729906 | 2.30E-04 | 166 |
| rs10008637 | 4 | 77414144 | t | c | 0.5405 | 0.2157 | 0.0302 | 9.24E-13 | 738169 | 2.90E-04 | 217 |
| rs12509595 | 4 | 81182554 | t | c | 0.7077 | -0.8367 | 0.0334 | 2.55E-138 | 737164 | 9.50E-04 | 702 |
| rs60909079 | 4 | 83830244 | c | g | 0.2492 | -0.2114 | 0.0351 | 1.73E-09 | 735151 | 2.20E-04 | 160 |
| rs17010957 | 4 | 86719165 | t | c | 0.8537 | -0.534 | 0.043 | 1.78E-35 | 735152 | 3.70E-04 | 270 |
| rs10028284 | 4 | 89752913 | a | t | 0.8184 | 0.2937 | 0.0398 | 1.69E-13 | 734146 | 2.40E-04 | 176 |
| rs13107325 | 4 | 103188709 | t | c | 0.0739 | -0.9086 | 0.0592 | 4.22E-53 | 735152 | 3.40E-04 | 251 |
| rs11097909 | 4 | 106911321 | t | c | 0.1472 | -0.3628 | 0.043 | 3.35E-17 | 735150 | 2.50E-04 | 184 |
| rs1493132 | 4 | 108861082 | t | c | 0.6603 | -0.1766 | 0.0318 | 2.73E-08 | 735150 | 2.20E-04 | 160 |
| rs1814951 | 4 | 111408718 | a | g | 0.8785 | -0.3231 | 0.0466 | 3.91E-12 | 735150 | 1.90E-04 | 139 |
| rs4834792 | 4 | 120555696 | a | t | 0.4796 | 0.1973 | 0.0303 | 7.24E-11 | 735152 | 2.70E-04 | 199 |
| rs7439567 | 4 | 138464842 | t | c | 0.4106 | 0.2537 | 0.0309 | 2.31E-16 | 734147 | 3.40E-04 | 248 |
| rs72719160 | 4 | 144051276 | a | t | 0.6829 | -0.2243 | 0.0324 | 4.34E-12 | 735151 | 2.70E-04 | 196 |
| rs2353940 | 4 | 145740898 | t | c | 0.7507 | -0.2075 | 0.0358 | 6.85E-09 | 732820 | 2.10E-04 | 156 |
| rs73855810 | 4 | 148383424 | a | g | 0.1406 | 0.2732 | 0.0434 | 3.04E-10 | 738169 | 1.80E-04 | 134 |
| rs7683728 | 4 | 156402654 | t | c | 0.5312 | -0.3654 | 0.0304 | 2.43E-33 | 728337 | 5.00E-04 | 364 |
| rs12643599 | 4 | 156639846 | a | g | 0.6395 | 0.3134 | 0.0313 | 1.23E-23 | 738170 | 4.00E-04 | 293 |
| rs17035181 | 4 | 157678511 | t | g | 0.8552 | 0.3074 | 0.0429 | 7.61E-13 | 737055 | 2.10E-04 | 154 |
| rs869396 | 4 | 169688000 | a | c | 0.4659 | -0.2115 | 0.0305 | 4.12E-12 | 737165 | 2.90E-04 | 213 |

| | | | | | | | | | | | |
|---|---|---|---|---|---|---|---|---|---|---|---|
| rs4957026 | 5 | 361148 | a | g | 0.3399 | 0.1982 | 0.0323 | 8.12E-10 | 735051 | 2.40E-04 | 180 |
| rs10069690 | 5 | 1279790 | t | c | 0.2582 | 0.3098 | 0.0369 | 4.47E-17 | 707524 | 3.30E-04 | 231 |
| rs7725413 | 5 | 15695987 | t | c | 0.7699 | -0.1985 | 0.0359 | 3.07E-08 | 737054 | 1.90E-04 | 143 |
| rs12656497 | 5 | 32831939 | t | c | 0.4034 | -0.6382 | 0.0307 | 7.14E-96 | 736111 | 8.40E-04 | 622 |
| rs10941043 | 5 | 33194751 | t | g | 0.7098 | -0.2585 | 0.0332 | 6.42E-15 | 738168 | 2.90E-04 | 216 |
| rs2113077 | 5 | 50799442 | a | g | 0.4303 | 0.2097 | 0.0305 | 6.09E-12 | 737056 | 2.80E-04 | 208 |
| rs1694068 | 5 | 53283630 | a | t | 0.6139 | 0.2657 | 0.0311 | 1.18E-17 | 738169 | 3.50E-04 | 256 |
| rs13179413 | 5 | 55868097 | t | c | 0.2819 | 0.2238 | 0.0347 | 1.08E-10 | 737164 | 2.50E-04 | 184 |
| rs34496659 | 5 | 61798934 | a | g | 0.0702 | 0.4545 | 0.0616 | 1.54E-13 | 738167 | 1.60E-04 | 120 |
| rs6870654 | 5 | 63831964 | t | c | 0.7454 | 0.2136 | 0.0347 | 7.58E-10 | 729908 | 2.20E-04 | 163 |
| rs4286632 | 5 | 66291370 | a | g | 0.7306 | 0.211 | 0.0343 | 7.64E-10 | 738169 | 2.30E-04 | 169 |
| rs7703560 | 5 | 67678506 | a | g | 0.7002 | -0.2246 | 0.0333 | 1.51E-11 | 729449 | 2.60E-04 | 189 |
| rs246973 | 5 | 68007803 | t | c | 0.2882 | 0.2479 | 0.0335 | 1.45E-13 | 737164 | 2.80E-04 | 206 |
| rs6452769 | 5 | 87389027 | a | g | 0.2053 | -0.3143 | 0.0377 | 7.82E-17 | 737165 | 2.80E-04 | 208 |
| rs76443575 | 5 | 96211594 | c | g | 0.0359 | -0.5233 | 0.0816 | 1.40E-10 | 737711 | 1.00E-04 | 73 |
| rs1871190 | 5 | 97953719 | t | g | 0.3349 | 0.1954 | 0.0324 | 1.66E-09 | 737164 | 2.40E-04 | 176 |
| rs11241313 | 5 | 114428167 | t | c | 0.3112 | -0.2071 | 0.0326 | 2.23E-10 | 738169 | 2.40E-04 | 180 |
| rs1624822 | 5 | 122475437 | t | c | 0.6195 | -0.3362 | 0.0312 | 5.12E-27 | 738168 | 4.40E-04 | 322 |
| rs9327297 | 5 | 122835051 | c | g | 0.6676 | 0.2747 | 0.0319 | 8.07E-18 | 738169 | 3.40E-04 | 247 |
| rs758180 | 5 | 127354423 | a | t | 0.2245 | 0.2084 | 0.0367 | 1.35E-08 | 736049 | 2.00E-04 | 147 |
| rs6892983 | 5 | 127845030 | a | c | 0.4022 | 0.3427 | 0.0307 | 7.11E-29 | 737164 | 4.50E-04 | 334 |
| rs702395 | 5 | 140086677 | t | c | 0.4369 | 0.2318 | 0.0305 | 3.24E-14 | 737165 | 3.10E-04 | 231 |
| rs2913920 | 5 | 141726983 | t | c | 0.765 | 0.2418 | 0.0359 | 1.62E-11 | 735150 | 2.40E-04 | 176 |
| rs1957563 | 5 | 157474590 | t | c | 0.265 | 0.3629 | 0.0342 | 2.32E-26 | 735150 | 3.90E-04 | 286 |
| rs11960210 | 5 | 157817634 | t | c | 0.6245 | 0.4727 | 0.0313 | 1.25E-51 | 726890 | 6.10E-04 | 443 |
| rs13358657 | 5 | 157938070 | a | g | 0.8668 | -0.388 | 0.0445 | 2.95E-18 | 735151 | 2.50E-04 | 181 |
| rs3860770 | 5 | 173301427 | a | g | 0.2916 | -0.2663 | 0.0333 | 1.20E-15 | 733093 | 3.00E-04 | 222 |
| rs12153395 | 5 | 179411477 | a | g | 0.1147 | -0.3303 | 0.0486 | 1.07E-11 | 734145 | 1.80E-04 | 135 |
| rs2745599 | 6 | 1613686 | a | g | 0.552 | 0.2164 | 0.0317 | 8.96E-12 | 728445 | 2.90E-04 | 214 |
| rs1575290 | 6 | 7715689 | t | c | 0.4733 | 0.1973 | 0.0301 | 5.59E-11 | 738170 | 2.70E-04 | 200 |
| rs1630736 | 6 | 12295987 | t | c | 0.465 | -0.1706 | 0.0309 | 3.52E-08 | 737165 | 2.30E-04 | 172 |
| rs9349379 | 6 | 12903957 | a | g | 0.593 | 0.2664 | 0.0312 | 1.31E-17 | 737164 | 3.50E-04 | 261 |
| rs9368222 | 6 | 20686996 | a | c | 0.2688 | 0.2281 | 0.0339 | 1.84E-11 | 738169 | 2.50E-04 | 182 |
| rs9393231 | 6 | 22123695 | a | c | 0.4924 | -0.2148 | 0.0309 | 3.39E-12 | 737164 | 3.00E-04 | 218 |
| rs7753826 | 6 | 26042239 | a | t | 0.1899 | 0.4276 | 0.0385 | 9.96E-29 | 738170 | 3.60E-04 | 267 |
| rs2596498 | 6 | 31322688 | t | c | 0.6379 | -0.233 | 0.0337 | 4.90E-12 | 692081 | 3.00E-04 | 205 |
| rs3132442 | 6 | 31839494 | t | c | 0.5199 | 0.3931 | 0.0304 | 2.65E-38 | 727903 | 5.40E-04 | 393 |
| rs7763558 | 6 | 43349215 | a | g | 0.3241 | 0.3363 | 0.0321 | 1.17E-25 | 738170 | 4.00E-04 | 299 |
| rs11967262 | 6 | 43760327 | c | g | 0.5132 | -0.1715 | 0.0311 | 3.43E-08 | 730376 | 2.40E-04 | 172 |
| rs78648104 | 6 | 50683009 | t | c | 0.9075 | -0.4287 | 0.0541 | 2.37E-15 | 735105 | 2.00E-04 | 145 |
| rs1984195 | 6 | 79657391 | a | g | 0.4887 | 0.2409 | 0.0303 | 1.77E-15 | 729908 | 3.30E-04 | 242 |
| rs9361836 | 6 | 82235408 | t | c | 0.3172 | 0.2196 | 0.0324 | 1.25E-11 | 738170 | 2.60E-04 | 193 |
| rs6921291 | 6 | 97066242 | t | c | 0.1907 | 0.3575 | 0.0385 | 1.58E-20 | 738169 | 3.00E-04 | 224 |
| rs9486916 | 6 | 109013930 | t | c | 0.1979 | 0.2657 | 0.0385 | 5.42E-12 | 729449 | 2.30E-04 | 169 |
| rs961764 | 6 | 117522156 | c | g | 0.4254 | -0.1909 | 0.0305 | 3.75E-10 | 738170 | 2.60E-04 | 189 |
| rs10782230 | 6 | 126228512 | a | g | 0.4845 | 0.2106 | 0.0302 | 2.91E-12 | 738169 | 2.90E-04 | 214 |

| | | | | | | | | | | | | |
|---|---|---|---|---|---|---|---|---|---|---|---|---|
| rs9401913 | 6 | 127159982 | a | g | 0.4387 | 0.5202 | 0.0305 | 3.66E-65 | 738170 | 7.00E-04 | 520 |
| rs9285476 | 6 | 134159976 | c | g | 0.7071 | 0.1844 | 0.0333 | 3.07E-08 | 738169 | 2.10E-04 | 155 |
| rs13204703 | 6 | 140692862 | t | c | 0.7511 | 0.1967 | 0.035 | 1.94E-08 | 738168 | 2.00E-04 | 149 |
| rs8180684 | 6 | 143200936 | t | c | 0.2896 | 0.2134 | 0.0335 | 1.80E-10 | 737164 | 2.40E-04 | 178 |
| rs7765526 | 6 | 147713764 | a | g | 0.4633 | 0.201 | 0.0307 | 5.88E-11 | 737165 | 2.70E-04 | 203 |
| rs17080102 | 6 | 151004770 | c | g | 0.0694 | -0.8085 | 0.0594 | 3.52E-42 | 738169 | 2.90E-04 | 212 |
| rs1293969 | 6 | 151959945 | t | c | 0.7484 | -0.1988 | 0.0347 | 1.03E-08 | 738169 | 2.10E-04 | 152 |
| rs509833 | 6 | 159711515 | a | g | 0.1386 | 0.329 | 0.044 | 7.08E-14 | 737164 | 2.20E-04 | 159 |
| rs12661036 | 6 | 163737476 | t | c | 0.775 | -0.2104 | 0.0374 | 1.82E-08 | 737165 | 2.00E-04 | 149 |
| rs7744902 | 6 | 166176722 | a | g | 0.0766 | -0.4088 | 0.0593 | 5.64E-12 | 713753 | 1.60E-04 | 113 |
| rs6978112 | 7 | 1966841 | t | c | 0.4112 | 0.2286 | 0.0309 | 1.34E-13 | 735052 | 3.00E-04 | 224 |
| rs10282122 | 7 | 2529623 | t | c | 0.6684 | -0.302 | 0.0327 | 2.46E-20 | 735052 | 3.70E-04 | 271 |
| rs73049928 | 7 | 4669949 | a | g | 0.8061 | -0.2382 | 0.0392 | 1.20E-09 | 737053 | 2.00E-04 | 151 |
| rs3807925 | 7 | 18543250 | a | g | 0.6496 | -0.1859 | 0.0319 | 5.39E-09 | 736051 | 2.30E-04 | 171 |
| rs28688791 | 7 | 19039605 | t | c | 0.8018 | -0.3222 | 0.038 | 2.34E-17 | 738167 | 2.80E-04 | 208 |
| rs112509803 | 7 | 24735004 | c | g | 0.1138 | -0.2641 | 0.0477 | 3.18E-08 | 738168 | 1.50E-04 | 108 |
| rs3735533 | 7 | 27245893 | t | c | 0.0743 | -0.91 | 0.0577 | 5.29E-56 | 737165 | 3.40E-04 | 254 |
| rs6961048 | 7 | 27328187 | c | g | 0.896 | -0.5304 | 0.0497 | 1.43E-26 | 734292 | 2.70E-04 | 200 |
| rs11977526 | 7 | 46008110 | a | g | 0.4009 | -0.3213 | 0.0312 | 6.62E-25 | 732149 | 4.20E-04 | 311 |
| rs12668436 | 7 | 47548893 | t | c | 0.7541 | -0.2151 | 0.035 | 7.88E-10 | 738168 | 2.20E-04 | 162 |
| rs848445 | 7 | 77572461 | t | c | 0.2851 | -0.2025 | 0.0339 | 2.28E-09 | 738169 | 2.30E-04 | 168 |
| rs67617547 | 7 | 90297177 | c | g | 0.6697 | 0.1799 | 0.0322 | 2.39E-08 | 738168 | 2.20E-04 | 162 |
| rs42032 | 7 | 92237426 | a | g | 0.2641 | -0.3231 | 0.0345 | 7.39E-21 | 736050 | 3.50E-04 | 254 |
| rs2392929 | 7 | 106414069 | t | g | 0.7973 | -0.7507 | 0.0379 | 1.96E-87 | 737165 | 6.70E-04 | 492 |
| rs34072724 | 7 | 130432469 | a | g | 0.4889 | -0.2422 | 0.0303 | 1.37E-15 | 736111 | 3.30E-04 | 245 |
| rs35680304 | 7 | 130973495 | t | c | 0.5929 | 0.2694 | 0.031 | 3.76E-18 | 736051 | 3.60E-04 | 263 |
| rs75672964 | 7 | 131321010 | t | c | 0.0418 | 0.5885 | 0.0839 | 2.35E-12 | 712274 | 1.30E-04 | 92 |
| rs6957161 | 7 | 131361319 | a | g | 0.2618 | 0.2064 | 0.0345 | 2.20E-09 | 735052 | 2.20E-04 | 161 |
| rs73727605 | 7 | 149474622 | a | g | 0.0663 | 0.3616 | 0.0623 | 6.60E-09 | 726466 | 1.20E-04 | 89 |
| rs3918226 | 7 | 150690176 | t | c | 0.0811 | 0.664 | 0.0575 | 8.46E-31 | 731379 | 2.70E-04 | 199 |
| rs10224210 | 7 | 151413194 | t | c | 0.7211 | -0.3831 | 0.034 | 1.60E-29 | 738170 | 4.20E-04 | 313 |
| rs1870735 | 7 | 155744303 | c | g | 0.4531 | 0.206 | 0.0311 | 3.61E-11 | 737163 | 2.80E-04 | 207 |
| rs71499040 | 8 | 1711918 | c | g | 0.7076 | 0.2215 | 0.0338 | 5.63E-11 | 732671 | 2.50E-04 | 185 |
| rs1821002 | 8 | 10640065 | c | g | 0.4108 | 0.3794 | 0.0307 | 5.19E-35 | 738169 | 5.00E-04 | 373 |
| rs7844887 | 8 | 23402482 | a | g | 0.2208 | 0.2662 | 0.0363 | 2.40E-13 | 738169 | 2.50E-04 | 186 |
| rs7821832 | 8 | 25889446 | t | g | 0.7447 | 0.4222 | 0.0348 | 6.67E-34 | 729908 | 4.40E-04 | 322 |
| rs77375686 | 8 | 26043622 | a | g | 0.8883 | -0.3467 | 0.0485 | 8.38E-13 | 738166 | 1.90E-04 | 140 |
| rs1906672 | 8 | 38130025 | a | g | 0.2319 | 0.2966 | 0.0358 | 1.20E-16 | 738169 | 2.90E-04 | 214 |
| rs4873492 | 8 | 51947549 | t | c | 0.1724 | 0.3431 | 0.0403 | 1.61E-17 | 738170 | 2.70E-04 | 199 |
| rs2354862 | 8 | 64501744 | a | c | 0.6407 | 0.2507 | 0.0317 | 2.42E-15 | 729451 | 3.20E-04 | 231 |
| rs13253358 | 8 | 68920135 | t | c | 0.2979 | 0.2127 | 0.033 | 1.13E-10 | 738169 | 2.40E-04 | 181 |
| rs2126474 | 8 | 76878957 | t | g | 0.4125 | -0.2601 | 0.0306 | 1.87E-17 | 737054 | 3.50E-04 | 255 |
| rs9918876 | 8 | 77681097 | a | c | 0.1037 | -0.2975 | 0.0498 | 2.28E-09 | 738167 | 1.50E-04 | 112 |
| rs148401029 | 8 | 81386066 | a | c | 0.0352 | -0.4623 | 0.0848 | 4.97E-08 | 738168 | 8.60E-05 | 64 |
| rs10091532 | 8 | 82853793 | a | c | 0.4168 | -0.2067 | 0.0305 | 1.33E-11 | 738170 | 2.80E-04 | 204 |
| rs843093 | 8 | 92528310 | a | g | 0.7088 | -0.2085 | 0.0338 | 6.95E-10 | 737165 | 2.40E-04 | 174 |

| | | | | | | | | | | | |
|---|---|---|---|---|---|---|---|---|---|---|---|
| rs2613203 | 8 | 95253197 | a | t | 0.8148 | -0.2681 | 0.0389 | 5.81E-12 | 738168 | 2.20E-04 | 164 |
| rs79069610 | 8 | 105921209 | t | c | 0.95 | -0.4005 | 0.0727 | 3.68E-08 | 738168 | 1.00E-04 | 77 |
| rs35783704 | 8 | 105966258 | a | g | 0.1042 | -0.4619 | 0.0507 | 8.81E-20 | 737055 | 2.40E-04 | 175 |
| rs7830607 | 8 | 110097287 | a | g | 0.3046 | -0.206 | 0.0327 | 3.09E-10 | 738170 | 2.40E-04 | 177 |
| rs2470004 | 8 | 120358445 | t | c | 0.8175 | -0.3454 | 0.0392 | 1.28E-18 | 738168 | 2.80E-04 | 209 |
| rs6986368 | 8 | 126513197 | a | t | 0.6726 | -0.2132 | 0.0329 | 9.62E-11 | 726538 | 2.60E-04 | 188 |
| rs2608029 | 8 | 129170126 | c | g | 0.6653 | 0.1805 | 0.032 | 1.61E-08 | 737164 | 2.20E-04 | 163 |
| rs4260863 | 8 | 129386613 | c | g | 0.6163 | 0.1911 | 0.0314 | 1.17E-09 | 725573 | 2.50E-04 | 180 |
| rs7012866 | 8 | 135616959 | t | g | 0.4991 | -0.2325 | 0.0301 | 1.21E-14 | 737165 | 3.20E-04 | 236 |
| rs4440615 | 8 | 141057641 | a | g | 0.6321 | -0.2201 | 0.0312 | 1.87E-12 | 738170 | 2.80E-04 | 208 |
| rs4961293 | 8 | 141812374 | t | c | 0.4513 | 0.2268 | 0.0303 | 7.35E-14 | 738169 | 3.10E-04 | 228 |
| rs7463212 | 8 | 143991858 | a | t | 0.5445 | -0.2753 | 0.0305 | 1.81E-19 | 728903 | 3.80E-04 | 274 |
| rs60191654 | 9 | 753648 | a | g | 0.8118 | -0.2382 | 0.0385 | 5.88E-10 | 745818 | 2.00E-04 | 149 |
| rs927315 | 9 | 4117713 | t | c | 0.4713 | 0.1689 | 0.0303 | 2.44E-08 | 744705 | 2.30E-04 | 172 |
| rs1332813 | 9 | 9350706 | t | c | 0.3514 | 0.2203 | 0.0314 | 2.32E-12 | 745819 | 2.80E-04 | 206 |
| rs9886665 | 9 | 22942770 | t | c | 0.2671 | 0.2048 | 0.0343 | 2.47E-09 | 736095 | 2.20E-04 | 162 |
| rs4553000 | 9 | 34223553 | t | c | 0.5141 | -0.2035 | 0.03 | 1.09E-11 | 745820 | 2.80E-04 | 208 |
| rs76452347 | 9 | 35906471 | t | c | 0.205 | -0.2974 | 0.0397 | 7.13E-14 | 743700 | 2.70E-04 | 198 |
| rs1410222 | 9 | 77239540 | t | c | 0.8166 | 0.2173 | 0.0388 | 2.17E-08 | 745818 | 1.80E-04 | 133 |
| rs7045409 | 9 | 95201540 | a | t | 0.3669 | -0.1862 | 0.0313 | 2.55E-09 | 741943 | 2.40E-04 | 176 |
| rs10980408 | 9 | 113249071 | t | c | 0.9641 | -0.7606 | 0.0827 | 3.83E-20 | 745817 | 1.40E-04 | 108 |
| rs7026176 | 9 | 116670743 | t | g | 0.5118 | -0.1869 | 0.0299 | 4.01E-10 | 745820 | 2.60E-04 | 192 |
| rs34025993 | 9 | 123516572 | a | g | 0.414 | 0.223 | 0.0308 | 4.71E-13 | 744814 | 3.00E-04 | 222 |
| rs4838021 | 9 | 125657099 | t | c | 0.1289 | -0.3009 | 0.0453 | 3.13E-11 | 737097 | 1.90E-04 | 137 |
| rs13289468 | 9 | 128180332 | a | c | 0.5743 | 0.2488 | 0.0306 | 3.93E-16 | 744815 | 3.30E-04 | 249 |
| rs6271 | 9 | 136522274 | t | c | 0.0735 | -0.5547 | 0.0611 | 1.18E-19 | 736797 | 2.10E-04 | 153 |
| rs11145807 | 9 | 139520789 | a | g | 0.4057 | 0.2135 | 0.0322 | 3.54E-11 | 721505 | 2.80E-04 | 204 |
| rs11252324 | 10 | 4124568 | t | g | 0.0771 | -0.4164 | 0.0573 | 3.61E-13 | 738167 | 1.60E-04 | 120 |
| rs1623474 | 10 | 18471794 | t | c | 0.3303 | 0.3827 | 0.0321 | 7.66E-33 | 738167 | 4.70E-04 | 344 |
| rs12258967 | 10 | 18727959 | c | g | 0.7047 | 0.6327 | 0.0337 | 1.08E-78 | 737165 | 7.20E-04 | 534 |
| rs3802517 | 10 | 28233469 | a | t | 0.4618 | 0.2527 | 0.0301 | 4.65E-17 | 738169 | 3.50E-04 | 255 |
| rs12264186 | 10 | 32289986 | t | c | 0.1871 | 0.2135 | 0.0387 | 3.58E-08 | 738168 | 1.80E-04 | 132 |
| rs4948643 | 10 | 45379759 | t | c | 0.2819 | 0.2258 | 0.0338 | 2.40E-11 | 737164 | 2.50E-04 | 185 |
| rs34130368 | 10 | 48411796 | t | g | 0.117 | -0.3016 | 0.0497 | 1.28E-09 | 736051 | 1.70E-04 | 126 |
| rs4245599 | 10 | 60365755 | a | g | 0.4584 | -0.1794 | 0.0305 | 4.04E-09 | 738169 | 2.40E-04 | 181 |
| rs57946343 | 10 | 63499951 | t | c | 0.8527 | 0.716 | 0.0426 | 2.10E-63 | 736110 | 4.90E-04 | 364 |
| rs2236295 | 10 | 64564892 | t | g | 0.3978 | -0.3028 | 0.0309 | 1.05E-22 | 738169 | 4.00E-04 | 294 |
| rs2177843 | 10 | 75409877 | t | c | 0.1505 | 0.4394 | 0.0432 | 2.80E-24 | 738168 | 3.10E-04 | 228 |
| rs10749572 | 10 | 82136664 | t | g | 0.5444 | -0.203 | 0.0302 | 1.88E-11 | 738170 | 2.80E-04 | 204 |
| rs111866816 | 10 | 94441507 | t | c | 0.0709 | 0.3569 | 0.0597 | 2.29E-09 | 737163 | 1.30E-04 | 95 |
| rs2689690 | 10 | 95899706 | t | c | 0.3678 | -0.2702 | 0.0316 | 1.15E-17 | 727391 | 3.50E-04 | 251 |
| rs2274224 | 10 | 96039597 | c | g | 0.4324 | -0.4517 | 0.0304 | 5.99E-50 | 737056 | 6.10E-04 | 449 |
| rs1006545 | 10 | 102553647 | t | g | 0.8872 | 0.6846 | 0.048 | 3.50E-46 | 738169 | 3.80E-04 | 278 |
| rs11191580 | 10 | 104906211 | t | c | 0.9176 | 1.0995 | 0.055 | 7.74E-89 | 738169 | 4.60E-04 | 337 |
| rs117464403 | 10 | 107158054 | a | g | 0.0183 | 0.864 | 0.1199 | 5.80E-13 | 727329 | 8.50E-05 | 62 |
| rs12255372 | 10 | 114808902 | t | g | 0.2883 | 0.2358 | 0.0335 | 1.94E-12 | 729908 | 2.70E-04 | 194 |

| | | | | | | | | | | | |
|---|---|---|---|---|---|---|---|---|---|---|---|
| rs1801253 | 10 | 115805056 | c | g | 0.7338 | 0.4626 | 0.0344 | 2.84E-41 | 738169 | 5.00E-04 | 367 |
| rs72842207 | 10 | 121433675 | t | c | 0.2144 | -0.203 | 0.0367 | 3.14E-08 | 738167 | 1.90E-04 | 139 |
| rs11592107 | 10 | 122968964 | a | g | 0.3096 | 0.3024 | 0.0326 | 1.55E-20 | 738169 | 3.60E-04 | 262 |
| rs7093894 | 10 | 124234880 | a | c | 0.1512 | 0.236 | 0.0427 | 3.16E-08 | 738169 | 1.70E-04 | 123 |
| rs7912283 | 10 | 133773019 | a | g | 0.6468 | -0.2144 | 0.0322 | 2.94E-11 | 736050 | 2.70E-04 | 198 |
| rs1133400 | 10 | 134459388 | a | g | 0.786 | -0.2975 | 0.0376 | 2.53E-15 | 722557 | 2.80E-04 | 199 |
| rs569550 | 11 | 1887068 | t | g | 0.6037 | -0.5765 | 0.0318 | 1.33E-73 | 718172 | 7.60E-04 | 545 |
| rs74048190 | 11 | 2114221 | t | c | 0.9522 | -0.4404 | 0.0757 | 6.07E-09 | 718169 | 1.10E-04 | 79 |
| rs360153 | 11 | 9762274 | t | c | 0.4166 | -0.3445 | 0.0306 | 1.73E-29 | 738170 | 4.60E-04 | 340 |
| rs2014408 | 11 | 16365282 | t | c | 0.2087 | 0.5169 | 0.0373 | 1.26E-43 | 738168 | 4.70E-04 | 347 |
| rs7926335 | 11 | 16917869 | t | c | 0.2691 | 0.3135 | 0.0339 | 2.52E-20 | 736109 | 3.40E-04 | 250 |
| rs17762 | 11 | 22492454 | a | g | 0.0777 | 0.4117 | 0.0571 | 5.60E-13 | 738167 | 1.60E-04 | 120 |
| rs1382472 | 11 | 27273967 | a | g | 0.4041 | -0.1917 | 0.0307 | 4.47E-10 | 738170 | 2.50E-04 | 187 |
| rs871004 | 11 | 28512458 | a | g | 0.3481 | 0.2336 | 0.0317 | 1.65E-13 | 738170 | 2.90E-04 | 215 |
| rs1340030 | 11 | 30182068 | t | c | 0.6345 | 0.1936 | 0.0312 | 5.77E-10 | 738169 | 2.50E-04 | 182 |
| rs11604310 | 11 | 45351420 | t | c | 0.1655 | -0.2778 | 0.0411 | 1.46E-11 | 738168 | 2.10E-04 | 156 |
| rs7107356 | 11 | 47676170 | a | g | 0.4959 | -0.4598 | 0.0301 | 1.63E-52 | 738170 | 6.30E-04 | 467 |
| rs2904315 | 11 | 48109948 | a | g | 0.3131 | -0.2081 | 0.0325 | 1.58E-10 | 738167 | 2.50E-04 | 182 |
| rs7125196 | 11 | 61272565 | t | c | 0.8817 | 0.4422 | 0.0472 | 7.31E-21 | 736058 | 2.50E-04 | 187 |
| rs2306363 | 11 | 65405600 | t | g | 0.2045 | -0.4358 | 0.0376 | 5.24E-31 | 738167 | 3.90E-04 | 288 |
| rs7395791 | 11 | 69262916 | a | g | 0.4419 | -0.2162 | 0.0308 | 2.19E-12 | 738170 | 2.90E-04 | 216 |
| rs10501410 | 11 | 72088806 | a | g | 0.0692 | 0.4122 | 0.0607 | 1.10E-11 | 738166 | 1.50E-04 | 108 |
| rs7927515 | 11 | 76125330 | a | c | 0.3459 | 0.2271 | 0.0319 | 1.05E-12 | 729908 | 2.80E-04 | 206 |
| rs2289124 | 11 | 89224477 | a | g | 0.1673 | -0.308 | 0.0415 | 1.14E-13 | 737164 | 2.40E-04 | 174 |
| rs67885470 | 11 | 99998431 | t | c | 0.2094 | -0.2087 | 0.038 | 4.12E-08 | 737164 | 1.90E-04 | 140 |
| rs604723 | 11 | 100610546 | t | c | 0.2756 | -0.655 | 0.0339 | 2.55E-83 | 737165 | 7.20E-04 | 530 |
| rs7926110 | 11 | 107086143 | t | g | 0.6733 | 0.2603 | 0.0321 | 5.71E-16 | 738168 | 3.10E-04 | 232 |
| rs641620 | 11 | 117074229 | t | c | 0.8547 | -0.3193 | 0.044 | 3.74E-13 | 729449 | 2.20E-04 | 159 |
| rs573455 | 11 | 117267884 | a | g | 0.461 | 0.1994 | 0.0303 | 4.77E-11 | 737056 | 2.70E-04 | 201 |
| rs11222084 | 11 | 130273230 | a | t | 0.6379 | -0.3363 | 0.0316 | 1.80E-26 | 737164 | 4.30E-04 | 315 |
| rs7944927 | 11 | 130490917 | t | c | 0.7819 | 0.2235 | 0.0392 | 1.23E-08 | 737163 | 2.10E-04 | 154 |
| rs78998485 | 12 | 434755 | c | g | 0.7443 | -0.2449 | 0.0346 | 1.48E-12 | 744814 | 2.60E-04 | 191 |
| rs3819532 | 12 | 2436837 | t | c | 0.3913 | -0.1875 | 0.0306 | 9.44E-10 | 744814 | 2.50E-04 | 183 |
| rs113695818 | 12 | 8837407 | t | c | 0.3032 | -0.1835 | 0.033 | 2.62E-08 | 745820 | 2.10E-04 | 159 |
| rs2024385 | 12 | 12888438 | a | t | 0.424 | -0.2642 | 0.0306 | 5.88E-18 | 745819 | 3.50E-04 | 265 |
| rs1010064 | 12 | 20000315 | a | c | 0.8163 | 0.3571 | 0.0387 | 3.02E-20 | 745818 | 2.90E-04 | 220 |
| rs73075659 | 12 | 20373541 | a | g | 0.6654 | 0.3962 | 0.0321 | 5.52E-35 | 744703 | 4.80E-04 | 361 |
| rs2129869 | 12 | 26457650 | a | t | 0.7778 | -0.2643 | 0.0361 | 2.44E-13 | 743761 | 2.50E-04 | 187 |
| rs61917655 | 12 | 48210787 | t | c | 0.1014 | 0.3427 | 0.0514 | 2.68E-11 | 745817 | 1.70E-04 | 128 |
| rs57342147 | 12 | 50129422 | a | g | 0.9026 | 0.2787 | 0.0508 | 4.17E-08 | 745816 | 1.30E-04 | 100 |
| rs12426261 | 12 | 50573037 | a | g | 0.3792 | 0.3775 | 0.0309 | 2.31E-34 | 745819 | 4.90E-04 | 364 |
| rs7134440 | 12 | 53450097 | t | c | 0.0822 | 0.4788 | 0.0562 | 1.58E-17 | 745819 | 2.00E-04 | 148 |
| rs7134677 | 12 | 54441498 | t | c | 0.2978 | -0.3851 | 0.0332 | 4.46E-31 | 744813 | 4.40E-04 | 330 |
| rs7306710 | 12 | 66376091 | t | c | 0.481 | -0.2429 | 0.0303 | 1.03E-15 | 745819 | 3.30E-04 | 249 |
| rs4143175 | 12 | 67782397 | t | c | 0.2409 | 0.2187 | 0.0352 | 5.10E-10 | 744706 | 2.20E-04 | 164 |
| rs7963801 | 12 | 79685226 | t | c | 0.4221 | -0.2362 | 0.0311 | 2.87E-14 | 744815 | 3.20E-04 | 236 |

| | | | | | | | | | | | |
|---|---|---|---|---|---|---|---|---|---|---|---|
| rs6539467 | 12 | 79955306 | a | g | 0.1661 | 0.265 | 0.0404 | 5.57E-11 | 745818 | 2.00E-04 | 151 |
| rs17249754 | 12 | 90060586 | a | g | 0.1683 | -0.8446 | 0.0403 | 1.25E-97 | 743707 | 6.50E-04 | 484 |
| rs10777213 | 12 | 90349999 | a | g | 0.5244 | -0.1786 | 0.0299 | 2.45E-09 | 745820 | 2.40E-04 | 183 |
| rs5742643 | 12 | 102837863 | t | c | 0.2487 | -0.2233 | 0.0349 | 1.53E-10 | 740938 | 2.30E-04 | 170 |
| rs7310615 | 12 | 111865049 | c | g | 0.4816 | 0.585 | 0.0306 | 1.32E-81 | 737101 | 8.00E-04 | 592 |
| rs1896326 | 12 | 115342956 | a | g | 0.2291 | -0.2797 | 0.0371 | 4.41E-14 | 743700 | 2.70E-04 | 202 |
| rs35444 | 12 | 115552437 | a | g | 0.6138 | 0.4368 | 0.031 | 3.47E-45 | 737556 | 5.70E-04 | 420 |
| rs6490019 | 12 | 115920472 | a | g | 0.3796 | -0.2897 | 0.0309 | 6.61E-21 | 745818 | 3.70E-04 | 280 |
| rs1169078 | 12 | 122416254 | c | g | 0.6879 | -0.1971 | 0.0327 | 1.68E-09 | 744811 | 2.30E-04 | 173 |
| rs117206641 | 12 | 133086888 | t | c | 0.1108 | 0.3154 | 0.0499 | 2.66E-10 | 725612 | 1.70E-04 | 124 |
| rs483071 | 13 | 22294117 | t | c | 0.6248 | 0.2709 | 0.0313 | 5.09E-18 | 744705 | 3.50E-04 | 260 |
| rs9507885 | 13 | 27951090 | t | c | 0.0953 | -0.3208 | 0.0542 | 3.23E-09 | 740743 | 1.50E-04 | 113 |
| rs7338758 | 13 | 30137828 | t | c | 0.2448 | 0.3552 | 0.0352 | 7.02E-24 | 737099 | 3.60E-04 | 266 |
| rs4274337 | 13 | 41967193 | a | g | 0.1697 | -0.2968 | 0.0406 | 2.48E-13 | 745818 | 2.30E-04 | 172 |
| rs7491248 | 13 | 47180671 | a | g | 0.2239 | 0.2163 | 0.0362 | 2.38E-09 | 737558 | 2.10E-04 | 152 |
| rs9526707 | 13 | 51489186 | a | g | 0.3216 | -0.2039 | 0.0323 | 2.77E-10 | 744706 | 2.40E-04 | 182 |
| rs75961402 | 13 | 56398286 | a | g | 0.1534 | 0.2659 | 0.0418 | 1.95E-10 | 745819 | 1.90E-04 | 142 |
| rs17245822 | 13 | 73131694 | a | c | 0.6267 | -0.1899 | 0.0312 | 1.15E-09 | 745819 | 2.40E-04 | 182 |
| rs78474310 | 13 | 73826901 | a | g | 0.9552 | -0.4699 | 0.0734 | 1.51E-10 | 745820 | 1.10E-04 | 82 |
| rs6562778 | 13 | 74223828 | a | g | 0.4589 | 0.178 | 0.0304 | 4.96E-09 | 744815 | 2.40E-04 | 181 |
| rs9549627 | 13 | 113652369 | a | g | 0.1175 | 0.2846 | 0.05 | 1.25E-08 | 729202 | 1.60E-04 | 118 |
| rs7331680 | 13 | 115000650 | t | g | 0.1491 | 0.4101 | 0.0423 | 3.35E-22 | 742899 | 2.90E-04 | 213 |
| rs365990 | 14 | 23861811 | a | g | 0.6342 | 0.225 | 0.0312 | 5.95E-13 | 745820 | 2.90E-04 | 214 |
| rs8904 | 14 | 35871217 | a | g | 0.3678 | 0.3061 | 0.0314 | 1.71E-22 | 739799 | 3.90E-04 | 290 |
| rs7493678 | 14 | 39400917 | a | t | 0.6514 | -0.189 | 0.0316 | 2.31E-09 | 745819 | 2.40E-04 | 176 |
| rs72683923 | 14 | 50735947 | t | c | 0.9788 | 0.9587 | 0.1101 | 3.08E-18 | 743244 | 1.10E-04 | 81 |
| rs35413927 | 14 | 53420358 | a | g | 0.6946 | -0.3002 | 0.0328 | 5.25E-20 | 745820 | 3.50E-04 | 261 |
| rs12883810 | 14 | 68032235 | t | c | 0.1462 | -0.2382 | 0.0428 | 2.70E-08 | 736552 | 1.60E-04 | 120 |
| rs57786342 | 14 | 69260028 | a | g | 0.2059 | 0.2317 | 0.0374 | 5.63E-10 | 745818 | 2.10E-04 | 155 |
| rs8003103 | 14 | 71451265 | a | g | 0.3447 | -0.1755 | 0.0319 | 3.60E-08 | 745819 | 2.20E-04 | 163 |
| rs3815460 | 14 | 73422259 | c | g | 0.8976 | -0.285 | 0.05 | 1.21E-08 | 740939 | 1.40E-04 | 107 |
| rs11159091 | 14 | 75074316 | a | g | 0.4615 | 0.1978 | 0.0303 | 6.79E-11 | 735987 | 2.70E-04 | 199 |
| rs7154723 | 14 | 98590629 | a | g | 0.385 | 0.253 | 0.0309 | 2.72E-16 | 744815 | 3.30E-04 | 245 |
| rs17562391 | 14 | 100133250 | t | c | 0.4186 | 0.1967 | 0.0306 | 1.35E-10 | 745818 | 2.60E-04 | 196 |
| rs75016974 | 14 | 100197940 | t | c | 0.1423 | -0.2513 | 0.0439 | 1.05E-08 | 744815 | 1.70E-04 | 126 |
| rs12885878 | 14 | 104007555 | a | g | 0.2337 | -0.2291 | 0.0367 | 4.32E-10 | 744814 | 2.30E-04 | 168 |
| rs8030856 | 15 | 40314967 | c | g | 0.6047 | -0.1764 | 0.031 | 1.21E-08 | 744815 | 2.30E-04 | 173 |
| rs28866311 | 15 | 41442195 | t | g | 0.5263 | -0.2762 | 0.0302 | 5.45E-20 | 745820 | 3.80E-04 | 282 |
| rs4775769 | 15 | 48939888 | t | g | 0.0945 | -0.4162 | 0.0517 | 7.76E-16 | 745818 | 2.00E-04 | 146 |
| rs3098186 | 15 | 50810621 | t | c | 0.5156 | -0.2422 | 0.0303 | 1.41E-15 | 745820 | 3.30E-04 | 248 |
| rs2652812 | 15 | 63406170 | t | c | 0.7544 | -0.2516 | 0.0353 | 1.03E-12 | 744814 | 2.60E-04 | 191 |
| rs28429256 | 15 | 66931617 | a | g | 0.3342 | 0.215 | 0.0325 | 3.89E-11 | 743700 | 2.60E-04 | 196 |
| rs11636952 | 15 | 75114322 | t | c | 0.3141 | 0.5313 | 0.0328 | 4.22E-59 | 727894 | 6.30E-04 | 458 |
| rs2627313 | 15 | 81006712 | t | c | 0.4454 | 0.3208 | 0.0303 | 3.55E-26 | 737101 | 4.40E-04 | 321 |
| rs1994158 | 15 | 86064327 | a | g | 0.8193 | 0.2513 | 0.0391 | 1.23E-10 | 743705 | 2.00E-04 | 152 |
| rs17807723 | 15 | 90023558 | a | g | 0.138 | -0.2721 | 0.0443 | 8.43E-10 | 737795 | 1.80E-04 | 131 |

| | | | | | | | | | | | |
|---|---|---|---|---|---|---|---|---|---|---|---|
| rs4932373 | 15 | 91429287 | a | c | 0.6742 | -0.635 | 0.0328 | 2.49E-83 | 724766 | 7.70E-04 | 556 |
| rs12906962 | 15 | 95312071 | t | c | 0.676 | -0.2653 | 0.0325 | 3.28E-16 | 742702 | 3.20E-04 | 237 |
| rs2589218 | 15 | 96785017 | t | c | 0.7297 | -0.2258 | 0.0339 | 2.54E-11 | 743708 | 2.40E-04 | 182 |
| rs4606697 | 15 | 100087596 | a | g | 0.1041 | -0.3196 | 0.0523 | 9.71E-10 | 740084 | 1.60E-04 | 121 |
| rs11641374 | 16 | 1347717 | a | c | 0.5995 | -0.1943 | 0.0309 | 3.26E-10 | 741588 | 2.60E-04 | 190 |
| rs12596630 | 16 | 2065666 | t | c | 0.0903 | 0.4278 | 0.0547 | 5.01E-15 | 723276 | 1.90E-04 | 140 |
| rs7189884 | 16 | 4145164 | a | g | 0.1146 | -0.3138 | 0.0476 | 4.20E-11 | 745818 | 1.70E-04 | 131 |
| rs12446456 | 16 | 4922201 | t | c | 0.4274 | -0.3003 | 0.0302 | 2.97E-23 | 745820 | 4.00E-04 | 301 |
| rs11075030 | 16 | 11976414 | a | c | 0.5937 | -0.1745 | 0.0309 | 1.72E-08 | 728838 | 2.30E-04 | 169 |
| rs77924615 | 16 | 20392332 | a | g | 0.1986 | -0.4081 | 0.039 | 1.12E-25 | 743700 | 3.60E-04 | 266 |
| rs7186298 | 16 | 21088031 | t | c | 0.4295 | -0.2315 | 0.0302 | 1.88E-14 | 745820 | 3.10E-04 | 233 |
| rs8044992 | 16 | 24811207 | t | c | 0.7123 | 0.2138 | 0.0331 | 1.07E-10 | 745819 | 2.40E-04 | 180 |
| rs34941092 | 16 | 50550137 | a | g | 0.1498 | -0.3225 | 0.0425 | 3.23E-14 | 745818 | 2.30E-04 | 168 |
| rs4784541 | 16 | 51704452 | t | c | 0.4748 | -0.2015 | 0.0307 | 4.93E-11 | 744815 | 2.80E-04 | 206 |
| rs35098810 | 16 | 60635748 | a | c | 0.7683 | 0.1967 | 0.0356 | 3.20E-08 | 745817 | 1.90E-04 | 144 |
| rs146550789 | 16 | 66781040 | t | c | 0.9583 | -0.4824 | 0.0778 | 5.64E-10 | 745818 | 1.10E-04 | 79 |
| rs62047964 | 16 | 70729954 | t | c | 0.0622 | 0.5115 | 0.0686 | 9.29E-14 | 736711 | 1.60E-04 | 121 |
| rs1012089 | 16 | 74171973 | c | g | 0.4752 | -0.192 | 0.0302 | 1.95E-10 | 745819 | 2.60E-04 | 196 |
| rs4888408 | 16 | 75432824 | a | g | 0.5855 | 0.3653 | 0.0307 | 1.42E-32 | 744815 | 4.90E-04 | 363 |
| rs12926550 | 16 | 81510155 | a | g | 0.3156 | -0.2548 | 0.0324 | 3.43E-15 | 745819 | 3.00E-04 | 226 |
| rs8054587 | 16 | 86170044 | t | c | 0.5272 | 0.1665 | 0.0302 | 3.41E-08 | 744813 | 2.30E-04 | 170 |
| rs3950627 | 16 | 86436343 | a | c | 0.531 | 0.1851 | 0.0308 | 1.82E-09 | 738794 | 2.50E-04 | 187 |
| rs6540119 | 16 | 87984477 | a | t | 0.334 | 0.2016 | 0.0322 | 3.93E-10 | 745819 | 2.50E-04 | 184 |
| rs908951 | 16 | 89697625 | t | c | 0.4378 | -0.2261 | 0.0315 | 7.14E-13 | 734063 | 3.10E-04 | 225 |
| rs8079811 | 17 | 1371473 | c | g | 0.3479 | -0.2101 | 0.0325 | 1.03E-10 | 739409 | 2.60E-04 | 194 |
| rs2760748 | 17 | 2001604 | a | t | 0.0981 | 0.3626 | 0.0509 | 1.05E-12 | 744704 | 1.80E-04 | 131 |
| rs113086489 | 17 | 7171356 | t | c | 0.5525 | 0.3249 | 0.0307 | 3.80E-26 | 744814 | 4.40E-04 | 329 |
| rs4511593 | 17 | 7455536 | t | c | 0.6528 | -0.2881 | 0.0318 | 1.28E-19 | 737557 | 3.60E-04 | 265 |
| rs79930761 | 17 | 7815712 | t | c | 0.0872 | -0.4688 | 0.0559 | 4.90E-17 | 745818 | 2.10E-04 | 153 |
| rs4925159 | 17 | 18185510 | a | g | 0.4246 | 0.2174 | 0.0305 | 9.66E-13 | 737558 | 2.90E-04 | 215 |
| rs7211535 | 17 | 19922364 | a | g | 0.4764 | -0.1779 | 0.0304 | 4.61E-09 | 737558 | 2.40E-04 | 180 |
| rs1551355 | 17 | 30032420 | t | c | 0.2334 | 0.2098 | 0.0356 | 3.89E-09 | 745818 | 2.10E-04 | 154 |
| rs9899540 | 17 | 30777924 | a | t | 0.3999 | 0.2011 | 0.0316 | 1.87E-10 | 744813 | 2.70E-04 | 198 |
| rs7213273 | 17 | 43155914 | a | g | 0.655 | -0.4 | 0.0315 | 6.24E-37 | 745819 | 5.00E-04 | 371 |
| rs17608766 | 17 | 45013271 | t | c | 0.8555 | -0.6903 | 0.0433 | 2.48E-57 | 737558 | 4.70E-04 | 346 |
| rs3764400 | 17 | 46123932 | t | c | 0.8635 | 0.3748 | 0.0445 | 3.69E-17 | 744815 | 2.40E-04 | 181 |
| rs9897429 | 17 | 47518378 | a | g | 0.52 | 0.2645 | 0.0319 | 1.19E-16 | 744815 | 3.60E-04 | 270 |
| rs1000423 | 17 | 59475642 | t | c | 0.7316 | 0.4138 | 0.0346 | 6.50E-33 | 737099 | 4.50E-04 | 329 |
| rs56288724 | 17 | 60767135 | a | g | 0.5831 | -0.2178 | 0.031 | 2.01E-12 | 744706 | 2.90E-04 | 217 |
| rs62076622 | 17 | 61090958 | a | g | 0.8013 | 0.2363 | 0.0377 | 3.79E-10 | 745819 | 2.10E-04 | 154 |
| rs6504213 | 17 | 62381714 | t | c | 0.4182 | -0.2982 | 0.0312 | 1.25E-21 | 744814 | 4.00E-04 | 297 |
| rs1436138 | 17 | 75316880 | a | g | 0.6367 | 0.3119 | 0.0315 | 4.73E-23 | 744705 | 4.00E-04 | 295 |
| rs9302885 | 17 | 76799898 | a | g | 0.4452 | 0.2242 | 0.0302 | 1.03E-13 | 744706 | 3.00E-04 | 227 |
| rs11655604 | 17 | 79365861 | t | c | 0.3579 | -0.2033 | 0.0333 | 1.09E-09 | 699816 | 2.60E-04 | 180 |
| rs34413141 | 18 | 777282 | a | t | 0.1822 | -0.3531 | 0.0393 | 2.47E-19 | 745818 | 2.90E-04 | 216 |
| rs62082230 | 18 | 22676071 | a | t | 0.2773 | -0.1884 | 0.0345 | 4.69E-08 | 744814 | 2.10E-04 | 155 |

| | | | | | | | | | | | | |
|---|---|---|---|---|---|---|---|---|---|---|---|---|
| rs1154214 | 18 | 24546824 | t | g | 0.3963 | -0.2031 | 0.0306 | 3.27E-11 | 744706 | 2.70E-04 | 199 |
| rs56407827 | 18 | 42179819 | t | c | 0.2687 | 0.3603 | 0.034 | 2.78E-26 | 744704 | 3.90E-04 | 290 |
| rs11874246 | 18 | 42596789 | t | c | 0.2963 | 0.2856 | 0.0328 | 3.23E-18 | 745818 | 3.30E-04 | 244 |
| rs7236548 | 18 | 43097750 | a | c | 0.1848 | 0.3431 | 0.0388 | 8.51E-19 | 745818 | 2.80E-04 | 212 |
| rs1437649 | 18 | 48132646 | a | g | 0.2345 | -0.2189 | 0.0357 | 8.57E-10 | 745819 | 2.20E-04 | 161 |
| rs665445 | 18 | 51842682 | a | c | 0.2794 | -0.1909 | 0.0334 | 1.15E-08 | 745819 | 2.10E-04 | 158 |
| rs10048404 | 18 | 54578482 | t | c | 0.3701 | -0.2607 | 0.0317 | 1.91E-16 | 744815 | 3.30E-04 | 249 |
| rs10460108 | 18 | 73034151 | a | g | 0.4801 | 0.2141 | 0.0301 | 1.12E-12 | 745819 | 2.90E-04 | 219 |
| rs698748 | 19 | 1424888 | a | g | 0.421 | 0.1871 | 0.0325 | 8.90E-09 | 722073 | 2.50E-04 | 181 |
| rs149339216 | 19 | 2144046 | t | c | 0.9566 | -0.6912 | 0.0779 | 6.93E-19 | 724208 | 1.60E-04 | 114 |
| rs68096471 | 19 | 5175709 | a | g | 0.2659 | -0.2098 | 0.0343 | 9.26E-10 | 745819 | 2.30E-04 | 168 |
| rs12985940 | 19 | 7262734 | t | c | 0.8408 | 0.4642 | 0.0434 | 1.08E-26 | 721439 | 3.40E-04 | 247 |
| rs3816865 | 19 | 11507855 | a | g | 0.0801 | 0.3092 | 0.0565 | 4.39E-08 | 737556 | 1.30E-04 | 92 |
| rs167479 | 19 | 11526765 | t | g | 0.4726 | -0.5642 | 0.0327 | 7.21E-67 | 675533 | 7.70E-04 | 523 |
| rs8106184 | 19 | 17159779 | a | c | 0.7449 | -0.2371 | 0.0347 | 8.34E-12 | 745819 | 2.50E-04 | 185 |
| rs4319878 | 19 | 21924452 | t | c | 0.5606 | 0.1694 | 0.0308 | 3.77E-08 | 744815 | 2.30E-04 | 171 |
| rs8108027 | 19 | 22115901 | c | g | 0.2947 | 0.1948 | 0.033 | 3.67E-09 | 745819 | 2.20E-04 | 166 |
| rs28572357 | 19 | 31867447 | a | c | 0.6023 | -0.2733 | 0.0308 | 6.34E-19 | 741283 | 3.60E-04 | 267 |
| rs1433121 | 19 | 32591878 | t | c | 0.6906 | -0.228 | 0.0326 | 2.66E-12 | 745817 | 2.70E-04 | 200 |
| rs33836 | 19 | 34008600 | t | c | 0.4622 | 0.1766 | 0.0304 | 6.56E-09 | 745820 | 2.40E-04 | 180 |
| rs10420519 | 19 | 45298461 | t | g | 0.0347 | -0.4921 | 0.0887 | 2.86E-08 | 738581 | 9.10E-05 | 67 |
| rs7255933 | 19 | 45766729 | a | g | 0.2574 | 0.2306 | 0.0345 | 2.44E-11 | 745819 | 2.40E-04 | 181 |
| rs11672660 | 19 | 46180184 | t | c | 0.1996 | 0.2212 | 0.0381 | 6.32E-09 | 737033 | 1.90E-04 | 143 |
| rs571689 | 19 | 49207554 | t | c | 0.5196 | 0.228 | 0.0304 | 6.77E-14 | 737035 | 3.10E-04 | 231 |
| rs73046792 | 19 | 49605705 | a | g | 0.1588 | -0.3554 | 0.0426 | 7.23E-17 | 737035 | 2.60E-04 | 192 |
| rs6054139 | 20 | 6327810 | a | g | 0.606 | 0.2094 | 0.0306 | 8.23E-12 | 745818 | 2.70E-04 | 205 |
| rs2423514 | 20 | 10693337 | a | g | 0.5411 | 0.3011 | 0.0302 | 1.77E-23 | 745820 | 4.10E-04 | 307 |
| rs6108787 | 20 | 10967214 | t | g | 0.5296 | -0.4274 | 0.03 | 5.38E-46 | 743761 | 5.90E-04 | 436 |
| rs6078093 | 20 | 11168669 | a | g | 0.428 | -0.1849 | 0.0304 | 1.20E-09 | 744815 | 2.50E-04 | 185 |
| rs8125763 | 20 | 17883531 | a | c | 0.4717 | 0.1761 | 0.0301 | 4.84E-09 | 745819 | 2.40E-04 | 180 |
| rs17812022 | 20 | 19007099 | t | c | 0.0958 | -0.3613 | 0.0525 | 5.65E-12 | 744814 | 1.70E-04 | 128 |
| rs6058088 | 20 | 30139886 | t | g | 0.8439 | 0.2832 | 0.0417 | 1.14E-11 | 745819 | 2.10E-04 | 153 |
| rs79384779 | 20 | 31214944 | t | c | 0.1512 | 0.3179 | 0.0428 | 1.08E-13 | 745820 | 2.20E-04 | 167 |
| rs6029756 | 20 | 40266681 | a | g | 0.3225 | -0.2712 | 0.033 | 1.88E-16 | 744814 | 3.30E-04 | 243 |
| rs6031431 | 20 | 42795152 | a | g | 0.5376 | -0.2617 | 0.0304 | 7.05E-18 | 743700 | 3.60E-04 | 266 |
| rs2598 | 20 | 47241618 | a | g | 0.533 | 0.168 | 0.0303 | 2.87E-08 | 745818 | 2.30E-04 | 171 |
| rs6090907 | 20 | 47410231 | a | g | 0.147 | -0.3854 | 0.0425 | 1.29E-19 | 745820 | 2.70E-04 | 198 |
| rs234623 | 20 | 57488964 | a | g | 0.5041 | -0.1804 | 0.0302 | 2.43E-09 | 741285 | 2.50E-04 | 184 |
| rs6026744 | 20 | 57742388 | a | t | 0.8771 | -0.7131 | 0.0461 | 7.00E-54 | 741284 | 4.20E-04 | 313 |
| rs28374392 | 20 | 61189717 | t | c | 0.6231 | 0.1924 | 0.0338 | 1.21E-08 | 678027 | 2.50E-04 | 168 |
| rs6062324 | 20 | 62446351 | a | g | 0.2364 | -0.3294 | 0.0363 | 1.18E-19 | 738823 | 3.30E-04 | 242 |
| rs2776037 | 21 | 16317933 | t | c | 0.4151 | -0.1851 | 0.0309 | 2.15E-09 | 743701 | 2.50E-04 | 184 |
| rs1882961 | 21 | 16556367 | t | c | 0.3087 | 0.2443 | 0.0326 | 6.69E-14 | 745820 | 2.90E-04 | 214 |
| rs2833834 | 21 | 33814378 | a | c | 0.2765 | 0.2177 | 0.0338 | 1.22E-10 | 737558 | 2.40E-04 | 177 |
| rs12627651 | 21 | 44760603 | a | g | 0.2872 | 0.3498 | 0.0341 | 1.02E-24 | 741589 | 3.90E-04 | 292 |
| rs34487963 | 21 | 44838330 | a | c | 0.0185 | -0.8819 | 0.1244 | 1.35E-12 | 716018 | 8.80E-05 | 63 |

| | | | | | | | | | | | |
|---|---|---|---|---|---|---|---|---|---|---|---|
| rs7278003 | 21 | 44966069 | t | c | 0.4378 | -0.1876 | 0.0304 | 6.63E-10 | 744814 | 2.50E-04 | 189 |
| rs2238787 | 22 | 19976406 | a | g | 0.292 | 0.2552 | 0.0332 | 1.45E-14 | 743706 | 2.90E-04 | 216 |
| rs12321 | 22 | 29453193 | c | g | 0.4328 | -0.2292 | 0.0303 | 3.81E-14 | 745820 | 3.10E-04 | 231 |
| rs112854918 | 22 | 30588910 | c | g | 0.9745 | -0.5577 | 0.1004 | 2.77E-08 | 744068 | 7.60E-05 | 57 |
| rs8142376 | 22 | 32001037 | t | c | 0.491 | 0.1676 | 0.03 | 2.20E-08 | 745819 | 2.30E-04 | 172 |
| rs148140538 | 22 | 50228044 | t | c | 0.0808 | -0.3252 | 0.0562 | 7.39E-09 | 742190 | 1.30E-04 | 99 |
| rs28578714 | 22 | 50727921 | t | c | 0.6062 | 0.2066 | 0.0327 | 2.53E-10 | 713093 | 2.70E-04 | 193 |

Chr: chromosome, Pos: position (hg19), EA: effect allele, OA: other allele, EAF: effect allele frequency, SE: standard error, $R^2$: proportion of variance explained.

| Supplementary Table 2. Regions used to identify instruments for each drug. | | | | |
|---|---|---|---|---|
| Drug | Target gene | Chromosome | Position (hg19) | Function |
| **ACEI** | ***ACE*** | **17** | **61554422-61599205** | **Gene** |
| ACEI | *ACE* | 17 | 61551058-61556950 | Promoter/Enhancer |
| ACEI | *ACE* | 17 | 61562201-61562303 | Promoter/Enhancer |
| ACEI | *ACE* | 17 | 61508611-61515166 | Promoter/Enhancer |
| ACEI | *ACE* | 17 | 61626418-61630304 | Promoter/Enhancer |
| ACEI | *ACE* | 17 | 61431510-61431613 | Enhancer |
| ACEI | *ACE* | 17 | 62090924-62103850 | Promoter/Enhancer |
| ACEI | *ACE* | 17 | 61497048-61498662 | Enhancer |
| ACEI | *ACE* | 17 | 61505277-61506104 | Enhancer |
| ACEI | *ACE* | 17 | 61689560-61689960 | Enhancer |
| ACEI | *ACE* | 17 | 61594421-61594870 | Enhancer |
| ACEI | *ACE* | 17 | 61502881-61503030 | Enhancer |
| ACEI | *ACE* | 17 | 61656647-61657871 | Enhancer |
| ACEI | *ACE* | 17 | 61500762-61501161 | Enhancer |
| ACEI | *ACE* | 17 | 60855121-60860435 | Enhancer |
| ACEI | *ACE* | 17 | 61574731-61577281 | Enhancer |
| ACEI | *ACE* | 17 | 60972606-60973907 | Enhancer |
| **ARB** | ***AGTR1*** | **3** | **148415571-148460795** | **Gene** |
| ARB | *AGTR1* | 3 | 148415061-148416388 | Promoter/Enhancer |
| ARB | *AGTR1* | 3 | 148366071-148367473 | Enhancer |
| ARB | *AGTR1* | 3 | 148441102-148442130 | Enhancer |
| ARB | *AGTR1* | 3 | 148360847-148362186 | Enhancer |
| ARB | *AGTR1* | 3 | 148360520-148360788 | Enhancer |
| ARB | *AGTR1* | 3 | 148899476-148899525 | Enhancer |
| **BB** | ***ADRB1*** | **10** | **115803806-115806667** | **Gene** |
| BB | *ADRB1* | 10 | 115802241-115807338 | Promoter/Enhancer |
| BB | *ADRB1* | 10 | 115716558-115722360 | Enhancer |
| BB | *ADRB1* | 10 | 115706609-115708137 | Enhancer |
| BB | *ADRB1* | 10 | 115824009-115824850 | Enhancer |
| BB | *ADRB1* | 10 | 115548188-115549279 | Enhancer |
| BB | *ADRB1* | 10 | 115833610-115834154 | Enhancer |
| BB | *ADRB1* | 10 | 115704333-115705870 | Enhancer |
| BB | *ADRB1* | 10 | 116441242-116446390 | Promoter/Enhancer |
| BB | *ADRB1* | 10 | 115842035-115843254 | Enhancer |
| BB | *ADRB1* | 10 | 115784258-115788102 | Enhancer |
| BB | *ADRB1* | 10 | 115725160-115725959 | Enhancer |
| BB | *ADRB1* | 10 | 115697701-115697810 | Enhancer |
| BB | *ADRB1* | 10 | 115741347-115744110 | Enhancer |
| BB | *ADRB1* | 10 | 115782141-115782270 | Enhancer |
| BB | *ADRB1* | 10 | 115559441-115559610 | Enhancer |
| BB | *ADRB1* | 10 | 115841821-115841970 | Enhancer |
| BB | *ADRB1* | 10 | 115826224-115827332 | Enhancer |
| BB | *ADRB1* | 10 | 115827575-115828874 | Enhancer |
| BB | *ADRB1* | 10 | 115910708-115912162 | Enhancer |
| BB | *ADRB1* | 10 | 115752960-115753842 | Enhancer |
| BB | *ADRB1* | 10 | 115651401-115651550 | Enhancer |
| BB | *ADRB1* | 10 | 115683621-115684573 | Enhancer |
| BB | *ADRB1* | 10 | 115758960-115759559 | Enhancer |
| BB | *ADRB1* | 10 | 116457630-116458961 | Enhancer |
| BB | *ADRB1* | 10 | 115561321-115561530 | Enhancer |
| BB | *ADRB1* | 10 | 115625638-115626159 | Enhancer |
| BB | *ADRB1* | 10 | 116437959-116439282 | Enhancer |
| BB | *ADRB1* | 10 | 115789385-115790216 | Enhancer |
| BB | *ADRB1* | 10 | 115800822-115802086 | Enhancer |
| BB | *ADRB1* | 10 | 115799814-115800507 | Enhancer |
| **CCB** | ***CACNA1S*** | **1** | **201008640-201081694** | **Gene** |
| CCB | *CACNA1S* | 1 | 201082861-201084129 | Promoter/Enhancer |
| CCB | *CACNA1S* | 1 | 201079942-201082115 | Enhancer |
| CCB | *CACNA1S* | 1 | 201122647-201124394 | Promoter/Enhancer |
| CCB | *CACNA1S* | 1 | 201274996-201282487 | Enhancer |
| CCB | *CACNA1S* | 1 | 201012141-201012270 | Enhancer |
| CCB | *CACNA1S* | 1 | 201263489-201273426 | Enhancer |
| CCB | *CACNA1S* | 1 | 200941201-200941350 | Enhancer |

| | | | | |
|---|---|---|---|---|
| CCB | *CACNA1S* | 1 | 201106054-201107629 | Enhancer |
| CCB | *CACNA1S* | 1 | 201056384-201057751 | Enhancer |
| CCB | *CACNA1S* | 1 | 201057880-201061411 | Enhancer |
| CCB | *CACNA1S* | 1 | 201111781-201111890 | Enhancer |
| CCB | *CACNA1S* | 1 | 201063580-201070116 | Enhancer |
| CCB | *CACNA1S* | 1 | 201071203-201078371 | Enhancer |
| CCB | *CACNA1S* | 1 | 201032066-201032442 | Enhancer |
| **CCB** | ***CACNA1F*** | **X** | **49061523-49089833** | **Gene** |
| CCB | *CACNA1F* | X | 49091301-49092868 | Promoter/Enhancer |
| CCB | *CACNA1F* | X | 49089792-49089851 | Promoter |
| CCB | *CACNA1F* | X | 49086401-49088145 | Enhancer |
| CCB | *CACNA1F* | X | 49044793-49049057 | Enhancer |
| CCB | *CACNA1F* | X | 49122932-49127611 | Promoter/Enhancer |
| CCB | *CACNA1F* | X | 49056201-49057353 | Promoter/Enhancer |
| CCB | *CACNA1F* | X | 49019538-49025109 | Promoter/Enhancer |
| CCB | *CACNA1F* | X | 49040381-49044351 | Promoter/Enhancer |
| CCB | *CACNA1F* | X | 48915921-48920218 | Promoter/Enhancer |
| CCB | *CACNA1F* | X | 49093233-49093850 | Enhancer |
| CCB | *CACNA1F* | X | 49085421-49085530 | Enhancer |
| CCB | *CACNA1F* | X | 49066401-49066570 | Enhancer |
| CCB | *CACNA1F* | X | 49065861-49066060 | Enhancer |
| **CCB** | ***CACNA1D*** | **3** | **53528683-53847760** | **Gene** |
| CCB | *CACNA1D* | 3 | 53526751-53529027 | Promoter/Enhancer |
| CCB | *CACNA1D* | 3 | 53529053-53530500 | Promoter/Enhancer |
| CCB | *CACNA1D* | 3 | 53361979-53363570 | Enhancer |
| CCB | *CACNA1D* | 3 | 53551784-53554581 | Enhancer |
| CCB | *CACNA1D* | 3 | 53558098-53559174 | Enhancer |
| CCB | *CACNA1D* | 3 | 53379987-53382717 | Promoter/Enhancer |
| CCB | *CACNA1D* | 3 | 53859957-53860202 | Enhancer |
| CCB | *CACNA1D* | 3 | 53384627-53385227 | Enhancer |
| CCB | *CACNA1D* | 3 | 53388806-53389878 | Enhancer |
| CCB | *CACNA1D* | 3 | 53354541-53355561 | Enhancer |
| CCB | *CACNA1D* | 3 | 53511553-53514862 | Enhancer |
| CCB | *CACNA1D* | 3 | 53457125-53457810 | Enhancer |
| CCB | *CACNA1D* | 3 | 53539797-53541150 | Enhancer |
| CCB | *CACNA1D* | 3 | 53531730-53534232 | Enhancer |
| CCB | *CACNA1D* | 3 | 53405028-53405227 | Enhancer |
| CCB | *CACNA1D* | 3 | 53559666-53560346 | Enhancer |
| CCB | *CACNA1D* | 3 | 53698941-53699090 | Enhancer |
| CCB | *CACNA1D* | 3 | 53604273-53605793 | Enhancer |
| CCB | *CACNA1D* | 3 | 53742028-53742227 | Enhancer |
| CCB | *CACNA1D* | 3 | 53664741-53664930 | Enhancer |
| CCB | *CACNA1D* | 3 | 53714181-53714330 | Enhancer |
| CCB | *CACNA1D* | 3 | 53664161-53664290 | Enhancer |
| CCB | *CACNA1D* | 3 | 53647109-53647870 | Enhancer |
| CCB | *CACNA1D* | 3 | 53568043-53568300 | Enhancer |
| CCB | *CACNA1D* | 3 | 53707221-53707290 | Enhancer |
| CCB | *CACNA1D* | 3 | 53723669-53724036 | Enhancer |
| CCB | *CACNA1D* | 3 | 53741428-53741627 | Enhancer |
| CCB | *CACNA1D* | 3 | 53787436-53788581 | Enhancer |
| CCB | *CACNA1D* | 3 | 53793207-53793702 | Enhancer |
| CCB | *CACNA1D* | 3 | 53796777-53798080 | Enhancer |
| CCB | *CACNA1D* | 3 | 53798222-53799233 | Enhancer |
| CCB | *CACNA1D* | 3 | 53810959-53811945 | Enhancer |
| CCB | *CACNA1D* | 3 | 53809709-53810892 | Enhancer |
| CCB | *CACNA1D* | 3 | 53817819-53818839 | Enhancer |
| CCB | *CACNA1D* | 3 | 53828693-53829307 | Enhancer |
| CCB | *CACNA1D* | 3 | 53829341-53829430 | Enhancer |
| CCB | *CACNA1D* | 3 | 53838172-53842050 | Enhancer |
| CCB | *CACNA1D* | 3 | 53744028-53744627 | Enhancer |
| CCB | *CACNA1D* | 3 | 53759658-53759955 | Enhancer |
| CCB | *CACNA1D* | 3 | 53843873-53844716 | Enhancer |
| CCB | *CACNA1D* | 3 | 53802862-53803908 | Enhancer |
| CCB | *CACNA1D* | 3 | 53806640-53808437 | Enhancer |
| CCB | *CACNA1D* | 3 | 53799850-53800909 | Enhancer |

| | | | | |
|---|---|---|---|---|
| CCB | *CACNA1D* | 3 | 53801465-53802329 | Enhancer |
| CCB | *CACNA1D* | 3 | 53782002-53784852 | Enhancer |
| CCB | *CACNA1D* | 3 | 53777569-53779058 | Enhancer |
| CCB | *CACNA1D* | 3 | 53764428-53765080 | Enhancer |
| CCB | *CACNA1D* | 3 | 53766775-53767435 | Enhancer |
| **CCB** | ***CACNA2D1*** | **7** | **81575760-82073114** | **Gene** |
| CCB | *CACNA2D1* | 7 | 82071056-82074330 | Promoter/Enhancer |
| CCB | *CACNA2D1* | 7 | 82039597-82041108 | Enhancer |
| CCB | *CACNA2D1* | 7 | 82223184-82225724 | Enhancer |
| CCB | *CACNA2D1* | 7 | 82171489-82172576 | Enhancer |
| CCB | *CACNA2D1* | 7 | 82164161-82164310 | Enhancer |
| CCB | *CACNA2D1* | 7 | 82063702-82063952 | Enhancer |
| CCB | *CACNA2D1* | 7 | 81584968-81585322 | Enhancer |
| CCB | *CACNA2D1* | 7 | 81858656-81858971 | Enhancer |
| CCB | *CACNA2D1* | 7 | 82057141-82058356 | Enhancer |
| CCB | *CACNA2D1* | 7 | 82059961-82060110 | Enhancer |
| CCB | *CACNA2D1* | 7 | 82013345-82013689 | Enhancer |
| CCB | *CACNA2D1* | 7 | 81785601-81785750 | Enhancer |
| CCB | *CACNA2D1* | 7 | 81948701-81949880 | Enhancer |
| CCB | *CACNA2D1* | 7 | 81833679-81834445 | Enhancer |
| CCB | *CACNA2D1* | 7 | 81734523-81735564 | Enhancer |
| CCB | *CACNA2D1* | 7 | 82034101-82034250 | Enhancer |
| CCB | *CACNA2D1* | 7 | 81920961-81922045 | Enhancer |
| CCB | *CACNA2D1* | 7 | 81709702-81710455 | Enhancer |
| CCB | *CACNA2D1* | 7 | 81914377-81914723 | Enhancer |
| CCB | *CACNA2D1* | 7 | 81808741-81808930 | Enhancer |
| CCB | *CACNA2D1* | 7 | 81739157-81739440 | Enhancer |
| CCB | *CACNA2D1* | 7 | 81916621-81916770 | Enhancer |
| CCB | *CACNA2D1* | 7 | 81787741-81787827 | Enhancer |
| CCB | *CACNA2D1* | 7 | 81946361-81947530 | Enhancer |
| CCB | *CACNA2D1* | 7 | 81941894-81943091 | Enhancer |
| CCB | *CACNA2D1* | 7 | 81915266-81916265 | Enhancer |
| CCB | *CACNA2D1* | 7 | 81914861-81914999 | Enhancer |
| CCB | *CACNA2D1* | 7 | 81809481-81810272 | Enhancer |
| CCB | *CACNA2D1* | 7 | 81739641-81739790 | Enhancer |
| CCB | *CACNA2D1* | 7 | 81841421-81842618 | Enhancer |
| CCB | *CACNA2D1* | 7 | 81813081-81813230 | Enhancer |
| CCB | *CACNA2D1* | 7 | 81590210-81590492 | Enhancer |
| CCB | *CACNA2D1* | 7 | 81678694-81681663 | Enhancer |
| CCB | *CACNA2D1* | 7 | 81664197-81666962 | Enhancer |
| **CCB** | ***CACNA2D2*** | **3** | **50400230-50541675** | **Gene** |
| CCB | *CACNA2D2* | 3 | 50540431-50541530 | Promoter/Enhancer |
| CCB | *CACNA2D2* | 3 | 50535234-50535293 | Promoter |
| CCB | *CACNA2D2* | 3 | 51420877-51430387 | Promoter/Enhancer |
| CCB | *CACNA2D2* | 3 | 50510252-50511323 | Enhancer |
| CCB | *CACNA2D2* | 3 | 50624952-50631056 | Enhancer |
| CCB | *CACNA2D2* | 3 | 50486750-50489034 | Enhancer |
| CCB | *CACNA2D2* | 3 | 50552257-50555213 | Enhancer |
| CCB | *CACNA2D2* | 3 | 50557041-50557190 | Enhancer |
| CCB | *CACNA2D2* | 3 | 50560739-50564041 | Enhancer |
| CCB | *CACNA2D2* | 3 | 50483138-50484199 | Enhancer |
| CCB | *CACNA2D2* | 3 | 50484337-50485546 | Enhancer |
| CCB | *CACNA2D2* | 3 | 50473601-50473790 | Enhancer |
| CCB | *CACNA2D2* | 3 | 50464650-50466328 | Enhancer |
| CCB | *CACNA2D2* | 3 | 50401766-50403767 | Promoter/Enhancer |
| CCB | *CACNA2D2* | 3 | 50479739-50481683 | Enhancer |
| CCB | *CACNA2D2* | 3 | 50472754-50473413 | Enhancer |
| CCB | *CACNA2D2* | 3 | 50467315-50469064 | Enhancer |
| CCB | *CACNA2D2* | 3 | 50427643-50429192 | Enhancer |
| CCB | *CACNA2D2* | 3 | 50410601-50411305 | Enhancer |
| CCB | *CACNA2D2* | 3 | 50491422-50491591 | Enhancer |
| CCB | *CACNA2D2* | 3 | 50425346-50425879 | Enhancer |
| CCB | *CACNA2D2* | 3 | 50411624-50411893 | Enhancer |
| **CCB** | ***CACNB1*** | **17** | **37329709-37353956** | **Gene** |
| CCB | *CACNB1* | 17 | 37347421-37359080 | Promoter/Enhancer |

| | | | | |
|---|---|---|---|---|
| CCB | *CACNB1* | 17 | 37330363-37331930 | Enhancer |
| CCB | *CACNB1* | 17 | 37170207-37173852 | Enhancer |
| CCB | *CACNB1* | 17 | 37392822-37395535 | Enhancer |
| CCB | *CACNB1* | 17 | 37512839-37515343 | Enhancer |
| CCB | *CACNB1* | 17 | 37401081-37401270 | Enhancer |
| CCB | *CACNB1* | 17 | 37401361-37401510 | Enhancer |
| CCB | *CACNB1* | 17 | 37174255-37175468 | Enhancer |
| CCB | *CACNB1* | 17 | 37332121-37332270 | Enhancer |
| CCB | *CACNB1* | 17 | 37333681-37333850 | Enhancer |
| CCB | *CACNB1* | 17 | 37395941-37396110 | Enhancer |
| CCB | *CACNB1* | 17 | 37364621-37367146 | Enhancer |
| CCB | *CACNB1* | 17 | 37427341-37427490 | Enhancer |
| CCB | *CACNB1* | 17 | 37326315-37327866 | Enhancer |
| CCB | *CACNB1* | 17 | 37325454-37325653 | Enhancer |
| CCB | *CACNB1* | 17 | 37328301-37328450 | Enhancer |
| CCB | *CACNB1* | 17 | 37329881-37330030 | Enhancer |
| CCB | *CACNB1* | 17 | 37420122-37420730 | Enhancer |
| CCB | *CACNB1* | 17 | 37566364-37566971 | Enhancer |
| CCB | *CACNB1* | 17 | 37749329-37749495 | Enhancer |
| CCB | *CACNB1* | 17 | 37334901-37335070 | Enhancer |
| CCB | *CACNB1* | 17 | 37334100-37334787 | Enhancer |
| **CCB** | ***CACNB2*** | **10** | **18429606-18830798** | **Gene** |
| CCB | *CACNB2* | 10 | 18428674-18431380 | Promoter/Enhancer |
| CCB | *CACNB2* | 10 | 18688989-18690673 | Promoter/Enhancer |
| CCB | *CACNB2* | 10 | 18629529-18630129 | Promoter |
| CCB | *CACNB2* | 10 | 18549618-18549677 | Promoter |
| CCB | *CACNB2* | 10 | 18452390-18454590 | Enhancer |
| CCB | *CACNB2* | 10 | 18467498-18470172 | Enhancer |
| CCB | *CACNB2* | 10 | 18451281-18451430 | Enhancer |
| CCB | *CACNB2* | 10 | 18493061-18493210 | Enhancer |
| CCB | *CACNB2* | 10 | 18592794-18593791 | Enhancer |
| CCB | *CACNB2* | 10 | 18725955-18730327 | Enhancer |
| CCB | *CACNB2* | 10 | 18473833-18475245 | Enhancer |
| CCB | *CACNB2* | 10 | 18383961-18384110 | Enhancer |
| CCB | *CACNB2* | 10 | 18475337-18476696 | Enhancer |
| CCB | *CACNB2* | 10 | 18406301-18406450 | Enhancer |
| CCB | *CACNB2* | 10 | 18759506-18760793 | Enhancer |
| CCB | *CACNB2* | 10 | 18550812-18550951 | Enhancer |
| CCB | *CACNB2* | 10 | 18775187-18776352 | Enhancer |
| CCB | *CACNB2* | 10 | 18700589-18701887 | Enhancer |
| CCB | *CACNB2* | 10 | 18578722-18579070 | Enhancer |
| CCB | *CACNB2* | 10 | 18433444-18435058 | Enhancer |
| CCB | *CACNB2* | 10 | 18447501-18448892 | Enhancer |
| CCB | *CACNB2* | 10 | 18446528-18447484 | Enhancer |
| CCB | *CACNB2* | 10 | 18534018-18534028 | Enhancer |
| CCB | *CACNB2* | 10 | 18435549-18436688 | Enhancer |
| CCB | *CACNB2* | 10 | 18504001-18504170 | Enhancer |
| CCB | *CACNB2* | 10 | 18486006-18486731 | Enhancer |
| CCB | *CACNB2* | 10 | 18707269-18708134 | Enhancer |
| CCB | *CACNB2* | 10 | 18527921-18528030 | Enhancer |
| CCB | *CACNB2* | 10 | 18522241-18522992 | Enhancer |
| CCB | *CACNB2* | 10 | 18505561-18505670 | Enhancer |
| CCB | *CACNB2* | 10 | 18601402-18602376 | Enhancer |
| CCB | *CACNB2* | 10 | 18436916-18441361 | Enhancer |
| CCB | *CACNB2* | 10 | 18482789-18485238 | Enhancer |
| CCB | *CACNB2* | 10 | 18333406-18335050 | Enhancer |
| CCB | *CACNB2* | 10 | 18790055-18790529 | Enhancer |
| CCB | *CACNB2* | 10 | 18691881-18692030 | Enhancer |
| CCB | *CACNB2* | 10 | 18688181-18688330 | Enhancer |
| CCB | *CACNB2* | 10 | 18567741-18568674 | Enhancer |
| CCB | *CACNB2* | 10 | 18621941-18623677 | Enhancer |
| CCB | *CACNB2* | 10 | 18762421-18762610 | Enhancer |
| CCB | *CACNB2* | 10 | 18760801-18761292 | Enhancer |
| CCB | *CACNB2* | 10 | 18712923-18713793 | Enhancer |
| CCB | *CACNB2* | 10 | 18794315-18795372 | Enhancer |

| | | | | |
|---|---|---|---|---|
| CCB | *CACNB2* | 10 | 18745609-18746373 | Enhancer |
| CCB | *CACNB2* | 10 | 18514101-18514250 | Enhancer |
| CCB | *CACNB2* | 10 | 18348427-18349711 | Enhancer |
| CCB | *CACNB2* | 10 | 18583664-18583897 | Enhancer |
| CCB | *CACNB2* | 10 | 18738269-18739296 | Enhancer |
| CCB | *CACNB2* | 10 | 18744481-18744630 | Enhancer |
| CCB | *CACNB2* | 10 | 18744181-18744330 | Enhancer |
| CCB | *CACNB2* | 10 | 18528875-18530208 | Enhancer |
| CCB | *CACNB2* | 10 | 18718065-18718645 | Enhancer |
| CCB | *CACNB2* | 10 | 18751830-18756160 | Enhancer |
| CCB | *CACNB2* | 10 | 18749922-18751579 | Enhancer |
| CCB | *CACNB2* | 10 | 18579741-18580614 | Enhancer |
| CCB | *CACNB2* | 10 | 18567265-18567424 | Enhancer |
| CCB | *CACNB2* | 10 | 18648500-18649316 | Enhancer |
| CCB | *CACNB2* | 10 | 18702212-18703692 | Enhancer |
| CCB | *CACNB2* | 10 | 18703721-18704168 | Enhancer |
| CCB | *CACNB2* | 10 | 18778915-18780659 | Enhancer |
| CCB | *CACNB2* | 10 | 18787912-18789147 | Enhancer |
| CCB | *CACNB2* | 10 | 18792942-18793535 | Enhancer |
| CCB | *CACNB2* | 10 | 18817341-18817490 | Enhancer |
| CCB | *CACNB2* | 10 | 18816741-18816890 | Enhancer |
| CCB | *CACNB2* | 10 | 18740308-18740990 | Enhancer |
| CCB | *CACNB2* | 10 | 18741628-18742721 | Enhancer |
| CCB | *CACNB2* | 10 | 18732026-18732639 | Enhancer |
| CCB | *CACNB2* | 10 | 18723647-18724379 | Enhancer |
| **CCB** | ***CACNB3*** | **12** | **49207577-49222726** | **Gene** |
| CCB | *CACNB3* | 12 | 49203763-49210435 | Promoter/Enhancer |
| CCB | *CACNB3* | 12 | 49211575-49213450 | Promoter/Enhancer |
| CCB | *CACNB3* | 12 | 49448916-49455655 | Promoter/Enhancer |
| CCB | *CACNB3* | 12 | 49108721-49111783 | Promoter/Enhancer |
| CCB | *CACNB3* | 12 | 49522862-49527437 | Promoter/Enhancer |
| CCB | *CACNB3* | 12 | 49657029-49663594 | Promoter/Enhancer |
| CCB | *CACNB3* | 12 | 49180347-49184467 | Promoter/Enhancer |
| CCB | *CACNB3* | 12 | 48225635-48230585 | Enhancer |
| CCB | *CACNB3* | 12 | 49687781-49689222 | Promoter/Enhancer |
| CCB | *CACNB3* | 12 | 49147321-49147490 | Enhancer |
| CCB | *CACNB3* | 12 | 49173178-49175941 | Enhancer |
| CCB | *CACNB3* | 12 | 49159161-49159930 | Enhancer |
| CCB | *CACNB3* | 12 | 49580149-49584425 | Promoter/Enhancer |
| CCB | *CACNB3* | 12 | 49162061-49162210 | Enhancer |
| CCB | *CACNB3* | 12 | 49144534-49144559 | Enhancer |
| CCB | *CACNB3* | 12 | 49160229-49161861 | Enhancer |
| CCB | *CACNB3* | 12 | 49179679-49180345 | Enhancer |
| CCB | *CACNB3* | 12 | 49217901-49218190 | Enhancer |
| CCB | *CACNB3* | 12 | 49267159-49267283 | Enhancer |
| **CCB** | ***CACNB4*** | **2** | **152689285-152955593** | **Gene** |
| CCB | *CACNB4* | 2 | 152954056-152956046 | Promoter/Enhancer |
| CCB | *CACNB4* | 2 | 152830115-152830847 | Promoter/Enhancer |
| CCB | *CACNB4* | 2 | 152918555-152920030 | Enhancer |
| CCB | *CACNB4* | 2 | 152894160-152895545 | Enhancer |
| CCB | *CACNB4* | 2 | 152851019-152853401 | Enhancer |
| CCB | *CACNB4* | 2 | 152910872-152912587 | Enhancer |
| CCB | *CACNB4* | 2 | 152855681-152855810 | Enhancer |
| CCB | *CACNB4* | 2 | 152882049-152882769 | Enhancer |
| CCB | *CACNB4* | 2 | 152779338-152780439 | Enhancer |
| CCB | *CACNB4* | 2 | 152803715-152805070 | Enhancer |
| CCB | *CACNB4* | 2 | 152906315-152907630 | Enhancer |
| CCB | *CACNB4* | 2 | 153308651-153311667 | Enhancer |
| CCB | *CACNB4* | 2 | 152564775-152568412 | Enhancer |
| CCB | *CACNB4* | 2 | 152573345-152574228 | Enhancer |
| CCB | *CACNB4* | 2 | 152700971-152701203 | Enhancer |
| CCB | *CACNB4* | 2 | 153319291-153319615 | Enhancer |
| CCB | *CACNB4* | 2 | 152757130-152757527 | Enhancer |
| CCB | *CACNB4* | 2 | 152860915-152861914 | Enhancer |
| CCB | *CACNB4* | 2 | 152945115-152946314 | Enhancer |

| | | | | |
|---|---|---|---|---|
| CCB | *CACNB4* | 2 | 152899381-152899610 | Enhancer |
| CCB | *CACNB4* | 2 | 152036987-152037136 | Enhancer |
| CCB | *CACNB4* | 2 | 152735004-152736098 | Enhancer |
| CCB | *CACNB4* | 2 | 152742479-152743543 | Enhancer |
| CCB | *CACNB4* | 2 | 152887915-152888514 | Enhancer |
| CCB | *CACNB4* | 2 | 152971717-152971973 | Enhancer |
| CCB | *CACNB4* | 2 | 152699171-152700353 | Enhancer |
| CCB | *CACNB4* | 2 | 152714201-152714370 | Enhancer |
| CCB | *CACNB4* | 2 | 152789657-152790028 | Enhancer |
| CCB | *CACNB4* | 2 | 152748315-152748714 | Enhancer |
| CCB | *CACNB4* | 2 | 152774315-152775314 | Enhancer |
| CCB | *CACNB4* | 2 | 152800230-152802596 | Enhancer |
| CCB | *CACNB4* | 2 | 152800061-152800210 | Enhancer |
| **CCB** | ***CACNG1*** | **17** | **65040652-65052913** | **Gene** |
| CCB | *CACNG1* | 17 | 65038563-65044391 | Promoter/Enhancer |
| CCB | *CACNG1* | 17 | 65239492-65244198 | Promoter/Enhancer |
| CCB | *CACNG1* | 17 | 65049530-65051511 | Enhancer |
| CCB | *CACNG1* | 17 | 65106597-65109013 | Enhancer |
| CCB | *CACNG1* | 17 | 65090722-65092662 | Enhancer |
| CCB | *CACNG1* | 17 | 65253360-65257469 | Enhancer |
| CCB | *CACNG1* | 17 | 65154098-65154970 | Enhancer |
| CCB | *CACNG1* | 17 | 65276135-65278586 | Enhancer |
| CCB | *CACNG1* | 17 | 65052236-65053547 | Enhancer |
| CCB | *CACNG1* | 17 | 65234500-65237415 | Enhancer |
| CCB | *CACNG1* | 17 | 65045559-65048695 | Enhancer |
| CCB | *CACNG1* | 17 | 65103906-65106201 | Enhancer |
| CCB | *CACNG1* | 17 | 65211601-65212541 | Enhancer |
| CCB | *CACNG1* | 17 | 65072516-65072985 | Enhancer |
| CCB | *CACNG1* | 17 | 65055208-65056211 | Enhancer |
| **CCB** | ***CACNA1C*** | **12** | **2079952-2807115** | **Gene** |
| CCB | *CACNA1C* | 12 | 2161566-2164566 | Promoter/Enhancer |
| CCB | *CACNA1C* | 12 | 2081194-2082566 | Enhancer |
| CCB | *CACNA1C* | 12 | 2079295-2081006 | Enhancer |
| CCB | *CACNA1C* | 12 | 2691972-2692849 | Enhancer |
| CCB | *CACNA1C* | 12 | 2143967-2144936 | Enhancer |
| CCB | *CACNA1C* | 12 | 2183654-2186279 | Enhancer |
| CCB | *CACNA1C* | 12 | 2372777-2379299 | Enhancer |
| CCB | *CACNA1C* | 12 | 2216896-2226195 | Enhancer |
| CCB | *CACNA1C* | 12 | 1767798-1773105 | Enhancer |
| CCB | *CACNA1C* | 12 | 2161154-2161552 | Enhancer |
| CCB | *CACNA1C* | 12 | 2419663-2421680 | Enhancer |
| CCB | *CACNA1C* | 12 | 2228156-2230922 | Enhancer |
| CCB | *CACNA1C* | 12 | 2206541-2209473 | Enhancer |
| CCB | *CACNA1C* | 12 | 2364427-2364812 | Enhancer |
| CCB | *CACNA1C* | 12 | 2444050-2449090 | Enhancer |
| CCB | *CACNA1C* | 12 | 2181896-2183390 | Enhancer |
| CCB | *CACNA1C* | 12 | 2210028-2211778 | Enhancer |
| CCB | *CACNA1C* | 12 | 2200243-2201838 | Enhancer |
| CCB | *CACNA1C* | 12 | 2112379-2114467 | Promoter/Enhancer |
| CCB | *CACNA1C* | 12 | 2101946-2105129 | Enhancer |
| CCB | *CACNA1C* | 12 | 2044799-2047397 | Enhancer |
| CCB | *CACNA1C* | 12 | 2118861-2119010 | Enhancer |
| CCB | *CACNA1C* | 12 | 2121501-2121650 | Enhancer |
| CCB | *CACNA1C* | 12 | 2048444-2049910 | Enhancer |
| CCB | *CACNA1C* | 12 | 2122654-2123350 | Enhancer |
| CCB | *CACNA1C* | 12 | 2075648-2076741 | Enhancer |
| CCB | *CACNA1C* | 12 | 2058121-2060041 | Enhancer |
| CCB | *CACNA1C* | 12 | 2055375-2057136 | Enhancer |
| CCB | *CACNA1C* | 12 | 2120201-2121450 | Enhancer |
| CCB | *CACNA1C* | 12 | 2117981-2118130 | Enhancer |
| CCB | *CACNA1C* | 12 | 2173006-2173941 | Enhancer |
| CCB | *CACNA1C* | 12 | 2082932-2084071 | Enhancer |
| CCB | *CACNA1C* | 12 | 2069591-2073188 | Enhancer |
| CCB | *CACNA1C* | 12 | 2089853-2091243 | Enhancer |
| CCB | *CACNA1C* | 12 | 2125886-2127966 | Enhancer |

| | | | | |
|---|---|---|---|---|
| CCB | *CACNA1C* | 12 | 2166481-2166670 | Enhancer |
| CCB | *CACNA1C* | 12 | 2466656-2469074 | Enhancer |
| CCB | *CACNA1C* | 12 | 2403410-2405816 | Enhancer |
| CCB | *CACNA1C* | 12 | 2356637-2359010 | Enhancer |
| CCB | *CACNA1C* | 12 | 2057237-2057501 | Enhancer |
| CCB | *CACNA1C* | 12 | 2431967-2434188 | Enhancer |
| CCB | *CACNA1C* | 12 | 2352323-2354776 | Enhancer |
| CCB | *CACNA1C* | 12 | 2365074-2366654 | Enhancer |
| CCB | *CACNA1C* | 12 | 2391640-2398088 | Enhancer |
| CCB | *CACNA1C* | 12 | 2450181-2451861 | Enhancer |
| CCB | *CACNA1C* | 12 | 2164767-2165166 | Enhancer |
| CCB | *CACNA1C* | 12 | 2277277-2279377 | Enhancer |
| CCB | *CACNA1C* | 12 | 2271349-2274081 | Enhancer |
| CCB | *CACNA1C* | 12 | 2322103-2323450 | Enhancer |
| CCB | *CACNA1C* | 12 | 2110661-2111403 | Enhancer |
| CCB | *CACNA1C* | 12 | 2288839-2290765 | Enhancer |
| CCB | *CACNA1C* | 12 | 2347936-2349143 | Enhancer |
| CCB | *CACNA1C* | 12 | 2415078-2416557 | Enhancer |
| CCB | *CACNA1C* | 12 | 2306940-2308560 | Enhancer |
| CCB | *CACNA1C* | 12 | 2292498-2294028 | Enhancer |
| CCB | *CACNA1C* | 12 | 2453367-2455170 | Enhancer |
| CCB | *CACNA1C* | 12 | 2084572-2086775 | Enhancer |
| CCB | *CACNA1C* | 12 | 2087127-2087591 | Enhancer |
| CCB | *CACNA1C* | 12 | 2095160-2096573 | Enhancer |
| CCB | *CACNA1C* | 12 | 2139487-2143377 | Enhancer |
| CCB | *CACNA1C* | 12 | 2175103-2177148 | Enhancer |
| CCB | *CACNA1C* | 12 | 2262602-2264090 | Enhancer |
| CCB | *CACNA1C* | 12 | 2193334-2194490 | Enhancer |
| CCB | *CACNA1C* | 12 | 2409918-2412424 | Enhancer |
| CCB | *CACNA1C* | 12 | 2158809-2159784 | Enhancer |
| CCB | *CACNA1C* | 12 | 2359329-2360982 | Enhancer |
| CCB | *CACNA1C* | 12 | 2428657-2429566 | Enhancer |
| CCB | *CACNA1C* | 12 | 2400461-2400630 | Enhancer |
| CCB | *CACNA1C* | 12 | 2119384-2120057 | Enhancer |
| CCB | *CACNA1C* | 12 | 2203341-2204336 | Enhancer |
| CCB | *CACNA1C* | 12 | 2170143-2171251 | Enhancer |
| CCB | *CACNA1C* | 12 | 2281781-2281930 | Enhancer |
| CCB | *CACNA1C* | 12 | 2260436-2262389 | Enhancer |
| CCB | *CACNA1C* | 12 | 2302013-2306687 | Enhancer |
| CCB | *CACNA1C* | 12 | 2368400-2369290 | Enhancer |
| CCB | *CACNA1C* | 12 | 2442171-2442390 | Enhancer |
| CCB | *CACNA1C* | 12 | 2270781-2270930 | Enhancer |
| CCB | *CACNA1C* | 12 | 2339165-2339784 | Enhancer |
| CCB | *CACNA1C* | 12 | 2238381-2238550 | Enhancer |
| CCB | *CACNA1C* | 12 | 2268934-2269887 | Enhancer |
| CCB | *CACNA1C* | 12 | 2254615-2256378 | Enhancer |
| CCB | *CACNA1C* | 12 | 2342039-2342990 | Enhancer |
| CCB | *CACNA1C* | 12 | 2428367-2428566 | Enhancer |
| CCB | *CACNA1C* | 12 | 2427157-2427717 | Enhancer |
| CCB | *CACNA1C* | 12 | 2398761-2400405 | Enhancer |
| CCB | *CACNA1C* | 12 | 2335414-2336748 | Enhancer |
| CCB | *CACNA1C* | 12 | 2167767-2169880 | Enhancer |
| CCB | *CACNA1C* | 12 | 2148495-2149324 | Enhancer |
| CCB | *CACNA1C* | 12 | 2138706-2139344 | Enhancer |
| CCB | *CACNA1C* | 12 | 2171351-2172258 | Enhancer |
| CCB | *CACNA1C* | 12 | 2245167-2245366 | Enhancer |
| CCB | *CACNA1C* | 12 | 2246831-2248330 | Enhancer |
| CCB | *CACNA1C* | 12 | 2280498-2280632 | Enhancer |
| CCB | *CACNA1C* | 12 | 2281401-2281550 | Enhancer |
| CCB | *CACNA1C* | 12 | 2265561-2265750 | Enhancer |
| CCB | *CACNA1C* | 12 | 2407617-2408956 | Enhancer |
| CCB | *CACNA1C* | 12 | 2192366-2192566 | Enhancer |
| CCB | *CACNA1C* | 12 | 2274261-2274410 | Enhancer |
| CCB | *CACNA1C* | 12 | 2281201-2281350 | Enhancer |
| CCB | *CACNA1C* | 12 | 2177211-2178762 | Enhancer |

| | | | | |
|---|---|---|---|---|
| CCB | *CACNA1C* | 12 | 2442567-2442967 | Enhancer |
| CCB | *CACNA1C* | 12 | 2441541-2441690 | Enhancer |
| CCB | *CACNA1C* | 12 | 2451967-2452766 | Enhancer |
| CCB | *CACNA1C* | 12 | 2416801-2416950 | Enhancer |
| CCB | *CACNA1C* | 12 | 2323730-2325500 | Enhancer |
| CCB | *CACNA1C* | 12 | 2349249-2351414 | Enhancer |
| CCB | *CACNA1C* | 12 | 2380803-2386142 | Enhancer |
| CCB | *CACNA1C* | 12 | 2336889-2337604 | Enhancer |
| CCB | *CACNA1C* | 12 | 2457223-2458492 | Enhancer |
| CCB | *CACNA1C* | 12 | 2249789-2251721 | Enhancer |
| CCB | *CACNA1C* | 12 | 2294781-2295709 | Enhancer |
| CCB | *CACNA1C* | 12 | 2186647-2187626 | Enhancer |
| CCB | *CACNA1C* | 12 | 2438360-2439517 | Enhancer |
| CCB | *CACNA1C* | 12 | 2407095-2407603 | Enhancer |
| CCB | *CACNA1C* | 12 | 2386355-2387332 | Enhancer |
| CCB | *CACNA1C* | 12 | 2328750-2331231 | Enhancer |
| CCB | *CACNA1C* | 12 | 2400774-2402962 | Enhancer |
| CCB | *CACNA1C* | 12 | 2245404-2246734 | Enhancer |
| CCB | *CACNA1C* | 12 | 2274438-2275487 | Enhancer |
| CCB | *CACNA1C* | 12 | 2195393-2196970 | Enhancer |
| CCB | *CACNA1C* | 12 | 2233400-2237679 | Enhancer |
| CCB | *CACNA1C* | 12 | 2283997-2286741 | Enhancer |
| CCB | *CACNA1C* | 12 | 2287176-2288132 | Enhancer |
| CCB | *CACNA1C* | 12 | 2258324-2259667 | Enhancer |
| CCB | *CACNA1C* | 12 | 2343203-2345601 | Enhancer |
| CCB | *CACNA1C* | 12 | 2299019-2299959 | Enhancer |
| CCB | *CACNA1C* | 12 | 2412745-2414119 | Enhancer |
| CCB | *CACNA1C* | 12 | 2188468-2189111 | Enhancer |
| CCB | *CACNA1C* | 12 | 2434584-2435528 | Enhancer |
| CCB | *CACNA1C* | 12 | 2316947-2318292 | Enhancer |
| CCB | *CACNA1C* | 12 | 2333218-2334248 | Enhancer |
| CCB | *CACNA1C* | 12 | 2331290-2332420 | Enhancer |
| CCB | *CACNA1C* | 12 | 2440488-2441051 | Enhancer |
| CCB | *CACNA1C* | 12 | 2320137-2320914 | Enhancer |
| CCB | *CACNA1C* | 12 | 2326691-2328736 | Enhancer |
| CCB | *CACNA1C* | 12 | 2649836-2650311 | Enhancer |
| CCB | *CACNA1C* | 12 | 2506981-2507050 | Enhancer |
| CCB | *CACNA1C* | 12 | 2659879-2661404 | Enhancer |
| CCB | *CACNA1C* | 12 | 2515793-2516578 | Enhancer |
| CCB | *CACNA1C* | 12 | 2522460-2525715 | Enhancer |
| CCB | *CACNA1C* | 12 | 2526621-2527392 | Enhancer |
| CCB | *CACNA1C* | 12 | 2695181-2696063 | Enhancer |
| CCB | *CACNA1C* | 12 | 2543714-2545518 | Enhancer |
| CCB | *CACNA1C* | 12 | 2721754-2724057 | Enhancer |
| CCB | *CACNA1C* | 12 | 2547538-2548942 | Enhancer |
| CCB | *CACNA1C* | 12 | 2497459-2500942 | Enhancer |
| CCB | *CACNA1C* | 12 | 2733321-2734829 | Enhancer |
| CCB | *CACNA1C* | 12 | 2555271-2555641 | Enhancer |
| CCB | *CACNA1C* | 12 | 2558507-2559370 | Enhancer |
| CCB | *CACNA1C* | 12 | 2649619-2649812 | Enhancer |
| CCB | *CACNA1C* | 12 | 2736852-2737810 | Enhancer |
| CCB | *CACNA1C* | 12 | 2504526-2506285 | Enhancer |
| CCB | *CACNA1C* | 12 | 2740641-2741724 | Enhancer |
| CCB | *CACNA1C* | 12 | 2511126-2512729 | Enhancer |
| CCB | *CACNA1C* | 12 | 2512831-2514124 | Enhancer |
| CCB | *CACNA1C* | 12 | 2565222-2568247 | Enhancer |
| CCB | *CACNA1C* | 12 | 2749071-2750838 | Enhancer |
| CCB | *CACNA1C* | 12 | 2518209-2521853 | Enhancer |
| CCB | *CACNA1C* | 12 | 2463531-2463940 | Enhancer |
| CCB | *CACNA1C* | 12 | 2792481-2792530 | Enhancer |
| CCB | *CACNA1C* | 12 | 2792101-2792350 | Enhancer |
| CCB | *CACNA1C* | 12 | 2791561-2791670 | Enhancer |
| CCB | *CACNA1C* | 12 | 2791761-2791970 | Enhancer |
| CCB | *CACNA1C* | 12 | 2473710-2474314 | Enhancer |
| CCB | *CACNA1C* | 12 | 2799725-2801764 | Promoter/Enhancer |

| | | | | |
|---|---|---|---|---|
| CCB | *CACNA1C* | 12 | 2603721-2603850 | Enhancer |
| CCB | *CACNA1C* | 12 | 2724729-2726058 | Enhancer |
| CCB | *CACNA1C* | 12 | 2500386-2500884 | Enhancer |
| CCB | *CACNA1C* | 12 | 2552550-2553765 | Enhancer |
| CCB | *CACNA1C* | 12 | 2561981-2563825 | Enhancer |
| CCB | *CACNA1C* | 12 | 2559627-2561813 | Enhancer |
| CCB | *CACNA1C* | 12 | 2488061-2488210 | Enhancer |
| CCB | *CACNA1C* | 12 | 2531341-2532583 | Enhancer |
| CCB | *CACNA1C* | 12 | 2537218-2542473 | Enhancer |
| CCB | *CACNA1C* | 12 | 2489067-2491096 | Enhancer |
| CCB | *CACNA1C* | 12 | 2471978-2473410 | Enhancer |
| CCB | *CACNA1C* | 12 | 2608221-2608370 | Enhancer |
| CCB | *CACNA1C* | 12 | 2613061-2613207 | Enhancer |
| CCB | *CACNA1C* | 12 | 2610308-2611812 | Enhancer |
| CCB | *CACNA1C* | 12 | 2481105-2484809 | Enhancer |
| CCB | *CACNA1C* | 12 | 2486081-2486390 | Enhancer |
| CCB | *CACNA1C* | 12 | 2491894-2495082 | Enhancer |
| **TD** | ***SLC12A3*** | **16** | **56899119-56949762** | **Gene** |
| TD | *SLC12A3* | 16 | 56899070-56899129 | Promoter |
| TD | *SLC12A3* | 16 | 56964403-56974458 | Promoter/Enhancer |
| TD | *SLC12A3* | 16 | 56839748-56841942 | Enhancer |
| TD | *SLC12A3* | 16 | 56834256-56834865 | Enhancer |
| TD | *SLC12A3* | 16 | 56893506-56897709 | Enhancer |
| TD | *SLC12A3* | 16 | 56903512-56904197 | Enhancer |
| TD | *SLC12A3* | 16 | 56901241-56901712 | Enhancer |
| TD | *SLC12A3* | 16 | 56905112-56905513 | Enhancer |
| TD | *SLC12A3* | 16 | 56948424-56953992 | Enhancer |
| TD | *SLC12A3* | 16 | 56942554-56948010 | Enhancer |
| TD | *SLC12A3* | 16 | 56908210-56909649 | Enhancer |
| TD | *SLC12A3* | 16 | 56932921-56933070 | Enhancer |
| TD | *SLC12A3* | 16 | 56922841-56922990 | Enhancer |

Supplementary Table 3. Main instrument single-nucleotide polymorphism (SNP) for angiotensin-converting-enzyme inhibitors (ACEIs).

| SNP | Chr | Pos | EA | OA | EAF | Effect | SE | P value | Total sample size | $R^2$ | F statistic |
|---|---|---|---|---|---|---|---|---|---|---|---|
| rs4291 | 17 | 61554194 | a | t | 0.6155 | -0.2839 | 0.0312 | 8.65E-20 | 745820 | 3.69E-04 | 276 |

Chr: chromosome, Pos: position (hg19), EA: effect allele, OA: other allele, EAF: effect allele frequency, SE: standard error, $R^2$: proportion of variance explained.

| Supplementary Table 4. Main instrument single-nucleotide polymorphisms (SNPs) for beta-blockers (BBs). | | | | | | | | | | | |
|---|---|---|---|---|---|---|---|---|---|---|---|
| SNP | Chr | Pos | EA | OA | EAF | Effect | SE | P value | Total sample size | $R^2$ | F statistic |
| rs11196549 | 10 | 115707298 | a | g | 0.0425 | 0.6884 | 0.0784 | 1.58E-18 | 738169 | 1.54E-04 | 114 |
| rs460718 | 10 | 115721364 | a | g | 0.3266 | -0.2764 | 0.0324 | 1.36E-17 | 738169 | 3.34E-04 | 247 |
| rs11196597 | 10 | 115788094 | a | g | 0.1330 | 0.2858 | 0.0458 | 4.23E-10 | 737164 | 1.81E-04 | 134 |
| rs17875473 | 10 | 115800294 | t | c | 0.0871 | 0.3283 | 0.0552 | 2.66E-09 | 738170 | 1.43E-04 | 106 |
| rs1801253 | 10 | 115805056 | c | g | 0.7338 | 0.4626 | 0.0344 | 2.84E-41 | 738169 | 4.97E-04 | 367 |
| rs4359161 | 10 | 115826508 | a | g | 0.1812 | -0.2662 | 0.0391 | 9.46E-12 | 738168 | 2.17E-04 | 160 |

Chr: chromosome, Pos: position (hg19), EA: effect allele, OA: other allele, EAF: effect allele frequency, SE: standard error, $R^2$: proportion of variance explained.

Supplementary Table 5. Main instrument single-nucleotide polymorphisms (SNPs) for calcium channel blockers (CCBs).

| SNP | Chr | Pos | EA | OA | EAF | Effect | SE | P value | Total sample size | $R^2$ | F statistic |
|---|---|---|---|---|---|---|---|---|---|---|---|
| rs3821843 | 3 | 53558012 | a | g | 0.6808 | 0.3373 | 0.0335 | 6.56E-24 | 736049 | 4.03E-04 | 297 |
| rs114987861 | 3 | 53605712 | a | g | 0.0284 | 0.5289 | 0.0958 | 3.36E-08 | 737054 | 8.02E-05 | 59 |
| rs113210396 | 3 | 53612327 | t | g | 0.0451 | -0.4338 | 0.0770 | 1.76E-08 | 737164 | 1.03E-04 | 76 |
| rs7340705 | 3 | 53734443 | t | c | 0.6732 | -0.2425 | 0.0322 | 4.87E-14 | 738169 | 2.93E-04 | 217 |
| rs2488136 | 10 | 18334521 | a | g | 0.2875 | 0.2261 | 0.0334 | 1.22E-11 | 738169 | 2.55E-04 | 188 |
| rs1888693 | 10 | 18440444 | a | g | 0.3449 | 0.3858 | 0.0317 | 4.69E-34 | 736050 | 4.79E-04 | 353 |
| rs16916914 | 10 | 18457722 | t | c | 0.9631 | -0.5636 | 0.0806 | 2.72E-12 | 737424 | 1.10E-04 | 81 |
| rs7076319 | 10 | 18459450 | a | g | 0.7339 | -0.3210 | 0.0341 | 5.07E-21 | 737054 | 3.45E-04 | 254 |
| rs61278674 | 10 | 18481737 | a | g | 0.9062 | -0.3298 | 0.0540 | 1.03E-09 | 737163 | 1.54E-04 | 114 |
| rs1779209 | 10 | 18514561 | t | c | 0.2876 | 0.2736 | 0.0336 | 4.23E-16 | 729448 | 3.08E-04 | 225 |
| rs10828399 | 10 | 18553968 | a | g | 0.5218 | -0.1947 | 0.0302 | 1.10E-10 | 738168 | 2.67E-04 | 197 |
| rs10828452 | 10 | 18592450 | a | t | 0.7930 | 0.3046 | 0.0388 | 4.20E-15 | 737164 | 2.75E-04 | 203 |
| rs10828542 | 10 | 18627285 | a | g | 0.6137 | 0.1817 | 0.0311 | 5.18E-09 | 738170 | 2.37E-04 | 175 |
| rs12780039 | 10 | 18678987 | c | g | 0.1210 | 0.2852 | 0.0470 | 1.26E-09 | 738167 | 1.67E-04 | 123 |
| rs112133583 | 10 | 18695681 | t | c | 0.0299 | -0.5546 | 0.0973 | 1.18E-08 | 737169 | 8.84E-05 | 65 |
| rs11014170 | 10 | 18710991 | a | g | 0.0206 | -0.6701 | 0.1150 | 5.61E-09 | 732148 | 7.43E-05 | 54 |
| rs7923191 | 10 | 18727901 | a | g | 0.7918 | -0.3690 | 0.0376 | 1.10E-22 | 737054 | 3.34E-04 | 246 |
| rs12258967 | 10 | 18727959 | c | g | 0.7047 | 0.6327 | 0.0337 | 1.08E-78 | 737165 | 7.24E-04 | 534 |
| rs72786098 | 10 | 18729855 | a | g | 0.0322 | -0.5033 | 0.0883 | 1.18E-08 | 737055 | 8.62E-05 | 64 |
| rs1998822 | 10 | 18755664 | a | g | 0.7234 | -0.1958 | 0.0343 | 1.15E-08 | 727331 | 2.15E-04 | 157 |
| rs4748474 | 10 | 18790727 | a | g | 0.5214 | 0.1946 | 0.0304 | 1.61E-10 | 729908 | 2.67E-04 | 195 |
| rs150857355 | 12 | 49209340 | c | g | 0.0217 | 0.9406 | 0.1122 | 5.20E-17 | 731300 | 1.10E-04 | 80 |
| rs2239046 | 12 | 2434419 | a | g | 0.6817 | 0.2082 | 0.0322 | 9.58E-11 | 745818 | 2.48E-04 | 185 |
| rs714277 | 12 | 2514270 | t | c | 0.2834 | 0.1986 | 0.0333 | 2.38E-09 | 745820 | 2.22E-04 | 165 |

Chr: chromosome, Pos: position (hg19), EA: effect allele, OA: other allele, EAF: effect allele frequency, SE: standard error, $R^2$: proportion of variance explained.

| Supplementary Table 6. Mendelian randomization relative risk estimates for different disease incidence rates | | | | | | | | | | | | | |
|---|---|---|---|---|---|---|---|---|---|---|---|---|---|
| | | Odds ratio | | | 1% incidence | | | 5% incidence | | | 10% incidence | | |
| Drug | Outcome | Odds ratio | Low 95% CI | Upp 95% CI | Relative risk | Low 95% CI | Upp 95% CI | Relative risk | Low 95% CI | Upp 95% CI | Relative risk | Low 95% CI | Upp 95% CI |
| ACEI | CHD | 0.66 | 0.16 | 2.75 | 0.66 | 0.16 | 2.70 | 0.67 | 0.16 | 2.53 | 0.68 | 0.17 | 2.34 |
| ACEI | Stroke | 0.21 | 0.06 | 0.72 | 0.21 | 0.06 | 0.72 | 0.22 | 0.06 | 0.73 | 0.23 | 0.07 | 0.74 |
| BB | CHD | 0.61 | 0.46 | 0.80 | 0.61 | 0.46 | 0.80 | 0.62 | 0.47 | 0.81 | 0.63 | 0.49 | 0.82 |
| BB | Stroke | 0.91 | 0.72 | 1.15 | 0.91 | 0.72 | 1.14 | 0.91 | 0.73 | 1.14 | 0.92 | 0.74 | 1.13 |
| CCB | CHD | 0.72 | 0.63 | 0.83 | 0.72 | 0.63 | 0.83 | 0.73 | 0.64 | 0.84 | 0.74 | 0.65 | 0.84 |
| CCB | Stroke | 0.74 | 0.66 | 0.84 | 0.74 | 0.66 | 0.84 | 0.75 | 0.67 | 0.84 | 0.76 | 0.68 | 0.85 |

ACEI: angiotensin-converting-enzyme inhibitor; BB: beta-blocker; CCB: calcium channel blocker

| Supplementary Table 7. Sensitivity analysis instrument single-nucleotide polymorphisms (SNPs) for beta-blockers (BBs) using genetic association estimates that did not correct for medication use or adjust for body mass index. | | | | | | | | |
|---|---|---|---|---|---|---|---|---|
| SNP | Chr | Pos | EA | OA | Effect | SE | P value | Total sample size |
| rs151597 | 10 | 115720514 | c | g | 0.0160 | 0.0026 | 1.23E-09 | 317754 |
| rs1801253 | 10 | 115805056 | c | g | 0.0182 | 0.0028 | 9.24E-11 | 317754 |

Chr: chromosome, Pos: position (hg19), EA: effect allele, OA: other allele, SE: standard error.

| Supplementary Table 8. Sensitivity analysis instrument single-nucleotide polymorphisms (SNPs) for calcium channel blockers (CCBs) using genetic association estimates that did not correct for medication use or adjust for body mass index. | | | | | | | | |
|---|---|---|---|---|---|---|---|---|
| SNP | Chr | Pos | EA | OA | Effect | SE | P value | Total sample size |
| rs10741083 | 10 | 18790858 | c | t | 0.0142 | 0.0026 | 2.64E-08 | 317754 |
| rs12258967 | 10 | 18727959 | g | c | -0.0314 | 0.0027 | 1.73E-31 | 317754 |
| rs17604757 | 10 | 18442940 | g | a | 0.0318 | 0.0050 | 1.78E-10 | 317754 |
| rs1779240 | 10 | 18476313 | a | g | -0.0189 | 0.0029 | 6.89E-11 | 317754 |
| rs10828650 | 10 | 18691531 | g | a | 0.0185 | 0.0026 | 1.56E-12 | 317754 |
| rs35593046 | 3 | 53553923 | t | g | -0.0164 | 0.0028 | 6.23E-09 | 317754 |

Chr: chromosome, Pos: position (hg19), EA: effect allele, OA: other allele, SE: standard error.

Supplementary Table 9. Possible pleiotropic effects related to the angiotensin-converting-enzyme inhibitor (ACEI), beta-blocker (BB) and calcium channel blocker (CCB) genetic variants, as identified using PhenoScanner.

| Drug | Instrument SNP | Position (hg19) | Effect allele | Other allele | SNP | Position (hg19) | Effect allele | Other allele | Proxy | $r^2$ | Trait | Study | PMID | Ancestry | Year | Beta | Standard error | P | Total sample size | Cases | Controls | Unit |
|---|---|---|---|---|---|---|---|---|---|---|---|---|---|---|---|---|---|---|---|---|---|---|
| ACEI | rs4291 | chr17:61554194 | A | T | rs4291 | chr17:61554194 | A | T | No | 1.00 | Medication for cholesterol, blood pressure or diabetes: blood pressure medication | Neale B | UKBB | European | 2017 | -0.0097 | 0.0016 | 1.71E-09 | 154702 | 38548 | 116154 | - |
| | rs4291 | chr17:61554194 | A | T | rs4291 | chr17:61554194 | A | T | No | 1.00 | Medication for cholesterol, blood pressure or diabetes: none of the above | Neale B | UKBB | European | 2017 | 0.0104 | 0.0018 | 3.80E-09 | 154702 | 103004 | 51698 | - |
| | rs4291 | chr17:61554194 | A | T | rs4291 | chr17:61554194 | A | T | No | 1.00 | Self-reported hypertension | Neale B | UKBB | European | 2017 | -0.0087 | 0.0011 | 3.90E-15 | 337159 | 87690 | 249469 | - |
| | rs4291 | chr17:61554194 | A | T | rs4291 | chr17:61554194 | A | T | No | 1.00 | Vascular or heart problems diagnosed by doctor: high blood pressure | Neale B | UKBB | European | 2017 | -0.0088 | 0.0011 | 5.30E-15 | 336683 | 91033 | 245650 | - |
| | rs4291 | chr17:61554194 | A | T | rs4291 | chr17:61554194 | A | T | No | 1.00 | Vascular or heart problems diagnosed by doctor: none of the above | Neale B | UKBB | European | 2017 | 0.0087 | 0.0012 | 3.30E-14 | 336683 | 236530 | 100153 | - |
| | rs4291 | chr17:61554194 | A | T | rs4295 | chr17:61556298 | G | C | Yes | 0.99 | Medication for cholesterol, blood pressure or diabetes: blood pressure medication | Neale B | UKBB | European | 2017 | -0.0097 | 0.0016 | 1.84E-09 | 154702 | 38548 | 116154 | - |
| | rs4291 | chr17:61554194 | A | T | rs4295 | chr17:61556298 | G | C | Yes | 0.99 | Medication for cholesterol, blood pressure or diabetes: none of the above | Neale B | UKBB | European | 2017 | 0.0103 | 0.0018 | 4.67E-09 | 154702 | 103004 | 51698 | - |
| | rs4291 | chr17:61554194 | A | T | rs4295 | chr17:61556298 | G | C | Yes | 0.99 | Self-reported hypertension | Neale B | UKBB | European | 2017 | -0.0087 | 0.0011 | 3.58E-15 | 337159 | 87690 | 249469 | - |
| | rs4291 | chr17:61554194 | A | T | rs4295 | chr17:61556298 | G | C | Yes | 0.99 | Vascular or heart problems diagnosed by doctor: high blood pressure | Neale B | UKBB | European | 2017 | -0.0088 | 0.0011 | 5.02E-15 | 336683 | 91033 | 245650 | - |
| | rs4291 | chr17:61554194 | A | T | rs4295 | chr17:61556298 | G | C | Yes | 0.99 | Vascular or heart problems diagnosed by doctor: none of the above | Neale B | UKBB | European | 2017 | 0.0088 | 0.0012 | 2.55E-14 | 336683 | 236530 | 100153 | - |
| | rs4291 | chr17:61554194 | A | T | rs4292 | chr17:61554341 | T | C | Yes | 0.98 | Medication for cholesterol, blood pressure or diabetes: blood pressure medication | Neale B | UKBB | European | 2017 | -0.0098 | 0.0016 | 1.15E-09 | 154702 | 38548 | 116154 | - |
| | rs4291 | chr17:61554194 | A | T | rs4292 | chr17:61554341 | T | C | Yes | 0.98 | Medication for cholesterol, blood pressure or diabetes: none of the above | Neale B | UKBB | European | 2017 | 0.0104 | 0.0018 | 2.98E-09 | 154702 | 103004 | 51698 | - |
| | rs4291 | chr17:61554194 | A | T | rs4292 | chr17:61554341 | T | C | Yes | 0.98 | Self-reported hypertension | Neale B | UKBB | European | 2017 | -0.0088 | 0.0011 | 2.66E-15 | 337159 | 87690 | 249469 | - |
| | rs4291 | chr17:61554194 | A | T | rs4292 | chr17:61554341 | T | C | Yes | 0.98 | Vascular or heart problems diagnosed by doctor: high blood pressure | Neale B | UKBB | European | 2017 | -0.0088 | 0.0011 | 3.64E-15 | 336683 | 91033 | 245650 | - |
| | rs4291 | chr17:61554194 | A | T | rs4292 | chr17:61554341 | T | C | Yes | 0.98 | Vascular or heart problems diagnosed by doctor: none of the above | Neale B | UKBB | European | 2017 | 0.0088 | 0.0012 | 2.65E-14 | 336683 | 236530 | 100153 | - |
| | rs4291 | chr17:61554194 | A | T | rs4308 | chr17:61559625 | G | A | Yes | 0.98 | Diastolic blood pressure | Warren HR | 28135244 | European | 2017 | -0.2130 | 0.0285 | 7.00E-14 | - | - | - | unit increase |
| | rs4291 | chr17:61554194 | A | T | rs4308 | chr17:61559625 | G | A | Yes | 0.98 | Medication for cholesterol, blood pressure or diabetes: blood pressure medication | Neale B | UKBB | European | 2017 | -0.0099 | 0.0016 | 1.05E-09 | 154702 | 38548 | 116154 | - |
| | rs4291 | chr17:61554194 | A | T | rs4308 | chr17:61559625 | G | A | Yes | 0.98 | Medication for cholesterol, blood pressure or diabetes: none of the above | Neale B | UKBB | European | 2017 | 0.0105 | 0.0018 | 2.38E-09 | 154702 | 103004 | 51698 | - |
| | rs4291 | chr17:61554194 | A | T | rs4308 | chr17:61559625 | G | A | Yes | 0.98 | Self-reported hypertension | Neale B | UKBB | European | 2017 | -0.0087 | 0.0011 | 3.57E-15 | 337159 | 87690 | 249469 | - |
| | rs4291 | chr17:61554194 | A | T | rs4308 | chr17:61559625 | G | A | Yes | 0.98 | Vascular or heart problems diagnosed by doctor: high blood pressure | Neale B | UKBB | European | 2017 | -0.0088 | 0.0011 | 4.83E-15 | 336683 | 91033 | 245650 | - |
| | rs4291 | chr17:61554194 | A | T | rs4308 | chr17:61559625 | G | A | Yes | 0.98 | Vascular or heart problems diagnosed by doctor: none of the above | Neale B | UKBB | European | 2017 | 0.0088 | 0.0012 | 2.11E-14 | 336683 | 236530 | 100153 | - |
| | rs4291 | chr17:61554194 | A | T | rs4459609 | chr17:61548948 | A | C | Yes | 0.96 | Diastolic blood pressure | Wain LV | 28739976 | European | 2017 | 0.1980 | 0.0259 | 2.00E-14 | - | - | - | unit decrease |
| | rs4291 | chr17:61554194 | A | T | rs4459609 | chr17:61548948 | A | C | Yes | 0.96 | Medication for cholesterol, blood pressure or diabetes: blood pressure medication | Neale B | UKBB | European | 2017 | -0.0096 | 0.0016 | 1.87E-09 | 154702 | 38548 | 116154 | - |
| | rs4291 | chr17:61554194 | A | T | rs4459609 | chr17:61548948 | A | C | Yes | 0.96 | Medication for cholesterol, blood pressure or diabetes: none of the above | Neale B | UKBB | European | 2017 | 0.0104 | 0.0017 | 2.54E-09 | 154702 | 103004 | 51698 | - |
| | rs4291 | chr17:61554194 | A | T | rs4459609 | chr17:61548948 | A | C | Yes | 0.96 | Self-reported hypertension | Neale B | UKBB | European | 2017 | -0.0088 | 0.0011 | 9.45E-16 | 337159 | 87690 | 249469 | - |

| | | | | | | | | | | | | | | | | | | | | | | |
|---|---|---|---|---|---|---|---|---|---|---|---|---|---|---|---|---|---|---|---|---|---|---|
| | rs4291 | chr17:61554194 | A | T | rs4459609 | chr17:61548948 | A | C | Yes | 0.96 | Treatment with blood pressure medication | Neale B | UKBB | European | 2017 | -0.0075 | 0.0013 | 1.09E-08 | 180203 | 31488 | 148715 | - |
| | rs4291 | chr17:61554194 | A | T | rs4459609 | chr17:61548948 | A | C | Yes | 0.96 | Vascular or heart problems diagnosed by doctor: high blood pressure | Neale B | UKBB | European | 2017 | -0.0089 | 0.0011 | 1.32E-15 | 336683 | 91033 | 245650 | - |
| | rs4291 | chr17:61554194 | A | T | rs4459609 | chr17:61548948 | A | C | Yes | 0.96 | Vascular or heart problems diagnosed by doctor: none of the above | Neale B | UKBB | European | 2017 | 0.0090 | 0.0011 | 4.78E-15 | 336683 | 236530 | 100153 | - |
| BB | rs460718 | chr10:115721364 | A | G | rs460718 | chr10:115721364 | A | G | No | 1.00 | Diastolic blood pressure | Neale B | UKBB | European | 2017 | -0.0196 | 0.0026 | 8.85E-14 | 317756 | 0 | 317756 | IVNT |
| | rs460718 | chr10:115721364 | A | G | rs460718 | chr10:115721364 | A | G | No | 1.00 | Self-reported hypertension | Neale B | UKBB | European | 2017 | -0.0078 | 0.0011 | 8.18E-12 | 337159 | 87690 | 249469 | - |
| | rs460718 | chr10:115721364 | A | G | rs460718 | chr10:115721364 | A | G | No | 1.00 | Systolic blood pressure | Neale B | UKBB | European | 2017 | -0.0158 | 0.0026 | 1.82E-09 | 317754 | 0 | 317754 | IVNT |
| | rs460718 | chr10:115721364 | A | G | rs460718 | chr10:115721364 | A | G | No | 1.00 | Vascular or heart problems diagnosed by doctor: high blood pressure | Neale B | UKBB | European | 2017 | -0.0080 | 0.0012 | 3.75E-12 | 336683 | 91033 | 245650 | - |
| | rs460718 | chr10:115721364 | A | G | rs460718 | chr10:115721364 | A | G | No | 1.00 | Vascular or heart problems diagnosed by doctor: none of the above | Neale B | UKBB | European | 2017 | 0.0075 | 0.0012 | 2.58E-10 | 336683 | 236530 | 100153 | - |
| | rs460718 | chr10:115721364 | A | G | rs466328 | chr10:115721369 | G | T | Yes | 1.00 | Diastolic blood pressure | Neale B | UKBB | European | 2017 | -0.0196 | 0.0026 | 8.19E-14 | 317756 | 0 | 317756 | IVNT |
| | rs460718 | chr10:115721364 | A | G | rs466328 | chr10:115721369 | G | T | Yes | 1.00 | Self-reported hypertension | Neale B | UKBB | European | 2017 | -0.0078 | 0.0011 | 8.19E-12 | 337159 | 87690 | 249469 | - |
| | rs460718 | chr10:115721364 | A | G | rs466328 | chr10:115721369 | G | T | Yes | 1.00 | Systolic blood pressure | Neale B | UKBB | European | 2017 | -0.0158 | 0.0026 | 2.04E-09 | 317754 | 0 | 317754 | IVNT |
| | rs460718 | chr10:115721364 | A | G | rs466328 | chr10:115721369 | G | T | Yes | 1.00 | Vascular or heart problems diagnosed by doctor: high blood pressure | Neale B | UKBB | European | 2017 | -0.0080 | 0.0012 | 3.94E-12 | 336683 | 91033 | 245650 | - |
| | rs460718 | chr10:115721364 | A | G | rs466328 | chr10:115721369 | G | T | Yes | 1.00 | Vascular or heart problems diagnosed by doctor: none of the above | Neale B | UKBB | European | 2017 | 0.0075 | 0.0012 | 2.55E-10 | 336683 | 236530 | 100153 | - |
| | rs460718 | chr10:115721364 | A | G | rs467173 | chr10:115721430 | T | G | Yes | 1.00 | Diastolic blood pressure | Neale B | UKBB | European | 2017 | -0.0196 | 0.0026 | 9.09E-14 | 317756 | 0 | 317756 | IVNT |
| | rs460718 | chr10:115721364 | A | G | rs467173 | chr10:115721430 | T | G | Yes | 1.00 | Self-reported hypertension | Neale B | UKBB | European | 2017 | -0.0078 | 0.0011 | 8.30E-12 | 337159 | 87690 | 249469 | - |
| | rs460718 | chr10:115721364 | A | G | rs467173 | chr10:115721430 | T | G | Yes | 1.00 | Systolic blood pressure | Neale B | UKBB | European | 2017 | -0.0158 | 0.0026 | 1.77E-09 | 317754 | 0 | 317754 | IVNT |
| | rs460718 | chr10:115721364 | A | G | rs467173 | chr10:115721430 | T | G | Yes | 1.00 | Vascular or heart problems diagnosed by doctor: high blood pressure | Neale B | UKBB | European | 2017 | -0.0080 | 0.0012 | 3.53E-12 | 336683 | 91033 | 245650 | - |
| | rs460718 | chr10:115721364 | A | G | rs467173 | chr10:115721430 | T | G | Yes | 1.00 | Vascular or heart problems diagnosed by doctor: none of the above | Neale B | UKBB | European | 2017 | 0.0075 | 0.0012 | 2.47E-10 | 336683 | 236530 | 100153 | - |
| | rs460718 | chr10:115721364 | A | G | rs180940 | chr10:115722411 | A | G | Yes | 1.00 | Diastolic blood pressure | Neale B | UKBB | European | 2017 | -0.0198 | 0.0026 | 4.48E-14 | 317756 | 0 | 317756 | IVNT |
| | rs460718 | chr10:115721364 | A | G | rs180940 | chr10:115722411 | A | G | Yes | 1.00 | Self-reported hypertension | Neale B | UKBB | European | 2017 | -0.0079 | 0.0011 | 4.95E-12 | 337159 | 87690 | 249469 | - |
| | rs460718 | chr10:115721364 | A | G | rs180940 | chr10:115722411 | A | G | Yes | 1.00 | Systolic blood pressure | Neale B | UKBB | European | 2017 | -0.0159 | 0.0026 | 1.35E-09 | 317754 | 0 | 317754 | IVNT |
| | rs460718 | chr10:115721364 | A | G | rs180940 | chr10:115722411 | A | G | Yes | 1.00 | Vascular or heart problems diagnosed by doctor: high blood pressure | Neale B | UKBB | European | 2017 | -0.0081 | 0.0012 | 2.23E-12 | 336683 | 91033 | 245650 | - |
| | rs460718 | chr10:115721364 | A | G | rs180940 | chr10:115722411 | A | G | Yes | 1.00 | Vascular or heart problems diagnosed by doctor: none of the above | Neale B | UKBB | European | 2017 | 0.0076 | 0.0012 | 1.34E-10 | 336683 | 236530 | 100153 | - |
| | rs460718 | chr10:115721364 | A | G | rs151599 | chr10:115724039 | G | A | Yes | 1.00 | Diastolic blood pressure | Neale B | UKBB | European | 2017 | -0.0198 | 0.0026 | 5.08E-14 | 317756 | 0 | 317756 | IVNT |
| | rs460718 | chr10:115721364 | A | G | rs151599 | chr10:115724039 | G | A | Yes | 1.00 | Self-reported hypertension | Neale B | UKBB | European | 2017 | -0.0079 | 0.0011 | 5.01E-12 | 337159 | 87690 | 249469 | - |
| | rs460718 | chr10:115721364 | A | G | rs151599 | chr10:115724039 | G | A | Yes | 1.00 | Systolic blood pressure | Neale B | UKBB | European | 2017 | -0.0159 | 0.0026 | 1.32E-09 | 317754 | 0 | 317754 | IVNT |
| | rs460718 | chr10:115721364 | A | G | rs151599 | chr10:115724039 | G | A | Yes | 1.00 | Vascular or heart problems diagnosed by doctor: high blood pressure | Neale B | UKBB | European | 2017 | -0.0081 | 0.0012 | 2.28E-12 | 336683 | 91033 | 245650 | - |
| | rs460718 | chr10:115721364 | A | G | rs151599 | chr10:115724039 | G | A | Yes | 1.00 | Vascular or heart problems diagnosed by doctor: none of the above | Neale B | UKBB | European | 2017 | 0.0076 | 0.0012 | 1.41E-10 | 336683 | 236530 | 100153 | - |
| | rs1801253 | chr10:115805056 | C | G | rs1801253 | chr10:115805056 | C | G | No | 1.00 | Diastolic blood pressure | BPExome | NA | Mixed | 2016 | NA | NA | 2.44E-16 | 192763 | 0 | 192763 | INVT |
| | rs1801253 | chr10:115805056 | C | G | rs1801253 | chr10:115805056 | C | G | No | 1.00 | Systolic blood pressure | BPExome | NA | Mixed | 2016 | NA | NA | 1.19E-10 | 192763 | 0 | 192763 | INVT |
| | rs1801253 | chr10:115805056 | C | G | rs1801253 | chr10:115805056 | C | G | No | 1.00 | **Birth weight and gestational age** | EGGC | 23202124 | European | 2013 | NA | NA | 3.57E-09 | 26836 | - | - | - |

| | | | | | | | | | | | | | | | | | | | | | | |
|---|---|---|---|---|---|---|---|---|---|---|---|---|---|---|---|---|---|---|---|---|---|---|
| | rs1801253 | chr10:115805056 | C | G | rs1801253 | chr10:115805056 | C | G | No | 1.00 | **Birth weight** | EGGC | 23202124 | European | 2013 | -0.0410 | 0.0070 | 4.00E-09 | - | - | - | gram decrease |
| | rs1801253 | chr10:115805056 | C | G | rs1801253 | chr10:115805056 | C | G | No | 1.00 | **Birth weight** | Neale B | UKBB | European | 2017 | 0.0286 | 0.0036 | 2.86E-15 | 193063 | 0 | 193063 | IVNT |
| | rs1801253 | chr10:115805056 | C | G | rs1801253 | chr10:115805056 | C | G | No | 1.00 | Diastolic blood pressure | Neale B | UKBB | European | 2017 | 0.0242 | 0.0028 | 6.04E-18 | 317756 | 0 | 317756 | IVNT |
| | rs1801253 | chr10:115805056 | C | G | rs1801253 | chr10:115805056 | C | G | No | 1.00 | **Height** | Neale B | UKBB | European | 2017 | 0.0125 | 0.0020 | 2.17E-10 | 336474 | 0 | 336474 | IVNT |
| | rs1801253 | chr10:115805056 | C | G | rs1801253 | chr10:115805056 | C | G | No | 1.00 | Self-reported hypertension | Neale B | UKBB | European | 2017 | 0.0108 | 0.0012 | 6.95E-19 | 337159 | 87690 | 249469 | - |
| | rs1801253 | chr10:115805056 | C | G | rs1801253 | chr10:115805056 | C | G | No | 1.00 | Systolic blood pressure | Neale B | UKBB | European | 2017 | 0.0182 | 0.0028 | 9.24E-11 | 317754 | 0 | 317754 | IVNT |
| | rs1801253 | chr10:115805056 | C | G | rs1801253 | chr10:115805056 | C | G | No | 1.00 | Treatment with blood pressure medication | Neale B | UKBB | European | 2017 | 0.0096 | 0.0014 | 3.14E-11 | 180203 | 31488 | 148715 | - |
| | rs1801253 | chr10:115805056 | C | G | rs1801253 | chr10:115805056 | C | G | No | 1.00 | Vascular or heart problems diagnosed by doctor: high blood pressure | Neale B | UKBB | European | 2017 | 0.0117 | 0.0012 | 1.70E-21 | 336683 | 91033 | 245650 | - |
| | rs1801253 | chr10:115805056 | C | G | rs1801253 | chr10:115805056 | C | G | No | 1.00 | Vascular or heart problems diagnosed by doctor: none of the above | Neale B | UKBB | European | 2017 | -0.0115 | 0.0013 | 1.35E-19 | 336683 | 236530 | 100153 | - |
| | rs1801253 | chr10:115805056 | C | G | rs2484294 | chr10:115792062 | A | G | Yes | 0.97 | **Birth weight** | Neale B | UKBB | European | 2017 | 0.0280 | 0.0036 | 8.12E-15 | 193063 | 0 | 193063 | IVNT |
| | rs1801253 | chr10:115805056 | C | G | rs2484294 | chr10:115792062 | A | G | Yes | 0.97 | Diastolic blood pressure | Neale B | UKBB | European | 2017 | 0.0241 | 0.0028 | 7.73E-18 | 317756 | 0 | 317756 | IVNT |
| | rs1801253 | chr10:115805056 | C | G | rs2484294 | chr10:115792062 | A | G | Yes | 0.97 | **Height** | Neale B | UKBB | European | 2017 | 0.0125 | 0.0020 | 1.79E-10 | 336474 | 0 | 336474 | IVNT |
| | rs1801253 | chr10:115805056 | C | G | rs2484294 | chr10:115792062 | A | G | Yes | 0.97 | Self-reported hypertension | Neale B | UKBB | European | 2017 | 0.0107 | 0.0012 | 8.45E-19 | 337159 | 87690 | 249469 | - |
| | rs1801253 | chr10:115805056 | C | G | rs2484294 | chr10:115792062 | A | G | Yes | 0.97 | Systolic blood pressure | Neale B | UKBB | European | 2017 | 0.0179 | 0.0028 | 1.70E-10 | 317754 | 0 | 317754 | IVNT |
| | rs1801253 | chr10:115805056 | C | G | rs2484294 | chr10:115792062 | A | G | Yes | 0.97 | Treatment with blood pressure medication | Neale B | UKBB | European | 2017 | 0.0096 | 0.0014 | 2.43E-11 | 180203 | 31488 | 148715 | - |
| | rs1801253 | chr10:115805056 | C | G | rs2484294 | chr10:115792062 | A | G | Yes | 0.97 | Vascular or heart problems diagnosed by doctor: high blood pressure | Neale B | UKBB | European | 2017 | 0.0117 | 0.0012 | 2.37E-21 | 336683 | 91033 | 245650 | - |
| | rs1801253 | chr10:115805056 | C | G | rs2484294 | chr10:115792062 | A | G | Yes | 0.97 | Vascular or heart problems diagnosed by doctor: none of the above | Neale B | UKBB | European | 2017 | -0.0114 | 0.0013 | 2.12E-19 | 336683 | 236530 | 100153 | - |
| | rs1801253 | chr10:115805056 | C | G | rs740746 | chr10:115792787 | A | G | Yes | 0.97 | **Birth weight** | Neale B | UKBB | European | 2017 | 0.0278 | 0.0036 | 1.03E-14 | 193063 | 0 | 193063 | IVNT |
| | rs1801253 | chr10:115805056 | C | G | rs740746 | chr10:115792787 | A | G | Yes | 0.97 | Diastolic blood pressure | Neale B | UKBB | European | 2017 | 0.0240 | 0.0028 | 7.93E-18 | 317756 | 0 | 317756 | IVNT |
| | rs1801253 | chr10:115805056 | C | G | rs740746 | chr10:115792787 | A | G | Yes | 0.97 | **Height** | Neale B | UKBB | European | 2017 | 0.0125 | 0.0020 | 1.96E-10 | 336474 | 0 | 336474 | IVNT |
| | rs1801253 | chr10:115805056 | C | G | rs740746 | chr10:115792787 | A | G | Yes | 0.97 | Self-reported hypertension | Neale B | UKBB | European | 2017 | 0.0108 | 0.0012 | 6.26E-19 | 337159 | 87690 | 249469 | - |
| | rs1801253 | chr10:115805056 | C | G | rs740746 | chr10:115792787 | A | G | Yes | 0.97 | Systolic blood pressure | Neale B | UKBB | European | 2017 | 0.0180 | 0.0028 | 1.28E-10 | 317754 | 0 | 317754 | IVNT |
| | rs1801253 | chr10:115805056 | C | G | rs740746 | chr10:115792787 | A | G | Yes | 0.97 | Treatment with blood pressure medication | Neale B | UKBB | European | 2017 | 0.0096 | 0.0014 | 2.07E-11 | 180203 | 31488 | 148715 | - |
| | rs1801253 | chr10:115805056 | C | G | rs740746 | chr10:115792787 | A | G | Yes | 0.97 | Vascular or heart problems diagnosed by doctor: high blood pressure | Neale B | UKBB | European | 2017 | 0.0117 | 0.0012 | 1.66E-21 | 336683 | 91033 | 245650 | - |
| | rs1801253 | chr10:115805056 | C | G | rs740746 | chr10:115792787 | A | G | Yes | 0.97 | Vascular or heart problems diagnosed by doctor: none of the above | Neale B | UKBB | European | 2017 | -0.0114 | 0.0013 | 1.88E-19 | 336683 | 236530 | 100153 | - |
| | rs1801253 | chr10:115805056 | C | G | rs2773469 | chr10:115798895 | G | A | Yes | 0.97 | **Birth weight** | Neale B | UKBB | European | 2017 | 0.0281 | 0.0036 | 6.15E-15 | 193063 | 0 | 193063 | IVNT |
| | rs1801253 | chr10:115805056 | C | G | rs2773469 | chr10:115798895 | G | A | Yes | 0.97 | Diastolic blood pressure | Neale B | UKBB | European | 2017 | 0.0240 | 0.0028 | 9.55E-18 | 317756 | 0 | 317756 | IVNT |
| | rs1801253 | chr10:115805056 | C | G | rs2773469 | chr10:115798895 | G | A | Yes | 0.97 | **Height** | Neale B | UKBB | European | 2017 | 0.0125 | 0.0020 | 1.93E-10 | 336474 | 0 | 336474 | IVNT |
| | rs1801253 | chr10:115805056 | C | G | rs2773469 | chr10:115798895 | G | A | Yes | 0.97 | Self-reported hypertension | Neale B | UKBB | European | 2017 | 0.0107 | 0.0012 | 9.39E-19 | 337159 | 87690 | 249469 | - |
| | rs1801253 | chr10:115805056 | C | G | rs2773469 | chr10:115798895 | G | A | Yes | 0.97 | Systolic blood pressure | Neale B | UKBB | European | 2017 | 0.0178 | 0.0028 | 1.99E-10 | 317754 | 0 | 317754 | IVNT |
| | rs1801253 | chr10:115805056 | C | G | rs2773469 | chr10:115798895 | G | A | Yes | 0.97 | Treatment with blood pressure medication | Neale B | UKBB | European | 2017 | 0.0096 | 0.0014 | 2.56E-11 | 180203 | 31488 | 148715 | - |
| | rs1801253 | chr10:115805056 | C | G | rs2773469 | chr10:115798895 | G | A | Yes | 0.97 | Vascular or heart problems diagnosed by doctor: high blood pressure | Neale B | UKBB | European | 2017 | 0.0116 | 0.0012 | 2.59E-21 | 336683 | 91033 | 245650 | - |
| | rs1801253 | chr10:115805056 | C | G | rs2773469 | chr10:115798895 | G | A | Yes | 0.97 | Vascular or heart problems diagnosed by doctor: none of the above | Neale B | UKBB | European | 2017 | -0.0113 | 0.0013 | 2.36E-19 | 336683 | 236530 | 100153 | - |

| | | | | | | | | | | | | | | | | | | | | | | |
|---|---|---|---|---|---|---|---|---|---|---|---|---|---|---|---|---|---|---|---|---|---|---|
| | rs1801253 | chr10:115805056 | C | G | rs7076938 | chr10:115789375 | T | C | Yes | 0.96 | Diastolic blood pressure | BPExome | NA | Mixed | 2016 | NA | NA | 4.91E-17 | 192763 | 0 | 192763 | INVT |
| | rs1801253 | chr10:115805056 | C | G | rs7076938 | chr10:115789375 | T | C | Yes | 0.96 | Systolic blood pressure | BPExome | NA | Mixed | 2016 | NA | NA | 3.76E-12 | 192763 | 0 | 192763 | INVT |
| | rs1801253 | chr10:115805056 | C | G | rs7076938 | chr10:115789375 | T | C | Yes | 0.96 | **Birth weight** | Horikoshi M | 27680694 | Mixed | 2016 | 0.0349 | 0.0040 | 5.00E-18 | - | - | - | unit increase |
| | rs1801253 | chr10:115805056 | C | G | rs7076938 | chr10:115789375 | T | C | Yes | 0.96 | **Birth weight** | Neale B | UKBB | European | 2017 | 0.0280 | 0.0036 | 7.82E-15 | 193063 | 0 | 193063 | IVNT |
| | rs1801253 | chr10:115805056 | C | G | rs7076938 | chr10:115789375 | T | C | Yes | 0.96 | Diastolic blood pressure | Neale B | UKBB | European | 2017 | 0.0241 | 0.0028 | 6.75E-18 | 317756 | 0 | 317756 | IVNT |
| | rs1801253 | chr10:115805056 | C | G | rs7076938 | chr10:115789375 | T | C | Yes | 0.96 | **Height** | Neale B | UKBB | European | 2017 | 0.0125 | 0.0020 | 1.74E-10 | 336474 | 0 | 336474 | IVNT |
| | rs1801253 | chr10:115805056 | C | G | rs7076938 | chr10:115789375 | T | C | Yes | 0.96 | Self-reported hypertension | Neale B | UKBB | European | 2017 | 0.0107 | 0.0012 | 8.24E-19 | 337159 | 87690 | 249469 | - |
| | rs1801253 | chr10:115805056 | C | G | rs7076938 | chr10:115789375 | T | C | Yes | 0.96 | Systolic blood pressure | Neale B | UKBB | European | 2017 | 0.0180 | 0.0028 | 1.28E-10 | 317754 | 0 | 317754 | IVNT |
| | rs1801253 | chr10:115805056 | C | G | rs7076938 | chr10:115789375 | T | C | Yes | 0.96 | Treatment with blood pressure medication | Neale B | UKBB | European | 2017 | 0.0096 | 0.0014 | 2.81E-11 | 180203 | 31488 | 148715 | - |
| | rs1801253 | chr10:115805056 | C | G | rs7076938 | chr10:115789375 | T | C | Yes | 0.96 | Vascular or heart problems diagnosed by doctor: high blood pressure | Neale B | UKBB | European | 2017 | 0.0117 | 0.0012 | 2.34E-21 | 336683 | 91033 | 245650 | - |
| | rs1801253 | chr10:115805056 | C | G | rs7076938 | chr10:115789375 | T | C | Yes | 0.96 | Vascular or heart problems diagnosed by doctor: none of the above | Neale B | UKBB | European | 2017 | -0.0113 | 0.0013 | 2.34E-19 | 336683 | 236530 | 100153 | - |
| | rs4359161 | chr10:115826508 | A | G | rs4918889 | chr10:115830718 | G | C | Yes | 0.90 | Diastolic blood pressure | Neale B | UKBB | European | 2017 | -0.0197 | 0.0033 | 1.46E-09 | 317756 | 0 | 317756 | IVNT |
| | rs4359161 | chr10:115826508 | A | G | rs68122733 | chr10:115831533 | G | A | Yes | 0.90 | Diastolic blood pressure | Neale B | UKBB | European | 2017 | -0.0197 | 0.0033 | 1.49E-09 | 317756 | 0 | 317756 | IVNT |
| | rs3821843 | chr3:53558012 | A | G | rs3821843 | chr3:53558012 | A | G | No | 1.00 | **Impedance of whole body** | Neale B | UKBB | European | 2017 | -0.0110 | 0.0020 | 4.21E-08 | 331284 | 0 | 331284 | IVNT |
| | rs3821843 | chr3:53558012 | A | G | rs3821843 | chr3:53558012 | A | G | No | 1.00 | Self-reported hypertension | Neale B | UKBB | European | 2017 | 0.0095 | 0.0012 | 2.46E-16 | 337159 | 87690 | 249469 | - |
| | rs3821843 | chr3:53558012 | A | G | rs3821843 | chr3:53558012 | A | G | No | 1.00 | Systolic blood pressure | Neale B | UKBB | European | 2017 | 0.0155 | 0.0027 | 7.33E-09 | 317754 | 0 | 317754 | IVNT |
| | rs3821843 | chr3:53558012 | A | G | rs3821843 | chr3:53558012 | A | G | No | 1.00 | Treatment with amlodipine | Neale B | UKBB | European | 2017 | 0.0029 | 0.0005 | 3.57E-08 | 337159 | 13693 | 323466 | - |
| | rs3821843 | chr3:53558012 | A | G | rs3821843 | chr3:53558012 | A | G | No | 1.00 | Treatment with bendroflumethiazide | Neale B | UKBB | European | 2017 | 0.0036 | 0.0006 | 4.21E-09 | 337159 | 19084 | 318075 | - |
| | rs3821843 | chr3:53558012 | A | G | rs3821843 | chr3:53558012 | A | G | No | 1.00 | Vascular or heart problems diagnosed by doctor: high blood pressure | Neale B | UKBB | European | 2017 | 0.0095 | 0.0012 | 8.66E-16 | 336683 | 91033 | 245650 | - |
| | rs3821843 | chr3:53558012 | A | G | rs3821843 | chr3:53558012 | A | G | No | 1.00 | Vascular or heart problems diagnosed by doctor: none of the above | Neale B | UKBB | European | 2017 | -0.0094 | 0.0012 | 8.32E-15 | 336683 | 236530 | 100153 | - |
| | rs7340705 | chr3:53734443 | C | T | rs7340705 | chr3:53734443 | C | T | No | 1.00 | Self-reported hypertension | Neale B | UKBB | European | 2017 | 0.0071 | 0.0011 | 4.93E-10 | 337159 | 87690 | 249469 | - |
| | rs7340705 | chr3:53734443 | C | T | rs7340705 | chr3:53734443 | C | T | No | 1.00 | Vascular or heart problems diagnosed by doctor: high blood pressure | Neale B | UKBB | European | 2017 | 0.0073 | 0.0012 | 4.43E-10 | 336683 | 91033 | 245650 | - |
| CCB | rs7340705 | chr3:53734443 | C | T | rs7340705 | chr3:53734443 | C | T | No | 1.00 | Vascular or heart problems diagnosed by doctor: none of the above | Neale B | UKBB | European | 2017 | -0.0068 | 0.0012 | 1.40E-08 | 336683 | 236530 | 100153 | - |
| | rs7340705 | chr3:53734443 | C | T | rs2612016 | chr3:53734453 | C | G | Yes | 1.00 | Self-reported hypertension | Neale B | UKBB | European | 2017 | 0.0072 | 0.0011 | 4.57E-10 | 337159 | 87690 | 249469 | - |
| | rs7340705 | chr3:53734443 | C | T | rs2612016 | chr3:53734453 | C | G | Yes | 1.00 | Vascular or heart problems diagnosed by doctor: high blood pressure | Neale B | UKBB | European | 2017 | 0.0073 | 0.0012 | 3.90E-10 | 336683 | 91033 | 245650 | - |
| | rs7340705 | chr3:53734443 | C | T | rs2612016 | chr3:53734453 | C | G | Yes | 1.00 | Vascular or heart problems diagnosed by doctor: none of the above | Neale B | UKBB | European | 2017 | -0.0068 | 0.0012 | 1.21E-08 | 336683 | 236530 | 100153 | - |
| | rs7340705 | chr3:53734443 | C | T | rs6445597 | chr3:53734531 | A | G | Yes | 1.00 | Self-reported hypertension | Neale B | UKBB | European | 2017 | 0.0071 | 0.0011 | 5.24E-10 | 337159 | 87690 | 249469 | - |
| | rs7340705 | chr3:53734443 | C | T | rs6445597 | chr3:53734531 | A | G | Yes | 1.00 | Vascular or heart problems diagnosed by doctor: high blood pressure | Neale B | UKBB | European | 2017 | 0.0072 | 0.0012 | 4.74E-10 | 336683 | 91033 | 245650 | - |
| | rs7340705 | chr3:53734443 | C | T | rs6445597 | chr3:53734531 | A | G | Yes | 1.00 | Vascular or heart problems diagnosed by doctor: none of the above | Neale B | UKBB | European | 2017 | -0.0068 | 0.0012 | 1.37E-08 | 336683 | 236530 | 100153 | - |
| | rs7340705 | chr3:53734443 | C | T | rs2680663 | chr3:53735299 | G | A | Yes | 1.00 | Self-reported hypertension | Neale B | UKBB | European | 2017 | 0.0072 | 0.0011 | 4.26E-10 | 337159 | 87690 | 249469 | - |
| | rs7340705 | chr3:53734443 | C | T | rs2680663 | chr3:53735299 | G | A | Yes | 1.00 | Vascular or heart problems diagnosed by doctor: high blood pressure | Neale B | UKBB | European | 2017 | 0.0073 | 0.0012 | 4.05E-10 | 336683 | 91033 | 245650 | - |

| | | | | | | | | | | | | | | | | | | | | | |
|---|---|---|---|---|---|---|---|---|---|---|---|---|---|---|---|---|---|---|---|---|---|
| | rs7340705 | chr3:53734443 | C | T | rs2680663 | chr3:53735299 | G | A | Yes | 1.00 | Vascular or heart problems diagnosed by doctor: none of the above | Neale B | UKBB | European | 2017 | -0.0068 | 0.0012 | 1.11E-08 | 336683 | 236530 | 100153 | - |
| | rs7340705 | chr3:53734443 | C | T | rs9841978 | chr3:53730735 | A | G | Yes | 0.97 | Self-reported hypertension | Neale B | UKBB | European | 2017 | 0.0070 | 0.0011 | 9.61E-10 | 337159 | 87690 | 249469 | - |
| | rs7340705 | chr3:53734443 | C | T | rs9841978 | chr3:53730735 | A | G | Yes | 0.97 | Vascular or heart problems diagnosed by doctor: high blood pressure | Neale B | UKBB | European | 2017 | 0.0071 | 0.0012 | 8.39E-10 | 336683 | 91033 | 245650 | - |
| | rs7340705 | chr3:53734443 | C | T | rs9841978 | chr3:53730735 | A | G | Yes | 0.97 | Vascular or heart problems diagnosed by doctor: none of the above | Neale B | UKBB | European | 2017 | -0.0067 | 0.0012 | 1.97E-08 | 336683 | 236530 | 100153 | - |
| | rs1888693 | chr10:18440444 | G | A | rs1888693 | chr10:18440444 | G | A | No | 1.00 | Self-reported hypertension | Neale B | UKBB | European | 2017 | -0.0074 | 0.0011 | 4.21E-11 | 337159 | 87690 | 249469 | - |
| | rs1888693 | chr10:18440444 | G | A | rs1888693 | chr10:18440444 | G | A | No | 1.00 | Systolic blood pressure | Neale B | UKBB | European | 2017 | -0.0159 | 0.0026 | 9.98E-10 | 317754 | 0 | 317754 | IVNT |
| | rs1888693 | chr10:18440444 | G | A | rs1888693 | chr10:18440444 | G | A | No | 1.00 | Treatment with blood pressure medication | Neale B | UKBB | European | 2017 | -0.0081 | 0.0013 | 1.43E-09 | 180203 | 31488 | 148715 | - |
| | rs1888693 | chr10:18440444 | G | A | rs1888693 | chr10:18440444 | G | A | No | 1.00 | Vascular or heart problems diagnosed by doctor: high blood pressure | Neale B | UKBB | European | 2017 | -0.0078 | 0.0011 | 9.81E-12 | 336683 | 91033 | 245650 | - |
| | rs1888693 | chr10:18440444 | G | A | rs1888693 | chr10:18440444 | G | A | No | 1.00 | Vascular or heart problems diagnosed by doctor: none of the above | Neale B | UKBB | European | 2017 | 0.0074 | 0.0012 | 2.49E-10 | 336683 | 236530 | 100153 | - |
| | rs16916914 | chr10:18457722 | C | T | rs16916914 | chr10:18457722 | C | T | No | 1.00 | Diastolic blood pressure | Neale B | UKBB | European | 2017 | 0.0372 | 0.0068 | 3.51E-08 | 317756 | 0 | 317756 | IVNT |
| | rs16916914 | chr10:18457722 | C | T | rs16916914 | chr10:18457722 | C | T | No | 1.00 | Systolic blood pressure | Neale B | UKBB | European | 2017 | 0.0376 | 0.0067 | 2.53E-08 | 317754 | 0 | 317754 | IVNT |
| | rs16916914 | chr10:18457722 | C | T | rs138094231 | chr10:18429624 | A | C | Yes | 0.97 | Diastolic blood pressure | Neale B | UKBB | European | 2017 | 0.0381 | 0.0068 | 2.07E-08 | 317756 | 0 | 317756 | IVNT |
| | rs16916914 | chr10:18457722 | C | T | rs138094231 | chr10:18429624 | A | C | Yes | 0.97 | Systolic blood pressure | Neale B | UKBB | European | 2017 | 0.0382 | 0.0068 | 1.83E-08 | 317754 | 0 | 317754 | IVNT |
| | rs16916914 | chr10:18457722 | C | T | rs1929423 | chr10:18445684 | A | G | Yes | 0.97 | Systolic blood pressure | Neale B | UKBB | European | 2017 | 0.0382 | 0.0067 | 1.46E-08 | 317754 | 0 | 317754 | IVNT |
| | rs16916914 | chr10:18457722 | C | T | rs144009622 | chr10:18461061 | T | G | Yes | 0.97 | Diastolic blood pressure | Neale B | UKBB | European | 2017 | 0.0370 | 0.0068 | 4.30E-08 | 317756 | 0 | 317756 | IVNT |
| | rs16916914 | chr10:18457722 | C | T | rs144009622 | chr10:18461061 | T | G | Yes | 0.97 | Systolic blood pressure | Neale B | UKBB | European | 2017 | 0.0374 | 0.0067 | 2.98E-08 | 317754 | 0 | 317754 | IVNT |
| | rs7076319 | chr10:18459450 | A | G | rs7076319 | chr10:18459450 | A | G | No | 1.00 | Systolic blood pressure | Neale B | UKBB | European | 2017 | -0.0161 | 0.0028 | 9.01E-09 | 317754 | 0 | 317754 | IVNT |
| | rs7076319 | chr10:18459450 | A | G | rs7076319 | chr10:18459450 | A | G | No | 1.00 | Vascular or heart problems diagnosed by doctor: high blood pressure | Neale B | UKBB | European | 2017 | -0.0070 | 0.0012 | 1.40E-08 | 336683 | 91033 | 245650 | - |
| | rs7076319 | chr10:18459450 | A | G | rs11594555 | chr10:18446207 | T | C | Yes | 0.87 | Self-reported hypertension | Neale B | UKBB | European | 2017 | -0.0078 | 0.0012 | 1.81E-10 | 337159 | 87690 | 249469 | - |
| | rs7076319 | chr10:18459450 | A | G | rs11594555 | chr10:18446207 | T | C | Yes | 0.87 | Systolic blood pressure | Neale B | UKBB | European | 2017 | -0.0170 | 0.0028 | 1.88E-09 | 317754 | 0 | 317754 | IVNT |
| | rs7076319 | chr10:18459450 | A | G | rs11594555 | chr10:18446207 | T | C | Yes | 0.87 | Vascular or heart problems diagnosed by doctor: high blood pressure | Neale B | UKBB | European | 2017 | -0.0084 | 0.0012 | 1.59E-11 | 336683 | 91033 | 245650 | - |
| | rs7076319 | chr10:18459450 | A | G | rs11594555 | chr10:18446207 | T | C | Yes | 0.87 | Vascular or heart problems diagnosed by doctor: none of the above | Neale B | UKBB | European | 2017 | 0.0077 | 0.0013 | 1.89E-09 | 336683 | 236530 | 100153 | - |
| | rs1779209 | chr10:18514561 | T | C | rs1779209 | chr10:18514561 | T | C | No | 1.00 | Vascular or heart problems diagnosed by doctor: high blood pressure | Neale B | UKBB | European | 2017 | 0.0066 | 0.0012 | 3.96E-08 | 336683 | 91033 | 245650 | - |
| | rs10828399 | chr10:18553968 | A | G | rs10828399 | chr10:18553968 | A | G | No | 1.00 | **Body mass index** | Akiyama M | 28892062 | East Asian | 2017 | 0.0215 | 0.0037 | 4.85E-09 | 173430 | 0 | 173430 | IVNT |
| | rs10828399 | chr10:18553968 | A | G | rs10764373 | chr10:18553665 | T | G | Yes | 0.99 | **Body mass index** | Akiyama M | 28892062 | East Asian | 2017 | 0.0215 | 0.0037 | 4.59E-09 | 173430 | 0 | 173430 | IVNT |
| | rs10828399 | chr10:18553968 | A | G | rs2357928 | chr10:18549641 | A | G | Yes | 0.94 | **Body mass index** | Akiyama M | 28892062 | East Asian | 2017 | 0.0198 | 0.0036 | 4.92E-08 | 173430 | 0 | 173430 | IVNT |
| | rs12780039 | chr10:18678987 | C | G | rs79586955 | chr10:18674765 | T | A | Yes | 0.92 | **Pulse rate** | Neale B | UKBB | European | 2017 | -0.0215 | 0.0038 | 2.24E-08 | 317756 | 0 | 317756 | IVNT |
| | rs7923191 | chr10:18727901 | A | G | rs7923191 | chr10:18727901 | A | G | No | 1.00 | Systolic blood pressure | Neale B | UKBB | European | 2017 | -0.0183 | 0.0031 | 2.84E-09 | 317754 | 0 | 317754 | IVNT |
| | rs7923191 | chr10:18727901 | A | G | rs7923191 | chr10:18727901 | A | G | No | 1.00 | Treatment with blood pressure medication | Neale B | UKBB | European | 2017 | -0.0094 | 0.0016 | 2.77E-09 | 180203 | 31488 | 148715 | - |
| | rs7923191 | chr10:18727901 | A | G | rs7923191 | chr10:18727901 | A | G | No | 1.00 | Vascular or heart problems diagnosed by doctor: high blood pressure | Neale B | UKBB | European | 2017 | -0.0077 | 0.0014 | 1.11E-08 | 336683 | 91033 | 245650 | - |
| | rs7923191 | chr10:18727901 | A | G | rs7923191 | chr10:18727901 | A | G | No | 1.00 | Vascular or heart problems diagnosed by doctor: none of the above | Neale B | UKBB | European | 2017 | 0.0086 | 0.0014 | 6.37E-10 | 336683 | 236530 | 100153 | - |

| | | | | | | | | | | | | | | | | | | | | | | |
|---|---|---|---|---|---|---|---|---|---|---|---|---|---|---|---|---|---|---|---|---|---|---|
| | rs12258967 | chr10:18727959 | C | G | rs12258967 | chr10:18727959 | C | G | No | 1.00 | Mean arterial pressure | Wain LV | 21909110 | European | 2011 | NA | NA | 2.05E-16 | 74064 | - | - | - |
| | rs12258967 | chr10:18727959 | C | G | rs12258967 | chr10:18727959 | C | G | No | 1.00 | Blood pressure | Wain LV | 21909110 | European | 2011 | -0.4310 | 0.0524 | 2.00E-16 | - | - | - | mmHg decrease |
| | rs12258967 | chr10:18727959 | C | G | rs12258967 | chr10:18727959 | C | G | No | 1.00 | Diastolic blood pressure | Neale B | UKBB | European | 2017 | 0.0256 | 0.0027 | 2.05E-21 | 317756 | 0 | 317756 | IVNT |
| | rs12258967 | chr10:18727959 | C | G | rs12258967 | chr10:18727959 | C | G | No | 1.00 | Self-reported hypertension | Neale B | UKBB | European | 2017 | 0.0105 | 0.0012 | 1.97E-19 | 337159 | 87690 | 249469 | - |
| | rs12258967 | chr10:18727959 | C | G | rs12258967 | chr10:18727959 | C | G | No | 1.00 | Systolic blood pressure | Neale B | UKBB | European | 2017 | 0.0314 | 0.0027 | 1.73E-31 | 317754 | 0 | 317754 | IVNT |
| | rs12258967 | chr10:18727959 | C | G | rs12258967 | chr10:18727959 | C | G | No | 1.00 | Treatment with blood pressure medication | Neale B | UKBB | European | 2017 | 0.0097 | 0.0014 | 2.02E-12 | 180203 | 31488 | 148715 | - |
| | rs12258967 | chr10:18727959 | C | G | rs12258967 | chr10:18727959 | C | G | No | 1.00 | Vascular or heart problems diagnosed by doctor: high blood pressure | Neale B | UKBB | European | 2017 | 0.0110 | 0.0012 | 1.74E-20 | 336683 | 91033 | 245650 | - |
| | rs12258967 | chr10:18727959 | C | G | rs12258967 | chr10:18727959 | C | G | No | 1.00 | Vascular or heart problems diagnosed by doctor: none of the above | Neale B | UKBB | European | 2017 | -0.0113 | 0.0012 | 1.01E-20 | 336683 | 236530 | 100153 | - |
| | rs12258967 | chr10:18727959 | C | G | rs12258967 | chr10:18727959 | C | G | No | 1.00 | Blood pressure | Wain LV | 21909110 | European | 2011 | NA | NA | 2.00E-16 | - | - | - | - |
| | rs72786098 | chr10:18729855 | A | G | rs72786098 | chr10:18729855 | A | G | No | 1.00 | **Small vessel stroke** | Cheng YC | 26732560 | Mixed | 2016 | 1.2730 | 0.2268 | 2.00E-08 | - | - | - | log OR |
| | rs714277 | chr12:2514270 | C | T | rs714277 | chr12:2514270 | C | T | No | 1.00 | **Hematocrit** | Astle W | 27863252 | European | 2016 | -0.0245 | 0.0039 | 3.38E-10 | 173480 | 0 | 173480 | - |
| | rs714277 | chr12:2514270 | C | T | rs714277 | chr12:2514270 | C | T | No | 1.00 | **Red blood cell count** | Astle W | 27863252 | European | 2016 | -0.0242 | 0.0039 | 7.96E-10 | 173480 | 0 | 173480 | - |
| | rs714277 | chr12:2514270 | C | T | rs714277 | chr12:2514270 | C | T | No | 1.00 | **Schizophrenia** | PGC | 25056061 | Mixed | 2014 | 0.0686 | 0.0118 | 6.64E-09 | 82315 | 35476 | 46839 | log OR |
| | rs714277 | chr12:2514270 | C | T | rs12823424 | chr12:2514112 | A | G | Yes | 1.00 | **Hematocrit** | Astle W | 27863252 | European | 2016 | -0.0246 | 0.0039 | 2.85E-10 | 173480 | 0 | 173480 | - |
| | rs714277 | chr12:2514270 | C | T | rs12823424 | chr12:2514112 | A | G | Yes | 1.00 | **Red blood cell count** | Astle W | 27863252 | European | 2016 | -0.0243 | 0.0039 | 5.88E-10 | 173480 | 0 | 173480 | - |
| | rs714277 | chr12:2514270 | C | T | rs12823424 | chr12:2514112 | A | G | Yes | 1.00 | **Schizophrenia** | Goes FS | 26198764 | European | 2015 | 0.0677 | 0.0116 | 5.00E-09 | - | - | - | log OR |
| | rs714277 | chr12:2514270 | C | T | rs12823424 | chr12:2514112 | A | G | Yes | 1.00 | **Schizophrenia** | PGC | 25056061 | Mixed | 2014 | 0.0690 | 0.0118 | 5.47E-09 | 82315 | 35476 | 46839 | log OR |
| | rs714277 | chr12:2514270 | C | T | rs2239063 | chr12:2511831 | A | C | Yes | 1.00 | **Hematocrit** | Astle W | 27863252 | European | 2016 | -0.0245 | 0.0039 | 3.04E-10 | 173480 | 0 | 173480 | - |
| | rs714277 | chr12:2514270 | C | T | rs2239063 | chr12:2511831 | A | C | Yes | 1.00 | **Red blood cell count** | Astle W | 27863252 | European | 2016 | -0.0242 | 0.0039 | 6.62E-10 | 173480 | 0 | 173480 | - |
| | rs714277 | chr12:2514270 | C | T | rs2239063 | chr12:2511831 | A | C | Yes | 1.00 | **Schizophrenia** | PGC | 25056061 | Mixed | 2014 | 0.0690 | 0.0118 | 5.39E-09 | 82315 | 35476 | 46839 | log OR |
| | rs714277 | chr12:2514270 | C | T | rs758117 | chr12:2513309 | C | T | Yes | 1.00 | **Hematocrit** | Astle W | 27863252 | European | 2016 | -0.0245 | 0.0039 | 3.28E-10 | 173480 | 0 | 173480 | - |
| | rs714277 | chr12:2514270 | C | T | rs758117 | chr12:2513309 | C | T | Yes | 1.00 | **Red blood cell count** | Astle W | 27863252 | European | 2016 | -0.0243 | 0.0039 | 6.17E-10 | 173480 | 0 | 173480 | - |
| | rs714277 | chr12:2514270 | C | T | rs758117 | chr12:2513309 | C | T | Yes | 1.00 | **Schizophrenia** | Li Z | 28991256 | Mixed | 2017 | 0.0608 | 0.0108 | 2.00E-08 | - | - | - | log OR |
| | rs714277 | chr12:2514270 | C | T | rs758117 | chr12:2513309 | C | T | Yes | 1.00 | **Schizophrenia** | PGC | 25056061 | Mixed | 2014 | 0.0686 | 0.0118 | 6.46E-09 | 82315 | 35476 | 46839 | log OR |
| | rs714277 | chr12:2514270 | C | T | rs10491964 | chr12:2500431 | G | A | Yes | 0.92 | **Hematocrit** | Astle W | 27863252 | European | 2016 | -0.0259 | 0.0040 | 7.19E-11 | 173480 | 0 | 173480 | - |
| | rs714277 | chr12:2514270 | C | T | rs10491964 | chr12:2500431 | G | A | Yes | 0.92 | **Hemoglobin concentration** | Astle W | 27863252 | European | 2016 | -0.0223 | 0.0040 | 2.21E-08 | 173480 | 0 | 173480 | - |
| | rs714277 | chr12:2514270 | C | T | rs10491964 | chr12:2500431 | G | A | Yes | 0.92 | **Red blood cell count** | Astle W | 27863252 | European | 2016 | -0.0250 | 0.0040 | 3.79E-10 | 173480 | 0 | 173480 | - |
| | rs714277 | chr12:2514270 | C | T | rs10491964 | chr12:2500431 | G | A | Yes | 0.92 | **Schizophrenia** | PGC | 25056061 | Mixed | 2014 | 0.0656 | 0.0118 | 2.79E-08 | 82315 | 35476 | 46839 | log OR |

| Supplementary Table 10. Phenome-wide association study (PheWAS) results for the angiotensin-converting-enzyme inhibitor (ACEI) instrument single-nucleotide polymorphism (rs4291). | | | | | | | | | | |
|---|---|---|---|---|---|---|---|---|---|---|
| Phecode | Trait | Category | OR | Low 95% CI | Upp 95% CI | P value | Total sample size | Cases | Controls | FDR |
| 401.1 | Essential hypertension | Circulatory System | 0.96 | 0.95 | 0.97 | 3.76E-11 | 328000 | 79235 | 248765 | TRUE |
| 401 | Hypertension | Circulatory System | 1.04 | 0.95 | 0.97 | 4.62E-11 | 328256 | 79491 | 248765 | TRUE |
| 459.9 | Circulatory disease NEC | Circulatory System | 1.02 | 0.97 | 0.99 | 6.96E-06 | 327945 | 133749 | 194196 | TRUE |
| 697 | Sarcoidosis | Dermatologic | 1.21 | 0.73 | 0.93 | 1.78E-03 | 322326 | 560 | 321766 | FALSE |
| 250.7 | Diabetic retinopathy | Endocrine/Metabolic | 1.13 | 0.82 | 0.96 | 2.02E-03 | 317249 | 1380 | 315869 | FALSE |
| 250.2 | Type 2 diabetes | Endocrine/Metabolic | 1.03 | 0.95 | 0.99 | 2.59E-03 | 326620 | 19113 | 307507 | FALSE |
| 90 | Sexually transmitted infections (not HIV or hepatitis) | Infectious Diseases | 1.28 | 0.66 | 0.92 | 3.06E-03 | 328256 | 298 | 327958 | FALSE |
| 250.23 | Type 2 diabetes with ophthalmic manifestations | Endocrine/Metabolic | 1.12 | 0.82 | 0.96 | 3.08E-03 | 308851 | 1344 | 307507 | FALSE |
| 250.22 | Type 2 diabetes with renal manifestations | Endocrine/Metabolic | 1.32 | 0.62 | 0.92 | 4.77E-03 | 307715 | 208 | 307507 | FALSE |
| 136 | Other infectious and parasitic diseases | Infectious Diseases | 1.22 | 0.72 | 0.94 | 0.01 | 327999 | 428 | 327571 | FALSE |
| 250 | Diabetes mellitus | Endocrine/Metabolic | 1.03 | 0.95 | 0.99 | 0.01 | 327747 | 20240 | 307507 | FALSE |
| 585.31 | Renal dialysis | Genitourinary | 0.83 | 1.05 | 1.36 | 0.01 | 314620 | 524 | 314096 | FALSE |
| 153.3 | Malignant neoplasm of rectum, rectosigmoid junction, and anus | Neoplasms | 1.09 | 0.86 | 0.98 | 0.01 | 303414 | 2117 | 301297 | FALSE |
| 604 | Disorders of penis | Genitourinary | 1.16 | 0.78 | 0.96 | 0.01 | 308658 | 735 | 307923 | FALSE |
| 418.1 | Precordial pain | Circulatory System | 0.94 | 1.02 | 1.12 | 0.01 | 299523 | 3686 | 295837 | FALSE |
| 276.12 | Hyposmolality and/or hyponatremia | Endocrine/Metabolic | 0.91 | 1.02 | 1.17 | 0.01 | 322513 | 1852 | 320661 | FALSE |
| 361 | Retinal detachments and defects | Sense Organs | 0.92 | 1.02 | 1.16 | 0.01 | 319028 | 2229 | 316799 | FALSE |
| 250.24 | Type 2 diabetes with neurological manifestations | Endocrine/Metabolic | 1.16 | 0.77 | 0.96 | 0.01 | 308119 | 612 | 307507 | FALSE |
| 357 | Inflammatory and toxic neuropathy | Neurological | 1.10 | 0.84 | 0.98 | 0.01 | 327392 | 1423 | 325969 | FALSE |
| 647.1 | Infections of genitourinary tract during pregnancy | Pregnancy Complications | 0.79 | 1.05 | 1.51 | 0.01 | 328145 | 284 | 327861 | FALSE |
| 724.1 | Disorders of sacrum | Musculoskeletal | 0.79 | 1.05 | 1.51 | 0.01 | 311242 | 281 | 310961 | FALSE |
| 724.2 | Disorders of coccyx | Musculoskeletal | 0.80 | 1.05 | 1.50 | 0.01 | 311243 | 282 | 310961 | FALSE |
| 580.2 | Nephrotic syndrome without mention of glomerulonephritis | Genitourinary | 1.17 | 0.75 | 0.97 | 0.01 | 314592 | 496 | 314096 | FALSE |
| 916 | Contusion | Injuries & Poisonings | 0.91 | 1.02 | 1.19 | 0.01 | 328257 | 1478 | 326779 | FALSE |
| 428.1 | Congestive heart failure (CHF) NOS | Circulatory System | 1.08 | 0.87 | 0.98 | 0.01 | 324103 | 2053 | 322050 | FALSE |
| 618 | Genital prolapse | Genitourinary | 1.15 | 0.77 | 0.97 | 0.01 | 316879 | 620 | 316259 | FALSE |
| 740.2 | Osteoarthrosis, generalized | Musculoskeletal | 1.15 | 0.77 | 0.97 | 0.02 | 306931 | 589 | 306342 | FALSE |
| 379.2 | Disorders of vitreous body | Sense Organs | 0.91 | 1.02 | 1.19 | 0.02 | 289616 | 1407 | 288209 | FALSE |
| 642 | Hypertension complicating pregnancy, childbirth, and the puerperium | Pregnancy Complications | 1.12 | 0.82 | 0.98 | 0.02 | 328056 | 989 | 327067 | FALSE |
| 293.1 | Swelling, mass, or lump in head and neck [Space-occupying lesion, intracranial NOS] | Mental Disorders | 0.89 | 1.02 | 1.24 | 0.02 | 326061 | 925 | 325136 | FALSE |
| 427.8 | Sinoatrial node dysfunction (Bradycardia) | Circulatory System | 0.84 | 1.03 | 1.37 | 0.02 | 299957 | 419 | 299538 | FALSE |
| 791 | Gangrene | Symptoms | 1.15 | 0.77 | 0.98 | 0.02 | 328257 | 579 | 327678 | FALSE |
| 610.2 | Fibroadenosis of breast | Genitourinary | 1.20 | 0.71 | 0.98 | 0.02 | 321206 | 316 | 320890 | FALSE |
| 227.3 | Benign neoplasm of pituitary gland and craniopharyngeal duct (pouch) | Neoplasms | 0.83 | 1.03 | 1.42 | 0.02 | 326987 | 328 | 326659 | FALSE |
| 70.3 | Viral hepatitis C | Infectious Diseases | 1.19 | 0.72 | 0.98 | 0.02 | 322566 | 343 | 322223 | FALSE |
| 783 | Fever of unknown origin | Symptoms | 0.95 | 1.01 | 1.10 | 0.03 | 328257 | 4111 | 324146 | FALSE |

| | | | | | | | | | | | | |
|---|---|---|---|---|---|---|---|---|---|---|---|---|
| 250.6 | Polyneuropathy in diabetes | Endocrine/Metabolic | 1.18 | 0.73 | 0.98 | 0.03 | 307888 | 381 | 307507 | FALSE |
| 561 | Symptoms involving digestive system | Digestive | 0.97 | 1.00 | 1.05 | 0.03 | 272958 | 14545 | 258413 | FALSE |
| 187.1 | Malignant neoplasm of unspecified male genital organ | Neoplasms | 1.04 | 0.93 | 1.00 | 0.03 | 327716 | 8481 | 319235 | FALSE |
| 291.8 | Alteration of consciousness | Mental Disorders | 0.86 | 1.01 | 1.35 | 0.03 | 321921 | 434 | 321487 | FALSE |
| 250.1 | Type 1 diabetes | Endocrine/Metabolic | 1.06 | 0.89 | 1.00 | 0.03 | 310206 | 2699 | 307507 | FALSE |
| 626.8 | Infertility, female | Genitourinary | 1.09 | 0.85 | 0.99 | 0.03 | 297655 | 1407 | 296248 | FALSE |
| 496.21 | Obstructive chronic bronchitis | Respiratory | 1.06 | 0.89 | 1.00 | 0.04 | 296938 | 2729 | 294209 | FALSE |
| 360.2 | Progressive myopia | Sense Organs | 0.82 | 1.01 | 1.47 | 0.04 | 317049 | 250 | 316799 | FALSE |
| 735.23 | Hallux rigidus | Musculoskeletal | 0.92 | 1.00 | 1.17 | 0.04 | 318260 | 1556 | 316704 | FALSE |
| 185 | Cancer of prostate | Neoplasms | 1.03 | 0.94 | 1.00 | 0.04 | 315873 | 8463 | 307410 | FALSE |
| 618.2 | Uterine/Uterovaginal prolapse | Genitourinary | 1.04 | 0.92 | 1.00 | 0.04 | 321730 | 5471 | 316259 | FALSE |
| 701 | Other hypertrophic and atrophic conditions of skin | Dermatologic | 0.93 | 1.00 | 1.15 | 0.04 | 324908 | 1827 | 323081 | FALSE |
| 714.1 | Rheumatoid arthritis | Musculoskeletal | 1.06 | 0.89 | 1.00 | 0.04 | 319577 | 2307 | 317270 | FALSE |
| 519.9 | Symptoms involving respiratory system and other chest symptoms | Respiratory | 0.84 | 1.00 | 1.41 | 0.04 | 265772 | 308 | 265464 | FALSE |
| 272.11 | Hypercholesterolemia | Endocrine/Metabolic | 1.02 | 0.97 | 1.00 | 0.04 | 325516 | 34002 | 291514 | FALSE |
| 528.11 | Stomatitis and mucositis (ulcerative) | Digestive | 0.86 | 1.00 | 1.34 | 0.05 | 322962 | 411 | 322551 | FALSE |
| 253.2 | Pituitary hypofunction | Endocrine/Metabolic | 0.84 | 1.00 | 1.43 | 0.05 | 324843 | 281 | 324562 | FALSE |
| 721.8 | Other allied disorders of spine | Musculoskeletal | 0.95 | 1.00 | 1.11 | 0.05 | 314188 | 3227 | 310961 | FALSE |
| 290.11 | Alzheimer's disease | Mental Disorders | 0.86 | 1.00 | 1.35 | 0.05 | 321889 | 402 | 321487 | FALSE |
| 351 | Other peripheral nerve disorders | Neurological | 1.03 | 0.95 | 1.00 | 0.05 | 325920 | 12892 | 313028 | FALSE |
| 720 | Spinal stenosis | Musculoskeletal | 0.89 | 1.00 | 1.25 | 0.05 | 311653 | 692 | 310961 | FALSE |
| 386.9 | Dizziness and giddiness (Light-headedness and vertigo) | Sense Organs | 0.96 | 1.00 | 1.09 | 0.05 | 326405 | 4739 | 321666 | FALSE |
| 323 | Encephalitis | Neurological | 0.91 | 1.00 | 1.22 | 0.05 | 328016 | 861 | 327155 | FALSE |
| 300.13 | Phobia | Mental Disorders | 0.88 | 1.00 | 1.29 | 0.05 | 283429 | 514 | 282915 | FALSE |
| 149 | Cancer of larynx, pharynx, nasal cavities | Neoplasms | 0.91 | 1.00 | 1.21 | 0.05 | 327003 | 945 | 326058 | FALSE |
| 669 | Complications of labor and delivery NEC | Pregnancy Complications | 1.04 | 0.93 | 1.00 | 0.05 | 328257 | 9534 | 318723 | FALSE |
| 272.1 | Hyperlipidemia | Endocrine/Metabolic | 0.96 | 1.00 | 1.08 | 0.05 | 296745 | 5231 | 291514 | FALSE |
| 528.7 | Sialolithiasis | Digestive | 0.85 | 1.00 | 1.39 | 0.05 | 322864 | 313 | 322551 | FALSE |
| 362.4 | Retinal vascular changes and abnomalities | Sense Organs | 0.91 | 1.00 | 1.22 | 0.05 | 316750 | 881 | 315869 | FALSE |
| 225.1 | Benign neoplasm of brain, cranial nerves, meninges | Neoplasms | 0.90 | 1.00 | 1.22 | 0.05 | 327284 | 820 | 326464 | FALSE |
| 269 | Proteinuria | Endocrine/Metabolic | 1.16 | 0.74 | 1.00 | 0.06 | 327867 | 355 | 327512 | FALSE |
| 180.1 | Cervical cancer | Neoplasms | 1.04 | 0.92 | 1.00 | 0.06 | 302154 | 4554 | 297600 | FALSE |
| 634 | Miscarriage; stillbirth | Pregnancy Complications | 1.05 | 0.91 | 1.00 | 0.06 | 324551 | 4569 | 319982 | FALSE |
| 187.2 | Malignant neoplasm of testis | Neoplasms | 1.04 | 0.92 | 1.00 | 0.06 | 323807 | 4572 | 319235 | FALSE |
| 280.2 | Iron deficiency anemia secondary to blood loss (chronic) | Hematopoietic | 1.13 | 0.77 | 1.01 | 0.06 | 309393 | 476 | 308917 | FALSE |
| 348.2 | Cerebral edema and compression of brain | Neurological | 1.18 | 0.71 | 1.01 | 0.07 | 286429 | 260 | 286169 | FALSE |
| 426.31 | Right bundle branch block | Circulatory System | 1.07 | 0.87 | 1.00 | 0.07 | 301111 | 1573 | 299538 | FALSE |
| 426.21 | First degree AV block | Circulatory System | 1.09 | 0.84 | 1.01 | 0.07 | 300543 | 1005 | 299538 | FALSE |

| | | | | | | | | | | |
|---|---|---|---|---|---|---|---|---|---|---|
| 289.3 | Personal history of diseases of blood and blood-forming organs | Hematopoietic | 1.17 | 0.73 | 1.01 | 0.07 | 320632 | 297 | 320335 | FALSE |
| 394 | Rheumatic disease of the heart valves | Circulatory System | 1.09 | 0.84 | 1.01 | 0.07 | 322488 | 943 | 321545 | FALSE |
| 598 | Abnormal findings on examination of urine | Genitourinary | 1.05 | 0.91 | 1.00 | 0.07 | 328257 | 3386 | 324871 | FALSE |
| 472 | Chronic pharyngitis and nasopharyngitis | Respiratory | 0.92 | 0.99 | 1.20 | 0.07 | 309914 | 947 | 308967 | FALSE |
| 562.1 | Diverticulosis | Digestive | 1.02 | 0.97 | 1.00 | 0.07 | 286298 | 27885 | 258413 | FALSE |
| 514 | Abnormal findings examination of lungs | Respiratory | 1.05 | 0.90 | 1.01 | 0.08 | 328229 | 2577 | 325652 | FALSE |
| 655 | Known or suspected fetal abnormality affecting management of mother | Pregnancy Complications | 1.04 | 0.92 | 1.00 | 0.08 | 328257 | 4575 | 323682 | FALSE |
| 318 | Tobacco use disorder | Mental Disorders | 0.98 | 1.00 | 1.04 | 0.08 | 318189 | 20503 | 297686 | FALSE |
| 627.3 | Postmenopausal atrophic vaginitis | Genitourinary | 0.92 | 0.99 | 1.18 | 0.08 | 297376 | 1128 | 296248 | FALSE |
| 702.2 | Seborrheic keratosis | Dermatologic | 0.95 | 0.99 | 1.10 | 0.08 | 325786 | 3169 | 322617 | FALSE |
| 420.3 | Endocarditis | Circulatory System | 1.10 | 0.81 | 1.01 | 0.08 | 325685 | 685 | 325000 | FALSE |
| 215 | Other benign neoplasm of connective and other soft tissue | Neoplasms | 1.08 | 0.85 | 1.01 | 0.08 | 321821 | 1128 | 320693 | FALSE |
| 275.3 | Disorders of magnesium metabolism | Endocrine/Metabolic | 0.87 | 0.98 | 1.33 | 0.08 | 326427 | 375 | 326052 | FALSE |
| 454.1 | Varicose veins of lower extremity | Circulatory System | 0.98 | 1.00 | 1.05 | 0.08 | 299292 | 11562 | 287730 | FALSE |
| 189.2 | Cancer of bladder | Neoplasms | 1.09 | 0.83 | 1.01 | 0.08 | 324815 | 836 | 323979 | FALSE |
| 575.1 | Cholangitis | Digestive | 0.89 | 0.98 | 1.28 | 0.09 | 310837 | 497 | 310340 | FALSE |
| 512.9 | Other dyspnea | Respiratory | 1.08 | 0.84 | 1.01 | 0.09 | 315228 | 943 | 314285 | FALSE |
| 80 | Postoperative infection | Infectious Diseases | 1.04 | 0.92 | 1.01 | 0.09 | 326118 | 4622 | 321496 | FALSE |
| 272.9 | Unspecified disorder of lipoid metabolism | Endocrine/Metabolic | 1.18 | 0.70 | 1.02 | 0.09 | 291738 | 224 | 291514 | FALSE |
| 352.1 | Trigeminal nerve disorders [CN5] | Neurological | 1.12 | 0.78 | 1.02 | 0.09 | 313490 | 462 | 313028 | FALSE |
| 442.11 | Abdominal aortic aneurysm | Circulatory System | 1.09 | 0.84 | 1.01 | 0.09 | 320274 | 897 | 319377 | FALSE |
| 349 | Other and unspecified disorders of the nervous system | Neurological | 1.01 | 0.97 | 1.00 | 0.09 | 326872 | 40703 | 286169 | FALSE |
| 420.2 | Pericarditis | Circulatory System | 0.93 | 0.99 | 1.18 | 0.09 | 326087 | 1087 | 325000 | FALSE |
| 368.4 | Visual field defects | Sense Organs | 1.14 | 0.75 | 1.02 | 0.09 | 325232 | 333 | 324899 | FALSE |
| 550 | Abdominal hernia | Digestive | 0.99 | 1.00 | 1.03 | 0.10 | 328257 | 48451 | 279806 | FALSE |
| 151 | Cancer of stomach | Neoplasms | 0.90 | 0.98 | 1.25 | 0.10 | 312922 | 574 | 312348 | FALSE |
| 530.1 | Esophagitis, GERD and related diseases | Digestive | 0.97 | 1.00 | 1.06 | 0.10 | 297359 | 9539 | 287820 | FALSE |
| 380 | Disorders of external ear | Sense Organs | 1.06 | 0.87 | 1.01 | 0.10 | 328244 | 1475 | 326769 | FALSE |
| 260.6 | Anorexia | Endocrine/Metabolic | 0.92 | 0.98 | 1.20 | 0.10 | 326653 | 850 | 325803 | FALSE |
| 278.1 | Obesity | Endocrine/Metabolic | 0.98 | 1.00 | 1.05 | 0.10 | 328127 | 11143 | 316984 | FALSE |
| 304 | Adjustment reaction | Mental Disorders | 0.87 | 0.97 | 1.35 | 0.11 | 283238 | 323 | 282915 | FALSE |
| 458.1 | Orthostatic hypotension | Circulatory System | 0.94 | 0.99 | 1.16 | 0.11 | 195570 | 1374 | 194196 | FALSE |
| 771 | Musculoskeletal symptoms referable to limbs | Symptoms | 1.08 | 0.84 | 1.02 | 0.11 | 327422 | 891 | 326531 | FALSE |
| 433.12 | Cerebral atherosclerosis | Circulatory System | 1.16 | 0.72 | 1.03 | 0.11 | 318362 | 238 | 318124 | FALSE |
| 717 | Polymyalgia Rheumatica | Musculoskeletal | 1.07 | 0.86 | 1.02 | 0.11 | 328257 | 1147 | 327110 | FALSE |
| 737.3 | Kyphoscoliosis and scoliosis | Musculoskeletal | 1.13 | 0.76 | 1.03 | 0.11 | 317075 | 371 | 316704 | FALSE |
| 636.2 | Early onset of delivery | Pregnancy Complications | 1.10 | 0.81 | 1.02 | 0.11 | 320611 | 629 | 319982 | FALSE |
| 217.1 | Nevus, non-neoplastic | Neoplasms | 1.10 | 0.81 | 1.02 | 0.11 | 320363 | 597 | 319766 | FALSE |

| | | | | | | | | | | | |
|---|---|---|---|---|---|---|---|---|---|---|---|
| 941 | Adverse reaction to serum or vaccine | Injuries & Poisonings | 0.85 | 0.96 | 1.43 | 0.12 | 322145 | 222 | 321923 | FALSE |
| 345 | Epilepsy, recurrent seizures, convulsions | Neurological | 1.04 | 0.92 | 1.01 | 0.12 | 289630 | 3461 | 286169 | FALSE |
| 575.2 | Obstruction of bile duct | Digestive | 1.08 | 0.83 | 1.02 | 0.12 | 311152 | 812 | 310340 | FALSE |
| 722.9 | Other and unspecified disc disorder | Musculoskeletal | 0.96 | 0.99 | 1.09 | 0.12 | 314911 | 3950 | 310961 | FALSE |
| 751.21 | Cystic kidney disease | Congenital Anomalies | 0.90 | 0.97 | 1.28 | 0.12 | 326478 | 458 | 326020 | FALSE |
| 967 | Adverse effects of sedatives or other central nervous system depressants and anesthetics | Injuries & Poisonings | 1.10 | 0.81 | 1.02 | 0.12 | 300807 | 579 | 300228 | FALSE |
| 870.3 | Other open wound of head and face | Injuries & Poisonings | 0.96 | 0.99 | 1.10 | 0.12 | 322799 | 3051 | 319748 | FALSE |
| 394.7 | Disease of tricuspid valve | Circulatory System | 0.93 | 0.98 | 1.17 | 0.12 | 322631 | 1086 | 321545 | FALSE |
| 740.12 | Osteoarthrosis, localized, secondary | Musculoskeletal | 0.87 | 0.96 | 1.38 | 0.12 | 306606 | 264 | 306342 | FALSE |
| 480.5 | Bronchopneumonia and lung abscess | Respiratory | 1.12 | 0.77 | 1.03 | 0.12 | 318094 | 388 | 317706 | FALSE |
| 578.1 | Hematemesis | Digestive | 0.95 | 0.99 | 1.12 | 0.12 | 305445 | 2034 | 303411 | FALSE |
| 610 | Benign mammary dysplasias | Genitourinary | 0.85 | 0.96 | 1.44 | 0.13 | 321098 | 208 | 320890 | FALSE |
| 378.5 | Paralytic strabismus | Sense Organs | 0.87 | 0.96 | 1.36 | 0.13 | 288496 | 287 | 288209 | FALSE |
| 38.2 | Gram positive septicemia | Infectious Diseases | 0.90 | 0.97 | 1.26 | 0.13 | 313140 | 491 | 312649 | FALSE |
| 530.5 | Disorders of esophageal motility | Digestive | 0.91 | 0.97 | 1.23 | 0.13 | 288469 | 649 | 287820 | FALSE |
| 623 | Hypertrophy of female genital organs | Genitourinary | 0.94 | 0.98 | 1.16 | 0.13 | 316596 | 1272 | 315324 | FALSE |
| 327 | Sleep disorders | Neurological | 1.07 | 0.85 | 1.02 | 0.13 | 323771 | 1034 | 322737 | FALSE |
| 184.11 | Malignant neoplasm of ovary | Neoplasms | 1.03 | 0.93 | 1.01 | 0.13 | 310579 | 5009 | 305570 | FALSE |
| 348.8 | Encephalopathy, not elsewhere classified | Neurological | 1.17 | 0.70 | 1.05 | 0.13 | 286373 | 204 | 286169 | FALSE |
| 714 | Rheumatoid arthritis and other inflammatory polyarthropathies | Musculoskeletal | 1.02 | 0.95 | 1.01 | 0.13 | 325922 | 8652 | 317270 | FALSE |
| 158 | Neoplasm of unspecified nature of digestive system | Neoplasms | 1.07 | 0.86 | 1.02 | 0.14 | 313446 | 1098 | 312348 | FALSE |
| 350.1 | Abnormal involuntary movements | Neurological | 0.93 | 0.98 | 1.18 | 0.14 | 326388 | 927 | 325461 | FALSE |
| 496.2 | Chronic bronchitis | Respiratory | 1.13 | 0.75 | 1.04 | 0.14 | 294502 | 293 | 294209 | FALSE |
| 362 | Other retinal disorders | Sense Organs | 0.93 | 0.98 | 1.18 | 0.14 | 316842 | 973 | 315869 | FALSE |
| 614.33 | Pelvic inflammatory disease, NOS | Genitourinary | 1.03 | 0.94 | 1.01 | 0.14 | 324961 | 6145 | 318816 | FALSE |
| 292.4 | Altered mental status | Mental Disorders | 1.05 | 0.90 | 1.01 | 0.14 | 323759 | 2272 | 321487 | FALSE |
| 315 | Develomental delays and disorders | Mental Disorders | 0.91 | 0.97 | 1.24 | 0.14 | 327009 | 549 | 326460 | FALSE |
| 564.9 | Personal history of diseases of digestive system | Digestive | 0.98 | 0.99 | 1.04 | 0.14 | 274337 | 15924 | 258413 | FALSE |
| 560.2 | Impaction of intestine | Digestive | 0.87 | 0.96 | 1.37 | 0.14 | 258684 | 271 | 258413 | FALSE |
| 324 | Other CNS infection and poliomyelitis | Neurological | 0.88 | 0.96 | 1.35 | 0.14 | 327443 | 288 | 327155 | FALSE |
| 527.2 | Sialoadenitis | Digestive | 1.12 | 0.77 | 1.04 | 0.14 | 322925 | 374 | 322551 | FALSE |
| 742.9 | Other derangement of joint | Musculoskeletal | 1.11 | 0.78 | 1.04 | 0.14 | 311059 | 400 | 310659 | FALSE |
| 706 | Diseases of sebaceous glands | Dermatologic | 1.09 | 0.81 | 1.03 | 0.14 | 318906 | 595 | 318311 | FALSE |
| 573.5 | Jaundice (not of newborn) | Digestive | 0.93 | 0.98 | 1.18 | 0.14 | 319286 | 938 | 318348 | FALSE |
| 603.1 | Hydrocele | Genitourinary | 1.06 | 0.87 | 1.02 | 0.14 | 309325 | 1402 | 307923 | FALSE |
| 250.41 | Impaired fasting glucose | Endocrine/Metabolic | 1.14 | 0.74 | 1.05 | 0.14 | 307775 | 268 | 307507 | FALSE |
| 250.11 | Type 1 diabetes with ketoacidosis | Endocrine/Metabolic | 1.14 | 0.73 | 1.05 | 0.14 | 307762 | 255 | 307507 | FALSE |
| 870.4 | Open wound of nose and sinus | Injuries & Poisonings | 0.87 | 0.95 | 1.39 | 0.15 | 319986 | 238 | 319748 | FALSE |

| | | | | | | | | | | |
|---|---|---|---|---|---|---|---|---|---|---|
| 574.3 | Cholecystitis without cholelithiasis | Digestive | 1.04 | 0.91 | 1.01 | 0.15 | 313158 | 2818 | 310340 | FALSE |
| 367.9 | Blindness and low vision | Sense Organs | 0.92 | 0.97 | 1.20 | 0.15 | 326527 | 756 | 325771 | FALSE |
| 749 | Congenital anomalies of face and neck | Congenital Anomalies | 1.14 | 0.74 | 1.05 | 0.15 | 328154 | 271 | 327883 | FALSE |
| 368.9 | Subjective visual disturbances | Sense Organs | 1.09 | 0.82 | 1.03 | 0.15 | 325532 | 633 | 324899 | FALSE |
| 276.11 | Hyperosmolality and/or hypernatremia | Endocrine/Metabolic | 0.87 | 0.95 | 1.38 | 0.15 | 320908 | 247 | 320661 | FALSE |
| 415.21 | Primary pulmonary hypertension | Circulatory System | 1.10 | 0.79 | 1.04 | 0.15 | 322030 | 466 | 321564 | FALSE |
| 790.6 | Other abnormal blood chemistry | Symptoms | 1.03 | 0.94 | 1.01 | 0.15 | 328046 | 6529 | 321517 | FALSE |
| 550.5 | Ventral hernia | Digestive | 0.96 | 0.99 | 1.09 | 0.15 | 283388 | 3582 | 279806 | FALSE |
| 184.2 | Cancer of other female genital organs | Neoplasms | 1.12 | 0.76 | 1.04 | 0.15 | 305893 | 323 | 305570 | FALSE |
| 759 | Other and unspecified congenital anomalies | Congenital Anomalies | 1.08 | 0.83 | 1.03 | 0.15 | 327266 | 679 | 326587 | FALSE |
| 575.9 | Nonspecific abnormal findings on radiological and other examination of biliary tract | Digestive | 0.89 | 0.96 | 1.30 | 0.15 | 310707 | 367 | 310340 | FALSE |
| 853 | Complication of colostomy or enterostomy | Injuries & Poisonings | 1.10 | 0.80 | 1.04 | 0.15 | 314495 | 514 | 313981 | FALSE |
| 807 | Fracture of ribs | Injuries & Poisonings | 0.87 | 0.95 | 1.41 | 0.15 | 323797 | 223 | 323574 | FALSE |
| 316 | Substance addiction and disorders | Mental Disorders | 1.11 | 0.78 | 1.04 | 0.16 | 298059 | 373 | 297686 | FALSE |
| 594.2 | Calculus of lower urinary tract | Genitourinary | 1.07 | 0.84 | 1.03 | 0.16 | 320778 | 830 | 319948 | FALSE |
| 614.53 | Cyst or abscess of Bartholin's gland | Genitourinary | 1.08 | 0.84 | 1.03 | 0.16 | 319619 | 803 | 318816 | FALSE |
| 614.51 | Cervicitis and endocervicitis | Genitourinary | 1.06 | 0.87 | 1.02 | 0.16 | 320065 | 1249 | 318816 | FALSE |
| 289.8 | Polycythemia vera, secondary | Hematopoietic | 0.88 | 0.95 | 1.34 | 0.16 | 320056 | 291 | 319765 | FALSE |
| 695.7 | Prurigo and Lichen | Dermatologic | 0.93 | 0.97 | 1.19 | 0.16 | 322558 | 792 | 321766 | FALSE |
| 379 | Other disorders of eye | Sense Organs | 0.99 | 1.00 | 1.03 | 0.16 | 328207 | 39998 | 288209 | FALSE |
| 389 | Hearing loss | Sense Organs | 0.96 | 0.98 | 1.09 | 0.17 | 321381 | 3170 | 318211 | FALSE |
| 871 | Open wounds of extremities | Injuries & Poisonings | 1.03 | 0.93 | 1.01 | 0.17 | 323890 | 4142 | 319748 | FALSE |
| 530.14 | Reflux esophagitis | Digestive | 0.98 | 0.99 | 1.05 | 0.17 | 298713 | 10893 | 287820 | FALSE |
| 174.1 | Breast cancer [female] | Neoplasms | 1.04 | 0.90 | 1.02 | 0.17 | 306728 | 2287 | 304441 | FALSE |
| 288 | Diseases of white blood cells | Hematopoietic | 0.92 | 0.97 | 1.22 | 0.17 | 320945 | 610 | 320335 | FALSE |
| 389.2 | Conductive hearing loss | Sense Organs | 0.91 | 0.96 | 1.27 | 0.17 | 318646 | 435 | 318211 | FALSE |
| 345.3 | Convulsions | Neurological | 1.04 | 0.90 | 1.02 | 0.17 | 288480 | 2311 | 286169 | FALSE |
| 949 | Allergies, other | Injuries & Poisonings | 1.10 | 0.79 | 1.04 | 0.17 | 322343 | 420 | 321923 | FALSE |
| 531 | Peptic ulcer (excl. esophageal) | Digestive | 0.89 | 0.95 | 1.34 | 0.17 | 320850 | 284 | 320566 | FALSE |
| 501 | Pneumonitis due to inhalation of food or vomitus | Respiratory | 1.09 | 0.82 | 1.04 | 0.17 | 317093 | 592 | 316501 | FALSE |
| 653 | Problems associated with amniotic cavity and membranes | Pregnancy Complications | 1.05 | 0.88 | 1.02 | 0.17 | 328136 | 1523 | 326613 | FALSE |
| 513.4 | Hyperventilation | Respiratory | 1.13 | 0.75 | 1.05 | 0.17 | 327921 | 280 | 327641 | FALSE |
| 229 | Benign neoplasm of unspecified sites | Neoplasms | 0.99 | 1.00 | 1.02 | 0.17 | 328257 | 46165 | 282092 | FALSE |
| 965.2 | Antirheumatics causing adverse effects in therapeutic use | Injuries & Poisonings | 0.91 | 0.96 | 1.25 | 0.18 | 300727 | 499 | 300228 | FALSE |
| 201 | Hodgkin's disease | Neoplasms | 1.13 | 0.75 | 1.06 | 0.18 | 323811 | 271 | 323540 | FALSE |
| 958 | Certain early complications of trauma or procedure | Injuries & Poisonings | 1.10 | 0.78 | 1.05 | 0.18 | 328135 | 381 | 327754 | FALSE |
| 508 | Pulmonary collapse; interstitial and compensatory emphysema | Respiratory | 0.96 | 0.98 | 1.11 | 0.18 | 318587 | 2086 | 316501 | FALSE |
| 751.11 | Congenital anomalies of female genital organs | Congenital Anomalies | 1.10 | 0.80 | 1.04 | 0.18 | 326462 | 442 | 326020 | FALSE |

| | | | | | | | | | | | | |
|---|---|---|---|---|---|---|---|---|---|---|---|---|
| 202.24 | Large cell lymphoma | Neoplasms | 0.92 | 0.96 | 1.22 | 0.18 | 324129 | 589 | 323540 | FALSE |
| 411.2 | Myocardial infarction | Circulatory System | 1.02 | 0.96 | 1.01 | 0.18 | 307675 | 11969 | 295706 | FALSE |
| 275.1 | Disorders of iron metabolism | Hematopoietic | 1.08 | 0.83 | 1.04 | 0.19 | 326752 | 700 | 326052 | FALSE |
| 323.8 | Encephalitis, non-infectious | Neurological | 0.92 | 0.96 | 1.21 | 0.19 | 327788 | 633 | 327155 | FALSE |
| 395.2 | Nonrheumatic aortic valve disorders | Circulatory System | 1.13 | 0.74 | 1.06 | 0.19 | 321792 | 247 | 321545 | FALSE |
| 695.42 | Systemic lupus erythematosus | Dermatologic | 1.11 | 0.78 | 1.05 | 0.19 | 320801 | 363 | 320438 | FALSE |
| 210 | Benign neoplasm of lip, oral cavity, and pharynx | Neoplasms | 0.94 | 0.97 | 1.17 | 0.19 | 327051 | 993 | 326058 | FALSE |
| 681.6 | Cellulitis and abscess of foot, toe | Dermatologic | 0.97 | 0.99 | 1.07 | 0.19 | 322158 | 5734 | 316424 | FALSE |
| 726.2 | Synoviopathy | Musculoskeletal | 0.90 | 0.95 | 1.31 | 0.19 | 305302 | 331 | 304971 | FALSE |
| 715.2 | Ankylosing spondylitis | Musculoskeletal | 0.91 | 0.95 | 1.28 | 0.19 | 317654 | 384 | 317270 | FALSE |
| 601.8 | Other inflammatory disorders of male genital organs | Genitourinary | 1.11 | 0.76 | 1.06 | 0.19 | 307715 | 305 | 307410 | FALSE |
| 70 | Viral hepatitis | Infectious Diseases | 1.07 | 0.84 | 1.04 | 0.20 | 322945 | 722 | 322223 | FALSE |
| 474.2 | Chronic tonsillitis and adenoiditis | Respiratory | 0.95 | 0.97 | 1.15 | 0.20 | 310152 | 1185 | 308967 | FALSE |
| 738 | Other acquired musculoskeletal deformity | Musculoskeletal | 0.91 | 0.95 | 1.27 | 0.20 | 317121 | 417 | 316704 | FALSE |
| 624.9 | stress incontinence, female | Genitourinary | 1.02 | 0.94 | 1.01 | 0.20 | 324786 | 6100 | 318686 | FALSE |
| 681.5 | Cellulitis and abscess of leg, except foot | Dermatologic | 0.98 | 0.99 | 1.07 | 0.20 | 322201 | 5777 | 316424 | FALSE |
| 735.2 | Acquired toe deformities | Musculoskeletal | 1.04 | 0.90 | 1.02 | 0.21 | 318930 | 2226 | 316704 | FALSE |
| 300.1 | Anxiety disorder | Mental Disorders | 1.03 | 0.94 | 1.01 | 0.21 | 288385 | 5470 | 282915 | FALSE |
| 374.3 | Ptosis of eyelid | Sense Organs | 0.96 | 0.98 | 1.12 | 0.21 | 320301 | 1896 | 318405 | FALSE |
| 38.1 | Gram negative septicemia | Infectious Diseases | 0.94 | 0.96 | 1.18 | 0.21 | 313504 | 855 | 312649 | FALSE |
| 290.1 | Dementias | Mental Disorders | 0.93 | 0.96 | 1.21 | 0.22 | 322138 | 651 | 321487 | FALSE |
| 601.4 | Balanoposthitis | Genitourinary | 1.11 | 0.76 | 1.06 | 0.22 | 307706 | 296 | 307410 | FALSE |
| 737 | Curvature of spine | Musculoskeletal | 1.07 | 0.84 | 1.04 | 0.22 | 317447 | 743 | 316704 | FALSE |
| 381.9 | Otorrhea | Sense Organs | 0.90 | 0.94 | 1.33 | 0.22 | 324437 | 277 | 324160 | FALSE |
| 286.12 | Congenital deficiency of other clotting factors (including factor VII) | Hematopoietic | 1.11 | 0.77 | 1.06 | 0.22 | 325817 | 303 | 325514 | FALSE |
| 174.11 | Malignant neoplasm of female breast | Neoplasms | 1.02 | 0.96 | 1.01 | 0.22 | 316855 | 12414 | 304441 | FALSE |
| 433.1 | Occlusion and stenosis of precerebral arteries | Circulatory System | 0.95 | 0.97 | 1.16 | 0.22 | 319166 | 1042 | 318124 | FALSE |
| 578.8 | Hemorrhage of rectum and anus | Digestive | 0.98 | 0.99 | 1.04 | 0.22 | 317237 | 13826 | 303411 | FALSE |
| 281 | Other deficiency anemia | Hematopoietic | 1.02 | 0.95 | 1.01 | 0.22 | 317704 | 8787 | 308917 | FALSE |
| 458.9 | Hypotension NOS | Circulatory System | 0.97 | 0.98 | 1.08 | 0.22 | 197834 | 3638 | 194196 | FALSE |
| 504 | Other alveolar and parietoalveolar pneumonopathy | Respiratory | 1.11 | 0.77 | 1.06 | 0.22 | 316810 | 309 | 316501 | FALSE |
| 241.2 | Nontoxic multinodular goiter | Endocrine/Metabolic | 1.07 | 0.84 | 1.04 | 0.22 | 311060 | 714 | 310346 | FALSE |
| 741.4 | Joint effusions | Musculoskeletal | 1.11 | 0.76 | 1.07 | 0.22 | 310942 | 283 | 310659 | FALSE |
| 961.1 | Poisoning/allergy of sulfonamides | Injuries & Poisonings | 0.94 | 0.96 | 1.17 | 0.23 | 301118 | 890 | 300228 | FALSE |
| 277.4 | Disorders of bilirubin excretion | Endocrine/Metabolic | 0.91 | 0.94 | 1.28 | 0.23 | 283317 | 373 | 282944 | FALSE |
| 681.3 | Cellulitis and abscess of arm/hand | Dermatologic | 0.98 | 0.99 | 1.06 | 0.23 | 322194 | 5770 | 316424 | FALSE |
| 426.24 | Atrioventricular block, complete | Circulatory System | 1.08 | 0.83 | 1.05 | 0.23 | 300129 | 591 | 299538 | FALSE |
| 594 | Urinary calculus | Genitourinary | 1.02 | 0.95 | 1.01 | 0.23 | 326914 | 6966 | 319948 | FALSE |

| | | | | | | | | | | |
|---|---|---|---|---|---|---|---|---|---|---|
| 198.1 | Secondary malignancy of lymph nodes | Neoplasms | 0.98 | 0.98 | 1.07 | 0.23 | 234832 | 5503 | 229329 | FALSE |
| 560.1 | Paralytic ileus | Digestive | 0.92 | 0.95 | 1.23 | 0.23 | 258933 | 520 | 258413 | FALSE |
| 317.1 | Alcoholism | Mental Disorders | 0.98 | 0.99 | 1.05 | 0.23 | 306383 | 8697 | 297686 | FALSE |
| 550.3 | Femoral hernia | Digestive | 0.93 | 0.96 | 1.20 | 0.23 | 280459 | 653 | 279806 | FALSE |
| 204.21 | Myeloid leukemia, acute | Neoplasms | 1.10 | 0.77 | 1.06 | 0.23 | 323859 | 319 | 323540 | FALSE |
| 722 | Intervertebral disc disorders | Musculoskeletal | 0.98 | 0.99 | 1.06 | 0.24 | 316924 | 5963 | 310961 | FALSE |
| 526.41 | Temporomandibular joint disorder, unspecified | Digestive | 1.12 | 0.74 | 1.08 | 0.24 | 311903 | 228 | 311675 | FALSE |
| 378.1 | Strabismus (not specified as paralytic) | Sense Organs | 0.95 | 0.96 | 1.16 | 0.24 | 289156 | 947 | 288209 | FALSE |
| 381.11 | Suppurative and unspecified otitis media | Sense Organs | 1.06 | 0.85 | 1.04 | 0.24 | 325010 | 850 | 324160 | FALSE |
| 681.7 | Cellulitis and abscess of trunk | Dermatologic | 1.07 | 0.83 | 1.05 | 0.24 | 317047 | 623 | 316424 | FALSE |
| 535.1 | Acute gastritis | Digestive | 0.95 | 0.97 | 1.14 | 0.25 | 297721 | 1193 | 296528 | FALSE |
| 619.3 | Noninflammatory disorders of cervix | Genitourinary | 0.97 | 0.98 | 1.09 | 0.25 | 281410 | 2845 | 278565 | FALSE |
| 597 | Other disorders of urethra and urinary tract | Genitourinary | 1.06 | 0.86 | 1.04 | 0.25 | 314527 | 874 | 313653 | FALSE |
| 602 | Other disorders of prostate | Genitourinary | 0.95 | 0.97 | 1.13 | 0.25 | 308782 | 1372 | 307410 | FALSE |
| 636 | Early or threatened labor; hemorrhage in early pregnancy | Pregnancy Complications | 1.05 | 0.88 | 1.03 | 0.25 | 321244 | 1262 | 319982 | FALSE |
| 384.4 | Perforation of tympanic membrane | Sense Organs | 1.05 | 0.87 | 1.04 | 0.25 | 325184 | 1024 | 324160 | FALSE |
| 604.1 | Redundant prepuce and phimosis/BXO | Genitourinary | 1.03 | 0.92 | 1.02 | 0.25 | 310733 | 2810 | 307923 | FALSE |
| 218 | Benign neoplasm of uterus | Neoplasms | 0.92 | 0.94 | 1.27 | 0.25 | 308185 | 388 | 307797 | FALSE |
| 740.9 | Osteoarthrosis NOS | Musculoskeletal | 1.03 | 0.93 | 1.02 | 0.25 | 310774 | 4432 | 306342 | FALSE |
| 374 | Other disorders of eyelids | Sense Organs | 0.97 | 0.98 | 1.09 | 0.25 | 321610 | 3205 | 318405 | FALSE |
| 613 | Other nonmalignant breast conditions | Genitourinary | 0.91 | 0.94 | 1.27 | 0.25 | 322803 | 368 | 322435 | FALSE |
| 624.2 | Atrophy of female genital tract | Genitourinary | 1.08 | 0.81 | 1.06 | 0.25 | 319128 | 442 | 318686 | FALSE |
| 371 | Inflammation of the eye | Sense Organs | 1.09 | 0.80 | 1.06 | 0.25 | 318813 | 408 | 318405 | FALSE |
| 705.8 | Hyperhidrosis | Dermatologic | 1.07 | 0.83 | 1.05 | 0.25 | 318913 | 602 | 318311 | FALSE |
| 470 | Septal Deviations/Turbinate Hypertrophy | Respiratory | 1.02 | 0.94 | 1.02 | 0.25 | 313788 | 4821 | 308967 | FALSE |
| 300 | Anxiety, phobic and dissociative disorders | Mental Disorders | 0.91 | 0.94 | 1.27 | 0.25 | 283278 | 363 | 282915 | FALSE |
| 447 | Other disorders of arteries and arterioles | Circulatory System | 0.93 | 0.95 | 1.22 | 0.25 | 319933 | 556 | 319377 | FALSE |
| 202 | Cancer of other lymphoid, histiocytic tissue | Neoplasms | 1.05 | 0.87 | 1.04 | 0.26 | 324678 | 1138 | 323540 | FALSE |
| 153.2 | Colon cancer | Neoplasms | 0.97 | 0.98 | 1.09 | 0.26 | 304419 | 3122 | 301297 | FALSE |
| 427.2 | Atrial fibrillation and flutter | Circulatory System | 0.99 | 0.99 | 1.04 | 0.26 | 314590 | 15052 | 299538 | FALSE |
| 626.13 | Irregular menstrual cycle | Genitourinary | 1.04 | 0.90 | 1.03 | 0.26 | 298206 | 1958 | 296248 | FALSE |
| 823 | Fracture of tibia and fibula | Injuries & Poisonings | 0.91 | 0.94 | 1.28 | 0.26 | 322379 | 342 | 322037 | FALSE |
| 599.2 | Retention of urine | Genitourinary | 1.02 | 0.95 | 1.02 | 0.26 | 227301 | 6857 | 220444 | FALSE |
| 723 | Other disorders of cervical region | Musculoskeletal | 0.92 | 0.94 | 1.26 | 0.26 | 311362 | 401 | 310961 | FALSE |
| 939 | Atopic/contact dermatitis due to other or unspecified | Dermatologic | 0.96 | 0.97 | 1.10 | 0.26 | 324077 | 2154 | 321923 | FALSE |
| 165 | Cancer within the respiratory system | Neoplasms | 0.97 | 0.98 | 1.09 | 0.26 | 328240 | 2954 | 325286 | FALSE |
| 626.1 | Irregular menstrual cycle/bleeding | Genitourinary | 1.03 | 0.93 | 1.02 | 0.26 | 299841 | 3593 | 296248 | FALSE |
| 530.7 | Gastroesophageal laceration-hemorrhage syndrome | Digestive | 0.91 | 0.93 | 1.29 | 0.26 | 288153 | 333 | 287820 | FALSE |

| | | | | | | | | | | |
|---|---|---|---|---|---|---|---|---|---|---|
| 622.1 | Polyp of corpus uteri | Genitourinary | 0.98 | 0.99 | 1.05 | 0.27 | 323507 | 8183 | 315324 | FALSE |
| 564.1 | Irritable Bowel Syndrome | Digestive | 0.98 | 0.98 | 1.06 | 0.27 | 264074 | 5661 | 258413 | FALSE |
| 79 | Viral infection | Infectious Diseases | 1.03 | 0.93 | 1.02 | 0.27 | 326456 | 4233 | 322223 | FALSE |
| 281.11 | Pernicious anemia | Hematopoietic | 1.06 | 0.85 | 1.05 | 0.27 | 309667 | 750 | 308917 | FALSE |
| 338.1 | Acute pain | Neurological | 1.08 | 0.80 | 1.06 | 0.27 | 327863 | 420 | 327443 | FALSE |
| 724.9 | Other unspecified back disorders | Musculoskeletal | 0.99 | 0.99 | 1.04 | 0.28 | 328046 | 17085 | 310961 | FALSE |
| 331 | Other cerebral degenerations | Neurological | 0.90 | 0.92 | 1.34 | 0.28 | 286409 | 240 | 286169 | FALSE |
| 530.12 | Ulcer of esophagus | Digestive | 0.98 | 0.98 | 1.06 | 0.28 | 293168 | 5348 | 287820 | FALSE |
| 573.7 | Abnormal results of function study of liver | Digestive | 0.97 | 0.98 | 1.08 | 0.28 | 321913 | 3565 | 318348 | FALSE |
| 550.2 | Diaphragmatic hernia | Digestive | 0.99 | 0.99 | 1.03 | 0.28 | 307470 | 27664 | 279806 | FALSE |
| 579 | Other symptoms involving abdomen and pelvis | Digestive | 1.05 | 0.88 | 1.04 | 0.28 | 304597 | 1186 | 303411 | FALSE |
| 496 | Chronic airway obstruction | Respiratory | 0.98 | 0.98 | 1.05 | 0.28 | 301844 | 7635 | 294209 | FALSE |
| 619.5 | Noninflammatory disorders of vulva and perineum | Genitourinary | 1.04 | 0.89 | 1.04 | 0.28 | 279944 | 1379 | 278565 | FALSE |
| 523.32 | Chronic periodontitis | Digestive | 1.07 | 0.83 | 1.06 | 0.28 | 312265 | 590 | 311675 | FALSE |
| 340.1 | Migrain with aura | Neurological | 1.11 | 0.74 | 1.09 | 0.28 | 318184 | 217 | 317967 | FALSE |
| 165.1 | Cancer of bronchus; lung | Neoplasms | 0.97 | 0.97 | 1.10 | 0.29 | 327467 | 2181 | 325286 | FALSE |
| 191 | Malignant and unknown neoplasms of brain and nervous system | Neoplasms | 0.90 | 0.91 | 1.36 | 0.29 | 326679 | 215 | 326464 | FALSE |
| 189.21 | Malignant neoplasm of bladder | Neoplasms | 1.03 | 0.91 | 1.03 | 0.29 | 326167 | 2188 | 323979 | FALSE |
| 380.4 | Impacted cerumen | Sense Organs | 1.09 | 0.79 | 1.07 | 0.29 | 327123 | 354 | 326769 | FALSE |
| 371.1 | Uveitis, noninfectious or NOS | Sense Organs | 1.07 | 0.82 | 1.06 | 0.29 | 318903 | 498 | 318405 | FALSE |
| 750.21 | Congenital anomalies of intestine | Congenital Anomalies | 0.90 | 0.91 | 1.35 | 0.29 | 326247 | 227 | 326020 | FALSE |
| 429.2 | Abnormal function study of cardiovascular system | Circulatory System | 0.94 | 0.94 | 1.21 | 0.29 | 322594 | 544 | 322050 | FALSE |
| 302 | Sexual and gender identity disorders | Mental Disorders | 1.09 | 0.79 | 1.07 | 0.29 | 283255 | 340 | 282915 | FALSE |
| 441 | Vascular insufficiency of intestine | Circulatory System | 1.09 | 0.78 | 1.08 | 0.30 | 319674 | 297 | 319377 | FALSE |
| 204.12 | Lymphoid leukemia, chronic | Neoplasms | 0.93 | 0.94 | 1.22 | 0.30 | 324047 | 507 | 323540 | FALSE |
| 614.5 | Inflammatory disease of cervix, vagina, and vulva | Genitourinary | 1.09 | 0.79 | 1.08 | 0.30 | 319150 | 334 | 318816 | FALSE |
| 614.52 | Vaginitis and vulvovaginitis | Genitourinary | 0.93 | 0.94 | 1.24 | 0.30 | 319236 | 420 | 318816 | FALSE |
| 766 | Neuralgia, neuritis, and radiculitis NOS | Symptoms | 1.09 | 0.78 | 1.08 | 0.30 | 327004 | 304 | 326700 | FALSE |
| 350.3 | Lack of coordination | Neurological | 0.92 | 0.93 | 1.27 | 0.30 | 325821 | 360 | 325461 | FALSE |
| 597.1 | Urethral stricture (not specified as infectious) | Genitourinary | 0.97 | 0.98 | 1.08 | 0.30 | 317096 | 3443 | 313653 | FALSE |
| 396 | Abnormal heart sounds | Circulatory System | 1.05 | 0.87 | 1.04 | 0.30 | 322620 | 1075 | 321545 | FALSE |
| 965.3 | Salicylates causing adverse effects in therapeutic use | Injuries & Poisonings | 1.08 | 0.80 | 1.07 | 0.30 | 300629 | 401 | 300228 | FALSE |
| 289.4 | Lymphadenitis | Hematopoietic | 1.03 | 0.92 | 1.03 | 0.31 | 323050 | 2715 | 320335 | FALSE |
| 695.3 | Rosacea | Dermatologic | 1.09 | 0.78 | 1.08 | 0.31 | 322085 | 319 | 321766 | FALSE |
| 771.1 | Swelling of limb | Symptoms | 0.95 | 0.95 | 1.16 | 0.31 | 327373 | 842 | 326531 | FALSE |
| 752 | Nervous system congenital anomalies | Congenital Anomalies | 1.09 | 0.77 | 1.08 | 0.31 | 327980 | 288 | 327692 | FALSE |
| 599 | Other symptoms/disorders or the urinary system | Genitourinary | 1.01 | 0.98 | 1.01 | 0.31 | 324273 | 103829 | 220444 | FALSE |
| 364.5 | Corneal dystrophy | Sense Organs | 1.11 | 0.74 | 1.10 | 0.31 | 317015 | 216 | 316799 | FALSE |

| | | | | | | | | | | |
|---|---|---|---|---|---|---|---|---|---|---|
| 747.1 | Cardiac congenital anomalies | Congenital Anomalies | 1.11 | 0.74 | 1.10 | 0.31 | 325582 | 211 | 325371 | FALSE |
| 751.1 | Congenital anomalies of genital organs | Congenital Anomalies | 1.06 | 0.85 | 1.05 | 0.31 | 326726 | 706 | 326020 | FALSE |
| 615 | Endometriosis | Genitourinary | 1.02 | 0.93 | 1.02 | 0.31 | 322905 | 4089 | 318816 | FALSE |
| 686.4 | Pyogenic granuloma | Dermatologic | 0.93 | 0.93 | 1.25 | 0.31 | 316804 | 380 | 316424 | FALSE |
| 603 | Other disorders of testis | Genitourinary | 1.05 | 0.87 | 1.05 | 0.32 | 308912 | 989 | 307923 | FALSE |
| 962.3 | Hormones and synthetic substitutes causing adverse effects in therapeutic use | Injuries & Poisonings | 1.08 | 0.80 | 1.08 | 0.32 | 300592 | 364 | 300228 | FALSE |
| 622.2 | Mucous polyp of cervix | Genitourinary | 0.98 | 0.98 | 1.08 | 0.32 | 318846 | 3522 | 315324 | FALSE |
| 800.1 | Fracture of neck of femur | Injuries & Poisonings | 0.91 | 0.91 | 1.33 | 0.32 | 323813 | 239 | 323574 | FALSE |
| 578.2 | Blood in stool | Digestive | 1.03 | 0.92 | 1.03 | 0.32 | 306160 | 2749 | 303411 | FALSE |
| 686 | Other local infections of skin and subcutaneous tissue | Dermatologic | 0.99 | 0.99 | 1.04 | 0.32 | 327427 | 11003 | 316424 | FALSE |
| 703.1 | Ingrowing nail | Dermatologic | 1.05 | 0.87 | 1.05 | 0.33 | 322456 | 1010 | 321446 | FALSE |
| 965.1 | Opiates and related narcotics causing adverse effects in therapeutic use | Injuries & Poisonings | 0.96 | 0.96 | 1.14 | 0.33 | 301389 | 1161 | 300228 | FALSE |
| 202.21 | Nodular lymphoma | Neoplasms | 1.08 | 0.80 | 1.08 | 0.33 | 323930 | 390 | 323540 | FALSE |
| 591 | Urinary tract infection | Genitourinary | 1.01 | 0.96 | 1.01 | 0.33 | 309728 | 12676 | 297052 | FALSE |
| 726 | Peripheral enthesopathies and allied syndromes | Musculoskeletal | 1.02 | 0.95 | 1.02 | 0.33 | 313739 | 8768 | 304971 | FALSE |
| 635.3 | Placenta previa and abruptio placenta | Pregnancy Complications | 1.04 | 0.89 | 1.04 | 0.34 | 321318 | 1336 | 319982 | FALSE |
| 536.8 | Dyspepsia and other specified disorders of function of stomach | Digestive | 1.09 | 0.76 | 1.10 | 0.34 | 296775 | 247 | 296528 | FALSE |
| 512.8 | Cough | Respiratory | 0.97 | 0.97 | 1.08 | 0.34 | 317249 | 2964 | 314285 | FALSE |
| 907 | Injuries to the nervous system | Injuries & Poisonings | 1.03 | 0.91 | 1.04 | 0.34 | 328257 | 1845 | 326412 | FALSE |
| 377 | Disorders of optic nerve and visual pathways | Sense Organs | 1.07 | 0.82 | 1.07 | 0.35 | 288645 | 436 | 288209 | FALSE |
| 530 | Diseases of esophagus | Digestive | 0.95 | 0.95 | 1.16 | 0.35 | 288621 | 801 | 287820 | FALSE |
| 202.2 | Non-Hodgkins lymphoma | Neoplasms | 1.02 | 0.93 | 1.03 | 0.35 | 327072 | 3532 | 323540 | FALSE |
| 574.1 | Cholelithiasis | Digestive | 0.99 | 0.98 | 1.05 | 0.35 | 319646 | 9306 | 310340 | FALSE |
| 755.1 | Congenital deformities of feet | Congenital Anomalies | 1.10 | 0.75 | 1.11 | 0.35 | 327548 | 220 | 327328 | FALSE |
| 520 | Disorders of tooth development | Digestive | 1.07 | 0.82 | 1.07 | 0.35 | 312145 | 470 | 311675 | FALSE |
| 870 | Open wounds of head; neck; and trunk | Injuries & Poisonings | 1.06 | 0.84 | 1.06 | 0.35 | 320344 | 596 | 319748 | FALSE |
| 556 | Ulceration of the lower GI tract | Digestive | 0.94 | 0.93 | 1.23 | 0.35 | 258853 | 440 | 258413 | FALSE |
| 78 | Viral warts & HPV | Infectious Diseases | 1.04 | 0.88 | 1.05 | 0.35 | 323235 | 1012 | 322223 | FALSE |
| 743.9 | Osteopenia or other disorder of bone and cartilage | Musculoskeletal | 1.02 | 0.93 | 1.02 | 0.35 | 328205 | 3989 | 324216 | FALSE |
| 605 | Erectile dysfunction [ED] | Genitourinary | 0.92 | 0.91 | 1.29 | 0.35 | 308214 | 291 | 307923 | FALSE |
| 411.41 | Aneurysm and dissection of heart | Circulatory System | 0.95 | 0.94 | 1.17 | 0.35 | 296427 | 721 | 295706 | FALSE |
| 41.1 | Staphylococcus infections | Infectious Diseases | 1.02 | 0.93 | 1.03 | 0.35 | 315849 | 3200 | 312649 | FALSE |
| 345.1 | Epilepsy | Neurological | 1.08 | 0.79 | 1.09 | 0.36 | 286500 | 331 | 286169 | FALSE |
| 960.2 | Allergy/adverse effect of penicillin | Injuries & Poisonings | 1.01 | 0.97 | 1.01 | 0.36 | 316911 | 16683 | 300228 | FALSE |
| 618.5 | Prolapse of vaginal vault after hysterectomy | Genitourinary | 1.06 | 0.83 | 1.07 | 0.36 | 316760 | 501 | 316259 | FALSE |
| 54 | Herpes simplex | Infectious Diseases | 1.09 | 0.75 | 1.11 | 0.36 | 322442 | 219 | 322223 | FALSE |
| 535.6 | Duodenitis | Digestive | 1.02 | 0.95 | 1.02 | 0.36 | 304422 | 7894 | 296528 | FALSE |
| 854 | Complications of cardiac/vascular device, implant, and graft | Injuries & Poisonings | 0.97 | 0.96 | 1.10 | 0.36 | 315824 | 1843 | 313981 | FALSE |

| | | | | | | | | | | |
|---|---|---|---|---|---|---|---|---|---|---|
| 627 | Menopausal and postmenopausal disorders | Genitourinary | 0.95 | 0.95 | 1.16 | 0.36 | 297095 | 847 | 296248 | FALSE |
| 686.1 | Carbuncle and furuncle | Dermatologic | 0.97 | 0.97 | 1.09 | 0.36 | 318846 | 2422 | 316424 | FALSE |
| 618.6 | Vaginal enterocele, congenital or acquired | Genitourinary | 1.05 | 0.85 | 1.06 | 0.37 | 316926 | 667 | 316259 | FALSE |
| 250.42 | Other abnormal glucose | Endocrine/Metabolic | 1.06 | 0.82 | 1.08 | 0.37 | 307944 | 437 | 307507 | FALSE |
| 626.2 | Dysmenorrhea | Genitourinary | 0.97 | 0.96 | 1.11 | 0.37 | 297927 | 1679 | 296248 | FALSE |
| 592.13 | Chronic interstitial cystitis | Genitourinary | 0.92 | 0.91 | 1.31 | 0.37 | 297306 | 254 | 297052 | FALSE |
| 342 | Hemiplegia | Neurological | 0.97 | 0.96 | 1.11 | 0.37 | 287694 | 1525 | 286169 | FALSE |
| 524.3 | Anomalies of tooth position/malocclusion | Digestive | 1.08 | 0.78 | 1.10 | 0.37 | 311970 | 295 | 311675 | FALSE |
| 529 | Diseases and other conditions of the tongue | Digestive | 0.96 | 0.95 | 1.15 | 0.37 | 323502 | 951 | 322551 | FALSE |
| 608 | Other disorders of male genital organs | Genitourinary | 1.01 | 0.97 | 1.01 | 0.38 | 328015 | 20092 | 307923 | FALSE |
| 872 | Traumatic amputation | Injuries & Poisonings | 0.94 | 0.93 | 1.21 | 0.38 | 320230 | 482 | 319748 | FALSE |
| 198.2 | Secondary malignancy of respiratory organs | Neoplasms | 0.97 | 0.97 | 1.09 | 0.38 | 231606 | 2277 | 229329 | FALSE |
| 512 | Other symptoms of respiratory system | Respiratory | 1.02 | 0.94 | 1.02 | 0.38 | 319789 | 5504 | 314285 | FALSE |
| 117 | Mycoses | Infectious Diseases | 0.98 | 0.97 | 1.08 | 0.38 | 328252 | 2735 | 325517 | FALSE |
| 241.1 | Nontoxic uninodular goiter | Endocrine/Metabolic | 0.94 | 0.93 | 1.20 | 0.38 | 310857 | 511 | 310346 | FALSE |
| 333.1 | Essential tremor | Neurological | 1.09 | 0.76 | 1.11 | 0.38 | 286387 | 218 | 286169 | FALSE |
| 426.3 | Bundle branch block | Circulatory System | 1.08 | 0.78 | 1.10 | 0.38 | 299829 | 291 | 299538 | FALSE |
| 394.2 | Mitral valve disease | Circulatory System | 0.98 | 0.97 | 1.08 | 0.38 | 324620 | 3075 | 321545 | FALSE |
| 303.3 | Psychogenic disorder | Mental Disorders | 1.04 | 0.87 | 1.06 | 0.38 | 283756 | 841 | 282915 | FALSE |
| 565.1 | Anal and rectal polyp | Digestive | 1.01 | 0.95 | 1.02 | 0.38 | 254346 | 7669 | 246677 | FALSE |
| 384 | Other disorders of tympanic membrane | Sense Organs | 1.06 | 0.82 | 1.08 | 0.39 | 324590 | 430 | 324160 | FALSE |
| 540.1 | Appendicitis | Digestive | 0.96 | 0.94 | 1.16 | 0.39 | 325191 | 795 | 324396 | FALSE |
| 573.3 | Hepatomegaly | Digestive | 0.93 | 0.91 | 1.27 | 0.39 | 318658 | 310 | 318348 | FALSE |
| 530.9 | Heartburn | Digestive | 1.03 | 0.91 | 1.04 | 0.39 | 289928 | 2108 | 287820 | FALSE |
| 366 | Cataract | Sense Organs | 0.99 | 0.99 | 1.03 | 0.39 | 323842 | 16441 | 307401 | FALSE |
| 274.21 | Chondrocalcinosis | Endocrine/Metabolic | 0.93 | 0.91 | 1.28 | 0.39 | 326569 | 290 | 326279 | FALSE |
| 427.3 | Other specified cardiac dysrhythmias | Circulatory System | 0.98 | 0.97 | 1.08 | 0.39 | 302853 | 3315 | 299538 | FALSE |
| 701.2 | Scar conditions and fibrosis of skin | Dermatologic | 0.97 | 0.97 | 1.09 | 0.39 | 325488 | 2407 | 323081 | FALSE |
| 473.3 | Paralysis/spasm of vocal cords or larynx | Respiratory | 0.93 | 0.90 | 1.29 | 0.40 | 309234 | 267 | 308967 | FALSE |
| 624.1 | Dystrophy of female genital tract | Genitourinary | 1.08 | 0.77 | 1.11 | 0.40 | 318926 | 240 | 318686 | FALSE |
| 727.5 | Rupture of synovium | Musculoskeletal | 0.93 | 0.91 | 1.25 | 0.40 | 305315 | 344 | 304971 | FALSE |
| 614 | Inflammatory diseases of female pelvic organs | Genitourinary | 1.07 | 0.81 | 1.09 | 0.40 | 319190 | 374 | 318816 | FALSE |
| 727.4 | Ganglion and cyst of synovium, tendon, and bursa | Musculoskeletal | 0.98 | 0.97 | 1.08 | 0.40 | 307953 | 2982 | 304971 | FALSE |
| 579.2 | Splenomegaly | Digestive | 0.94 | 0.92 | 1.22 | 0.40 | 303854 | 443 | 303411 | FALSE |
| 596.5 | Functional disorders of bladder | Genitourinary | 1.03 | 0.90 | 1.04 | 0.40 | 315335 | 1682 | 313653 | FALSE |
| 212 | Benign neoplasm of respiratory and intrathoracic organs | Neoplasms | 1.07 | 0.80 | 1.09 | 0.40 | 328257 | 355 | 327902 | FALSE |
| 388 | Other disorders of ear | Sense Organs | 0.99 | 0.98 | 1.04 | 0.40 | 328097 | 9886 | 318211 | FALSE |
| 555.21 | Ulcerative colitis (chronic) | Digestive | 0.95 | 0.93 | 1.19 | 0.40 | 258984 | 571 | 258413 | FALSE |

| | | | | | | | | | | | |
|---|---|---|---|---|---|---|---|---|---|---|---|
| 989 | Toxic effect of other substances, chiefly nonmedicinal as to source | Injuries & Poisonings | 1.03 | 0.90 | 1.04 | 0.40 | 328153 | 1438 | 326715 | FALSE |
| 379.3 | Aphakia and other disorders of lens | Sense Organs | 0.99 | 0.99 | 1.03 | 0.40 | 308937 | 20728 | 288209 | FALSE |
| 41.2 | Streptococcus infection | Infectious Diseases | 1.03 | 0.90 | 1.04 | 0.40 | 314297 | 1648 | 312649 | FALSE |
| 535.8 | Other specified gastritis | Digestive | 0.99 | 0.98 | 1.05 | 0.41 | 304943 | 8415 | 296528 | FALSE |
| 369 | Infection of the eye | Sense Organs | 1.08 | 0.77 | 1.11 | 0.41 | 318659 | 254 | 318405 | FALSE |
| 571.5 | Other chronic nonalcoholic liver disease | Digestive | 0.97 | 0.96 | 1.11 | 0.41 | 320021 | 1673 | 318348 | FALSE |
| 286.7 | Other and unspecified coagulation defects | Hematopoietic | 0.94 | 0.92 | 1.23 | 0.41 | 325919 | 405 | 325514 | FALSE |
| 512.1 | Wheezing | Respiratory | 0.92 | 0.89 | 1.31 | 0.41 | 314516 | 231 | 314285 | FALSE |
| 497 | Bronchitis | Respiratory | 0.95 | 0.94 | 1.18 | 0.41 | 294851 | 642 | 294209 | FALSE |
| 389.1 | Sensorineural hearing loss | Sense Organs | 0.95 | 0.92 | 1.21 | 0.41 | 318675 | 464 | 318211 | FALSE |
| 736.2 | Acquired deformities of finger | Musculoskeletal | 0.93 | 0.90 | 1.30 | 0.41 | 316957 | 253 | 316704 | FALSE |
| 175 | Acquired absence of breast | Neoplasms | 1.02 | 0.92 | 1.03 | 0.41 | 305924 | 2516 | 303408 | FALSE |
| 287.3 | Thrombocytopenia | Hematopoietic | 0.97 | 0.95 | 1.12 | 0.42 | 326785 | 1271 | 325514 | FALSE |
| 395.1 | Nonrheumatic mitral valve disorders | Circulatory System | 0.98 | 0.97 | 1.08 | 0.42 | 324514 | 2969 | 321545 | FALSE |
| 426.23 | Second degree AV block | Circulatory System | 1.06 | 0.83 | 1.08 | 0.42 | 299999 | 461 | 299538 | FALSE |
| 367.1 | Myopia | Sense Organs | 0.97 | 0.95 | 1.12 | 0.42 | 327042 | 1271 | 325771 | FALSE |
| 145.2 | Cancer of tongue | Neoplasms | 0.94 | 0.91 | 1.26 | 0.42 | 326383 | 325 | 326058 | FALSE |
| 443.1 | Raynaud's syndrome | Circulatory System | 1.03 | 0.89 | 1.05 | 0.42 | 320542 | 1165 | 319377 | FALSE |
| 535 | Gastritis and duodenitis | Digestive | 0.99 | 0.99 | 1.03 | 0.43 | 314276 | 17748 | 296528 | FALSE |
| 414 | Other forms of chronic heart disease | Circulatory System | 0.97 | 0.96 | 1.10 | 0.43 | 297474 | 1768 | 295706 | FALSE |
| 536 | Disorders of function of stomach | Digestive | 1.07 | 0.78 | 1.11 | 0.43 | 296785 | 257 | 296528 | FALSE |
| 614.32 | Chronic inflammatory pelvic disease | Genitourinary | 0.94 | 0.92 | 1.22 | 0.43 | 319241 | 425 | 318816 | FALSE |
| 574 | Cholelithiasis and cholecystitis | Digestive | 1.06 | 0.82 | 1.09 | 0.43 | 310743 | 403 | 310340 | FALSE |
| 296 | Mood disorders | Mental Disorders | 1.01 | 0.96 | 1.02 | 0.43 | 295870 | 12955 | 282915 | FALSE |
| 594.8 | Renal colic | Genitourinary | 1.03 | 0.92 | 1.04 | 0.43 | 322079 | 2131 | 319948 | FALSE |
| 242 | Thyrotoxicosis with or without goiter | Endocrine/Metabolic | 1.03 | 0.90 | 1.05 | 0.44 | 311811 | 1465 | 310346 | FALSE |
| 352.2 | Facial nerve disorders [CN7] | Neurological | 1.04 | 0.87 | 1.06 | 0.44 | 313928 | 900 | 313028 | FALSE |
| 495.2 | Asthma with exacerbation | Respiratory | 0.93 | 0.90 | 1.29 | 0.44 | 294470 | 261 | 294209 | FALSE |
| 256.4 | Polycystic ovaries | Endocrine/Metabolic | 0.92 | 0.89 | 1.32 | 0.44 | 324781 | 219 | 324562 | FALSE |
| 555.2 | Ulcerative colitis | Digestive | 0.98 | 0.97 | 1.07 | 0.44 | 261567 | 3154 | 258413 | FALSE |
| 495 | Asthma | Respiratory | 0.99 | 0.99 | 1.03 | 0.44 | 320988 | 26779 | 294209 | FALSE |
| 557.1 | Celiac disease | Digestive | 1.03 | 0.91 | 1.04 | 0.44 | 260307 | 1894 | 258413 | FALSE |
| 204.4 | Multiple myeloma | Neoplasms | 1.05 | 0.85 | 1.08 | 0.44 | 324114 | 574 | 323540 | FALSE |
| 448 | Disease of capillaries | Circulatory System | 1.01 | 0.95 | 1.02 | 0.44 | 326858 | 7481 | 319377 | FALSE |
| 798.1 | Chronic fatigue syndrome | Symptoms | 1.05 | 0.85 | 1.07 | 0.44 | 325352 | 606 | 324746 | FALSE |
| 427 | Cardiac dysrhythmias | Circulatory System | 0.94 | 0.91 | 1.25 | 0.44 | 299861 | 323 | 299538 | FALSE |
| 782.3 | Edema | Symptoms | 1.03 | 0.91 | 1.04 | 0.44 | 328084 | 1715 | 326369 | FALSE |
| 364 | Corneal opacity and other disorders of cornea | Sense Organs | 1.08 | 0.77 | 1.12 | 0.45 | 317026 | 227 | 316799 | FALSE |

| | | | | | | | | | | | |
|---|---|---|---|---|---|---|---|---|---|---|---|
| 613.8 | Other specified disorders of breast | Genitourinary | 1.06 | 0.81 | 1.10 | 0.45 | 322790 | 355 | 322435 | FALSE |
| 218.1 | Uterine leiomyoma | Neoplasms | 0.99 | 0.98 | 1.04 | 0.45 | 318363 | 10566 | 307797 | FALSE |
| 716.2 | Unspecified monoarthritis | Musculoskeletal | 1.01 | 0.97 | 1.01 | 0.45 | 289814 | 15901 | 273913 | FALSE |
| 442.8 | Aneurysm of other specified artery | Circulatory System | 0.93 | 0.88 | 1.32 | 0.45 | 319587 | 210 | 319377 | FALSE |
| 507 | Pleurisy; pleural effusion | Respiratory | 1.01 | 0.95 | 1.02 | 0.45 | 323108 | 6607 | 316501 | FALSE |
| 296.2 | Depression | Mental Disorders | 1.01 | 0.96 | 1.02 | 0.45 | 295060 | 12145 | 282915 | FALSE |
| 574.11 | Cholelithiasis with acute cholecystitis | Digestive | 1.03 | 0.90 | 1.05 | 0.45 | 311853 | 1513 | 310340 | FALSE |
| 189.11 | Malignant neoplasm of kidney, except pelvis | Neoplasms | 0.97 | 0.95 | 1.13 | 0.45 | 325014 | 1035 | 323979 | FALSE |
| 611.3 | Lump or mass in breast | Genitourinary | 0.97 | 0.96 | 1.11 | 0.45 | 322478 | 1588 | 320890 | FALSE |
| 476 | Allergic rhinitis | Respiratory | 0.97 | 0.95 | 1.13 | 0.45 | 310042 | 1075 | 308967 | FALSE |
| 198.5 | Secondary malignancy of brain/spine | Neoplasms | 0.96 | 0.94 | 1.15 | 0.45 | 230130 | 801 | 229329 | FALSE |
| 726.1 | Enthesopathy | Musculoskeletal | 1.01 | 0.95 | 1.02 | 0.46 | 311674 | 6703 | 304971 | FALSE |
| 180.3 | Cervical intraepithelial neoplasia [CIN] [Cervical dysplasia] | Neoplasms | 1.02 | 0.92 | 1.04 | 0.46 | 299753 | 2153 | 297600 | FALSE |
| 274.1 | Gout | Endocrine/Metabolic | 1.03 | 0.91 | 1.04 | 0.46 | 327947 | 1668 | 326279 | FALSE |
| 801.1 | Fracture of foot | Injuries & Poisonings | 0.95 | 0.92 | 1.22 | 0.46 | 323992 | 418 | 323574 | FALSE |
| 571.81 | Portal hypertension | Digestive | 1.05 | 0.85 | 1.08 | 0.46 | 318917 | 569 | 318348 | FALSE |
| 378.2 | Nystagmus and other irregular eye movements | Sense Organs | 0.93 | 0.88 | 1.31 | 0.46 | 288424 | 215 | 288209 | FALSE |
| 587 | Kidney replaced by transpant | Genitourinary | 0.95 | 0.91 | 1.22 | 0.46 | 314494 | 398 | 314096 | FALSE |
| 521.1 | Dental caries | Digestive | 1.02 | 0.93 | 1.03 | 0.46 | 314929 | 3254 | 311675 | FALSE |
| 798 | Malaise and fatigue | Symptoms | 1.02 | 0.93 | 1.03 | 0.46 | 327712 | 2966 | 324746 | FALSE |
| 331.9 | Cerebral degeneration, unspecified | Neurological | 1.05 | 0.82 | 1.09 | 0.47 | 286586 | 417 | 286169 | FALSE |
| 433.21 | Cerebral artery occlusion, with cerebral infarction | Circulatory System | 0.97 | 0.95 | 1.11 | 0.47 | 319658 | 1534 | 318124 | FALSE |
| 840 | Sprains and strains | Injuries & Poisonings | 1.05 | 0.84 | 1.09 | 0.47 | 328218 | 481 | 327737 | FALSE |
| 375 | Disorders of lacrimal system | Sense Organs | 0.97 | 0.96 | 1.10 | 0.47 | 289869 | 1660 | 288209 | FALSE |
| 443.9 | Peripheral vascular disease, unspecified | Circulatory System | 0.98 | 0.96 | 1.08 | 0.47 | 321986 | 2609 | 319377 | FALSE |
| 216 | Benign neoplasm of skin | Neoplasms | 0.99 | 0.98 | 1.05 | 0.47 | 327631 | 7865 | 319766 | FALSE |
| 614.1 | Pelvic peritoneal adhesions, female (postoperative) (postinfection) | Genitourinary | 1.02 | 0.92 | 1.04 | 0.47 | 321387 | 2571 | 318816 | FALSE |
| 619.4 | Noninflammatory disorders of vagina | Genitourinary | 1.03 | 0.91 | 1.04 | 0.48 | 280345 | 1780 | 278565 | FALSE |
| 800 | Fracture of lower limb | Injuries & Poisonings | 1.04 | 0.86 | 1.07 | 0.48 | 324273 | 699 | 323574 | FALSE |
| 149.1 | Cancer of oropharynx | Neoplasms | 0.94 | 0.89 | 1.28 | 0.48 | 326314 | 256 | 326058 | FALSE |
| 540 | Appendiceal conditions | Digestive | 0.98 | 0.97 | 1.07 | 0.48 | 328257 | 3861 | 324396 | FALSE |
| 198.4 | Secondary malignant neoplasm of liver | Neoplasms | 0.98 | 0.96 | 1.08 | 0.48 | 232026 | 2697 | 229329 | FALSE |
| 425.1 | Primary/intrinsic cardiomyopathies | Circulatory System | 1.03 | 0.89 | 1.06 | 0.48 | 326053 | 1053 | 325000 | FALSE |
| 625.1 | Dyspareunia | Genitourinary | 1.03 | 0.89 | 1.06 | 0.48 | 319841 | 1155 | 318686 | FALSE |
| 600 | Hyperplasia of prostate | Genitourinary | 1.01 | 0.96 | 1.02 | 0.48 | 318982 | 11572 | 307410 | FALSE |
| 149.4 | Cancer of larynx | Neoplasms | 0.94 | 0.89 | 1.27 | 0.48 | 326324 | 266 | 326058 | FALSE |
| 426.9 | Cardiac pacemaker/device in situ | Circulatory System | 1.07 | 0.78 | 1.13 | 0.49 | 299775 | 237 | 299538 | FALSE |
| 627.4 | Premenopausal menorrhagia | Genitourinary | 1.06 | 0.81 | 1.11 | 0.49 | 296580 | 332 | 296248 | FALSE |

| | | | | | | | | | | |
|---|---|---|---|---|---|---|---|---|---|---|
| 729.1 | Rheumatism, unspecified and fibrositis | Musculoskeletal | 1.05 | 0.82 | 1.10 | 0.49 | 305358 | 387 | 304971 | FALSE |
| 277 | Other disorders of metabolism | Endocrine/Metabolic | 1.01 | 0.98 | 1.01 | 0.49 | 328247 | 45303 | 282944 | FALSE |
| 306 | Other mental disorder | Mental Disorders | 0.99 | 0.99 | 1.02 | 0.49 | 312320 | 29405 | 282915 | FALSE |
| 275.5 | Disorders of calcium/phosphorus metabolism | Endocrine/Metabolic | 0.97 | 0.94 | 1.12 | 0.49 | 327161 | 1109 | 326052 | FALSE |
| 368.2 | Diplopia and disorders of binocular vision | Sense Organs | 0.96 | 0.93 | 1.15 | 0.49 | 325652 | 753 | 324899 | FALSE |
| 681 | Superficial cellulitis and abscess | Dermatologic | 1.04 | 0.87 | 1.07 | 0.50 | 317152 | 728 | 316424 | FALSE |
| 850 | Hemorrhage or hematoma complicating a procedure | Injuries & Poisonings | 0.99 | 0.97 | 1.05 | 0.50 | 319345 | 5364 | 313981 | FALSE |
| 433.31 | Transient cerebral ischemia | Circulatory System | 1.02 | 0.92 | 1.04 | 0.50 | 320298 | 2174 | 318124 | FALSE |
| 401.22 | Hypertensive chronic kidney disease | Circulatory System | 0.98 | 0.95 | 1.10 | 0.50 | 250360 | 1595 | 248765 | FALSE |
| 614.4 | Inflammatory diseases of uterus, except cervix | Genitourinary | 1.06 | 0.80 | 1.11 | 0.50 | 319124 | 308 | 318816 | FALSE |
| 750 | Digestive congenital anomalies | Congenital Anomalies | 0.96 | 0.93 | 1.16 | 0.50 | 326722 | 702 | 326020 | FALSE |
| 618.1 | Prolapse of vaginal walls | Genitourinary | 0.99 | 0.98 | 1.05 | 0.50 | 323743 | 7484 | 316259 | FALSE |
| 334.2 | Anterior horn cell disease | Neurological | 1.07 | 0.77 | 1.13 | 0.50 | 286393 | 224 | 286169 | FALSE |
| 443.7 | Peripheral angiopathy in diseases classified elsewhere | Circulatory System | 1.05 | 0.83 | 1.10 | 0.51 | 319792 | 415 | 319377 | FALSE |
| 242.1 | Graves' disease | Endocrine/Metabolic | 0.96 | 0.91 | 1.20 | 0.51 | 310806 | 460 | 310346 | FALSE |
| 990 | Effects radiation NOS | Injuries & Poisonings | 1.02 | 0.93 | 1.03 | 0.51 | 325650 | 3244 | 322406 | FALSE |
| 560.3 | Peritoneal or intestinal adhesions | Digestive | 0.97 | 0.94 | 1.14 | 0.51 | 259274 | 861 | 258413 | FALSE |
| 590 | Pyelonephritis | Genitourinary | 1.02 | 0.94 | 1.03 | 0.51 | 300977 | 3925 | 297052 | FALSE |
| 281.13 | Folate-deficiency anemia | Hematopoietic | 1.07 | 0.76 | 1.14 | 0.51 | 309118 | 201 | 308917 | FALSE |
| 565 | Anal and rectal conditions | Digestive | 0.99 | 0.98 | 1.04 | 0.51 | 255338 | 8661 | 246677 | FALSE |
| 371.3 | Inflammation of eyelids | Sense Organs | 0.98 | 0.96 | 1.08 | 0.51 | 320803 | 2398 | 318405 | FALSE |
| 687.1 | Rash and other nonspecific skin eruption | Dermatologic | 0.98 | 0.96 | 1.09 | 0.52 | 325049 | 2218 | 322831 | FALSE |
| 550.4 | Umbilical hernia | Digestive | 0.98 | 0.97 | 1.06 | 0.52 | 283710 | 3904 | 279806 | FALSE |
| 575.7 | Other disorders of gallbladder | Digestive | 1.03 | 0.90 | 1.05 | 0.52 | 311736 | 1396 | 310340 | FALSE |
| 389.4 | Tinnitus | Sense Organs | 1.04 | 0.85 | 1.09 | 0.52 | 318752 | 541 | 318211 | FALSE |
| 801 | Fracture of ankle and foot | Injuries & Poisonings | 0.94 | 0.88 | 1.28 | 0.52 | 323825 | 251 | 323574 | FALSE |
| 701.4 | Keloid scar | Dermatologic | 0.94 | 0.88 | 1.29 | 0.52 | 323309 | 228 | 323081 | FALSE |
| 681.2 | Cellulitis and abscess of face/neck | Dermatologic | 1.04 | 0.85 | 1.09 | 0.52 | 316953 | 529 | 316424 | FALSE |
| 333.4 | Torsion dystonia | Neurological | 0.94 | 0.89 | 1.27 | 0.52 | 286434 | 265 | 286169 | FALSE |
| 474 | Acute and chronic tonsillitis | Respiratory | 0.96 | 0.92 | 1.17 | 0.52 | 309571 | 604 | 308967 | FALSE |
| 735 | Acquired foot deformities | Musculoskeletal | 0.95 | 0.89 | 1.25 | 0.53 | 317003 | 299 | 316704 | FALSE |
| 577.3 | Cyst and pseudocyst of pancreas | Digestive | 1.05 | 0.82 | 1.11 | 0.53 | 326312 | 375 | 325937 | FALSE |
| 38 | Septicemia | Infectious Diseases | 1.02 | 0.93 | 1.04 | 0.53 | 315694 | 3045 | 312649 | FALSE |
| 381.1 | Otitis media | Sense Organs | 0.97 | 0.94 | 1.12 | 0.53 | 325313 | 1153 | 324160 | FALSE |
| 579.8 | Nonspecific abnormal findings in stool contents | Digestive | 0.98 | 0.95 | 1.10 | 0.53 | 305151 | 1740 | 303411 | FALSE |
| 531.4 | Peptic ulcer, site unspecified | Digestive | 1.04 | 0.84 | 1.10 | 0.53 | 321015 | 449 | 320566 | FALSE |
| 738.4 | Acquired spondylolisthesis | Musculoskeletal | 0.94 | 0.88 | 1.27 | 0.53 | 316959 | 255 | 316704 | FALSE |
| 386 | Vertiginous syndromes and other disorders of vestibular system | Sense Organs | 0.98 | 0.96 | 1.09 | 0.53 | 323778 | 2112 | 321666 | FALSE |

| | | | | | | | | | | |
|---|---|---|---|---|---|---|---|---|---|---|
| 803 | Fracture of upper limb | Injuries & Poisonings | 1.04 | 0.83 | 1.10 | 0.53 | 324007 | 433 | 323574 | FALSE |
| 276.14 | Hypopotassemia | Endocrine/Metabolic | 0.98 | 0.95 | 1.10 | 0.54 | 322132 | 1471 | 320661 | FALSE |
| 599.4 | Urinary incontinence | Genitourinary | 1.01 | 0.96 | 1.02 | 0.54 | 229440 | 8996 | 220444 | FALSE |
| 745 | Pain in joint | Musculoskeletal | 1.02 | 0.92 | 1.05 | 0.54 | 328257 | 1940 | 326317 | FALSE |
| 427.12 | Paroxysmal ventricular tachycardia | Circulatory System | 1.03 | 0.89 | 1.07 | 0.54 | 300507 | 969 | 299538 | FALSE |
| 333 | Extrapyramidal disease and abnormal movement disorders | Neurological | 0.98 | 0.96 | 1.09 | 0.54 | 288204 | 2035 | 286169 | FALSE |
| 585.3 | Chronic renal failure [CKD] | Genitourinary | 0.98 | 0.96 | 1.08 | 0.54 | 316681 | 2585 | 314096 | FALSE |
| 427.6 | Premature beats | Circulatory System | 1.04 | 0.84 | 1.10 | 0.54 | 299996 | 458 | 299538 | FALSE |
| 375.2 | Epiphora | Sense Organs | 1.03 | 0.88 | 1.07 | 0.54 | 289111 | 902 | 288209 | FALSE |
| 70.9 | Hepatitis NOS | Infectious Diseases | 1.04 | 0.84 | 1.10 | 0.55 | 322675 | 452 | 322223 | FALSE |
| 689 | Disorder of skin and subcutaneous tissue NOS | Dermatologic | 1.00 | 0.99 | 1.02 | 0.55 | 328257 | 42194 | 286063 | FALSE |
| 701.5 | Abnormal granulation tissue | Dermatologic | 1.04 | 0.84 | 1.10 | 0.55 | 323560 | 479 | 323081 | FALSE |
| 569 | Other disorders of intestine | Digestive | 1.00 | 0.98 | 1.01 | 0.55 | 326165 | 79488 | 246677 | FALSE |
| 429.3 | Symptoms involving cardiovascular system | Circulatory System | 0.94 | 0.87 | 1.30 | 0.55 | 322258 | 208 | 322050 | FALSE |
| 531.3 | Duodenal ulcer | Digestive | 0.98 | 0.96 | 1.07 | 0.55 | 323661 | 3095 | 320566 | FALSE |
| 512.7 | Shortness of breath | Respiratory | 0.99 | 0.97 | 1.05 | 0.55 | 320401 | 6116 | 314285 | FALSE |
| 150 | Cancer of esophagus | Neoplasms | 1.03 | 0.87 | 1.08 | 0.55 | 313075 | 727 | 312348 | FALSE |
| 374.1 | Ectropion or entropion | Sense Organs | 0.97 | 0.94 | 1.12 | 0.55 | 319497 | 1092 | 318405 | FALSE |
| 726.3 | Bursitis | Musculoskeletal | 1.04 | 0.85 | 1.09 | 0.55 | 305514 | 543 | 304971 | FALSE |
| 41 | Bacterial infection NOS | Infectious Diseases | 0.99 | 0.98 | 1.04 | 0.55 | 323401 | 10752 | 312649 | FALSE |
| 550.1 | Inguinal hernia | Digestive | 0.99 | 0.98 | 1.03 | 0.55 | 296191 | 16385 | 279806 | FALSE |
| 450 | Noninfectious disorders of lymphatic channels | Circulatory System | 0.97 | 0.93 | 1.15 | 0.55 | 328257 | 717 | 327540 | FALSE |
| 226 | Benign neoplasm of thyroid glands | Neoplasms | 0.95 | 0.88 | 1.26 | 0.55 | 326925 | 266 | 326659 | FALSE |
| 320 | Meningitis | Neurological | 0.96 | 0.91 | 1.20 | 0.55 | 327594 | 439 | 327155 | FALSE |
| 446.9 | Arteritis NOS | Circulatory System | 1.06 | 0.79 | 1.14 | 0.56 | 319622 | 245 | 319377 | FALSE |
| 594.3 | Calculus of ureter | Genitourinary | 1.02 | 0.93 | 1.04 | 0.56 | 322488 | 2540 | 319948 | FALSE |
| 208 | Benign neoplasm of colon | Neoplasms | 0.99 | 0.99 | 1.03 | 0.56 | 325454 | 20827 | 304627 | FALSE |
| 529.1 | Glossitis | Digestive | 0.95 | 0.89 | 1.24 | 0.56 | 322855 | 304 | 322551 | FALSE |
| 728.7 | Fasciitis | Musculoskeletal | 0.94 | 0.87 | 1.30 | 0.56 | 305184 | 213 | 304971 | FALSE |
| 317 | Alcohol-related disorders | Mental Disorders | 1.01 | 0.95 | 1.03 | 0.56 | 303546 | 5860 | 297686 | FALSE |
| 440 | Atherosclerosis | Circulatory System | 0.94 | 0.87 | 1.30 | 0.56 | 319581 | 204 | 319377 | FALSE |
| 426.4 | Anomalous atrioventricular excitation | Circulatory System | 0.95 | 0.88 | 1.28 | 0.56 | 299778 | 240 | 299538 | FALSE |
| 626.14 | Irregular menstrual bleeding | Genitourinary | 1.01 | 0.94 | 1.03 | 0.57 | 300194 | 3946 | 296248 | FALSE |
| 411.8 | Other chronic ischemic heart disease, unspecified | Circulatory System | 1.01 | 0.98 | 1.01 | 0.57 | 326986 | 31280 | 295706 | FALSE |
| 287.31 | Primary thrombocytopenia | Hematopoietic | 1.04 | 0.84 | 1.10 | 0.57 | 325942 | 428 | 325514 | FALSE |
| 348.7 | Coma | Neurological | 1.04 | 0.83 | 1.11 | 0.57 | 286548 | 379 | 286169 | FALSE |
| 592.12 | Chronic cystitis | Genitourinary | 0.97 | 0.93 | 1.13 | 0.57 | 297965 | 913 | 297052 | FALSE |
| 702.1 | Actinic keratosis | Dermatologic | 1.02 | 0.93 | 1.04 | 0.57 | 325281 | 2664 | 322617 | FALSE |

| | | | | | | | | | | | |
|---|---|---|---|---|---|---|---|---|---|---|---|
| 382 | Otalgia | Sense Organs | 0.95 | 0.89 | 1.23 | 0.58 | 324475 | 315 | 324160 | FALSE |
| 214 | Lipoma | Neoplasms | 1.02 | 0.92 | 1.05 | 0.58 | 322512 | 1819 | 320693 | FALSE |
| 830 | Dislocation | Injuries & Poisonings | 0.98 | 0.96 | 1.08 | 0.58 | 323944 | 2154 | 321790 | FALSE |
| 564 | Functional digestive disorders | Digestive | 0.97 | 0.94 | 1.13 | 0.58 | 259396 | 983 | 258413 | FALSE |
| 198.3 | Secondary malignant neoplasm of digestive systems | Neoplasms | 0.98 | 0.95 | 1.10 | 0.58 | 230894 | 1565 | 229329 | FALSE |
| 635.2 | Antepartum hemorrhage, abruptio placentae, and placenta previa | Pregnancy Complications | 1.03 | 0.88 | 1.07 | 0.58 | 320918 | 936 | 319982 | FALSE |
| 427.11 | Paroxysmal supraventricular tachycardia | Circulatory System | 1.02 | 0.93 | 1.04 | 0.58 | 301969 | 2431 | 299538 | FALSE |
| 687 | Symptoms affecting skin | Dermatologic | 0.95 | 0.87 | 1.29 | 0.58 | 323049 | 218 | 322831 | FALSE |
| 706.2 | Sebaceous cyst | Dermatologic | 1.01 | 0.96 | 1.02 | 0.59 | 327418 | 9107 | 318311 | FALSE |
| 365.11 | Primary open angle glaucoma | Sense Organs | 1.02 | 0.89 | 1.07 | 0.59 | 317855 | 1056 | 316799 | FALSE |
| 961 | Poisoning by other anti-infectives | Injuries & Poisonings | 0.96 | 0.90 | 1.19 | 0.59 | 300664 | 436 | 300228 | FALSE |
| 345.11 | Generalized convulsive epilepsy | Neurological | 0.96 | 0.91 | 1.19 | 0.59 | 286634 | 465 | 286169 | FALSE |
| 836 | Traumatic arthropathy | Injuries & Poisonings | 1.05 | 0.79 | 1.14 | 0.59 | 322033 | 243 | 321790 | FALSE |
| 276.6 | Fluid overload | Endocrine/Metabolic | 0.96 | 0.91 | 1.19 | 0.59 | 321134 | 473 | 320661 | FALSE |
| 736 | Other acquired deformities of limbs | Musculoskeletal | 1.05 | 0.81 | 1.13 | 0.59 | 317015 | 311 | 316704 | FALSE |
| 411.4 | Coronary atherosclerosis | Circulatory System | 1.00 | 0.98 | 1.01 | 0.59 | 327259 | 31553 | 295706 | FALSE |
| 531.1 | Hemorrhage from gastrointestinal ulcer | Digestive | 0.97 | 0.92 | 1.16 | 0.59 | 321221 | 655 | 320566 | FALSE |
| 227.2 | Benign neoplasm of parathyroid gland | Neoplasms | 0.96 | 0.90 | 1.21 | 0.59 | 327049 | 390 | 326659 | FALSE |
| 696.42 | Psoriatic arthropathy | Dermatologic | 1.03 | 0.87 | 1.08 | 0.59 | 314689 | 740 | 313949 | FALSE |
| 751.12 | Congenital anomalies of male genital organs | Congenital Anomalies | 0.95 | 0.87 | 1.28 | 0.60 | 326248 | 228 | 326020 | FALSE |
| 619.2 | Disorders of uterus, NEC | Genitourinary | 0.99 | 0.96 | 1.07 | 0.60 | 281956 | 3391 | 278565 | FALSE |
| 946 | Anaphylactic shock NOS | Injuries & Poisonings | 0.97 | 0.91 | 1.17 | 0.60 | 322467 | 544 | 321923 | FALSE |
| 751.22 | Other specified congenital anomalies of kidney | Congenital Anomalies | 1.04 | 0.82 | 1.12 | 0.60 | 326370 | 350 | 326020 | FALSE |
| 721.1 | Spondylosis without myelopathy | Musculoskeletal | 0.98 | 0.94 | 1.12 | 0.60 | 312031 | 1070 | 310961 | FALSE |
| 805 | Fracture of vertebral column without mention of spinal cord injury | Injuries & Poisonings | 0.96 | 0.89 | 1.22 | 0.60 | 323907 | 333 | 323574 | FALSE |
| 743.11 | Osteoporosis NOS | Musculoskeletal | 1.01 | 0.94 | 1.04 | 0.60 | 327252 | 3036 | 324216 | FALSE |
| 519.2 | Respiratory complications | Respiratory | 1.05 | 0.81 | 1.13 | 0.60 | 265760 | 296 | 265464 | FALSE |
| 586.2 | Cyst of kidney, acquired | Genitourinary | 0.98 | 0.94 | 1.11 | 0.60 | 315389 | 1293 | 314096 | FALSE |
| 458.2 | Iatrogenic hypotension | Circulatory System | 1.05 | 0.79 | 1.15 | 0.60 | 194434 | 238 | 194196 | FALSE |
| 559 | Ileostomy status | Digestive | 1.02 | 0.91 | 1.05 | 0.61 | 260086 | 1673 | 258413 | FALSE |
| 442.1 | Aortic aneurysm | Circulatory System | 0.97 | 0.92 | 1.16 | 0.61 | 319963 | 586 | 319377 | FALSE |
| 571.6 | Primary biliary cirrhosis | Digestive | 1.05 | 0.80 | 1.14 | 0.61 | 318606 | 258 | 318348 | FALSE |
| 728.71 | Contracture of palmar fascia [Dupuytren's disease] | Musculoskeletal | 0.98 | 0.96 | 1.08 | 0.61 | 307420 | 2449 | 304971 | FALSE |
| 306.9 | Tension headache | Mental Disorders | 0.96 | 0.88 | 1.23 | 0.61 | 283220 | 305 | 282915 | FALSE |
| 938 | Dermatitis due to solar radiation | Injuries & Poisonings | 1.02 | 0.93 | 1.04 | 0.61 | 324461 | 2538 | 321923 | FALSE |
| 245.21 | Chronic lymphocytic thyroiditis | Endocrine/Metabolic | 0.95 | 0.86 | 1.28 | 0.61 | 310565 | 219 | 310346 | FALSE |
| 441.1 | Acute vascular insufficiency of intestine | Circulatory System | 0.96 | 0.88 | 1.25 | 0.62 | 319648 | 271 | 319377 | FALSE |
| 804 | Fracture of hand or wrist | Injuries & Poisonings | 0.97 | 0.92 | 1.15 | 0.62 | 324249 | 675 | 323574 | FALSE |

| | | | | | | | | | | |
|---|---|---|---|---|---|---|---|---|---|---|
| 411 | Ischemic Heart Disease | Circulatory System | 1.00 | 0.98 | 1.01 | 0.62 | 327049 | 31343 | 295706 | FALSE |
| 221 | Benign neoplasm of other female genital organs | Neoplasms | 1.05 | 0.79 | 1.15 | 0.62 | 295991 | 223 | 295768 | FALSE |
| 280.1 | Iron deficiency anemias, unspecified or not due to blood loss | Hematopoietic | 1.01 | 0.96 | 1.03 | 0.62 | 316419 | 7502 | 308917 | FALSE |
| 681.1 | Cellulitis and abscess of fingers/toes | Dermatologic | 0.97 | 0.91 | 1.16 | 0.62 | 317006 | 582 | 316424 | FALSE |
| 704 | Diseases of hair and hair follicles | Dermatologic | 0.99 | 0.97 | 1.05 | 0.62 | 326709 | 5263 | 321446 | FALSE |
| 386.1 | Meniere's disease | Sense Organs | 0.97 | 0.91 | 1.17 | 0.62 | 322230 | 564 | 321666 | FALSE |
| 519 | Other diseases of respiratory system, not elsewhere classified | Respiratory | 1.00 | 0.98 | 1.01 | 0.62 | 327632 | 62168 | 265464 | FALSE |
| 172.2 | Other non-epithelial cancer of skin | Neoplasms | 0.99 | 0.98 | 1.04 | 0.62 | 325479 | 11242 | 314237 | FALSE |
| 471 | Nasal polyps | Respiratory | 0.99 | 0.96 | 1.06 | 0.63 | 312359 | 3392 | 308967 | FALSE |
| 509.1 | Respiratory failure | Respiratory | 1.02 | 0.92 | 1.05 | 0.63 | 318581 | 2080 | 316501 | FALSE |
| 523.31 | Acute periodontitis | Digestive | 1.03 | 0.87 | 1.08 | 0.63 | 312388 | 713 | 311675 | FALSE |
| 555.1 | Regional enteritis | Digestive | 1.02 | 0.92 | 1.05 | 0.63 | 260218 | 1805 | 258413 | FALSE |
| 612.2 | Hypertrophy of breast (Gynecomastia) | Genitourinary | 0.98 | 0.93 | 1.13 | 0.63 | 321749 | 859 | 320890 | FALSE |
| 574.12 | Cholelithiasis with other cholecystitis | Digestive | 1.01 | 0.95 | 1.03 | 0.63 | 315833 | 5493 | 310340 | FALSE |
| 599.9 | Other abnormality of urination | Genitourinary | 0.98 | 0.95 | 1.09 | 0.63 | 222388 | 1944 | 220444 | FALSE |
| 596 | Other disorders of bladder | Genitourinary | 1.01 | 0.96 | 1.03 | 0.63 | 321402 | 7749 | 313653 | FALSE |
| 385.5 | Tympanosclerosis and middle ear disease related to otitis media | Sense Organs | 0.95 | 0.87 | 1.27 | 0.63 | 324396 | 236 | 324160 | FALSE |
| 760 | Back pain | Symptoms | 1.01 | 0.95 | 1.03 | 0.63 | 328257 | 5923 | 322334 | FALSE |
| 200 | Myeloproliferative disease | Neoplasms | 1.03 | 0.87 | 1.09 | 0.63 | 324219 | 679 | 323540 | FALSE |
| 452 | Other venous embolism and thrombosis | Circulatory System | 0.97 | 0.91 | 1.17 | 0.64 | 288284 | 554 | 287730 | FALSE |
| 742.8 | Articular cartilage disorder | Musculoskeletal | 1.03 | 0.87 | 1.09 | 0.64 | 311314 | 655 | 310659 | FALSE |
| 440.9 | Atherosclerosis of aorta | Circulatory System | 1.05 | 0.78 | 1.17 | 0.64 | 319579 | 202 | 319377 | FALSE |
| 698 | Pruritus and related conditions | Dermatologic | 1.02 | 0.88 | 1.08 | 0.64 | 328257 | 799 | 327458 | FALSE |
| 312 | Conduct disorders | Mental Disorders | 0.98 | 0.94 | 1.11 | 0.64 | 327655 | 1195 | 326460 | FALSE |
| 262 | Mineral deficiency NEC | Endocrine/Metabolic | 0.96 | 0.87 | 1.26 | 0.64 | 326044 | 241 | 325803 | FALSE |
| 276.41 | Acidosis | Endocrine/Metabolic | 0.98 | 0.93 | 1.12 | 0.64 | 321717 | 1056 | 320661 | FALSE |
| 480.1 | Bacterial pneumonia | Respiratory | 0.98 | 0.93 | 1.12 | 0.64 | 318645 | 939 | 317706 | FALSE |
| 634.1 | Missed abortion/Hydatidiform mole | Pregnancy Complications | 0.98 | 0.94 | 1.11 | 0.64 | 321185 | 1203 | 319982 | FALSE |
| 331.1 | Hydrocephalus | Neurological | 1.03 | 0.85 | 1.10 | 0.64 | 286669 | 500 | 286169 | FALSE |
| 601.11 | Acute prostatitis | Genitourinary | 0.96 | 0.87 | 1.25 | 0.64 | 307670 | 260 | 307410 | FALSE |
| 199 | Neoplasm of uncertain behavior | Neoplasms | 1.02 | 0.90 | 1.07 | 0.64 | 230485 | 1156 | 229329 | FALSE |
| 751.2 | Congenital anomalies of urinary system | Congenital Anomalies | 0.98 | 0.93 | 1.13 | 0.65 | 326925 | 905 | 326020 | FALSE |
| 525 | Other diseases of the teeth and supporting structures | Digestive | 1.01 | 0.93 | 1.04 | 0.65 | 314374 | 2699 | 311675 | FALSE |
| 619.1 | Noninflammatory disorders of ovary, fallopian tube, and broad ligament | Genitourinary | 1.03 | 0.88 | 1.09 | 0.65 | 279299 | 734 | 278565 | FALSE |
| 613.7 | Other signs and symptoms in breast | Genitourinary | 0.98 | 0.92 | 1.14 | 0.65 | 323186 | 751 | 322435 | FALSE |
| 433.8 | Late effects of cerebrovascular disease | Circulatory System | 1.02 | 0.91 | 1.06 | 0.65 | 319401 | 1277 | 318124 | FALSE |
| 575.6 | Cholesterolosis of gallbladder | Digestive | 1.03 | 0.85 | 1.11 | 0.65 | 310806 | 466 | 310340 | FALSE |
| 535.2 | Atrophic gastritis | Digestive | 0.97 | 0.89 | 1.21 | 0.65 | 296886 | 358 | 296528 | FALSE |

| | | | | | | | | | | | |
|---|---|---|---|---|---|---|---|---|---|---|---|
| 530.11 | GERD | Digestive | 1.01 | 0.97 | 1.02 | 0.65 | 302309 | 14489 | 287820 | FALSE |
| 367.2 | Astigmatism | Sense Organs | 1.05 | 0.78 | 1.17 | 0.66 | 325981 | 210 | 325771 | FALSE |
| 198.6 | Secondary malignancy of bone | Neoplasms | 1.01 | 0.93 | 1.05 | 0.66 | 231522 | 2193 | 229329 | FALSE |
| 789 | Nausea and vomiting | Symptoms | 0.99 | 0.98 | 1.03 | 0.66 | 328257 | 11983 | 316274 | FALSE |
| 696.41 | Psoriasis vulgaris | Dermatologic | 1.02 | 0.92 | 1.06 | 0.66 | 315672 | 1723 | 313949 | FALSE |
| 707 | Chronic ulcer of skin | Dermatologic | 1.02 | 0.91 | 1.07 | 0.66 | 327976 | 1260 | 326716 | FALSE |
| 292.2 | Mild cognitive impairment | Mental Disorders | 0.96 | 0.85 | 1.28 | 0.66 | 321689 | 202 | 321487 | FALSE |
| 455 | Hemorrhoids | Circulatory System | 1.00 | 0.98 | 1.02 | 0.66 | 312427 | 24697 | 287730 | FALSE |
| 530.3 | Stricture and stenosis of esophagus | Digestive | 0.99 | 0.95 | 1.08 | 0.67 | 289774 | 1954 | 287820 | FALSE |
| 614.3 | Pelvic inflammatory disease (PID) | Genitourinary | 0.96 | 0.87 | 1.25 | 0.67 | 319065 | 249 | 318816 | FALSE |
| 189.4 | Malignant neoplasm of other urinary organs | Neoplasms | 1.01 | 0.94 | 1.04 | 0.67 | 327202 | 3223 | 323979 | FALSE |
| 512.2 | Painful respiration | Respiratory | 1.04 | 0.79 | 1.16 | 0.67 | 314513 | 228 | 314285 | FALSE |
| 674 | Other complications of the puerperium NEC | Pregnancy Complications | 1.04 | 0.81 | 1.15 | 0.68 | 328032 | 280 | 327752 | FALSE |
| 578.9 | Hemorrhage of gastrointestinal tract | Digestive | 1.01 | 0.95 | 1.03 | 0.68 | 308866 | 5455 | 303411 | FALSE |
| 447.1 | Stricture of artery | Circulatory System | 0.98 | 0.93 | 1.13 | 0.68 | 320263 | 886 | 319377 | FALSE |
| 480.11 | Pneumococcal pneumonia | Respiratory | 0.99 | 0.97 | 1.05 | 0.68 | 323697 | 5991 | 317706 | FALSE |
| 411.1 | Unstable angina (intermediate coronary syndrome) | Circulatory System | 1.01 | 0.95 | 1.03 | 0.68 | 301028 | 5322 | 295706 | FALSE |
| 327.3 | Sleep apnea | Neurological | 1.01 | 0.95 | 1.03 | 0.68 | 327436 | 4699 | 322737 | FALSE |
| 285.2 | Anemia of chronic disease | Hematopoietic | 0.97 | 0.87 | 1.23 | 0.68 | 309211 | 294 | 308917 | FALSE |
| 859 | Complication due to other implant and internal device | Injuries & Poisonings | 1.01 | 0.94 | 1.04 | 0.68 | 317319 | 3338 | 313981 | FALSE |
| 246 | Other disorders of thyroid | Endocrine/Metabolic | 1.00 | 0.97 | 1.02 | 0.68 | 327755 | 17409 | 310346 | FALSE |
| 296.22 | Major depressive disorder | Mental Disorders | 1.03 | 0.84 | 1.12 | 0.68 | 283299 | 384 | 282915 | FALSE |
| 430.1 | Subarachnoid hemorrhage | Circulatory System | 0.98 | 0.92 | 1.13 | 0.68 | 318948 | 824 | 318124 | FALSE |
| 585.2 | Renal failure NOS | Genitourinary | 1.01 | 0.96 | 1.03 | 0.68 | 322841 | 8745 | 314096 | FALSE |
| 568.1 | Peritoneal adhesions (postoperative) (postinfection) | Digestive | 1.01 | 0.94 | 1.04 | 0.68 | 249774 | 3097 | 246677 | FALSE |
| 386.3 | Labyrinthitis | Sense Organs | 0.98 | 0.92 | 1.13 | 0.68 | 322457 | 791 | 321666 | FALSE |
| 628 | Ovarian cyst | Genitourinary | 0.99 | 0.97 | 1.05 | 0.68 | 301068 | 4820 | 296248 | FALSE |
| 528.5 | Diseases of lips | Digestive | 1.02 | 0.88 | 1.09 | 0.69 | 323232 | 681 | 322551 | FALSE |
| 348.9 | Other conditions of brain, NOS | Neurological | 0.97 | 0.89 | 1.19 | 0.69 | 286576 | 407 | 286169 | FALSE |
| 599.5 | Frequency of urination and polyuria | Genitourinary | 1.01 | 0.95 | 1.04 | 0.69 | 224591 | 4147 | 220444 | FALSE |
| 530.2 | Esophageal bleeding (varices/hemorrhage) | Digestive | 0.99 | 0.95 | 1.09 | 0.69 | 289542 | 1722 | 287820 | FALSE |
| 593 | Hematuria | Genitourinary | 1.00 | 0.97 | 1.02 | 0.69 | 313812 | 16760 | 297052 | FALSE |
| 709.7 | Unspecified diffuse connective tissue disease | Dermatologic | 1.00 | 0.99 | 1.01 | 0.69 | 324652 | 82669 | 241983 | FALSE |
| 614.54 | Abscess or ulceration of vulva | Genitourinary | 0.97 | 0.87 | 1.23 | 0.69 | 319096 | 280 | 318816 | FALSE |
| 465.2 | Acute pharyngitis | Respiratory | 1.02 | 0.89 | 1.08 | 0.69 | 326070 | 874 | 325196 | FALSE |
| 555 | Inflammatory bowel disease and other gastroenteritis and colitis | Digestive | 1.00 | 0.98 | 1.03 | 0.69 | 278212 | 19799 | 258413 | FALSE |
| 427.41 | Ventricular fibrillation and flutter | Circulatory System | 1.03 | 0.83 | 1.13 | 0.69 | 299883 | 345 | 299538 | FALSE |
| 288.11 | Neutropenia | Hematopoietic | 1.01 | 0.94 | 1.04 | 0.69 | 323661 | 3326 | 320335 | FALSE |

| | | | | | | | | | | | |
|---|---|---|---|---|---|---|---|---|---|---|---|
| 174 | Breast cancer | Neoplasms | 1.03 | 0.82 | 1.15 | 0.69 | 304729 | 288 | 304441 | FALSE |
| 473 | Diseases of the larynx and vocal cords | Respiratory | 0.99 | 0.94 | 1.09 | 0.69 | 310581 | 1614 | 308967 | FALSE |
| 182 | Malignant neoplasm of uterus | Neoplasms | 0.98 | 0.94 | 1.10 | 0.70 | 298965 | 1303 | 297662 | FALSE |
| 707.1 | Decubitus ulcer | Dermatologic | 1.03 | 0.83 | 1.14 | 0.70 | 327042 | 326 | 326716 | FALSE |
| 532 | Dysphagia | Digestive | 0.99 | 0.97 | 1.04 | 0.70 | 294373 | 6553 | 287820 | FALSE |
| 426.32 | Left bundle branch block | Circulatory System | 0.99 | 0.95 | 1.09 | 0.70 | 301319 | 1781 | 299538 | FALSE |
| 41.4 | E. coli | Infectious Diseases | 1.01 | 0.94 | 1.05 | 0.70 | 315449 | 2800 | 312649 | FALSE |
| 415 | Pulmonary heart disease | Circulatory System | 0.99 | 0.96 | 1.06 | 0.70 | 325317 | 3753 | 321564 | FALSE |
| 687.4 | Disturbance of skin sensation | Dermatologic | 1.01 | 0.94 | 1.04 | 0.70 | 325810 | 2979 | 322831 | FALSE |
| 610.8 | Other specified benign mammary dysplasias | Genitourinary | 1.02 | 0.88 | 1.09 | 0.70 | 321593 | 703 | 320890 | FALSE |
| 781 | Symptoms involving nervous and musculoskeletal systems | Symptoms | 1.00 | 0.98 | 1.02 | 0.70 | 328255 | 22180 | 306075 | FALSE |
| 170.2 | Cancer of connective tissue | Neoplasms | 1.02 | 0.88 | 1.09 | 0.70 | 328139 | 734 | 327405 | FALSE |
| 568 | Other disorders of peritoneum | Digestive | 1.01 | 0.95 | 1.04 | 0.70 | 250481 | 3804 | 246677 | FALSE |
| 427.7 | Tachycardia NOS | Circulatory System | 1.01 | 0.93 | 1.05 | 0.70 | 301804 | 2266 | 299538 | FALSE |
| 427.4 | Cardiac arrest and ventricular fibrillation | Circulatory System | 1.03 | 0.83 | 1.13 | 0.71 | 299886 | 348 | 299538 | FALSE |
| 558 | Noninfectious gastroenteritis | Digestive | 1.00 | 0.98 | 1.03 | 0.71 | 278277 | 19864 | 258413 | FALSE |
| 292.1 | Aphasia/speech disturbance | Mental Disorders | 1.01 | 0.92 | 1.06 | 0.71 | 323036 | 1549 | 321487 | FALSE |
| 572 | Ascites (non malignant) | Digestive | 1.01 | 0.92 | 1.06 | 0.71 | 319942 | 1594 | 318348 | FALSE |
| 451 | Phlebitis and thrombophlebitis | Circulatory System | 1.03 | 0.84 | 1.12 | 0.71 | 288127 | 397 | 287730 | FALSE |
| 556.1 | Ulceration of intestine | Digestive | 0.98 | 0.91 | 1.14 | 0.71 | 259104 | 691 | 258413 | FALSE |
| 451.2 | Phlebitis and thrombophlebitis of lower extremities | Circulatory System | 0.99 | 0.96 | 1.06 | 0.71 | 291374 | 3644 | 287730 | FALSE |
| 379.5 | Disorders of iris and ciliary body | Sense Organs | 0.97 | 0.89 | 1.18 | 0.71 | 288621 | 412 | 288209 | FALSE |
| 577 | Diseases of pancreas | Digestive | 1.02 | 0.87 | 1.10 | 0.71 | 326495 | 558 | 325937 | FALSE |
| 195.1 | Malignant neoplasm, other | Neoplasms | 1.00 | 0.99 | 1.01 | 0.72 | 320155 | 90826 | 229329 | FALSE |
| 627.1 | Postmenopausal bleeding | Genitourinary | 0.99 | 0.98 | 1.04 | 0.72 | 305714 | 9466 | 296248 | FALSE |
| 626 | Disorders of menstruation and other abnormal bleeding from female genital tract | Genitourinary | 0.99 | 0.96 | 1.06 | 0.72 | 300195 | 3947 | 296248 | FALSE |
| 764 | Sciatica | Symptoms | 1.02 | 0.91 | 1.07 | 0.72 | 327961 | 1261 | 326700 | FALSE |
| 204 | Leukemia | Neoplasms | 0.98 | 0.90 | 1.17 | 0.72 | 324001 | 461 | 323540 | FALSE |
| 601.12 | Chronic prostatitis | Genitourinary | 1.02 | 0.90 | 1.08 | 0.72 | 308358 | 948 | 307410 | FALSE |
| 290 | Delirium dementia and amnestic and other cognitive disorders | Mental Disorders | 1.02 | 0.86 | 1.11 | 0.72 | 321967 | 480 | 321487 | FALSE |
| 803.1 | Fracture of humerus | Injuries & Poisonings | 0.97 | 0.87 | 1.22 | 0.73 | 323867 | 293 | 323574 | FALSE |
| 189 | Cancer of urinary organs (incl. kidney and bladder) | Neoplasms | 1.01 | 0.94 | 1.04 | 0.73 | 326982 | 3003 | 323979 | FALSE |
| 372 | Disorders of conjunctiva | Sense Organs | 0.98 | 0.93 | 1.11 | 0.73 | 319448 | 1043 | 318405 | FALSE |
| 338.2 | Chronic pain | Neurological | 0.97 | 0.89 | 1.18 | 0.73 | 327847 | 404 | 327443 | FALSE |
| 994.2 | Sepsis | Injuries & Poisonings | 1.01 | 0.94 | 1.05 | 0.73 | 328257 | 2885 | 325372 | FALSE |
| 172.3 | Carcinoma in situ of skin | Neoplasms | 0.98 | 0.91 | 1.14 | 0.73 | 314901 | 664 | 314237 | FALSE |
| 741 | Symptoms and disorders of the joints | Musculoskeletal | 1.00 | 0.97 | 1.02 | 0.73 | 327548 | 16889 | 310659 | FALSE |
| 385.3 | Cholesteatoma | Sense Organs | 0.98 | 0.91 | 1.15 | 0.73 | 324749 | 589 | 324160 | FALSE |

| | | | | | | | | | | |
|---|---|---|---|---|---|---|---|---|---|---|
| 433 | Cerebrovascular disease | Circulatory System | 0.99 | 0.97 | 1.04 | 0.73 | 326770 | 8646 | 318124 | FALSE |
| 747 | Cardiac and circulatory congenital anomalies | Congenital Anomalies | 1.01 | 0.91 | 1.07 | 0.73 | 326593 | 1222 | 325371 | FALSE |
| 361.1 | Retinal detachment with retinal defect | Sense Organs | 0.99 | 0.94 | 1.09 | 0.74 | 318255 | 1456 | 316799 | FALSE |
| 594.1 | Calculus of kidney | Genitourinary | 1.01 | 0.94 | 1.04 | 0.74 | 323282 | 3334 | 319948 | FALSE |
| 756 | Other congenital musculoskeletal anomalies | Congenital Anomalies | 1.02 | 0.89 | 1.09 | 0.74 | 328140 | 812 | 327328 | FALSE |
| 416 | Cardiomegaly | Circulatory System | 0.99 | 0.95 | 1.07 | 0.74 | 324174 | 2610 | 321564 | FALSE |
| 522.5 | Periapical abscess | Digestive | 0.99 | 0.93 | 1.10 | 0.74 | 312921 | 1246 | 311675 | FALSE |
| 367.8 | Hypermetropia | Sense Organs | 0.97 | 0.86 | 1.24 | 0.74 | 326012 | 241 | 325771 | FALSE |
| 585.1 | Acute renal failure | Genitourinary | 0.99 | 0.97 | 1.05 | 0.74 | 318761 | 4665 | 314096 | FALSE |
| 276.13 | Hyperpotassemia | Endocrine/Metabolic | 0.98 | 0.93 | 1.11 | 0.74 | 321640 | 979 | 320661 | FALSE |
| 735.21 | Hammer toe (acquired) | Musculoskeletal | 1.01 | 0.93 | 1.06 | 0.74 | 318718 | 2014 | 316704 | FALSE |
| 427.9 | Palpitations | Circulatory System | 1.01 | 0.95 | 1.04 | 0.74 | 303497 | 3959 | 299538 | FALSE |
| 480 | Pneumonia | Respiratory | 0.99 | 0.96 | 1.06 | 0.74 | 321620 | 3914 | 317706 | FALSE |
| 289 | Other diseases of blood and blood-forming organs | Hematopoietic | 0.99 | 0.97 | 1.05 | 0.75 | 325317 | 4982 | 320335 | FALSE |
| 819 | Skull and face fracture and other intercranial injury | Injuries & Poisonings | 0.99 | 0.94 | 1.09 | 0.75 | 328073 | 1517 | 326556 | FALSE |
| 295.1 | Schizophrenia | Mental Disorders | 1.02 | 0.87 | 1.10 | 0.75 | 283526 | 611 | 282915 | FALSE |
| 8.5 | Bacterial enteritis | Infectious Diseases | 1.01 | 0.93 | 1.05 | 0.75 | 321205 | 2109 | 319096 | FALSE |
| 610.3 | Fibrosclerosis of breast | Genitourinary | 0.97 | 0.87 | 1.21 | 0.76 | 321193 | 303 | 320890 | FALSE |
| 696.4 | Psoriasis | Dermatologic | 1.01 | 0.92 | 1.06 | 0.76 | 315571 | 1622 | 313949 | FALSE |
| 503 | Pulmonary congestion and hypostasis | Respiratory | 0.98 | 0.89 | 1.17 | 0.76 | 316976 | 475 | 316501 | FALSE |
| 520.2 | Disturbances in tooth eruption | Digestive | 1.01 | 0.93 | 1.05 | 0.76 | 314112 | 2437 | 311675 | FALSE |
| 155 | Cancer of liver and intrahepatic bile duct | Neoplasms | 0.97 | 0.86 | 1.24 | 0.76 | 312593 | 245 | 312348 | FALSE |
| 332 | Parkinson's disease | Neurological | 0.99 | 0.93 | 1.10 | 0.76 | 287351 | 1182 | 286169 | FALSE |
| 430.3 | Subdural hemorrhage | Circulatory System | 0.97 | 0.86 | 1.23 | 0.76 | 318395 | 271 | 318124 | FALSE |
| 870.1 | Open wound or laceration of eye or eyelid | Injuries & Poisonings | 1.03 | 0.82 | 1.16 | 0.76 | 320029 | 281 | 319748 | FALSE |
| 300.12 | Agorophobia, social phobia, and panic disorder | Mental Disorders | 0.98 | 0.91 | 1.13 | 0.76 | 283659 | 744 | 282915 | FALSE |
| 8 | Intestinal infection | Infectious Diseases | 1.00 | 0.97 | 1.04 | 0.76 | 328257 | 9161 | 319096 | FALSE |
| 580.32 | Nephritis and nephropathy with pathological lesion | Genitourinary | 0.99 | 0.93 | 1.11 | 0.76 | 315174 | 1078 | 314096 | FALSE |
| 522 | Diseases of pulp and periapical tissues | Digestive | 1.02 | 0.88 | 1.10 | 0.77 | 312314 | 639 | 311675 | FALSE |
| 211 | Benign neoplasm of other parts of digestive system | Neoplasms | 0.99 | 0.97 | 1.05 | 0.77 | 319700 | 5375 | 314325 | FALSE |
| 592.1 | Cystitis | Genitourinary | 0.99 | 0.95 | 1.08 | 0.77 | 299106 | 2054 | 297052 | FALSE |
| 722.1 | Displacement of intervertebral disc | Musculoskeletal | 1.02 | 0.86 | 1.11 | 0.77 | 311477 | 516 | 310961 | FALSE |
| 747.13 | Congenital anomalies of great vessels | Congenital Anomalies | 0.99 | 0.94 | 1.08 | 0.77 | 327194 | 1823 | 325371 | FALSE |
| 569.2 | Gastrointestinal complications | Digestive | 1.02 | 0.86 | 1.12 | 0.77 | 247149 | 472 | 246677 | FALSE |
| 427.5 | Arrhythmia (cardiac) NOS | Circulatory System | 1.01 | 0.90 | 1.08 | 0.77 | 300486 | 948 | 299538 | FALSE |
| 200.1 | Polycythemia vera | Neoplasms | 0.98 | 0.88 | 1.18 | 0.77 | 318153 | 404 | 317749 | FALSE |
| 502 | Postinflammatory pulmonary fibrosis | Respiratory | 0.99 | 0.92 | 1.12 | 0.77 | 317384 | 883 | 316501 | FALSE |
| 979 | Adverse drug events and drug allergies | Injuries & Poisonings | 1.02 | 0.88 | 1.10 | 0.78 | 300944 | 716 | 300228 | FALSE |

| | | | | | | | | | | | | |
|---|---|---|---|---|---|---|---|---|---|---|---|---|
| 255.21 | Glucocorticoid deficiency | Endocrine/Metabolic | 1.02 | 0.84 | 1.14 | 0.78 | 324934 | 372 | 324562 | FALSE |
| 285 | Other anemias | Hematopoietic | 1.00 | 0.98 | 1.03 | 0.78 | 320761 | 11844 | 308917 | FALSE |
| 721 | Spondylosis and allied disorders | Musculoskeletal | 0.99 | 0.92 | 1.11 | 0.78 | 311886 | 925 | 310961 | FALSE |
| 70.4 | Chronic hepatitis | Infectious Diseases | 1.02 | 0.82 | 1.16 | 0.79 | 322513 | 290 | 322223 | FALSE |
| 159 | Malignant neoplasm of other and ill-defined sites within the digestive organs and peritoneum | Neoplasms | 1.01 | 0.96 | 1.04 | 0.79 | 317637 | 5289 | 312348 | FALSE |
| 244.1 | Secondary hypothyroidism | Endocrine/Metabolic | 1.01 | 0.91 | 1.08 | 0.79 | 311501 | 1155 | 310346 | FALSE |
| 293 | Symptoms involving head and neck | Mental Disorders | 0.99 | 0.95 | 1.07 | 0.79 | 327340 | 2204 | 325136 | FALSE |
| 506 | Empyema and pneumothorax | Respiratory | 0.99 | 0.93 | 1.10 | 0.79 | 317647 | 1146 | 316501 | FALSE |
| 198 | Secondary malignant neoplasm | Neoplasms | 0.99 | 0.93 | 1.10 | 0.79 | 230448 | 1119 | 229329 | FALSE |
| 966 | Poisoning by anticonvulsants and anti-Parkinsonism drugs | Injuries & Poisonings | 1.02 | 0.87 | 1.11 | 0.79 | 300764 | 536 | 300228 | FALSE |
| 81 | Infection/inflammation of internal prosthetic device; implant; and graft | Infectious Diseases | 0.99 | 0.95 | 1.07 | 0.79 | 324010 | 2514 | 321496 | FALSE |
| 193 | Thyroid cancer | Neoplasms | 0.98 | 0.88 | 1.19 | 0.79 | 327026 | 367 | 326659 | FALSE |
| 733 | Other disorders of bone and cartilage | Musculoskeletal | 0.98 | 0.90 | 1.15 | 0.80 | 316018 | 569 | 315449 | FALSE |
| 727 | Other disorders of synovium, tendon, and bursa | Musculoskeletal | 1.01 | 0.89 | 1.10 | 0.80 | 305725 | 754 | 304971 | FALSE |
| 761 | Cervicalgia | Symptoms | 1.01 | 0.90 | 1.08 | 0.80 | 328257 | 1005 | 327252 | FALSE |
| 496.3 | Bronchiectasis | Respiratory | 0.99 | 0.94 | 1.08 | 0.80 | 296094 | 1885 | 294209 | FALSE |
| 317.11 | Alcoholic liver damage | Mental Disorders | 1.01 | 0.89 | 1.09 | 0.80 | 298551 | 865 | 297686 | FALSE |
| 366.2 | Senile cataract | Sense Organs | 1.00 | 0.97 | 1.04 | 0.80 | 315859 | 8458 | 307401 | FALSE |
| 334 | Degenerative disease of the spinal cord | Neurological | 0.99 | 0.94 | 1.09 | 0.80 | 287648 | 1479 | 286169 | FALSE |
| 477 | Epistaxis or throat hemorrhage | Respiratory | 0.99 | 0.95 | 1.07 | 0.80 | 311486 | 2519 | 308967 | FALSE |
| 573.9 | Abnormal serum enzyme levels | Digestive | 0.98 | 0.85 | 1.24 | 0.80 | 318578 | 230 | 318348 | FALSE |
| 276.5 | Hypovolemia | Endocrine/Metabolic | 1.01 | 0.94 | 1.05 | 0.80 | 323556 | 2895 | 320661 | FALSE |
| 523 | Gingival and periodontal diseases | Digestive | 0.98 | 0.90 | 1.15 | 0.80 | 312204 | 529 | 311675 | FALSE |
| 172.11 | Melanomas of skin | Neoplasms | 1.01 | 0.94 | 1.05 | 0.80 | 316960 | 2723 | 314237 | FALSE |
| 454 | Varicose veins | Circulatory System | 0.99 | 0.94 | 1.09 | 0.80 | 289333 | 1603 | 287730 | FALSE |
| 195 | Cancer, suspected or other | Neoplasms | 0.99 | 0.92 | 1.11 | 0.81 | 230244 | 915 | 229329 | FALSE |
| 289.5 | Diseases of spleen | Hematopoietic | 0.98 | 0.90 | 1.15 | 0.81 | 320863 | 528 | 320335 | FALSE |
| 528 | Diseases of the oral soft tissues, excluding lesions specific for gingiva and tongue | Digestive | 0.99 | 0.95 | 1.07 | 0.81 | 324610 | 2059 | 322551 | FALSE |
| 228 | Hemangioma and lymphangioma, any site | Neoplasms | 1.01 | 0.92 | 1.06 | 0.81 | 328257 | 1635 | 326622 | FALSE |
| 197 | Chemotherapy | Neoplasms | 1.00 | 0.98 | 1.02 | 0.81 | 251791 | 22462 | 229329 | FALSE |
| 279.7 | Other immunological findings | Endocrine/Metabolic | 1.02 | 0.82 | 1.17 | 0.81 | 327995 | 264 | 327731 | FALSE |
| 596.1 | Bladder neck obstruction | Genitourinary | 0.99 | 0.94 | 1.07 | 0.81 | 315682 | 2029 | 313653 | FALSE |
| 531.2 | Gastric ulcer | Digestive | 0.99 | 0.96 | 1.05 | 0.81 | 324794 | 4228 | 320566 | FALSE |
| 458 | Hypotension | Circulatory System | 0.99 | 0.93 | 1.10 | 0.82 | 195277 | 1081 | 194196 | FALSE |
| 369.5 | Conjunctivitis, infectious | Sense Organs | 0.98 | 0.84 | 1.24 | 0.82 | 318629 | 224 | 318405 | FALSE |
| 528.6 | Leukoplakia of oral mucosa | Digestive | 1.02 | 0.83 | 1.15 | 0.82 | 322863 | 312 | 322551 | FALSE |
| 610.1 | Cystic mastopathy | Genitourinary | 1.01 | 0.90 | 1.09 | 0.82 | 321849 | 959 | 320890 | FALSE |
| 8.6 | Viral Enteritis | Infectious Diseases | 1.01 | 0.90 | 1.09 | 0.82 | 319941 | 845 | 319096 | FALSE |

| | | | | | | | | | | |
|---|---|---|---|---|---|---|---|---|---|---|
| 595 | Hydronephrosis | Genitourinary | 1.01 | 0.93 | 1.06 | 0.82 | 321958 | 2010 | 319948 | FALSE |
| 418 | Nonspecific chest pain | Circulatory System | 1.00 | 0.98 | 1.02 | 0.83 | 326049 | 30212 | 295837 | FALSE |
| 359.2 | Myopathy | Neurological | 1.01 | 0.87 | 1.12 | 0.83 | 326467 | 498 | 325969 | FALSE |
| 500.2 | Pneumoconiosis | Respiratory | 1.01 | 0.90 | 1.08 | 0.83 | 317502 | 1001 | 316501 | FALSE |
| 516.1 | Hemoptysis | Respiratory | 0.99 | 0.95 | 1.07 | 0.83 | 328171 | 2103 | 326068 | FALSE |
| 788 | Syncope and collapse | Symptoms | 1.00 | 0.97 | 1.03 | 0.83 | 328257 | 9372 | 318885 | FALSE |
| 263 | Other nutritional deficiency | Endocrine/Metabolic | 1.01 | 0.92 | 1.07 | 0.83 | 327354 | 1551 | 325803 | FALSE |
| 383 | Otosclerosis | Sense Organs | 0.98 | 0.87 | 1.19 | 0.83 | 324490 | 330 | 324160 | FALSE |
| 642.1 | Preeclampsia and eclampsia | Pregnancy Complications | 1.02 | 0.84 | 1.15 | 0.83 | 327402 | 335 | 327067 | FALSE |
| 475 | Chronic sinusitis | Respiratory | 0.99 | 0.95 | 1.06 | 0.84 | 311597 | 2630 | 308967 | FALSE |
| 560.4 | Other intestinal obstruction | Digestive | 0.99 | 0.96 | 1.06 | 0.84 | 261856 | 3443 | 258413 | FALSE |
| 649.1 | Diabetes or abnormal glucose tolerance complicating pregnancy | Pregnancy Complications | 0.98 | 0.83 | 1.25 | 0.84 | 328144 | 208 | 327936 | FALSE |
| 440.2 | Atherosclerosis of the extremities | Circulatory System | 0.99 | 0.90 | 1.15 | 0.84 | 319929 | 552 | 319377 | FALSE |
| 627.2 | Symptomatic menopause | Genitourinary | 0.98 | 0.83 | 1.25 | 0.84 | 296451 | 203 | 296248 | FALSE |
| 858 | Complication of internal orthopedic device | Injuries & Poisonings | 0.99 | 0.95 | 1.06 | 0.84 | 317164 | 3183 | 313981 | FALSE |
| 433.3 | Cerebral ischemia | Circulatory System | 0.99 | 0.92 | 1.11 | 0.85 | 319058 | 934 | 318124 | FALSE |
| 509.8 | Dependence on respirator [Ventilator] or supplemental oxygen | Respiratory | 0.99 | 0.88 | 1.17 | 0.85 | 316902 | 401 | 316501 | FALSE |
| 446.5 | Giant cell arteritis | Circulatory System | 0.99 | 0.88 | 1.17 | 0.85 | 319767 | 390 | 319377 | FALSE |
| 722.6 | Degeneration of intervertebral disc | Musculoskeletal | 0.99 | 0.95 | 1.06 | 0.85 | 313883 | 2922 | 310961 | FALSE |
| 301 | Personality disorders | Mental Disorders | 1.01 | 0.86 | 1.13 | 0.85 | 283347 | 432 | 282915 | FALSE |
| 292 | Neurological disorders | Mental Disorders | 0.99 | 0.88 | 1.16 | 0.86 | 321936 | 449 | 321487 | FALSE |
| 213 | Benign neoplasm of bone and articular cartilage | Neoplasms | 1.01 | 0.84 | 1.16 | 0.86 | 277545 | 322 | 277223 | FALSE |
| 191.11 | Cancer of brain | Neoplasms | 0.99 | 0.89 | 1.15 | 0.86 | 326962 | 498 | 326464 | FALSE |
| 716.9 | Arthropathy NOS | Musculoskeletal | 1.00 | 0.99 | 1.02 | 0.86 | 327736 | 53823 | 273913 | FALSE |
| 214.1 | Lipoma of skin and subcutaneous tissue | Neoplasms | 1.00 | 0.96 | 1.04 | 0.86 | 325449 | 4756 | 320693 | FALSE |
| 292.6 | Hallucinations | Mental Disorders | 0.99 | 0.86 | 1.19 | 0.86 | 321810 | 323 | 321487 | FALSE |
| 218.2 | Other benign neoplasm of uterus | Neoplasms | 1.01 | 0.85 | 1.15 | 0.86 | 308160 | 363 | 307797 | FALSE |
| 735.3 | Hallux valgus (Bunion) | Musculoskeletal | 1.00 | 0.97 | 1.04 | 0.86 | 323609 | 6905 | 316704 | FALSE |
| 496.1 | Emphysema | Respiratory | 1.01 | 0.93 | 1.07 | 0.87 | 295935 | 1726 | 294209 | FALSE |
| 752.11 | Spina bifida | Congenital Anomalies | 1.02 | 0.81 | 1.20 | 0.87 | 327903 | 211 | 327692 | FALSE |
| 613.1 | Inflammatory disease of breast | Genitourinary | 1.01 | 0.89 | 1.10 | 0.87 | 323173 | 738 | 322435 | FALSE |
| 284 | Aplastic anemia | Hematopoietic | 1.00 | 0.98 | 1.03 | 0.87 | 321676 | 12759 | 308917 | FALSE |
| 509.2 | Respiratory insufficiency | Respiratory | 0.99 | 0.94 | 1.08 | 0.87 | 318289 | 1788 | 316501 | FALSE |
| 574.2 | Calculus of bile duct | Digestive | 1.00 | 0.94 | 1.05 | 0.87 | 313028 | 2688 | 310340 | FALSE |
| 411.9 | Other acute and subacute forms of ischemic heart disease | Circulatory System | 1.01 | 0.91 | 1.08 | 0.87 | 296900 | 1194 | 295706 | FALSE |
| 567 | Peritonitis and retroperitoneal infections | Digestive | 0.99 | 0.92 | 1.11 | 0.87 | 247567 | 890 | 246677 | FALSE |
| 947 | Urticaria | Injuries & Poisonings | 0.99 | 0.87 | 1.17 | 0.87 | 322310 | 387 | 321923 | FALSE |
| 474.1 | Acute tonsillitis | Respiratory | 1.01 | 0.88 | 1.12 | 0.87 | 309532 | 565 | 308967 | FALSE |

| | | | | | | | | | | |
|---|---|---|---|---|---|---|---|---|---|---|
| 577.1 | Acute pancreatitis | Digestive | 1.01 | 0.92 | 1.07 | 0.87 | 327454 | 1517 | 325937 | FALSE |
| 479 | Other upper respiratory disease | Respiratory | 1.00 | 0.98 | 1.03 | 0.87 | 324258 | 15291 | 308967 | FALSE |
| 478 | Throat pain | Respiratory | 0.99 | 0.87 | 1.17 | 0.87 | 309354 | 387 | 308967 | FALSE |
| 365.2 | Primary angle-closure glaucoma | Sense Organs | 1.01 | 0.89 | 1.10 | 0.87 | 317508 | 709 | 316799 | FALSE |
| 526.1 | Cysts of the jaws | Digestive | 0.99 | 0.86 | 1.19 | 0.87 | 312010 | 335 | 311675 | FALSE |
| 526.9 | Jaw disease NOS | Digestive | 1.00 | 0.98 | 1.02 | 0.88 | 327637 | 15962 | 311675 | FALSE |
| 610.4 | Benign neoplasm of breast | Genitourinary | 0.99 | 0.93 | 1.08 | 0.88 | 322376 | 1486 | 320890 | FALSE |
| 622 | Polyp of female genital organs | Genitourinary | 0.99 | 0.89 | 1.15 | 0.88 | 315811 | 487 | 315324 | FALSE |
| 337 | Disorders of the autonomic nervous system | Neurological | 1.02 | 0.81 | 1.20 | 0.88 | 286376 | 207 | 286169 | FALSE |
| 8.52 | Intestinal infection due to C. difficile | Infectious Diseases | 1.01 | 0.89 | 1.11 | 0.88 | 319744 | 648 | 319096 | FALSE |
| 350.2 | Abnormality of gait | Neurological | 0.99 | 0.94 | 1.08 | 0.88 | 327094 | 1633 | 325461 | FALSE |
| 957 | Injury to other and unspecified nerves | Injuries & Poisonings | 1.01 | 0.83 | 1.17 | 0.88 | 328174 | 275 | 327899 | FALSE |
| 646 | Other complications of pregnancy NEC | Pregnancy Complications | 1.00 | 0.94 | 1.06 | 0.88 | 328257 | 2396 | 325861 | FALSE |
| 430.2 | Intracerebral hemorrhage | Circulatory System | 1.01 | 0.89 | 1.10 | 0.89 | 318847 | 723 | 318124 | FALSE |
| 411.3 | Angina pectoris | Circulatory System | 1.00 | 0.98 | 1.02 | 0.89 | 312065 | 16359 | 295706 | FALSE |
| 575.8 | Other disorders of biliary tract | Digestive | 0.99 | 0.92 | 1.10 | 0.89 | 311389 | 1049 | 310340 | FALSE |
| 601.1 | Prostatitis | Genitourinary | 1.01 | 0.89 | 1.11 | 0.89 | 308075 | 665 | 307410 | FALSE |
| 727.1 | Synovitis and tenosynovitis | Musculoskeletal | 0.99 | 0.90 | 1.13 | 0.89 | 305658 | 687 | 304971 | FALSE |
| 703 | Diseases of nail, NOS | Dermatologic | 1.01 | 0.85 | 1.15 | 0.89 | 321812 | 366 | 321446 | FALSE |
| 747.12 | Valvular heart disease/ heart chambers | Congenital Anomalies | 0.99 | 0.84 | 1.22 | 0.90 | 325609 | 238 | 325371 | FALSE |
| 526 | Diseases of the jaws | Digestive | 1.00 | 0.98 | 1.02 | 0.90 | 327662 | 15987 | 311675 | FALSE |
| 510 | Other diseases of lung | Respiratory | 0.99 | 0.91 | 1.12 | 0.90 | 328217 | 770 | 327447 | FALSE |
| 965 | Poisoning by analgesics, antipyretics, and antirheumatics | Injuries & Poisonings | 1.00 | 0.97 | 1.04 | 0.90 | 306310 | 6082 | 300228 | FALSE |
| 809 | Fracture of unspecified bones | Injuries & Poisonings | 0.99 | 0.87 | 1.16 | 0.90 | 323984 | 410 | 323574 | FALSE |
| 573 | Other disorders of liver | Digestive | 1.00 | 0.96 | 1.04 | 0.91 | 323550 | 5202 | 318348 | FALSE |
| 800.3 | Fracture of tibia and fibula | Injuries & Poisonings | 1.01 | 0.88 | 1.12 | 0.91 | 324109 | 535 | 323574 | FALSE |
| 368.1 | Amblyopia | Sense Organs | 0.99 | 0.89 | 1.14 | 0.91 | 325440 | 541 | 324899 | FALSE |
| 686.3 | Pilonidal cyst | Dermatologic | 1.01 | 0.88 | 1.12 | 0.91 | 317041 | 617 | 316424 | FALSE |
| 395.6 | Heart valve replaced | Circulatory System | 1.00 | 0.92 | 1.07 | 0.91 | 323073 | 1528 | 321545 | FALSE |
| 444.1 | Arterial embolism and thrombosis of lower extremity artery | Circulatory System | 0.99 | 0.89 | 1.14 | 0.91 | 319947 | 570 | 319377 | FALSE |
| 465 | Acute upper respiratory infections of multiple or unspecified sites | Respiratory | 1.00 | 0.95 | 1.05 | 0.91 | 328249 | 3053 | 325196 | FALSE |
| 341 | Other demyelinating diseases of central nervous system | Neurological | 1.00 | 0.93 | 1.08 | 0.91 | 287781 | 1612 | 286169 | FALSE |
| 285.22 | Anemia in neoplastic disease | Hematopoietic | 1.01 | 0.86 | 1.14 | 0.91 | 309354 | 437 | 308917 | FALSE |
| 240 | Simple and unspecified goiter | Endocrine/Metabolic | 1.01 | 0.88 | 1.12 | 0.91 | 310968 | 622 | 310346 | FALSE |
| 740.11 | Osteoarthrosis, localized, primary | Musculoskeletal | 1.00 | 0.97 | 1.03 | 0.91 | 315569 | 9227 | 306342 | FALSE |
| 386.2 | Peripheral or central vertigo | Sense Organs | 1.01 | 0.86 | 1.15 | 0.91 | 322053 | 387 | 321666 | FALSE |
| 626.12 | Excessive or frequent menstruation | Genitourinary | 1.00 | 0.97 | 1.03 | 0.92 | 306289 | 10041 | 296248 | FALSE |
| 586.4 | Stricture/obstruction of ureter | Genitourinary | 1.01 | 0.91 | 1.09 | 0.92 | 315035 | 939 | 314096 | FALSE |

| | | | | | | | | | | |
|---|---|---|---|---|---|---|---|---|---|---|
| 715 | Other inflammatory spondylopathies | Musculoskeletal | 1.01 | 0.83 | 1.18 | 0.92 | 317541 | 271 | 317270 | FALSE |
| 695.9 | Unspecified erythematous condition | Dermatologic | 0.99 | 0.88 | 1.15 | 0.92 | 322243 | 477 | 321766 | FALSE |
| 716.1 | Unspecified polyarthropathy or polyarthritis | Musculoskeletal | 1.00 | 0.95 | 1.05 | 0.92 | 277443 | 3530 | 273913 | FALSE |
| 339 | Other headache syndromes | Neurological | 1.00 | 0.97 | 1.03 | 0.92 | 325905 | 7938 | 317967 | FALSE |
| 969 | Poisoning by psychotropic agents | Injuries & Poisonings | 1.00 | 0.93 | 1.06 | 0.92 | 302224 | 1996 | 300228 | FALSE |
| 732 | Osteochondropathies | Musculoskeletal | 1.01 | 0.81 | 1.20 | 0.92 | 315668 | 219 | 315449 | FALSE |
| 380.1 | Otitis externa | Sense Organs | 1.01 | 0.88 | 1.13 | 0.92 | 327301 | 532 | 326769 | FALSE |
| 577.2 | Chronic pancreatitis | Digestive | 0.99 | 0.89 | 1.14 | 0.92 | 326473 | 536 | 325937 | FALSE |
| 340 | Migraine | Neurological | 1.00 | 0.94 | 1.05 | 0.92 | 320740 | 2773 | 317967 | FALSE |
| 420.21 | Acute pericarditis | Circulatory System | 1.01 | 0.82 | 1.20 | 0.92 | 325228 | 228 | 325000 | FALSE |
| 625 | Pain and other symptoms associated with female genital organs | Genitourinary | 1.00 | 0.94 | 1.07 | 0.93 | 320735 | 2049 | 318686 | FALSE |
| 785 | Abdominal pain | Symptoms | 1.00 | 0.98 | 1.01 | 0.93 | 328257 | 42311 | 285946 | FALSE |
| 157 | Pancreatic cancer | Neoplasms | 1.01 | 0.88 | 1.12 | 0.93 | 312950 | 602 | 312348 | FALSE |
| 261.4 | Vitamin D deficiency | Endocrine/Metabolic | 0.99 | 0.87 | 1.16 | 0.93 | 326211 | 408 | 325803 | FALSE |
| 964.1 | Anticoagulants causing adverse effects | Injuries & Poisonings | 0.99 | 0.84 | 1.21 | 0.93 | 300487 | 259 | 300228 | FALSE |
| 540.11 | Acute appendicitis | Digestive | 1.00 | 0.94 | 1.06 | 0.93 | 327054 | 2658 | 324396 | FALSE |
| 426.91 | Cardiac pacemaker in situ | Circulatory System | 1.00 | 0.94 | 1.07 | 0.93 | 301854 | 2316 | 299538 | FALSE |
| 537 | Other disorders of stomach and duodenum | Digestive | 1.00 | 0.95 | 1.05 | 0.93 | 300011 | 3483 | 296528 | FALSE |
| 557 | Intestinal malabsorption (non-celiac) | Digestive | 1.01 | 0.84 | 1.17 | 0.93 | 258707 | 294 | 258413 | FALSE |
| 433.5 | Cerebral aneurysm | Circulatory System | 0.99 | 0.87 | 1.16 | 0.93 | 318522 | 398 | 318124 | FALSE |
| 394.3 | Aortic valve disease | Circulatory System | 1.00 | 0.92 | 1.08 | 0.93 | 322853 | 1308 | 321545 | FALSE |
| 112 | Candidiasis | Infectious Diseases | 1.00 | 0.94 | 1.06 | 0.93 | 327667 | 2150 | 325517 | FALSE |
| 473.4 | Voice disturbance | Respiratory | 1.00 | 0.91 | 1.09 | 0.94 | 310068 | 1101 | 308967 | FALSE |
| 244.4 | Hypothyroidism NOS | Endocrine/Metabolic | 1.00 | 0.97 | 1.02 | 0.94 | 324776 | 14430 | 310346 | FALSE |
| 344 | Other paralytic syndromes | Neurological | 1.00 | 0.90 | 1.12 | 0.94 | 286858 | 689 | 286169 | FALSE |
| 938.2 | Chronic dermatitis due to solar radiation | Injuries & Poisonings | 1.01 | 0.83 | 1.19 | 0.94 | 322187 | 264 | 321923 | FALSE |
| 700 | Corns and callosities | Dermatologic | 0.99 | 0.85 | 1.19 | 0.94 | 323369 | 288 | 323081 | FALSE |
| 571.8 | Liver abscess and sequelae of chronic liver disease | Digestive | 1.00 | 0.89 | 1.13 | 0.94 | 318921 | 573 | 318348 | FALSE |
| 290.2 | Delirium due to conditions classified elsewhere | Mental Disorders | 1.00 | 0.90 | 1.12 | 0.94 | 322168 | 681 | 321487 | FALSE |
| 285.1 | Acute posthemorrhagic anemia | Hematopoietic | 1.01 | 0.83 | 1.19 | 0.95 | 309184 | 267 | 308917 | FALSE |
| 345.12 | Partial epilepsy | Neurological | 1.01 | 0.84 | 1.18 | 0.95 | 286467 | 298 | 286169 | FALSE |
| 636.3 | Hemorrhage in early pregnancy | Pregnancy Complications | 1.00 | 0.91 | 1.09 | 0.95 | 321095 | 1113 | 319982 | FALSE |
| 747.11 | Cardiac shunt/ heart septal defect | Congenital Anomalies | 1.00 | 0.88 | 1.12 | 0.95 | 325952 | 581 | 325371 | FALSE |
| 184.1 | Malignant neoplasm of ovary and other uterine adnexa | Neoplasms | 1.00 | 0.94 | 1.06 | 0.95 | 308123 | 2553 | 305570 | FALSE |
| 427.42 | Cardiac arrest | Circulatory System | 1.00 | 0.91 | 1.10 | 0.95 | 300487 | 949 | 299538 | FALSE |
| 709.2 | Sicca syndrome | Dermatologic | 1.00 | 0.88 | 1.13 | 0.95 | 242503 | 520 | 241983 | FALSE |
| 621 | Endometrial hyperplasia | Genitourinary | 1.00 | 0.92 | 1.09 | 0.95 | 316478 | 1154 | 315324 | FALSE |
| 481 | Influenza | Respiratory | 1.00 | 0.97 | 1.03 | 0.95 | 327723 | 10017 | 317706 | FALSE |

| | | | | | | | | | | |
|---|---|---|---|---|---|---|---|---|---|---|
| 261.2 | Vitamin B-complex deficiencies | Endocrine/Metabolic | 1.00 | 0.90 | 1.11 | 0.95 | 326576 | 773 | 325803 | FALSE |
| 483 | Acute bronchitis and bronchiolitis | Respiratory | 1.01 | 0.81 | 1.22 | 0.95 | 317906 | 200 | 317706 | FALSE |
| 857 | Mechanical complication of unspecified genitourinary device, implant, and graft | Injuries & Poisonings | 1.00 | 0.92 | 1.08 | 0.95 | 315265 | 1284 | 313981 | FALSE |
| 220 | Benign neoplasm of ovary | Neoplasms | 1.00 | 0.93 | 1.08 | 0.96 | 297563 | 1490 | 296073 | FALSE |
| 251.1 | Hypoglycemia | Endocrine/Metabolic | 1.00 | 0.91 | 1.09 | 0.96 | 305969 | 977 | 304992 | FALSE |
| 571 | Chronic liver disease and cirrhosis | Digestive | 1.00 | 0.89 | 1.14 | 0.96 | 318888 | 540 | 318348 | FALSE |
| 634.3 | Ectopic pregnancy | Pregnancy Complications | 1.00 | 0.85 | 1.17 | 0.96 | 320324 | 342 | 319982 | FALSE |
| 803.2 | Fracture of radius and ulna | Injuries & Poisonings | 1.00 | 0.91 | 1.11 | 0.96 | 324458 | 884 | 323574 | FALSE |
| 53 | Herpes zoster | Infectious Diseases | 1.00 | 0.85 | 1.16 | 0.96 | 322564 | 341 | 322223 | FALSE |
| 619 | Noninflammatory female genital disorders | Genitourinary | 1.00 | 0.99 | 1.02 | 0.96 | 328123 | 49558 | 278565 | FALSE |
| 292.3 | Memory loss | Mental Disorders | 1.00 | 0.90 | 1.12 | 0.96 | 322225 | 738 | 321487 | FALSE |
| 803.3 | Fracture of clavicle or scapula | Injuries & Poisonings | 1.00 | 0.85 | 1.19 | 0.96 | 323874 | 300 | 323574 | FALSE |
| 297.2 | Suicide or self-inflicted injury | Mental Disorders | 1.00 | 0.95 | 1.06 | 0.96 | 285672 | 2757 | 282915 | FALSE |
| 296.1 | Bipolar | Mental Disorders | 1.00 | 0.92 | 1.09 | 0.96 | 284036 | 1121 | 282915 | FALSE |
| 353 | Nerve root and plexus disorders | Neurological | 1.00 | 0.91 | 1.10 | 0.97 | 314022 | 994 | 313028 | FALSE |
| 444 | Arterial embolism and thrombosis | Circulatory System | 1.00 | 0.87 | 1.15 | 0.97 | 319811 | 434 | 319377 | FALSE |
| 694.2 | Other dyschromia | Dermatologic | 1.00 | 0.90 | 1.11 | 0.97 | 322566 | 800 | 321766 | FALSE |
| 599.3 | Dysuria | Genitourinary | 1.00 | 0.92 | 1.08 | 0.97 | 221653 | 1209 | 220444 | FALSE |
| 335 | Multiple sclerosis | Neurological | 1.00 | 0.92 | 1.08 | 0.97 | 287548 | 1379 | 286169 | FALSE |
| 365 | Glaucoma | Sense Organs | 1.00 | 0.96 | 1.04 | 0.98 | 321357 | 4558 | 316799 | FALSE |
| 368 | Visual disturbances | Sense Organs | 1.00 | 0.92 | 1.09 | 0.98 | 325980 | 1081 | 324899 | FALSE |
| 454.11 | Varicose veins of lower extremity, symptomtic | Circulatory System | 1.00 | 0.89 | 1.12 | 0.98 | 288382 | 652 | 287730 | FALSE |
| 613.9 | Breast disorder NOS | Genitourinary | 1.00 | 0.96 | 1.04 | 0.98 | 327954 | 5519 | 322435 | FALSE |
| 740.1 | Osteoarthritis; localized | Musculoskeletal | 1.00 | 0.97 | 1.03 | 0.98 | 315814 | 9472 | 306342 | FALSE |
| 362.29 | Macular degeneration (senile) of retina NOS | Sense Organs | 1.00 | 0.94 | 1.06 | 0.98 | 318069 | 2200 | 315869 | FALSE |
| 519.8 | Other diseases of respiratory system, NEC | Respiratory | 1.00 | 0.97 | 1.03 | 0.98 | 274546 | 9082 | 265464 | FALSE |
| 977 | Personal history of allergy to medicinal agents | Injuries & Poisonings | 1.00 | 0.83 | 1.20 | 0.98 | 300466 | 238 | 300228 | FALSE |
| 362.2 | Degeneration of macula and posterior pole of retina | Sense Organs | 1.00 | 0.94 | 1.06 | 0.98 | 318072 | 2203 | 315869 | FALSE |
| 252.1 | Hyperparathyroidism | Endocrine/Metabolic | 1.00 | 0.90 | 1.11 | 0.99 | 325376 | 814 | 324562 | FALSE |
| 428.2 | Heart failure NOS | Circulatory System | 1.00 | 0.96 | 1.04 | 0.99 | 326384 | 4334 | 322050 | FALSE |
| 835 | Internal derangement of knee | Injuries & Poisonings | 1.00 | 0.96 | 1.05 | 0.99 | 326029 | 4239 | 321790 | FALSE |
| 729 | Other disorders of soft tissues | Musculoskeletal | 1.00 | 0.98 | 1.02 | 0.99 | 327495 | 22524 | 304971 | FALSE |
| 586 | Other disorders of the kidney and ureters | Genitourinary | 1.00 | 0.95 | 1.05 | 0.99 | 317507 | 3411 | 314096 | FALSE |
| 464 | Acute sinusitis | Respiratory | 1.00 | 0.82 | 1.22 | 0.99 | 325404 | 208 | 325196 | FALSE |
| 960 | Poisoning by antibiotics | Injuries & Poisonings | 1.00 | 0.95 | 1.05 | 1.00 | 303756 | 3528 | 300228 | FALSE |
| 578 | Gastrointestinal hemorrhage | Digestive | 1.00 | 0.84 | 1.19 | 1.00 | 303696 | 285 | 303411 | FALSE |
| 433.2 | Occlusion of cerebral arteries | Circulatory System | 1.00 | 0.95 | 1.05 | 1.00 | 321104 | 2980 | 318124 | FALSE |

| Supplementary Table 11. Phenome-wide association study (PheWAS) results for the main beta-blocker (BB) genetic risk score. | | | | | | | | | | |
|---|---|---|---|---|---|---|---|---|---|---|
| Phecode | Trait | Category | OR | Low 95% CI | Upp 95% CI | P value | Total sample size | Cases | Controls | FDR |
| 401.1 | Essential hypertension | Circulatory System | 0.95 | 0.95 | 0.96 | 2.06E-28 | 327983 | 79235 | 248748 | TRUE |
| 401 | Hypertension | Circulatory System | 0.95 | 0.95 | 0.96 | 2.16E-28 | 328239 | 79491 | 248748 | TRUE |
| 459.9 | Circulatory disease NEC | Circulatory System | 0.97 | 0.97 | 0.98 | 1.89E-13 | 327928 | 133749 | 194179 | TRUE |
| 427.2 | Atrial fibrillation and flutter | Circulatory System | 0.97 | 0.95 | 0.99 | 1.99E-04 | 314573 | 15052 | 299521 | TRUE |
| 916 | Contusion | Injuries & Poisonings | 0.91 | 0.87 | 0.96 | 4.33E-04 | 328240 | 1478 | 326762 | FALSE |
| 395.1 | Nonrheumatic mitral valve disorders | Circulatory System | 0.94 | 0.91 | 0.97 | 7.14E-04 | 324497 | 2969 | 321528 | FALSE |
| 189.21 | Malignant neoplasm of bladder | Neoplasms | 0.93 | 0.89 | 0.97 | 7.48E-04 | 326150 | 2188 | 323962 | FALSE |
| 394.2 | Mitral valve disease | Circulatory System | 0.94 | 0.91 | 0.97 | 7.65E-04 | 324603 | 3075 | 321528 | FALSE |
| 289.3 | Personal history of diseases of blood and blood-forming organs | Hematopoietic | 0.82 | 0.74 | 0.92 | 8.13E-04 | 320615 | 297 | 320318 | FALSE |
| 427 | Cardiac dysrhythmias | Circulatory System | 0.83 | 0.75 | 0.93 | 1.10E-03 | 299844 | 323 | 299521 | FALSE |
| 411.8 | Other chronic ischemic heart disease, unspecified | Circulatory System | 0.98 | 0.97 | 0.99 | 1.19E-03 | 326969 | 31280 | 295689 | FALSE |
| 411 | Ischemic Heart Disease | Circulatory System | 0.98 | 0.97 | 0.99 | 1.25E-03 | 327032 | 31343 | 295689 | FALSE |
| 250 | Diabetes mellitus | Endocrine/Metabolic | 1.02 | 1.01 | 1.04 | 1.68E-03 | 327730 | 20240 | 307490 | FALSE |
| 250.2 | Type 2 diabetes | Endocrine/Metabolic | 1.02 | 1.01 | 1.04 | 1.70E-03 | 326603 | 19113 | 307490 | FALSE |
| 411.4 | Coronary atherosclerosis | Circulatory System | 0.98 | 0.97 | 0.99 | 1.71E-03 | 327242 | 31553 | 295689 | FALSE |
| 738.4 | Acquired spondylolisthesis | Musculoskeletal | 1.22 | 1.07 | 1.38 | 2.14E-03 | 316942 | 255 | 316687 | FALSE |
| 747.13 | Congenital anomalies of great vessels | Congenital Anomalies | 0.93 | 0.89 | 0.98 | 2.99E-03 | 327177 | 1823 | 325354 | FALSE |
| 371.1 | Uveitis, noninfectious or NOS | Sense Organs | 1.14 | 1.04 | 1.24 | 4.43E-03 | 318886 | 498 | 318388 | FALSE |
| 575.1 | Cholangitis | Digestive | 1.13 | 1.03 | 1.24 | 0.01 | 310820 | 497 | 310323 | FALSE |
| 380 | Disorders of external ear | Sense Organs | 0.93 | 0.89 | 0.98 | 0.01 | 328227 | 1475 | 326752 | FALSE |
| 395.6 | Heart valve replaced | Circulatory System | 0.93 | 0.89 | 0.98 | 0.01 | 323056 | 1528 | 321528 | FALSE |
| 528 | Diseases of the oral soft tissues, excluding lesions specific for gingiva and tongue | Digestive | 0.94 | 0.90 | 0.99 | 0.01 | 324593 | 2059 | 322534 | FALSE |
| 850 | Hemorrhage or hematoma complicating a procedure | Injuries & Poisonings | 0.97 | 0.94 | 0.99 | 0.01 | 319328 | 5364 | 313964 | FALSE |
| 277 | Other disorders of metabolism | Endocrine/Metabolic | 0.99 | 0.98 | 1.00 | 0.01 | 328230 | 45303 | 282927 | FALSE |
| 189.4 | Malignant neoplasm of other urinary organs | Neoplasms | 0.96 | 0.92 | 0.99 | 0.01 | 327185 | 3223 | 323962 | FALSE |
| 857 | Mechanical complication of unspecified genitourinary device, implant, and graft | Injuries & Poisonings | 0.93 | 0.88 | 0.98 | 0.01 | 315248 | 1284 | 313964 | FALSE |
| 586.2 | Cyst of kidney, acquired | Genitourinary | 0.93 | 0.88 | 0.98 | 0.01 | 315372 | 1293 | 314079 | FALSE |
| 276.12 | Hyposmolality and/or hyponatremia | Endocrine/Metabolic | 0.94 | 0.90 | 0.99 | 0.01 | 322496 | 1852 | 320644 | FALSE |
| 550.3 | Femoral hernia | Digestive | 0.91 | 0.84 | 0.98 | 0.01 | 280442 | 653 | 279789 | FALSE |
| 634.3 | Ectopic pregnancy | Pregnancy Complications | 0.87 | 0.78 | 0.97 | 0.01 | 320307 | 342 | 319965 | FALSE |
| 704 | Diseases of hair and hair follicles | Dermatologic | 1.04 | 1.01 | 1.06 | 0.01 | 326692 | 5263 | 321429 | FALSE |
| 702.1 | Actinic keratosis | Dermatologic | 1.05 | 1.01 | 1.09 | 0.01 | 325264 | 2664 | 322600 | FALSE |
| 803.2 | Fracture of radius and ulna | Injuries & Poisonings | 0.92 | 0.86 | 0.98 | 0.01 | 324441 | 884 | 323557 | FALSE |
| 225.1 | Benign neoplasm of brain, cranial nerves, meninges | Neoplasms | 0.92 | 0.86 | 0.98 | 0.01 | 327267 | 820 | 326447 | FALSE |
| 613.1 | Inflammatory disease of breast | Genitourinary | 1.09 | 1.02 | 1.18 | 0.02 | 323156 | 738 | 322418 | FALSE |
| 180.3 | Cervical intraepithelial neoplasia [CIN] [Cervical dysplasia] | Neoplasms | 0.95 | 0.91 | 0.99 | 0.02 | 299736 | 2153 | 297583 | FALSE |

| | | | | | | | | | | |
|---|---|---|---|---|---|---|---|---|---|---|
| 747 | Cardiac and circulatory congenital anomalies | Congenital Anomalies | 0.93 | 0.88 | 0.99 | 0.02 | 326576 | 1222 | 325354 | FALSE |
| 649.1 | Diabetes or abnormal glucose tolerance complicating pregnancy | Pregnancy Complications | 1.18 | 1.03 | 1.36 | 0.02 | 328127 | 208 | 327919 | FALSE |
| 623 | Hypertrophy of female genital organs | Genitourinary | 1.07 | 1.01 | 1.13 | 0.02 | 316579 | 1272 | 315307 | FALSE |
| 747.12 | Valvular heart disease/ heart chambers | Congenital Anomalies | 0.86 | 0.76 | 0.98 | 0.02 | 325592 | 238 | 325354 | FALSE |
| 293 | Symptoms involving head and neck | Mental Disorders | 1.05 | 1.01 | 1.10 | 0.02 | 327323 | 2204 | 325119 | FALSE |
| 149.4 | Cancer of larynx | Neoplasms | 0.87 | 0.77 | 0.98 | 0.02 | 326307 | 266 | 326041 | FALSE |
| 260.6 | Anorexia | Endocrine/Metabolic | 0.93 | 0.87 | 0.99 | 0.02 | 326636 | 850 | 325786 | FALSE |
| 594.1 | Calculus of kidney | Genitourinary | 0.96 | 0.93 | 0.99 | 0.02 | 323265 | 3334 | 319931 | FALSE |
| 471 | Nasal polyps | Respiratory | 1.04 | 1.00 | 1.08 | 0.03 | 312342 | 3392 | 308950 | FALSE |
| 427.9 | Palpitations | Circulatory System | 0.97 | 0.94 | 1.00 | 0.03 | 303480 | 3959 | 299521 | FALSE |
| 636.3 | Hemorrhage in early pregnancy | Pregnancy Complications | 0.94 | 0.88 | 0.99 | 0.03 | 321078 | 1113 | 319965 | FALSE |
| 574.2 | Calculus of bile duct | Digestive | 1.04 | 1.00 | 1.08 | 0.03 | 313011 | 2688 | 310323 | FALSE |
| 989 | Toxic effect of other substances, chiefly nonmedicinal as to source | Injuries & Poisonings | 1.06 | 1.01 | 1.12 | 0.03 | 328136 | 1438 | 326698 | FALSE |
| 414 | Other forms of chronic heart disease | Circulatory System | 0.95 | 0.91 | 1.00 | 0.03 | 297457 | 1768 | 295689 | FALSE |
| 250.1 | Type 1 diabetes | Endocrine/Metabolic | 1.04 | 1.00 | 1.08 | 0.03 | 310189 | 2699 | 307490 | FALSE |
| 297.2 | Suicide or self-inflicted injury | Mental Disorders | 1.04 | 1.00 | 1.08 | 0.03 | 285655 | 2757 | 282898 | FALSE |
| 202.21 | Nodular lymphoma | Neoplasms | 0.90 | 0.81 | 0.99 | 0.03 | 323913 | 390 | 323523 | FALSE |
| 756 | Other congenital musculoskeletal anomalies | Congenital Anomalies | 1.08 | 1.01 | 1.16 | 0.03 | 328123 | 812 | 327311 | FALSE |
| 348.8 | Encephalopathy, not elsewhere classified | Neurological | 1.16 | 1.01 | 1.34 | 0.03 | 286356 | 204 | 286152 | FALSE |
| 550.4 | Umbilical hernia | Digestive | 0.97 | 0.94 | 1.00 | 0.04 | 283693 | 3904 | 279789 | FALSE |
| 411.41 | Aneurysm and dissection of heart | Circulatory System | 0.92 | 0.86 | 0.99 | 0.04 | 296410 | 721 | 295689 | FALSE |
| 571.6 | Primary biliary cirrhosis | Digestive | 1.14 | 1.01 | 1.29 | 0.04 | 318589 | 258 | 318331 | FALSE |
| 740.2 | Osteoarthrosis, generalized | Musculoskeletal | 0.92 | 0.85 | 1.00 | 0.04 | 306914 | 589 | 306325 | FALSE |
| 706.2 | Sebaceous cyst | Dermatologic | 1.02 | 1.00 | 1.04 | 0.04 | 327401 | 9107 | 318294 | FALSE |
| 555.2 | Ulcerative colitis | Digestive | 0.96 | 0.93 | 1.00 | 0.04 | 261550 | 3154 | 258396 | FALSE |
| 291.8 | Alteration of consciousness | Mental Disorders | 1.10 | 1.00 | 1.21 | 0.04 | 321904 | 434 | 321470 | FALSE |
| 535 | Gastritis and duodenitis | Digestive | 1.02 | 1.00 | 1.03 | 0.04 | 314259 | 17748 | 296511 | FALSE |
| 562.1 | Diverticulosis | Digestive | 0.99 | 0.97 | 1.00 | 0.04 | 286281 | 27885 | 258396 | FALSE |
| 151 | Cancer of stomach | Neoplasms | 0.92 | 0.85 | 1.00 | 0.04 | 312905 | 574 | 312331 | FALSE |
| 555.21 | Ulcerative colitis (chronic) | Digestive | 1.09 | 1.00 | 1.18 | 0.05 | 258967 | 571 | 258396 | FALSE |
| 416 | Cardiomegaly | Circulatory System | 0.96 | 0.93 | 1.00 | 0.05 | 324157 | 2610 | 321547 | FALSE |
| 184.11 | Malignant neoplasm of ovary | Neoplasms | 0.97 | 0.95 | 1.00 | 0.05 | 310562 | 5009 | 305553 | FALSE |
| 977 | Personal history of allergy to medicinal agents | Injuries & Poisonings | 1.14 | 1.00 | 1.30 | 0.05 | 300449 | 238 | 300211 | FALSE |
| 427.3 | Other specified cardiac dysrhythmias | Circulatory System | 0.97 | 0.93 | 1.00 | 0.05 | 302836 | 3315 | 299521 | FALSE |
| 426.4 | Anomalous atrioventricular excitation | Circulatory System | 0.88 | 0.78 | 1.00 | 0.05 | 299761 | 240 | 299521 | FALSE |
| 440.2 | Atherosclerosis of the extremities | Circulatory System | 0.92 | 0.85 | 1.00 | 0.05 | 319912 | 552 | 319360 | FALSE |
| 804 | Fracture of hand or wrist | Injuries & Poisonings | 1.08 | 1.00 | 1.16 | 0.05 | 324232 | 675 | 323557 | FALSE |
| 426.9 | Cardiac pacemaker/device in situ | Circulatory System | 1.14 | 1.00 | 1.29 | 0.05 | 299758 | 237 | 299521 | FALSE |

| | | | | | | | | | | |
|---|---|---|---|---|---|---|---|---|---|---|
| 362.4 | Retinal vascular changes and abnomalities | Sense Organs | 1.07 | 1.00 | 1.14 | 0.05 | 316733 | 881 | 315852 | FALSE |
| 303.3 | Psychogenic disorder | Mental Disorders | 1.07 | 1.00 | 1.14 | 0.05 | 283739 | 841 | 282898 | FALSE |
| 766 | Neuralgia, neuritis, and radiculitis NOS | Symptoms | 1.12 | 1.00 | 1.25 | 0.06 | 326987 | 304 | 326683 | FALSE |
| 642.1 | Preeclampsia and eclampsia | Pregnancy Complications | 0.90 | 0.81 | 1.00 | 0.06 | 327385 | 335 | 327050 | FALSE |
| 938 | Dermatitis due to solar radiation | Injuries & Poisonings | 1.04 | 1.00 | 1.08 | 0.06 | 324444 | 2538 | 321906 | FALSE |
| 380.4 | Impacted cerumen | Sense Organs | 0.91 | 0.82 | 1.00 | 0.06 | 327106 | 354 | 326752 | FALSE |
| 317.11 | Alcoholic liver damage | Mental Disorders | 1.07 | 1.00 | 1.14 | 0.06 | 298534 | 865 | 297669 | FALSE |
| 687.4 | Disturbance of skin sensation | Dermatologic | 0.97 | 0.93 | 1.00 | 0.07 | 325793 | 2979 | 322814 | FALSE |
| 411.3 | Angina pectoris | Circulatory System | 0.99 | 0.97 | 1.00 | 0.07 | 312048 | 16359 | 295689 | FALSE |
| 90 | Sexually transmitted infections (not HIV or hepatitis) | Infectious Diseases | 0.90 | 0.80 | 1.01 | 0.07 | 328239 | 298 | 327941 | FALSE |
| 53 | Herpes zoster | Infectious Diseases | 0.91 | 0.81 | 1.01 | 0.07 | 322547 | 341 | 322206 | FALSE |
| 295.1 | Schizophrenia | Mental Disorders | 1.08 | 0.99 | 1.17 | 0.07 | 283509 | 611 | 282898 | FALSE |
| 596.1 | Bladder neck obstruction | Genitourinary | 0.96 | 0.92 | 1.00 | 0.07 | 315665 | 2029 | 313636 | FALSE |
| 187.2 | Malignant neoplasm of testis | Neoplasms | 0.97 | 0.95 | 1.00 | 0.07 | 323790 | 4572 | 319218 | FALSE |
| 333.4 | Torsion dystonia | Neurological | 1.12 | 0.99 | 1.26 | 0.07 | 286417 | 265 | 286152 | FALSE |
| 208 | Benign neoplasm of colon | Neoplasms | 0.99 | 0.97 | 1.00 | 0.07 | 325437 | 20827 | 304610 | FALSE |
| 737 | Curvature of spine | Musculoskeletal | 1.07 | 0.99 | 1.15 | 0.07 | 317430 | 743 | 316687 | FALSE |
| 444.1 | Arterial embolism and thrombosis of lower extremity artery | Circulatory System | 0.93 | 0.85 | 1.01 | 0.07 | 319930 | 570 | 319360 | FALSE |
| 747.11 | Cardiac shunt/ heart septal defect | Congenital Anomalies | 0.93 | 0.86 | 1.01 | 0.08 | 325935 | 581 | 325354 | FALSE |
| 524.3 | Anomalies of tooth position/malocclusion | Digestive | 0.90 | 0.80 | 1.01 | 0.08 | 311953 | 295 | 311658 | FALSE |
| 871 | Open wounds of extremities | Injuries & Poisonings | 1.03 | 1.00 | 1.06 | 0.08 | 323873 | 4142 | 319731 | FALSE |
| 272.11 | Hypercholesterolemia | Endocrine/Metabolic | 0.99 | 0.98 | 1.00 | 0.08 | 325499 | 34002 | 291497 | FALSE |
| 586 | Other disorders of the kidney and ureters | Genitourinary | 0.97 | 0.94 | 1.00 | 0.08 | 317490 | 3411 | 314079 | FALSE |
| 8 | Intestinal infection | Infectious Diseases | 0.98 | 0.96 | 1.00 | 0.08 | 328240 | 9161 | 319079 | FALSE |
| 158 | Neoplasm of unspecified nature of digestive system | Neoplasms | 1.05 | 0.99 | 1.12 | 0.08 | 313429 | 1098 | 312331 | FALSE |
| 840 | Sprains and strains | Injuries & Poisonings | 1.08 | 0.99 | 1.19 | 0.08 | 328201 | 481 | 327720 | FALSE |
| 569.2 | Gastrointestinal complications | Digestive | 1.08 | 0.99 | 1.19 | 0.09 | 247132 | 472 | 246660 | FALSE |
| 755.1 | Congenital deformities of feet | Congenital Anomalies | 1.12 | 0.98 | 1.28 | 0.09 | 327531 | 220 | 327311 | FALSE |
| 184.1 | Malignant neoplasm of ovary and other uterine adnexa | Neoplasms | 0.97 | 0.93 | 1.01 | 0.09 | 308106 | 2553 | 305553 | FALSE |
| 8.5 | Bacterial enteritis | Infectious Diseases | 0.96 | 0.92 | 1.01 | 0.09 | 321188 | 2109 | 319079 | FALSE |
| 342 | Hemiplegia | Neurological | 0.96 | 0.91 | 1.01 | 0.09 | 287677 | 1525 | 286152 | FALSE |
| 394.7 | Disease of tricuspid valve | Circulatory System | 0.95 | 0.89 | 1.01 | 0.09 | 322614 | 1086 | 321528 | FALSE |
| 331.9 | Cerebral degeneration, unspecified | Neurological | 1.09 | 0.99 | 1.20 | 0.09 | 286569 | 417 | 286152 | FALSE |
| 509.8 | Dependence on respirator [Ventilator] or supplemental oxygen | Respiratory | 1.09 | 0.99 | 1.20 | 0.09 | 316885 | 401 | 316484 | FALSE |
| 783 | Fever of unknown origin | Symptoms | 1.03 | 1.00 | 1.06 | 0.09 | 328240 | 4111 | 324129 | FALSE |
| 256.4 | Polycystic ovaries | Endocrine/Metabolic | 1.12 | 0.98 | 1.28 | 0.09 | 324764 | 219 | 324545 | FALSE |
| 595 | Hydronephrosis | Genitourinary | 0.96 | 0.92 | 1.01 | 0.09 | 321941 | 2010 | 319931 | FALSE |
| 577 | Diseases of pancreas | Digestive | 0.93 | 0.86 | 1.01 | 0.10 | 326478 | 558 | 325920 | FALSE |

| | | | | | | | | | | |
|---|---|---|---|---|---|---|---|---|---|---|
| 647.1 | Infections of genitourinary tract during pregnancy | Pregnancy Complications | 1.11 | 0.98 | 1.24 | 0.10 | 328128 | 284 | 327844 | FALSE |
| 722.6 | Degeneration of intervertebral disc | Musculoskeletal | 0.97 | 0.93 | 1.01 | 0.10 | 313866 | 2922 | 310944 | FALSE |
| 189 | Cancer of urinary organs (incl. kidney and bladder) | Neoplasms | 0.97 | 0.94 | 1.01 | 0.10 | 326965 | 3003 | 323962 | FALSE |
| 613.9 | Breast disorder NOS | Genitourinary | 1.02 | 1.00 | 1.05 | 0.10 | 327937 | 5519 | 322418 | FALSE |
| 577.1 | Acute pancreatitis | Digestive | 0.96 | 0.91 | 1.01 | 0.10 | 327437 | 1517 | 325920 | FALSE |
| 627.2 | Symptomatic menopause | Genitourinary | 0.89 | 0.78 | 1.02 | 0.10 | 296434 | 203 | 296231 | FALSE |
| 578.8 | Hemorrhage of rectum and anus | Digestive | 0.99 | 0.97 | 1.00 | 0.10 | 317220 | 13826 | 303394 | FALSE |
| 180.1 | Cervical cancer | Neoplasms | 0.98 | 0.95 | 1.00 | 0.10 | 302137 | 4554 | 297583 | FALSE |
| 513.4 | Hyperventilation | Respiratory | 1.10 | 0.98 | 1.24 | 0.10 | 327904 | 280 | 327624 | FALSE |
| 703 | Diseases of nail, NOS | Dermatologic | 1.09 | 0.98 | 1.21 | 0.10 | 321795 | 366 | 321429 | FALSE |
| 415.21 | Primary pulmonary hypertension | Circulatory System | 0.93 | 0.85 | 1.02 | 0.10 | 322013 | 466 | 321547 | FALSE |
| 578 | Gastrointestinal hemorrhage | Digestive | 0.91 | 0.81 | 1.02 | 0.11 | 303679 | 285 | 303394 | FALSE |
| 350.3 | Lack of coordination | Neurological | 0.92 | 0.83 | 1.02 | 0.11 | 325804 | 360 | 325444 | FALSE |
| 585.31 | Renal dialysis | Genitourinary | 0.93 | 0.86 | 1.02 | 0.11 | 314603 | 524 | 314079 | FALSE |
| 386.1 | Meniere's disease | Sense Organs | 1.07 | 0.98 | 1.16 | 0.11 | 322213 | 564 | 321649 | FALSE |
| 733 | Other disorders of bone and cartilage | Musculoskeletal | 1.07 | 0.98 | 1.16 | 0.11 | 316001 | 569 | 315432 | FALSE |
| 374.3 | Ptosis of eyelid | Sense Organs | 1.04 | 0.99 | 1.09 | 0.11 | 320284 | 1896 | 318388 | FALSE |
| 365.2 | Primary angle-closure glaucoma | Sense Organs | 0.94 | 0.88 | 1.01 | 0.12 | 317491 | 709 | 316782 | FALSE |
| 364.5 | Corneal dystrophy | Sense Organs | 1.11 | 0.97 | 1.27 | 0.12 | 316998 | 216 | 316782 | FALSE |
| 112 | Candidiasis | Infectious Diseases | 0.97 | 0.93 | 1.01 | 0.12 | 327650 | 2150 | 325500 | FALSE |
| 261.4 | Vitamin D deficiency | Endocrine/Metabolic | 0.93 | 0.84 | 1.02 | 0.12 | 326194 | 408 | 325786 | FALSE |
| 159 | Malignant neoplasm of other and ill-defined sites within the digestive organs and peritoneum | Neoplasms | 0.98 | 0.95 | 1.01 | 0.12 | 317620 | 5289 | 312331 | FALSE |
| 250.23 | Type 2 diabetes with ophthalmic manifestations | Endocrine/Metabolic | 1.04 | 0.99 | 1.10 | 0.12 | 308834 | 1344 | 307490 | FALSE |
| 201 | Hodgkin's disease | Neoplasms | 1.10 | 0.97 | 1.24 | 0.12 | 323794 | 271 | 323523 | FALSE |
| 426.3 | Bundle branch block | Circulatory System | 0.91 | 0.81 | 1.02 | 0.12 | 299812 | 291 | 299521 | FALSE |
| 80 | Postoperative infection | Infectious Diseases | 0.98 | 0.95 | 1.01 | 0.12 | 326101 | 4622 | 321479 | FALSE |
| 473.3 | Paralysis/spasm of vocal cords or larynx | Respiratory | 1.10 | 0.97 | 1.24 | 0.13 | 309217 | 267 | 308950 | FALSE |
| 586.4 | Stricture/obstruction of ureter | Genitourinary | 0.95 | 0.89 | 1.01 | 0.13 | 315018 | 939 | 314079 | FALSE |
| 472 | Chronic pharyngitis and nasopharyngitis | Respiratory | 1.05 | 0.99 | 1.12 | 0.13 | 309897 | 947 | 308950 | FALSE |
| 381.9 | Otorrhea | Sense Organs | 0.91 | 0.81 | 1.03 | 0.13 | 324420 | 277 | 324143 | FALSE |
| 374 | Other disorders of eyelids | Sense Organs | 0.97 | 0.94 | 1.01 | 0.13 | 321593 | 3205 | 318388 | FALSE |
| 411.2 | Myocardial infarction | Circulatory System | 0.99 | 0.97 | 1.00 | 0.13 | 307658 | 11969 | 295689 | FALSE |
| 357 | Inflammatory and toxic neuropathy | Neurological | 1.04 | 0.99 | 1.10 | 0.13 | 327375 | 1423 | 325952 | FALSE |
| 364 | Corneal opacity and other disorders of cornea | Sense Organs | 1.11 | 0.97 | 1.26 | 0.13 | 317009 | 227 | 316782 | FALSE |
| 446.9 | Arteritis NOS | Circulatory System | 0.91 | 0.80 | 1.03 | 0.13 | 319605 | 245 | 319360 | FALSE |
| 477 | Epistaxis or throat hemorrhage | Respiratory | 0.97 | 0.93 | 1.01 | 0.13 | 311469 | 2519 | 308950 | FALSE |
| 858 | Complication of internal orthopedic device | Injuries & Poisonings | 1.03 | 0.99 | 1.06 | 0.13 | 317147 | 3183 | 313964 | FALSE |
| 504 | Other alveolar and parietoalveolar pneumonopathy | Respiratory | 1.09 | 0.97 | 1.22 | 0.13 | 316793 | 309 | 316484 | FALSE |

| | | | | | | | | | | |
|---|---|---|---|---|---|---|---|---|---|---|
| 728.71 | Contracture of palmar fascia [Dupuytren's disease] | Musculoskeletal | 0.97 | 0.93 | 1.01 | 0.13 | 307403 | 2449 | 304954 | FALSE |
| 290.2 | Delirium due to conditions classified elsewhere | Mental Disorders | 0.94 | 0.88 | 1.02 | 0.14 | 322151 | 681 | 321470 | FALSE |
| 729 | Other disorders of soft tissues | Musculoskeletal | 0.99 | 0.98 | 1.00 | 0.14 | 327478 | 22524 | 304954 | FALSE |
| 368 | Visual disturbances | Sense Organs | 1.05 | 0.99 | 1.11 | 0.14 | 325963 | 1081 | 324882 | FALSE |
| 450 | Noninfectious disorders of lymphatic channels | Circulatory System | 0.95 | 0.88 | 1.02 | 0.14 | 328240 | 717 | 327523 | FALSE |
| 182 | Malignant neoplasm of uterus | Neoplasms | 0.96 | 0.91 | 1.01 | 0.14 | 298948 | 1303 | 297645 | FALSE |
| 701.4 | Keloid scar | Dermatologic | 1.10 | 0.97 | 1.26 | 0.14 | 323292 | 228 | 323064 | FALSE |
| 614.4 | Inflammatory diseases of uterus, except cervix | Genitourinary | 0.92 | 0.82 | 1.03 | 0.14 | 319107 | 308 | 318799 | FALSE |
| 732 | Osteochondropathies | Musculoskeletal | 0.91 | 0.79 | 1.03 | 0.14 | 315651 | 219 | 315432 | FALSE |
| 382 | Otalgia | Sense Organs | 0.92 | 0.82 | 1.03 | 0.15 | 324458 | 315 | 324143 | FALSE |
| 520.2 | Disturbances in tooth eruption | Digestive | 1.03 | 0.99 | 1.07 | 0.15 | 314095 | 2437 | 311658 | FALSE |
| 497 | Bronchitis | Respiratory | 0.94 | 0.87 | 1.02 | 0.15 | 294834 | 642 | 294192 | FALSE |
| 344 | Other paralytic syndromes | Neurological | 0.95 | 0.88 | 1.02 | 0.15 | 286841 | 689 | 286152 | FALSE |
| 275.1 | Disorders of iron metabolism | Hematopoietic | 0.95 | 0.88 | 1.02 | 0.15 | 326735 | 700 | 326035 | FALSE |
| 262 | Mineral deficiency NEC | Endocrine/Metabolic | 1.10 | 0.97 | 1.25 | 0.15 | 326027 | 241 | 325786 | FALSE |
| 289.4 | Lymphadenitis | Hematopoietic | 0.97 | 0.94 | 1.01 | 0.15 | 323033 | 2715 | 320318 | FALSE |
| 379.5 | Disorders of iris and ciliary body | Sense Organs | 1.07 | 0.97 | 1.18 | 0.16 | 288604 | 412 | 288192 | FALSE |
| 301 | Personality disorders | Mental Disorders | 1.07 | 0.97 | 1.18 | 0.16 | 283330 | 432 | 282898 | FALSE |
| 191.11 | Cancer of brain | Neoplasms | 0.94 | 0.86 | 1.03 | 0.16 | 326945 | 498 | 326447 | FALSE |
| 374.1 | Ectropion or entropion | Sense Organs | 0.96 | 0.90 | 1.02 | 0.16 | 319480 | 1092 | 318388 | FALSE |
| 304 | Adjustment reaction | Mental Disorders | 1.08 | 0.97 | 1.21 | 0.16 | 283221 | 323 | 282898 | FALSE |
| 296.2 | Depression | Mental Disorders | 1.01 | 0.99 | 1.03 | 0.16 | 295043 | 12145 | 282898 | FALSE |
| 433.3 | Cerebral ischemia | Circulatory System | 0.96 | 0.90 | 1.02 | 0.16 | 319041 | 934 | 318107 | FALSE |
| 150 | Cancer of esophagus | Neoplasms | 0.95 | 0.88 | 1.02 | 0.16 | 313058 | 727 | 312331 | FALSE |
| 474.2 | Chronic tonsillitis and adenoiditis | Respiratory | 1.04 | 0.98 | 1.10 | 0.16 | 310135 | 1185 | 308950 | FALSE |
| 965.1 | Opiates and related narcotics causing adverse effects in therapeutic use | Injuries & Poisonings | 0.96 | 0.91 | 1.02 | 0.16 | 301372 | 1161 | 300211 | FALSE |
| 250.42 | Other abnormal glucose | Endocrine/Metabolic | 1.07 | 0.97 | 1.18 | 0.17 | 307927 | 437 | 307490 | FALSE |
| 296 | Mood disorders | Mental Disorders | 1.01 | 0.99 | 1.03 | 0.17 | 295853 | 12955 | 282898 | FALSE |
| 475 | Chronic sinusitis | Respiratory | 1.03 | 0.99 | 1.07 | 0.17 | 311580 | 2630 | 308950 | FALSE |
| 366.2 | Senile cataract | Sense Organs | 0.98 | 0.96 | 1.01 | 0.17 | 315842 | 8458 | 307384 | FALSE |
| 564.1 | Irritable Bowel Syndrome | Digestive | 1.02 | 0.99 | 1.05 | 0.17 | 264057 | 5661 | 258396 | FALSE |
| 241.1 | Nontoxic uninodular goiter | Endocrine/Metabolic | 0.94 | 0.86 | 1.03 | 0.17 | 310840 | 511 | 310329 | FALSE |
| 388 | Other disorders of ear | Sense Organs | 0.99 | 0.97 | 1.01 | 0.17 | 328080 | 9886 | 318194 | FALSE |
| 476 | Allergic rhinitis | Respiratory | 0.96 | 0.90 | 1.02 | 0.18 | 310025 | 1075 | 308950 | FALSE |
| 741.4 | Joint effusions | Musculoskeletal | 0.92 | 0.82 | 1.04 | 0.18 | 310925 | 283 | 310642 | FALSE |
| 306.9 | Tension headache | Mental Disorders | 1.08 | 0.97 | 1.21 | 0.18 | 283203 | 305 | 282898 | FALSE |
| 70.4 | Chronic hepatitis | Infectious Diseases | 1.08 | 0.96 | 1.22 | 0.18 | 322496 | 290 | 322206 | FALSE |
| 324 | Other CNS infection and poliomyelitis | Neurological | 0.92 | 0.82 | 1.04 | 0.18 | 327426 | 288 | 327138 | FALSE |

| | | | | | | | | | | | |
|---|---|---|---|---|---|---|---|---|---|---|---|
| 426.32 | Left bundle branch block | Circulatory System | 0.97 | 0.92 | 1.01 | 0.18 | 301302 | 1781 | 299521 | FALSE |
| 605 | Erectile dysfunction [ED] | Genitourinary | 0.92 | 0.82 | 1.04 | 0.18 | 308197 | 291 | 307906 | FALSE |
| 608 | Other disorders of male genital organs | Genitourinary | 0.99 | 0.98 | 1.00 | 0.18 | 327998 | 20092 | 307906 | FALSE |
| 292.2 | Mild cognitive impairment | Mental Disorders | 1.10 | 0.96 | 1.26 | 0.18 | 321672 | 202 | 321470 | FALSE |
| 377 | Disorders of optic nerve and visual pathways | Sense Organs | 0.94 | 0.85 | 1.03 | 0.18 | 288628 | 436 | 288192 | FALSE |
| 441.1 | Acute vascular insufficiency of intestine | Circulatory System | 1.08 | 0.96 | 1.22 | 0.18 | 319631 | 271 | 319360 | FALSE |
| 611.3 | Lump or mass in breast | Genitourinary | 1.03 | 0.98 | 1.09 | 0.19 | 322461 | 1588 | 320873 | FALSE |
| 386.9 | Dizziness and giddiness (Light-headedness and vertigo) | Sense Organs | 0.98 | 0.95 | 1.01 | 0.19 | 326388 | 4739 | 321649 | FALSE |
| 369.5 | Conjunctivitis, infectious | Sense Organs | 0.92 | 0.80 | 1.04 | 0.19 | 318612 | 224 | 318388 | FALSE |
| 426.21 | First degree AV block | Circulatory System | 0.96 | 0.90 | 1.02 | 0.19 | 300526 | 1005 | 299521 | FALSE |
| 204.4 | Multiple myeloma | Neoplasms | 0.95 | 0.87 | 1.03 | 0.19 | 324097 | 574 | 323523 | FALSE |
| 117 | Mycoses | Infectious Diseases | 0.98 | 0.94 | 1.01 | 0.19 | 328235 | 2735 | 325500 | FALSE |
| 191 | Malignant and unknown neoplasms of brain and nervous system | Neoplasms | 0.92 | 0.80 | 1.05 | 0.19 | 326662 | 215 | 326447 | FALSE |
| 747.1 | Cardiac congenital anomalies | Congenital Anomalies | 1.09 | 0.95 | 1.25 | 0.20 | 325565 | 211 | 325354 | FALSE |
| 674 | Other complications of the puerperium NEC | Pregnancy Complications | 1.08 | 0.96 | 1.22 | 0.20 | 328015 | 280 | 327735 | FALSE |
| 960 | Poisoning by antibiotics | Injuries & Poisonings | 0.98 | 0.95 | 1.01 | 0.20 | 303739 | 3528 | 300211 | FALSE |
| 529 | Diseases and other conditions of the tongue | Digestive | 1.04 | 0.98 | 1.11 | 0.20 | 323485 | 951 | 322534 | FALSE |
| 939 | Atopic/contact dermatitis due to other or unspecified | Dermatologic | 1.03 | 0.99 | 1.07 | 0.20 | 324060 | 2154 | 321906 | FALSE |
| 429.2 | Abnormal function study of cardiovascular system | Circulatory System | 0.95 | 0.87 | 1.03 | 0.20 | 322577 | 544 | 322033 | FALSE |
| 433.8 | Late effects of cerebrovascular disease | Circulatory System | 0.96 | 0.91 | 1.02 | 0.20 | 319384 | 1277 | 318107 | FALSE |
| 695.7 | Prurigo and Lichen | Dermatologic | 0.96 | 0.89 | 1.02 | 0.20 | 322541 | 792 | 321749 | FALSE |
| 275.5 | Disorders of calcium/phosphorus metabolism | Endocrine/Metabolic | 0.96 | 0.91 | 1.02 | 0.20 | 327144 | 1109 | 326035 | FALSE |
| 198 | Secondary malignant neoplasm | Neoplasms | 0.96 | 0.91 | 1.02 | 0.20 | 230431 | 1119 | 229312 | FALSE |
| 530.14 | Reflux esophagitis | Digestive | 1.01 | 0.99 | 1.03 | 0.21 | 298696 | 10893 | 287803 | FALSE |
| 430.3 | Subdural hemorrhage | Circulatory System | 0.93 | 0.82 | 1.04 | 0.21 | 318378 | 271 | 318107 | FALSE |
| 204.21 | Myeloid leukemia, acute | Neoplasms | 1.07 | 0.96 | 1.20 | 0.21 | 323842 | 319 | 323523 | FALSE |
| 495 | Asthma | Respiratory | 0.99 | 0.98 | 1.00 | 0.21 | 320971 | 26779 | 294192 | FALSE |
| 430.2 | Intracerebral hemorrhage | Circulatory System | 0.95 | 0.89 | 1.03 | 0.21 | 318830 | 723 | 318107 | FALSE |
| 394.3 | Aortic valve disease | Circulatory System | 0.97 | 0.91 | 1.02 | 0.21 | 322836 | 1308 | 321528 | FALSE |
| 226 | Benign neoplasm of thyroid glands | Neoplasms | 1.08 | 0.96 | 1.22 | 0.21 | 326908 | 266 | 326642 | FALSE |
| 800 | Fracture of lower limb | Injuries & Poisonings | 1.05 | 0.97 | 1.13 | 0.21 | 324256 | 699 | 323557 | FALSE |
| 800.3 | Fracture of tibia and fibula | Injuries & Poisonings | 1.06 | 0.97 | 1.15 | 0.21 | 324092 | 535 | 323557 | FALSE |
| 966 | Poisoning by anticonvulsants and anti-Parkinsonism drugs | Injuries & Poisonings | 1.06 | 0.97 | 1.15 | 0.22 | 300747 | 536 | 300211 | FALSE |
| 596.5 | Functional disorders of bladder | Genitourinary | 1.03 | 0.98 | 1.08 | 0.22 | 315318 | 1682 | 313636 | FALSE |
| 360.2 | Progressive myopia | Sense Organs | 0.93 | 0.82 | 1.05 | 0.22 | 317032 | 250 | 316782 | FALSE |
| 602 | Other disorders of prostate | Genitourinary | 0.97 | 0.92 | 1.02 | 0.22 | 308765 | 1372 | 307393 | FALSE |
| 564.9 | Personal history of diseases of digestive system | Digestive | 0.99 | 0.97 | 1.01 | 0.22 | 274320 | 15924 | 258396 | FALSE |
| 530.2 | Esophageal bleeding (varices/hemorrhage) | Digestive | 1.03 | 0.98 | 1.08 | 0.22 | 289525 | 1722 | 287803 | FALSE |

| | | | | | | | | | | |
|---|---|---|---|---|---|---|---|---|---|---|
| 749 | Congenital anomalies of face and neck | Congenital Anomalies | 0.93 | 0.82 | 1.05 | 0.22 | 328137 | 271 | 327866 | FALSE |
| 242 | Thyrotoxicosis with or without goiter | Endocrine/Metabolic | 0.97 | 0.92 | 1.02 | 0.22 | 311794 | 1465 | 310329 | FALSE |
| 751.12 | Congenital anomalies of male genital organs | Congenital Anomalies | 0.92 | 0.81 | 1.05 | 0.22 | 326231 | 228 | 326003 | FALSE |
| 745 | Pain in joint | Musculoskeletal | 0.97 | 0.93 | 1.02 | 0.22 | 328240 | 1940 | 326300 | FALSE |
| 501 | Pneumonitis due to inhalation of food or vomitus | Respiratory | 0.95 | 0.88 | 1.03 | 0.22 | 317076 | 592 | 316484 | FALSE |
| 286.7 | Other and unspecified coagulation defects | Hematopoietic | 1.06 | 0.96 | 1.17 | 0.23 | 325902 | 405 | 325497 | FALSE |
| 276.5 | Hypovolemia | Endocrine/Metabolic | 0.98 | 0.94 | 1.01 | 0.23 | 323539 | 2895 | 320644 | FALSE |
| 962.3 | Hormones and synthetic substitutes causing adverse effects in therapeutic use | Injuries & Poisonings | 0.94 | 0.85 | 1.04 | 0.23 | 300575 | 364 | 300211 | FALSE |
| 627.1 | Postmenopausal bleeding | Genitourinary | 1.01 | 0.99 | 1.03 | 0.23 | 305697 | 9466 | 296231 | FALSE |
| 296.22 | Major depressive disorder | Mental Disorders | 1.06 | 0.96 | 1.18 | 0.23 | 283282 | 384 | 282898 | FALSE |
| 764 | Sciatica | Symptoms | 0.97 | 0.91 | 1.02 | 0.23 | 327944 | 1261 | 326683 | FALSE |
| 803 | Fracture of upper limb | Injuries & Poisonings | 0.94 | 0.86 | 1.04 | 0.23 | 323990 | 433 | 323557 | FALSE |
| 389.4 | Tinnitus | Sense Organs | 0.95 | 0.87 | 1.03 | 0.23 | 318735 | 541 | 318194 | FALSE |
| 250.7 | Diabetic retinopathy | Endocrine/Metabolic | 1.03 | 0.98 | 1.09 | 0.23 | 317232 | 1380 | 315852 | FALSE |
| 345 | Epilepsy, recurrent seizures, convulsions | Neurological | 0.98 | 0.95 | 1.01 | 0.23 | 289613 | 3461 | 286152 | FALSE |
| 722.9 | Other and unspecified disc disorder | Musculoskeletal | 1.02 | 0.99 | 1.05 | 0.24 | 314894 | 3950 | 310944 | FALSE |
| 565 | Anal and rectal conditions | Digestive | 1.01 | 0.99 | 1.04 | 0.24 | 255321 | 8661 | 246660 | FALSE |
| 275.3 | Disorders of magnesium metabolism | Endocrine/Metabolic | 0.94 | 0.85 | 1.04 | 0.24 | 326410 | 375 | 326035 | FALSE |
| 136 | Other infectious and parasitic diseases | Infectious Diseases | 0.94 | 0.86 | 1.04 | 0.24 | 327982 | 428 | 327554 | FALSE |
| 696.42 | Psoriatic arthropathy | Dermatologic | 0.96 | 0.89 | 1.03 | 0.24 | 314672 | 740 | 313932 | FALSE |
| 599.5 | Frequency of urination and polyuria | Genitourinary | 0.98 | 0.95 | 1.01 | 0.24 | 224574 | 4147 | 220427 | FALSE |
| 425.1 | Primary/intrinsic cardiomyopathies | Circulatory System | 0.96 | 0.91 | 1.02 | 0.24 | 326036 | 1053 | 324983 | FALSE |
| 386.3 | Labyrinthitis | Sense Organs | 1.04 | 0.97 | 1.12 | 0.24 | 322440 | 791 | 321649 | FALSE |
| 300.13 | Phobia | Mental Disorders | 0.95 | 0.87 | 1.04 | 0.25 | 283412 | 514 | 282898 | FALSE |
| 967 | Adverse effects of sedatives or other central nervous system depressants and anesthetics | Injuries & Poisonings | 0.95 | 0.88 | 1.03 | 0.25 | 300790 | 579 | 300211 | FALSE |
| 276.11 | Hyperosmolality and/or hypernatremia | Endocrine/Metabolic | 1.08 | 0.95 | 1.22 | 0.25 | 320891 | 247 | 320644 | FALSE |
| 965.2 | Antirheumatics causing adverse effects in therapeutic use | Injuries & Poisonings | 1.05 | 0.96 | 1.15 | 0.25 | 300710 | 499 | 300211 | FALSE |
| 594 | Urinary calculus | Genitourinary | 0.99 | 0.96 | 1.01 | 0.25 | 326897 | 6966 | 319931 | FALSE |
| 250.41 | Impaired fasting glucose | Endocrine/Metabolic | 1.07 | 0.95 | 1.21 | 0.25 | 307758 | 268 | 307490 | FALSE |
| 380.1 | Otitis externa | Sense Organs | 0.95 | 0.87 | 1.04 | 0.25 | 327284 | 532 | 326752 | FALSE |
| 306 | Other mental disorder | Mental Disorders | 0.99 | 0.98 | 1.01 | 0.25 | 312303 | 29405 | 282898 | FALSE |
| 41 | Bacterial infection NOS | Infectious Diseases | 1.01 | 0.99 | 1.03 | 0.25 | 323384 | 10752 | 312632 | FALSE |
| 276.13 | Hyperpotassemia | Endocrine/Metabolic | 1.04 | 0.97 | 1.10 | 0.25 | 321623 | 979 | 320644 | FALSE |
| 333.1 | Essential tremor | Neurological | 1.08 | 0.95 | 1.24 | 0.26 | 286370 | 218 | 286152 | FALSE |
| 571 | Chronic liver disease and cirrhosis | Digestive | 1.05 | 0.96 | 1.14 | 0.26 | 318871 | 540 | 318331 | FALSE |
| 599.4 | Urinary incontinence | Genitourinary | 0.99 | 0.97 | 1.01 | 0.26 | 229423 | 8996 | 220427 | FALSE |
| 420.3 | Endocarditis | Circulatory System | 0.96 | 0.89 | 1.03 | 0.26 | 325668 | 685 | 324983 | FALSE |
| 686.3 | Pilonidal cyst | Dermatologic | 1.05 | 0.97 | 1.13 | 0.26 | 317024 | 617 | 316407 | FALSE |

| | | | | | | | | | | |
|---|---|---|---|---|---|---|---|---|---|---|
| 451 | Phlebitis and thrombophlebitis | Circulatory System | 0.94 | 0.86 | 1.04 | 0.26 | 288110 | 397 | 287713 | FALSE |
| 521.1 | Dental caries | Digestive | 1.02 | 0.99 | 1.06 | 0.26 | 314912 | 3254 | 311658 | FALSE |
| 695.9 | Unspecified erythematous condition | Dermatologic | 1.05 | 0.96 | 1.15 | 0.26 | 322226 | 477 | 321749 | FALSE |
| 198.2 | Secondary malignancy of respiratory organs | Neoplasms | 1.02 | 0.98 | 1.07 | 0.26 | 231589 | 2277 | 229312 | FALSE |
| 530.7 | Gastroesophageal laceration-hemorrhage syndrome | Digestive | 1.06 | 0.95 | 1.19 | 0.26 | 288136 | 333 | 287803 | FALSE |
| 785 | Abdominal pain | Symptoms | 1.01 | 1.00 | 1.02 | 0.26 | 328240 | 42311 | 285929 | FALSE |
| 604.1 | Redundant prepuce and phimosis/BXO | Genitourinary | 0.98 | 0.94 | 1.02 | 0.26 | 310716 | 2810 | 307906 | FALSE |
| 597.1 | Urethral stricture (not specified as infectious) | Genitourinary | 0.98 | 0.95 | 1.01 | 0.26 | 317079 | 3443 | 313636 | FALSE |
| 743.9 | Osteopenia or other disorder of bone and cartilage | Musculoskeletal | 1.02 | 0.99 | 1.05 | 0.26 | 328188 | 3989 | 324199 | FALSE |
| 618.6 | Vaginal enterocele, congenital or acquired | Genitourinary | 1.04 | 0.97 | 1.13 | 0.26 | 316909 | 667 | 316242 | FALSE |
| 174.1 | Breast cancer [female] | Neoplasms | 0.98 | 0.94 | 1.02 | 0.27 | 306711 | 2287 | 304424 | FALSE |
| 531 | Peptic ulcer (excl. esophageal) | Digestive | 0.94 | 0.83 | 1.05 | 0.27 | 320833 | 284 | 320549 | FALSE |
| 495.2 | Asthma with exacerbation | Respiratory | 1.07 | 0.95 | 1.21 | 0.27 | 294453 | 261 | 294192 | FALSE |
| 561 | Symptoms involving digestive system | Digestive | 1.01 | 0.99 | 1.03 | 0.27 | 272941 | 14545 | 258396 | FALSE |
| 717 | Polymyalgia Rheumatica | Musculoskeletal | 0.97 | 0.91 | 1.03 | 0.27 | 328240 | 1147 | 327093 | FALSE |
| 969 | Poisoning by psychotropic agents | Injuries & Poisonings | 1.03 | 0.98 | 1.07 | 0.27 | 302207 | 1996 | 300211 | FALSE |
| 740.1 | Osteoarthritis; localized | Musculoskeletal | 0.99 | 0.97 | 1.01 | 0.27 | 315797 | 9472 | 306325 | FALSE |
| 362 | Other retinal disorders | Sense Organs | 1.04 | 0.97 | 1.10 | 0.27 | 316825 | 973 | 315852 | FALSE |
| 689 | Disorder of skin and subcutaneous tissue NOS | Dermatologic | 1.01 | 1.00 | 1.02 | 0.27 | 328240 | 42194 | 286046 | FALSE |
| 715 | Other inflammatory spondylopathies | Musculoskeletal | 0.94 | 0.83 | 1.05 | 0.28 | 317524 | 271 | 317253 | FALSE |
| 447.1 | Stricture of artery | Circulatory System | 0.96 | 0.90 | 1.03 | 0.28 | 320246 | 886 | 319360 | FALSE |
| 870.1 | Open wound or laceration of eye or eyelid | Injuries & Poisonings | 0.94 | 0.83 | 1.05 | 0.28 | 320012 | 281 | 319731 | FALSE |
| 788 | Syncope and collapse | Symptoms | 0.99 | 0.97 | 1.01 | 0.28 | 328240 | 9372 | 318868 | FALSE |
| 348.9 | Other conditions of brain, NOS | Neurological | 0.95 | 0.86 | 1.04 | 0.28 | 286559 | 407 | 286152 | FALSE |
| 622.2 | Mucous polyp of cervix | Genitourinary | 1.02 | 0.98 | 1.05 | 0.28 | 318829 | 3522 | 315307 | FALSE |
| 740.11 | Osteoarthrosis, localized, primary | Musculoskeletal | 1.01 | 0.99 | 1.03 | 0.29 | 315552 | 9227 | 306325 | FALSE |
| 720 | Spinal stenosis | Musculoskeletal | 1.04 | 0.97 | 1.12 | 0.29 | 311636 | 692 | 310944 | FALSE |
| 145.2 | Cancer of tongue | Neoplasms | 1.06 | 0.95 | 1.18 | 0.29 | 326366 | 325 | 326041 | FALSE |
| 610.8 | Other specified benign mammary dysplasias | Genitourinary | 0.96 | 0.89 | 1.03 | 0.29 | 321576 | 703 | 320873 | FALSE |
| 381.11 | Suppurative and unspecified otitis media | Sense Organs | 0.96 | 0.90 | 1.03 | 0.29 | 324993 | 850 | 324143 | FALSE |
| 508 | Pulmonary collapse; interstitial and compensatory emphysema | Respiratory | 0.98 | 0.94 | 1.02 | 0.29 | 318570 | 2086 | 316484 | FALSE |
| 41.2 | Streptococcus infection | Infectious Diseases | 0.97 | 0.93 | 1.02 | 0.29 | 314280 | 1648 | 312632 | FALSE |
| 701.5 | Abnormal granulation tissue | Dermatologic | 0.95 | 0.87 | 1.04 | 0.29 | 323543 | 479 | 323064 | FALSE |
| 333 | Extrapyramidal disease and abnormal movement disorders | Neurological | 1.02 | 0.98 | 1.07 | 0.29 | 288187 | 2035 | 286152 | FALSE |
| 383 | Otosclerosis | Sense Organs | 0.94 | 0.85 | 1.05 | 0.29 | 324473 | 330 | 324143 | FALSE |
| 573 | Other disorders of liver | Digestive | 1.01 | 0.99 | 1.04 | 0.29 | 323533 | 5202 | 318331 | FALSE |
| 70 | Viral hepatitis | Infectious Diseases | 0.96 | 0.89 | 1.03 | 0.30 | 322928 | 722 | 322206 | FALSE |
| 523.32 | Chronic periodontitis | Digestive | 0.96 | 0.88 | 1.04 | 0.30 | 312248 | 590 | 311658 | FALSE |

| | | | | | | | | | | | |
|---|---|---|---|---|---|---|---|---|---|---|---|
| 185 | Cancer of prostate | Neoplasms | 0.99 | 0.97 | 1.01 | 0.30 | 315856 | 8463 | 307393 | FALSE |
| 627.3 | Postmenopausal atrophic vaginitis | Genitourinary | 1.03 | 0.97 | 1.09 | 0.30 | 297359 | 1128 | 296231 | FALSE |
| 345.1 | Epilepsy | Neurological | 0.94 | 0.85 | 1.05 | 0.30 | 286483 | 331 | 286152 | FALSE |
| 619.5 | Noninflammatory disorders of vulva and perineum | Genitourinary | 0.97 | 0.92 | 1.03 | 0.30 | 279927 | 1379 | 278548 | FALSE |
| 337 | Disorders of the autonomic nervous system | Neurological | 0.93 | 0.81 | 1.07 | 0.30 | 286359 | 207 | 286152 | FALSE |
| 389.2 | Conductive hearing loss | Sense Organs | 1.05 | 0.96 | 1.16 | 0.31 | 318629 | 435 | 318194 | FALSE |
| 197 | Chemotherapy | Neoplasms | 0.99 | 0.98 | 1.01 | 0.31 | 251774 | 22462 | 229312 | FALSE |
| 726.3 | Bursitis | Musculoskeletal | 0.96 | 0.88 | 1.04 | 0.31 | 305497 | 543 | 304954 | FALSE |
| 789 | Nausea and vomiting | Symptoms | 0.99 | 0.97 | 1.01 | 0.31 | 328240 | 11983 | 316257 | FALSE |
| 530.3 | Stricture and stenosis of esophagus | Digestive | 1.02 | 0.98 | 1.07 | 0.31 | 289757 | 1954 | 287803 | FALSE |
| 285.22 | Anemia in neoplastic disease | Hematopoietic | 0.95 | 0.87 | 1.05 | 0.31 | 309337 | 437 | 308900 | FALSE |
| 522.5 | Periapical abscess | Digestive | 1.03 | 0.97 | 1.09 | 0.31 | 312904 | 1246 | 311658 | FALSE |
| 577.2 | Chronic pancreatitis | Digestive | 1.04 | 0.96 | 1.14 | 0.32 | 326456 | 536 | 325920 | FALSE |
| 523 | Gingival and periodontal diseases | Digestive | 0.96 | 0.88 | 1.04 | 0.32 | 312187 | 529 | 311658 | FALSE |
| 427.11 | Paroxysmal supraventricular tachycardia | Circulatory System | 0.98 | 0.94 | 1.02 | 0.32 | 301952 | 2431 | 299521 | FALSE |
| 496 | Chronic airway obstruction | Respiratory | 0.99 | 0.97 | 1.01 | 0.32 | 301827 | 7635 | 294192 | FALSE |
| 585.3 | Chronic renal failure [CKD] | Genitourinary | 0.98 | 0.94 | 1.02 | 0.32 | 316664 | 2585 | 314079 | FALSE |
| 428.2 | Heart failure NOS | Circulatory System | 0.99 | 0.96 | 1.02 | 0.33 | 326367 | 4334 | 322033 | FALSE |
| 8.6 | Viral Enteritis | Infectious Diseases | 0.97 | 0.90 | 1.03 | 0.33 | 319924 | 845 | 319079 | FALSE |
| 751.22 | Other specified congenital anomalies of kidney | Congenital Anomalies | 1.05 | 0.95 | 1.17 | 0.33 | 326353 | 350 | 326003 | FALSE |
| 614.3 | Pelvic inflammatory disease (PID) | Genitourinary | 1.06 | 0.94 | 1.21 | 0.33 | 319048 | 249 | 318799 | FALSE |
| 614.1 | Pelvic peritoneal adhesions, female (postoperative) (postinfection) | Genitourinary | 1.02 | 0.98 | 1.06 | 0.33 | 321370 | 2571 | 318799 | FALSE |
| 569 | Other disorders of intestine | Digestive | 1.00 | 0.99 | 1.00 | 0.34 | 326148 | 79488 | 246660 | FALSE |
| 572 | Ascites (non malignant) | Digestive | 1.02 | 0.98 | 1.08 | 0.34 | 319925 | 1594 | 318331 | FALSE |
| 714.1 | Rheumatoid arthritis | Musculoskeletal | 1.02 | 0.98 | 1.06 | 0.34 | 319560 | 2307 | 317253 | FALSE |
| 613 | Other nonmalignant breast conditions | Genitourinary | 1.05 | 0.95 | 1.17 | 0.34 | 322786 | 368 | 322418 | FALSE |
| 603.1 | Hydrocele | Genitourinary | 0.97 | 0.92 | 1.03 | 0.34 | 309308 | 1402 | 307906 | FALSE |
| 433.2 | Occlusion of cerebral arteries | Circulatory System | 1.02 | 0.98 | 1.06 | 0.34 | 321087 | 2980 | 318107 | FALSE |
| 519.9 | Symptoms involving respiratory system and other chest symptoms | Respiratory | 0.95 | 0.85 | 1.06 | 0.34 | 265755 | 308 | 265447 | FALSE |
| 345.3 | Convulsions | Neurological | 1.02 | 0.98 | 1.06 | 0.35 | 288463 | 2311 | 286152 | FALSE |
| 214 | Lipoma | Neoplasms | 1.02 | 0.98 | 1.07 | 0.35 | 322495 | 1819 | 320676 | FALSE |
| 348.7 | Coma | Neurological | 0.95 | 0.86 | 1.05 | 0.35 | 286531 | 379 | 286152 | FALSE |
| 696.4 | Psoriasis | Dermatologic | 0.98 | 0.93 | 1.03 | 0.35 | 315554 | 1622 | 313932 | FALSE |
| 372 | Disorders of conjunctiva | Sense Organs | 0.97 | 0.91 | 1.03 | 0.35 | 319431 | 1043 | 318388 | FALSE |
| 289.5 | Diseases of spleen | Hematopoietic | 1.04 | 0.96 | 1.13 | 0.35 | 320846 | 528 | 320318 | FALSE |
| 707 | Chronic ulcer of skin | Dermatologic | 0.97 | 0.92 | 1.03 | 0.35 | 327959 | 1260 | 326699 | FALSE |
| 550.1 | Inguinal hernia | Digestive | 1.01 | 0.99 | 1.02 | 0.35 | 296174 | 16385 | 279789 | FALSE |
| 574 | Cholelithiasis and cholecystitis | Digestive | 0.95 | 0.86 | 1.05 | 0.35 | 310726 | 403 | 310323 | FALSE |

| | | | | | | | | | | | |
|---|---|---|---|---|---|---|---|---|---|---|---|
| 480 | Pneumonia | Respiratory | 0.99 | 0.95 | 1.02 | 0.35 | 321603 | 3914 | 317689 | FALSE |
| 614.33 | Pelvic inflammatory disease, NOS | Genitourinary | 1.01 | 0.99 | 1.04 | 0.35 | 324944 | 6145 | 318799 | FALSE |
| 204.12 | Lymphoid leukemia, chronic | Neoplasms | 1.04 | 0.95 | 1.14 | 0.35 | 324030 | 507 | 323523 | FALSE |
| 759 | Other and unspecified congenital anomalies | Congenital Anomalies | 0.97 | 0.90 | 1.04 | 0.36 | 327249 | 679 | 326570 | FALSE |
| 626.12 | Excessive or frequent menstruation | Genitourinary | 1.01 | 0.99 | 1.03 | 0.36 | 306272 | 10041 | 296231 | FALSE |
| 367.1 | Myopia | Sense Organs | 0.97 | 0.92 | 1.03 | 0.36 | 327025 | 1271 | 325754 | FALSE |
| 216 | Benign neoplasm of skin | Neoplasms | 1.01 | 0.99 | 1.03 | 0.36 | 327614 | 7865 | 319749 | FALSE |
| 741 | Symptoms and disorders of the joints | Musculoskeletal | 0.99 | 0.98 | 1.01 | 0.36 | 327531 | 16889 | 310642 | FALSE |
| 612.2 | Hypertrophy of breast (Gynecomastia) | Genitourinary | 1.03 | 0.96 | 1.10 | 0.36 | 321732 | 859 | 320873 | FALSE |
| 695.42 | Systemic lupus erythematosus | Dermatologic | 0.95 | 0.86 | 1.06 | 0.36 | 320784 | 363 | 320421 | FALSE |
| 627.4 | Premenopausal menorrhagia | Genitourinary | 0.95 | 0.85 | 1.06 | 0.36 | 296563 | 332 | 296231 | FALSE |
| 38 | Septicemia | Infectious Diseases | 1.02 | 0.98 | 1.05 | 0.36 | 315677 | 3045 | 312632 | FALSE |
| 626.14 | Irregular menstrual bleeding | Genitourinary | 1.01 | 0.98 | 1.05 | 0.36 | 300177 | 3946 | 296231 | FALSE |
| 624.9 | stress incontinence, female | Genitourinary | 0.99 | 0.96 | 1.01 | 0.37 | 324769 | 6100 | 318669 | FALSE |
| 530.11 | GERD | Digestive | 0.99 | 0.98 | 1.01 | 0.37 | 302292 | 14489 | 287803 | FALSE |
| 535.2 | Atrophic gastritis | Digestive | 1.05 | 0.95 | 1.16 | 0.37 | 296869 | 358 | 296511 | FALSE |
| 809 | Fracture of unspecified bones | Injuries & Poisonings | 0.96 | 0.87 | 1.05 | 0.37 | 323967 | 410 | 323557 | FALSE |
| 394 | Rheumatic disease of the heart valves | Circulatory System | 0.97 | 0.91 | 1.04 | 0.37 | 322471 | 943 | 321528 | FALSE |
| 525 | Other diseases of the teeth and supporting structures | Digestive | 1.02 | 0.98 | 1.06 | 0.37 | 314357 | 2699 | 311658 | FALSE |
| 599.2 | Retention of urine | Genitourinary | 0.99 | 0.97 | 1.01 | 0.37 | 227284 | 6857 | 220427 | FALSE |
| 250.11 | Type 1 diabetes with ketoacidosis | Endocrine/Metabolic | 0.95 | 0.84 | 1.07 | 0.37 | 307745 | 255 | 307490 | FALSE |
| 696.41 | Psoriasis vulgaris | Dermatologic | 0.98 | 0.93 | 1.03 | 0.37 | 315655 | 1723 | 313932 | FALSE |
| 378.1 | Strabismus (not specified as paralytic) | Sense Organs | 0.97 | 0.91 | 1.04 | 0.37 | 289139 | 947 | 288192 | FALSE |
| 530 | Diseases of esophagus | Digestive | 0.97 | 0.90 | 1.04 | 0.37 | 288604 | 801 | 287803 | FALSE |
| 681.1 | Cellulitis and abscess of fingers/toes | Dermatologic | 0.96 | 0.89 | 1.05 | 0.38 | 316989 | 582 | 316407 | FALSE |
| 614.53 | Cyst or abscess of Bartholin's gland | Genitourinary | 1.03 | 0.96 | 1.11 | 0.38 | 319602 | 803 | 318799 | FALSE |
| 285.1 | Acute posthemorrhagic anemia | Hematopoietic | 0.95 | 0.84 | 1.07 | 0.38 | 309167 | 267 | 308900 | FALSE |
| 440 | Atherosclerosis | Circulatory System | 1.06 | 0.93 | 1.22 | 0.38 | 319564 | 204 | 319360 | FALSE |
| 870.3 | Other open wound of head and face | Injuries & Poisonings | 1.02 | 0.98 | 1.05 | 0.38 | 322782 | 3051 | 319731 | FALSE |
| 496.21 | Obstructive chronic bronchitis | Respiratory | 0.98 | 0.95 | 1.02 | 0.38 | 296921 | 2729 | 294192 | FALSE |
| 726.2 | Synoviopathy | Musculoskeletal | 0.95 | 0.86 | 1.06 | 0.38 | 305285 | 331 | 304954 | FALSE |
| 598 | Abnormal findings on examination of urine | Genitourinary | 0.99 | 0.95 | 1.02 | 0.38 | 328240 | 3386 | 324854 | FALSE |
| 626.2 | Dysmenorrhea | Genitourinary | 0.98 | 0.93 | 1.03 | 0.39 | 297910 | 1679 | 296231 | FALSE |
| 614.5 | Inflammatory disease of cervix, vagina, and vulva | Genitourinary | 0.95 | 0.86 | 1.06 | 0.39 | 319133 | 334 | 318799 | FALSE |
| 618.1 | Prolapse of vaginal walls | Genitourinary | 0.99 | 0.97 | 1.01 | 0.39 | 323726 | 7484 | 316242 | FALSE |
| 418 | Nonspecific chest pain | Circulatory System | 0.99 | 0.98 | 1.01 | 0.39 | 326032 | 30212 | 295820 | FALSE |
| 202.2 | Non-Hodgkins lymphoma | Neoplasms | 0.99 | 0.95 | 1.02 | 0.39 | 327055 | 3532 | 323523 | FALSE |
| 599.9 | Other abnormality of urination | Genitourinary | 0.98 | 0.94 | 1.03 | 0.39 | 222371 | 1944 | 220427 | FALSE |

| | | | | | | | | | | |
|---|---|---|---|---|---|---|---|---|---|---|
| 601.1 | Prostatitis | Genitourinary | 1.03 | 0.96 | 1.12 | 0.40 | 308058 | 665 | 307393 | FALSE |
| 681.7 | Cellulitis and abscess of trunk | Dermatologic | 0.97 | 0.89 | 1.05 | 0.40 | 317030 | 623 | 316407 | FALSE |
| 426.91 | Cardiac pacemaker in situ | Circulatory System | 0.98 | 0.94 | 1.02 | 0.40 | 301837 | 2316 | 299521 | FALSE |
| 575.2 | Obstruction of bile duct | Digestive | 0.97 | 0.91 | 1.04 | 0.40 | 311135 | 812 | 310323 | FALSE |
| 736.2 | Acquired deformities of finger | Musculoskeletal | 0.95 | 0.84 | 1.07 | 0.40 | 316940 | 253 | 316687 | FALSE |
| 451.2 | Phlebitis and thrombophlebitis of lower extremities | Circulatory System | 0.99 | 0.95 | 1.02 | 0.41 | 291357 | 3644 | 287713 | FALSE |
| 528.7 | Sialolithiasis | Digestive | 1.05 | 0.94 | 1.17 | 0.41 | 322847 | 313 | 322534 | FALSE |
| 567 | Peritonitis and retroperitoneal infections | Digestive | 0.97 | 0.91 | 1.04 | 0.41 | 247550 | 890 | 246660 | FALSE |
| 706 | Diseases of sebaceous glands | Dermatologic | 1.04 | 0.95 | 1.12 | 0.41 | 318889 | 595 | 318294 | FALSE |
| 187.1 | Malignant neoplasm of unspecified male genital organ | Neoplasms | 0.99 | 0.97 | 1.01 | 0.41 | 327699 | 8481 | 319218 | FALSE |
| 938.2 | Chronic dermatitis due to solar radiation | Injuries & Poisonings | 0.95 | 0.84 | 1.07 | 0.41 | 322170 | 264 | 321906 | FALSE |
| 300 | Anxiety, phobic and dissociative disorders | Mental Disorders | 0.96 | 0.86 | 1.06 | 0.41 | 283261 | 363 | 282898 | FALSE |
| 574.3 | Cholecystitis without cholelithiasis | Digestive | 0.98 | 0.95 | 1.02 | 0.42 | 313141 | 2818 | 310323 | FALSE |
| 411.1 | Unstable angina (intermediate coronary syndrome) | Circulatory System | 0.99 | 0.96 | 1.02 | 0.42 | 301011 | 5322 | 295689 | FALSE |
| 365 | Glaucoma | Sense Organs | 0.99 | 0.96 | 1.02 | 0.42 | 321340 | 4558 | 316782 | FALSE |
| 38.2 | Gram positive septicemia | Infectious Diseases | 1.04 | 0.95 | 1.13 | 0.42 | 313123 | 491 | 312632 | FALSE |
| 250.6 | Polyneuropathy in diabetes | Endocrine/Metabolic | 1.04 | 0.94 | 1.15 | 0.42 | 307871 | 381 | 307490 | FALSE |
| 215 | Other benign neoplasm of connective and other soft tissue | Neoplasms | 0.98 | 0.92 | 1.04 | 0.42 | 321804 | 1128 | 320676 | FALSE |
| 427.7 | Tachycardia NOS | Circulatory System | 0.98 | 0.94 | 1.02 | 0.42 | 301787 | 2266 | 299521 | FALSE |
| 379.2 | Disorders of vitreous body | Sense Organs | 0.98 | 0.93 | 1.03 | 0.42 | 289599 | 1407 | 288192 | FALSE |
| 300.1 | Anxiety disorder | Mental Disorders | 0.99 | 0.96 | 1.02 | 0.42 | 288368 | 5470 | 282898 | FALSE |
| 252.1 | Hyperparathyroidism | Endocrine/Metabolic | 1.03 | 0.96 | 1.10 | 0.43 | 325359 | 814 | 324545 | FALSE |
| 195.1 | Malignant neoplasm, other | Neoplasms | 1.00 | 0.99 | 1.00 | 0.43 | 320138 | 90826 | 229312 | FALSE |
| 801 | Fracture of ankle and foot | Injuries & Poisonings | 0.95 | 0.84 | 1.08 | 0.43 | 323808 | 251 | 323557 | FALSE |
| 735 | Acquired foot deformities | Musculoskeletal | 0.95 | 0.85 | 1.07 | 0.43 | 316986 | 299 | 316687 | FALSE |
| 591 | Urinary tract infection | Genitourinary | 1.01 | 0.99 | 1.03 | 0.43 | 309711 | 12676 | 297035 | FALSE |
| 946 | Anaphylactic shock NOS | Injuries & Poisonings | 0.97 | 0.89 | 1.05 | 0.43 | 322450 | 544 | 321906 | FALSE |
| 385.5 | Tympanosclerosis and middle ear disease related to otitis media | Sense Organs | 0.95 | 0.84 | 1.08 | 0.43 | 324379 | 236 | 324143 | FALSE |
| 516.1 | Hemoptysis | Respiratory | 1.02 | 0.97 | 1.06 | 0.43 | 328154 | 2103 | 326051 | FALSE |
| 550.2 | Diaphragmatic hernia | Digestive | 1.00 | 0.98 | 1.01 | 0.43 | 307453 | 27664 | 279789 | FALSE |
| 455 | Hemorrhoids | Circulatory System | 1.01 | 0.99 | 1.02 | 0.43 | 312410 | 24697 | 287713 | FALSE |
| 210 | Benign neoplasm of lip, oral cavity, and pharynx | Neoplasms | 1.03 | 0.96 | 1.09 | 0.43 | 327034 | 993 | 326041 | FALSE |
| 227.2 | Benign neoplasm of parathyroid gland | Neoplasms | 1.04 | 0.94 | 1.15 | 0.44 | 327032 | 390 | 326642 | FALSE |
| 965 | Poisoning by analgesics, antipyretics, and antirheumatics | Injuries & Poisonings | 0.99 | 0.97 | 1.02 | 0.44 | 306293 | 6082 | 300211 | FALSE |
| 389 | Hearing loss | Sense Organs | 0.99 | 0.95 | 1.02 | 0.44 | 321364 | 3170 | 318194 | FALSE |
| 415 | Pulmonary heart disease | Circulatory System | 1.01 | 0.98 | 1.05 | 0.44 | 325300 | 3753 | 321547 | FALSE |
| 281.11 | Pernicious anemia | Hematopoietic | 0.97 | 0.90 | 1.04 | 0.44 | 309650 | 750 | 308900 | FALSE |
| 212 | Benign neoplasm of respiratory and intrathoracic organs | Neoplasms | 0.96 | 0.87 | 1.07 | 0.44 | 328240 | 355 | 327885 | FALSE |

| | | | | | | | | | | |
|---|---|---|---|---|---|---|---|---|---|---|
| 619.4 | Noninflammatory disorders of vagina | Genitourinary | 1.02 | 0.97 | 1.07 | 0.44 | 280328 | 1780 | 278548 | FALSE |
| 618 | Genital prolapse | Genitourinary | 0.97 | 0.90 | 1.05 | 0.44 | 316862 | 620 | 316242 | FALSE |
| 458.2 | Iatrogenic hypotension | Circulatory System | 0.95 | 0.84 | 1.08 | 0.45 | 194417 | 238 | 194179 | FALSE |
| 578.2 | Blood in stool | Digestive | 1.01 | 0.98 | 1.05 | 0.45 | 306143 | 2749 | 303394 | FALSE |
| 228 | Hemangioma and lymphangioma, any site | Neoplasms | 1.02 | 0.97 | 1.07 | 0.45 | 328240 | 1635 | 326605 | FALSE |
| 636.2 | Early onset of delivery | Pregnancy Complications | 1.03 | 0.95 | 1.12 | 0.45 | 320594 | 629 | 319965 | FALSE |
| 859 | Complication due to other implant and internal device | Injuries & Poisonings | 1.01 | 0.98 | 1.05 | 0.45 | 317302 | 3338 | 313964 | FALSE |
| 531.3 | Duodenal ulcer | Digestive | 0.99 | 0.95 | 1.02 | 0.45 | 323644 | 3095 | 320549 | FALSE |
| 386 | Vertiginous syndromes and other disorders of vestibular system | Sense Organs | 1.02 | 0.97 | 1.06 | 0.45 | 323761 | 2112 | 321649 | FALSE |
| 994.2 | Sepsis | Injuries & Poisonings | 1.01 | 0.98 | 1.05 | 0.45 | 328240 | 2885 | 325355 | FALSE |
| 427.4 | Cardiac arrest and ventricular fibrillation | Circulatory System | 0.96 | 0.86 | 1.07 | 0.45 | 299869 | 348 | 299521 | FALSE |
| 351 | Other peripheral nerve disorders | Neurological | 0.99 | 0.98 | 1.01 | 0.45 | 325903 | 12892 | 313011 | FALSE |
| 614.51 | Cervicitis and endocervicitis | Genitourinary | 1.02 | 0.97 | 1.08 | 0.45 | 320048 | 1249 | 318799 | FALSE |
| 559 | Ileostomy status | Digestive | 0.98 | 0.94 | 1.03 | 0.45 | 260069 | 1673 | 258396 | FALSE |
| 290 | Delirium dementia and amnestic and other cognitive disorders | Mental Disorders | 1.04 | 0.95 | 1.13 | 0.45 | 321950 | 480 | 321470 | FALSE |
| 290.1 | Dementias | Mental Disorders | 1.03 | 0.95 | 1.11 | 0.45 | 322121 | 651 | 321470 | FALSE |
| 603 | Other disorders of testis | Genitourinary | 0.98 | 0.92 | 1.04 | 0.45 | 308895 | 989 | 307906 | FALSE |
| 560.4 | Other intestinal obstruction | Digestive | 0.99 | 0.95 | 1.02 | 0.46 | 261839 | 3443 | 258396 | FALSE |
| 323 | Encephalitis | Neurological | 1.03 | 0.96 | 1.10 | 0.46 | 327999 | 861 | 327138 | FALSE |
| 496.2 | Chronic bronchitis | Respiratory | 1.04 | 0.93 | 1.17 | 0.46 | 294485 | 293 | 294192 | FALSE |
| 781 | Symptoms involving nervous and musculoskeletal systems | Symptoms | 0.99 | 0.98 | 1.01 | 0.46 | 328238 | 22180 | 306058 | FALSE |
| 359.2 | Myopathy | Neurological | 1.03 | 0.95 | 1.13 | 0.46 | 326450 | 498 | 325952 | FALSE |
| 274.1 | Gout | Endocrine/Metabolic | 0.98 | 0.94 | 1.03 | 0.46 | 327930 | 1668 | 326262 | FALSE |
| 389.1 | Sensorineural hearing loss | Sense Organs | 1.04 | 0.94 | 1.13 | 0.46 | 318658 | 464 | 318194 | FALSE |
| 560.2 | Impaction of intestine | Digestive | 0.96 | 0.85 | 1.08 | 0.46 | 258667 | 271 | 258396 | FALSE |
| 189.2 | Cancer of bladder | Neoplasms | 0.97 | 0.91 | 1.04 | 0.46 | 324798 | 836 | 323962 | FALSE |
| 565.1 | Anal and rectal polyp | Digestive | 0.99 | 0.97 | 1.01 | 0.46 | 254329 | 7669 | 246660 | FALSE |
| 742.9 | Other derangement of joint | Musculoskeletal | 1.04 | 0.94 | 1.15 | 0.46 | 311042 | 400 | 310642 | FALSE |
| 353 | Nerve root and plexus disorders | Neurological | 0.98 | 0.92 | 1.04 | 0.46 | 314005 | 994 | 313011 | FALSE |
| 274.21 | Chondrocalcinosis | Endocrine/Metabolic | 0.96 | 0.85 | 1.07 | 0.47 | 326552 | 290 | 326262 | FALSE |
| 292.3 | Memory loss | Mental Disorders | 1.03 | 0.96 | 1.10 | 0.47 | 322208 | 738 | 321470 | FALSE |
| 165.1 | Cancer of bronchus; lung | Neoplasms | 1.02 | 0.97 | 1.06 | 0.47 | 327450 | 2181 | 325269 | FALSE |
| 379.3 | Aphakia and other disorders of lens | Sense Organs | 0.99 | 0.98 | 1.01 | 0.47 | 308920 | 20728 | 288192 | FALSE |
| 610 | Benign mammary dysplasias | Genitourinary | 1.05 | 0.92 | 1.21 | 0.47 | 321081 | 208 | 320873 | FALSE |
| 727.4 | Ganglion and cyst of synovium, tendon, and bursa | Musculoskeletal | 0.99 | 0.95 | 1.02 | 0.47 | 307936 | 2982 | 304954 | FALSE |
| 642 | Hypertension complicating pregnancy, childbirth, and the puerperium | Pregnancy Complications | 0.98 | 0.92 | 1.04 | 0.47 | 328039 | 989 | 327050 | FALSE |
| 54 | Herpes simplex | Infectious Diseases | 0.95 | 0.83 | 1.09 | 0.47 | 322425 | 219 | 322206 | FALSE |
| 519 | Other diseases of respiratory system, not elsewhere classified | Respiratory | 1.00 | 0.99 | 1.01 | 0.47 | 327615 | 62168 | 265447 | FALSE |

| | | | | | | | | | | |
|---|---|---|---|---|---|---|---|---|---|---|
| 550.5 | Ventral hernia | Digestive | 0.99 | 0.96 | 1.02 | 0.47 | 283371 | 3582 | 279789 | FALSE |
| 276.14 | Hypopotassemia | Endocrine/Metabolic | 0.98 | 0.93 | 1.03 | 0.47 | 322115 | 1471 | 320644 | FALSE |
| 285 | Other anemias | Hematopoietic | 1.01 | 0.99 | 1.03 | 0.47 | 320744 | 11844 | 308900 | FALSE |
| 184.2 | Cancer of other female genital organs | Neoplasms | 1.04 | 0.93 | 1.16 | 0.48 | 305876 | 323 | 305553 | FALSE |
| 695.3 | Rosacea | Dermatologic | 0.96 | 0.86 | 1.07 | 0.48 | 322068 | 319 | 321749 | FALSE |
| 198.3 | Secondary malignant neoplasm of digestive systems | Neoplasms | 0.98 | 0.93 | 1.03 | 0.48 | 230877 | 1565 | 229312 | FALSE |
| 614 | Inflammatory diseases of female pelvic organs | Genitourinary | 1.04 | 0.94 | 1.15 | 0.48 | 319173 | 374 | 318799 | FALSE |
| 70.9 | Hepatitis NOS | Infectious Diseases | 1.03 | 0.94 | 1.13 | 0.48 | 322658 | 452 | 322206 | FALSE |
| 686.4 | Pyogenic granuloma | Dermatologic | 0.96 | 0.87 | 1.07 | 0.48 | 316787 | 380 | 316407 | FALSE |
| 170.2 | Cancer of connective tissue | Neoplasms | 1.03 | 0.95 | 1.10 | 0.48 | 328122 | 734 | 327388 | FALSE |
| 420.21 | Acute pericarditis | Circulatory System | 0.95 | 0.84 | 1.09 | 0.48 | 325211 | 228 | 324983 | FALSE |
| 771.1 | Swelling of limb | Symptoms | 0.98 | 0.91 | 1.04 | 0.49 | 327356 | 842 | 326514 | FALSE |
| 571.81 | Portal hypertension | Digestive | 1.03 | 0.95 | 1.12 | 0.49 | 318900 | 569 | 318331 | FALSE |
| 293.1 | Swelling, mass, or lump in head and neck [Space-occupying lesion, intracranial NOS] | Mental Disorders | 0.98 | 0.92 | 1.04 | 0.49 | 326044 | 925 | 325119 | FALSE |
| 512.7 | Shortness of breath | Respiratory | 0.99 | 0.97 | 1.02 | 0.49 | 320384 | 6116 | 314268 | FALSE |
| 500.2 | Pneumoconiosis | Respiratory | 0.98 | 0.92 | 1.04 | 0.49 | 317485 | 1001 | 316484 | FALSE |
| 626.13 | Irregular menstrual cycle | Genitourinary | 1.02 | 0.97 | 1.06 | 0.49 | 298189 | 1958 | 296231 | FALSE |
| 550 | Abdominal hernia | Digestive | 1.00 | 0.99 | 1.01 | 0.49 | 328240 | 48451 | 279789 | FALSE |
| 594.3 | Calculus of ureter | Genitourinary | 1.01 | 0.97 | 1.05 | 0.50 | 322471 | 2540 | 319931 | FALSE |
| 577.3 | Cyst and pseudocyst of pancreas | Digestive | 1.04 | 0.94 | 1.15 | 0.50 | 326295 | 375 | 325920 | FALSE |
| 339 | Other headache syndromes | Neurological | 1.01 | 0.99 | 1.03 | 0.50 | 325888 | 7938 | 317950 | FALSE |
| 805 | Fracture of vertebral column without mention of spinal cord injury | Injuries & Poisonings | 0.96 | 0.87 | 1.07 | 0.50 | 323890 | 333 | 323557 | FALSE |
| 433.21 | Cerebral artery occlusion, with cerebral infarction | Circulatory System | 1.02 | 0.97 | 1.07 | 0.50 | 319641 | 1534 | 318107 | FALSE |
| 8.52 | Intestinal infection due to C. difficile | Infectious Diseases | 0.97 | 0.90 | 1.05 | 0.50 | 319727 | 648 | 319079 | FALSE |
| 429.3 | Symptoms involving cardiovascular system | Circulatory System | 0.95 | 0.83 | 1.09 | 0.50 | 322241 | 208 | 322033 | FALSE |
| 379 | Other disorders of eye | Sense Organs | 1.00 | 0.99 | 1.01 | 0.51 | 328190 | 39998 | 288192 | FALSE |
| 535.8 | Other specified gastritis | Digestive | 0.99 | 0.97 | 1.01 | 0.51 | 304926 | 8415 | 296511 | FALSE |
| 327 | Sleep disorders | Neurological | 0.98 | 0.92 | 1.04 | 0.51 | 323754 | 1034 | 322720 | FALSE |
| 519.2 | Respiratory complications | Respiratory | 1.04 | 0.93 | 1.17 | 0.51 | 265743 | 296 | 265447 | FALSE |
| 426.23 | Second degree AV block | Circulatory System | 0.97 | 0.88 | 1.06 | 0.51 | 299982 | 461 | 299521 | FALSE |
| 79 | Viral infection | Infectious Diseases | 0.99 | 0.96 | 1.02 | 0.51 | 326439 | 4233 | 322206 | FALSE |
| 338.1 | Acute pain | Neurological | 0.97 | 0.88 | 1.07 | 0.51 | 327846 | 420 | 327426 | FALSE |
| 872 | Traumatic amputation | Injuries & Poisonings | 1.03 | 0.94 | 1.13 | 0.51 | 320213 | 482 | 319731 | FALSE |
| 590 | Pyelonephritis | Genitourinary | 0.99 | 0.96 | 1.02 | 0.51 | 300960 | 3925 | 297035 | FALSE |
| 375.2 | Epiphora | Sense Organs | 0.98 | 0.92 | 1.04 | 0.52 | 289094 | 902 | 288192 | FALSE |
| 165 | Cancer within the respiratory system | Neoplasms | 1.01 | 0.98 | 1.05 | 0.52 | 328223 | 2954 | 325269 | FALSE |
| 571.8 | Liver abscess and sequelae of chronic liver disease | Digestive | 0.97 | 0.90 | 1.06 | 0.52 | 318904 | 573 | 318331 | FALSE |
| 172.11 | Melanomas of skin | Neoplasms | 0.99 | 0.95 | 1.03 | 0.52 | 316943 | 2723 | 314220 | FALSE |

| | | | | | | | | | | |
|---|---|---|---|---|---|---|---|---|---|---|
| 470 | Septal Deviations/Turbinate Hypertrophy | Respiratory | 0.99 | 0.96 | 1.02 | 0.52 | 313771 | 4821 | 308950 | FALSE |
| 172.2 | Other non-epithelial cancer of skin | Neoplasms | 0.99 | 0.98 | 1.01 | 0.52 | 325462 | 11242 | 314220 | FALSE |
| 790.6 | Other abnormal blood chemistry | Symptoms | 0.99 | 0.97 | 1.02 | 0.52 | 328029 | 6529 | 321500 | FALSE |
| 958 | Certain early complications of trauma or procedure | Injuries & Poisonings | 0.97 | 0.88 | 1.07 | 0.52 | 328118 | 381 | 327737 | FALSE |
| 798.1 | Chronic fatigue syndrome | Symptoms | 0.97 | 0.90 | 1.06 | 0.52 | 325335 | 606 | 324729 | FALSE |
| 433.1 | Occlusion and stenosis of precerebral arteries | Circulatory System | 1.02 | 0.96 | 1.08 | 0.52 | 319149 | 1042 | 318107 | FALSE |
| 245.21 | Chronic lymphocytic thyroiditis | Endocrine/Metabolic | 1.04 | 0.91 | 1.19 | 0.52 | 310548 | 219 | 310329 | FALSE |
| 286.12 | Congenital deficiency of other clotting factors (including factor VII) | Hematopoietic | 1.04 | 0.93 | 1.16 | 0.52 | 325800 | 303 | 325497 | FALSE |
| 241.2 | Nontoxic multinodular goiter | Endocrine/Metabolic | 0.98 | 0.91 | 1.05 | 0.53 | 311043 | 714 | 310329 | FALSE |
| 722.1 | Displacement of intervertebral disc | Musculoskeletal | 1.03 | 0.94 | 1.12 | 0.53 | 311460 | 516 | 310944 | FALSE |
| 496.1 | Emphysema | Respiratory | 0.98 | 0.94 | 1.03 | 0.53 | 295918 | 1726 | 294192 | FALSE |
| 433.31 | Transient cerebral ischemia | Circulatory System | 1.01 | 0.97 | 1.06 | 0.53 | 320281 | 2174 | 318107 | FALSE |
| 276.6 | Fluid overload | Endocrine/Metabolic | 0.97 | 0.89 | 1.06 | 0.53 | 321117 | 473 | 320644 | FALSE |
| 350.2 | Abnormality of gait | Neurological | 1.02 | 0.97 | 1.07 | 0.53 | 327077 | 1633 | 325444 | FALSE |
| 427.41 | Ventricular fibrillation and flutter | Circulatory System | 0.97 | 0.87 | 1.07 | 0.53 | 299866 | 345 | 299521 | FALSE |
| 281 | Other deficiency anemia | Hematopoietic | 0.99 | 0.97 | 1.01 | 0.53 | 317687 | 8787 | 308900 | FALSE |
| 281.13 | Folate-deficiency anemia | Hematopoietic | 0.96 | 0.83 | 1.10 | 0.54 | 309101 | 201 | 308900 | FALSE |
| 444 | Arterial embolism and thrombosis | Circulatory System | 0.97 | 0.88 | 1.07 | 0.54 | 319794 | 434 | 319360 | FALSE |
| 535.1 | Acute gastritis | Digestive | 0.98 | 0.93 | 1.04 | 0.54 | 297704 | 1193 | 296511 | FALSE |
| 557 | Intestinal malabsorption (non-celiac) | Digestive | 1.04 | 0.92 | 1.16 | 0.54 | 258690 | 294 | 258396 | FALSE |
| 480.11 | Pneumococcal pneumonia | Respiratory | 0.99 | 0.97 | 1.02 | 0.54 | 323680 | 5991 | 317689 | FALSE |
| 503 | Pulmonary congestion and hypostasis | Respiratory | 1.03 | 0.94 | 1.13 | 0.54 | 316959 | 475 | 316484 | FALSE |
| 716.2 | Unspecified monoarthritis | Musculoskeletal | 0.99 | 0.98 | 1.01 | 0.54 | 289797 | 15901 | 273896 | FALSE |
| 573.5 | Jaundice (not of newborn) | Digestive | 1.02 | 0.96 | 1.09 | 0.54 | 319269 | 938 | 318331 | FALSE |
| 427.8 | Sinoatrial node dysfunction (Bradycardia) | Circulatory System | 0.97 | 0.88 | 1.07 | 0.54 | 299940 | 419 | 299521 | FALSE |
| 735.3 | Hallux valgus (Bunion) | Musculoskeletal | 0.99 | 0.97 | 1.02 | 0.54 | 323592 | 6905 | 316687 | FALSE |
| 420.2 | Pericarditis | Circulatory System | 1.02 | 0.96 | 1.08 | 0.54 | 326070 | 1087 | 324983 | FALSE |
| 447 | Other disorders of arteries and arterioles | Circulatory System | 0.97 | 0.90 | 1.06 | 0.54 | 319916 | 556 | 319360 | FALSE |
| 751.11 | Congenital anomalies of female genital organs | Congenital Anomalies | 1.03 | 0.94 | 1.13 | 0.54 | 326445 | 442 | 326003 | FALSE |
| 703.1 | Ingrowing nail | Dermatologic | 1.02 | 0.96 | 1.08 | 0.54 | 322439 | 1010 | 321429 | FALSE |
| 440.9 | Atherosclerosis of aorta | Circulatory System | 1.04 | 0.91 | 1.20 | 0.55 | 319562 | 202 | 319360 | FALSE |
| 255.21 | Glucocorticoid deficiency | Endocrine/Metabolic | 1.03 | 0.93 | 1.14 | 0.55 | 324917 | 372 | 324545 | FALSE |
| 593 | Hematuria | Genitourinary | 1.00 | 0.99 | 1.02 | 0.55 | 313795 | 16760 | 297035 | FALSE |
| 610.3 | Fibrosclerosis of breast | Genitourinary | 0.97 | 0.86 | 1.08 | 0.55 | 321176 | 303 | 320873 | FALSE |
| 320 | Meningitis | Neurological | 1.03 | 0.94 | 1.13 | 0.55 | 327577 | 439 | 327138 | FALSE |
| 594.8 | Renal colic | Genitourinary | 0.99 | 0.95 | 1.03 | 0.55 | 322062 | 2131 | 319931 | FALSE |
| 514 | Abnormal findings examination of lungs | Respiratory | 0.99 | 0.95 | 1.03 | 0.56 | 328212 | 2577 | 325635 | FALSE |
| 600 | Hyperplasia of prostate | Genitourinary | 0.99 | 0.98 | 1.01 | 0.56 | 318965 | 11572 | 307393 | FALSE |

| | | | | | | | | | | |
|---|---|---|---|---|---|---|---|---|---|---|
| 479 | Other upper respiratory disease | Respiratory | 1.00 | 0.99 | 1.02 | 0.56 | 324241 | 15291 | 308950 | FALSE |
| 540 | Appendiceal conditions | Digestive | 0.99 | 0.96 | 1.02 | 0.56 | 328240 | 3861 | 324379 | FALSE |
| 636 | Early or threatened labor; hemorrhage in early pregnancy | Pregnancy Complications | 0.98 | 0.93 | 1.04 | 0.56 | 321227 | 1262 | 319965 | FALSE |
| 941 | Adverse reaction to serum or vaccine | Injuries & Poisonings | 1.04 | 0.91 | 1.19 | 0.56 | 322128 | 222 | 321906 | FALSE |
| 290.11 | Alzheimer's disease | Mental Disorders | 1.03 | 0.93 | 1.14 | 0.57 | 321872 | 402 | 321470 | FALSE |
| 430.1 | Subarachnoid hemorrhage | Circulatory System | 0.98 | 0.92 | 1.05 | 0.57 | 318931 | 824 | 318107 | FALSE |
| 726.1 | Enthesopathy | Musculoskeletal | 0.99 | 0.97 | 1.02 | 0.57 | 311657 | 6703 | 304954 | FALSE |
| 149.1 | Cancer of oropharynx | Neoplasms | 1.04 | 0.92 | 1.17 | 0.57 | 326297 | 256 | 326041 | FALSE |
| 556 | Ulceration of the lower GI tract | Digestive | 0.97 | 0.89 | 1.07 | 0.57 | 258836 | 440 | 258396 | FALSE |
| 213 | Benign neoplasm of bone and articular cartilage | Neoplasms | 0.97 | 0.87 | 1.08 | 0.57 | 277528 | 322 | 277206 | FALSE |
| 750.21 | Congenital anomalies of intestine | Congenital Anomalies | 0.96 | 0.85 | 1.10 | 0.58 | 326230 | 227 | 326003 | FALSE |
| 742.8 | Articular cartilage disorder | Musculoskeletal | 0.98 | 0.91 | 1.06 | 0.58 | 311297 | 655 | 310642 | FALSE |
| 78 | Viral warts & HPV | Infectious Diseases | 0.98 | 0.92 | 1.05 | 0.58 | 323218 | 1012 | 322206 | FALSE |
| 473.4 | Voice disturbance | Respiratory | 0.98 | 0.93 | 1.04 | 0.58 | 310051 | 1101 | 308950 | FALSE |
| 193 | Thyroid cancer | Neoplasms | 0.97 | 0.88 | 1.08 | 0.58 | 327009 | 367 | 326642 | FALSE |
| 174.11 | Malignant neoplasm of female breast | Neoplasms | 1.01 | 0.99 | 1.02 | 0.58 | 316838 | 12414 | 304424 | FALSE |
| 653 | Problems associated with amniotic cavity and membranes | Pregnancy Complications | 0.99 | 0.94 | 1.04 | 0.58 | 328119 | 1523 | 326596 | FALSE |
| 263 | Other nutritional deficiency | Endocrine/Metabolic | 0.99 | 0.94 | 1.04 | 0.58 | 327337 | 1551 | 325786 | FALSE |
| 585.2 | Renal failure NOS | Genitourinary | 0.99 | 0.97 | 1.02 | 0.58 | 322824 | 8745 | 314079 | FALSE |
| 604 | Disorders of penis | Genitourinary | 0.98 | 0.91 | 1.05 | 0.58 | 308641 | 735 | 307906 | FALSE |
| 700 | Corns and callosities | Dermatologic | 0.97 | 0.86 | 1.09 | 0.59 | 323352 | 288 | 323064 | FALSE |
| 278.1 | Obesity | Endocrine/Metabolic | 1.01 | 0.99 | 1.02 | 0.59 | 328110 | 11143 | 316967 | FALSE |
| 509.2 | Respiratory insufficiency | Respiratory | 0.99 | 0.94 | 1.03 | 0.59 | 318272 | 1788 | 316484 | FALSE |
| 480.1 | Bacterial pneumonia | Respiratory | 1.02 | 0.95 | 1.09 | 0.59 | 318628 | 939 | 317689 | FALSE |
| 378.2 | Nystagmus and other irregular eye movements | Sense Organs | 1.04 | 0.91 | 1.19 | 0.59 | 288407 | 215 | 288192 | FALSE |
| 454 | Varicose veins | Circulatory System | 0.99 | 0.94 | 1.04 | 0.59 | 289316 | 1603 | 287713 | FALSE |
| 823 | Fracture of tibia and fibula | Injuries & Poisonings | 1.03 | 0.93 | 1.15 | 0.59 | 322362 | 342 | 322020 | FALSE |
| 458.9 | Hypotension NOS | Circulatory System | 1.01 | 0.98 | 1.04 | 0.59 | 197817 | 3638 | 194179 | FALSE |
| 723 | Other disorders of cervical region | Musculoskeletal | 0.97 | 0.88 | 1.07 | 0.59 | 311345 | 401 | 310944 | FALSE |
| 341 | Other demyelinating diseases of central nervous system | Neurological | 0.99 | 0.94 | 1.04 | 0.60 | 287764 | 1612 | 286152 | FALSE |
| 532 | Dysphagia | Digestive | 0.99 | 0.97 | 1.02 | 0.60 | 294356 | 6553 | 287803 | FALSE |
| 961.1 | Poisoning/allergy of sulfonamides | Injuries & Poisonings | 0.98 | 0.92 | 1.05 | 0.60 | 301101 | 890 | 300211 | FALSE |
| 473 | Diseases of the larynx and vocal cords | Respiratory | 1.01 | 0.96 | 1.06 | 0.60 | 310564 | 1614 | 308950 | FALSE |
| 574.12 | Cholelithiasis with other cholecystitis | Digestive | 0.99 | 0.97 | 1.02 | 0.60 | 315816 | 5493 | 310323 | FALSE |
| 634.1 | Missed abortion/Hydatidiform mole | Pregnancy Complications | 1.02 | 0.96 | 1.08 | 0.60 | 321168 | 1203 | 319965 | FALSE |
| 155 | Cancer of liver and intrahepatic bile duct | Neoplasms | 0.97 | 0.85 | 1.10 | 0.60 | 312576 | 245 | 312331 | FALSE |
| 204 | Leukemia | Neoplasms | 0.98 | 0.89 | 1.07 | 0.60 | 323984 | 461 | 323523 | FALSE |
| 803.3 | Fracture of clavicle or scapula | Injuries & Poisonings | 0.97 | 0.87 | 1.09 | 0.60 | 323857 | 300 | 323557 | FALSE |

| | | | | | | | | | | |
|---|---|---|---|---|---|---|---|---|---|---|
| 614.32 | Chronic inflammatory pelvic disease | Genitourinary | 1.03 | 0.93 | 1.13 | 0.60 | 319224 | 425 | 318799 | FALSE |
| 246 | Other disorders of thyroid | Endocrine/Metabolic | 1.00 | 0.98 | 1.01 | 0.61 | 327738 | 17409 | 310329 | FALSE |
| 442.1 | Aortic aneurysm | Circulatory System | 0.98 | 0.90 | 1.06 | 0.61 | 319946 | 586 | 319360 | FALSE |
| 331.1 | Hydrocephalus | Neurological | 1.02 | 0.94 | 1.12 | 0.61 | 286652 | 500 | 286152 | FALSE |
| 560.3 | Peritoneal or intestinal adhesions | Digestive | 0.98 | 0.92 | 1.05 | 0.61 | 259257 | 861 | 258396 | FALSE |
| 340.1 | Migrain with aura | Neurological | 0.97 | 0.85 | 1.10 | 0.62 | 318167 | 217 | 317950 | FALSE |
| 528.11 | Stomatitis and mucositis (ulcerative) | Digestive | 0.98 | 0.89 | 1.08 | 0.62 | 322945 | 411 | 322534 | FALSE |
| 481 | Influenza | Respiratory | 0.99 | 0.98 | 1.02 | 0.62 | 327706 | 10017 | 317689 | FALSE |
| 195 | Cancer, suspected or other | Neoplasms | 0.98 | 0.92 | 1.05 | 0.62 | 230227 | 915 | 229312 | FALSE |
| 613.8 | Other specified disorders of breast | Genitourinary | 1.03 | 0.92 | 1.14 | 0.62 | 322773 | 355 | 322418 | FALSE |
| 361 | Retinal detachments and defects | Sense Organs | 1.01 | 0.97 | 1.05 | 0.62 | 319011 | 2229 | 316782 | FALSE |
| 292 | Neurological disorders | Mental Disorders | 0.98 | 0.89 | 1.07 | 0.62 | 321919 | 449 | 321470 | FALSE |
| 41.4 | E. coli | Infectious Diseases | 1.01 | 0.97 | 1.05 | 0.62 | 315432 | 2800 | 312632 | FALSE |
| 724.9 | Other unspecified back disorders | Musculoskeletal | 1.00 | 0.99 | 1.02 | 0.63 | 328029 | 17085 | 310944 | FALSE |
| 738 | Other acquired musculoskeletal deformity | Musculoskeletal | 0.98 | 0.89 | 1.08 | 0.63 | 317104 | 417 | 316687 | FALSE |
| 502 | Postinflammatory pulmonary fibrosis | Respiratory | 1.02 | 0.95 | 1.09 | 0.63 | 317367 | 883 | 316484 | FALSE |
| 628 | Ovarian cyst | Genitourinary | 1.01 | 0.98 | 1.04 | 0.63 | 301051 | 4820 | 296231 | FALSE |
| 721.1 | Spondylosis without myelopathy | Musculoskeletal | 0.99 | 0.93 | 1.05 | 0.63 | 312014 | 1070 | 310944 | FALSE |
| 568 | Other disorders of peritoneum | Digestive | 0.99 | 0.96 | 1.02 | 0.63 | 250464 | 3804 | 246660 | FALSE |
| 798 | Malaise and fatigue | Symptoms | 0.99 | 0.96 | 1.03 | 0.63 | 327695 | 2966 | 324729 | FALSE |
| 687.1 | Rash and other nonspecific skin eruption | Dermatologic | 0.99 | 0.95 | 1.03 | 0.63 | 325032 | 2218 | 322814 | FALSE |
| 454.1 | Varicose veins of lower extremity | Circulatory System | 1.00 | 0.98 | 1.01 | 0.63 | 299275 | 11562 | 287713 | FALSE |
| 318 | Tobacco use disorder | Mental Disorders | 1.00 | 0.99 | 1.02 | 0.64 | 318172 | 20503 | 297669 | FALSE |
| 701.2 | Scar conditions and fibrosis of skin | Dermatologic | 1.01 | 0.97 | 1.05 | 0.64 | 325471 | 2407 | 323064 | FALSE |
| 287.31 | Primary thrombocytopenia | Hematopoietic | 1.02 | 0.93 | 1.13 | 0.64 | 325925 | 428 | 325497 | FALSE |
| 530.5 | Disorders of esophageal motility | Digestive | 1.02 | 0.94 | 1.10 | 0.64 | 288452 | 649 | 287803 | FALSE |
| 526.41 | Temporomandibular joint disorder, unspecified | Digestive | 1.03 | 0.91 | 1.18 | 0.64 | 311886 | 228 | 311658 | FALSE |
| 800.1 | Fracture of neck of femur | Injuries & Poisonings | 0.97 | 0.85 | 1.10 | 0.64 | 323796 | 239 | 323557 | FALSE |
| 743.11 | Osteoporosis NOS | Musculoskeletal | 1.01 | 0.97 | 1.05 | 0.64 | 327235 | 3036 | 324199 | FALSE |
| 519.8 | Other diseases of respiratory system, NEC | Respiratory | 1.00 | 0.98 | 1.03 | 0.64 | 274529 | 9082 | 265447 | FALSE |
| 626.1 | Irregular menstrual cycle/bleeding | Genitourinary | 0.99 | 0.96 | 1.03 | 0.65 | 299824 | 3593 | 296231 | FALSE |
| 465.2 | Acute pharyngitis | Respiratory | 0.98 | 0.92 | 1.05 | 0.65 | 326053 | 874 | 325179 | FALSE |
| 289 | Other diseases of blood and blood-forming organs | Hematopoietic | 1.01 | 0.98 | 1.04 | 0.65 | 325300 | 4982 | 320318 | FALSE |
| 276.41 | Acidosis | Endocrine/Metabolic | 0.99 | 0.93 | 1.05 | 0.65 | 321700 | 1056 | 320644 | FALSE |
| 214.1 | Lipoma of skin and subcutaneous tissue | Neoplasms | 0.99 | 0.97 | 1.02 | 0.65 | 325432 | 4756 | 320676 | FALSE |
| 211 | Benign neoplasm of other parts of digestive system | Neoplasms | 0.99 | 0.97 | 1.02 | 0.65 | 319683 | 5375 | 314308 | FALSE |
| 433 | Cerebrovascular disease | Circulatory System | 1.00 | 0.97 | 1.02 | 0.65 | 326753 | 8646 | 318107 | FALSE |
| 721.8 | Other allied disorders of spine | Musculoskeletal | 1.01 | 0.97 | 1.04 | 0.65 | 314171 | 3227 | 310944 | FALSE |

| | | | | | | | | | | | |
|---|---|---|---|---|---|---|---|---|---|---|---|
| 536 | Disorders of function of stomach | Digestive | 1.03 | 0.91 | 1.17 | 0.65 | 296768 | 257 | 296511 | FALSE |
| 427.6 | Premature beats | Circulatory System | 1.02 | 0.93 | 1.12 | 0.65 | 299979 | 458 | 299521 | FALSE |
| 601.4 | Balanoposthitis | Genitourinary | 0.97 | 0.87 | 1.09 | 0.66 | 307689 | 296 | 307393 | FALSE |
| 752.11 | Spina bifida | Congenital Anomalies | 1.03 | 0.90 | 1.18 | 0.66 | 327886 | 211 | 327675 | FALSE |
| 558 | Noninfectious gastroenteritis | Digestive | 1.00 | 0.98 | 1.01 | 0.66 | 278260 | 19864 | 258396 | FALSE |
| 334 | Degenerative disease of the spinal cord | Neurological | 1.01 | 0.96 | 1.06 | 0.66 | 287631 | 1479 | 286152 | FALSE |
| 292.4 | Altered mental status | Mental Disorders | 1.01 | 0.97 | 1.05 | 0.66 | 323742 | 2272 | 321470 | FALSE |
| 284 | Aplastic anemia | Hematopoietic | 1.00 | 0.99 | 1.02 | 0.66 | 321659 | 12759 | 308900 | FALSE |
| 323.8 | Encephalitis, non-infectious | Neurological | 1.02 | 0.94 | 1.10 | 0.66 | 327771 | 633 | 327138 | FALSE |
| 528.6 | Leukoplakia of oral mucosa | Digestive | 1.03 | 0.92 | 1.15 | 0.66 | 322846 | 312 | 322534 | FALSE |
| 350.1 | Abnormal involuntary movements | Neurological | 0.99 | 0.92 | 1.05 | 0.67 | 326371 | 927 | 325444 | FALSE |
| 448 | Disease of capillaries | Circulatory System | 0.99 | 0.97 | 1.02 | 0.67 | 326841 | 7481 | 319360 | FALSE |
| 70.3 | Viral hepatitis C | Infectious Diseases | 0.98 | 0.88 | 1.09 | 0.67 | 322549 | 343 | 322206 | FALSE |
| 578.9 | Hemorrhage of gastrointestinal tract | Digestive | 1.01 | 0.98 | 1.03 | 0.67 | 308849 | 5455 | 303394 | FALSE |
| 332 | Parkinson's disease | Neurological | 1.01 | 0.96 | 1.07 | 0.67 | 287334 | 1182 | 286152 | FALSE |
| 522 | Diseases of pulp and periapical tissues | Digestive | 0.98 | 0.91 | 1.06 | 0.67 | 312297 | 639 | 311658 | FALSE |
| 427.12 | Paroxysmal ventricular tachycardia | Circulatory System | 0.99 | 0.93 | 1.05 | 0.67 | 300490 | 969 | 299521 | FALSE |
| 536.8 | Dyspepsia and other specified disorders of function of stomach | Digestive | 1.03 | 0.90 | 1.17 | 0.67 | 296758 | 247 | 296511 | FALSE |
| 531.1 | Hemorrhage from gastrointestinal ulcer | Digestive | 0.98 | 0.91 | 1.06 | 0.67 | 321204 | 655 | 320549 | FALSE |
| 535.6 | Duodenitis | Digestive | 1.00 | 0.98 | 1.03 | 0.68 | 304405 | 7894 | 296511 | FALSE |
| 594.2 | Calculus of lower urinary tract | Genitourinary | 1.01 | 0.95 | 1.09 | 0.68 | 320761 | 830 | 319931 | FALSE |
| 512.2 | Painful respiration | Respiratory | 0.97 | 0.85 | 1.11 | 0.68 | 314496 | 228 | 314268 | FALSE |
| 735.2 | Acquired toe deformities | Musculoskeletal | 1.01 | 0.97 | 1.05 | 0.68 | 318913 | 2226 | 316687 | FALSE |
| 251.1 | Hypoglycemia | Endocrine/Metabolic | 1.01 | 0.95 | 1.08 | 0.68 | 305952 | 977 | 304975 | FALSE |
| 681.5 | Cellulitis and abscess of leg, except foot | Dermatologic | 1.01 | 0.98 | 1.03 | 0.68 | 322184 | 5777 | 316407 | FALSE |
| 613.7 | Other signs and symptoms in breast | Genitourinary | 1.02 | 0.94 | 1.09 | 0.68 | 323169 | 751 | 322418 | FALSE |
| 316 | Substance addiction and disorders | Mental Disorders | 0.98 | 0.88 | 1.08 | 0.69 | 298042 | 373 | 297669 | FALSE |
| 537 | Other disorders of stomach and duodenum | Digestive | 1.01 | 0.97 | 1.04 | 0.69 | 299994 | 3483 | 296511 | FALSE |
| 610.1 | Cystic mastopathy | Genitourinary | 1.01 | 0.95 | 1.08 | 0.69 | 321832 | 959 | 320873 | FALSE |
| 568.1 | Peritoneal adhesions (postoperative) (postinfection) | Digestive | 0.99 | 0.96 | 1.03 | 0.69 | 249757 | 3097 | 246660 | FALSE |
| 530.1 | Esophagitis, GERD and related diseases | Digestive | 1.00 | 0.98 | 1.03 | 0.69 | 297342 | 9539 | 287803 | FALSE |
| 157 | Pancreatic cancer | Neoplasms | 1.02 | 0.94 | 1.10 | 0.69 | 312933 | 602 | 312331 | FALSE |
| 705.8 | Hyperhidrosis | Dermatologic | 1.02 | 0.94 | 1.10 | 0.69 | 318896 | 602 | 318294 | FALSE |
| 509.1 | Respiratory failure | Respiratory | 1.01 | 0.97 | 1.05 | 0.69 | 318564 | 2080 | 316484 | FALSE |
| 707.1 | Decubitus ulcer | Dermatologic | 1.02 | 0.92 | 1.14 | 0.69 | 327025 | 326 | 326699 | FALSE |
| 771 | Musculoskeletal symptoms referable to limbs | Symptoms | 0.99 | 0.92 | 1.05 | 0.69 | 327405 | 891 | 326514 | FALSE |
| 625 | Pain and other symptoms associated with female genital organs | Genitourinary | 1.01 | 0.97 | 1.05 | 0.70 | 320718 | 2049 | 318669 | FALSE |
| 751.21 | Cystic kidney disease | Congenital Anomalies | 1.02 | 0.93 | 1.12 | 0.70 | 326461 | 458 | 326003 | FALSE |

| | | | | | | | | | | |
|---|---|---|---|---|---|---|---|---|---|---|
| 277.4 | Disorders of bilirubin excretion | Endocrine/Metabolic | 0.98 | 0.89 | 1.08 | 0.70 | 283300 | 373 | 282927 | FALSE |
| 428.1 | Congestive heart failure (CHF) NOS | Circulatory System | 1.01 | 0.97 | 1.05 | 0.70 | 324086 | 2053 | 322033 | FALSE |
| 296.1 | Bipolar | Mental Disorders | 0.99 | 0.93 | 1.05 | 0.70 | 284019 | 1121 | 282898 | FALSE |
| 227.3 | Benign neoplasm of pituitary gland and craniopharyngeal duct (pouch) | Neoplasms | 0.98 | 0.88 | 1.09 | 0.71 | 326970 | 328 | 326642 | FALSE |
| 458 | Hypotension | Circulatory System | 0.99 | 0.93 | 1.05 | 0.71 | 195260 | 1081 | 194179 | FALSE |
| 302 | Sexual and gender identity disorders | Mental Disorders | 1.02 | 0.92 | 1.14 | 0.71 | 283238 | 340 | 282898 | FALSE |
| 555 | Inflammatory bowel disease and other gastroenteritis and colitis | Digestive | 1.00 | 0.98 | 1.01 | 0.71 | 278195 | 19799 | 258396 | FALSE |
| 530.9 | Heartburn | Digestive | 1.01 | 0.97 | 1.05 | 0.71 | 289911 | 2108 | 287803 | FALSE |
| 960.2 | Allergy/adverse effect of penicillin | Injuries & Poisonings | 1.00 | 0.98 | 1.01 | 0.71 | 316894 | 16683 | 300211 | FALSE |
| 349 | Other and unspecified disorders of the nervous system | Neurological | 1.00 | 0.99 | 1.01 | 0.71 | 326855 | 40703 | 286152 | FALSE |
| 740.9 | Osteoarthrosis NOS | Musculoskeletal | 1.01 | 0.98 | 1.04 | 0.71 | 310757 | 4432 | 306325 | FALSE |
| 686 | Other local infections of skin and subcutaneous tissue | Dermatologic | 1.00 | 0.98 | 1.02 | 0.71 | 327410 | 11003 | 316407 | FALSE |
| 317.1 | Alcoholism | Mental Disorders | 1.00 | 0.97 | 1.02 | 0.71 | 306366 | 8697 | 297669 | FALSE |
| 385.3 | Cholesteatoma | Sense Organs | 1.02 | 0.94 | 1.10 | 0.71 | 324732 | 589 | 324143 | FALSE |
| 575.9 | Nonspecific abnormal findings on radiological and other examination of biliary tract | Digestive | 1.02 | 0.92 | 1.13 | 0.72 | 310690 | 367 | 310323 | FALSE |
| 367.8 | Hypermetropia | Sense Organs | 0.98 | 0.86 | 1.11 | 0.72 | 325995 | 241 | 325754 | FALSE |
| 528.5 | Diseases of lips | Digestive | 0.99 | 0.91 | 1.06 | 0.72 | 323215 | 681 | 322534 | FALSE |
| 529.1 | Glossitis | Digestive | 1.02 | 0.91 | 1.14 | 0.72 | 322838 | 304 | 322534 | FALSE |
| 716.9 | Arthropathy NOS | Musculoskeletal | 1.00 | 0.99 | 1.01 | 0.72 | 327719 | 53823 | 273896 | FALSE |
| 427.5 | Arrhythmia (cardiac) NOS | Circulatory System | 1.01 | 0.95 | 1.08 | 0.73 | 300469 | 948 | 299521 | FALSE |
| 727.5 | Rupture of synovium | Musculoskeletal | 1.02 | 0.92 | 1.13 | 0.73 | 305298 | 344 | 304954 | FALSE |
| 961 | Poisoning by other anti-infectives | Injuries & Poisonings | 0.98 | 0.90 | 1.08 | 0.73 | 300647 | 436 | 300211 | FALSE |
| 315 | Develomental delays and disorders | Mental Disorders | 0.99 | 0.91 | 1.07 | 0.73 | 326992 | 549 | 326443 | FALSE |
| 458.1 | Orthostatic hypotension | Circulatory System | 1.01 | 0.96 | 1.06 | 0.74 | 195553 | 1374 | 194179 | FALSE |
| 378.5 | Paralytic strabismus | Sense Organs | 1.02 | 0.91 | 1.15 | 0.74 | 288479 | 287 | 288192 | FALSE |
| 694.2 | Other dyschromia | Dermatologic | 0.99 | 0.92 | 1.06 | 0.74 | 322549 | 800 | 321749 | FALSE |
| 189.11 | Malignant neoplasm of kidney, except pelvis | Neoplasms | 0.99 | 0.93 | 1.05 | 0.74 | 324997 | 1035 | 323962 | FALSE |
| 418.1 | Precordial pain | Circulatory System | 1.01 | 0.97 | 1.04 | 0.74 | 299506 | 3686 | 295820 | FALSE |
| 761 | Cervicalgia | Symptoms | 0.99 | 0.93 | 1.05 | 0.74 | 328240 | 1005 | 327235 | FALSE |
| 345.11 | Generalized convulsive epilepsy | Neurological | 0.98 | 0.90 | 1.08 | 0.74 | 286617 | 465 | 286152 | FALSE |
| 735.21 | Hammer toe (acquired) | Musculoskeletal | 0.99 | 0.95 | 1.04 | 0.74 | 318701 | 2014 | 316687 | FALSE |
| 599.3 | Dysuria | Genitourinary | 1.01 | 0.95 | 1.07 | 0.74 | 221636 | 1209 | 220427 | FALSE |
| 964.1 | Anticoagulants causing adverse effects | Injuries & Poisonings | 1.02 | 0.90 | 1.15 | 0.74 | 300470 | 259 | 300211 | FALSE |
| 202 | Cancer of other lymphoid, histiocytic tissue | Neoplasms | 0.99 | 0.93 | 1.05 | 0.74 | 324661 | 1138 | 323523 | FALSE |
| 175 | Acquired absence of breast | Neoplasms | 0.99 | 0.95 | 1.03 | 0.74 | 305907 | 2516 | 303391 | FALSE |
| 261.2 | Vitamin B-complex deficiencies | Endocrine/Metabolic | 1.01 | 0.94 | 1.09 | 0.74 | 326559 | 773 | 325786 | FALSE |
| 362.2 | Degeneration of macula and posterior pole of retina | Sense Organs | 0.99 | 0.95 | 1.04 | 0.75 | 318055 | 2203 | 315852 | FALSE |
| 253.2 | Pituitary hypofunction | Endocrine/Metabolic | 0.98 | 0.87 | 1.10 | 0.75 | 324826 | 281 | 324545 | FALSE |

| | | | | | | | | | | |
|---|---|---|---|---|---|---|---|---|---|---|
| 575.8 | Other disorders of biliary tract | Digestive | 0.99 | 0.93 | 1.05 | 0.75 | 311372 | 1049 | 310323 | FALSE |
| 396 | Abnormal heart sounds | Circulatory System | 1.01 | 0.95 | 1.07 | 0.75 | 322603 | 1075 | 321528 | FALSE |
| 367.9 | Blindness and low vision | Sense Organs | 1.01 | 0.94 | 1.09 | 0.75 | 326510 | 756 | 325754 | FALSE |
| 557.1 | Celiac disease | Digestive | 0.99 | 0.95 | 1.04 | 0.75 | 260290 | 1894 | 258396 | FALSE |
| 386.2 | Peripheral or central vertigo | Sense Organs | 0.98 | 0.89 | 1.09 | 0.75 | 322036 | 387 | 321649 | FALSE |
| 81 | Infection/inflammation of internal prosthetic device; implant; and graft | Infectious Diseases | 0.99 | 0.96 | 1.03 | 0.75 | 323993 | 2514 | 321479 | FALSE |
| 540.1 | Appendicitis | Digestive | 0.99 | 0.92 | 1.06 | 0.75 | 325174 | 795 | 324379 | FALSE |
| 317 | Alcohol-related disorders | Mental Disorders | 1.00 | 0.98 | 1.03 | 0.75 | 303529 | 5860 | 297669 | FALSE |
| 300.12 | Agorophobia, social phobia, and panic disorder | Mental Disorders | 0.99 | 0.92 | 1.06 | 0.75 | 283642 | 744 | 282898 | FALSE |
| 250.24 | Type 2 diabetes with neurological manifestations | Endocrine/Metabolic | 1.01 | 0.94 | 1.10 | 0.76 | 308102 | 612 | 307490 | FALSE |
| 464 | Acute sinusitis | Respiratory | 1.02 | 0.89 | 1.17 | 0.76 | 325387 | 208 | 325179 | FALSE |
| 368.2 | Diplopia and disorders of binocular vision | Sense Organs | 0.99 | 0.92 | 1.06 | 0.76 | 325635 | 753 | 324882 | FALSE |
| 722 | Intervertebral disc disorders | Musculoskeletal | 1.00 | 0.98 | 1.03 | 0.76 | 316907 | 5963 | 310944 | FALSE |
| 681.2 | Cellulitis and abscess of face/neck | Dermatologic | 0.99 | 0.91 | 1.08 | 0.76 | 316936 | 529 | 316407 | FALSE |
| 571.5 | Other chronic nonalcoholic liver disease | Digestive | 1.01 | 0.96 | 1.06 | 0.76 | 320004 | 1673 | 318331 | FALSE |
| 540.11 | Acute appendicitis | Digestive | 1.01 | 0.97 | 1.05 | 0.77 | 327037 | 2658 | 324379 | FALSE |
| 870 | Open wounds of head; neck; and trunk | Injuries & Poisonings | 0.99 | 0.91 | 1.07 | 0.77 | 320327 | 596 | 319731 | FALSE |
| 334.2 | Anterior horn cell disease | Neurological | 0.98 | 0.86 | 1.12 | 0.77 | 286376 | 224 | 286152 | FALSE |
| 592.13 | Chronic interstitial cystitis | Genitourinary | 0.98 | 0.87 | 1.11 | 0.77 | 297289 | 254 | 297035 | FALSE |
| 366 | Cataract | Sense Organs | 1.00 | 0.98 | 1.01 | 0.77 | 323825 | 16441 | 307384 | FALSE |
| 362.29 | Macular degeneration (senile) of retina NOS | Sense Organs | 0.99 | 0.95 | 1.04 | 0.77 | 318052 | 2200 | 315852 | FALSE |
| 442.11 | Abdominal aortic aneurysm | Circulatory System | 1.01 | 0.95 | 1.08 | 0.77 | 320257 | 897 | 319360 | FALSE |
| 622 | Polyp of female genital organs | Genitourinary | 0.99 | 0.90 | 1.08 | 0.77 | 315794 | 487 | 315307 | FALSE |
| 625.1 | Dyspareunia | Genitourinary | 1.01 | 0.95 | 1.07 | 0.77 | 319824 | 1155 | 318669 | FALSE |
| 512.8 | Cough | Respiratory | 1.01 | 0.97 | 1.04 | 0.77 | 317232 | 2964 | 314268 | FALSE |
| 751.2 | Congenital anomalies of urinary system | Congenital Anomalies | 1.01 | 0.95 | 1.08 | 0.78 | 326908 | 905 | 326003 | FALSE |
| 465 | Acute upper respiratory infections of multiple or unspecified sites | Respiratory | 0.99 | 0.96 | 1.03 | 0.78 | 328232 | 3053 | 325179 | FALSE |
| 601.11 | Acute prostatitis | Genitourinary | 1.02 | 0.90 | 1.15 | 0.78 | 307653 | 260 | 307393 | FALSE |
| 669 | Complications of labor and delivery NEC | Pregnancy Complications | 1.00 | 0.97 | 1.02 | 0.78 | 328240 | 9534 | 318706 | FALSE |
| 244.4 | Hypothyroidism NOS | Endocrine/Metabolic | 1.00 | 0.99 | 1.02 | 0.78 | 324759 | 14430 | 310329 | FALSE |
| 807 | Fracture of ribs | Injuries & Poisonings | 0.98 | 0.86 | 1.12 | 0.78 | 323780 | 223 | 323557 | FALSE |
| 728.7 | Fasciitis | Musculoskeletal | 1.02 | 0.89 | 1.17 | 0.78 | 305167 | 213 | 304954 | FALSE |
| 601.8 | Other inflammatory disorders of male genital organs | Genitourinary | 1.02 | 0.91 | 1.14 | 0.78 | 307698 | 305 | 307393 | FALSE |
| 426.31 | Right bundle branch block | Circulatory System | 0.99 | 0.94 | 1.04 | 0.78 | 301094 | 1573 | 299521 | FALSE |
| 381.1 | Otitis media | Sense Organs | 0.99 | 0.94 | 1.05 | 0.78 | 325296 | 1153 | 324143 | FALSE |
| 217.1 | Nevus, non-neoplastic | Neoplasms | 1.01 | 0.93 | 1.10 | 0.78 | 320346 | 597 | 319749 | FALSE |
| 371.3 | Inflammation of eyelids | Sense Organs | 0.99 | 0.96 | 1.04 | 0.78 | 320786 | 2398 | 318388 | FALSE |
| 854 | Complications of cardiac/vascular device, implant, and graft | Injuries & Poisonings | 0.99 | 0.95 | 1.04 | 0.79 | 315807 | 1843 | 313964 | FALSE |

| | | | | | | | | | | |
|---|---|---|---|---|---|---|---|---|---|---|
| 835 | Internal derangement of knee | Injuries & Poisonings | 1.00 | 0.97 | 1.04 | 0.79 | 326012 | 4239 | 321773 | FALSE |
| 512.1 | Wheezing | Respiratory | 1.02 | 0.89 | 1.16 | 0.79 | 314499 | 231 | 314268 | FALSE |
| 361.1 | Retinal detachment with retinal defect | Sense Organs | 0.99 | 0.94 | 1.05 | 0.79 | 318238 | 1456 | 316782 | FALSE |
| 573.9 | Abnormal serum enzyme levels | Digestive | 1.02 | 0.89 | 1.16 | 0.79 | 318561 | 230 | 318331 | FALSE |
| 218.2 | Other benign neoplasm of uterus | Neoplasms | 0.99 | 0.89 | 1.09 | 0.79 | 308143 | 363 | 307780 | FALSE |
| 587 | Kidney replaced by transpant | Genitourinary | 1.01 | 0.92 | 1.12 | 0.79 | 314477 | 398 | 314079 | FALSE |
| 285.2 | Anemia of chronic disease | Hematopoietic | 1.02 | 0.91 | 1.14 | 0.79 | 309194 | 294 | 308900 | FALSE |
| 474.1 | Acute tonsillitis | Respiratory | 1.01 | 0.93 | 1.10 | 0.79 | 309515 | 565 | 308950 | FALSE |
| 288.11 | Neutropenia | Hematopoietic | 1.00 | 0.97 | 1.04 | 0.79 | 323644 | 3326 | 320318 | FALSE |
| 979 | Adverse drug events and drug allergies | Injuries & Poisonings | 1.01 | 0.94 | 1.09 | 0.79 | 300927 | 716 | 300211 | FALSE |
| 526.9 | Jaw disease NOS | Digestive | 1.00 | 0.99 | 1.02 | 0.79 | 327620 | 15962 | 311658 | FALSE |
| 646 | Other complications of pregnancy NEC | Pregnancy Complications | 1.01 | 0.96 | 1.05 | 0.79 | 328240 | 2396 | 325844 | FALSE |
| 947 | Urticaria | Injuries & Poisonings | 0.99 | 0.89 | 1.09 | 0.80 | 322293 | 387 | 321906 | FALSE |
| 687 | Symptoms affecting skin | Dermatologic | 1.02 | 0.89 | 1.16 | 0.80 | 323032 | 218 | 322814 | FALSE |
| 368.4 | Visual field defects | Sense Organs | 1.01 | 0.91 | 1.13 | 0.80 | 325215 | 333 | 324882 | FALSE |
| 681.6 | Cellulitis and abscess of foot, toe | Dermatologic | 1.00 | 0.98 | 1.03 | 0.80 | 322141 | 5734 | 316407 | FALSE |
| 272.1 | Hyperlipidemia | Endocrine/Metabolic | 1.00 | 0.97 | 1.02 | 0.80 | 296728 | 5231 | 291497 | FALSE |
| 624.2 | Atrophy of female genital tract | Genitourinary | 1.01 | 0.92 | 1.11 | 0.80 | 319111 | 442 | 318669 | FALSE |
| 724.2 | Disorders of coccyx | Musculoskeletal | 1.02 | 0.90 | 1.14 | 0.80 | 311226 | 282 | 310944 | FALSE |
| 579.2 | Splenomegaly | Digestive | 0.99 | 0.90 | 1.08 | 0.80 | 303837 | 443 | 303394 | FALSE |
| 627 | Menopausal and postmenopausal disorders | Genitourinary | 1.01 | 0.94 | 1.08 | 0.80 | 297078 | 847 | 296231 | FALSE |
| 681.3 | Cellulitis and abscess of arm/hand | Dermatologic | 1.00 | 0.98 | 1.03 | 0.80 | 322177 | 5770 | 316407 | FALSE |
| 198.4 | Secondary malignant neoplasm of liver | Neoplasms | 1.00 | 0.97 | 1.04 | 0.80 | 232009 | 2697 | 229312 | FALSE |
| 870.4 | Open wound of nose and sinus | Injuries & Poisonings | 0.98 | 0.87 | 1.12 | 0.80 | 319969 | 238 | 319731 | FALSE |
| 526 | Diseases of the jaws | Digestive | 1.00 | 0.99 | 1.02 | 0.81 | 327645 | 15987 | 311658 | FALSE |
| 250.22 | Type 2 diabetes with renal manifestations | Endocrine/Metabolic | 1.02 | 0.89 | 1.17 | 0.81 | 307698 | 208 | 307490 | FALSE |
| 507 | Pleurisy; pleural effusion | Respiratory | 1.00 | 0.97 | 1.02 | 0.81 | 323091 | 6607 | 316484 | FALSE |
| 352.1 | Trigeminal nerve disorders [CN5] | Neurological | 0.99 | 0.90 | 1.08 | 0.81 | 313473 | 462 | 313011 | FALSE |
| 526.1 | Cysts of the jaws | Digestive | 0.99 | 0.89 | 1.10 | 0.81 | 311993 | 335 | 311658 | FALSE |
| 443.7 | Peripheral angiopathy in diseases classified elsewhere | Circulatory System | 0.99 | 0.90 | 1.09 | 0.81 | 319775 | 415 | 319360 | FALSE |
| 716.1 | Unspecified polyarthropathy or polyarthritis | Musculoskeletal | 1.00 | 0.96 | 1.03 | 0.82 | 277426 | 3530 | 273896 | FALSE |
| 506 | Empyema and pneumothorax | Respiratory | 0.99 | 0.94 | 1.05 | 0.82 | 317630 | 1146 | 316484 | FALSE |
| 701 | Other hypertrophic and atrophic conditions of skin | Dermatologic | 1.01 | 0.96 | 1.05 | 0.82 | 324891 | 1827 | 323064 | FALSE |
| 760 | Back pain | Symptoms | 1.00 | 0.98 | 1.03 | 0.82 | 328240 | 5923 | 322317 | FALSE |
| 530.12 | Ulcer of esophagus | Digestive | 1.00 | 0.97 | 1.02 | 0.82 | 293151 | 5348 | 287803 | FALSE |
| 592.1 | Cystitis | Genitourinary | 1.01 | 0.96 | 1.05 | 0.82 | 299089 | 2054 | 297035 | FALSE |
| 949 | Allergies, other | Injuries & Poisonings | 0.99 | 0.90 | 1.09 | 0.82 | 322326 | 420 | 321906 | FALSE |
| 614.54 | Abscess or ulceration of vulva | Genitourinary | 0.99 | 0.88 | 1.11 | 0.82 | 319079 | 280 | 318799 | FALSE |

| | | | | | | | | | | | | |
|---|---|---|---|---|---|---|---|---|---|---|---|---|
| 803.1 | Fracture of humerus | Injuries & Poisonings | 1.01 | 0.90 | 1.14 | 0.82 | 323850 | 293 | 323557 | FALSE |
| 340 | Migraine | Neurological | 1.00 | 0.97 | 1.04 | 0.82 | 320723 | 2773 | 317950 | FALSE |
| 578.1 | Hematemesis | Digestive | 1.01 | 0.96 | 1.05 | 0.82 | 305428 | 2034 | 303394 | FALSE |
| 715.2 | Ankylosing spondylitis | Musculoskeletal | 1.01 | 0.91 | 1.12 | 0.82 | 317637 | 384 | 317253 | FALSE |
| 564 | Functional digestive disorders | Digestive | 1.01 | 0.95 | 1.07 | 0.82 | 259379 | 983 | 258396 | FALSE |
| 433.12 | Cerebral atherosclerosis | Circulatory System | 0.99 | 0.87 | 1.12 | 0.83 | 318345 | 238 | 318107 | FALSE |
| 352.2 | Facial nerve disorders [CN7] | Neurological | 0.99 | 0.93 | 1.06 | 0.83 | 313911 | 900 | 313011 | FALSE |
| 365.11 | Primary open angle glaucoma | Sense Organs | 1.01 | 0.95 | 1.07 | 0.83 | 317838 | 1056 | 316782 | FALSE |
| 957 | Injury to other and unspecified nerves | Injuries & Poisonings | 0.99 | 0.88 | 1.11 | 0.83 | 328157 | 275 | 327882 | FALSE |
| 433.5 | Cerebral aneurysm | Circulatory System | 0.99 | 0.90 | 1.09 | 0.83 | 318505 | 398 | 318107 | FALSE |
| 496.3 | Bronchiectasis | Respiratory | 1.00 | 0.96 | 1.05 | 0.83 | 296077 | 1885 | 294192 | FALSE |
| 801.1 | Fracture of foot | Injuries & Poisonings | 1.01 | 0.92 | 1.11 | 0.84 | 323975 | 418 | 323557 | FALSE |
| 702.2 | Seborrheic keratosis | Dermatologic | 1.00 | 0.97 | 1.04 | 0.84 | 325769 | 3169 | 322600 | FALSE |
| 655 | Known or suspected fetal abnormality affecting management of mother | Pregnancy Complications | 1.00 | 0.97 | 1.04 | 0.84 | 328240 | 4575 | 323665 | FALSE |
| 395.2 | Nonrheumatic aortic valve disorders | Circulatory System | 0.99 | 0.87 | 1.12 | 0.84 | 321775 | 247 | 321528 | FALSE |
| 427.42 | Cardiac arrest | Circulatory System | 1.01 | 0.94 | 1.07 | 0.85 | 300470 | 949 | 299521 | FALSE |
| 740.12 | Osteoarthrosis, localized, secondary | Musculoskeletal | 1.01 | 0.90 | 1.14 | 0.85 | 306589 | 264 | 306325 | FALSE |
| 686.1 | Carbuncle and furuncle | Dermatologic | 1.00 | 0.96 | 1.05 | 0.85 | 318829 | 2422 | 316407 | FALSE |
| 737.3 | Kyphoscoliosis and scoliosis | Musculoskeletal | 0.99 | 0.89 | 1.10 | 0.85 | 317058 | 371 | 316687 | FALSE |
| 727 | Other disorders of synovium, tendon, and bursa | Musculoskeletal | 0.99 | 0.92 | 1.07 | 0.85 | 305708 | 754 | 304954 | FALSE |
| 681 | Superficial cellulitis and abscess | Dermatologic | 0.99 | 0.92 | 1.07 | 0.85 | 317135 | 728 | 316407 | FALSE |
| 384.4 | Perforation of tympanic membrane | Sense Organs | 1.01 | 0.95 | 1.07 | 0.85 | 325167 | 1024 | 324143 | FALSE |
| 288 | Diseases of white blood cells | Hematopoietic | 1.01 | 0.93 | 1.09 | 0.85 | 320928 | 610 | 320318 | FALSE |
| 751.1 | Congenital anomalies of genital organs | Congenital Anomalies | 0.99 | 0.92 | 1.07 | 0.85 | 326709 | 706 | 326003 | FALSE |
| 709.2 | Sicca syndrome | Dermatologic | 0.99 | 0.91 | 1.08 | 0.86 | 242486 | 520 | 241966 | FALSE |
| 452 | Other venous embolism and thrombosis | Circulatory System | 1.01 | 0.93 | 1.10 | 0.86 | 288267 | 554 | 287713 | FALSE |
| 371 | Inflammation of the eye | Sense Organs | 1.01 | 0.92 | 1.11 | 0.86 | 318796 | 408 | 318388 | FALSE |
| 287.3 | Thrombocytopenia | Hematopoietic | 1.00 | 0.95 | 1.06 | 0.86 | 326768 | 1271 | 325497 | FALSE |
| 289.8 | Polycythemia vera, secondary | Hematopoietic | 1.01 | 0.90 | 1.13 | 0.86 | 320039 | 291 | 319748 | FALSE |
| 626.8 | Infertility, female | Genitourinary | 1.00 | 0.94 | 1.05 | 0.86 | 297638 | 1407 | 296231 | FALSE |
| 174 | Breast cancer | Neoplasms | 0.99 | 0.88 | 1.11 | 0.86 | 304712 | 288 | 304424 | FALSE |
| 38.1 | Gram negative septicemia | Infectious Diseases | 0.99 | 0.93 | 1.06 | 0.86 | 313487 | 855 | 312632 | FALSE |
| 426.24 | Atrioventricular block, complete | Circulatory System | 0.99 | 0.92 | 1.08 | 0.86 | 300112 | 591 | 299521 | FALSE |
| 750 | Digestive congenital anomalies | Congenital Anomalies | 0.99 | 0.92 | 1.07 | 0.87 | 326705 | 702 | 326003 | FALSE |
| 724.1 | Disorders of sacrum | Musculoskeletal | 1.01 | 0.90 | 1.14 | 0.87 | 311225 | 281 | 310944 | FALSE |
| 698 | Pruritus and related conditions | Dermatologic | 1.01 | 0.94 | 1.08 | 0.87 | 328240 | 799 | 327441 | FALSE |
| 752 | Nervous system congenital anomalies | Congenital Anomalies | 1.01 | 0.90 | 1.13 | 0.87 | 327963 | 288 | 327675 | FALSE |
| 368.1 | Amblyopia | Sense Organs | 1.01 | 0.93 | 1.10 | 0.87 | 325423 | 541 | 324882 | FALSE |

| | | | | | | | | | | |
|---|---|---|---|---|---|---|---|---|---|---|
| 411.9 | Other acute and subacute forms of ischemic heart disease | Circulatory System | 1.00 | 0.95 | 1.06 | 0.87 | 296883 | 1194 | 295689 | FALSE |
| 401.22 | Hypertensive chronic kidney disease | Circulatory System | 1.00 | 0.95 | 1.05 | 0.87 | 250343 | 1595 | 248748 | FALSE |
| 531.4 | Peptic ulcer, site unspecified | Digestive | 0.99 | 0.90 | 1.09 | 0.87 | 320998 | 449 | 320549 | FALSE |
| 601.12 | Chronic prostatitis | Genitourinary | 0.99 | 0.93 | 1.06 | 0.87 | 308341 | 948 | 307393 | FALSE |
| 619.3 | Noninflammatory disorders of cervix | Genitourinary | 1.00 | 0.97 | 1.04 | 0.87 | 281393 | 2845 | 278548 | FALSE |
| 585.1 | Acute renal failure | Genitourinary | 1.00 | 0.97 | 1.03 | 0.87 | 318744 | 4665 | 314079 | FALSE |
| 614.52 | Vaginitis and vulvovaginitis | Genitourinary | 1.01 | 0.92 | 1.11 | 0.87 | 319219 | 420 | 318799 | FALSE |
| 218 | Benign neoplasm of uterus | Neoplasms | 0.99 | 0.90 | 1.10 | 0.88 | 308168 | 388 | 307780 | FALSE |
| 244.1 | Secondary hypothyroidism | Endocrine/Metabolic | 1.00 | 0.95 | 1.06 | 0.88 | 311484 | 1155 | 310329 | FALSE |
| 454.11 | Varicose veins of lower extremity, symptomtic | Circulatory System | 1.01 | 0.93 | 1.09 | 0.88 | 288365 | 652 | 287713 | FALSE |
| 368.9 | Subjective visual disturbances | Sense Organs | 0.99 | 0.92 | 1.07 | 0.88 | 325515 | 633 | 324882 | FALSE |
| 574.11 | Cholelithiasis with acute cholecystitis | Digestive | 1.00 | 0.95 | 1.06 | 0.88 | 311836 | 1513 | 310323 | FALSE |
| 615 | Endometriosis | Genitourinary | 1.00 | 0.97 | 1.03 | 0.88 | 322888 | 4089 | 318799 | FALSE |
| 709.7 | Unspecified diffuse connective tissue disease | Dermatologic | 1.00 | 0.99 | 1.01 | 0.88 | 324635 | 82669 | 241966 | FALSE |
| 619.1 | Noninflammatory disorders of ovary, fallopian tube, and broad ligament | Genitourinary | 0.99 | 0.92 | 1.07 | 0.89 | 279282 | 734 | 278548 | FALSE |
| 622.1 | Polyp of corpus uteri | Genitourinary | 1.00 | 0.98 | 1.02 | 0.89 | 323490 | 8183 | 315307 | FALSE |
| 990 | Effects radiation NOS | Injuries & Poisonings | 1.00 | 0.97 | 1.04 | 0.90 | 325633 | 3244 | 322389 | FALSE |
| 596 | Other disorders of bladder | Genitourinary | 1.00 | 0.98 | 1.02 | 0.90 | 321385 | 7749 | 313636 | FALSE |
| 635.2 | Antepartum hemorrhage, abruptio placentae, and placenta previa | Pregnancy Complications | 1.00 | 0.94 | 1.07 | 0.90 | 320901 | 936 | 319965 | FALSE |
| 483 | Acute bronchitis and bronchiolitis | Respiratory | 1.01 | 0.88 | 1.16 | 0.90 | 317889 | 200 | 317689 | FALSE |
| 198.5 | Secondary malignancy of brain/spine | Neoplasms | 1.00 | 0.93 | 1.07 | 0.90 | 230113 | 801 | 229312 | FALSE |
| 272.9 | Unspecified disorder of lipoid metabolism | Endocrine/Metabolic | 1.01 | 0.88 | 1.15 | 0.91 | 291721 | 224 | 291497 | FALSE |
| 335 | Multiple sclerosis | Neurological | 1.00 | 0.95 | 1.05 | 0.91 | 287531 | 1379 | 286152 | FALSE |
| 621 | Endometrial hyperplasia | Genitourinary | 1.00 | 0.94 | 1.06 | 0.91 | 316461 | 1154 | 315307 | FALSE |
| 200.1 | Polycythemia vera | Neoplasms | 1.01 | 0.91 | 1.11 | 0.91 | 318136 | 404 | 317732 | FALSE |
| 510 | Other diseases of lung | Respiratory | 1.00 | 0.94 | 1.08 | 0.91 | 328200 | 770 | 327430 | FALSE |
| 198.1 | Secondary malignancy of lymph nodes | Neoplasms | 1.00 | 0.98 | 1.03 | 0.91 | 234815 | 5503 | 229312 | FALSE |
| 853 | Complication of colostomy or enterostomy | Injuries & Poisonings | 0.99 | 0.91 | 1.09 | 0.91 | 314478 | 514 | 313964 | FALSE |
| 221 | Benign neoplasm of other female genital organs | Neoplasms | 0.99 | 0.87 | 1.13 | 0.91 | 295974 | 223 | 295751 | FALSE |
| 573.3 | Hepatomegaly | Digestive | 0.99 | 0.89 | 1.11 | 0.91 | 318641 | 310 | 318331 | FALSE |
| 41.1 | Staphylococcus infections | Infectious Diseases | 1.00 | 0.96 | 1.03 | 0.91 | 315832 | 3200 | 312632 | FALSE |
| 280.2 | Iron deficiency anemia secondary to blood loss (chronic) | Hematopoietic | 1.00 | 0.92 | 1.10 | 0.91 | 309376 | 476 | 308900 | FALSE |
| 279.7 | Other immunological findings | Endocrine/Metabolic | 1.01 | 0.89 | 1.14 | 0.91 | 327978 | 264 | 327714 | FALSE |
| 512 | Other symptoms of respiratory system | Respiratory | 1.00 | 0.97 | 1.03 | 0.91 | 319772 | 5504 | 314268 | FALSE |
| 312 | Conduct disorders | Mental Disorders | 1.00 | 0.95 | 1.06 | 0.91 | 327638 | 1195 | 326443 | FALSE |
| 331 | Other cerebral degenerations | Neurological | 0.99 | 0.87 | 1.13 | 0.92 | 286392 | 240 | 286152 | FALSE |
| 597 | Other disorders of urethra and urinary tract | Genitourinary | 1.00 | 0.93 | 1.07 | 0.92 | 314510 | 874 | 313636 | FALSE |
| 200 | Myeloproliferative disease | Neoplasms | 1.00 | 0.93 | 1.08 | 0.92 | 324202 | 679 | 323523 | FALSE |

| | | | | | | | | | | |
|---|---|---|---|---|---|---|---|---|---|---|
| 736 | Other acquired deformities of limbs | Musculoskeletal | 0.99 | 0.89 | 1.11 | 0.92 | 316998 | 311 | 316687 | FALSE |
| 626 | Disorders of menstruation and other abnormal bleeding from female genital tract | Genitourinary | 1.00 | 0.97 | 1.03 | 0.92 | 300178 | 3947 | 296231 | FALSE |
| 348.2 | Cerebral edema and compression of brain | Neurological | 0.99 | 0.88 | 1.12 | 0.92 | 286412 | 260 | 286152 | FALSE |
| 442.8 | Aneurysm of other specified artery | Circulatory System | 1.01 | 0.88 | 1.15 | 0.92 | 319570 | 210 | 319360 | FALSE |
| 618.2 | Uterine/Uterovaginal prolapse | Genitourinary | 1.00 | 0.97 | 1.03 | 0.92 | 321713 | 5471 | 316242 | FALSE |
| 384 | Other disorders of tympanic membrane | Sense Organs | 1.00 | 0.91 | 1.11 | 0.92 | 324573 | 430 | 324143 | FALSE |
| 573.7 | Abnormal results of function study of liver | Digestive | 1.00 | 0.97 | 1.03 | 0.92 | 321896 | 3565 | 318331 | FALSE |
| 292.6 | Hallucinations | Mental Disorders | 1.01 | 0.90 | 1.12 | 0.93 | 321793 | 323 | 321470 | FALSE |
| 819 | Skull and face fracture and other intercranial injury | Injuries & Poisonings | 1.00 | 0.95 | 1.05 | 0.93 | 328056 | 1517 | 326539 | FALSE |
| 369 | Infection of the eye | Sense Organs | 0.99 | 0.88 | 1.12 | 0.93 | 318642 | 254 | 318388 | FALSE |
| 575.7 | Other disorders of gallbladder | Digestive | 1.00 | 0.95 | 1.05 | 0.93 | 311719 | 1396 | 310323 | FALSE |
| 523.31 | Acute periodontitis | Digestive | 1.00 | 0.93 | 1.08 | 0.93 | 312371 | 713 | 311658 | FALSE |
| 367.2 | Astigmatism | Sense Organs | 1.01 | 0.88 | 1.15 | 0.93 | 325964 | 210 | 325754 | FALSE |
| 697 | Sarcoidosis | Dermatologic | 1.00 | 0.92 | 1.09 | 0.93 | 322309 | 560 | 321749 | FALSE |
| 480.5 | Bronchopneumonia and lung abscess | Respiratory | 1.00 | 0.90 | 1.10 | 0.93 | 318077 | 388 | 317689 | FALSE |
| 556.1 | Ulceration of intestine | Digestive | 1.00 | 0.93 | 1.08 | 0.93 | 259087 | 691 | 258396 | FALSE |
| 327.3 | Sleep apnea | Neurological | 1.00 | 0.97 | 1.03 | 0.94 | 327419 | 4699 | 322720 | FALSE |
| 441 | Vascular insufficiency of intestine | Circulatory System | 1.00 | 0.89 | 1.12 | 0.94 | 319657 | 297 | 319360 | FALSE |
| 619.2 | Disorders of uterus, NEC | Genitourinary | 1.00 | 0.97 | 1.04 | 0.94 | 281939 | 3391 | 278548 | FALSE |
| 153.3 | Malignant neoplasm of rectum, rectosigmoid junction, and anus | Neoplasms | 1.00 | 0.96 | 1.05 | 0.94 | 303397 | 2117 | 301280 | FALSE |
| 830 | Dislocation | Injuries & Poisonings | 1.00 | 0.96 | 1.05 | 0.94 | 323927 | 2154 | 321773 | FALSE |
| 478 | Throat pain | Respiratory | 1.00 | 0.91 | 1.11 | 0.94 | 309337 | 387 | 308950 | FALSE |
| 836 | Traumatic arthropathy | Injuries & Poisonings | 1.00 | 0.88 | 1.13 | 0.94 | 322016 | 243 | 321773 | FALSE |
| 269 | Proteinuria | Endocrine/Metabolic | 1.00 | 0.90 | 1.11 | 0.95 | 327850 | 355 | 327495 | FALSE |
| 443.9 | Peripheral vascular disease, unspecified | Circulatory System | 1.00 | 0.96 | 1.04 | 0.95 | 321969 | 2609 | 319360 | FALSE |
| 153.2 | Colon cancer | Neoplasms | 1.00 | 0.97 | 1.04 | 0.95 | 304402 | 3122 | 301280 | FALSE |
| 560.1 | Paralytic ileus | Digestive | 1.00 | 0.92 | 1.09 | 0.95 | 258916 | 520 | 258396 | FALSE |
| 618.5 | Prolapse of vaginal vault after hysterectomy | Genitourinary | 1.00 | 0.92 | 1.10 | 0.95 | 316743 | 501 | 316242 | FALSE |
| 624.1 | Dystrophy of female genital tract | Genitourinary | 1.00 | 0.88 | 1.14 | 0.95 | 318909 | 240 | 318669 | FALSE |
| 735.23 | Hallux rigidus | Musculoskeletal | 1.00 | 0.95 | 1.05 | 0.95 | 318243 | 1556 | 316687 | FALSE |
| 635.3 | Placenta previa and abruptio placenta | Pregnancy Complications | 1.00 | 0.95 | 1.06 | 0.95 | 321301 | 1336 | 319965 | FALSE |
| 610.2 | Fibroadenosis of breast | Genitourinary | 1.00 | 0.90 | 1.12 | 0.95 | 321189 | 316 | 320873 | FALSE |
| 555.1 | Regional enteritis | Digestive | 1.00 | 0.95 | 1.05 | 0.95 | 260201 | 1805 | 258396 | FALSE |
| 527.2 | Sialoadenitis | Digestive | 1.00 | 0.91 | 1.11 | 0.96 | 322908 | 374 | 322534 | FALSE |
| 791 | Gangrene | Symptoms | 1.00 | 0.92 | 1.09 | 0.96 | 328240 | 579 | 327661 | FALSE |
| 714 | Rheumatoid arthritis and other inflammatory polyarthropathies | Musculoskeletal | 1.00 | 0.98 | 1.02 | 0.96 | 325905 | 8652 | 317253 | FALSE |
| 446.5 | Giant cell arteritis | Circulatory System | 1.00 | 0.90 | 1.10 | 0.96 | 319750 | 390 | 319360 | FALSE |
| 218.1 | Uterine leiomyoma | Neoplasms | 1.00 | 0.98 | 1.02 | 0.96 | 318346 | 10566 | 307780 | FALSE |

| | | | | | | | | | | | | |
|---|---|---|---|---|---|---|---|---|---|---|---|---|
| 172.3 | Carcinoma in situ of skin | Neoplasms | 1.00 | 0.93 | 1.08 | 0.96 | 314884 | 664 | 314220 | FALSE |
| 531.2 | Gastric ulcer | Digestive | 1.00 | 0.97 | 1.03 | 0.96 | 324777 | 4228 | 320549 | FALSE |
| 229 | Benign neoplasm of unspecified sites | Neoplasms | 1.00 | 0.99 | 1.01 | 0.96 | 328240 | 46165 | 282075 | FALSE |
| 965.3 | Salicylates causing adverse effects in therapeutic use | Injuries & Poisonings | 1.00 | 0.91 | 1.11 | 0.96 | 300612 | 401 | 300211 | FALSE |
| 727.1 | Synovitis and tenosynovitis | Musculoskeletal | 1.00 | 0.93 | 1.08 | 0.96 | 305641 | 687 | 304954 | FALSE |
| 520 | Disorders of tooth development | Digestive | 1.00 | 0.91 | 1.10 | 0.96 | 312128 | 470 | 311658 | FALSE |
| 610.4 | Benign neoplasm of breast | Genitourinary | 1.00 | 0.95 | 1.05 | 0.97 | 322359 | 1486 | 320873 | FALSE |
| 580.32 | Nephritis and nephropathy with pathological lesion | Genitourinary | 1.00 | 0.94 | 1.06 | 0.97 | 315157 | 1078 | 314079 | FALSE |
| 907 | Injuries to the nervous system | Injuries & Poisonings | 1.00 | 0.96 | 1.05 | 0.97 | 328240 | 1845 | 326395 | FALSE |
| 782.3 | Edema | Symptoms | 1.00 | 0.95 | 1.05 | 0.97 | 328067 | 1715 | 326352 | FALSE |
| 375 | Disorders of lacrimal system | Sense Organs | 1.00 | 0.95 | 1.05 | 0.97 | 289852 | 1660 | 288192 | FALSE |
| 634 | Miscarriage; stillbirth | Pregnancy Complications | 1.00 | 0.97 | 1.03 | 0.97 | 324534 | 4569 | 319965 | FALSE |
| 474 | Acute and chronic tonsillitis | Respiratory | 1.00 | 0.92 | 1.08 | 0.97 | 309554 | 604 | 308950 | FALSE |
| 240 | Simple and unspecified goiter | Endocrine/Metabolic | 1.00 | 0.93 | 1.08 | 0.97 | 310951 | 622 | 310329 | FALSE |
| 202.24 | Large cell lymphoma | Neoplasms | 1.00 | 0.92 | 1.09 | 0.97 | 324112 | 589 | 323523 | FALSE |
| 592.12 | Chronic cystitis | Genitourinary | 1.00 | 0.94 | 1.07 | 0.97 | 297948 | 913 | 297035 | FALSE |
| 574.1 | Cholelithiasis | Digestive | 1.00 | 0.98 | 1.02 | 0.98 | 319629 | 9306 | 310323 | FALSE |
| 198.6 | Secondary malignancy of bone | Neoplasms | 1.00 | 0.96 | 1.04 | 0.98 | 231505 | 2193 | 229312 | FALSE |
| 599 | Other symptoms/disorders or the urinary system | Genitourinary | 1.00 | 0.99 | 1.01 | 0.98 | 324256 | 103829 | 220427 | FALSE |
| 580.2 | Nephrotic syndrome without mention of glomerulonephritis | Genitourinary | 1.00 | 0.92 | 1.09 | 0.98 | 314575 | 496 | 314079 | FALSE |
| 619 | Noninflammatory female genital disorders | Genitourinary | 1.00 | 0.99 | 1.01 | 0.98 | 328106 | 49558 | 278548 | FALSE |
| 220 | Benign neoplasm of ovary | Neoplasms | 1.00 | 0.95 | 1.05 | 0.98 | 297546 | 1490 | 296056 | FALSE |
| 721 | Spondylosis and allied disorders | Musculoskeletal | 1.00 | 0.94 | 1.07 | 0.98 | 311869 | 925 | 310944 | FALSE |
| 726 | Peripheral enthesopathies and allied syndromes | Musculoskeletal | 1.00 | 0.98 | 1.02 | 0.98 | 313722 | 8768 | 304954 | FALSE |
| 579.8 | Nonspecific abnormal findings in stool contents | Digestive | 1.00 | 0.95 | 1.05 | 0.98 | 305134 | 1740 | 303394 | FALSE |
| 199 | Neoplasm of uncertain behavior | Neoplasms | 1.00 | 0.94 | 1.06 | 0.99 | 230468 | 1156 | 229312 | FALSE |
| 443.1 | Raynaud's syndrome | Circulatory System | 1.00 | 0.94 | 1.06 | 0.99 | 320525 | 1165 | 319360 | FALSE |
| 579 | Other symptoms involving abdomen and pelvis | Digestive | 1.00 | 0.94 | 1.06 | 0.99 | 304580 | 1186 | 303394 | FALSE |
| 149 | Cancer of larynx, pharynx, nasal cavities | Neoplasms | 1.00 | 0.94 | 1.07 | 0.99 | 326986 | 945 | 326041 | FALSE |
| 292.1 | Aphasia/speech disturbance | Mental Disorders | 1.00 | 0.95 | 1.05 | 0.99 | 323019 | 1549 | 321470 | FALSE |
| 575.6 | Cholesterolosis of gallbladder | Digestive | 1.00 | 0.91 | 1.10 | 0.99 | 310789 | 466 | 310323 | FALSE |
| 512.9 | Other dyspnea | Respiratory | 1.00 | 0.94 | 1.07 | 0.99 | 315211 | 943 | 314268 | FALSE |
| 280.1 | Iron deficiency anemias, unspecified or not due to blood loss | Hematopoietic | 1.00 | 0.98 | 1.02 | 0.99 | 316402 | 7502 | 308900 | FALSE |
| 729.1 | Rheumatism, unspecified and fibrositis | Musculoskeletal | 1.00 | 0.90 | 1.11 | 0.99 | 305341 | 387 | 304954 | FALSE |
| 338.2 | Chronic pain | Neurological | 1.00 | 0.91 | 1.10 | 1.00 | 327830 | 404 | 327426 | FALSE |
| 242.1 | Graves' disease | Endocrine/Metabolic | 1.00 | 0.91 | 1.10 | 1.00 | 310789 | 460 | 310329 | FALSE |
| 345.12 | Partial epilepsy | Neurological | 1.00 | 0.89 | 1.12 | 1.00 | 286450 | 298 | 286152 | FALSE |

| Supplementary Table 12. Phenome-wide association study (PheWAS) results for the main calcium channel blocker (CCB) genetic risk score. | | | | | | | | | | |
|---|---|---|---|---|---|---|---|---|---|---|
| Phecode | Trait | Category | OR | Low 95% CI | Upp 95% CI | P value | Total sample size | Cases | Controls | FDR |
| 401.1 | Essential hypertension | Circulatory System | 0.95 | 0.94 | 0.95 | 1.49E-37 | 327983 | 79235 | 248748 | TRUE |
| 401 | Hypertension | Circulatory System | 0.95 | 0.94 | 0.96 | 3.01E-37 | 328239 | 79491 | 248748 | TRUE |
| 459.9 | Circulatory disease NEC | Circulatory System | 0.97 | 0.96 | 0.98 | 1.34E-15 | 327928 | 133749 | 194179 | TRUE |
| 411.4 | Coronary atherosclerosis | Circulatory System | 0.97 | 0.96 | 0.99 | 2.64E-05 | 327242 | 31553 | 295689 | TRUE |
| 411 | Ischemic Heart Disease | Circulatory System | 0.97 | 0.96 | 0.99 | 2.89E-05 | 327032 | 31343 | 295689 | TRUE |
| 411.8 | Other chronic ischemic heart disease, unspecified | Circulatory System | 0.97 | 0.96 | 0.99 | 3.07E-05 | 326969 | 31280 | 295689 | TRUE |
| 427.2 | Atrial fibrillation and flutter | Circulatory System | 0.97 | 0.95 | 0.98 | 1.18E-04 | 314573 | 15052 | 299521 | TRUE |
| 411.2 | Myocardial infarction | Circulatory System | 0.97 | 0.95 | 0.98 | 2.35E-04 | 307658 | 11969 | 295689 | TRUE |
| 562.1 | Diverticulosis | Digestive | 1.02 | 1.01 | 1.04 | 2.39E-04 | 286281 | 27885 | 258396 | TRUE |
| 411.41 | Aneurysm and dissection of heart | Circulatory System | 0.87 | 0.81 | 0.94 | 3.07E-04 | 296410 | 721 | 295689 | TRUE |
| 411.3 | Angina pectoris | Circulatory System | 0.97 | 0.96 | 0.99 | 5.44E-04 | 312048 | 16359 | 295689 | TRUE |
| 716.2 | Unspecified monoarthritis | Musculoskeletal | 1.03 | 1.01 | 1.04 | 2.30E-03 | 289797 | 15901 | 273896 | FALSE |
| 416 | Cardiomegaly | Circulatory System | 0.94 | 0.91 | 0.98 | 3.50E-03 | 324157 | 2610 | 321547 | FALSE |
| 428.1 | Congestive heart failure (CHF) NOS | Circulatory System | 0.94 | 0.90 | 0.98 | 0.01 | 324086 | 2053 | 322033 | FALSE |
| 38.2 | Gram positive septicemia | Infectious Diseases | 0.89 | 0.81 | 0.97 | 0.01 | 313123 | 491 | 312632 | FALSE |
| 946 | Anaphylactic shock NOS | Injuries & Poisonings | 0.89 | 0.82 | 0.97 | 0.01 | 322450 | 544 | 321906 | FALSE |
| 395.1 | Nonrheumatic mitral valve disorders | Circulatory System | 0.95 | 0.92 | 0.99 | 0.01 | 324497 | 2969 | 321528 | FALSE |
| 977 | Personal history of allergy to medicinal agents | Injuries & Poisonings | 0.85 | 0.74 | 0.96 | 0.01 | 300449 | 238 | 300211 | FALSE |
| 476 | Allergic rhinitis | Respiratory | 1.08 | 1.02 | 1.15 | 0.01 | 310025 | 1075 | 308950 | FALSE |
| 428.2 | Heart failure NOS | Circulatory System | 0.96 | 0.93 | 0.99 | 0.01 | 326367 | 4334 | 322033 | FALSE |
| 751.11 | Congenital anomalies of female genital organs | Congenital Anomalies | 0.89 | 0.81 | 0.97 | 0.01 | 326445 | 442 | 326003 | FALSE |
| 240 | Simple and unspecified goiter | Endocrine/Metabolic | 0.90 | 0.83 | 0.98 | 0.01 | 310951 | 622 | 310329 | FALSE |
| 352.2 | Facial nerve disorders [CN7] | Neurological | 0.92 | 0.86 | 0.98 | 0.01 | 313911 | 900 | 313011 | FALSE |
| 333.1 | Essential tremor | Neurological | 0.84 | 0.74 | 0.96 | 0.01 | 286370 | 218 | 286152 | FALSE |
| 200.1 | Polycythemia vera | Neoplasms | 0.88 | 0.80 | 0.97 | 0.01 | 318136 | 404 | 317732 | FALSE |
| 634.1 | Missed abortion/Hydatidiform mole | Pregnancy Complications | 1.07 | 1.01 | 1.14 | 0.01 | 321168 | 1203 | 319965 | FALSE |
| 686.3 | Pilonidal cyst | Dermatologic | 1.10 | 1.02 | 1.19 | 0.01 | 317024 | 617 | 316407 | FALSE |
| 503 | Pulmonary congestion and hypostasis | Respiratory | 0.89 | 0.82 | 0.98 | 0.02 | 316959 | 475 | 316484 | FALSE |
| 555 | Inflammatory bowel disease and other gastroenteritis and colitis | Digestive | 1.02 | 1.00 | 1.03 | 0.02 | 278195 | 19799 | 258396 | FALSE |
| 741.4 | Joint effusions | Musculoskeletal | 0.87 | 0.77 | 0.98 | 0.02 | 310925 | 283 | 310642 | FALSE |
| 220 | Benign neoplasm of ovary | Neoplasms | 1.06 | 1.01 | 1.12 | 0.02 | 297546 | 1490 | 296056 | FALSE |
| 558 | Noninfectious gastroenteritis | Digestive | 1.02 | 1.00 | 1.03 | 0.02 | 278260 | 19864 | 258396 | FALSE |
| 513.4 | Hyperventilation | Respiratory | 1.14 | 1.02 | 1.28 | 0.03 | 327904 | 280 | 327624 | FALSE |
| 574 | Cholelithiasis and cholecystitis | Digestive | 0.90 | 0.81 | 0.99 | 0.03 | 310726 | 403 | 310323 | FALSE |
| 394.2 | Mitral valve disease | Circulatory System | 0.96 | 0.93 | 1.00 | 0.03 | 324603 | 3075 | 321528 | FALSE |
| 381.11 | Suppurative and unspecified otitis media | Sense Organs | 0.93 | 0.87 | 0.99 | 0.03 | 324993 | 850 | 324143 | FALSE |

| | | | | | | | | | | | |
|---|---|---|---|---|---|---|---|---|---|---|---|
| 411.9 | Other acute and subacute forms of ischemic heart disease | Circulatory System | 0.94 | 0.89 | 0.99 | 0.03 | 296883 | 1194 | 295689 | FALSE |
| 375 | Disorders of lacrimal system | Sense Organs | 1.05 | 1.00 | 1.11 | 0.03 | 289852 | 1660 | 288192 | FALSE |
| 574.12 | Cholelithiasis with other cholecystitis | Digestive | 0.97 | 0.95 | 1.00 | 0.03 | 315816 | 5493 | 310323 | FALSE |
| 335 | Multiple sclerosis | Neurological | 1.06 | 1.00 | 1.12 | 0.03 | 287531 | 1379 | 286152 | FALSE |
| 850 | Hemorrhage or hematoma complicating a procedure | Injuries & Poisonings | 0.97 | 0.95 | 1.00 | 0.03 | 319328 | 5364 | 313964 | FALSE |
| 378.5 | Paralytic strabismus | Sense Organs | 0.88 | 0.79 | 0.99 | 0.04 | 288479 | 287 | 288192 | FALSE |
| 594.3 | Calculus of ureter | Genitourinary | 0.96 | 0.92 | 1.00 | 0.04 | 322471 | 2540 | 319931 | FALSE |
| 960 | Poisoning by antibiotics | Injuries & Poisonings | 0.97 | 0.93 | 1.00 | 0.04 | 303739 | 3528 | 300211 | FALSE |
| 560.4 | Other intestinal obstruction | Digestive | 1.04 | 1.00 | 1.07 | 0.04 | 261839 | 3443 | 258396 | FALSE |
| 170.2 | Cancer of connective tissue | Neoplasms | 0.93 | 0.86 | 1.00 | 0.04 | 328122 | 734 | 327388 | FALSE |
| 574.3 | Cholecystitis without cholelithiasis | Digestive | 1.04 | 1.00 | 1.08 | 0.04 | 313141 | 2818 | 310323 | FALSE |
| 426.24 | Atrioventricular block, complete | Circulatory System | 0.92 | 0.85 | 1.00 | 0.04 | 300112 | 591 | 299521 | FALSE |
| 601.11 | Acute prostatitis | Genitourinary | 1.13 | 1.00 | 1.28 | 0.04 | 307653 | 260 | 307393 | FALSE |
| 564.9 | Personal history of diseases of digestive system | Digestive | 1.02 | 1.00 | 1.03 | 0.04 | 274320 | 15924 | 258396 | FALSE |
| 380.4 | Impacted cerumen | Sense Organs | 0.90 | 0.81 | 1.00 | 0.05 | 327106 | 354 | 326752 | FALSE |
| 989 | Toxic effect of other substances, chiefly nonmedicinal as to source | Injuries & Poisonings | 1.05 | 1.00 | 1.11 | 0.05 | 328136 | 1438 | 326698 | FALSE |
| 750 | Digestive congenital anomalies | Congenital Anomalies | 0.93 | 0.86 | 1.00 | 0.05 | 326705 | 702 | 326003 | FALSE |
| 751.1 | Congenital anomalies of genital organs | Congenital Anomalies | 0.93 | 0.86 | 1.00 | 0.05 | 326709 | 706 | 326003 | FALSE |
| 215 | Other benign neoplasm of connective and other soft tissue | Neoplasms | 1.06 | 1.00 | 1.12 | 0.05 | 321804 | 1128 | 320676 | FALSE |
| 388 | Other disorders of ear | Sense Organs | 0.98 | 0.96 | 1.00 | 0.05 | 328080 | 9886 | 318194 | FALSE |
| 735 | Acquired foot deformities | Musculoskeletal | 1.12 | 1.00 | 1.25 | 0.05 | 316986 | 299 | 316687 | FALSE |
| 530.12 | Ulcer of esophagus | Digestive | 0.97 | 0.95 | 1.00 | 0.06 | 293151 | 5348 | 287803 | FALSE |
| 452 | Other venous embolism and thrombosis | Circulatory System | 0.92 | 0.85 | 1.00 | 0.06 | 288267 | 554 | 287713 | FALSE |
| 304 | Adjustment reaction | Mental Disorders | 1.11 | 1.00 | 1.24 | 0.06 | 283221 | 323 | 282898 | FALSE |
| 442.8 | Aneurysm of other specified artery | Circulatory System | 0.87 | 0.76 | 1.00 | 0.06 | 319570 | 210 | 319360 | FALSE |
| 478 | Throat pain | Respiratory | 0.91 | 0.82 | 1.00 | 0.06 | 309337 | 387 | 308950 | FALSE |
| 560.3 | Peritoneal or intestinal adhesions | Digestive | 1.07 | 1.00 | 1.14 | 0.06 | 259257 | 861 | 258396 | FALSE |
| 414 | Other forms of chronic heart disease | Circulatory System | 0.96 | 0.91 | 1.00 | 0.06 | 297457 | 1768 | 295689 | FALSE |
| 415.21 | Primary pulmonary hypertension | Circulatory System | 1.09 | 1.00 | 1.19 | 0.06 | 322013 | 466 | 321547 | FALSE |
| 745 | Pain in joint | Musculoskeletal | 0.96 | 0.92 | 1.00 | 0.06 | 328240 | 1940 | 326300 | FALSE |
| 365 | Glaucoma | Sense Organs | 1.03 | 1.00 | 1.06 | 0.06 | 321340 | 4558 | 316782 | FALSE |
| 800.1 | Fracture of neck of femur | Injuries & Poisonings | 0.88 | 0.78 | 1.01 | 0.06 | 323796 | 239 | 323557 | FALSE |
| 429.3 | Symptoms involving cardiovascular system | Circulatory System | 0.88 | 0.76 | 1.01 | 0.06 | 322241 | 208 | 322033 | FALSE |
| 286.7 | Other and unspecified coagulation defects | Hematopoietic | 1.10 | 1.00 | 1.21 | 0.06 | 325902 | 405 | 325497 | FALSE |
| 716.9 | Arthropathy NOS | Musculoskeletal | 1.01 | 1.00 | 1.02 | 0.06 | 327719 | 53823 | 273896 | FALSE |
| 736.2 | Acquired deformities of finger | Musculoskeletal | 0.89 | 0.78 | 1.01 | 0.06 | 316940 | 253 | 316687 | FALSE |
| 380 | Disorders of external ear | Sense Organs | 0.95 | 0.90 | 1.00 | 0.06 | 328227 | 1475 | 326752 | FALSE |
| 341 | Other demyelinating diseases of central nervous system | Neurological | 1.05 | 1.00 | 1.10 | 0.06 | 287764 | 1612 | 286152 | FALSE |

| | | | | | | | | | | |
|---|---|---|---|---|---|---|---|---|---|---|
| 70.9 | Hepatitis NOS | Infectious Diseases | 1.09 | 0.99 | 1.20 | 0.06 | 322658 | 452 | 322206 | FALSE |
| 250.41 | Impaired fasting glucose | Endocrine/Metabolic | 1.12 | 0.99 | 1.26 | 0.07 | 307758 | 268 | 307490 | FALSE |
| 681 | Superficial cellulitis and abscess | Dermatologic | 0.93 | 0.87 | 1.00 | 0.07 | 317135 | 728 | 316407 | FALSE |
| 360.2 | Progressive myopia | Sense Organs | 1.12 | 0.99 | 1.27 | 0.07 | 317032 | 250 | 316782 | FALSE |
| 272.1 | Hyperlipidemia | Endocrine/Metabolic | 0.97 | 0.95 | 1.00 | 0.07 | 296728 | 5231 | 291497 | FALSE |
| 277 | Other disorders of metabolism | Endocrine/Metabolic | 0.99 | 0.98 | 1.00 | 0.07 | 328230 | 45303 | 282927 | FALSE |
| 250 | Diabetes mellitus | Endocrine/Metabolic | 1.01 | 1.00 | 1.03 | 0.07 | 327730 | 20240 | 307490 | FALSE |
| 250.2 | Type 2 diabetes | Endocrine/Metabolic | 1.01 | 1.00 | 1.03 | 0.07 | 326603 | 19113 | 307490 | FALSE |
| 180.1 | Cervical cancer | Neoplasms | 0.97 | 0.95 | 1.00 | 0.08 | 302137 | 4554 | 297583 | FALSE |
| 375.2 | Epiphora | Sense Organs | 1.06 | 0.99 | 1.13 | 0.08 | 289094 | 902 | 288192 | FALSE |
| 350.2 | Abnormality of gait | Neurological | 0.96 | 0.91 | 1.00 | 0.08 | 327077 | 1633 | 325444 | FALSE |
| 507 | Pleurisy; pleural effusion | Respiratory | 0.98 | 0.95 | 1.00 | 0.08 | 323091 | 6607 | 316484 | FALSE |
| 709.7 | Unspecified diffuse connective tissue disease | Dermatologic | 1.01 | 1.00 | 1.02 | 0.08 | 324635 | 82669 | 241966 | FALSE |
| 368.1 | Amblyopia | Sense Organs | 0.93 | 0.85 | 1.01 | 0.08 | 325423 | 541 | 324882 | FALSE |
| 907 | Injuries to the nervous system | Injuries & Poisonings | 0.96 | 0.92 | 1.01 | 0.08 | 328240 | 1845 | 326395 | FALSE |
| 250.42 | Other abnormal glucose | Endocrine/Metabolic | 0.92 | 0.84 | 1.01 | 0.08 | 307927 | 437 | 307490 | FALSE |
| 510 | Other diseases of lung | Respiratory | 0.94 | 0.87 | 1.01 | 0.08 | 328200 | 770 | 327430 | FALSE |
| 726 | Peripheral enthesopathies and allied syndromes | Musculoskeletal | 1.02 | 1.00 | 1.04 | 0.08 | 313722 | 8768 | 304954 | FALSE |
| 334 | Degenerative disease of the spinal cord | Neurological | 1.05 | 0.99 | 1.10 | 0.08 | 287631 | 1479 | 286152 | FALSE |
| 244.1 | Secondary hypothyroidism | Endocrine/Metabolic | 0.95 | 0.90 | 1.01 | 0.08 | 311484 | 1155 | 310329 | FALSE |
| 292.2 | Mild cognitive impairment | Mental Disorders | 1.13 | 0.98 | 1.29 | 0.09 | 321672 | 202 | 321470 | FALSE |
| 528.6 | Leukoplakia of oral mucosa | Digestive | 0.91 | 0.81 | 1.02 | 0.09 | 322846 | 312 | 322534 | FALSE |
| 202.24 | Large cell lymphoma | Neoplasms | 1.07 | 0.99 | 1.16 | 0.09 | 324112 | 589 | 323523 | FALSE |
| 857 | Mechanical complication of unspecified genitourinary device, implant, and graft | Injuries & Poisonings | 0.95 | 0.90 | 1.01 | 0.09 | 315248 | 1284 | 313964 | FALSE |
| 379.2 | Disorders of vitreous body | Sense Organs | 1.05 | 0.99 | 1.10 | 0.09 | 289599 | 1407 | 288192 | FALSE |
| 151 | Cancer of stomach | Neoplasms | 1.07 | 0.99 | 1.16 | 0.09 | 312905 | 574 | 312331 | FALSE |
| 425.1 | Primary/intrinsic cardiomyopathies | Circulatory System | 0.95 | 0.89 | 1.01 | 0.09 | 326036 | 1053 | 324983 | FALSE |
| 345.12 | Partial epilepsy | Neurological | 0.91 | 0.81 | 1.02 | 0.09 | 286450 | 298 | 286152 | FALSE |
| 371 | Inflammation of the eye | Sense Organs | 0.92 | 0.83 | 1.01 | 0.09 | 318796 | 408 | 318388 | FALSE |
| 480.11 | Pneumococcal pneumonia | Respiratory | 1.02 | 1.00 | 1.05 | 0.10 | 323680 | 5991 | 317689 | FALSE |
| 798 | Malaise and fatigue | Symptoms | 1.03 | 0.99 | 1.07 | 0.10 | 327695 | 2966 | 324729 | FALSE |
| 157 | Pancreatic cancer | Neoplasms | 1.07 | 0.99 | 1.16 | 0.10 | 312933 | 602 | 312331 | FALSE |
| 389.2 | Conductive hearing loss | Sense Organs | 0.92 | 0.84 | 1.01 | 0.10 | 318629 | 435 | 318194 | FALSE |
| 593 | Hematuria | Genitourinary | 1.01 | 1.00 | 1.03 | 0.10 | 313795 | 16760 | 297035 | FALSE |
| 961.1 | Poisoning/allergy of sulfonamides | Injuries & Poisonings | 0.95 | 0.89 | 1.01 | 0.10 | 301101 | 890 | 300211 | FALSE |
| 642.1 | Preeclampsia and eclampsia | Pregnancy Complications | 0.91 | 0.82 | 1.02 | 0.10 | 327385 | 335 | 327050 | FALSE |
| 324 | Other CNS infection and poliomyelitis | Neurological | 1.10 | 0.98 | 1.23 | 0.10 | 327426 | 288 | 327138 | FALSE |
| 601.12 | Chronic prostatitis | Genitourinary | 1.05 | 0.99 | 1.12 | 0.11 | 308341 | 948 | 307393 | FALSE |

| | | | | | | | | | | |
|---|---|---|---|---|---|---|---|---|---|---|
| 149.4 | Cancer of larynx | Neoplasms | 0.90 | 0.80 | 1.02 | 0.11 | 326307 | 266 | 326041 | FALSE |
| 601.1 | Prostatitis | Genitourinary | 1.06 | 0.99 | 1.15 | 0.11 | 308058 | 665 | 307393 | FALSE |
| 480.5 | Bronchopneumonia and lung abscess | Respiratory | 0.92 | 0.83 | 1.02 | 0.11 | 318077 | 388 | 317689 | FALSE |
| 433.12 | Cerebral atherosclerosis | Circulatory System | 1.11 | 0.98 | 1.26 | 0.11 | 318345 | 238 | 318107 | FALSE |
| 700 | Corns and callosities | Dermatologic | 0.91 | 0.81 | 1.02 | 0.11 | 323352 | 288 | 323064 | FALSE |
| 525 | Other diseases of the teeth and supporting structures | Digestive | 1.03 | 0.99 | 1.07 | 0.11 | 314357 | 2699 | 311658 | FALSE |
| 458.9 | Hypotension NOS | Circulatory System | 0.97 | 0.94 | 1.01 | 0.11 | 197817 | 3638 | 194179 | FALSE |
| 184.11 | Malignant neoplasm of ovary | Neoplasms | 0.98 | 0.95 | 1.01 | 0.11 | 310562 | 5009 | 305553 | FALSE |
| 728.71 | Contracture of palmar fascia [Dupuytren's disease] | Musculoskeletal | 0.97 | 0.93 | 1.01 | 0.11 | 307403 | 2449 | 304954 | FALSE |
| 41 | Bacterial infection NOS | Infectious Diseases | 0.98 | 0.97 | 1.00 | 0.11 | 323384 | 10752 | 312632 | FALSE |
| 627.4 | Premenopausal menorrhagia | Genitourinary | 0.92 | 0.82 | 1.02 | 0.11 | 296563 | 332 | 296231 | FALSE |
| 187.2 | Malignant neoplasm of testis | Neoplasms | 0.98 | 0.95 | 1.01 | 0.11 | 323790 | 4572 | 319218 | FALSE |
| 636 | Early or threatened labor; hemorrhage in early pregnancy | Pregnancy Complications | 1.05 | 0.99 | 1.11 | 0.12 | 321227 | 1262 | 319965 | FALSE |
| 371.1 | Uveitis, noninfectious or NOS | Sense Organs | 1.07 | 0.98 | 1.17 | 0.12 | 318886 | 498 | 318388 | FALSE |
| 198.6 | Secondary malignancy of bone | Neoplasms | 1.03 | 0.99 | 1.08 | 0.12 | 231505 | 2193 | 229312 | FALSE |
| 451.2 | Phlebitis and thrombophlebitis of lower extremities | Circulatory System | 1.03 | 0.99 | 1.06 | 0.12 | 291357 | 3644 | 287713 | FALSE |
| 722.1 | Displacement of intervertebral disc | Musculoskeletal | 1.07 | 0.98 | 1.17 | 0.12 | 311460 | 516 | 310944 | FALSE |
| 473 | Diseases of the larynx and vocal cords | Respiratory | 0.96 | 0.92 | 1.01 | 0.12 | 310564 | 1614 | 308950 | FALSE |
| 386.9 | Dizziness and giddiness (Light-headedness and vertigo) | Sense Organs | 0.98 | 0.95 | 1.01 | 0.12 | 326388 | 4739 | 321649 | FALSE |
| 916 | Contusion | Injuries & Poisonings | 0.96 | 0.91 | 1.01 | 0.12 | 328240 | 1478 | 326762 | FALSE |
| 395.6 | Heart valve replaced | Circulatory System | 0.96 | 0.91 | 1.01 | 0.12 | 323056 | 1528 | 321528 | FALSE |
| 418.1 | Precordial pain | Circulatory System | 0.97 | 0.94 | 1.01 | 0.13 | 299506 | 3686 | 295820 | FALSE |
| 280.2 | Iron deficiency anemia secondary to blood loss (chronic) | Hematopoietic | 1.07 | 0.98 | 1.17 | 0.13 | 309376 | 476 | 308900 | FALSE |
| 722.6 | Degeneration of intervertebral disc | Musculoskeletal | 1.03 | 0.99 | 1.07 | 0.13 | 313866 | 2922 | 310944 | FALSE |
| 374 | Other disorders of eyelids | Sense Organs | 0.97 | 0.94 | 1.01 | 0.13 | 321593 | 3205 | 318388 | FALSE |
| 426.31 | Right bundle branch block | Circulatory System | 0.96 | 0.92 | 1.01 | 0.13 | 301094 | 1573 | 299521 | FALSE |
| 389 | Hearing loss | Sense Organs | 0.97 | 0.94 | 1.01 | 0.13 | 321364 | 3170 | 318194 | FALSE |
| 276.14 | Hypopotassemia | Endocrine/Metabolic | 0.96 | 0.91 | 1.01 | 0.13 | 322115 | 1471 | 320644 | FALSE |
| 614.54 | Abscess or ulceration of vulva | Genitourinary | 1.09 | 0.97 | 1.23 | 0.13 | 319079 | 280 | 318799 | FALSE |
| 333 | Extrapyramidal disease and abnormal movement disorders | Neurological | 0.97 | 0.93 | 1.01 | 0.13 | 288187 | 2035 | 286152 | FALSE |
| 722.9 | Other and unspecified disc disorder | Musculoskeletal | 1.02 | 0.99 | 1.06 | 0.13 | 314894 | 3950 | 310944 | FALSE |
| 378.1 | Strabismus (not specified as paralytic) | Sense Organs | 1.05 | 0.99 | 1.12 | 0.13 | 289139 | 947 | 288192 | FALSE |
| 477 | Epistaxis or throat hemorrhage | Respiratory | 0.97 | 0.93 | 1.01 | 0.14 | 311469 | 2519 | 308950 | FALSE |
| 702.2 | Seborrheic keratosis | Dermatologic | 1.03 | 0.99 | 1.06 | 0.14 | 325769 | 3169 | 322600 | FALSE |
| 174.11 | Malignant neoplasm of female breast | Neoplasms | 0.99 | 0.97 | 1.00 | 0.14 | 316838 | 12414 | 304424 | FALSE |
| 250.24 | Type 2 diabetes with neurological manifestations | Endocrine/Metabolic | 0.94 | 0.87 | 1.02 | 0.14 | 308102 | 612 | 307490 | FALSE |
| 530.2 | Esophageal bleeding (varices/hemorrhage) | Digestive | 1.04 | 0.99 | 1.09 | 0.14 | 289525 | 1722 | 287803 | FALSE |
| 962.3 | Hormones and synthetic substitutes causing adverse effects in therapeutic use | Injuries & Poisonings | 1.08 | 0.98 | 1.20 | 0.14 | 300575 | 364 | 300211 | FALSE |

| | | | | | | | | | | |
|---|---|---|---|---|---|---|---|---|---|---|
| 578.8 | Hemorrhage of rectum and anus | Digestive | 0.99 | 0.97 | 1.00 | 0.14 | 317220 | 13826 | 303394 | FALSE |
| 509.8 | Dependence on respirator [Ventilator] or supplemental oxygen | Respiratory | 1.08 | 0.98 | 1.19 | 0.14 | 316885 | 401 | 316484 | FALSE |
| 465 | Acute upper respiratory infections of multiple or unspecified sites | Respiratory | 0.97 | 0.94 | 1.01 | 0.14 | 328232 | 3053 | 325179 | FALSE |
| 681.2 | Cellulitis and abscess of face/neck | Dermatologic | 0.94 | 0.86 | 1.02 | 0.14 | 316936 | 529 | 316407 | FALSE |
| 726.3 | Bursitis | Musculoskeletal | 1.06 | 0.98 | 1.16 | 0.14 | 305497 | 543 | 304954 | FALSE |
| 681.7 | Cellulitis and abscess of trunk | Dermatologic | 1.06 | 0.98 | 1.15 | 0.15 | 317030 | 623 | 316407 | FALSE |
| 444.1 | Arterial embolism and thrombosis of lower extremity artery | Circulatory System | 1.06 | 0.98 | 1.15 | 0.15 | 319930 | 570 | 319360 | FALSE |
| 202.21 | Nodular lymphoma | Neoplasms | 1.08 | 0.97 | 1.19 | 0.15 | 323913 | 390 | 323523 | FALSE |
| 619.3 | Noninflammatory disorders of cervix | Genitourinary | 0.97 | 0.94 | 1.01 | 0.15 | 281393 | 2845 | 278548 | FALSE |
| 703 | Diseases of nail, NOS | Dermatologic | 0.93 | 0.84 | 1.03 | 0.15 | 321795 | 366 | 321429 | FALSE |
| 528 | Diseases of the oral soft tissues, excluding lesions specific for gingiva and tongue | Digestive | 0.97 | 0.93 | 1.01 | 0.15 | 324593 | 2059 | 322534 | FALSE |
| 221 | Benign neoplasm of other female genital organs | Neoplasms | 0.91 | 0.79 | 1.04 | 0.15 | 295974 | 223 | 295751 | FALSE |
| 475 | Chronic sinusitis | Respiratory | 1.03 | 0.99 | 1.07 | 0.15 | 311580 | 2630 | 308950 | FALSE |
| 281.13 | Folate-deficiency anemia | Hematopoietic | 1.10 | 0.96 | 1.27 | 0.16 | 309101 | 201 | 308900 | FALSE |
| 441 | Vascular insufficiency of intestine | Circulatory System | 0.92 | 0.82 | 1.03 | 0.16 | 319657 | 297 | 319360 | FALSE |
| 642 | Hypertension complicating pregnancy, childbirth, and the puerperium | Pregnancy Complications | 0.95 | 0.90 | 1.02 | 0.16 | 328039 | 989 | 327050 | FALSE |
| 306 | Other mental disorder | Mental Disorders | 0.99 | 0.98 | 1.00 | 0.16 | 312303 | 29405 | 282898 | FALSE |
| 939 | Atopic/contact dermatitis due to other or unspecified | Dermatologic | 1.03 | 0.99 | 1.08 | 0.16 | 324060 | 2154 | 321906 | FALSE |
| 695.3 | Rosacea | Dermatologic | 0.92 | 0.83 | 1.03 | 0.16 | 322068 | 319 | 321749 | FALSE |
| 573.3 | Hepatomegaly | Digestive | 1.08 | 0.97 | 1.21 | 0.16 | 318641 | 310 | 318331 | FALSE |
| 191.11 | Cancer of brain | Neoplasms | 0.94 | 0.86 | 1.03 | 0.16 | 326945 | 498 | 326447 | FALSE |
| 578 | Gastrointestinal hemorrhage | Digestive | 1.09 | 0.97 | 1.22 | 0.16 | 303679 | 285 | 303394 | FALSE |
| 367.2 | Astigmatism | Sense Organs | 1.10 | 0.96 | 1.26 | 0.16 | 325964 | 210 | 325754 | FALSE |
| 727.5 | Rupture of synovium | Musculoskeletal | 1.08 | 0.97 | 1.20 | 0.17 | 305298 | 344 | 304954 | FALSE |
| 702.1 | Actinic keratosis | Dermatologic | 1.03 | 0.99 | 1.07 | 0.17 | 325264 | 2664 | 322600 | FALSE |
| 255.21 | Glucocorticoid deficiency | Endocrine/Metabolic | 1.07 | 0.97 | 1.19 | 0.17 | 324917 | 372 | 324545 | FALSE |
| 361 | Retinal detachments and defects | Sense Organs | 1.03 | 0.99 | 1.07 | 0.17 | 319011 | 2229 | 316782 | FALSE |
| 979 | Adverse drug events and drug allergies | Injuries & Poisonings | 0.95 | 0.88 | 1.02 | 0.17 | 300927 | 716 | 300211 | FALSE |
| 696.42 | Psoriatic arthropathy | Dermatologic | 1.05 | 0.98 | 1.13 | 0.17 | 314672 | 740 | 313932 | FALSE |
| 217.1 | Nevus, non-neoplastic | Neoplasms | 0.94 | 0.87 | 1.02 | 0.17 | 320346 | 597 | 319749 | FALSE |
| 8.6 | Viral Enteritis | Infectious Diseases | 0.95 | 0.89 | 1.02 | 0.18 | 319924 | 845 | 319079 | FALSE |
| 433.3 | Cerebral ischemia | Circulatory System | 0.96 | 0.90 | 1.02 | 0.18 | 319041 | 934 | 318107 | FALSE |
| 508 | Pulmonary collapse; interstitial and compensatory emphysema | Respiratory | 1.03 | 0.99 | 1.08 | 0.18 | 318570 | 2086 | 316484 | FALSE |
| 585.1 | Acute renal failure | Genitourinary | 0.98 | 0.95 | 1.01 | 0.18 | 318744 | 4665 | 314079 | FALSE |
| 8.5 | Bacterial enteritis | Infectious Diseases | 1.03 | 0.99 | 1.07 | 0.18 | 321188 | 2109 | 319079 | FALSE |
| 281.11 | Pernicious anemia | Hematopoietic | 0.95 | 0.89 | 1.02 | 0.18 | 309650 | 750 | 308900 | FALSE |
| 426.3 | Bundle branch block | Circulatory System | 1.08 | 0.96 | 1.21 | 0.18 | 299812 | 291 | 299521 | FALSE |
| 526.1 | Cysts of the jaws | Digestive | 1.08 | 0.97 | 1.20 | 0.18 | 311993 | 335 | 311658 | FALSE |

| | | | | | | | | | | |
|---|---|---|---|---|---|---|---|---|---|---|
| 278.1 | Obesity | Endocrine/Metabolic | 0.99 | 0.97 | 1.01 | 0.19 | 328110 | 11143 | 316967 | FALSE |
| 752 | Nervous system congenital anomalies | Congenital Anomalies | 0.92 | 0.82 | 1.04 | 0.19 | 327963 | 288 | 327675 | FALSE |
| 528.11 | Stomatitis and mucositis (ulcerative) | Digestive | 1.07 | 0.97 | 1.17 | 0.19 | 322945 | 411 | 322534 | FALSE |
| 969 | Poisoning by psychotropic agents | Injuries & Poisonings | 1.03 | 0.99 | 1.08 | 0.19 | 302207 | 1996 | 300211 | FALSE |
| 394 | Rheumatic disease of the heart valves | Circulatory System | 0.96 | 0.90 | 1.02 | 0.19 | 322471 | 943 | 321528 | FALSE |
| 226 | Benign neoplasm of thyroid glands | Neoplasms | 1.08 | 0.96 | 1.22 | 0.19 | 326908 | 266 | 326642 | FALSE |
| 211 | Benign neoplasm of other parts of digestive system | Neoplasms | 1.02 | 0.99 | 1.05 | 0.19 | 319683 | 5375 | 314308 | FALSE |
| 199 | Neoplasm of uncertain behavior | Neoplasms | 0.96 | 0.91 | 1.02 | 0.19 | 230468 | 1156 | 229312 | FALSE |
| 442.1 | Aortic aneurysm | Circulatory System | 0.95 | 0.87 | 1.03 | 0.19 | 319946 | 586 | 319360 | FALSE |
| 717 | Polymyalgia Rheumatica | Musculoskeletal | 1.04 | 0.98 | 1.10 | 0.19 | 328240 | 1147 | 327093 | FALSE |
| 374.1 | Ectropion or entropion | Sense Organs | 1.04 | 0.98 | 1.10 | 0.19 | 319480 | 1092 | 318388 | FALSE |
| 610.8 | Other specified benign mammary dysplasias | Genitourinary | 1.05 | 0.98 | 1.13 | 0.20 | 321576 | 703 | 320873 | FALSE |
| 474 | Acute and chronic tonsillitis | Respiratory | 1.05 | 0.97 | 1.14 | 0.20 | 309554 | 604 | 308950 | FALSE |
| 530.5 | Disorders of esophageal motility | Digestive | 1.05 | 0.97 | 1.14 | 0.20 | 288452 | 649 | 287803 | FALSE |
| 624.9 | stress incontinence, female | Genitourinary | 1.02 | 0.99 | 1.04 | 0.20 | 324769 | 6100 | 318669 | FALSE |
| 550.2 | Diaphragmatic hernia | Digestive | 1.01 | 1.00 | 1.02 | 0.20 | 307453 | 27664 | 279789 | FALSE |
| 859 | Complication due to other implant and internal device | Injuries & Poisonings | 0.98 | 0.95 | 1.01 | 0.20 | 317302 | 3338 | 313964 | FALSE |
| 473.3 | Paralysis/spasm of vocal cords or larynx | Respiratory | 0.92 | 0.82 | 1.04 | 0.20 | 309217 | 267 | 308950 | FALSE |
| 571.5 | Other chronic nonalcoholic liver disease | Digestive | 1.03 | 0.98 | 1.08 | 0.20 | 320004 | 1673 | 318331 | FALSE |
| 348.8 | Encephalopathy, not elsewhere classified | Neurological | 1.09 | 0.95 | 1.25 | 0.21 | 286356 | 204 | 286152 | FALSE |
| 689 | Disorder of skin and subcutaneous tissue NOS | Dermatologic | 1.01 | 1.00 | 1.02 | 0.21 | 328240 | 42194 | 286046 | FALSE |
| 426.23 | Second degree AV block | Circulatory System | 1.06 | 0.97 | 1.16 | 0.21 | 299982 | 461 | 299521 | FALSE |
| 647.1 | Infections of genitourinary tract during pregnancy | Pregnancy Complications | 1.08 | 0.96 | 1.21 | 0.21 | 328128 | 284 | 327844 | FALSE |
| 614.33 | Pelvic inflammatory disease, NOS | Genitourinary | 0.98 | 0.96 | 1.01 | 0.21 | 324944 | 6145 | 318799 | FALSE |
| 573 | Other disorders of liver | Digestive | 1.02 | 0.99 | 1.05 | 0.21 | 323533 | 5202 | 318331 | FALSE |
| 938.2 | Chronic dermatitis due to solar radiation | Injuries & Poisonings | 0.93 | 0.82 | 1.04 | 0.21 | 322170 | 264 | 321906 | FALSE |
| 316 | Substance addiction and disorders | Mental Disorders | 1.07 | 0.96 | 1.18 | 0.21 | 298042 | 373 | 297669 | FALSE |
| 858 | Complication of internal orthopedic device | Injuries & Poisonings | 0.98 | 0.94 | 1.01 | 0.21 | 317147 | 3183 | 313964 | FALSE |
| 290.2 | Delirium due to conditions classified elsewhere | Mental Disorders | 0.95 | 0.88 | 1.03 | 0.21 | 322151 | 681 | 321470 | FALSE |
| 276.5 | Hypovolemia | Endocrine/Metabolic | 1.02 | 0.99 | 1.06 | 0.21 | 323539 | 2895 | 320644 | FALSE |
| 704 | Diseases of hair and hair follicles | Dermatologic | 1.02 | 0.99 | 1.05 | 0.21 | 326692 | 5263 | 321429 | FALSE |
| 361.1 | Retinal detachment with retinal defect | Sense Organs | 0.97 | 0.92 | 1.02 | 0.22 | 318238 | 1456 | 316782 | FALSE |
| 195 | Cancer, suspected or other | Neoplasms | 1.04 | 0.98 | 1.11 | 0.22 | 230227 | 915 | 229312 | FALSE |
| 618.2 | Uterine/Uterovaginal prolapse | Genitourinary | 1.02 | 0.99 | 1.04 | 0.22 | 321713 | 5471 | 316242 | FALSE |
| 575.8 | Other disorders of biliary tract | Digestive | 0.96 | 0.91 | 1.02 | 0.22 | 311372 | 1049 | 310323 | FALSE |
| 574.1 | Cholelithiasis | Digestive | 1.01 | 0.99 | 1.03 | 0.22 | 319629 | 9306 | 310323 | FALSE |
| 458.1 | Orthostatic hypotension | Circulatory System | 0.97 | 0.92 | 1.02 | 0.22 | 195553 | 1374 | 194179 | FALSE |
| 729 | Other disorders of soft tissues | Musculoskeletal | 1.01 | 0.99 | 1.02 | 0.22 | 327478 | 22524 | 304954 | FALSE |

| | | | | | | | | | | |
|---|---|---|---|---|---|---|---|---|---|---|
| 331 | Other cerebral degenerations | Neurological | 1.08 | 0.95 | 1.23 | 0.22 | 286392 | 240 | 286152 | FALSE |
| 634 | Miscarriage; stillbirth | Pregnancy Complications | 0.98 | 0.95 | 1.01 | 0.22 | 324534 | 4569 | 319965 | FALSE |
| 193 | Thyroid cancer | Neoplasms | 0.94 | 0.85 | 1.04 | 0.23 | 327009 | 367 | 326642 | FALSE |
| 790.6 | Other abnormal blood chemistry | Symptoms | 0.98 | 0.96 | 1.01 | 0.23 | 328029 | 6529 | 321500 | FALSE |
| 41.4 | E. coli | Infectious Diseases | 0.98 | 0.94 | 1.01 | 0.23 | 315432 | 2800 | 312632 | FALSE |
| 740.12 | Osteoarthrosis, localized, secondary | Musculoskeletal | 0.93 | 0.82 | 1.05 | 0.23 | 306589 | 264 | 306325 | FALSE |
| 218.1 | Uterine leiomyoma | Neoplasms | 0.99 | 0.97 | 1.01 | 0.23 | 318346 | 10566 | 307780 | FALSE |
| 450 | Noninfectious disorders of lymphatic channels | Circulatory System | 0.96 | 0.89 | 1.03 | 0.23 | 328240 | 717 | 327523 | FALSE |
| 497 | Bronchitis | Respiratory | 1.05 | 0.97 | 1.13 | 0.23 | 294834 | 642 | 294192 | FALSE |
| 519 | Other diseases of respiratory system, not elsewhere classified | Respiratory | 0.99 | 0.99 | 1.00 | 0.23 | 327615 | 62168 | 265447 | FALSE |
| 427.12 | Paroxysmal ventricular tachycardia | Circulatory System | 1.04 | 0.98 | 1.11 | 0.23 | 300490 | 969 | 299521 | FALSE |
| 743.9 | Osteopenia or other disorder of bone and cartilage | Musculoskeletal | 0.98 | 0.95 | 1.01 | 0.23 | 328188 | 3989 | 324199 | FALSE |
| 965.1 | Opiates and related narcotics causing adverse effects in therapeutic use | Injuries & Poisonings | 1.04 | 0.98 | 1.10 | 0.23 | 301372 | 1161 | 300211 | FALSE |
| 519.8 | Other diseases of respiratory system, NEC | Respiratory | 1.01 | 0.99 | 1.03 | 0.24 | 274529 | 9082 | 265447 | FALSE |
| 70.4 | Chronic hepatitis | Infectious Diseases | 1.07 | 0.96 | 1.20 | 0.24 | 322496 | 290 | 322206 | FALSE |
| 200 | Myeloproliferative disease | Neoplasms | 1.05 | 0.97 | 1.13 | 0.24 | 324202 | 679 | 323523 | FALSE |
| 735.3 | Hallux valgus (Bunion) | Musculoskeletal | 1.01 | 0.99 | 1.04 | 0.24 | 323592 | 6905 | 316687 | FALSE |
| 440.9 | Atherosclerosis of aorta | Circulatory System | 0.92 | 0.80 | 1.06 | 0.24 | 319562 | 202 | 319360 | FALSE |
| 274.1 | Gout | Endocrine/Metabolic | 0.97 | 0.93 | 1.02 | 0.24 | 327930 | 1668 | 326262 | FALSE |
| 618.1 | Prolapse of vaginal walls | Genitourinary | 1.01 | 0.99 | 1.04 | 0.24 | 323726 | 7484 | 316242 | FALSE |
| 458.2 | Iatrogenic hypotension | Circulatory System | 0.93 | 0.82 | 1.05 | 0.25 | 194417 | 238 | 194179 | FALSE |
| 556 | Ulceration of the lower GI tract | Digestive | 0.95 | 0.86 | 1.04 | 0.25 | 258836 | 440 | 258396 | FALSE |
| 208 | Benign neoplasm of colon | Neoplasms | 0.99 | 0.98 | 1.01 | 0.25 | 325437 | 20827 | 304610 | FALSE |
| 526.41 | Temporomandibular joint disorder, unspecified | Digestive | 1.08 | 0.95 | 1.23 | 0.25 | 311886 | 228 | 311658 | FALSE |
| 427.11 | Paroxysmal supraventricular tachycardia | Circulatory System | 0.98 | 0.94 | 1.02 | 0.25 | 301952 | 2431 | 299521 | FALSE |
| 184.1 | Malignant neoplasm of ovary and other uterine adnexa | Neoplasms | 0.98 | 0.94 | 1.02 | 0.25 | 308106 | 2553 | 305553 | FALSE |
| 727.1 | Synovitis and tenosynovitis | Musculoskeletal | 1.04 | 0.97 | 1.13 | 0.25 | 305641 | 687 | 304954 | FALSE |
| 646 | Other complications of pregnancy NEC | Pregnancy Complications | 1.02 | 0.98 | 1.07 | 0.26 | 328240 | 2396 | 325844 | FALSE |
| 626.1 | Irregular menstrual cycle/bleeding | Genitourinary | 1.02 | 0.99 | 1.05 | 0.26 | 299824 | 3593 | 296231 | FALSE |
| 506 | Empyema and pneumothorax | Respiratory | 0.97 | 0.91 | 1.02 | 0.26 | 317630 | 1146 | 316484 | FALSE |
| 512.7 | Shortness of breath | Respiratory | 1.01 | 0.99 | 1.04 | 0.26 | 320384 | 6116 | 314268 | FALSE |
| 317.1 | Alcoholism | Mental Disorders | 0.99 | 0.97 | 1.01 | 0.26 | 306366 | 8697 | 297669 | FALSE |
| 286.12 | Congenital deficiency of other clotting factors (including factor VII) | Hematopoietic | 0.94 | 0.84 | 1.05 | 0.26 | 325800 | 303 | 325497 | FALSE |
| 371.3 | Inflammation of eyelids | Sense Organs | 0.98 | 0.94 | 1.02 | 0.26 | 320786 | 2398 | 318388 | FALSE |
| 571.6 | Primary biliary cirrhosis | Digestive | 0.93 | 0.82 | 1.05 | 0.26 | 318589 | 258 | 318331 | FALSE |
| 458 | Hypotension | Circulatory System | 0.97 | 0.91 | 1.03 | 0.26 | 195260 | 1081 | 194179 | FALSE |
| 465.2 | Acute pharyngitis | Respiratory | 0.96 | 0.90 | 1.03 | 0.26 | 326053 | 874 | 325179 | FALSE |
| 292 | Neurological disorders | Mental Disorders | 1.05 | 0.96 | 1.16 | 0.27 | 321919 | 449 | 321470 | FALSE |

| | | | | | | | | | | |
|---|---|---|---|---|---|---|---|---|---|---|
| 297.2 | Suicide or self-inflicted injury | Mental Disorders | 1.02 | 0.98 | 1.06 | 0.27 | 285655 | 2757 | 282898 | FALSE |
| 364.5 | Corneal dystrophy | Sense Organs | 1.08 | 0.94 | 1.23 | 0.27 | 316998 | 216 | 316782 | FALSE |
| 870.4 | Open wound of nose and sinus | Injuries & Poisonings | 1.07 | 0.95 | 1.22 | 0.27 | 319969 | 238 | 319731 | FALSE |
| 994.2 | Sepsis | Injuries & Poisonings | 0.98 | 0.94 | 1.02 | 0.27 | 328240 | 2885 | 325355 | FALSE |
| 597 | Other disorders of urethra and urinary tract | Genitourinary | 0.96 | 0.90 | 1.03 | 0.27 | 314510 | 874 | 313636 | FALSE |
| 604 | Disorders of penis | Genitourinary | 1.04 | 0.97 | 1.12 | 0.27 | 308641 | 735 | 307906 | FALSE |
| 724.9 | Other unspecified back disorders | Musculoskeletal | 1.01 | 0.99 | 1.02 | 0.27 | 328029 | 17085 | 310944 | FALSE |
| 564.1 | Irritable Bowel Syndrome | Digestive | 1.01 | 0.99 | 1.04 | 0.27 | 264057 | 5661 | 258396 | FALSE |
| 202 | Cancer of other lymphoid, histiocytic tissue | Neoplasms | 0.97 | 0.91 | 1.03 | 0.27 | 324661 | 1138 | 323523 | FALSE |
| 569 | Other disorders of intestine | Digestive | 1.00 | 1.00 | 1.01 | 0.27 | 326148 | 79488 | 246660 | FALSE |
| 337 | Disorders of the autonomic nervous system | Neurological | 1.08 | 0.94 | 1.24 | 0.28 | 286359 | 207 | 286152 | FALSE |
| 695.42 | Systemic lupus erythematosus | Dermatologic | 1.06 | 0.96 | 1.17 | 0.28 | 320784 | 363 | 320421 | FALSE |
| 803.1 | Fracture of humerus | Injuries & Poisonings | 0.94 | 0.84 | 1.05 | 0.28 | 323850 | 293 | 323557 | FALSE |
| 535.2 | Atrophic gastritis | Digestive | 1.06 | 0.96 | 1.17 | 0.28 | 296869 | 358 | 296511 | FALSE |
| 619.4 | Noninflammatory disorders of vagina | Genitourinary | 1.03 | 0.98 | 1.08 | 0.28 | 280328 | 1780 | 278548 | FALSE |
| 272.11 | Hypercholesterolemia | Endocrine/Metabolic | 0.99 | 0.98 | 1.01 | 0.28 | 325499 | 34002 | 291497 | FALSE |
| 627.3 | Postmenopausal atrophic vaginitis | Genitourinary | 1.03 | 0.97 | 1.10 | 0.28 | 297359 | 1128 | 296231 | FALSE |
| 242 | Thyrotoxicosis with or without goiter | Endocrine/Metabolic | 0.97 | 0.92 | 1.02 | 0.28 | 311794 | 1465 | 310329 | FALSE |
| 560.1 | Paralytic ileus | Digestive | 0.95 | 0.87 | 1.04 | 0.28 | 258916 | 520 | 258396 | FALSE |
| 225.1 | Benign neoplasm of brain, cranial nerves, meninges | Neoplasms | 1.04 | 0.97 | 1.11 | 0.28 | 327267 | 820 | 326447 | FALSE |
| 716.1 | Unspecified polyarthropathy or polyarthritis | Musculoskeletal | 1.02 | 0.99 | 1.05 | 0.28 | 277426 | 3530 | 273896 | FALSE |
| 292.3 | Memory loss | Mental Disorders | 0.96 | 0.89 | 1.03 | 0.28 | 322208 | 738 | 321470 | FALSE |
| 530.1 | Esophagitis, GERD and related diseases | Digestive | 0.99 | 0.97 | 1.01 | 0.29 | 297342 | 9539 | 287803 | FALSE |
| 365.11 | Primary open angle glaucoma | Sense Organs | 1.03 | 0.97 | 1.10 | 0.29 | 317838 | 1056 | 316782 | FALSE |
| 540.1 | Appendicitis | Digestive | 1.04 | 0.97 | 1.11 | 0.29 | 325174 | 795 | 324379 | FALSE |
| 504 | Other alveolar and parietoalveolar pneumonopathy | Respiratory | 0.94 | 0.84 | 1.05 | 0.29 | 316793 | 309 | 316484 | FALSE |
| 614.51 | Cervicitis and endocervicitis | Genitourinary | 0.97 | 0.92 | 1.03 | 0.29 | 320048 | 1249 | 318799 | FALSE |
| 592.13 | Chronic interstitial cystitis | Genitourinary | 0.94 | 0.83 | 1.06 | 0.29 | 297289 | 254 | 297035 | FALSE |
| 559 | Ileostomy status | Digestive | 1.03 | 0.98 | 1.08 | 0.29 | 260069 | 1673 | 258396 | FALSE |
| 433.5 | Cerebral aneurysm | Circulatory System | 0.95 | 0.86 | 1.05 | 0.29 | 318505 | 398 | 318107 | FALSE |
| 771 | Musculoskeletal symptoms referable to limbs | Symptoms | 1.04 | 0.97 | 1.11 | 0.29 | 327405 | 891 | 326514 | FALSE |
| 636.3 | Hemorrhage in early pregnancy | Pregnancy Complications | 1.03 | 0.97 | 1.10 | 0.29 | 321078 | 1113 | 319965 | FALSE |
| 172.2 | Other non-epithelial cancer of skin | Neoplasms | 1.01 | 0.99 | 1.03 | 0.30 | 325462 | 11242 | 314220 | FALSE |
| 512.1 | Wheezing | Respiratory | 1.07 | 0.94 | 1.22 | 0.30 | 314499 | 231 | 314268 | FALSE |
| 594.2 | Calculus of lower urinary tract | Genitourinary | 0.96 | 0.90 | 1.03 | 0.30 | 320761 | 830 | 319931 | FALSE |
| 751.22 | Other specified congenital anomalies of kidney | Congenital Anomalies | 0.95 | 0.85 | 1.05 | 0.30 | 326353 | 350 | 326003 | FALSE |
| 870.1 | Open wound or laceration of eye or eyelid | Injuries & Poisonings | 0.94 | 0.83 | 1.06 | 0.30 | 320012 | 281 | 319731 | FALSE |
| 136 | Other infectious and parasitic diseases | Infectious Diseases | 1.05 | 0.96 | 1.16 | 0.30 | 327982 | 428 | 327554 | FALSE |

| | | | | | | | | | | |
|---|---|---|---|---|---|---|---|---|---|---|
| 246 | Other disorders of thyroid | Endocrine/Metabolic | 0.99 | 0.98 | 1.01 | 0.30 | 327738 | 17409 | 310329 | FALSE |
| 967 | Adverse effects of sedatives or other central nervous system depressants and anesthetics | Injuries & Poisonings | 0.96 | 0.88 | 1.04 | 0.30 | 300790 | 579 | 300211 | FALSE |
| 480 | Pneumonia | Respiratory | 0.98 | 0.95 | 1.02 | 0.30 | 321603 | 3914 | 317689 | FALSE |
| 384.4 | Perforation of tympanic membrane | Sense Organs | 0.97 | 0.91 | 1.03 | 0.30 | 325167 | 1024 | 324143 | FALSE |
| 724.1 | Disorders of sacrum | Musculoskeletal | 0.94 | 0.84 | 1.06 | 0.30 | 311225 | 281 | 310944 | FALSE |
| 724.2 | Disorders of coccyx | Musculoskeletal | 0.94 | 0.84 | 1.06 | 0.30 | 311226 | 282 | 310944 | FALSE |
| 512 | Other symptoms of respiratory system | Respiratory | 0.99 | 0.96 | 1.01 | 0.30 | 319772 | 5504 | 314268 | FALSE |
| 359.2 | Myopathy | Neurological | 0.95 | 0.87 | 1.04 | 0.30 | 326450 | 498 | 325952 | FALSE |
| 655 | Known or suspected fetal abnormality affecting management of mother | Pregnancy Complications | 1.02 | 0.99 | 1.05 | 0.30 | 328240 | 4575 | 323665 | FALSE |
| 427.41 | Ventricular fibrillation and flutter | Circulatory System | 1.06 | 0.95 | 1.17 | 0.30 | 299866 | 345 | 299521 | FALSE |
| 213 | Benign neoplasm of bone and articular cartilage | Neoplasms | 1.06 | 0.95 | 1.18 | 0.31 | 277528 | 322 | 277206 | FALSE |
| 276.41 | Acidosis | Endocrine/Metabolic | 0.97 | 0.91 | 1.03 | 0.31 | 321700 | 1056 | 320644 | FALSE |
| 557 | Intestinal malabsorption (non-celiac) | Digestive | 1.06 | 0.95 | 1.19 | 0.31 | 258690 | 294 | 258396 | FALSE |
| 153.3 | Malignant neoplasm of rectum, rectosigmoid junction, and anus | Neoplasms | 0.98 | 0.94 | 1.02 | 0.31 | 303397 | 2117 | 301280 | FALSE |
| 153.2 | Colon cancer | Neoplasms | 0.98 | 0.95 | 1.02 | 0.31 | 304402 | 3122 | 301280 | FALSE |
| 686.1 | Carbuncle and furuncle | Dermatologic | 1.02 | 0.98 | 1.06 | 0.31 | 318829 | 2422 | 316407 | FALSE |
| 728.7 | Fasciitis | Musculoskeletal | 0.93 | 0.81 | 1.07 | 0.31 | 305167 | 213 | 304954 | FALSE |
| 180.3 | Cervical intraepithelial neoplasia [CIN] [Cervical dysplasia] | Neoplasms | 1.02 | 0.98 | 1.07 | 0.31 | 299736 | 2153 | 297583 | FALSE |
| 530 | Diseases of esophagus | Digestive | 1.04 | 0.97 | 1.11 | 0.31 | 288604 | 801 | 287803 | FALSE |
| 574.2 | Calculus of bile duct | Digestive | 1.02 | 0.98 | 1.06 | 0.31 | 313011 | 2688 | 310323 | FALSE |
| 579 | Other symptoms involving abdomen and pelvis | Digestive | 0.97 | 0.92 | 1.03 | 0.31 | 304580 | 1186 | 303394 | FALSE |
| 145.2 | Cancer of tongue | Neoplasms | 0.95 | 0.85 | 1.05 | 0.32 | 326366 | 325 | 326041 | FALSE |
| 367.1 | Myopia | Sense Organs | 1.03 | 0.97 | 1.09 | 0.32 | 327025 | 1271 | 325754 | FALSE |
| 443.1 | Raynaud's syndrome | Circulatory System | 1.03 | 0.97 | 1.09 | 0.32 | 320525 | 1165 | 319360 | FALSE |
| 516.1 | Hemoptysis | Respiratory | 1.02 | 0.98 | 1.07 | 0.32 | 328154 | 2103 | 326051 | FALSE |
| 714.1 | Rheumatoid arthritis | Musculoskeletal | 1.02 | 0.98 | 1.06 | 0.32 | 319560 | 2307 | 317253 | FALSE |
| 352.1 | Trigeminal nerve disorders [CN5] | Neurological | 0.95 | 0.87 | 1.05 | 0.32 | 313473 | 462 | 313011 | FALSE |
| 598 | Abnormal findings on examination of urine | Genitourinary | 1.02 | 0.98 | 1.05 | 0.33 | 328240 | 3386 | 324854 | FALSE |
| 276.6 | Fluid overload | Endocrine/Metabolic | 0.96 | 0.87 | 1.05 | 0.33 | 321117 | 473 | 320644 | FALSE |
| 736 | Other acquired deformities of limbs | Musculoskeletal | 1.06 | 0.95 | 1.18 | 0.33 | 316998 | 311 | 316687 | FALSE |
| 599.4 | Urinary incontinence | Genitourinary | 1.01 | 0.99 | 1.03 | 0.33 | 229423 | 8996 | 220427 | FALSE |
| 696.4 | Psoriasis | Dermatologic | 1.02 | 0.98 | 1.08 | 0.33 | 315554 | 1622 | 313932 | FALSE |
| 738 | Other acquired musculoskeletal deformity | Musculoskeletal | 0.95 | 0.87 | 1.05 | 0.33 | 317104 | 417 | 316687 | FALSE |
| 189 | Cancer of urinary organs (incl. kidney and bladder) | Neoplasms | 1.02 | 0.98 | 1.06 | 0.33 | 326965 | 3003 | 323962 | FALSE |
| 496 | Chronic airway obstruction | Respiratory | 0.99 | 0.97 | 1.01 | 0.33 | 301827 | 7635 | 294192 | FALSE |
| 276.11 | Hyperosmolality and/or hypernatremia | Endocrine/Metabolic | 1.06 | 0.94 | 1.20 | 0.33 | 320891 | 247 | 320644 | FALSE |
| 741 | Symptoms and disorders of the joints | Musculoskeletal | 1.01 | 0.99 | 1.02 | 0.34 | 327531 | 16889 | 310642 | FALSE |
| 381.9 | Otorrhea | Sense Organs | 0.94 | 0.84 | 1.06 | 0.34 | 324420 | 277 | 324143 | FALSE |

| | | | | | | | | | | |
|---|---|---|---|---|---|---|---|---|---|---|
| 353 | Nerve root and plexus disorders | Neurological | 1.03 | 0.97 | 1.10 | 0.34 | 314005 | 994 | 313011 | FALSE |
| 602 | Other disorders of prostate | Genitourinary | 0.97 | 0.92 | 1.03 | 0.34 | 308765 | 1372 | 307393 | FALSE |
| 625 | Pain and other symptoms associated with female genital organs | Genitourinary | 0.98 | 0.94 | 1.02 | 0.34 | 320718 | 2049 | 318669 | FALSE |
| 608 | Other disorders of male genital organs | Genitourinary | 1.01 | 0.99 | 1.02 | 0.34 | 327998 | 20092 | 307906 | FALSE |
| 622.2 | Mucous polyp of cervix | Genitourinary | 0.98 | 0.95 | 1.02 | 0.34 | 318829 | 3522 | 315307 | FALSE |
| 78 | Viral warts & HPV | Infectious Diseases | 1.03 | 0.97 | 1.10 | 0.34 | 323218 | 1012 | 322206 | FALSE |
| 289.3 | Personal history of diseases of blood and blood-forming organs | Hematopoietic | 0.95 | 0.84 | 1.06 | 0.34 | 320615 | 297 | 320318 | FALSE |
| 250.7 | Diabetic retinopathy | Endocrine/Metabolic | 0.97 | 0.92 | 1.03 | 0.35 | 317232 | 1380 | 315852 | FALSE |
| 627.2 | Symptomatic menopause | Genitourinary | 1.07 | 0.93 | 1.23 | 0.35 | 296434 | 203 | 296231 | FALSE |
| 442.11 | Abdominal aortic aneurysm | Circulatory System | 0.97 | 0.91 | 1.04 | 0.35 | 320257 | 897 | 319360 | FALSE |
| 737.3 | Kyphoscoliosis and scoliosis | Musculoskeletal | 0.95 | 0.86 | 1.06 | 0.35 | 317058 | 371 | 316687 | FALSE |
| 372 | Disorders of conjunctiva | Sense Organs | 0.97 | 0.91 | 1.03 | 0.35 | 319431 | 1043 | 318388 | FALSE |
| 350.3 | Lack of coordination | Neurological | 1.05 | 0.95 | 1.16 | 0.35 | 325804 | 360 | 325444 | FALSE |
| 496.21 | Obstructive chronic bronchitis | Respiratory | 0.98 | 0.95 | 1.02 | 0.35 | 296921 | 2729 | 294192 | FALSE |
| 597.1 | Urethral stricture (not specified as infectious) | Genitourinary | 1.02 | 0.98 | 1.05 | 0.35 | 317079 | 3443 | 313636 | FALSE |
| 789 | Nausea and vomiting | Symptoms | 0.99 | 0.97 | 1.01 | 0.35 | 328240 | 11983 | 316257 | FALSE |
| 512.8 | Cough | Respiratory | 1.02 | 0.98 | 1.05 | 0.35 | 317232 | 2964 | 314268 | FALSE |
| 289.4 | Lymphadenitis | Hematopoietic | 0.98 | 0.95 | 1.02 | 0.35 | 323033 | 2715 | 320318 | FALSE |
| 427.7 | Tachycardia NOS | Circulatory System | 1.02 | 0.98 | 1.06 | 0.35 | 301787 | 2266 | 299521 | FALSE |
| 187.1 | Malignant neoplasm of unspecified male genital organ | Neoplasms | 0.99 | 0.97 | 1.01 | 0.35 | 327699 | 8481 | 319218 | FALSE |
| 801 | Fracture of ankle and foot | Injuries & Poisonings | 0.94 | 0.83 | 1.07 | 0.36 | 323808 | 251 | 323557 | FALSE |
| 38 | Septicemia | Infectious Diseases | 0.98 | 0.95 | 1.02 | 0.36 | 315677 | 3045 | 312632 | FALSE |
| 70 | Viral hepatitis | Infectious Diseases | 1.04 | 0.96 | 1.11 | 0.36 | 322928 | 722 | 322206 | FALSE |
| 681.3 | Cellulitis and abscess of arm/hand | Dermatologic | 0.99 | 0.96 | 1.01 | 0.36 | 322177 | 5770 | 316407 | FALSE |
| 751.2 | Congenital anomalies of urinary system | Congenital Anomalies | 0.97 | 0.91 | 1.04 | 0.36 | 326908 | 905 | 326003 | FALSE |
| 590 | Pyelonephritis | Genitourinary | 1.01 | 0.98 | 1.05 | 0.36 | 300960 | 3925 | 297035 | FALSE |
| 618 | Genital prolapse | Genitourinary | 0.96 | 0.89 | 1.04 | 0.36 | 316862 | 620 | 316242 | FALSE |
| 740.11 | Osteoarthrosis, localized, primary | Musculoskeletal | 1.01 | 0.99 | 1.03 | 0.36 | 315552 | 9227 | 306325 | FALSE |
| 427.4 | Cardiac arrest and ventricular fibrillation | Circulatory System | 1.05 | 0.95 | 1.17 | 0.36 | 299869 | 348 | 299521 | FALSE |
| 327 | Sleep disorders | Neurological | 1.03 | 0.97 | 1.09 | 0.36 | 323754 | 1034 | 322720 | FALSE |
| 81 | Infection/inflammation of internal prosthetic device; implant; and graft | Infectious Diseases | 0.98 | 0.94 | 1.02 | 0.36 | 323993 | 2514 | 321479 | FALSE |
| 573.5 | Jaundice (not of newborn) | Digestive | 1.03 | 0.97 | 1.10 | 0.36 | 319269 | 938 | 318331 | FALSE |
| 577.3 | Cyst and pseudocyst of pancreas | Digestive | 0.95 | 0.86 | 1.06 | 0.37 | 326295 | 375 | 325920 | FALSE |
| 202.2 | Non-Hodgkins lymphoma | Neoplasms | 1.02 | 0.98 | 1.05 | 0.37 | 327055 | 3532 | 323523 | FALSE |
| 345.1 | Epilepsy | Neurological | 1.05 | 0.94 | 1.17 | 0.37 | 286483 | 331 | 286152 | FALSE |
| 364 | Corneal opacity and other disorders of cornea | Sense Organs | 1.06 | 0.93 | 1.21 | 0.37 | 317009 | 227 | 316782 | FALSE |
| 296.1 | Bipolar | Mental Disorders | 1.03 | 0.97 | 1.09 | 0.37 | 284019 | 1121 | 282898 | FALSE |
| 601.8 | Other inflammatory disorders of male genital organs | Genitourinary | 0.95 | 0.85 | 1.06 | 0.37 | 307698 | 305 | 307393 | FALSE |

| | | | | | | | | | | |
|---|---|---|---|---|---|---|---|---|---|---|
| 454.1 | Varicose veins of lower extremity | Circulatory System | 1.01 | 0.99 | 1.03 | 0.37 | 299275 | 11562 | 287713 | FALSE |
| 430.2 | Intracerebral hemorrhage | Circulatory System | 0.97 | 0.90 | 1.04 | 0.37 | 318830 | 723 | 318107 | FALSE |
| 585.2 | Renal failure NOS | Genitourinary | 0.99 | 0.97 | 1.01 | 0.37 | 322824 | 8745 | 314079 | FALSE |
| 715 | Other inflammatory spondylopathies | Musculoskeletal | 1.05 | 0.94 | 1.19 | 0.38 | 317524 | 271 | 317253 | FALSE |
| 327.3 | Sleep apnea | Neurological | 1.01 | 0.98 | 1.04 | 0.38 | 327419 | 4699 | 322720 | FALSE |
| 727 | Other disorders of synovium, tendon, and bursa | Musculoskeletal | 1.03 | 0.96 | 1.11 | 0.38 | 305708 | 754 | 304954 | FALSE |
| 290.1 | Dementias | Mental Disorders | 1.04 | 0.96 | 1.12 | 0.38 | 322121 | 651 | 321470 | FALSE |
| 613.8 | Other specified disorders of breast | Genitourinary | 0.95 | 0.86 | 1.06 | 0.38 | 322773 | 355 | 322418 | FALSE |
| 272.9 | Unspecified disorder of lipoid metabolism | Endocrine/Metabolic | 0.94 | 0.83 | 1.08 | 0.39 | 291721 | 224 | 291497 | FALSE |
| 854 | Complications of cardiac/vascular device, implant, and graft | Injuries & Poisonings | 0.98 | 0.94 | 1.03 | 0.39 | 315807 | 1843 | 313964 | FALSE |
| 681.6 | Cellulitis and abscess of foot, toe | Dermatologic | 0.99 | 0.96 | 1.01 | 0.39 | 322141 | 5734 | 316407 | FALSE |
| 172.3 | Carcinoma in situ of skin | Neoplasms | 1.03 | 0.96 | 1.12 | 0.39 | 314884 | 664 | 314220 | FALSE |
| 613.1 | Inflammatory disease of breast | Genitourinary | 0.97 | 0.90 | 1.04 | 0.39 | 323156 | 738 | 322418 | FALSE |
| 836 | Traumatic arthropathy | Injuries & Poisonings | 1.06 | 0.93 | 1.20 | 0.39 | 322016 | 243 | 321773 | FALSE |
| 446.5 | Giant cell arteritis | Circulatory System | 0.96 | 0.87 | 1.06 | 0.39 | 319750 | 390 | 319360 | FALSE |
| 509.1 | Respiratory failure | Respiratory | 0.98 | 0.94 | 1.02 | 0.39 | 318564 | 2080 | 316484 | FALSE |
| 455 | Hemorrhoids | Circulatory System | 0.99 | 0.98 | 1.01 | 0.39 | 312410 | 24697 | 287713 | FALSE |
| 586.4 | Stricture/obstruction of ureter | Genitourinary | 0.97 | 0.91 | 1.04 | 0.39 | 315018 | 939 | 314079 | FALSE |
| 149.1 | Cancer of oropharynx | Neoplasms | 1.05 | 0.93 | 1.19 | 0.39 | 326297 | 256 | 326041 | FALSE |
| 550 | Abdominal hernia | Digestive | 1.00 | 0.99 | 1.01 | 0.39 | 328240 | 48451 | 279789 | FALSE |
| 269 | Proteinuria | Endocrine/Metabolic | 1.05 | 0.94 | 1.16 | 0.39 | 327850 | 355 | 327495 | FALSE |
| 369 | Infection of the eye | Sense Organs | 0.95 | 0.84 | 1.07 | 0.40 | 318642 | 254 | 318388 | FALSE |
| 344 | Other paralytic syndromes | Neurological | 0.97 | 0.90 | 1.04 | 0.40 | 286841 | 689 | 286152 | FALSE |
| 514 | Abnormal findings examination of lungs | Respiratory | 1.02 | 0.98 | 1.06 | 0.40 | 328212 | 2577 | 325635 | FALSE |
| 90 | Sexually transmitted infections (not HIV or hepatitis) | Infectious Diseases | 1.05 | 0.94 | 1.18 | 0.40 | 328239 | 298 | 327941 | FALSE |
| 433.21 | Cerebral artery occlusion, with cerebral infarction | Circulatory System | 0.98 | 0.93 | 1.03 | 0.40 | 319641 | 1534 | 318107 | FALSE |
| 427.5 | Arrhythmia (cardiac) NOS | Circulatory System | 0.97 | 0.91 | 1.04 | 0.40 | 300469 | 948 | 299521 | FALSE |
| 348.2 | Cerebral edema and compression of brain | Neurological | 1.05 | 0.93 | 1.19 | 0.41 | 286412 | 260 | 286152 | FALSE |
| 368.4 | Visual field defects | Sense Organs | 0.96 | 0.86 | 1.06 | 0.41 | 325215 | 333 | 324882 | FALSE |
| 429.2 | Abnormal function study of cardiovascular system | Circulatory System | 0.96 | 0.89 | 1.05 | 0.41 | 322577 | 544 | 322033 | FALSE |
| 275.3 | Disorders of magnesium metabolism | Endocrine/Metabolic | 0.96 | 0.87 | 1.06 | 0.41 | 326410 | 375 | 326035 | FALSE |
| 362.2 | Degeneration of macula and posterior pole of retina | Sense Organs | 0.98 | 0.94 | 1.02 | 0.41 | 318055 | 2203 | 315852 | FALSE |
| 362.29 | Macular degeneration (senile) of retina NOS | Sense Organs | 0.98 | 0.94 | 1.02 | 0.41 | 318052 | 2200 | 315852 | FALSE |
| 415 | Pulmonary heart disease | Circulatory System | 1.01 | 0.98 | 1.05 | 0.41 | 325300 | 3753 | 321547 | FALSE |
| 348.7 | Coma | Neurological | 1.04 | 0.94 | 1.15 | 0.41 | 286531 | 379 | 286152 | FALSE |
| 185 | Cancer of prostate | Neoplasms | 0.99 | 0.97 | 1.01 | 0.41 | 315856 | 8463 | 307393 | FALSE |
| 599.3 | Dysuria | Genitourinary | 1.02 | 0.97 | 1.08 | 0.41 | 221636 | 1209 | 220427 | FALSE |
| 681.5 | Cellulitis and abscess of leg, except foot | Dermatologic | 0.99 | 0.96 | 1.02 | 0.42 | 322184 | 5777 | 316407 | FALSE |

| | | | | | | | | | | |
|---|---|---|---|---|---|---|---|---|---|---|
| 614.32 | Chronic inflammatory pelvic disease | Genitourinary | 0.96 | 0.87 | 1.06 | 0.42 | 319224 | 425 | 318799 | FALSE |
| 214.1 | Lipoma of skin and subcutaneous tissue | Neoplasms | 0.99 | 0.96 | 1.02 | 0.42 | 325432 | 4756 | 320676 | FALSE |
| 348.9 | Other conditions of brain, NOS | Neurological | 1.04 | 0.94 | 1.15 | 0.42 | 286559 | 407 | 286152 | FALSE |
| 578.1 | Hematemesis | Digestive | 0.98 | 0.94 | 1.03 | 0.42 | 305428 | 2034 | 303394 | FALSE |
| 441.1 | Acute vascular insufficiency of intestine | Circulatory System | 1.05 | 0.93 | 1.18 | 0.43 | 319631 | 271 | 319360 | FALSE |
| 276.13 | Hyperpotassemia | Endocrine/Metabolic | 0.97 | 0.92 | 1.04 | 0.43 | 321623 | 979 | 320644 | FALSE |
| 275.1 | Disorders of iron metabolism | Hematopoietic | 1.03 | 0.96 | 1.11 | 0.43 | 326735 | 700 | 326035 | FALSE |
| 394.3 | Aortic valve disease | Circulatory System | 0.98 | 0.93 | 1.03 | 0.43 | 322836 | 1308 | 321528 | FALSE |
| 279.7 | Other immunological findings | Endocrine/Metabolic | 0.95 | 0.84 | 1.08 | 0.44 | 327978 | 264 | 327714 | FALSE |
| 159 | Malignant neoplasm of other and ill-defined sites within the digestive organs and peritoneum | Neoplasms | 0.99 | 0.96 | 1.02 | 0.44 | 317620 | 5289 | 312331 | FALSE |
| 195.1 | Malignant neoplasm, other | Neoplasms | 1.00 | 0.99 | 1.00 | 0.44 | 320138 | 90826 | 229312 | FALSE |
| 430.1 | Subarachnoid hemorrhage | Circulatory System | 1.03 | 0.96 | 1.10 | 0.44 | 318931 | 824 | 318107 | FALSE |
| 444 | Arterial embolism and thrombosis | Circulatory System | 0.96 | 0.88 | 1.06 | 0.44 | 319794 | 434 | 319360 | FALSE |
| 586 | Other disorders of the kidney and ureters | Genitourinary | 0.99 | 0.95 | 1.02 | 0.44 | 317490 | 3411 | 314079 | FALSE |
| 687 | Symptoms affecting skin | Dermatologic | 0.95 | 0.83 | 1.08 | 0.44 | 323032 | 218 | 322814 | FALSE |
| 250.23 | Type 2 diabetes with ophthalmic manifestations | Endocrine/Metabolic | 0.98 | 0.93 | 1.03 | 0.44 | 308834 | 1344 | 307490 | FALSE |
| 433.31 | Transient cerebral ischemia | Circulatory System | 0.98 | 0.94 | 1.03 | 0.45 | 320281 | 2174 | 318107 | FALSE |
| 599.9 | Other abnormality of urination | Genitourinary | 1.02 | 0.97 | 1.06 | 0.45 | 222371 | 1944 | 220427 | FALSE |
| 555.1 | Regional enteritis | Digestive | 1.02 | 0.97 | 1.07 | 0.45 | 260201 | 1805 | 258396 | FALSE |
| 579.8 | Nonspecific abnormal findings in stool contents | Digestive | 1.02 | 0.97 | 1.07 | 0.45 | 305134 | 1740 | 303394 | FALSE |
| 634.3 | Ectopic pregnancy | Pregnancy Complications | 1.04 | 0.94 | 1.16 | 0.45 | 320307 | 342 | 319965 | FALSE |
| 451 | Phlebitis and thrombophlebitis | Circulatory System | 0.96 | 0.87 | 1.06 | 0.46 | 288110 | 397 | 287713 | FALSE |
| 697 | Sarcoidosis | Dermatologic | 1.03 | 0.95 | 1.12 | 0.46 | 322309 | 560 | 321749 | FALSE |
| 743.11 | Osteoporosis NOS | Musculoskeletal | 0.99 | 0.95 | 1.02 | 0.46 | 327235 | 3036 | 324199 | FALSE |
| 550.5 | Ventral hernia | Digestive | 1.01 | 0.98 | 1.05 | 0.46 | 283371 | 3582 | 279789 | FALSE |
| 331.1 | Hydrocephalus | Neurological | 0.97 | 0.89 | 1.06 | 0.46 | 286652 | 500 | 286152 | FALSE |
| 622.1 | Polyp of corpus uteri | Genitourinary | 1.01 | 0.99 | 1.03 | 0.47 | 323490 | 8183 | 315307 | FALSE |
| 747.13 | Congenital anomalies of great vessels | Congenital Anomalies | 0.98 | 0.94 | 1.03 | 0.47 | 327177 | 1823 | 325354 | FALSE |
| 289.5 | Diseases of spleen | Hematopoietic | 0.97 | 0.89 | 1.06 | 0.47 | 320846 | 528 | 320318 | FALSE |
| 150 | Cancer of esophagus | Neoplasms | 1.03 | 0.96 | 1.10 | 0.47 | 313058 | 727 | 312331 | FALSE |
| 540 | Appendiceal conditions | Digestive | 1.01 | 0.98 | 1.04 | 0.47 | 328240 | 3861 | 324379 | FALSE |
| 427.9 | Palpitations | Circulatory System | 0.99 | 0.96 | 1.02 | 0.48 | 303480 | 3959 | 299521 | FALSE |
| 801.1 | Fracture of foot | Injuries & Poisonings | 0.97 | 0.88 | 1.06 | 0.48 | 323975 | 418 | 323557 | FALSE |
| 635.3 | Placenta previa and abruptio placenta | Pregnancy Complications | 1.02 | 0.97 | 1.08 | 0.48 | 321301 | 1336 | 319965 | FALSE |
| 262 | Mineral deficiency NEC | Endocrine/Metabolic | 1.05 | 0.92 | 1.19 | 0.48 | 326027 | 241 | 325786 | FALSE |
| 292.4 | Altered mental status | Mental Disorders | 1.01 | 0.97 | 1.06 | 0.48 | 323742 | 2272 | 321470 | FALSE |
| 749 | Congenital anomalies of face and neck | Congenital Anomalies | 0.96 | 0.85 | 1.08 | 0.48 | 328137 | 271 | 327866 | FALSE |
| 752.11 | Spina bifida | Congenital Anomalies | 0.95 | 0.83 | 1.09 | 0.48 | 327886 | 211 | 327675 | FALSE |

| | | | | | | | | | | |
|---|---|---|---|---|---|---|---|---|---|---|
| 218.2 | Other benign neoplasm of uterus | Neoplasms | 1.04 | 0.94 | 1.15 | 0.48 | 308143 | 363 | 307780 | FALSE |
| 38.1 | Gram negative septicemia | Infectious Diseases | 1.02 | 0.96 | 1.10 | 0.48 | 313487 | 855 | 312632 | FALSE |
| 189.21 | Malignant neoplasm of bladder | Neoplasms | 1.02 | 0.97 | 1.06 | 0.48 | 326150 | 2188 | 323962 | FALSE |
| 395.2 | Nonrheumatic aortic valve disorders | Circulatory System | 0.96 | 0.84 | 1.08 | 0.48 | 321775 | 247 | 321528 | FALSE |
| 596.5 | Functional disorders of bladder | Genitourinary | 1.02 | 0.97 | 1.07 | 0.48 | 315318 | 1682 | 313636 | FALSE |
| 687.1 | Rash and other nonspecific skin eruption | Dermatologic | 1.01 | 0.97 | 1.06 | 0.49 | 325032 | 2218 | 322814 | FALSE |
| 522 | Diseases of pulp and periapical tissues | Digestive | 1.03 | 0.95 | 1.11 | 0.49 | 312297 | 639 | 311658 | FALSE |
| 722 | Intervertebral disc disorders | Musculoskeletal | 1.01 | 0.98 | 1.04 | 0.49 | 316907 | 5963 | 310944 | FALSE |
| 284 | Aplastic anemia | Hematopoietic | 0.99 | 0.98 | 1.01 | 0.49 | 321659 | 12759 | 308900 | FALSE |
| 496.3 | Bronchiectasis | Respiratory | 0.98 | 0.94 | 1.03 | 0.49 | 296077 | 1885 | 294192 | FALSE |
| 723 | Other disorders of cervical region | Musculoskeletal | 1.04 | 0.94 | 1.14 | 0.49 | 311345 | 401 | 310944 | FALSE |
| 870 | Open wounds of head; neck; and trunk | Injuries & Poisonings | 0.97 | 0.90 | 1.05 | 0.49 | 320327 | 596 | 319731 | FALSE |
| 624.1 | Dystrophy of female genital tract | Genitourinary | 0.96 | 0.84 | 1.09 | 0.49 | 318909 | 240 | 318669 | FALSE |
| 340 | Migraine | Neurological | 1.01 | 0.98 | 1.05 | 0.49 | 320723 | 2773 | 317950 | FALSE |
| 531.3 | Duodenal ulcer | Digestive | 0.99 | 0.95 | 1.02 | 0.49 | 323644 | 3095 | 320549 | FALSE |
| 300.12 | Agorophobia, social phobia, and panic disorder | Mental Disorders | 0.98 | 0.91 | 1.05 | 0.49 | 283642 | 744 | 282898 | FALSE |
| 528.5 | Diseases of lips | Digestive | 1.03 | 0.95 | 1.11 | 0.49 | 323215 | 681 | 322534 | FALSE |
| 384 | Other disorders of tympanic membrane | Sense Organs | 0.97 | 0.88 | 1.06 | 0.50 | 324573 | 430 | 324143 | FALSE |
| 292.1 | Aphasia/speech disturbance | Mental Disorders | 0.98 | 0.93 | 1.03 | 0.50 | 323019 | 1549 | 321470 | FALSE |
| 557.1 | Celiac disease | Digestive | 0.98 | 0.94 | 1.03 | 0.50 | 260290 | 1894 | 258396 | FALSE |
| 626.8 | Infertility, female | Genitourinary | 0.98 | 0.93 | 1.04 | 0.50 | 297638 | 1407 | 296231 | FALSE |
| 614.5 | Inflammatory disease of cervix, vagina, and vulva | Genitourinary | 1.04 | 0.93 | 1.15 | 0.50 | 319133 | 334 | 318799 | FALSE |
| 430.3 | Subdural hemorrhage | Circulatory System | 0.96 | 0.85 | 1.08 | 0.50 | 318378 | 271 | 318107 | FALSE |
| 819 | Skull and face fracture and other intercranial injury | Injuries & Poisonings | 1.02 | 0.97 | 1.07 | 0.51 | 328056 | 1517 | 326539 | FALSE |
| 426.4 | Anomalous atrioventricular excitation | Circulatory System | 1.04 | 0.92 | 1.18 | 0.51 | 299761 | 240 | 299521 | FALSE |
| 536.8 | Dyspepsia and other specified disorders of function of stomach | Digestive | 1.04 | 0.92 | 1.18 | 0.51 | 296758 | 247 | 296511 | FALSE |
| 394.7 | Disease of tricuspid valve | Circulatory System | 0.98 | 0.92 | 1.04 | 0.51 | 322614 | 1086 | 321528 | FALSE |
| 729.1 | Rheumatism, unspecified and fibrositis | Musculoskeletal | 1.03 | 0.94 | 1.14 | 0.52 | 305341 | 387 | 304954 | FALSE |
| 625.1 | Dyspareunia | Genitourinary | 1.02 | 0.96 | 1.08 | 0.52 | 319824 | 1155 | 318669 | FALSE |
| 362 | Other retinal disorders | Sense Organs | 0.98 | 0.92 | 1.04 | 0.52 | 316825 | 973 | 315852 | FALSE |
| 338.1 | Acute pain | Neurological | 0.97 | 0.88 | 1.07 | 0.52 | 327846 | 420 | 327426 | FALSE |
| 303.3 | Psychogenic disorder | Mental Disorders | 1.02 | 0.96 | 1.09 | 0.52 | 283739 | 841 | 282898 | FALSE |
| 531.4 | Peptic ulcer, site unspecified | Digestive | 0.97 | 0.88 | 1.06 | 0.52 | 320998 | 449 | 320549 | FALSE |
| 285.22 | Anemia in neoplastic disease | Hematopoietic | 0.97 | 0.88 | 1.07 | 0.52 | 309337 | 437 | 308900 | FALSE |
| 382 | Otalgia | Sense Organs | 1.04 | 0.93 | 1.16 | 0.52 | 324458 | 315 | 324143 | FALSE |
| 694.2 | Other dyschromia | Dermatologic | 0.98 | 0.91 | 1.05 | 0.52 | 322549 | 800 | 321749 | FALSE |
| 674 | Other complications of the puerperium NEC | Pregnancy Complications | 1.04 | 0.92 | 1.17 | 0.52 | 328015 | 280 | 327735 | FALSE |
| 614.52 | Vaginitis and vulvovaginitis | Genitourinary | 0.97 | 0.88 | 1.07 | 0.52 | 319219 | 420 | 318799 | FALSE |

| | | | | | | | | | | |
|---|---|---|---|---|---|---|---|---|---|---|
| 805 | Fracture of vertebral column without mention of spinal cord injury | Injuries & Poisonings | 0.97 | 0.87 | 1.08 | 0.52 | 323890 | 333 | 323557 | FALSE |
| 531.2 | Gastric ulcer | Digestive | 1.01 | 0.98 | 1.04 | 0.53 | 324777 | 4228 | 320549 | FALSE |
| 788 | Syncope and collapse | Symptoms | 0.99 | 0.97 | 1.01 | 0.53 | 328240 | 9372 | 318868 | FALSE |
| 871 | Open wounds of extremities | Injuries & Poisonings | 0.99 | 0.96 | 1.02 | 0.53 | 323873 | 4142 | 319731 | FALSE |
| 591 | Urinary tract infection | Genitourinary | 1.01 | 0.99 | 1.02 | 0.53 | 309711 | 12676 | 297035 | FALSE |
| 263 | Other nutritional deficiency | Endocrine/Metabolic | 0.98 | 0.94 | 1.03 | 0.53 | 327337 | 1551 | 325786 | FALSE |
| 627 | Menopausal and postmenopausal disorders | Genitourinary | 1.02 | 0.95 | 1.09 | 0.53 | 297078 | 847 | 296231 | FALSE |
| 740.9 | Osteoarthrosis NOS | Musculoskeletal | 1.01 | 0.98 | 1.04 | 0.54 | 310757 | 4432 | 306325 | FALSE |
| 389.4 | Tinnitus | Sense Organs | 1.03 | 0.94 | 1.12 | 0.54 | 318735 | 541 | 318194 | FALSE |
| 613 | Other nonmalignant breast conditions | Genitourinary | 0.97 | 0.87 | 1.07 | 0.54 | 322786 | 368 | 322418 | FALSE |
| 420.2 | Pericarditis | Circulatory System | 0.98 | 0.92 | 1.04 | 0.54 | 326070 | 1087 | 324983 | FALSE |
| 705.8 | Hyperhidrosis | Dermatologic | 1.03 | 0.95 | 1.11 | 0.54 | 318896 | 602 | 318294 | FALSE |
| 293 | Symptoms involving head and neck | Mental Disorders | 1.01 | 0.97 | 1.06 | 0.54 | 327323 | 2204 | 325119 | FALSE |
| 274.21 | Chondrocalcinosis | Endocrine/Metabolic | 1.04 | 0.92 | 1.16 | 0.54 | 326552 | 290 | 326262 | FALSE |
| 610.3 | Fibrosclerosis of breast | Genitourinary | 0.97 | 0.86 | 1.08 | 0.55 | 321176 | 303 | 320873 | FALSE |
| 500.2 | Pneumoconiosis | Respiratory | 1.02 | 0.96 | 1.08 | 0.55 | 317485 | 1001 | 316484 | FALSE |
| 747.11 | Cardiac shunt/ heart septal defect | Congenital Anomalies | 0.98 | 0.90 | 1.06 | 0.55 | 325935 | 581 | 325354 | FALSE |
| 853 | Complication of colostomy or enterostomy | Injuries & Poisonings | 1.03 | 0.94 | 1.12 | 0.55 | 314478 | 514 | 313964 | FALSE |
| 383 | Otosclerosis | Sense Organs | 1.03 | 0.93 | 1.15 | 0.55 | 324473 | 330 | 324143 | FALSE |
| 761 | Cervicalgia | Symptoms | 0.98 | 0.92 | 1.04 | 0.55 | 328240 | 1005 | 327235 | FALSE |
| 480.1 | Bacterial pneumonia | Respiratory | 0.98 | 0.92 | 1.05 | 0.55 | 318628 | 939 | 317689 | FALSE |
| 184.2 | Cancer of other female genital organs | Neoplasms | 0.97 | 0.87 | 1.08 | 0.55 | 305876 | 323 | 305553 | FALSE |
| 726.2 | Synoviopathy | Musculoskeletal | 0.97 | 0.87 | 1.08 | 0.55 | 305285 | 331 | 304954 | FALSE |
| 703.1 | Ingrowing nail | Dermatologic | 0.98 | 0.92 | 1.04 | 0.56 | 322439 | 1010 | 321429 | FALSE |
| 155 | Cancer of liver and intrahepatic bile duct | Neoplasms | 1.04 | 0.92 | 1.18 | 0.56 | 312576 | 245 | 312331 | FALSE |
| 174 | Breast cancer | Neoplasms | 1.04 | 0.92 | 1.16 | 0.56 | 304712 | 288 | 304424 | FALSE |
| 79 | Viral infection | Infectious Diseases | 1.01 | 0.98 | 1.04 | 0.56 | 326439 | 4233 | 322206 | FALSE |
| 242.1 | Graves' disease | Endocrine/Metabolic | 0.97 | 0.89 | 1.07 | 0.56 | 310789 | 460 | 310329 | FALSE |
| 447 | Other disorders of arteries and arterioles | Circulatory System | 1.02 | 0.94 | 1.11 | 0.56 | 319916 | 556 | 319360 | FALSE |
| 368.2 | Diplopia and disorders of binocular vision | Sense Organs | 1.02 | 0.95 | 1.10 | 0.56 | 325635 | 753 | 324882 | FALSE |
| 835 | Internal derangement of knee | Injuries & Poisonings | 1.01 | 0.98 | 1.04 | 0.56 | 326012 | 4239 | 321773 | FALSE |
| 201 | Hodgkin's disease | Neoplasms | 0.97 | 0.86 | 1.09 | 0.57 | 323794 | 271 | 323523 | FALSE |
| 198.1 | Secondary malignancy of lymph nodes | Neoplasms | 0.99 | 0.97 | 1.02 | 0.57 | 234815 | 5503 | 229312 | FALSE |
| 550.4 | Umbilical hernia | Digestive | 1.01 | 0.98 | 1.04 | 0.57 | 283693 | 3904 | 279789 | FALSE |
| 750.21 | Congenital anomalies of intestine | Congenital Anomalies | 0.96 | 0.85 | 1.10 | 0.57 | 326230 | 227 | 326003 | FALSE |
| 333.4 | Torsion dystonia | Neurological | 0.97 | 0.86 | 1.09 | 0.57 | 286417 | 265 | 286152 | FALSE |
| 427.42 | Cardiac arrest | Circulatory System | 0.98 | 0.92 | 1.05 | 0.57 | 300470 | 949 | 299521 | FALSE |
| 747.12 | Valvular heart disease/ heart chambers | Congenital Anomalies | 0.96 | 0.85 | 1.10 | 0.58 | 325592 | 238 | 325354 | FALSE |

| | | | | | | | | | | |
|---|---|---|---|---|---|---|---|---|---|---|
| 210 | Benign neoplasm of lip, oral cavity, and pharynx | Neoplasms | 1.02 | 0.96 | 1.08 | 0.58 | 327034 | 993 | 326041 | FALSE |
| 474.1 | Acute tonsillitis | Respiratory | 0.98 | 0.90 | 1.06 | 0.58 | 309515 | 565 | 308950 | FALSE |
| 698 | Pruritus and related conditions | Dermatologic | 1.02 | 0.95 | 1.09 | 0.58 | 328240 | 799 | 327441 | FALSE |
| 323 | Encephalitis | Neurological | 1.02 | 0.95 | 1.09 | 0.58 | 327999 | 861 | 327138 | FALSE |
| 189.11 | Malignant neoplasm of kidney, except pelvis | Neoplasms | 0.98 | 0.92 | 1.05 | 0.58 | 324997 | 1035 | 323962 | FALSE |
| 872 | Traumatic amputation | Injuries & Poisonings | 0.98 | 0.89 | 1.07 | 0.58 | 320213 | 482 | 319731 | FALSE |
| 614.1 | Pelvic peritoneal adhesions, female (postoperative) (postinfection) | Genitourinary | 0.99 | 0.95 | 1.03 | 0.58 | 321370 | 2571 | 318799 | FALSE |
| 369.5 | Conjunctivitis, infectious | Sense Organs | 1.04 | 0.91 | 1.18 | 0.58 | 318612 | 224 | 318388 | FALSE |
| 495.2 | Asthma with exacerbation | Respiratory | 1.03 | 0.92 | 1.17 | 0.58 | 294453 | 261 | 294192 | FALSE |
| 715.2 | Ankylosing spondylitis | Musculoskeletal | 0.97 | 0.88 | 1.08 | 0.58 | 317637 | 384 | 317253 | FALSE |
| 614.53 | Cyst or abscess of Bartholin's gland | Genitourinary | 0.98 | 0.91 | 1.05 | 0.59 | 319602 | 803 | 318799 | FALSE |
| 756 | Other congenital musculoskeletal anomalies | Congenital Anomalies | 0.98 | 0.92 | 1.05 | 0.59 | 328123 | 812 | 327311 | FALSE |
| 301 | Personality disorders | Mental Disorders | 1.03 | 0.93 | 1.13 | 0.59 | 283330 | 432 | 282898 | FALSE |
| 823 | Fracture of tibia and fibula | Injuries & Poisonings | 0.97 | 0.87 | 1.08 | 0.59 | 322362 | 342 | 322020 | FALSE |
| 172.11 | Melanomas of skin | Neoplasms | 1.01 | 0.97 | 1.05 | 0.59 | 316943 | 2723 | 314220 | FALSE |
| 714 | Rheumatoid arthritis and other inflammatory polyarthropathies | Musculoskeletal | 1.01 | 0.98 | 1.03 | 0.60 | 325905 | 8652 | 317253 | FALSE |
| 536 | Disorders of function of stomach | Digestive | 1.03 | 0.91 | 1.17 | 0.60 | 296768 | 257 | 296511 | FALSE |
| 619.2 | Disorders of uterus, NEC | Genitourinary | 1.01 | 0.98 | 1.04 | 0.60 | 281939 | 3391 | 278548 | FALSE |
| 938 | Dermatitis due to solar radiation | Injuries & Poisonings | 1.01 | 0.97 | 1.05 | 0.60 | 324444 | 2538 | 321906 | FALSE |
| 386.3 | Labyrinthitis | Sense Organs | 0.98 | 0.92 | 1.05 | 0.60 | 322440 | 791 | 321649 | FALSE |
| 800.3 | Fracture of tibia and fibula | Injuries & Poisonings | 0.98 | 0.90 | 1.06 | 0.60 | 324092 | 535 | 323557 | FALSE |
| 624.2 | Atrophy of female genital tract | Genitourinary | 1.02 | 0.93 | 1.13 | 0.61 | 319111 | 442 | 318669 | FALSE |
| 338.2 | Chronic pain | Neurological | 1.03 | 0.93 | 1.13 | 0.61 | 327830 | 404 | 327426 | FALSE |
| 612.2 | Hypertrophy of breast (Gynecomastia) | Genitourinary | 1.02 | 0.95 | 1.09 | 0.61 | 321732 | 859 | 320873 | FALSE |
| 614 | Inflammatory diseases of female pelvic organs | Genitourinary | 1.03 | 0.93 | 1.14 | 0.61 | 319173 | 374 | 318799 | FALSE |
| 427.6 | Premature beats | Circulatory System | 1.02 | 0.93 | 1.12 | 0.61 | 299979 | 458 | 299521 | FALSE |
| 519.9 | Symptoms involving respiratory system and other chest symptoms | Respiratory | 0.97 | 0.87 | 1.09 | 0.61 | 265755 | 308 | 265447 | FALSE |
| 293.1 | Swelling, mass, or lump in head and neck [Space-occupying lesion, intracranial NOS] | Mental Disorders | 1.02 | 0.95 | 1.08 | 0.61 | 326044 | 925 | 325119 | FALSE |
| 592.1 | Cystitis | Genitourinary | 0.99 | 0.95 | 1.03 | 0.61 | 299089 | 2054 | 297035 | FALSE |
| 701.5 | Abnormal granulation tissue | Dermatologic | 1.02 | 0.94 | 1.12 | 0.61 | 323543 | 479 | 323064 | FALSE |
| 291.8 | Alteration of consciousness | Mental Disorders | 1.02 | 0.93 | 1.13 | 0.61 | 321904 | 434 | 321470 | FALSE |
| 260.6 | Anorexia | Endocrine/Metabolic | 1.02 | 0.95 | 1.09 | 0.61 | 326636 | 850 | 325786 | FALSE |
| 669 | Complications of labor and delivery NEC | Pregnancy Complications | 1.01 | 0.98 | 1.03 | 0.61 | 328240 | 9534 | 318706 | FALSE |
| 528.7 | Sialolithiasis | Digestive | 0.97 | 0.87 | 1.09 | 0.61 | 322847 | 313 | 322534 | FALSE |
| 250.11 | Type 1 diabetes with ketoacidosis | Endocrine/Metabolic | 0.97 | 0.86 | 1.10 | 0.62 | 307745 | 255 | 307490 | FALSE |
| 653 | Problems associated with amniotic cavity and membranes | Pregnancy Complications | 1.01 | 0.96 | 1.07 | 0.62 | 328119 | 1523 | 326596 | FALSE |
| 603.1 | Hydrocele | Genitourinary | 1.01 | 0.96 | 1.07 | 0.62 | 309308 | 1402 | 307906 | FALSE |
| 585.3 | Chronic renal failure [CKD] | Genitourinary | 1.01 | 0.97 | 1.05 | 0.62 | 316664 | 2585 | 314079 | FALSE |

| | | | | | | | | | | |
|---|---|---|---|---|---|---|---|---|---|---|
| 323.8 | Encephalitis, non-infectious | Neurological | 1.02 | 0.94 | 1.10 | 0.62 | 327771 | 633 | 327138 | FALSE |
| 709.2 | Sicca syndrome | Dermatologic | 1.02 | 0.94 | 1.11 | 0.62 | 242486 | 520 | 241966 | FALSE |
| 523.32 | Chronic periodontitis | Digestive | 1.02 | 0.94 | 1.11 | 0.62 | 312248 | 590 | 311658 | FALSE |
| 532 | Dysphagia | Digestive | 0.99 | 0.97 | 1.02 | 0.62 | 294356 | 6553 | 287803 | FALSE |
| 740.2 | Osteoarthrosis, generalized | Musculoskeletal | 0.98 | 0.90 | 1.06 | 0.62 | 306914 | 589 | 306325 | FALSE |
| 426.32 | Left bundle branch block | Circulatory System | 0.99 | 0.94 | 1.04 | 0.62 | 301302 | 1781 | 299521 | FALSE |
| 447.1 | Stricture of artery | Circulatory System | 0.98 | 0.92 | 1.05 | 0.63 | 320246 | 886 | 319360 | FALSE |
| 696.41 | Psoriasis vulgaris | Dermatologic | 1.01 | 0.96 | 1.06 | 0.63 | 315655 | 1723 | 313932 | FALSE |
| 512.2 | Painful respiration | Respiratory | 1.03 | 0.91 | 1.18 | 0.63 | 314496 | 228 | 314268 | FALSE |
| 626 | Disorders of menstruation and other abnormal bleeding from female genital tract | Genitourinary | 1.01 | 0.98 | 1.04 | 0.63 | 300178 | 3947 | 296231 | FALSE |
| 595 | Hydronephrosis | Genitourinary | 0.99 | 0.95 | 1.03 | 0.63 | 321941 | 2010 | 319931 | FALSE |
| 300.13 | Phobia | Mental Disorders | 1.02 | 0.94 | 1.11 | 0.63 | 283412 | 514 | 282898 | FALSE |
| 340.1 | Migrain with aura | Neurological | 1.03 | 0.90 | 1.18 | 0.63 | 318167 | 217 | 317950 | FALSE |
| 626.12 | Excessive or frequent menstruation | Genitourinary | 1.00 | 0.97 | 1.02 | 0.64 | 306272 | 10041 | 296231 | FALSE |
| 446.9 | Arteritis NOS | Circulatory System | 1.03 | 0.91 | 1.17 | 0.64 | 319605 | 245 | 319360 | FALSE |
| 575.7 | Other disorders of gallbladder | Digestive | 1.01 | 0.96 | 1.07 | 0.64 | 311719 | 1396 | 310323 | FALSE |
| 385.3 | Cholesteatoma | Sense Organs | 1.02 | 0.94 | 1.11 | 0.64 | 324732 | 589 | 324143 | FALSE |
| 550.1 | Inguinal hernia | Digestive | 1.00 | 0.98 | 1.01 | 0.64 | 296174 | 16385 | 279789 | FALSE |
| 635.2 | Antepartum hemorrhage, abruptio placentae, and placenta previa | Pregnancy Complications | 1.02 | 0.95 | 1.08 | 0.65 | 320901 | 936 | 319965 | FALSE |
| 735.2 | Acquired toe deformities | Musculoskeletal | 1.01 | 0.97 | 1.05 | 0.65 | 318913 | 2226 | 316687 | FALSE |
| 261.2 | Vitamin B-complex deficiencies | Endocrine/Metabolic | 0.98 | 0.92 | 1.06 | 0.65 | 326559 | 773 | 325786 | FALSE |
| 965 | Poisoning by analgesics, antipyretics, and antirheumatics | Injuries & Poisonings | 1.01 | 0.98 | 1.03 | 0.65 | 306293 | 6082 | 300211 | FALSE |
| 530.7 | Gastroesophageal laceration-hemorrhage syndrome | Digestive | 1.03 | 0.92 | 1.14 | 0.65 | 288136 | 333 | 287803 | FALSE |
| 275.5 | Disorders of calcium/phosphorus metabolism | Endocrine/Metabolic | 1.01 | 0.96 | 1.08 | 0.65 | 327144 | 1109 | 326035 | FALSE |
| 345.3 | Convulsions | Neurological | 0.99 | 0.95 | 1.03 | 0.65 | 288463 | 2311 | 286152 | FALSE |
| 427.8 | Sinoatrial node dysfunction (Bradycardia) | Circulatory System | 0.98 | 0.89 | 1.08 | 0.65 | 299940 | 419 | 299521 | FALSE |
| 287.3 | Thrombocytopenia | Hematopoietic | 0.99 | 0.93 | 1.04 | 0.65 | 326768 | 1271 | 325497 | FALSE |
| 949 | Allergies, other | Injuries & Poisonings | 1.02 | 0.93 | 1.12 | 0.65 | 322326 | 420 | 321906 | FALSE |
| 443.7 | Peripheral angiopathy in diseases classified elsewhere | Circulatory System | 1.02 | 0.93 | 1.13 | 0.65 | 319775 | 415 | 319360 | FALSE |
| 751.12 | Congenital anomalies of male genital organs | Congenital Anomalies | 1.03 | 0.90 | 1.17 | 0.65 | 326231 | 228 | 326003 | FALSE |
| 379 | Other disorders of eye | Sense Organs | 1.00 | 0.99 | 1.01 | 0.65 | 328190 | 39998 | 288192 | FALSE |
| 577 | Diseases of pancreas | Digestive | 1.02 | 0.94 | 1.11 | 0.66 | 326478 | 558 | 325920 | FALSE |
| 613.7 | Other signs and symptoms in breast | Genitourinary | 0.98 | 0.92 | 1.06 | 0.66 | 323169 | 751 | 322418 | FALSE |
| 312 | Conduct disorders | Mental Disorders | 1.01 | 0.96 | 1.07 | 0.66 | 327638 | 1195 | 326443 | FALSE |
| 958 | Certain early complications of trauma or procedure | Injuries & Poisonings | 1.02 | 0.92 | 1.13 | 0.66 | 328118 | 381 | 327737 | FALSE |
| 579.2 | Splenomegaly | Digestive | 1.02 | 0.93 | 1.12 | 0.66 | 303837 | 443 | 303394 | FALSE |
| 531.1 | Hemorrhage from gastrointestinal ulcer | Digestive | 0.98 | 0.91 | 1.06 | 0.66 | 321204 | 655 | 320549 | FALSE |
| 807 | Fracture of ribs | Injuries & Poisonings | 1.03 | 0.90 | 1.17 | 0.66 | 323780 | 223 | 323557 | FALSE |

| | | | | | | | | | | |
|---|---|---|---|---|---|---|---|---|---|---|
| 433.1 | Occlusion and stenosis of precerebral arteries | Circulatory System | 0.99 | 0.93 | 1.05 | 0.66 | 319149 | 1042 | 318107 | FALSE |
| 433.8 | Late effects of cerebrovascular disease | Circulatory System | 0.99 | 0.93 | 1.04 | 0.66 | 319384 | 1277 | 318107 | FALSE |
| 302 | Sexual and gender identity disorders | Mental Disorders | 1.02 | 0.92 | 1.14 | 0.67 | 283238 | 340 | 282898 | FALSE |
| 53 | Herpes zoster | Infectious Diseases | 1.02 | 0.92 | 1.14 | 0.67 | 322547 | 341 | 322206 | FALSE |
| 573.7 | Abnormal results of function study of liver | Digestive | 1.01 | 0.97 | 1.04 | 0.67 | 321896 | 3565 | 318331 | FALSE |
| 357 | Inflammatory and toxic neuropathy | Neurological | 1.01 | 0.96 | 1.07 | 0.67 | 327375 | 1423 | 325952 | FALSE |
| 244.4 | Hypothyroidism NOS | Endocrine/Metabolic | 1.00 | 0.98 | 1.01 | 0.68 | 324759 | 14430 | 310329 | FALSE |
| 426.9 | Cardiac pacemaker/device in situ | Circulatory System | 1.03 | 0.90 | 1.17 | 0.68 | 299758 | 237 | 299521 | FALSE |
| 626.14 | Irregular menstrual bleeding | Genitourinary | 0.99 | 0.96 | 1.03 | 0.68 | 300177 | 3946 | 296231 | FALSE |
| 317 | Alcohol-related disorders | Mental Disorders | 0.99 | 0.97 | 1.02 | 0.68 | 303529 | 5860 | 297669 | FALSE |
| 961 | Poisoning by other anti-infectives | Injuries & Poisonings | 0.98 | 0.89 | 1.08 | 0.68 | 300647 | 436 | 300211 | FALSE |
| 701.2 | Scar conditions and fibrosis of skin | Dermatologic | 0.99 | 0.95 | 1.03 | 0.68 | 325471 | 2407 | 323064 | FALSE |
| 440 | Atherosclerosis | Circulatory System | 0.97 | 0.85 | 1.12 | 0.68 | 319564 | 204 | 319360 | FALSE |
| 735.21 | Hammer toe (acquired) | Musculoskeletal | 1.01 | 0.97 | 1.05 | 0.68 | 318701 | 2014 | 316687 | FALSE |
| 212 | Benign neoplasm of respiratory and intrathoracic organs | Neoplasms | 1.02 | 0.92 | 1.13 | 0.69 | 328240 | 355 | 327885 | FALSE |
| 426.91 | Cardiac pacemaker in situ | Circulatory System | 0.99 | 0.95 | 1.03 | 0.69 | 301837 | 2316 | 299521 | FALSE |
| 720 | Spinal stenosis | Musculoskeletal | 0.98 | 0.91 | 1.06 | 0.69 | 311636 | 692 | 310944 | FALSE |
| 964.1 | Anticoagulants causing adverse effects | Injuries & Poisonings | 1.03 | 0.91 | 1.16 | 0.69 | 300470 | 259 | 300211 | FALSE |
| 367.8 | Hypermetropia | Sense Organs | 1.03 | 0.90 | 1.16 | 0.69 | 325995 | 241 | 325754 | FALSE |
| 501 | Pneumonitis due to inhalation of food or vomitus | Respiratory | 1.02 | 0.94 | 1.10 | 0.69 | 317076 | 592 | 316484 | FALSE |
| 740.1 | Osteoarthritis; localized | Musculoskeletal | 1.00 | 0.98 | 1.02 | 0.69 | 315797 | 9472 | 306325 | FALSE |
| 613.9 | Breast disorder NOS | Genitourinary | 0.99 | 0.97 | 1.02 | 0.69 | 327937 | 5519 | 322418 | FALSE |
| 433 | Cerebrovascular disease | Circulatory System | 1.00 | 0.97 | 1.02 | 0.69 | 326753 | 8646 | 318107 | FALSE |
| 575.9 | Nonspecific abnormal findings on radiological and other examination of biliary tract | Digestive | 1.02 | 0.92 | 1.13 | 0.70 | 310690 | 367 | 310323 | FALSE |
| 755.1 | Congenital deformities of feet | Congenital Anomalies | 1.03 | 0.90 | 1.17 | 0.70 | 327531 | 220 | 327311 | FALSE |
| 334.2 | Anterior horn cell disease | Neurological | 1.03 | 0.90 | 1.17 | 0.70 | 286376 | 224 | 286152 | FALSE |
| 575.2 | Obstruction of bile duct | Digestive | 0.99 | 0.92 | 1.06 | 0.70 | 311135 | 812 | 310323 | FALSE |
| 619.1 | Noninflammatory disorders of ovary, fallopian tube, and broad ligament | Genitourinary | 0.99 | 0.92 | 1.06 | 0.70 | 279282 | 734 | 278548 | FALSE |
| 594.1 | Calculus of kidney | Genitourinary | 0.99 | 0.96 | 1.03 | 0.70 | 323265 | 3334 | 319931 | FALSE |
| 306.9 | Tension headache | Mental Disorders | 1.02 | 0.91 | 1.14 | 0.71 | 283203 | 305 | 282898 | FALSE |
| 427 | Cardiac dysrhythmias | Circulatory System | 0.98 | 0.88 | 1.09 | 0.71 | 299844 | 323 | 299521 | FALSE |
| 530.14 | Reflux esophagitis | Digestive | 1.00 | 0.98 | 1.02 | 0.71 | 298696 | 10893 | 287803 | FALSE |
| 520.2 | Disturbances in tooth eruption | Digestive | 1.01 | 0.97 | 1.05 | 0.71 | 314095 | 2437 | 311658 | FALSE |
| 189.2 | Cancer of bladder | Neoplasms | 0.99 | 0.92 | 1.06 | 0.71 | 324798 | 836 | 323962 | FALSE |
| 840 | Sprains and strains | Injuries & Poisonings | 1.02 | 0.93 | 1.11 | 0.71 | 328201 | 481 | 327720 | FALSE |
| 565.1 | Anal and rectal polyp | Digestive | 1.00 | 0.97 | 1.02 | 0.71 | 254329 | 7669 | 246660 | FALSE |
| 362.4 | Retinal vascular changes and abnomalities | Sense Organs | 0.99 | 0.92 | 1.06 | 0.71 | 316733 | 881 | 315852 | FALSE |
| 253.2 | Pituitary hypofunction | Endocrine/Metabolic | 0.98 | 0.87 | 1.10 | 0.71 | 324826 | 281 | 324545 | FALSE |

| | | | | | | | | | | |
|---|---|---|---|---|---|---|---|---|---|---|
| 285.2 | Anemia of chronic disease | Hematopoietic | 0.98 | 0.87 | 1.10 | 0.71 | 309194 | 294 | 308900 | FALSE |
| 227.2 | Benign neoplasm of parathyroid gland | Neoplasms | 0.98 | 0.89 | 1.08 | 0.72 | 327032 | 390 | 326642 | FALSE |
| 965.3 | Salicylates causing adverse effects in therapeutic use | Injuries & Poisonings | 0.98 | 0.89 | 1.08 | 0.72 | 300612 | 401 | 300211 | FALSE |
| 379.5 | Disorders of iris and ciliary body | Sense Organs | 1.02 | 0.92 | 1.12 | 0.72 | 288604 | 412 | 288192 | FALSE |
| 575.6 | Cholesterolosis of gallbladder | Digestive | 0.98 | 0.90 | 1.08 | 0.72 | 310789 | 466 | 310323 | FALSE |
| 622 | Polyp of female genital organs | Genitourinary | 0.98 | 0.90 | 1.08 | 0.72 | 315794 | 487 | 315307 | FALSE |
| 621 | Endometrial hyperplasia | Genitourinary | 1.01 | 0.95 | 1.07 | 0.72 | 316461 | 1154 | 315307 | FALSE |
| 426.21 | First degree AV block | Circulatory System | 1.01 | 0.95 | 1.08 | 0.72 | 300526 | 1005 | 299521 | FALSE |
| 941 | Adverse reaction to serum or vaccine | Injuries & Poisonings | 1.02 | 0.90 | 1.17 | 0.72 | 322128 | 222 | 321906 | FALSE |
| 601.4 | Balanoposthitis | Genitourinary | 1.02 | 0.91 | 1.14 | 0.72 | 307689 | 296 | 307393 | FALSE |
| 550.3 | Femoral hernia | Digestive | 1.01 | 0.94 | 1.10 | 0.72 | 280442 | 653 | 279789 | FALSE |
| 385.5 | Tympanosclerosis and middle ear disease related to otitis media | Sense Organs | 0.98 | 0.86 | 1.11 | 0.72 | 324379 | 236 | 324143 | FALSE |
| 618.6 | Vaginal enterocele, congenital or acquired | Genitourinary | 1.01 | 0.94 | 1.09 | 0.72 | 316909 | 667 | 316242 | FALSE |
| 586.2 | Cyst of kidney, acquired | Genitourinary | 0.99 | 0.94 | 1.05 | 0.72 | 315372 | 1293 | 314079 | FALSE |
| 367.9 | Blindness and low vision | Sense Organs | 0.99 | 0.92 | 1.06 | 0.72 | 326510 | 756 | 325754 | FALSE |
| 555.2 | Ulcerative colitis | Digestive | 1.01 | 0.97 | 1.04 | 0.73 | 261550 | 3154 | 258396 | FALSE |
| 727.4 | Ganglion and cyst of synovium, tendon, and bursa | Musculoskeletal | 0.99 | 0.96 | 1.03 | 0.73 | 307936 | 2982 | 304954 | FALSE |
| 204 | Leukemia | Neoplasms | 1.02 | 0.93 | 1.11 | 0.73 | 323984 | 461 | 323523 | FALSE |
| 649.1 | Diabetes or abnormal glucose tolerance complicating pregnancy | Pregnancy Complications | 0.98 | 0.85 | 1.12 | 0.73 | 328127 | 208 | 327919 | FALSE |
| 245.21 | Chronic lymphocytic thyroiditis | Endocrine/Metabolic | 0.98 | 0.86 | 1.12 | 0.73 | 310548 | 219 | 310329 | FALSE |
| 764 | Sciatica | Symptoms | 0.99 | 0.94 | 1.05 | 0.73 | 327944 | 1261 | 326683 | FALSE |
| 560.2 | Impaction of intestine | Digestive | 1.02 | 0.91 | 1.15 | 0.73 | 258667 | 271 | 258396 | FALSE |
| 568.1 | Peritoneal adhesions (postoperative) (postinfection) | Digestive | 0.99 | 0.96 | 1.03 | 0.73 | 249757 | 3097 | 246660 | FALSE |
| 594 | Urinary calculus | Genitourinary | 1.00 | 0.97 | 1.02 | 0.74 | 326897 | 6966 | 319931 | FALSE |
| 296.2 | Depression | Mental Disorders | 1.00 | 0.98 | 1.02 | 0.74 | 295043 | 12145 | 282898 | FALSE |
| 261.4 | Vitamin D deficiency | Endocrine/Metabolic | 1.02 | 0.92 | 1.12 | 0.74 | 326194 | 408 | 325786 | FALSE |
| 454.11 | Varicose veins of lower extremity, symptomtic | Circulatory System | 0.99 | 0.91 | 1.07 | 0.74 | 288365 | 652 | 287713 | FALSE |
| 440.2 | Atherosclerosis of the extremities | Circulatory System | 1.01 | 0.93 | 1.10 | 0.74 | 319912 | 552 | 319360 | FALSE |
| 567 | Peritonitis and retroperitoneal infections | Digestive | 0.99 | 0.93 | 1.06 | 0.74 | 247550 | 890 | 246660 | FALSE |
| 112 | Candidiasis | Infectious Diseases | 1.01 | 0.97 | 1.05 | 0.74 | 327650 | 2150 | 325500 | FALSE |
| 396 | Abnormal heart sounds | Circulatory System | 1.01 | 0.95 | 1.07 | 0.74 | 322603 | 1075 | 321528 | FALSE |
| 389.1 | Sensorineural hearing loss | Sense Organs | 1.02 | 0.93 | 1.11 | 0.74 | 318658 | 464 | 318194 | FALSE |
| 957 | Injury to other and unspecified nerves | Injuries & Poisonings | 1.02 | 0.91 | 1.15 | 0.74 | 328157 | 275 | 327882 | FALSE |
| 520 | Disorders of tooth development | Digestive | 1.02 | 0.93 | 1.11 | 0.74 | 312128 | 470 | 311658 | FALSE |
| 381.1 | Otitis media | Sense Organs | 1.01 | 0.95 | 1.07 | 0.74 | 325296 | 1153 | 324143 | FALSE |
| 771.1 | Swelling of limb | Symptoms | 0.99 | 0.92 | 1.06 | 0.74 | 327356 | 842 | 326514 | FALSE |
| 564 | Functional digestive disorders | Digestive | 0.99 | 0.93 | 1.05 | 0.74 | 259379 | 983 | 258396 | FALSE |
| 342 | Hemiplegia | Neurological | 1.01 | 0.96 | 1.06 | 0.74 | 287677 | 1525 | 286152 | FALSE |

| | | | | | | | | | | | |
|---|---|---|---|---|---|---|---|---|---|---|---|
| 600 | Hyperplasia of prostate | Genitourinary | 1.00 | 0.98 | 1.02 | 0.75 | 318965 | 11572 | 307393 | FALSE |
| 580.2 | Nephrotic syndrome without mention of glomerulonephritis | Genitourinary | 0.99 | 0.90 | 1.08 | 0.75 | 314575 | 496 | 314079 | FALSE |
| 610.4 | Benign neoplasm of breast | Genitourinary | 1.01 | 0.96 | 1.06 | 0.75 | 322359 | 1486 | 320873 | FALSE |
| 523.31 | Acute periodontitis | Digestive | 0.99 | 0.92 | 1.06 | 0.75 | 312371 | 713 | 311658 | FALSE |
| 785 | Abdominal pain | Symptoms | 1.00 | 0.99 | 1.01 | 0.75 | 328240 | 42311 | 285929 | FALSE |
| 721.1 | Spondylosis without myelopathy | Musculoskeletal | 1.01 | 0.95 | 1.07 | 0.75 | 312014 | 1070 | 310944 | FALSE |
| 803.3 | Fracture of clavicle or scapula | Injuries & Poisonings | 0.98 | 0.88 | 1.10 | 0.75 | 323857 | 300 | 323557 | FALSE |
| 803.2 | Fracture of radius and ulna | Injuries & Poisonings | 0.99 | 0.93 | 1.06 | 0.75 | 324441 | 884 | 323557 | FALSE |
| 701.4 | Keloid scar | Dermatologic | 1.02 | 0.90 | 1.16 | 0.75 | 323292 | 228 | 323064 | FALSE |
| 182 | Malignant neoplasm of uterus | Neoplasms | 0.99 | 0.94 | 1.05 | 0.75 | 298948 | 1303 | 297645 | FALSE |
| 285 | Other anemias | Hematopoietic | 1.00 | 0.98 | 1.02 | 0.75 | 320744 | 11844 | 308900 | FALSE |
| 568 | Other disorders of peritoneum | Digestive | 0.99 | 0.96 | 1.03 | 0.75 | 250464 | 3804 | 246660 | FALSE |
| 256.4 | Polycystic ovaries | Endocrine/Metabolic | 0.98 | 0.86 | 1.12 | 0.75 | 324764 | 219 | 324545 | FALSE |
| 158 | Neoplasm of unspecified nature of digestive system | Neoplasms | 0.99 | 0.93 | 1.05 | 0.75 | 313429 | 1098 | 312331 | FALSE |
| 290.11 | Alzheimer's disease | Mental Disorders | 1.02 | 0.92 | 1.12 | 0.75 | 321872 | 402 | 321470 | FALSE |
| 227.3 | Benign neoplasm of pituitary gland and craniopharyngeal duct (pouch) | Neoplasms | 1.02 | 0.91 | 1.13 | 0.75 | 326970 | 328 | 326642 | FALSE |
| 296 | Mood disorders | Mental Disorders | 1.00 | 0.98 | 1.01 | 0.76 | 295853 | 12955 | 282898 | FALSE |
| 379.3 | Aphakia and other disorders of lens | Sense Organs | 1.00 | 0.98 | 1.01 | 0.76 | 308920 | 20728 | 288192 | FALSE |
| 803 | Fracture of upper limb | Injuries & Poisonings | 0.99 | 0.90 | 1.08 | 0.76 | 323990 | 433 | 323557 | FALSE |
| 214 | Lipoma | Neoplasms | 0.99 | 0.95 | 1.04 | 0.76 | 322495 | 1819 | 320676 | FALSE |
| 70.3 | Viral hepatitis C | Infectious Diseases | 1.02 | 0.91 | 1.13 | 0.77 | 322549 | 343 | 322206 | FALSE |
| 618.5 | Prolapse of vaginal vault after hysterectomy | Genitourinary | 1.01 | 0.93 | 1.11 | 0.77 | 316743 | 501 | 316242 | FALSE |
| 401.22 | Hypertensive chronic kidney disease | Circulatory System | 0.99 | 0.94 | 1.04 | 0.77 | 250343 | 1595 | 248748 | FALSE |
| 572 | Ascites (non malignant) | Digestive | 1.01 | 0.96 | 1.06 | 0.77 | 319925 | 1594 | 318331 | FALSE |
| 830 | Dislocation | Injuries & Poisonings | 0.99 | 0.95 | 1.04 | 0.77 | 323927 | 2154 | 321773 | FALSE |
| 614.4 | Inflammatory diseases of uterus, except cervix | Genitourinary | 1.02 | 0.91 | 1.14 | 0.77 | 319107 | 308 | 318799 | FALSE |
| 627.1 | Postmenopausal bleeding | Genitourinary | 1.00 | 0.98 | 1.02 | 0.77 | 305697 | 9466 | 296231 | FALSE |
| 250.1 | Type 1 diabetes | Endocrine/Metabolic | 1.01 | 0.97 | 1.04 | 0.77 | 310189 | 2699 | 307490 | FALSE |
| 596.1 | Bladder neck obstruction | Genitourinary | 1.01 | 0.96 | 1.05 | 0.77 | 315665 | 2029 | 313636 | FALSE |
| 523 | Gingival and periodontal diseases | Digestive | 1.01 | 0.93 | 1.10 | 0.77 | 312187 | 529 | 311658 | FALSE |
| 578.2 | Blood in stool | Digestive | 1.01 | 0.97 | 1.04 | 0.78 | 306143 | 2749 | 303394 | FALSE |
| 610.2 | Fibroadenosis of breast | Genitourinary | 0.98 | 0.88 | 1.10 | 0.78 | 321189 | 316 | 320873 | FALSE |
| 165.1 | Cancer of bronchus; lung | Neoplasms | 1.01 | 0.96 | 1.05 | 0.78 | 327450 | 2181 | 325269 | FALSE |
| 960.2 | Allergy/adverse effect of penicillin | Injuries & Poisonings | 1.00 | 0.98 | 1.01 | 0.78 | 316894 | 16683 | 300211 | FALSE |
| 189.4 | Malignant neoplasm of other urinary organs | Neoplasms | 1.00 | 0.97 | 1.04 | 0.78 | 327185 | 3223 | 323962 | FALSE |
| 386.1 | Meniere's disease | Sense Organs | 0.99 | 0.91 | 1.07 | 0.78 | 322213 | 564 | 321649 | FALSE |
| 791 | Gangrene | Symptoms | 0.99 | 0.91 | 1.07 | 0.78 | 328240 | 579 | 327661 | FALSE |
| 386.2 | Peripheral or central vertigo | Sense Organs | 1.01 | 0.92 | 1.12 | 0.78 | 322036 | 387 | 321649 | FALSE |

| | | | | | | | | | | |
|---|---|---|---|---|---|---|---|---|---|---|
| 191 | Malignant and unknown neoplasms of brain and nervous system | Neoplasms | 0.98 | 0.86 | 1.12 | 0.78 | 326662 | 215 | 326447 | FALSE |
| 535.6 | Duodenitis | Digestive | 1.00 | 0.98 | 1.03 | 0.79 | 304405 | 7894 | 296511 | FALSE |
| 277.4 | Disorders of bilirubin excretion | Endocrine/Metabolic | 1.01 | 0.92 | 1.12 | 0.79 | 283300 | 373 | 282927 | FALSE |
| 522.5 | Periapical abscess | Digestive | 0.99 | 0.94 | 1.05 | 0.79 | 312904 | 1246 | 311658 | FALSE |
| 561 | Symptoms involving digestive system | Digestive | 1.00 | 0.98 | 1.01 | 0.79 | 272941 | 14545 | 258396 | FALSE |
| 615 | Endometriosis | Genitourinary | 1.00 | 0.97 | 1.03 | 0.79 | 322888 | 4089 | 318799 | FALSE |
| 368.9 | Subjective visual disturbances | Sense Organs | 1.01 | 0.93 | 1.09 | 0.79 | 325515 | 633 | 324882 | FALSE |
| 759 | Other and unspecified congenital anomalies | Congenital Anomalies | 0.99 | 0.92 | 1.07 | 0.79 | 327249 | 679 | 326570 | FALSE |
| 512.9 | Other dyspnea | Respiratory | 0.99 | 0.93 | 1.06 | 0.79 | 315211 | 943 | 314268 | FALSE |
| 706 | Diseases of sebaceous glands | Dermatologic | 0.99 | 0.91 | 1.07 | 0.79 | 318889 | 595 | 318294 | FALSE |
| 345.11 | Generalized convulsive epilepsy | Neurological | 1.01 | 0.92 | 1.11 | 0.79 | 286617 | 465 | 286152 | FALSE |
| 502 | Postinflammatory pulmonary fibrosis | Respiratory | 0.99 | 0.93 | 1.06 | 0.79 | 317367 | 883 | 316484 | FALSE |
| 707 | Chronic ulcer of skin | Dermatologic | 1.01 | 0.95 | 1.06 | 0.80 | 327959 | 1260 | 326699 | FALSE |
| 296.22 | Major depressive disorder | Mental Disorders | 1.01 | 0.92 | 1.12 | 0.80 | 283282 | 384 | 282898 | FALSE |
| 760 | Back pain | Symptoms | 1.00 | 0.98 | 1.03 | 0.80 | 328240 | 5923 | 322317 | FALSE |
| 276.12 | Hyposmolality and/or hyponatremia | Endocrine/Metabolic | 1.01 | 0.96 | 1.05 | 0.80 | 322496 | 1852 | 320644 | FALSE |
| 433.2 | Occlusion of cerebral arteries | Circulatory System | 1.00 | 0.96 | 1.03 | 0.80 | 321087 | 2980 | 318107 | FALSE |
| 377 | Disorders of optic nerve and visual pathways | Sense Organs | 1.01 | 0.92 | 1.11 | 0.80 | 288628 | 436 | 288192 | FALSE |
| 531 | Peptic ulcer (excl. esophageal) | Digestive | 0.98 | 0.88 | 1.11 | 0.80 | 320833 | 284 | 320549 | FALSE |
| 611.3 | Lump or mass in breast | Genitourinary | 0.99 | 0.95 | 1.04 | 0.80 | 322461 | 1588 | 320873 | FALSE |
| 368 | Visual disturbances | Sense Organs | 0.99 | 0.93 | 1.05 | 0.80 | 325963 | 1081 | 324882 | FALSE |
| 526 | Diseases of the jaws | Digestive | 1.00 | 0.99 | 1.02 | 0.80 | 327645 | 15987 | 311658 | FALSE |
| 483 | Acute bronchitis and bronchiolitis | Respiratory | 1.02 | 0.89 | 1.17 | 0.80 | 317889 | 200 | 317689 | FALSE |
| 726.1 | Enthesopathy | Musculoskeletal | 1.00 | 0.98 | 1.03 | 0.81 | 311657 | 6703 | 304954 | FALSE |
| 530.9 | Heartburn | Digestive | 0.99 | 0.95 | 1.04 | 0.81 | 289911 | 2108 | 287803 | FALSE |
| 366 | Cataract | Sense Organs | 1.00 | 0.98 | 1.01 | 0.81 | 323825 | 16441 | 307384 | FALSE |
| 695.9 | Unspecified erythematous condition | Dermatologic | 0.99 | 0.90 | 1.08 | 0.81 | 322226 | 477 | 321749 | FALSE |
| 537 | Other disorders of stomach and duodenum | Digestive | 1.00 | 0.96 | 1.03 | 0.81 | 299994 | 3483 | 296511 | FALSE |
| 174.1 | Breast cancer [female] | Neoplasms | 0.99 | 0.95 | 1.04 | 0.81 | 306711 | 2287 | 304424 | FALSE |
| 686 | Other local infections of skin and subcutaneous tissue | Dermatologic | 1.00 | 0.98 | 1.02 | 0.81 | 327410 | 11003 | 316407 | FALSE |
| 526.9 | Jaw disease NOS | Digestive | 1.00 | 0.99 | 1.02 | 0.81 | 327620 | 15962 | 311658 | FALSE |
| 386 | Vertiginous syndromes and other disorders of vestibular system | Sense Organs | 1.01 | 0.96 | 1.05 | 0.81 | 323761 | 2112 | 321649 | FALSE |
| 571.81 | Portal hypertension | Digestive | 1.01 | 0.93 | 1.10 | 0.82 | 318900 | 569 | 318331 | FALSE |
| 574.11 | Cholelithiasis with acute cholecystitis | Digestive | 0.99 | 0.94 | 1.05 | 0.82 | 311836 | 1513 | 310323 | FALSE |
| 54 | Herpes simplex | Infectious Diseases | 1.02 | 0.89 | 1.16 | 0.82 | 322425 | 219 | 322206 | FALSE |
| 198.3 | Secondary malignant neoplasm of digestive systems | Neoplasms | 1.01 | 0.96 | 1.06 | 0.82 | 230877 | 1565 | 229312 | FALSE |
| 198.5 | Secondary malignancy of brain/spine | Neoplasms | 0.99 | 0.93 | 1.06 | 0.82 | 230113 | 801 | 229312 | FALSE |
| 251.1 | Hypoglycemia | Endocrine/Metabolic | 0.99 | 0.93 | 1.06 | 0.82 | 305952 | 977 | 304975 | FALSE |

| | | | | | | | | | | |
|---|---|---|---|---|---|---|---|---|---|---|
| 587 | Kidney replaced by transpant | Genitourinary | 1.01 | 0.92 | 1.12 | 0.82 | 314477 | 398 | 314079 | FALSE |
| 804 | Fracture of hand or wrist | Injuries & Poisonings | 0.99 | 0.92 | 1.07 | 0.83 | 324232 | 675 | 323557 | FALSE |
| 577.1 | Acute pancreatitis | Digestive | 0.99 | 0.95 | 1.05 | 0.83 | 327437 | 1517 | 325920 | FALSE |
| 117 | Mycoses | Infectious Diseases | 1.00 | 0.97 | 1.04 | 0.83 | 328235 | 2735 | 325500 | FALSE |
| 204.21 | Myeloid leukemia, acute | Neoplasms | 1.01 | 0.91 | 1.13 | 0.83 | 323842 | 319 | 323523 | FALSE |
| 300.1 | Anxiety disorder | Mental Disorders | 1.00 | 0.97 | 1.02 | 0.83 | 288368 | 5470 | 282898 | FALSE |
| 800 | Fracture of lower limb | Injuries & Poisonings | 1.01 | 0.94 | 1.09 | 0.83 | 324256 | 699 | 323557 | FALSE |
| 165 | Cancer within the respiratory system | Neoplasms | 1.00 | 0.96 | 1.03 | 0.83 | 328223 | 2954 | 325269 | FALSE |
| 695.7 | Prurigo and Lichen | Dermatologic | 0.99 | 0.93 | 1.06 | 0.83 | 322541 | 792 | 321749 | FALSE |
| 585.31 | Renal dialysis | Genitourinary | 0.99 | 0.91 | 1.08 | 0.83 | 314603 | 524 | 314079 | FALSE |
| 216 | Benign neoplasm of skin | Neoplasms | 1.00 | 0.98 | 1.03 | 0.84 | 327614 | 7865 | 319749 | FALSE |
| 742.9 | Other derangement of joint | Musculoskeletal | 1.01 | 0.92 | 1.11 | 0.84 | 311042 | 400 | 310642 | FALSE |
| 578.9 | Hemorrhage of gastrointestinal tract | Digestive | 1.00 | 0.98 | 1.03 | 0.84 | 308849 | 5455 | 303394 | FALSE |
| 701 | Other hypertrophic and atrophic conditions of skin | Dermatologic | 1.00 | 0.95 | 1.04 | 0.84 | 324891 | 1827 | 323064 | FALSE |
| 496.1 | Emphysema | Respiratory | 1.00 | 0.96 | 1.05 | 0.84 | 295918 | 1726 | 294192 | FALSE |
| 577.2 | Chronic pancreatitis | Digestive | 0.99 | 0.91 | 1.08 | 0.84 | 326456 | 536 | 325920 | FALSE |
| 781 | Symptoms involving nervous and musculoskeletal systems | Symptoms | 1.00 | 0.99 | 1.02 | 0.85 | 328238 | 22180 | 306058 | FALSE |
| 345 | Epilepsy, recurrent seizures, convulsions | Neurological | 1.00 | 0.96 | 1.03 | 0.85 | 289613 | 3461 | 286152 | FALSE |
| 290 | Delirium dementia and amnestic and other cognitive disorders | Mental Disorders | 1.01 | 0.92 | 1.10 | 0.85 | 321950 | 480 | 321470 | FALSE |
| 250.22 | Type 2 diabetes with renal manifestations | Endocrine/Metabolic | 1.01 | 0.88 | 1.16 | 0.85 | 307698 | 208 | 307490 | FALSE |
| 737 | Curvature of spine | Musculoskeletal | 0.99 | 0.92 | 1.07 | 0.85 | 317430 | 743 | 316687 | FALSE |
| 339 | Other headache syndromes | Neurological | 1.00 | 0.98 | 1.02 | 0.86 | 325888 | 7938 | 317950 | FALSE |
| 509.2 | Respiratory insufficiency | Respiratory | 1.00 | 0.95 | 1.04 | 0.86 | 318272 | 1788 | 316484 | FALSE |
| 288 | Diseases of white blood cells | Hematopoietic | 0.99 | 0.92 | 1.08 | 0.86 | 320928 | 610 | 320318 | FALSE |
| 418 | Nonspecific chest pain | Circulatory System | 1.00 | 0.99 | 1.01 | 0.86 | 326032 | 30212 | 295820 | FALSE |
| 529.1 | Glossitis | Digestive | 0.99 | 0.88 | 1.11 | 0.86 | 322838 | 304 | 322534 | FALSE |
| 732 | Osteochondropathies | Musculoskeletal | 0.99 | 0.87 | 1.13 | 0.86 | 315651 | 219 | 315432 | FALSE |
| 747 | Cardiac and circulatory congenital anomalies | Congenital Anomalies | 1.00 | 0.95 | 1.06 | 0.87 | 326576 | 1222 | 325354 | FALSE |
| 427.3 | Other specified cardiac dysrhythmias | Circulatory System | 1.00 | 0.97 | 1.04 | 0.87 | 302836 | 3315 | 299521 | FALSE |
| 798.1 | Chronic fatigue syndrome | Symptoms | 1.01 | 0.93 | 1.09 | 0.87 | 325335 | 606 | 324729 | FALSE |
| 374.3 | Ptosis of eyelid | Sense Organs | 1.00 | 0.95 | 1.04 | 0.87 | 320284 | 1896 | 318388 | FALSE |
| 751.21 | Cystic kidney disease | Congenital Anomalies | 0.99 | 0.91 | 1.09 | 0.87 | 326461 | 458 | 326003 | FALSE |
| 281 | Other deficiency anemia | Hematopoietic | 1.00 | 0.98 | 1.02 | 0.87 | 317687 | 8787 | 308900 | FALSE |
| 580.32 | Nephritis and nephropathy with pathological lesion | Genitourinary | 1.00 | 0.94 | 1.06 | 0.87 | 315157 | 1078 | 314079 | FALSE |
| 464 | Acute sinusitis | Respiratory | 1.01 | 0.88 | 1.16 | 0.87 | 325387 | 208 | 325179 | FALSE |
| 481 | Influenza | Respiratory | 1.00 | 0.98 | 1.02 | 0.87 | 327706 | 10017 | 317689 | FALSE |
| 535.8 | Other specified gastritis | Digestive | 1.00 | 0.98 | 1.02 | 0.87 | 304926 | 8415 | 296511 | FALSE |
| 380.1 | Otitis externa | Sense Organs | 1.01 | 0.92 | 1.10 | 0.87 | 327284 | 532 | 326752 | FALSE |

| | | | | | | | | | | |
|---|---|---|---|---|---|---|---|---|---|---|
| 420.3 | Endocarditis | Circulatory System | 1.01 | 0.93 | 1.08 | 0.87 | 325668 | 685 | 324983 | FALSE |
| 280.1 | Iron deficiency anemias, unspecified or not due to blood loss | Hematopoietic | 1.00 | 0.98 | 1.02 | 0.87 | 316402 | 7502 | 308900 | FALSE |
| 556.1 | Ulceration of intestine | Digestive | 1.01 | 0.93 | 1.08 | 0.88 | 259087 | 691 | 258396 | FALSE |
| 218 | Benign neoplasm of uterus | Neoplasms | 0.99 | 0.90 | 1.10 | 0.88 | 308168 | 388 | 307780 | FALSE |
| 519.2 | Respiratory complications | Respiratory | 0.99 | 0.88 | 1.11 | 0.88 | 265743 | 296 | 265447 | FALSE |
| 411.1 | Unstable angina (intermediate coronary syndrome) | Circulatory System | 1.00 | 0.98 | 1.03 | 0.88 | 301011 | 5322 | 295689 | FALSE |
| 990 | Effects radiation NOS | Injuries & Poisonings | 1.00 | 0.97 | 1.04 | 0.88 | 325633 | 3244 | 322389 | FALSE |
| 569.2 | Gastrointestinal complications | Digestive | 0.99 | 0.91 | 1.09 | 0.88 | 247132 | 472 | 246660 | FALSE |
| 809 | Fracture of unspecified bones | Injuries & Poisonings | 0.99 | 0.90 | 1.09 | 0.88 | 323967 | 410 | 323557 | FALSE |
| 604.1 | Redundant prepuce and phimosis/BXO | Genitourinary | 1.00 | 0.96 | 1.04 | 0.88 | 310716 | 2810 | 307906 | FALSE |
| 351 | Other peripheral nerve disorders | Neurological | 1.00 | 0.98 | 1.02 | 0.88 | 325903 | 12892 | 313011 | FALSE |
| 738.4 | Acquired spondylolisthesis | Musculoskeletal | 1.01 | 0.89 | 1.14 | 0.89 | 316942 | 255 | 316687 | FALSE |
| 470 | Septal Deviations/Turbinate Hypertrophy | Respiratory | 1.00 | 0.97 | 1.03 | 0.89 | 313771 | 4821 | 308950 | FALSE |
| 332 | Parkinson's disease | Neurological | 1.00 | 0.95 | 1.06 | 0.89 | 287334 | 1182 | 286152 | FALSE |
| 575.1 | Cholangitis | Digestive | 1.01 | 0.92 | 1.10 | 0.89 | 310820 | 497 | 310323 | FALSE |
| 610 | Benign mammary dysplasias | Genitourinary | 1.01 | 0.88 | 1.16 | 0.89 | 321081 | 208 | 320873 | FALSE |
| 742.8 | Articular cartilage disorder | Musculoskeletal | 1.01 | 0.93 | 1.09 | 0.89 | 311297 | 655 | 310642 | FALSE |
| 555.21 | Ulcerative colitis (chronic) | Digestive | 0.99 | 0.92 | 1.08 | 0.90 | 258967 | 571 | 258396 | FALSE |
| 241.1 | Nontoxic uninodular goiter | Endocrine/Metabolic | 1.01 | 0.92 | 1.10 | 0.90 | 310840 | 511 | 310329 | FALSE |
| 197 | Chemotherapy | Neoplasms | 1.00 | 0.99 | 1.01 | 0.90 | 251774 | 22462 | 229312 | FALSE |
| 198 | Secondary malignant neoplasm | Neoplasms | 1.00 | 0.94 | 1.06 | 0.90 | 230431 | 1119 | 229312 | FALSE |
| 782.3 | Edema | Symptoms | 1.00 | 0.96 | 1.05 | 0.90 | 328067 | 1715 | 326352 | FALSE |
| 41.1 | Staphylococcus infections | Infectious Diseases | 1.00 | 0.97 | 1.04 | 0.90 | 315832 | 3200 | 312632 | FALSE |
| 965.2 | Antirheumatics causing adverse effects in therapeutic use | Injuries & Poisonings | 0.99 | 0.91 | 1.09 | 0.91 | 300710 | 499 | 300211 | FALSE |
| 626.2 | Dysmenorrhea | Genitourinary | 1.00 | 0.96 | 1.05 | 0.91 | 297910 | 1679 | 296231 | FALSE |
| 565 | Anal and rectal conditions | Digestive | 1.00 | 0.98 | 1.02 | 0.91 | 255321 | 8661 | 246660 | FALSE |
| 204.4 | Multiple myeloma | Neoplasms | 1.00 | 0.93 | 1.09 | 0.91 | 324097 | 574 | 323523 | FALSE |
| 289.8 | Polycythemia vera, secondary | Hematopoietic | 0.99 | 0.89 | 1.12 | 0.91 | 320039 | 291 | 319748 | FALSE |
| 599 | Other symptoms/disorders or the urinary system | Genitourinary | 1.00 | 0.99 | 1.01 | 0.91 | 324256 | 103829 | 220427 | FALSE |
| 285.1 | Acute posthemorrhagic anemia | Hematopoietic | 0.99 | 0.88 | 1.12 | 0.91 | 309167 | 267 | 308900 | FALSE |
| 747.1 | Cardiac congenital anomalies | Congenital Anomalies | 1.01 | 0.88 | 1.15 | 0.91 | 325565 | 211 | 325354 | FALSE |
| 315 | Develomental delays and disorders | Mental Disorders | 1.00 | 0.92 | 1.08 | 0.92 | 326992 | 549 | 326443 | FALSE |
| 573.9 | Abnormal serum enzyme levels | Digestive | 0.99 | 0.87 | 1.13 | 0.92 | 318561 | 230 | 318331 | FALSE |
| 250.6 | Polyneuropathy in diabetes | Endocrine/Metabolic | 1.01 | 0.91 | 1.11 | 0.92 | 307871 | 381 | 307490 | FALSE |
| 535 | Gastritis and duodenitis | Digestive | 1.00 | 0.98 | 1.01 | 0.92 | 314259 | 17748 | 296511 | FALSE |
| 707.1 | Decubitus ulcer | Dermatologic | 0.99 | 0.89 | 1.11 | 0.92 | 327025 | 326 | 326699 | FALSE |
| 947 | Urticaria | Injuries & Poisonings | 0.99 | 0.90 | 1.10 | 0.92 | 322293 | 387 | 321906 | FALSE |
| 149 | Cancer of larynx, pharynx, nasal cavities | Neoplasms | 1.00 | 0.94 | 1.07 | 0.92 | 326986 | 945 | 326041 | FALSE |

| | | | | | | | | | | |
|---|---|---|---|---|---|---|---|---|---|---|
| 540.11 | Acute appendicitis | Digestive | 1.00 | 0.96 | 1.04 | 0.92 | 327037 | 2658 | 324379 | FALSE |
| 292.6 | Hallucinations | Mental Disorders | 0.99 | 0.89 | 1.11 | 0.92 | 321793 | 323 | 321470 | FALSE |
| 636.2 | Early onset of delivery | Pregnancy Complications | 1.00 | 0.92 | 1.08 | 0.92 | 320594 | 629 | 319965 | FALSE |
| 596 | Other disorders of bladder | Genitourinary | 1.00 | 0.98 | 1.02 | 0.92 | 321385 | 7749 | 313636 | FALSE |
| 496.2 | Chronic bronchitis | Respiratory | 1.01 | 0.90 | 1.13 | 0.93 | 294485 | 293 | 294192 | FALSE |
| 420.21 | Acute pericarditis | Circulatory System | 0.99 | 0.87 | 1.13 | 0.93 | 325211 | 228 | 324983 | FALSE |
| 448 | Disease of capillaries | Circulatory System | 1.00 | 0.98 | 1.02 | 0.93 | 326841 | 7481 | 319360 | FALSE |
| 614.3 | Pelvic inflammatory disease (PID) | Genitourinary | 0.99 | 0.88 | 1.13 | 0.93 | 319048 | 249 | 318799 | FALSE |
| 766 | Neuralgia, neuritis, and radiculitis NOS | Symptoms | 1.01 | 0.90 | 1.13 | 0.93 | 326987 | 304 | 326683 | FALSE |
| 599.5 | Frequency of urination and polyuria | Genitourinary | 1.00 | 0.97 | 1.03 | 0.93 | 224574 | 4147 | 220427 | FALSE |
| 735.23 | Hallux rigidus | Musculoskeletal | 1.00 | 0.95 | 1.05 | 0.93 | 318243 | 1556 | 316687 | FALSE |
| 204.12 | Lymphoid leukemia, chronic | Neoplasms | 1.00 | 0.92 | 1.10 | 0.93 | 324030 | 507 | 323523 | FALSE |
| 443.9 | Peripheral vascular disease, unspecified | Circulatory System | 1.00 | 0.96 | 1.04 | 0.93 | 321969 | 2609 | 319360 | FALSE |
| 289 | Other diseases of blood and blood-forming organs | Hematopoietic | 1.00 | 0.97 | 1.03 | 0.94 | 325300 | 4982 | 320318 | FALSE |
| 198.2 | Secondary malignancy of respiratory organs | Neoplasms | 1.00 | 0.96 | 1.04 | 0.94 | 231589 | 2277 | 229312 | FALSE |
| 527.2 | Sialoadenitis | Digestive | 1.00 | 0.91 | 1.11 | 0.94 | 322908 | 374 | 322534 | FALSE |
| 479 | Other upper respiratory disease | Respiratory | 1.00 | 0.98 | 1.02 | 0.94 | 324241 | 15291 | 308950 | FALSE |
| 626.13 | Irregular menstrual cycle | Genitourinary | 1.00 | 0.96 | 1.05 | 0.94 | 298189 | 1958 | 296231 | FALSE |
| 603 | Other disorders of testis | Genitourinary | 1.00 | 0.94 | 1.06 | 0.94 | 308895 | 989 | 307906 | FALSE |
| 300 | Anxiety, phobic and dissociative disorders | Mental Disorders | 1.00 | 0.90 | 1.10 | 0.95 | 283261 | 363 | 282898 | FALSE |
| 320 | Meningitis | Neurological | 1.00 | 0.91 | 1.09 | 0.95 | 327577 | 439 | 327138 | FALSE |
| 288.11 | Neutropenia | Hematopoietic | 1.00 | 0.97 | 1.03 | 0.95 | 323644 | 3326 | 320318 | FALSE |
| 966 | Poisoning by anticonvulsants and anti-Parkinsonism drugs | Injuries & Poisonings | 1.00 | 0.92 | 1.09 | 0.95 | 300747 | 536 | 300211 | FALSE |
| 594.8 | Renal colic | Genitourinary | 1.00 | 0.96 | 1.05 | 0.95 | 322062 | 2131 | 319931 | FALSE |
| 331.9 | Cerebral degeneration, unspecified | Neurological | 1.00 | 0.91 | 1.10 | 0.95 | 286569 | 417 | 286152 | FALSE |
| 571 | Chronic liver disease and cirrhosis | Digestive | 1.00 | 0.92 | 1.09 | 0.95 | 318871 | 540 | 318331 | FALSE |
| 252.1 | Hyperparathyroidism | Endocrine/Metabolic | 1.00 | 0.93 | 1.07 | 0.95 | 325359 | 814 | 324545 | FALSE |
| 733 | Other disorders of bone and cartilage | Musculoskeletal | 1.00 | 0.92 | 1.08 | 0.95 | 316001 | 569 | 315432 | FALSE |
| 524.3 | Anomalies of tooth position/malocclusion | Digestive | 1.00 | 0.89 | 1.13 | 0.95 | 311953 | 295 | 311658 | FALSE |
| 530.3 | Stricture and stenosis of esophagus | Digestive | 1.00 | 0.96 | 1.04 | 0.95 | 289757 | 1954 | 287803 | FALSE |
| 471 | Nasal polyps | Respiratory | 1.00 | 0.97 | 1.03 | 0.96 | 312342 | 3392 | 308950 | FALSE |
| 198.4 | Secondary malignant neoplasm of liver | Neoplasms | 1.00 | 0.96 | 1.04 | 0.96 | 232009 | 2697 | 229312 | FALSE |
| 295.1 | Schizophrenia | Mental Disorders | 1.00 | 0.92 | 1.08 | 0.96 | 283509 | 611 | 282898 | FALSE |
| 349 | Other and unspecified disorders of the nervous system | Neurological | 1.00 | 0.99 | 1.01 | 0.96 | 326855 | 40703 | 286152 | FALSE |
| 599.2 | Retention of urine | Genitourinary | 1.00 | 0.98 | 1.03 | 0.96 | 227284 | 6857 | 220427 | FALSE |
| 619.5 | Noninflammatory disorders of vulva and perineum | Genitourinary | 1.00 | 0.95 | 1.06 | 0.96 | 279927 | 1379 | 278548 | FALSE |
| 783 | Fever of unknown origin | Symptoms | 1.00 | 0.97 | 1.03 | 0.96 | 328240 | 4111 | 324129 | FALSE |
| 529 | Diseases and other conditions of the tongue | Digestive | 1.00 | 0.94 | 1.06 | 0.96 | 323485 | 951 | 322534 | FALSE |

| | | | | | | | | | | |
|---|---|---|---|---|---|---|---|---|---|---|
| 521.1 | Dental caries | Digestive | 1.00 | 0.97 | 1.04 | 0.96 | 314912 | 3254 | 311658 | FALSE |
| 687.4 | Disturbance of skin sensation | Dermatologic | 1.00 | 0.96 | 1.04 | 0.96 | 325793 | 2979 | 322814 | FALSE |
| 41.2 | Streptococcus infection | Infectious Diseases | 1.00 | 0.95 | 1.05 | 0.96 | 314280 | 1648 | 312632 | FALSE |
| 317.11 | Alcoholic liver damage | Mental Disorders | 1.00 | 0.94 | 1.07 | 0.96 | 298534 | 865 | 297669 | FALSE |
| 287.31 | Primary thrombocytopenia | Hematopoietic | 1.00 | 0.91 | 1.10 | 0.97 | 325925 | 428 | 325497 | FALSE |
| 8.52 | Intestinal infection due to C. difficile | Infectious Diseases | 1.00 | 0.93 | 1.08 | 0.97 | 319727 | 648 | 319079 | FALSE |
| 535.1 | Acute gastritis | Digestive | 1.00 | 0.94 | 1.06 | 0.97 | 297704 | 1193 | 296511 | FALSE |
| 706.2 | Sebaceous cyst | Dermatologic | 1.00 | 0.98 | 1.02 | 0.97 | 327401 | 9107 | 318294 | FALSE |
| 365.2 | Primary angle-closure glaucoma | Sense Organs | 1.00 | 0.93 | 1.08 | 0.97 | 317491 | 709 | 316782 | FALSE |
| 350.1 | Abnormal involuntary movements | Neurological | 1.00 | 0.94 | 1.07 | 0.98 | 326371 | 927 | 325444 | FALSE |
| 229 | Benign neoplasm of unspecified sites | Neoplasms | 1.00 | 0.99 | 1.01 | 0.98 | 328240 | 46165 | 282075 | FALSE |
| 228 | Hemangioma and lymphangioma, any site | Neoplasms | 1.00 | 0.95 | 1.05 | 0.98 | 328240 | 1635 | 326605 | FALSE |
| 318 | Tobacco use disorder | Mental Disorders | 1.00 | 0.99 | 1.01 | 0.98 | 318172 | 20503 | 297669 | FALSE |
| 474.2 | Chronic tonsillitis and adenoiditis | Respiratory | 1.00 | 0.94 | 1.06 | 0.98 | 310135 | 1185 | 308950 | FALSE |
| 8 | Intestinal infection | Infectious Diseases | 1.00 | 0.98 | 1.02 | 0.98 | 328240 | 9161 | 319079 | FALSE |
| 571.8 | Liver abscess and sequelae of chronic liver disease | Digestive | 1.00 | 0.92 | 1.08 | 0.98 | 318904 | 573 | 318331 | FALSE |
| 592.12 | Chronic cystitis | Genitourinary | 1.00 | 0.94 | 1.07 | 0.98 | 297948 | 913 | 297035 | FALSE |
| 378.2 | Nystagmus and other irregular eye movements | Sense Organs | 1.00 | 0.88 | 1.15 | 0.98 | 288407 | 215 | 288192 | FALSE |
| 870.3 | Other open wound of head and face | Injuries & Poisonings | 1.00 | 0.96 | 1.04 | 0.98 | 322782 | 3051 | 319731 | FALSE |
| 80 | Postoperative infection | Infectious Diseases | 1.00 | 0.97 | 1.03 | 0.98 | 326101 | 4622 | 321479 | FALSE |
| 454 | Varicose veins | Circulatory System | 1.00 | 0.95 | 1.05 | 0.99 | 289316 | 1603 | 287713 | FALSE |
| 623 | Hypertrophy of female genital organs | Genitourinary | 1.00 | 0.95 | 1.06 | 0.99 | 316579 | 1272 | 315307 | FALSE |
| 495 | Asthma | Respiratory | 1.00 | 0.99 | 1.01 | 0.99 | 320971 | 26779 | 294192 | FALSE |
| 472 | Chronic pharyngitis and nasopharyngitis | Respiratory | 1.00 | 0.94 | 1.07 | 0.99 | 309897 | 947 | 308950 | FALSE |
| 241.2 | Nontoxic multinodular goiter | Endocrine/Metabolic | 1.00 | 0.93 | 1.08 | 0.99 | 311043 | 714 | 310329 | FALSE |
| 628 | Ovarian cyst | Genitourinary | 1.00 | 0.97 | 1.03 | 0.99 | 301051 | 4820 | 296231 | FALSE |
| 681.1 | Cellulitis and abscess of fingers/toes | Dermatologic | 1.00 | 0.92 | 1.08 | 0.99 | 316989 | 582 | 316407 | FALSE |
| 721.8 | Other allied disorders of spine | Musculoskeletal | 1.00 | 0.97 | 1.04 | 0.99 | 314171 | 3227 | 310944 | FALSE |
| 721 | Spondylosis and allied disorders | Musculoskeletal | 1.00 | 0.94 | 1.07 | 0.99 | 311869 | 925 | 310944 | FALSE |
| 686.4 | Pyogenic granuloma | Dermatologic | 1.00 | 0.90 | 1.11 | 0.99 | 316787 | 380 | 316407 | FALSE |
| 175 | Acquired absence of breast | Neoplasms | 1.00 | 0.96 | 1.04 | 0.99 | 305907 | 2516 | 303391 | FALSE |
| 366.2 | Senile cataract | Sense Organs | 1.00 | 0.98 | 1.02 | 0.99 | 315842 | 8458 | 307384 | FALSE |
| 610.1 | Cystic mastopathy | Genitourinary | 1.00 | 0.94 | 1.07 | 0.99 | 321832 | 959 | 320873 | FALSE |
| 619 | Noninflammatory female genital disorders | Genitourinary | 1.00 | 0.99 | 1.01 | 1.00 | 328106 | 49558 | 278548 | FALSE |
| 605 | Erectile dysfunction [ED] | Genitourinary | 1.00 | 0.89 | 1.12 | 1.00 | 308197 | 291 | 307906 | FALSE |
| 473.4 | Voice disturbance | Respiratory | 1.00 | 0.94 | 1.06 | 1.00 | 310051 | 1101 | 308950 | FALSE |
| 530.11 | GERD | Digestive | 1.00 | 0.98 | 1.02 | 1.00 | 302292 | 14489 | 287803 | FALSE |

| Supplementary Table 13. Phenome-wide association study (PheWAS) results for the beta-blocker (BB) genetic risk score when using single-nucleotide polymorphisms from the systolic blood pressure genome-wide association study that did not correct for antihypertensive medication use or adjust for body mass index. | | | | | | | | |
|---|---|---|---|---|---|---|---|---|
| Phecode | Trait | Category | OR | Low 95% CI | Upp 95% CI | Total sample size | Cases | Controls |
| 401 | Hypertension | Circulatory System | 0.96 | 0.95 | 0.97 | 328239 | 79491 | 248748 |
| 401.1 | Essential hypertension | Circulatory System | 0.96 | 0.95 | 0.97 | 327983 | 79235 | 248748 |
| 459.9 | Circulatory disease NEC | Circulatory System | 0.98 | 0.97 | 0.99 | 327928 | 133749 | 194179 |
| 189.21 | Malignant neoplasm of bladder | Neoplasms | 0.92 | 0.88 | 0.96 | 326150 | 2188 | 323962 |
| 747.12 | Valvular heart disease/ heart chambers | Congenital Anomalies | 0.81 | 0.70 | 0.93 | 325592 | 238 | 325354 |
| 289.3 | Personal history of diseases of blood and blood-forming organs | Hematopoietic | 0.83 | 0.74 | 0.94 | 320615 | 297 | 320318 |
| 747.13 | Congenital anomalies of great vessels | Congenital Anomalies | 0.93 | 0.89 | 0.98 | 327177 | 1823 | 325354 |
| 747 | Cardiac and circulatory congenital anomalies | Congenital Anomalies | 0.92 | 0.86 | 0.97 | 326576 | 1222 | 325354 |
| 427 | Cardiac dysrhythmias | Circulatory System | 0.85 | 0.75 | 0.95 | 299844 | 323 | 299521 |
| 530.3 | Stricture and stenosis of esophagus | Digestive | 1.06 | 1.02 | 1.11 | 289757 | 1954 | 287803 |
| 574.2 | Calculus of bile duct | Digestive | 1.05 | 1.01 | 1.09 | 313011 | 2688 | 310323 |
| 112 | Candidiasis | Infectious Diseases | 0.94 | 0.90 | 0.98 | 327650 | 2150 | 325500 |
| 395.6 | Heart valve replaced | Circulatory System | 0.93 | 0.88 | 0.98 | 323056 | 1528 | 321528 |
| 90 | Sexually transmitted infections (not HIV or hepatitis) | Infectious Diseases | 0.85 | 0.75 | 0.96 | 328239 | 298 | 327941 |
| 427.2 | Atrial fibrillation and flutter | Circulatory System | 0.98 | 0.96 | 0.99 | 314573 | 15052 | 299521 |
| 189.4 | Malignant neoplasm of other urinary organs | Neoplasms | 0.95 | 0.92 | 0.99 | 327185 | 3223 | 323962 |
| 117 | Mycoses | Infectious Diseases | 0.95 | 0.91 | 0.99 | 328235 | 2735 | 325500 |
| 857 | Mechanical complication of unspecified genitourinary device, implant, and graft | Injuries & Poisonings | 0.93 | 0.88 | 0.98 | 315248 | 1284 | 313964 |
| 362.4 | Retinal vascular changes and abnomalities | Sense Organs | 1.09 | 1.02 | 1.16 | 316733 | 881 | 315852 |
| 182 | Malignant neoplasm of uterus | Neoplasms | 0.93 | 0.88 | 0.99 | 298948 | 1303 | 297645 |
| 850 | Hemorrhage or hematoma complicating a procedure | Injuries & Poisonings | 0.97 | 0.94 | 0.99 | 319328 | 5364 | 313964 |
| 556 | Ulceration of the lower GI tract | Digestive | 0.89 | 0.80 | 0.98 | 258836 | 440 | 258396 |
| 535 | Gastritis and duodenitis | Digestive | 1.02 | 1.00 | 1.03 | 314259 | 17748 | 296511 |
| 509.8 | Dependence on respirator [Ventilator] or supplemental oxygen | Respiratory | 1.12 | 1.02 | 1.24 | 316885 | 401 | 316484 |
| 575.1 | Cholangitis | Digestive | 1.11 | 1.02 | 1.21 | 310820 | 497 | 310323 |
| 184.11 | Malignant neoplasm of ovary | Neoplasms | 0.97 | 0.94 | 0.99 | 310562 | 5009 | 305553 |
| 362 | Other retinal disorders | Sense Organs | 1.08 | 1.01 | 1.15 | 316825 | 973 | 315852 |
| 261.4 | Vitamin D deficiency | Endocrine/Metabolic | 0.88 | 0.80 | 0.98 | 326194 | 408 | 325786 |
| 250.1 | Type 1 diabetes | Endocrine/Metabolic | 1.05 | 1.01 | 1.09 | 310189 | 2699 | 307490 |
| 184.1 | Malignant neoplasm of ovary and other uterine adnexa | Neoplasms | 0.95 | 0.92 | 0.99 | 308106 | 2553 | 305553 |
| 426.21 | First degree AV block | Circulatory System | 0.93 | 0.87 | 0.99 | 300526 | 1005 | 299521 |
| 411 | Ischemic Heart Disease | Circulatory System | 0.99 | 0.97 | 1.00 | 327032 | 31343 | 295689 |
| 411.8 | Other chronic ischemic heart disease, unspecified | Circulatory System | 0.99 | 0.97 | 1.00 | 326969 | 31280 | 295689 |
| 555.21 | Ulcerative colitis (chronic) | Digestive | 1.10 | 1.01 | 1.19 | 258967 | 571 | 258396 |
| 226 | Benign neoplasm of thyroid glands | Neoplasms | 1.15 | 1.02 | 1.29 | 326908 | 266 | 326642 |

| | | | | | | | | |
|---|---|---|---|---|---|---|---|---|
| 450 | Noninfectious disorders of lymphatic channels | Circulatory System | 0.92 | 0.85 | 0.99 | 328240 | 717 | 327523 |
| 733 | Other disorders of bone and cartilage | Musculoskeletal | 1.10 | 1.01 | 1.19 | 316001 | 569 | 315432 |
| 70 | Viral hepatitis | Infectious Diseases | 0.92 | 0.85 | 0.99 | 322928 | 722 | 322206 |
| 187.2 | Malignant neoplasm of testis | Neoplasms | 0.97 | 0.94 | 1.00 | 323790 | 4572 | 319218 |
| 411.4 | Coronary atherosclerosis | Circulatory System | 0.99 | 0.97 | 1.00 | 327242 | 31553 | 295689 |
| 577 | Diseases of pancreas | Digestive | 0.91 | 0.83 | 0.99 | 326478 | 558 | 325920 |
| 293 | Symptoms involving head and neck | Mental Disorders | 1.05 | 1.00 | 1.09 | 327323 | 2204 | 325119 |
| 696.42 | Psoriatic arthropathy | Dermatologic | 0.92 | 0.85 | 0.99 | 314672 | 740 | 313932 |
| 180.1 | Cervical cancer | Neoplasms | 0.97 | 0.94 | 1.00 | 302137 | 4554 | 297583 |
| 738.4 | Acquired spondylolisthesis | Musculoskeletal | 1.14 | 1.01 | 1.28 | 316942 | 255 | 316687 |
| 597.1 | Urethral stricture (not specified as infectious) | Genitourinary | 0.96 | 0.93 | 1.00 | 317079 | 3443 | 313636 |
| 451.2 | Phlebitis and thrombophlebitis of lower extremities | Circulatory System | 0.96 | 0.93 | 1.00 | 291357 | 3644 | 287713 |
| 383 | Otosclerosis | Sense Organs | 0.88 | 0.79 | 0.99 | 324473 | 330 | 324143 |
| 747.11 | Cardiac shunt/ heart septal defect | Congenital Anomalies | 0.91 | 0.84 | 0.99 | 325935 | 581 | 325354 |
| 550.3 | Femoral hernia | Digestive | 0.92 | 0.85 | 0.99 | 280442 | 653 | 279789 |
| 250.41 | Impaired fasting glucose | Endocrine/Metabolic | 1.13 | 1.01 | 1.27 | 307758 | 268 | 307490 |
| 585.2 | Renal failure NOS | Genitourinary | 1.02 | 1.00 | 1.05 | 322824 | 8745 | 314079 |
| 870.1 | Open wound or laceration of eye or eyelid | Injuries & Poisonings | 0.88 | 0.78 | 0.99 | 320012 | 281 | 319731 |
| 374.3 | Ptosis of eyelid | Sense Organs | 1.05 | 1.00 | 1.10 | 320284 | 1896 | 318388 |
| 285 | Other anemias | Hematopoietic | 1.02 | 1.00 | 1.04 | 320744 | 11844 | 308900 |
| 414 | Other forms of chronic heart disease | Circulatory System | 0.95 | 0.91 | 1.00 | 297457 | 1768 | 295689 |
| 550.4 | Umbilical hernia | Digestive | 0.97 | 0.94 | 1.00 | 283693 | 3904 | 279789 |
| 522.5 | Periapical abscess | Digestive | 1.06 | 1.00 | 1.12 | 312904 | 1246 | 311658 |
| 191.11 | Cancer of brain | Neoplasms | 0.91 | 0.83 | 1.00 | 326945 | 498 | 326447 |
| 348.7 | Coma | Neurological | 0.90 | 0.81 | 1.00 | 286531 | 379 | 286152 |
| 260.6 | Anorexia | Endocrine/Metabolic | 0.93 | 0.87 | 1.00 | 326636 | 850 | 325786 |
| 149.4 | Cancer of larynx | Neoplasms | 0.88 | 0.78 | 1.00 | 326307 | 266 | 326041 |
| 636.3 | Hemorrhage in early pregnancy | Pregnancy Complications | 0.94 | 0.88 | 1.00 | 321078 | 1113 | 319965 |
| 426.9 | Cardiac pacemaker/device in situ | Circulatory System | 1.13 | 1.00 | 1.28 | 299758 | 237 | 299521 |
| 151 | Cancer of stomach | Neoplasms | 0.92 | 0.84 | 1.00 | 312905 | 574 | 312331 |
| 350.3 | Lack of coordination | Neurological | 0.90 | 0.81 | 1.00 | 325804 | 360 | 325444 |
| 804 | Fracture of hand or wrist | Injuries & Poisonings | 1.08 | 1.00 | 1.16 | 324232 | 675 | 323557 |
| 966 | Poisoning by anticonvulsants and anti-Parkinsonism drugs | Injuries & Poisonings | 1.09 | 1.00 | 1.18 | 300747 | 536 | 300211 |
| 250.2 | Type 2 diabetes | Endocrine/Metabolic | 1.01 | 1.00 | 1.03 | 326603 | 19113 | 307490 |
| 611.3 | Lump or mass in breast | Genitourinary | 1.05 | 1.00 | 1.10 | 322461 | 1588 | 320873 |
| 916 | Contusion | Injuries & Poisonings | 0.95 | 0.90 | 1.00 | 328240 | 1478 | 326762 |
| 386.1 | Meniere's disease | Sense Organs | 1.08 | 1.00 | 1.17 | 322213 | 564 | 321649 |
| 530.7 | Gastroesophageal laceration-hemorrhage syndrome | Digestive | 1.11 | 1.00 | 1.23 | 288136 | 333 | 287803 |

| | | | | | | | | | |
|---|---|---|---|---|---|---|---|---|---|
| 185 | Cancer of prostate | Neoplasms | 0.98 | 0.96 | 1.00 | 315856 | 8463 | 307393 |
| 377 | Disorders of optic nerve and visual pathways | Sense Organs | 0.91 | 0.83 | 1.00 | 288628 | 436 | 288192 |
| 395.1 | Nonrheumatic mitral valve disorders | Circulatory System | 0.97 | 0.93 | 1.00 | 324497 | 2969 | 321528 |
| 301 | Personality disorders | Mental Disorders | 1.09 | 1.00 | 1.20 | 283330 | 432 | 282898 |
| 594.1 | Calculus of kidney | Genitourinary | 0.97 | 0.93 | 1.00 | 323265 | 3334 | 319931 |
| 180.3 | Cervical intraepithelial neoplasia [CIN] [Cervical dysplasia] | Neoplasms | 0.96 | 0.92 | 1.00 | 299736 | 2153 | 297583 |
| 766 | Neuralgia, neuritis, and radiculitis NOS | Symptoms | 1.11 | 0.99 | 1.24 | 326987 | 304 | 326683 |
| 472 | Chronic pharyngitis and nasopharyngitis | Respiratory | 1.06 | 1.00 | 1.13 | 309897 | 947 | 308950 |
| 634.3 | Ectopic pregnancy | Pregnancy Complications | 0.90 | 0.81 | 1.01 | 320307 | 342 | 319965 |
| 256.4 | Polycystic ovaries | Endocrine/Metabolic | 1.13 | 0.99 | 1.29 | 324764 | 219 | 324545 |
| 603 | Other disorders of testis | Genitourinary | 0.94 | 0.88 | 1.00 | 308895 | 989 | 307906 |
| 272.11 | Hypercholesterolemia | Endocrine/Metabolic | 0.99 | 0.98 | 1.00 | 325499 | 34002 | 291497 |
| 241.2 | Nontoxic multinodular goiter | Endocrine/Metabolic | 0.93 | 0.86 | 1.00 | 311043 | 714 | 310329 |
| 480.5 | Bronchopneumonia and lung abscess | Respiratory | 0.91 | 0.82 | 1.01 | 318077 | 388 | 317689 |
| 369 | Infection of the eye | Sense Organs | 1.12 | 0.99 | 1.26 | 318642 | 254 | 318388 |
| 250 | Diabetes mellitus | Endocrine/Metabolic | 1.01 | 1.00 | 1.03 | 327730 | 20240 | 307490 |
| 172.3 | Carcinoma in situ of skin | Neoplasms | 1.07 | 0.99 | 1.15 | 314884 | 664 | 314220 |
| 565 | Anal and rectal conditions | Digestive | 1.02 | 1.00 | 1.04 | 255321 | 8661 | 246660 |
| 292.3 | Memory loss | Mental Disorders | 1.07 | 0.99 | 1.15 | 322208 | 738 | 321470 |
| 394.3 | Aortic valve disease | Circulatory System | 0.95 | 0.90 | 1.00 | 322836 | 1308 | 321528 |
| 394.2 | Mitral valve disease | Circulatory System | 0.97 | 0.93 | 1.00 | 324603 | 3075 | 321528 |
| 604 | Disorders of penis | Genitourinary | 0.93 | 0.87 | 1.01 | 308641 | 735 | 307906 |
| 599.5 | Frequency of urination and polyuria | Genitourinary | 0.97 | 0.94 | 1.00 | 224574 | 4147 | 220427 |
| 516.1 | Hemoptysis | Respiratory | 1.04 | 1.00 | 1.08 | 328154 | 2103 | 326051 |
| 989 | Toxic effect of other substances, chiefly nonmedicinal as to source | Injuries & Poisonings | 1.05 | 1.00 | 1.10 | 328136 | 1438 | 326698 |
| 380 | Disorders of external ear | Sense Organs | 0.95 | 0.91 | 1.00 | 328227 | 1475 | 326752 |
| 362.29 | Macular degeneration (senile) of retina NOS | Sense Organs | 1.04 | 1.00 | 1.08 | 318052 | 2200 | 315852 |
| 574.3 | Cholecystitis without cholelithiasis | Digestive | 0.97 | 0.93 | 1.00 | 313141 | 2818 | 310323 |
| 187.1 | Malignant neoplasm of unspecified male genital organ | Neoplasms | 0.98 | 0.96 | 1.00 | 327699 | 8481 | 319218 |
| 514 | Abnormal findings examination of lungs | Respiratory | 0.97 | 0.93 | 1.00 | 328212 | 2577 | 325635 |
| 430.2 | Intracerebral hemorrhage | Circulatory System | 0.94 | 0.87 | 1.01 | 318830 | 723 | 318107 |
| 742.8 | Articular cartilage disorder | Musculoskeletal | 0.93 | 0.86 | 1.01 | 311297 | 655 | 310642 |
| 427.9 | Palpitations | Circulatory System | 0.97 | 0.94 | 1.00 | 303480 | 3959 | 299521 |
| 362.2 | Degeneration of macula and posterior pole of retina | Sense Organs | 1.04 | 1.00 | 1.08 | 318055 | 2203 | 315852 |
| 476 | Allergic rhinitis | Respiratory | 0.95 | 0.89 | 1.01 | 310025 | 1075 | 308950 |
| 381.11 | Suppurative and unspecified otitis media | Sense Organs | 0.94 | 0.88 | 1.01 | 324993 | 850 | 324143 |
| 764 | Sciatica | Symptoms | 0.95 | 0.90 | 1.01 | 327944 | 1261 | 326683 |
| 189.2 | Cancer of bladder | Neoplasms | 0.94 | 0.88 | 1.01 | 324798 | 836 | 323962 |

| | | | | | | | | |
|---|---|---|---|---|---|---|---|---|
| 681.7 | Cellulitis and abscess of trunk | Dermatologic | 0.93 | 0.86 | 1.01 | 317030 | 623 | 316407 |
| 331.9 | Cerebral degeneration, unspecified | Neurological | 1.09 | 0.99 | 1.19 | 286569 | 417 | 286152 |
| 420.21 | Acute pericarditis | Circulatory System | 0.89 | 0.78 | 1.02 | 325211 | 228 | 324983 |
| 381.9 | Otorrhea | Sense Organs | 0.90 | 0.80 | 1.02 | 324420 | 277 | 324143 |
| 442.11 | Abdominal aortic aneurysm | Circulatory System | 1.06 | 0.99 | 1.13 | 320257 | 897 | 319360 |
| 613.1 | Inflammatory disease of breast | Genitourinary | 1.06 | 0.99 | 1.14 | 323156 | 738 | 322418 |
| 759 | Other and unspecified congenital anomalies | Congenital Anomalies | 0.94 | 0.87 | 1.01 | 327249 | 679 | 326570 |
| 303.3 | Psychogenic disorder | Mental Disorders | 1.06 | 0.99 | 1.13 | 283739 | 841 | 282898 |
| 585.1 | Acute renal failure | Genitourinary | 1.03 | 1.00 | 1.06 | 318744 | 4665 | 314079 |
| 596.1 | Bladder neck obstruction | Genitourinary | 0.96 | 0.92 | 1.01 | 315665 | 2029 | 313636 |
| 726.1 | Enthesopathy | Musculoskeletal | 0.98 | 0.96 | 1.00 | 311657 | 6703 | 304954 |
| 344 | Other paralytic syndromes | Neurological | 0.94 | 0.87 | 1.01 | 286841 | 689 | 286152 |
| 195.1 | Malignant neoplasm, other | Neoplasms | 0.99 | 0.99 | 1.00 | 320138 | 90826 | 229312 |
| 227.3 | Benign neoplasm of pituitary gland and craniopharyngeal duct (pouch) | Neoplasms | 0.91 | 0.81 | 1.02 | 326970 | 328 | 326642 |
| 614.4 | Inflammatory diseases of uterus, except cervix | Genitourinary | 0.91 | 0.81 | 1.02 | 319107 | 308 | 318799 |
| 803.2 | Fracture of radius and ulna | Injuries & Poisonings | 0.94 | 0.88 | 1.01 | 324441 | 884 | 323557 |
| 429.3 | Symptoms involving cardiovascular system | Circulatory System | 0.89 | 0.77 | 1.02 | 322241 | 208 | 322033 |
| 291.8 | Alteration of consciousness | Mental Disorders | 1.08 | 0.99 | 1.19 | 321904 | 434 | 321470 |
| 296.2 | Depression | Mental Disorders | 1.02 | 1.00 | 1.03 | 295043 | 12145 | 282898 |
| 333.4 | Torsion dystonia | Neurological | 1.10 | 0.98 | 1.24 | 286417 | 265 | 286152 |
| 284 | Aplastic anemia | Hematopoietic | 1.01 | 1.00 | 1.03 | 321659 | 12759 | 308900 |
| 150 | Cancer of esophagus | Neoplasms | 0.94 | 0.87 | 1.01 | 313058 | 727 | 312331 |
| 732 | Osteochondropathies | Musculoskeletal | 0.89 | 0.78 | 1.02 | 315651 | 219 | 315432 |
| 285.22 | Anemia in neoplastic disease | Hematopoietic | 0.92 | 0.84 | 1.02 | 309337 | 437 | 308900 |
| 292.2 | Mild cognitive impairment | Mental Disorders | 1.12 | 0.98 | 1.28 | 321672 | 202 | 321470 |
| 800.3 | Fracture of tibia and fibula | Injuries & Poisonings | 1.07 | 0.99 | 1.16 | 324092 | 535 | 323557 |
| 580.32 | Nephritis and nephropathy with pathological lesion | Genitourinary | 1.05 | 0.99 | 1.11 | 315157 | 1078 | 314079 |
| 715 | Other inflammatory spondylopathies | Musculoskeletal | 0.91 | 0.80 | 1.02 | 317524 | 271 | 317253 |
| 365.2 | Primary angle-closure glaucoma | Sense Organs | 0.94 | 0.87 | 1.01 | 317491 | 709 | 316782 |
| 197 | Chemotherapy | Neoplasms | 0.99 | 0.98 | 1.00 | 251774 | 22462 | 229312 |
| 586.2 | Cyst of kidney, acquired | Genitourinary | 0.96 | 0.90 | 1.01 | 315372 | 1293 | 314079 |
| 569.2 | Gastrointestinal complications | Digestive | 1.07 | 0.98 | 1.17 | 247132 | 472 | 246660 |
| 318 | Tobacco use disorder | Mental Disorders | 1.01 | 1.00 | 1.03 | 318172 | 20503 | 297669 |
| 189 | Cancer of urinary organs (incl. kidney and bladder) | Neoplasms | 0.97 | 0.94 | 1.01 | 326965 | 3003 | 323962 |
| 599.4 | Urinary incontinence | Genitourinary | 0.98 | 0.96 | 1.00 | 229423 | 8996 | 220427 |
| 803 | Fracture of upper limb | Injuries & Poisonings | 0.93 | 0.84 | 1.02 | 323990 | 433 | 323557 |
| 357 | Inflammatory and toxic neuropathy | Neurological | 1.04 | 0.99 | 1.10 | 327375 | 1423 | 325952 |
| 737 | Curvature of spine | Musculoskeletal | 1.06 | 0.98 | 1.14 | 317430 | 743 | 316687 |

| | | | | | | | | |
|---|---|---|---|---|---|---|---|---|
| 426.4 | Anomalous atrioventricular excitation | Circulatory System | 0.90 | 0.79 | 1.03 | 299761 | 240 | 299521 |
| 250.42 | Other abnormal glucose | Endocrine/Metabolic | 1.07 | 0.98 | 1.18 | 307927 | 437 | 307490 |
| 297.2 | Suicide or self-inflicted injury | Mental Disorders | 1.03 | 0.99 | 1.07 | 285655 | 2757 | 282898 |
| 687.4 | Disturbance of skin sensation | Dermatologic | 0.97 | 0.94 | 1.01 | 325793 | 2979 | 322814 |
| 360.2 | Progressive myopia | Sense Organs | 0.91 | 0.80 | 1.03 | 317032 | 250 | 316782 |
| 411.9 | Other acute and subacute forms of ischemic heart disease | Circulatory System | 1.04 | 0.99 | 1.11 | 296883 | 1194 | 295689 |
| 364.5 | Corneal dystrophy | Sense Organs | 1.11 | 0.97 | 1.26 | 316998 | 216 | 316782 |
| 296 | Mood disorders | Mental Disorders | 1.01 | 1.00 | 1.03 | 295853 | 12955 | 282898 |
| 430.1 | Subarachnoid hemorrhage | Circulatory System | 0.95 | 0.88 | 1.02 | 318931 | 824 | 318107 |
| 195 | Cancer, suspected or other | Neoplasms | 0.95 | 0.89 | 1.02 | 230227 | 915 | 229312 |
| 198.3 | Secondary malignant neoplasm of digestive systems | Neoplasms | 0.96 | 0.92 | 1.01 | 230877 | 1565 | 229312 |
| 290.2 | Delirium due to conditions classified elsewhere | Mental Disorders | 0.94 | 0.87 | 1.02 | 322151 | 681 | 321470 |
| 427.3 | Other specified cardiac dysrhythmias | Circulatory System | 0.97 | 0.94 | 1.01 | 302836 | 3315 | 299521 |
| 592.13 | Chronic interstitial cystitis | Genitourinary | 0.91 | 0.80 | 1.03 | 297289 | 254 | 297035 |
| 698 | Pruritus and related conditions | Dermatologic | 1.05 | 0.98 | 1.13 | 328240 | 799 | 327441 |
| 380.4 | Impacted cerumen | Sense Organs | 0.92 | 0.83 | 1.03 | 327106 | 354 | 326752 |
| 574.1 | Cholelithiasis | Digestive | 1.02 | 0.99 | 1.04 | 319629 | 9306 | 310323 |
| 385.5 | Tympanosclerosis and middle ear disease related to otitis media | Sense Organs | 0.91 | 0.80 | 1.04 | 324379 | 236 | 324143 |
| 290.1 | Dementias | Mental Disorders | 1.06 | 0.98 | 1.14 | 322121 | 651 | 321470 |
| 411.41 | Aneurysm and dissection of heart | Circulatory System | 0.95 | 0.88 | 1.02 | 296410 | 721 | 295689 |
| 368.2 | Diplopia and disorders of binocular vision | Sense Organs | 0.95 | 0.88 | 1.02 | 325635 | 753 | 324882 |
| 578 | Gastrointestinal hemorrhage | Digestive | 0.92 | 0.81 | 1.03 | 303679 | 285 | 303394 |
| 604.1 | Redundant prepuce and phimosis/BXO | Genitourinary | 0.97 | 0.94 | 1.01 | 310716 | 2810 | 307906 |
| 687 | Symptoms affecting skin | Dermatologic | 1.10 | 0.96 | 1.25 | 323032 | 218 | 322814 |
| 471 | Nasal polyps | Respiratory | 1.02 | 0.99 | 1.06 | 312342 | 3392 | 308950 |
| 53 | Herpes zoster | Infectious Diseases | 0.92 | 0.83 | 1.03 | 322547 | 341 | 322206 |
| 214.1 | Lipoma of skin and subcutaneous tissue | Neoplasms | 0.98 | 0.95 | 1.01 | 325432 | 4756 | 320676 |
| 871 | Open wounds of extremities | Injuries & Poisonings | 1.02 | 0.99 | 1.05 | 323873 | 4142 | 319731 |
| 530.14 | Reflux esophagitis | Digestive | 1.01 | 0.99 | 1.03 | 298696 | 10893 | 287803 |
| 603.1 | Hydrocele | Genitourinary | 0.96 | 0.91 | 1.02 | 309308 | 1402 | 307906 |
| 788 | Syncope and collapse | Symptoms | 0.99 | 0.97 | 1.01 | 328240 | 9372 | 318868 |
| 41.2 | Streptococcus infection | Infectious Diseases | 0.97 | 0.92 | 1.01 | 314280 | 1648 | 312632 |
| 496.2 | Chronic bronchitis | Respiratory | 1.08 | 0.97 | 1.21 | 294485 | 293 | 294192 |
| 277 | Other disorders of metabolism | Endocrine/Metabolic | 0.99 | 0.98 | 1.00 | 328230 | 45303 | 282927 |
| 416 | Cardiomegaly | Circulatory System | 0.97 | 0.94 | 1.01 | 324157 | 2610 | 321547 |
| 714.1 | Rheumatoid arthritis | Musculoskeletal | 1.03 | 0.99 | 1.07 | 319560 | 2307 | 317253 |
| 302 | Sexual and gender identity disorders | Mental Disorders | 1.08 | 0.97 | 1.20 | 283238 | 340 | 282898 |
| 532 | Dysphagia | Digestive | 0.98 | 0.96 | 1.01 | 294356 | 6553 | 287803 |

| | | | | | | | | |
|---|---|---|---|---|---|---|---|---|
| 368 | Visual disturbances | Sense Organs | 1.04 | 0.98 | 1.11 | 325963 | 1081 | 324882 |
| 276.12 | Hyposmolality and/or hyponatremia | Endocrine/Metabolic | 0.97 | 0.92 | 1.01 | 322496 | 1852 | 320644 |
| 695.7 | Prurigo and Lichen | Dermatologic | 0.95 | 0.89 | 1.02 | 322541 | 792 | 321749 |
| 969 | Poisoning by psychotropic agents | Injuries & Poisonings | 1.03 | 0.99 | 1.08 | 302207 | 1996 | 300211 |
| 367.2 | Astigmatism | Sense Organs | 0.91 | 0.79 | 1.04 | 325964 | 210 | 325754 |
| 433.1 | Occlusion and stenosis of precerebral arteries | Circulatory System | 1.04 | 0.98 | 1.11 | 319149 | 1042 | 318107 |
| 371.1 | Uveitis, noninfectious or NOS | Sense Organs | 1.06 | 0.97 | 1.16 | 318886 | 498 | 318388 |
| 508 | Pulmonary collapse; interstitial and compensatory emphysema | Respiratory | 0.97 | 0.93 | 1.01 | 318570 | 2086 | 316484 |
| 342 | Hemiplegia | Neurological | 0.97 | 0.92 | 1.02 | 287677 | 1525 | 286152 |
| 528.7 | Sialolithiasis | Digestive | 1.08 | 0.97 | 1.20 | 322847 | 313 | 322534 |
| 80 | Postoperative infection | Infectious Diseases | 0.98 | 0.95 | 1.01 | 326101 | 4622 | 321479 |
| 594.3 | Calculus of ureter | Genitourinary | 1.03 | 0.99 | 1.07 | 322471 | 2540 | 319931 |
| 448 | Disease of capillaries | Circulatory System | 1.02 | 0.99 | 1.04 | 326841 | 7481 | 319360 |
| 496.1 | Emphysema | Respiratory | 0.97 | 0.92 | 1.02 | 295918 | 1726 | 294192 |
| 618.6 | Vaginal enterocele, congenital or acquired | Genitourinary | 1.05 | 0.98 | 1.13 | 316909 | 667 | 316242 |
| 939 | Atopic/contact dermatitis due to other or unspecified | Dermatologic | 1.03 | 0.99 | 1.07 | 324060 | 2154 | 321906 |
| 571.8 | Liver abscess and sequelae of chronic liver disease | Digestive | 0.95 | 0.87 | 1.03 | 318904 | 573 | 318331 |
| 642 | Hypertension complicating pregnancy, childbirth, and the puerperium | Pregnancy Complications | 0.96 | 0.90 | 1.02 | 328039 | 989 | 327050 |
| 306.9 | Tension headache | Mental Disorders | 1.08 | 0.96 | 1.20 | 283203 | 305 | 282898 |
| 70.3 | Viral hepatitis C | Infectious Diseases | 0.93 | 0.84 | 1.04 | 322549 | 343 | 322206 |
| 564 | Functional digestive disorders | Digestive | 1.04 | 0.98 | 1.11 | 259379 | 983 | 258396 |
| 686.4 | Pyogenic granuloma | Dermatologic | 0.93 | 0.84 | 1.04 | 316787 | 380 | 316407 |
| 722.6 | Degeneration of intervertebral disc | Musculoskeletal | 0.98 | 0.94 | 1.01 | 313866 | 2922 | 310944 |
| 244.4 | Hypothyroidism NOS | Endocrine/Metabolic | 1.01 | 0.99 | 1.03 | 324759 | 14430 | 310329 |
| 626.14 | Irregular menstrual bleeding | Genitourinary | 1.02 | 0.99 | 1.05 | 300177 | 3946 | 296231 |
| 213 | Benign neoplasm of bone and articular cartilage | Neoplasms | 0.93 | 0.83 | 1.04 | 277528 | 322 | 277206 |
| 345.11 | Generalized convulsive epilepsy | Neurological | 0.94 | 0.86 | 1.03 | 286617 | 465 | 286152 |
| 374 | Other disorders of eyelids | Sense Organs | 0.98 | 0.94 | 1.01 | 321593 | 3205 | 318388 |
| 636 | Early or threatened labor; hemorrhage in early pregnancy | Pregnancy Complications | 0.96 | 0.91 | 1.02 | 321227 | 1262 | 319965 |
| 743.9 | Osteopenia or other disorder of bone and cartilage | Musculoskeletal | 1.02 | 0.99 | 1.05 | 328188 | 3989 | 324199 |
| 289.5 | Diseases of spleen | Hematopoietic | 1.06 | 0.97 | 1.15 | 320846 | 528 | 320318 |
| 525 | Other diseases of the teeth and supporting structures | Digestive | 1.02 | 0.99 | 1.06 | 314357 | 2699 | 311658 |
| 394.7 | Disease of tricuspid valve | Circulatory System | 0.96 | 0.91 | 1.02 | 322614 | 1086 | 321528 |
| 345 | Epilepsy, recurrent seizures, convulsions | Neurological | 0.98 | 0.95 | 1.01 | 289613 | 3461 | 286152 |
| 524.3 | Anomalies of tooth position/malocclusion | Digestive | 0.93 | 0.83 | 1.04 | 311953 | 295 | 311658 |
| 295.1 | Schizophrenia | Mental Disorders | 1.05 | 0.97 | 1.14 | 283509 | 611 | 282898 |
| 574 | Cholelithiasis and cholecystitis | Digestive | 0.94 | 0.85 | 1.04 | 310726 | 403 | 310323 |
| 341 | Other demyelinating diseases of central nervous system | Neurological | 0.97 | 0.92 | 1.02 | 287764 | 1612 | 286152 |

| | | | | | | | | | |
|---|---|---|---|---|---|---|---|---|---|
| 338.2 | Chronic pain | Neurological | 0.94 | 0.85 | 1.04 | 327830 | 404 | 327426 |
| 800 | Fracture of lower limb | Injuries & Poisonings | 1.05 | 0.97 | 1.13 | 324256 | 699 | 323557 |
| 723 | Other disorders of cervical region | Musculoskeletal | 0.94 | 0.85 | 1.04 | 311345 | 401 | 310944 |
| 250.7 | Diabetic retinopathy | Endocrine/Metabolic | 1.03 | 0.98 | 1.09 | 317232 | 1380 | 315852 |
| 386.3 | Labyrinthitis | Sense Organs | 1.04 | 0.97 | 1.12 | 322440 | 791 | 321649 |
| 740.12 | Osteoarthrosis, localized, secondary | Musculoskeletal | 1.08 | 0.96 | 1.21 | 306589 | 264 | 306325 |
| 218 | Benign neoplasm of uterus | Neoplasms | 0.94 | 0.85 | 1.04 | 308168 | 388 | 307780 |
| 54 | Herpes simplex | Infectious Diseases | 0.92 | 0.80 | 1.05 | 322425 | 219 | 322206 |
| 174.1 | Breast cancer [female] | Neoplasms | 0.97 | 0.93 | 1.02 | 306711 | 2287 | 304424 |
| 352.2 | Facial nerve disorders [CN7] | Neurological | 1.04 | 0.98 | 1.11 | 313911 | 900 | 313011 |
| 598 | Abnormal findings on examination of urine | Genitourinary | 0.98 | 0.95 | 1.01 | 328240 | 3386 | 324854 |
| 994.2 | Sepsis | Injuries & Poisonings | 1.02 | 0.99 | 1.06 | 328240 | 2885 | 325355 |
| 965.1 | Opiates and related narcotics causing adverse effects in therapeutic use | Injuries & Poisonings | 0.97 | 0.91 | 1.02 | 301372 | 1161 | 300211 |
| 38 | Septicemia | Infectious Diseases | 1.02 | 0.99 | 1.06 | 315677 | 3045 | 312632 |
| 628 | Ovarian cyst | Genitourinary | 1.02 | 0.99 | 1.05 | 301051 | 4820 | 296231 |
| 253.2 | Pituitary hypofunction | Endocrine/Metabolic | 0.93 | 0.82 | 1.05 | 324826 | 281 | 324545 |
| 729 | Other disorders of soft tissues | Musculoskeletal | 0.99 | 0.98 | 1.01 | 327478 | 22524 | 304954 |
| 440.2 | Atherosclerosis of the extremities | Circulatory System | 0.95 | 0.87 | 1.03 | 319912 | 552 | 319360 |
| 858 | Complication of internal orthopedic device | Injuries & Poisonings | 1.02 | 0.99 | 1.06 | 317147 | 3183 | 313964 |
| 613.9 | Breast disorder NOS | Genitourinary | 1.02 | 0.99 | 1.04 | 327937 | 5519 | 322418 |
| 735.23 | Hallux rigidus | Musculoskeletal | 0.97 | 0.92 | 1.02 | 318243 | 1556 | 316687 |
| 577.1 | Acute pancreatitis | Digestive | 0.97 | 0.92 | 1.02 | 327437 | 1517 | 325920 |
| 702.1 | Actinic keratosis | Dermatologic | 1.02 | 0.98 | 1.06 | 325264 | 2664 | 322600 |
| 427.11 | Paroxysmal supraventricular tachycardia | Circulatory System | 0.98 | 0.94 | 1.02 | 301952 | 2431 | 299521 |
| 938 | Dermatitis due to solar radiation | Injuries & Poisonings | 1.02 | 0.98 | 1.06 | 324444 | 2538 | 321906 |
| 372 | Disorders of conjunctiva | Sense Organs | 0.96 | 0.91 | 1.03 | 319431 | 1043 | 318388 |
| 501 | Pneumonitis due to inhalation of food or vomitus | Respiratory | 0.95 | 0.88 | 1.03 | 317076 | 592 | 316484 |
| 622 | Polyp of female genital organs | Genitourinary | 0.95 | 0.87 | 1.04 | 315794 | 487 | 315307 |
| 495 | Asthma | Respiratory | 0.99 | 0.98 | 1.01 | 320971 | 26779 | 294192 |
| 742.9 | Other derangement of joint | Musculoskeletal | 1.06 | 0.96 | 1.17 | 311042 | 400 | 310642 |
| 907 | Injuries to the nervous system | Injuries & Poisonings | 1.03 | 0.98 | 1.07 | 328240 | 1845 | 326395 |
| 564.9 | Personal history of diseases of digestive system | Digestive | 0.99 | 0.97 | 1.01 | 274320 | 15924 | 258396 |
| 317.11 | Alcoholic liver damage | Mental Disorders | 1.04 | 0.97 | 1.11 | 298534 | 865 | 297669 |
| 649.1 | Diabetes or abnormal glucose tolerance complicating pregnancy | Pregnancy Complications | 1.08 | 0.94 | 1.24 | 328127 | 208 | 327919 |
| 218.1 | Uterine leiomyoma | Neoplasms | 0.99 | 0.97 | 1.01 | 318346 | 10566 | 307780 |
| 340.1 | Migrain with aura | Neurological | 0.92 | 0.81 | 1.06 | 318167 | 217 | 317950 |
| 281.11 | Pernicious anemia | Hematopoietic | 0.96 | 0.89 | 1.03 | 309650 | 750 | 308900 |
| 571.81 | Portal hypertension | Digestive | 1.05 | 0.97 | 1.14 | 318900 | 569 | 318331 |

| | | | | | | | | |
|---|---|---|---|---|---|---|---|---|
| 380.1 | Otitis externa | Sense Organs | 0.95 | 0.87 | 1.04 | 327284 | 532 | 326752 |
| 789 | Nausea and vomiting | Symptoms | 0.99 | 0.97 | 1.01 | 328240 | 11983 | 316257 |
| 751.12 | Congenital anomalies of male genital organs | Congenital Anomalies | 0.93 | 0.81 | 1.06 | 326231 | 228 | 326003 |
| 481 | Influenza | Respiratory | 0.99 | 0.97 | 1.01 | 327706 | 10017 | 317689 |
| 519.2 | Respiratory complications | Respiratory | 1.07 | 0.95 | 1.19 | 265743 | 296 | 265447 |
| 526.9 | Jaw disease NOS | Digestive | 1.01 | 0.99 | 1.03 | 327620 | 15962 | 311658 |
| 687.1 | Rash and other nonspecific skin eruption | Dermatologic | 0.98 | 0.94 | 1.02 | 325032 | 2218 | 322814 |
| 427.8 | Sinoatrial node dysfunction (Bradycardia) | Circulatory System | 0.95 | 0.86 | 1.04 | 299940 | 419 | 299521 |
| 474.2 | Chronic tonsillitis and adenoiditis | Respiratory | 1.03 | 0.98 | 1.09 | 310135 | 1185 | 308950 |
| 411.1 | Unstable angina (intermediate coronary syndrome) | Circulatory System | 0.98 | 0.96 | 1.01 | 301011 | 5322 | 295689 |
| 263 | Other nutritional deficiency | Endocrine/Metabolic | 0.97 | 0.92 | 1.02 | 327337 | 1551 | 325786 |
| 626.2 | Dysmenorrhea | Genitourinary | 0.97 | 0.93 | 1.02 | 297910 | 1679 | 296231 |
| 575.8 | Other disorders of biliary tract | Digestive | 0.97 | 0.91 | 1.03 | 311372 | 1049 | 310323 |
| 496.21 | Obstructive chronic bronchitis | Respiratory | 0.98 | 0.94 | 1.02 | 296921 | 2729 | 294192 |
| 353 | Nerve root and plexus disorders | Neurological | 0.97 | 0.91 | 1.03 | 314005 | 994 | 313011 |
| 612.2 | Hypertrophy of breast (Gynecomastia) | Genitourinary | 1.04 | 0.97 | 1.11 | 321732 | 859 | 320873 |
| 526 | Diseases of the jaws | Digestive | 1.01 | 0.99 | 1.03 | 327645 | 15987 | 311658 |
| 573.5 | Jaundice (not of newborn) | Digestive | 1.04 | 0.97 | 1.10 | 319269 | 938 | 318331 |
| 579.2 | Splenomegaly | Digestive | 1.05 | 0.96 | 1.15 | 303837 | 443 | 303394 |
| 562.1 | Diverticulosis | Digestive | 0.99 | 0.98 | 1.01 | 286281 | 27885 | 258396 |
| 599.2 | Retention of urine | Genitourinary | 0.99 | 0.96 | 1.01 | 227284 | 6857 | 220427 |
| 627.2 | Symptomatic menopause | Genitourinary | 0.93 | 0.80 | 1.07 | 296434 | 203 | 296231 |
| 411.3 | Angina pectoris | Circulatory System | 0.99 | 0.98 | 1.01 | 312048 | 16359 | 295689 |
| 626.1 | Irregular menstrual cycle/bleeding | Genitourinary | 0.98 | 0.95 | 1.02 | 299824 | 3593 | 296231 |
| 624.9 | stress incontinence, female | Genitourinary | 0.99 | 0.96 | 1.01 | 324769 | 6100 | 318669 |
| 426.23 | Second degree AV block | Circulatory System | 0.95 | 0.87 | 1.04 | 299982 | 461 | 299521 |
| 530.5 | Disorders of esophageal motility | Digestive | 1.04 | 0.97 | 1.12 | 288452 | 649 | 287803 |
| 503 | Pulmonary congestion and hypostasis | Respiratory | 1.05 | 0.96 | 1.15 | 316959 | 475 | 316484 |
| 292.1 | Aphasia/speech disturbance | Mental Disorders | 0.97 | 0.93 | 1.02 | 323019 | 1549 | 321470 |
| 38.2 | Gram positive septicemia | Infectious Diseases | 1.05 | 0.96 | 1.14 | 313123 | 491 | 312632 |
| 506 | Empyema and pneumothorax | Respiratory | 0.97 | 0.91 | 1.03 | 317630 | 1146 | 316484 |
| 619.5 | Noninflammatory disorders of vulva and perineum | Genitourinary | 0.97 | 0.92 | 1.03 | 279927 | 1379 | 278548 |
| 480.11 | Pneumococcal pneumonia | Respiratory | 0.99 | 0.96 | 1.01 | 323680 | 5991 | 317689 |
| 608 | Other disorders of male genital organs | Genitourinary | 0.99 | 0.98 | 1.01 | 327998 | 20092 | 307906 |
| 722 | Intervertebral disc disorders | Musculoskeletal | 0.99 | 0.96 | 1.01 | 316907 | 5963 | 310944 |
| 621 | Endometrial hyperplasia | Genitourinary | 0.97 | 0.91 | 1.03 | 316461 | 1154 | 315307 |
| 560.4 | Other intestinal obstruction | Digestive | 0.98 | 0.95 | 1.02 | 261839 | 3443 | 258396 |
| 427.41 | Ventricular fibrillation and flutter | Circulatory System | 1.06 | 0.95 | 1.17 | 299866 | 345 | 299521 |

| | | | | | | | | |
|---|---|---|---|---|---|---|---|---|
| 756 | Other congenital musculoskeletal anomalies | Congenital Anomalies | 1.04 | 0.97 | 1.11 | 328123 | 812 | 327311 |
| 749 | Congenital anomalies of face and neck | Congenital Anomalies | 0.94 | 0.83 | 1.06 | 328137 | 271 | 327866 |
| 228 | Hemangioma and lymphangioma, any site | Neoplasms | 1.03 | 0.98 | 1.08 | 328240 | 1635 | 326605 |
| 977 | Personal history of allergy to medicinal agents | Injuries & Poisonings | 1.07 | 0.94 | 1.21 | 300449 | 238 | 300211 |
| 962.3 | Hormones and synthetic substitutes causing adverse effects in therapeutic use | Injuries & Poisonings | 0.95 | 0.85 | 1.05 | 300575 | 364 | 300211 |
| 537 | Other disorders of stomach and duodenum | Digestive | 1.02 | 0.98 | 1.05 | 299994 | 3483 | 296511 |
| 464 | Acute sinusitis | Respiratory | 1.07 | 0.94 | 1.23 | 325387 | 208 | 325179 |
| 378.1 | Strabismus (not specified as paralytic) | Sense Organs | 0.97 | 0.91 | 1.03 | 289139 | 947 | 288192 |
| 229 | Benign neoplasm of unspecified sites | Neoplasms | 0.99 | 0.99 | 1.00 | 328240 | 46165 | 282075 |
| 519.9 | Symptoms involving respiratory system and other chest symptoms | Respiratory | 0.94 | 0.84 | 1.06 | 265755 | 308 | 265447 |
| 528 | Diseases of the oral soft tissues, excluding lesions specific for gingiva and tongue | Digestive | 0.98 | 0.94 | 1.02 | 324593 | 2059 | 322534 |
| 425.1 | Primary/intrinsic cardiomyopathies | Circulatory System | 0.97 | 0.91 | 1.03 | 326036 | 1053 | 324983 |
| 782.3 | Edema | Symptoms | 1.02 | 0.98 | 1.07 | 328067 | 1715 | 326352 |
| 512.9 | Other dyspnea | Respiratory | 0.97 | 0.91 | 1.03 | 315211 | 943 | 314268 |
| 427.4 | Cardiac arrest and ventricular fibrillation | Circulatory System | 1.05 | 0.95 | 1.17 | 299869 | 348 | 299521 |
| 433.8 | Late effects of cerebrovascular disease | Circulatory System | 0.97 | 0.92 | 1.03 | 319384 | 1277 | 318107 |
| 599.3 | Dysuria | Genitourinary | 0.97 | 0.92 | 1.03 | 221636 | 1209 | 220427 |
| 704 | Diseases of hair and hair follicles | Dermatologic | 1.01 | 0.99 | 1.04 | 326692 | 5263 | 321429 |
| 350.1 | Abnormal involuntary movements | Neurological | 0.97 | 0.91 | 1.03 | 326371 | 927 | 325444 |
| 250.23 | Type 2 diabetes with ophthalmic manifestations | Endocrine/Metabolic | 1.03 | 0.97 | 1.08 | 308834 | 1344 | 307490 |
| 429.2 | Abnormal function study of cardiovascular system | Circulatory System | 0.96 | 0.88 | 1.04 | 322577 | 544 | 322033 |
| 202.21 | Nodular lymphoma | Neoplasms | 0.95 | 0.86 | 1.05 | 323913 | 390 | 323523 |
| 204.21 | Myeloid leukemia, acute | Neoplasms | 1.06 | 0.95 | 1.18 | 323842 | 319 | 323523 |
| 674 | Other complications of the puerperium NEC | Pregnancy Complications | 1.06 | 0.94 | 1.19 | 328015 | 280 | 327735 |
| 317.1 | Alcoholism | Mental Disorders | 0.99 | 0.97 | 1.01 | 306366 | 8697 | 297669 |
| 571.6 | Primary biliary cirrhosis | Digestive | 1.06 | 0.94 | 1.20 | 318589 | 258 | 318331 |
| 8.6 | Viral Enteritis | Infectious Diseases | 0.97 | 0.90 | 1.04 | 319924 | 845 | 319079 |
| 707 | Chronic ulcer of skin | Dermatologic | 0.97 | 0.92 | 1.03 | 327959 | 1260 | 326699 |
| 530.1 | Esophagitis, GERD and related diseases | Digestive | 1.01 | 0.99 | 1.03 | 297342 | 9539 | 287803 |
| 198.2 | Secondary malignancy of respiratory organs | Neoplasms | 0.98 | 0.94 | 1.02 | 231589 | 2277 | 229312 |
| 433 | Cerebrovascular disease | Circulatory System | 0.99 | 0.97 | 1.01 | 326753 | 8646 | 318107 |
| 555.2 | Ulcerative colitis | Digestive | 0.98 | 0.95 | 1.02 | 261550 | 3154 | 258396 |
| 614.51 | Cervicitis and endocervicitis | Genitourinary | 1.03 | 0.97 | 1.09 | 320048 | 1249 | 318799 |
| 527.2 | Sialoadenitis | Digestive | 1.05 | 0.95 | 1.16 | 322908 | 374 | 322534 |
| 306 | Other mental disorder | Mental Disorders | 0.99 | 0.98 | 1.01 | 312303 | 29405 | 282898 |
| 497 | Bronchitis | Respiratory | 0.96 | 0.89 | 1.04 | 294834 | 642 | 294192 |
| 521.1 | Dental caries | Digestive | 1.02 | 0.98 | 1.05 | 314912 | 3254 | 311658 |
| 430.3 | Subdural hemorrhage | Circulatory System | 0.94 | 0.84 | 1.07 | 318378 | 271 | 318107 |

| | | | | | | | | |
|---|---|---|---|---|---|---|---|---|
| 396 | Abnormal heart sounds | Circulatory System | 0.97 | 0.91 | 1.03 | 322603 | 1075 | 321528 |
| 272.9 | Unspecified disorder of lipoid metabolism | Endocrine/Metabolic | 1.06 | 0.93 | 1.21 | 291721 | 224 | 291497 |
| 304 | Adjustment reaction | Mental Disorders | 1.05 | 0.95 | 1.17 | 283221 | 323 | 282898 |
| 208 | Benign neoplasm of colon | Neoplasms | 0.99 | 0.98 | 1.01 | 325437 | 20827 | 304610 |
| 367.8 | Hypermetropia | Sense Organs | 0.94 | 0.83 | 1.07 | 325995 | 241 | 325754 |
| 452 | Other venous embolism and thrombosis | Circulatory System | 0.96 | 0.88 | 1.05 | 288267 | 554 | 287713 |
| 601.1 | Prostatitis | Genitourinary | 1.04 | 0.96 | 1.12 | 308058 | 665 | 307393 |
| 201 | Hodgkin's disease | Neoplasms | 1.06 | 0.94 | 1.19 | 323794 | 271 | 323523 |
| 760 | Back pain | Symptoms | 1.01 | 0.99 | 1.04 | 328240 | 5923 | 322317 |
| 870 | Open wounds of head; neck; and trunk | Injuries & Poisonings | 0.96 | 0.89 | 1.04 | 320327 | 596 | 319731 |
| 578.8 | Hemorrhage of rectum and anus | Digestive | 0.99 | 0.98 | 1.01 | 317220 | 13826 | 303394 |
| 276.6 | Fluid overload | Endocrine/Metabolic | 0.96 | 0.87 | 1.05 | 321117 | 473 | 320644 |
| 433.3 | Cerebral ischemia | Circulatory System | 0.97 | 0.91 | 1.04 | 319041 | 934 | 318107 |
| 741 | Symptoms and disorders of the joints | Musculoskeletal | 0.99 | 0.98 | 1.01 | 327531 | 16889 | 310642 |
| 626.8 | Infertility, female | Genitourinary | 1.03 | 0.97 | 1.08 | 297638 | 1407 | 296231 |
| 531 | Peptic ulcer (excl. esophageal) | Digestive | 0.95 | 0.84 | 1.07 | 320833 | 284 | 320549 |
| 965.2 | Antirheumatics causing adverse effects in therapeutic use | Injuries & Poisonings | 1.04 | 0.95 | 1.14 | 300710 | 499 | 300211 |
| 623 | Hypertrophy of female genital organs | Genitourinary | 1.03 | 0.97 | 1.08 | 316579 | 1272 | 315307 |
| 579.8 | Nonspecific abnormal findings in stool contents | Digestive | 0.98 | 0.93 | 1.03 | 305134 | 1740 | 303394 |
| 443.1 | Raynaud's syndrome | Circulatory System | 1.03 | 0.97 | 1.09 | 320525 | 1165 | 319360 |
| 700 | Corns and callosities | Dermatologic | 0.95 | 0.84 | 1.07 | 323352 | 288 | 323064 |
| 473.4 | Voice disturbance | Respiratory | 0.97 | 0.92 | 1.03 | 310051 | 1101 | 308950 |
| 726.2 | Synoviopathy | Musculoskeletal | 0.95 | 0.85 | 1.06 | 305285 | 331 | 304954 |
| 519 | Other diseases of respiratory system, not elsewhere classified | Respiratory | 1.00 | 0.99 | 1.00 | 327615 | 62168 | 265447 |
| 823 | Fracture of tibia and fibula | Injuries & Poisonings | 1.05 | 0.94 | 1.17 | 322362 | 342 | 322020 |
| 585.3 | Chronic renal failure [CKD] | Genitourinary | 1.02 | 0.98 | 1.06 | 316664 | 2585 | 314079 |
| 798 | Malaise and fatigue | Symptoms | 0.98 | 0.95 | 1.02 | 327695 | 2966 | 324729 |
| 427.7 | Tachycardia NOS | Circulatory System | 0.98 | 0.94 | 1.02 | 301787 | 2266 | 299521 |
| 379.2 | Disorders of vitreous body | Sense Organs | 0.98 | 0.93 | 1.03 | 289599 | 1407 | 288192 |
| 212 | Benign neoplasm of respiratory and intrathoracic organs | Neoplasms | 0.95 | 0.86 | 1.06 | 328240 | 355 | 327885 |
| 41.1 | Staphylococcus infections | Infectious Diseases | 0.98 | 0.95 | 1.02 | 315832 | 3200 | 312632 |
| 225.1 | Benign neoplasm of brain, cranial nerves, meninges | Neoplasms | 0.97 | 0.90 | 1.04 | 327267 | 820 | 326447 |
| 312 | Conduct disorders | Mental Disorders | 0.97 | 0.92 | 1.03 | 327638 | 1195 | 326443 |
| 204.12 | Lymphoid leukemia, chronic | Neoplasms | 1.04 | 0.95 | 1.13 | 324030 | 507 | 323523 |
| 706.2 | Sebaceous cyst | Dermatologic | 1.01 | 0.99 | 1.03 | 327401 | 9107 | 318294 |
| 155 | Cancer of liver and intrahepatic bile duct | Neoplasms | 0.94 | 0.83 | 1.07 | 312576 | 245 | 312331 |
| 736.2 | Acquired deformities of finger | Musculoskeletal | 0.95 | 0.83 | 1.07 | 316940 | 253 | 316687 |
| 681.6 | Cellulitis and abscess of foot, toe | Dermatologic | 0.99 | 0.96 | 1.01 | 322141 | 5734 | 316407 |

| | | | | | | | | |
|---|---|---|---|---|---|---|---|---|
| 597 | Other disorders of urethra and urinary tract | Genitourinary | 0.97 | 0.91 | 1.04 | 314510 | 874 | 313636 |
| 594 | Urinary calculus | Genitourinary | 0.99 | 0.97 | 1.01 | 326897 | 6966 | 319931 |
| 557 | Intestinal malabsorption (non-celiac) | Digestive | 1.05 | 0.94 | 1.18 | 258690 | 294 | 258396 |
| 331 | Other cerebral degenerations | Neurological | 1.06 | 0.93 | 1.20 | 286392 | 240 | 286152 |
| 158 | Neoplasm of unspecified nature of digestive system | Neoplasms | 1.03 | 0.97 | 1.09 | 313429 | 1098 | 312331 |
| 745 | Pain in joint | Musculoskeletal | 0.98 | 0.94 | 1.03 | 328240 | 1940 | 326300 |
| 504 | Other alveolar and parietoalveolar pneumonopathy | Respiratory | 1.05 | 0.94 | 1.17 | 316793 | 309 | 316484 |
| 564.1 | Irritable Bowel Syndrome | Digestive | 1.01 | 0.99 | 1.04 | 264057 | 5661 | 258396 |
| 722.1 | Displacement of intervertebral disc | Musculoskeletal | 1.04 | 0.95 | 1.13 | 311460 | 516 | 310944 |
| 681.3 | Cellulitis and abscess of arm/hand | Dermatologic | 0.99 | 0.96 | 1.01 | 322177 | 5770 | 316407 |
| 250.6 | Polyneuropathy in diabetes | Endocrine/Metabolic | 1.04 | 0.95 | 1.15 | 307871 | 381 | 307490 |
| 477 | Epistaxis or throat hemorrhage | Respiratory | 0.98 | 0.94 | 1.02 | 311469 | 2519 | 308950 |
| 964.1 | Anticoagulants causing adverse effects | Injuries & Poisonings | 1.05 | 0.93 | 1.19 | 300470 | 259 | 300211 |
| 433.2 | Occlusion of cerebral arteries | Circulatory System | 1.02 | 0.98 | 1.05 | 321087 | 2980 | 318107 |
| 348.8 | Encephalopathy, not elsewhere classified | Neurological | 1.06 | 0.93 | 1.21 | 286356 | 204 | 286152 |
| 242 | Thyrotoxicosis with or without goiter | Endocrine/Metabolic | 0.98 | 0.93 | 1.03 | 311794 | 1465 | 310329 |
| 290.11 | Alzheimer's disease | Mental Disorders | 1.04 | 0.95 | 1.15 | 321872 | 402 | 321470 |
| 601.8 | Other inflammatory disorders of male genital organs | Genitourinary | 1.05 | 0.94 | 1.17 | 307698 | 305 | 307393 |
| 520.2 | Disturbances in tooth eruption | Digestive | 1.02 | 0.98 | 1.06 | 314095 | 2437 | 311658 |
| 174.11 | Malignant neoplasm of female breast | Neoplasms | 1.01 | 0.99 | 1.03 | 316838 | 12414 | 304424 |
| 465.2 | Acute pharyngitis | Respiratory | 0.97 | 0.91 | 1.04 | 326053 | 874 | 325179 |
| 510 | Other diseases of lung | Respiratory | 1.03 | 0.96 | 1.11 | 328200 | 770 | 327430 |
| 585.31 | Renal dialysis | Genitourinary | 0.96 | 0.88 | 1.05 | 314603 | 524 | 314079 |
| 292.6 | Hallucinations | Mental Disorders | 1.05 | 0.94 | 1.17 | 321793 | 323 | 321470 |
| 619.4 | Noninflammatory disorders of vagina | Genitourinary | 1.02 | 0.97 | 1.07 | 280328 | 1780 | 278548 |
| 752 | Nervous system congenital anomalies | Congenital Anomalies | 0.95 | 0.85 | 1.07 | 327963 | 288 | 327675 |
| 202.24 | Large cell lymphoma | Neoplasms | 1.03 | 0.95 | 1.12 | 324112 | 589 | 323523 |
| 600 | Hyperplasia of prostate | Genitourinary | 0.99 | 0.97 | 1.01 | 318965 | 11572 | 307393 |
| 653 | Problems associated with amniotic cavity and membranes | Pregnancy Complications | 0.98 | 0.93 | 1.03 | 328119 | 1523 | 326596 |
| 386 | Vertiginous syndromes and other disorders of vestibular system | Sense Organs | 1.02 | 0.98 | 1.06 | 323761 | 2112 | 321649 |
| 783 | Fever of unknown origin | Symptoms | 1.01 | 0.98 | 1.04 | 328240 | 4111 | 324129 |
| 575.6 | Cholesterolosis of gallbladder | Digestive | 0.96 | 0.88 | 1.06 | 310789 | 466 | 310323 |
| 379.5 | Disorders of iris and ciliary body | Sense Organs | 1.04 | 0.95 | 1.15 | 288604 | 412 | 288192 |
| 281 | Other deficiency anemia | Hematopoietic | 0.99 | 0.97 | 1.01 | 317687 | 8787 | 308900 |
| 614.33 | Pelvic inflammatory disease, NOS | Genitourinary | 1.01 | 0.99 | 1.04 | 324944 | 6145 | 318799 |
| 415 | Pulmonary heart disease | Circulatory System | 1.01 | 0.98 | 1.05 | 325300 | 3753 | 321547 |
| 613 | Other nonmalignant breast conditions | Genitourinary | 1.04 | 0.94 | 1.16 | 322786 | 368 | 322418 |
| 726.3 | Bursitis | Musculoskeletal | 0.97 | 0.89 | 1.05 | 305497 | 543 | 304954 |

| | | | | | | | | |
|---|---|---|---|---|---|---|---|---|
| 751.22 | Other specified congenital anomalies of kidney | Congenital Anomalies | 1.04 | 0.94 | 1.16 | 326353 | 350 | 326003 |
| 38.1 | Gram negative septicemia | Infectious Diseases | 0.97 | 0.91 | 1.04 | 313487 | 855 | 312632 |
| 529.1 | Glossitis | Digestive | 1.05 | 0.94 | 1.17 | 322838 | 304 | 322534 |
| 427.12 | Paroxysmal ventricular tachycardia | Circulatory System | 0.97 | 0.91 | 1.04 | 300490 | 969 | 299521 |
| 427.5 | Arrhythmia (cardiac) NOS | Circulatory System | 0.97 | 0.91 | 1.04 | 300469 | 948 | 299521 |
| 853 | Complication of colostomy or enterostomy | Injuries & Poisonings | 0.96 | 0.88 | 1.05 | 314478 | 514 | 313964 |
| 279.7 | Other immunological findings | Endocrine/Metabolic | 0.95 | 0.84 | 1.08 | 327978 | 264 | 327714 |
| 519.8 | Other diseases of respiratory system, NEC | Respiratory | 0.99 | 0.97 | 1.01 | 274529 | 9082 | 265447 |
| 454 | Varicose veins | Circulatory System | 0.98 | 0.93 | 1.03 | 289316 | 1603 | 287713 |
| 540 | Appendiceal conditions | Digestive | 0.99 | 0.96 | 1.02 | 328240 | 3861 | 324379 |
| 286.12 | Congenital deficiency of other clotting factors (including factor VII) | Hematopoietic | 1.05 | 0.94 | 1.17 | 325800 | 303 | 325497 |
| 475 | Chronic sinusitis | Respiratory | 1.02 | 0.98 | 1.06 | 311580 | 2630 | 308950 |
| 573.7 | Abnormal results of function study of liver | Digestive | 1.01 | 0.98 | 1.05 | 321896 | 3565 | 318331 |
| 550.2 | Diaphragmatic hernia | Digestive | 1.00 | 0.99 | 1.02 | 307453 | 27664 | 279789 |
| 550.1 | Inguinal hernia | Digestive | 1.01 | 0.99 | 1.02 | 296174 | 16385 | 279789 |
| 800.1 | Fracture of neck of femur | Injuries & Poisonings | 0.95 | 0.84 | 1.08 | 323796 | 239 | 323557 |
| 378.5 | Paralytic strabismus | Sense Organs | 1.05 | 0.93 | 1.17 | 288479 | 287 | 288192 |
| 428.1 | Congestive heart failure (CHF) NOS | Circulatory System | 1.02 | 0.97 | 1.06 | 324086 | 2053 | 322033 |
| 165.1 | Cancer of bronchus; lung | Neoplasms | 1.02 | 0.97 | 1.06 | 327450 | 2181 | 325269 |
| 426.31 | Right bundle branch block | Circulatory System | 0.98 | 0.93 | 1.03 | 301094 | 1573 | 299521 |
| 214 | Lipoma | Neoplasms | 1.02 | 0.97 | 1.07 | 322495 | 1819 | 320676 |
| 245.21 | Chronic lymphocytic thyroiditis | Endocrine/Metabolic | 1.05 | 0.92 | 1.20 | 310548 | 219 | 310329 |
| 703 | Diseases of nail, NOS | Dermatologic | 1.04 | 0.94 | 1.15 | 321795 | 366 | 321429 |
| 740.2 | Osteoarthrosis, generalized | Musculoskeletal | 0.97 | 0.89 | 1.05 | 306914 | 589 | 306325 |
| 444.1 | Arterial embolism and thrombosis of lower extremity artery | Circulatory System | 0.97 | 0.89 | 1.05 | 319930 | 570 | 319360 |
| 371.3 | Inflammation of eyelids | Sense Organs | 1.02 | 0.98 | 1.06 | 320786 | 2398 | 318388 |
| 669 | Complications of labor and delivery NEC | Pregnancy Complications | 0.99 | 0.97 | 1.02 | 328240 | 9534 | 318706 |
| 327 | Sleep disorders | Neurological | 0.98 | 0.92 | 1.04 | 323754 | 1034 | 322720 |
| 361 | Retinal detachments and defects | Sense Organs | 1.02 | 0.97 | 1.06 | 319011 | 2229 | 316782 |
| 642.1 | Preeclampsia and eclampsia | Pregnancy Complications | 0.96 | 0.86 | 1.07 | 327385 | 335 | 327050 |
| 540.1 | Appendicitis | Digestive | 0.97 | 0.91 | 1.04 | 325174 | 795 | 324379 |
| 369.5 | Conjunctivitis, infectious | Sense Organs | 0.95 | 0.83 | 1.09 | 318612 | 224 | 318388 |
| 335 | Multiple sclerosis | Neurological | 0.98 | 0.93 | 1.03 | 287531 | 1379 | 286152 |
| 681.5 | Cellulitis and abscess of leg, except foot | Dermatologic | 0.99 | 0.96 | 1.02 | 322184 | 5777 | 316407 |
| 415.21 | Primary pulmonary hypertension | Circulatory System | 0.97 | 0.88 | 1.06 | 322013 | 466 | 321547 |
| 961 | Poisoning by other anti-infectives | Injuries & Poisonings | 0.97 | 0.88 | 1.06 | 300647 | 436 | 300211 |
| 614.32 | Chronic inflammatory pelvic disease | Genitourinary | 1.04 | 0.94 | 1.14 | 319224 | 425 | 318799 |
| 480.1 | Bacterial pneumonia | Respiratory | 1.02 | 0.96 | 1.09 | 318628 | 939 | 317689 |

| | | | | | | | | |
|---|---|---|---|---|---|---|---|---|
| 586.4 | Stricture/obstruction of ureter | Genitourinary | 0.98 | 0.91 | 1.04 | 315018 | 939 | 314079 |
| 480 | Pneumonia | Respiratory | 0.99 | 0.96 | 1.02 | 321603 | 3914 | 317689 |
| 418.1 | Precordial pain | Circulatory System | 1.01 | 0.98 | 1.05 | 299506 | 3686 | 295820 |
| 528.6 | Leukoplakia of oral mucosa | Digestive | 0.96 | 0.86 | 1.07 | 322846 | 312 | 322534 |
| 755.1 | Congenital deformities of feet | Congenital Anomalies | 1.05 | 0.92 | 1.20 | 327531 | 220 | 327311 |
| 625 | Pain and other symptoms associated with female genital organs | Genitourinary | 1.02 | 0.97 | 1.06 | 320718 | 2049 | 318669 |
| 573 | Other disorders of liver | Digestive | 1.01 | 0.98 | 1.04 | 323533 | 5202 | 318331 |
| 702.2 | Seborrheic keratosis | Dermatologic | 1.01 | 0.98 | 1.05 | 325769 | 3169 | 322600 |
| 375.2 | Epiphora | Sense Organs | 0.98 | 0.91 | 1.04 | 289094 | 902 | 288192 |
| 334 | Degenerative disease of the spinal cord | Neurological | 0.98 | 0.93 | 1.03 | 287631 | 1479 | 286152 |
| 610.8 | Other specified benign mammary dysplasias | Genitourinary | 0.97 | 0.90 | 1.05 | 321576 | 703 | 320873 |
| 513.4 | Hyperventilation | Respiratory | 1.04 | 0.93 | 1.17 | 327904 | 280 | 327624 |
| 451 | Phlebitis and thrombophlebitis | Circulatory System | 0.96 | 0.87 | 1.07 | 288110 | 397 | 287713 |
| 686.1 | Carbuncle and furuncle | Dermatologic | 1.01 | 0.97 | 1.06 | 318829 | 2422 | 316407 |
| 316 | Substance addiction and disorders | Mental Disorders | 0.96 | 0.87 | 1.07 | 298042 | 373 | 297669 |
| 384 | Other disorders of tympanic membrane | Sense Organs | 0.97 | 0.88 | 1.06 | 324573 | 430 | 324143 |
| 701.4 | Keloid scar | Dermatologic | 1.05 | 0.92 | 1.19 | 323292 | 228 | 323064 |
| 695.42 | Systemic lupus erythematosus | Dermatologic | 0.96 | 0.87 | 1.07 | 320784 | 363 | 320421 |
| 872 | Traumatic amputation | Injuries & Poisonings | 0.97 | 0.88 | 1.06 | 320213 | 482 | 319731 |
| 241.1 | Nontoxic uninodular goiter | Endocrine/Metabolic | 0.97 | 0.89 | 1.06 | 310840 | 511 | 310329 |
| 686 | Other local infections of skin and subcutaneous tissue | Dermatologic | 0.99 | 0.97 | 1.01 | 327410 | 11003 | 316407 |
| 738 | Other acquired musculoskeletal deformity | Musculoskeletal | 0.97 | 0.88 | 1.06 | 317104 | 417 | 316687 |
| 495.2 | Asthma with exacerbation | Respiratory | 1.04 | 0.93 | 1.18 | 294453 | 261 | 294192 |
| 721.1 | Spondylosis without myelopathy | Musculoskeletal | 1.02 | 0.96 | 1.08 | 312014 | 1070 | 310944 |
| 210 | Benign neoplasm of lip, oral cavity, and pharynx | Neoplasms | 0.98 | 0.92 | 1.04 | 327034 | 993 | 326041 |
| 443.7 | Peripheral angiopathy in diseases classified elsewhere | Circulatory System | 0.97 | 0.88 | 1.07 | 319775 | 415 | 319360 |
| 204 | Leukemia | Neoplasms | 0.97 | 0.88 | 1.06 | 323984 | 461 | 323523 |
| 626.12 | Excessive or frequent menstruation | Genitourinary | 1.01 | 0.99 | 1.03 | 306272 | 10041 | 296231 |
| 174 | Breast cancer | Neoplasms | 0.96 | 0.85 | 1.08 | 304712 | 288 | 304424 |
| 496 | Chronic airway obstruction | Respiratory | 0.99 | 0.97 | 1.02 | 301827 | 7635 | 294192 |
| 979 | Adverse drug events and drug allergies | Injuries & Poisonings | 0.97 | 0.90 | 1.05 | 300927 | 716 | 300211 |
| 385.3 | Cholesteatoma | Sense Organs | 1.03 | 0.95 | 1.11 | 324732 | 589 | 324143 |
| 252.1 | Hyperparathyroidism | Endocrine/Metabolic | 1.02 | 0.96 | 1.10 | 325359 | 814 | 324545 |
| 365 | Glaucoma | Sense Organs | 0.99 | 0.96 | 1.02 | 321340 | 4558 | 316782 |
| 371 | Inflammation of the eye | Sense Organs | 0.97 | 0.88 | 1.07 | 318796 | 408 | 318388 |
| 947 | Urticaria | Injuries & Poisonings | 1.03 | 0.94 | 1.14 | 322293 | 387 | 321906 |
| 605 | Erectile dysfunction [ED] | Genitourinary | 0.96 | 0.86 | 1.08 | 308197 | 291 | 307906 |
| 568.1 | Peritoneal adhesions (postoperative) (postinfection) | Digestive | 0.99 | 0.95 | 1.02 | 249757 | 3097 | 246660 |

| | | | | | | | | |
|---|---|---|---|---|---|---|---|---|
| 420.2 | Pericarditis | Circulatory System | 1.02 | 0.96 | 1.08 | 326070 | 1087 | 324983 |
| 276.11 | Hyperosmolality and/or hypernatremia | Endocrine/Metabolic | 1.04 | 0.92 | 1.18 | 320891 | 247 | 320644 |
| 558 | Noninfectious gastroenteritis | Digestive | 1.00 | 0.98 | 1.01 | 278260 | 19864 | 258396 |
| 41 | Bacterial infection NOS | Infectious Diseases | 1.01 | 0.99 | 1.03 | 323384 | 10752 | 312632 |
| 735.21 | Hammer toe (acquired) | Musculoskeletal | 0.99 | 0.94 | 1.03 | 318701 | 2014 | 316687 |
| 592.12 | Chronic cystitis | Genitourinary | 1.02 | 0.96 | 1.09 | 297948 | 913 | 297035 |
| 8.5 | Bacterial enteritis | Infectious Diseases | 0.99 | 0.94 | 1.03 | 321188 | 2109 | 319079 |
| 455 | Hemorrhoids | Circulatory System | 1.00 | 0.99 | 1.02 | 312410 | 24697 | 287713 |
| 172.2 | Other non-epithelial cancer of skin | Neoplasms | 1.01 | 0.99 | 1.03 | 325462 | 11242 | 314220 |
| 433.12 | Cerebral atherosclerosis | Circulatory System | 0.96 | 0.84 | 1.09 | 318345 | 238 | 318107 |
| 801.1 | Fracture of foot | Injuries & Poisonings | 1.03 | 0.94 | 1.14 | 323975 | 418 | 323557 |
| 618.5 | Prolapse of vaginal vault after hysterectomy | Genitourinary | 0.97 | 0.89 | 1.06 | 316743 | 501 | 316242 |
| 217.1 | Nevus, non-neoplastic | Neoplasms | 0.97 | 0.90 | 1.06 | 320346 | 597 | 319749 |
| 531.2 | Gastric ulcer | Digestive | 0.99 | 0.96 | 1.02 | 324777 | 4228 | 320549 |
| 285.1 | Acute posthemorrhagic anemia | Hematopoietic | 0.96 | 0.85 | 1.09 | 309167 | 267 | 308900 |
| 595 | Hydronephrosis | Genitourinary | 0.99 | 0.94 | 1.03 | 321941 | 2010 | 319931 |
| 728.71 | Contracture of palmar fascia [Dupuytren's disease] | Musculoskeletal | 0.99 | 0.95 | 1.03 | 307403 | 2449 | 304954 |
| 454.1 | Varicose veins of lower extremity | Circulatory System | 0.99 | 0.98 | 1.01 | 299275 | 11562 | 287713 |
| 244.1 | Secondary hypothyroidism | Endocrine/Metabolic | 1.02 | 0.96 | 1.08 | 311484 | 1155 | 310329 |
| 446.5 | Giant cell arteritis | Circulatory System | 0.97 | 0.88 | 1.07 | 319750 | 390 | 319360 |
| 386.2 | Peripheral or central vertigo | Sense Organs | 1.03 | 0.93 | 1.14 | 322036 | 387 | 321649 |
| 535.1 | Acute gastritis | Digestive | 0.98 | 0.93 | 1.04 | 297704 | 1193 | 296511 |
| 577.3 | Cyst and pseudocyst of pancreas | Digestive | 1.03 | 0.93 | 1.14 | 326295 | 375 | 325920 |
| 345.1 | Epilepsy | Neurological | 0.97 | 0.87 | 1.08 | 286483 | 331 | 286152 |
| 367.9 | Blindness and low vision | Sense Organs | 0.98 | 0.91 | 1.05 | 326510 | 756 | 325754 |
| 202 | Cancer of other lymphoid, histiocytic tissue | Neoplasms | 0.98 | 0.93 | 1.04 | 324661 | 1138 | 323523 |
| 389.1 | Sensorineural hearing loss | Sense Organs | 1.03 | 0.94 | 1.13 | 318658 | 464 | 318194 |
| 727.5 | Rupture of synovium | Musculoskeletal | 0.97 | 0.87 | 1.08 | 305298 | 344 | 304954 |
| 610.2 | Fibroadenosis of breast | Genitourinary | 0.97 | 0.86 | 1.08 | 321189 | 316 | 320873 |
| 389.4 | Tinnitus | Sense Organs | 0.97 | 0.89 | 1.06 | 318735 | 541 | 318194 |
| 175 | Acquired absence of breast | Neoplasms | 0.99 | 0.95 | 1.03 | 305907 | 2516 | 303391 |
| 199 | Neoplasm of uncertain behavior | Neoplasms | 1.02 | 0.96 | 1.08 | 230468 | 1156 | 229312 |
| 242.1 | Graves' disease | Endocrine/Metabolic | 1.03 | 0.94 | 1.13 | 310789 | 460 | 310329 |
| 531.4 | Peptic ulcer, site unspecified | Digestive | 0.97 | 0.89 | 1.07 | 320998 | 449 | 320549 |
| 368.1 | Amblyopia | Sense Organs | 1.03 | 0.94 | 1.11 | 325423 | 541 | 324882 |
| 615 | Endometriosis | Genitourinary | 0.99 | 0.96 | 1.02 | 322888 | 4089 | 318799 |
| 578.1 | Hematemesis | Digestive | 1.01 | 0.97 | 1.06 | 305428 | 2034 | 303394 |
| 324 | Other CNS infection and poliomyelitis | Neurological | 0.97 | 0.86 | 1.09 | 327426 | 288 | 327138 |

| | | | | | | | | |
|---|---|---|---|---|---|---|---|---|
| 509.2 | Respiratory insufficiency | Respiratory | 0.99 | 0.94 | 1.03 | 318272 | 1788 | 316484 |
| 624.2 | Atrophy of female genital tract | Genitourinary | 1.03 | 0.94 | 1.13 | 319111 | 442 | 318669 |
| 716.2 | Unspecified monoarthritis | Musculoskeletal | 1.00 | 0.98 | 1.01 | 289797 | 15901 | 273896 |
| 198.1 | Secondary malignancy of lymph nodes | Neoplasms | 0.99 | 0.97 | 1.02 | 234815 | 5503 | 229312 |
| 960.2 | Allergy/adverse effect of penicillin | Injuries & Poisonings | 1.00 | 0.98 | 1.01 | 316894 | 16683 | 300211 |
| 440.9 | Atherosclerosis of aorta | Circulatory System | 1.04 | 0.91 | 1.19 | 319562 | 202 | 319360 |
| 618.2 | Uterine/Uterovaginal prolapse | Genitourinary | 0.99 | 0.97 | 1.02 | 321713 | 5471 | 316242 |
| 803.3 | Fracture of clavicle or scapula | Injuries & Poisonings | 0.97 | 0.86 | 1.09 | 323857 | 300 | 323557 |
| 191 | Malignant and unknown neoplasms of brain and nervous system | Neoplasms | 0.96 | 0.84 | 1.10 | 326662 | 215 | 326447 |
| 367.1 | Myopia | Sense Organs | 0.98 | 0.93 | 1.04 | 327025 | 1271 | 325754 |
| 750 | Digestive congenital anomalies | Congenital Anomalies | 1.02 | 0.95 | 1.10 | 326705 | 702 | 326003 |
| 337 | Disorders of the autonomic nervous system | Neurological | 1.04 | 0.91 | 1.19 | 286359 | 207 | 286152 |
| 535.8 | Other specified gastritis | Digestive | 0.99 | 0.97 | 1.02 | 304926 | 8415 | 296511 |
| 714 | Rheumatoid arthritis and other inflammatory polyarthropathies | Musculoskeletal | 1.01 | 0.98 | 1.03 | 325905 | 8652 | 317253 |
| 803.1 | Fracture of humerus | Injuries & Poisonings | 0.97 | 0.86 | 1.09 | 323850 | 293 | 323557 |
| 443.9 | Peripheral vascular disease, unspecified | Circulatory System | 1.01 | 0.97 | 1.05 | 321969 | 2609 | 319360 |
| 458.1 | Orthostatic hypotension | Circulatory System | 0.98 | 0.93 | 1.04 | 195553 | 1374 | 194179 |
| 555 | Inflammatory bowel disease and other gastroenteritis and colitis | Digestive | 1.00 | 0.98 | 1.01 | 278195 | 19799 | 258396 |
| 375 | Disorders of lacrimal system | Sense Organs | 1.01 | 0.97 | 1.06 | 289852 | 1660 | 288192 |
| 751.1 | Congenital anomalies of genital organs | Congenital Anomalies | 0.98 | 0.91 | 1.05 | 326709 | 706 | 326003 |
| 500.2 | Pneumoconiosis | Respiratory | 0.98 | 0.92 | 1.05 | 317485 | 1001 | 316484 |
| 411.2 | Myocardial infarction | Circulatory System | 0.99 | 0.98 | 1.01 | 307658 | 11969 | 295689 |
| 276.13 | Hyperpotassemia | Endocrine/Metabolic | 1.02 | 0.96 | 1.08 | 321623 | 979 | 320644 |
| 556.1 | Ulceration of intestine | Digestive | 0.98 | 0.91 | 1.06 | 259087 | 691 | 258396 |
| 153.2 | Colon cancer | Neoplasms | 0.99 | 0.96 | 1.03 | 304402 | 3122 | 301280 |
| 364 | Corneal opacity and other disorders of cornea | Sense Organs | 1.04 | 0.91 | 1.18 | 317009 | 227 | 316782 |
| 340 | Migraine | Neurological | 1.01 | 0.97 | 1.05 | 320723 | 2773 | 317950 |
| 741.4 | Joint effusions | Musculoskeletal | 0.97 | 0.86 | 1.09 | 310925 | 283 | 310642 |
| 960 | Poisoning by antibiotics | Injuries & Poisonings | 0.99 | 0.96 | 1.02 | 303739 | 3528 | 300211 |
| 512.2 | Painful respiration | Respiratory | 0.96 | 0.85 | 1.10 | 314496 | 228 | 314268 |
| 323 | Encephalitis | Neurological | 0.98 | 0.92 | 1.05 | 327999 | 861 | 327138 |
| 334.2 | Anterior horn cell disease | Neurological | 0.96 | 0.84 | 1.10 | 286376 | 224 | 286152 |
| 618 | Genital prolapse | Genitourinary | 0.98 | 0.90 | 1.06 | 316862 | 620 | 316242 |
| 442.8 | Aneurysm of other specified artery | Circulatory System | 1.04 | 0.91 | 1.19 | 319570 | 210 | 319360 |
| 599.9 | Other abnormality of urination | Genitourinary | 0.99 | 0.94 | 1.03 | 222371 | 1944 | 220427 |
| 200.1 | Polycythemia vera | Neoplasms | 1.03 | 0.93 | 1.13 | 318136 | 404 | 317732 |
| 526.41 | Temporomandibular joint disorder, unspecified | Digestive | 0.96 | 0.85 | 1.10 | 311886 | 228 | 311658 |
| 529 | Diseases and other conditions of the tongue | Digestive | 1.02 | 0.95 | 1.08 | 323485 | 951 | 322534 |

| | | | | | | | | |
|---|---|---|---|---|---|---|---|---|
| 752.11 | Spina bifida | Congenital Anomalies | 0.96 | 0.84 | 1.11 | 327886 | 211 | 327675 |
| 587 | Kidney replaced by transpant | Genitourinary | 1.03 | 0.93 | 1.13 | 314477 | 398 | 314079 |
| 427.6 | Premature beats | Circulatory System | 1.02 | 0.94 | 1.12 | 299979 | 458 | 299521 |
| 593 | Hematuria | Genitourinary | 1.00 | 0.98 | 1.01 | 313795 | 16760 | 297035 |
| 473.3 | Paralysis/spasm of vocal cords or larynx | Respiratory | 1.03 | 0.92 | 1.16 | 309217 | 267 | 308950 |
| 512.8 | Cough | Respiratory | 1.01 | 0.97 | 1.05 | 317232 | 2964 | 314268 |
| 292 | Neurological disorders | Mental Disorders | 1.03 | 0.93 | 1.12 | 321919 | 449 | 321470 |
| 941 | Adverse reaction to serum or vaccine | Injuries & Poisonings | 0.96 | 0.84 | 1.10 | 322128 | 222 | 321906 |
| 149 | Cancer of larynx, pharynx, nasal cavities | Neoplasms | 0.98 | 0.92 | 1.05 | 326986 | 945 | 326041 |
| 272.1 | Hyperlipidemia | Endocrine/Metabolic | 1.01 | 0.98 | 1.04 | 296728 | 5231 | 291497 |
| 300.1 | Anxiety disorder | Mental Disorders | 0.99 | 0.97 | 1.02 | 288368 | 5470 | 282898 |
| 579 | Other symptoms involving abdomen and pelvis | Digestive | 1.02 | 0.96 | 1.07 | 304580 | 1186 | 303394 |
| 771.1 | Swelling of limb | Symptoms | 1.02 | 0.95 | 1.09 | 327356 | 842 | 326514 |
| 618.1 | Prolapse of vaginal walls | Genitourinary | 0.99 | 0.97 | 1.02 | 323726 | 7484 | 316242 |
| 382 | Otalgia | Sense Organs | 0.97 | 0.87 | 1.09 | 324458 | 315 | 324143 |
| 613.8 | Other specified disorders of breast | Genitourinary | 1.03 | 0.93 | 1.14 | 322773 | 355 | 322418 |
| 726 | Peripheral enthesopathies and allied syndromes | Musculoskeletal | 1.01 | 0.98 | 1.03 | 313722 | 8768 | 304954 |
| 275.5 | Disorders of calcium/phosphorus metabolism | Endocrine/Metabolic | 0.98 | 0.93 | 1.05 | 327144 | 1109 | 326035 |
| 320 | Meningitis | Neurological | 0.98 | 0.89 | 1.07 | 327577 | 439 | 327138 |
| 440 | Atherosclerosis | Circulatory System | 1.04 | 0.90 | 1.19 | 319564 | 204 | 319360 |
| 727.1 | Synovitis and tenosynovitis | Musculoskeletal | 0.98 | 0.91 | 1.06 | 305641 | 687 | 304954 |
| 636.2 | Early onset of delivery | Pregnancy Complications | 1.02 | 0.94 | 1.10 | 320594 | 629 | 319965 |
| 830 | Dislocation | Injuries & Poisonings | 0.99 | 0.95 | 1.03 | 323927 | 2154 | 321773 |
| 535.6 | Duodenitis | Digestive | 1.01 | 0.98 | 1.03 | 304405 | 7894 | 296511 |
| 550.5 | Ventral hernia | Digestive | 0.99 | 0.96 | 1.03 | 283371 | 3582 | 279789 |
| 707.1 | Decubitus ulcer | Dermatologic | 0.97 | 0.87 | 1.09 | 327025 | 326 | 326699 |
| 575.7 | Other disorders of gallbladder | Digestive | 0.99 | 0.94 | 1.04 | 311719 | 1396 | 310323 |
| 170.2 | Cancer of connective tissue | Neoplasms | 0.98 | 0.91 | 1.06 | 328122 | 734 | 327388 |
| 374.1 | Ectropion or entropion | Sense Organs | 0.99 | 0.93 | 1.05 | 319480 | 1092 | 318388 |
| 614.52 | Vaginitis and vulvovaginitis | Genitourinary | 1.02 | 0.93 | 1.13 | 319219 | 420 | 318799 |
| 198 | Secondary malignant neoplasm | Neoplasms | 0.99 | 0.93 | 1.05 | 230431 | 1119 | 229312 |
| 805 | Fracture of vertebral column without mention of spinal cord injury | Injuries & Poisonings | 0.97 | 0.87 | 1.09 | 323890 | 333 | 323557 |
| 172.11 | Melanomas of skin | Neoplasms | 0.99 | 0.95 | 1.03 | 316943 | 2723 | 314220 |
| 388 | Other disorders of ear | Sense Organs | 1.00 | 0.98 | 1.02 | 328080 | 9886 | 318194 |
| 559 | Ileostomy status | Digestive | 1.01 | 0.96 | 1.06 | 260069 | 1673 | 258396 |
| 384.4 | Perforation of tympanic membrane | Sense Organs | 0.99 | 0.93 | 1.05 | 325167 | 1024 | 324143 |
| 819 | Skull and face fracture and other intercranial injury | Injuries & Poisonings | 0.99 | 0.94 | 1.04 | 328056 | 1517 | 326539 |
| 751.21 | Cystic kidney disease | Congenital Anomalies | 1.02 | 0.93 | 1.12 | 326461 | 458 | 326003 |

| | | | | | | | | | |
|---|---|---|---|---|---|---|---|---|---|
| 701.2 | Scar conditions and fibrosis of skin | Dermatologic | 1.01 | 0.97 | 1.05 | 325471 | 2407 | 323064 |
| 946 | Anaphylactic shock NOS | Injuries & Poisonings | 0.98 | 0.90 | 1.07 | 322450 | 544 | 321906 |
| 614.1 | Pelvic peritoneal adhesions, female (postoperative) (postinfection) | Genitourinary | 1.01 | 0.97 | 1.05 | 321370 | 2571 | 318799 |
| 686.3 | Pilonidal cyst | Dermatologic | 1.02 | 0.94 | 1.10 | 317024 | 617 | 316407 |
| 550 | Abdominal hernia | Digestive | 1.00 | 0.99 | 1.01 | 328240 | 48451 | 279789 |
| 446.9 | Arteritis NOS | Circulatory System | 0.97 | 0.86 | 1.10 | 319605 | 245 | 319360 |
| 447 | Other disorders of arteries and arterioles | Circulatory System | 1.02 | 0.94 | 1.11 | 319916 | 556 | 319360 |
| 246 | Other disorders of thyroid | Endocrine/Metabolic | 1.00 | 0.99 | 1.02 | 327738 | 17409 | 310329 |
| 619.1 | Noninflammatory disorders of ovary, fallopian tube, and broad ligament | Genitourinary | 1.02 | 0.95 | 1.09 | 279282 | 734 | 278548 |
| 523.31 | Acute periodontitis | Digestive | 0.98 | 0.91 | 1.06 | 312371 | 713 | 311658 |
| 614.5 | Inflammatory disease of cervix, vagina, and vulva | Genitourinary | 0.98 | 0.87 | 1.09 | 319133 | 334 | 318799 |
| 352.1 | Trigeminal nerve disorders [CN5] | Neurological | 0.98 | 0.89 | 1.07 | 313473 | 462 | 313011 |
| 184.2 | Cancer of other female genital organs | Neoplasms | 1.02 | 0.92 | 1.14 | 305876 | 323 | 305553 |
| 560.3 | Peritoneal or intestinal adhesions | Digestive | 0.98 | 0.92 | 1.05 | 259257 | 861 | 258396 |
| 721 | Spondylosis and allied disorders | Musculoskeletal | 0.99 | 0.92 | 1.05 | 311869 | 925 | 310944 |
| 345.12 | Partial epilepsy | Neurological | 0.97 | 0.87 | 1.09 | 286450 | 298 | 286152 |
| 801 | Fracture of ankle and foot | Injuries & Poisonings | 0.97 | 0.86 | 1.10 | 323808 | 251 | 323557 |
| 220 | Benign neoplasm of ovary | Neoplasms | 0.99 | 0.94 | 1.04 | 297546 | 1490 | 296056 |
| 361.1 | Retinal detachment with retinal defect | Sense Organs | 0.99 | 0.94 | 1.04 | 318238 | 1456 | 316782 |
| 157 | Pancreatic cancer | Neoplasms | 1.02 | 0.94 | 1.10 | 312933 | 602 | 312331 |
| 281.13 | Folate-deficiency anemia | Hematopoietic | 0.97 | 0.84 | 1.12 | 309101 | 201 | 308900 |
| 338.1 | Acute pain | Neurological | 0.98 | 0.89 | 1.08 | 327846 | 420 | 327426 |
| 681.1 | Cellulitis and abscess of fingers/toes | Dermatologic | 0.98 | 0.90 | 1.07 | 316989 | 582 | 316407 |
| 526.1 | Cysts of the jaws | Digestive | 0.98 | 0.88 | 1.09 | 311993 | 335 | 311658 |
| 722.9 | Other and unspecified disc disorder | Musculoskeletal | 0.99 | 0.96 | 1.02 | 314894 | 3950 | 310944 |
| 840 | Sprains and strains | Injuries & Poisonings | 1.02 | 0.93 | 1.11 | 328201 | 481 | 327720 |
| 442.1 | Aortic aneurysm | Circulatory System | 0.98 | 0.91 | 1.07 | 319946 | 586 | 319360 |
| 535.2 | Atrophic gastritis | Digestive | 1.02 | 0.92 | 1.13 | 296869 | 358 | 296511 |
| 366.2 | Senile cataract | Sense Organs | 1.00 | 0.97 | 1.02 | 315842 | 8458 | 307384 |
| 614.53 | Cyst or abscess of Bartholin's gland | Genitourinary | 1.01 | 0.95 | 1.09 | 319602 | 803 | 318799 |
| 634.1 | Missed abortion/Hydatidiform mole | Pregnancy Complications | 1.01 | 0.96 | 1.07 | 321168 | 1203 | 319965 |
| 703.1 | Ingrowing nail | Dermatologic | 1.01 | 0.95 | 1.08 | 322439 | 1010 | 321429 |
| 689 | Disorder of skin and subcutaneous tissue NOS | Dermatologic | 1.00 | 0.99 | 1.01 | 328240 | 42194 | 286046 |
| 275.1 | Disorders of iron metabolism | Hematopoietic | 0.98 | 0.91 | 1.06 | 326735 | 700 | 326035 |
| 251.1 | Hypoglycemia | Endocrine/Metabolic | 0.99 | 0.93 | 1.05 | 305952 | 977 | 304975 |
| 348.2 | Cerebral edema and compression of brain | Neurological | 0.98 | 0.86 | 1.10 | 286412 | 260 | 286152 |
| 740.9 | Osteoarthrosis NOS | Musculoskeletal | 1.01 | 0.98 | 1.04 | 310757 | 4432 | 306325 |
| 522 | Diseases of pulp and periapical tissues | Digestive | 0.98 | 0.91 | 1.06 | 312297 | 639 | 311658 |

| | | | | | | | | |
|---|---|---|---|---|---|---|---|---|
| 701.5 | Abnormal granulation tissue | Dermatologic | 0.98 | 0.90 | 1.08 | 323543 | 479 | 323064 |
| 717 | Polymyalgia Rheumatica | Musculoskeletal | 0.99 | 0.93 | 1.05 | 328240 | 1147 | 327093 |
| 262 | Mineral deficiency NEC | Endocrine/Metabolic | 1.03 | 0.90 | 1.16 | 326027 | 241 | 325786 |
| 136 | Other infectious and parasitic diseases | Infectious Diseases | 0.98 | 0.89 | 1.08 | 327982 | 428 | 327554 |
| 365.11 | Primary open angle glaucoma | Sense Organs | 1.01 | 0.95 | 1.07 | 317838 | 1056 | 316782 |
| 8 | Intestinal infection | Infectious Diseases | 1.00 | 0.98 | 1.02 | 328240 | 9161 | 319079 |
| 586 | Other disorders of the kidney and ureters | Genitourinary | 0.99 | 0.96 | 1.03 | 317490 | 3411 | 314079 |
| 300 | Anxiety, phobic and dissociative disorders | Mental Disorders | 0.98 | 0.88 | 1.09 | 283261 | 363 | 282898 |
| 261.2 | Vitamin B-complex deficiencies | Endocrine/Metabolic | 1.01 | 0.94 | 1.09 | 326559 | 773 | 325786 |
| 578.2 | Blood in stool | Digestive | 0.99 | 0.96 | 1.03 | 306143 | 2749 | 303394 |
| 715.2 | Ankylosing spondylitis | Musculoskeletal | 1.02 | 0.92 | 1.13 | 317637 | 384 | 317253 |
| 389 | Hearing loss | Sense Organs | 1.01 | 0.97 | 1.04 | 321364 | 3170 | 318194 |
| 327.3 | Sleep apnea | Neurological | 1.01 | 0.98 | 1.04 | 327419 | 4699 | 322720 |
| 502 | Postinflammatory pulmonary fibrosis | Respiratory | 1.01 | 0.95 | 1.08 | 317367 | 883 | 316484 |
| 274.1 | Gout | Endocrine/Metabolic | 1.01 | 0.96 | 1.06 | 327930 | 1668 | 326262 |
| 870.4 | Open wound of nose and sinus | Injuries & Poisonings | 0.98 | 0.86 | 1.11 | 319969 | 238 | 319731 |
| 444 | Arterial embolism and thrombosis | Circulatory System | 0.98 | 0.89 | 1.08 | 319794 | 434 | 319360 |
| 967 | Adverse effects of sedatives or other central nervous system depressants and anesthetics | Injuries & Poisonings | 0.98 | 0.91 | 1.07 | 300790 | 579 | 300211 |
| 153.3 | Malignant neoplasm of rectum, rectosigmoid junction, and anus | Neoplasms | 1.01 | 0.97 | 1.05 | 303397 | 2117 | 301280 |
| 580.2 | Nephrotic syndrome without mention of glomerulonephritis | Genitourinary | 1.02 | 0.93 | 1.11 | 314575 | 496 | 314079 |
| 590 | Pyelonephritis | Genitourinary | 1.01 | 0.97 | 1.04 | 300960 | 3925 | 297035 |
| 523.32 | Chronic periodontitis | Digestive | 0.99 | 0.91 | 1.07 | 312248 | 590 | 311658 |
| 635.2 | Antepartum hemorrhage, abruptio placentae, and placenta previa | Pregnancy Complications | 1.01 | 0.95 | 1.08 | 320901 | 936 | 319965 |
| 479 | Other upper respiratory disease | Respiratory | 1.00 | 0.99 | 1.02 | 324241 | 15291 | 308950 |
| 957 | Injury to other and unspecified nerves | Injuries & Poisonings | 0.98 | 0.87 | 1.10 | 328157 | 275 | 327882 |
| 198.4 | Secondary malignant neoplasm of liver | Neoplasms | 0.99 | 0.96 | 1.03 | 232009 | 2697 | 229312 |
| 200 | Myeloproliferative disease | Neoplasms | 1.01 | 0.94 | 1.09 | 324202 | 679 | 323523 |
| 727.4 | Ganglion and cyst of synovium, tendon, and bursa | Musculoskeletal | 1.01 | 0.97 | 1.04 | 307936 | 2982 | 304954 |
| 790.6 | Other abnormal blood chemistry | Symptoms | 1.00 | 0.97 | 1.02 | 328029 | 6529 | 321500 |
| 697 | Sarcoidosis | Dermatologic | 0.99 | 0.91 | 1.07 | 322309 | 560 | 321749 |
| 250.11 | Type 1 diabetes with ketoacidosis | Endocrine/Metabolic | 0.98 | 0.86 | 1.11 | 307745 | 255 | 307490 |
| 737.3 | Kyphoscoliosis and scoliosis | Musculoskeletal | 1.02 | 0.92 | 1.13 | 317058 | 371 | 316687 |
| 785 | Abdominal pain | Symptoms | 1.00 | 0.99 | 1.01 | 328240 | 42311 | 285929 |
| 601.4 | Balanoposthitis | Genitourinary | 0.98 | 0.87 | 1.10 | 307689 | 296 | 307393 |
| 791 | Gangrene | Symptoms | 1.01 | 0.93 | 1.10 | 328240 | 579 | 327661 |
| 458 | Hypotension | Circulatory System | 0.99 | 0.93 | 1.05 | 195260 | 1081 | 194179 |
| 578.9 | Hemorrhage of gastrointestinal tract | Digestive | 1.00 | 0.98 | 1.03 | 308849 | 5455 | 303394 |
| 216 | Benign neoplasm of skin | Neoplasms | 1.00 | 0.98 | 1.03 | 327614 | 7865 | 319749 |

| | | | | | | | | |
|---|---|---|---|---|---|---|---|---|
| 193 | Thyroid cancer | Neoplasms | 1.02 | 0.92 | 1.13 | 327009 | 367 | 326642 |
| 591 | Urinary tract infection | Genitourinary | 1.00 | 0.99 | 1.02 | 309711 | 12676 | 297035 |
| 624.1 | Dystrophy of female genital tract | Genitourinary | 1.02 | 0.90 | 1.16 | 318909 | 240 | 318669 |
| 571.5 | Other chronic nonalcoholic liver disease | Digestive | 1.01 | 0.96 | 1.06 | 320004 | 1673 | 318331 |
| 575.9 | Nonspecific abnormal findings on radiological and other examination of biliary tract | Digestive | 1.02 | 0.92 | 1.13 | 310690 | 367 | 310323 |
| 560.1 | Paralytic ileus | Digestive | 0.99 | 0.90 | 1.07 | 258916 | 520 | 258396 |
| 949 | Allergies, other | Injuries & Poisonings | 1.02 | 0.92 | 1.12 | 322326 | 420 | 321906 |
| 601.11 | Acute prostatitis | Genitourinary | 1.02 | 0.90 | 1.15 | 307653 | 260 | 307393 |
| 565.1 | Anal and rectal polyp | Digestive | 1.00 | 0.97 | 1.02 | 254329 | 7669 | 246660 |
| 721.8 | Other allied disorders of spine | Musculoskeletal | 1.01 | 0.97 | 1.04 | 314171 | 3227 | 310944 |
| 740.1 | Osteoarthritis; localized | Musculoskeletal | 1.00 | 0.98 | 1.02 | 315797 | 9472 | 306325 |
| 426.32 | Left bundle branch block | Circulatory System | 0.99 | 0.95 | 1.04 | 301302 | 1781 | 299521 |
| 536 | Disorders of function of stomach | Digestive | 1.02 | 0.90 | 1.15 | 296768 | 257 | 296511 |
| 614 | Inflammatory diseases of female pelvic organs | Genitourinary | 0.98 | 0.89 | 1.09 | 319173 | 374 | 318799 |
| 292.4 | Altered mental status | Mental Disorders | 1.01 | 0.97 | 1.05 | 323742 | 2272 | 321470 |
| 540.11 | Acute appendicitis | Digestive | 0.99 | 0.96 | 1.03 | 327037 | 2658 | 324379 |
| 709.2 | Sicca syndrome | Dermatologic | 1.01 | 0.93 | 1.11 | 242486 | 520 | 241966 |
| 289.8 | Polycythemia vera, secondary | Hematopoietic | 0.98 | 0.87 | 1.10 | 320039 | 291 | 319748 |
| 401.22 | Hypertensive chronic kidney disease | Circulatory System | 1.01 | 0.96 | 1.06 | 250343 | 1595 | 248748 |
| 447.1 | Stricture of artery | Circulatory System | 0.99 | 0.93 | 1.06 | 320246 | 886 | 319360 |
| 870.3 | Other open wound of head and face | Injuries & Poisonings | 0.99 | 0.96 | 1.03 | 322782 | 3051 | 319731 |
| 420.3 | Endocarditis | Circulatory System | 0.99 | 0.92 | 1.07 | 325668 | 685 | 324983 |
| 198.5 | Secondary malignancy of brain/spine | Neoplasms | 0.99 | 0.92 | 1.06 | 230113 | 801 | 229312 |
| 567 | Peritonitis and retroperitoneal infections | Digestive | 1.01 | 0.95 | 1.08 | 247550 | 890 | 246660 |
| 743.11 | Osteoporosis NOS | Musculoskeletal | 1.01 | 0.97 | 1.04 | 327235 | 3036 | 324199 |
| 635.3 | Placenta previa and abruptio placenta | Pregnancy Complications | 1.01 | 0.95 | 1.07 | 321301 | 1336 | 319965 |
| 159 | Malignant neoplasm of other and ill-defined sites within the digestive organs and peritoneum | Neoplasms | 1.00 | 0.97 | 1.02 | 317620 | 5289 | 312331 |
| 512 | Other symptoms of respiratory system | Respiratory | 1.00 | 0.97 | 1.02 | 319772 | 5504 | 314268 |
| 530.11 | GERD | Digestive | 1.00 | 0.98 | 1.01 | 302292 | 14489 | 287803 |
| 507 | Pleurisy; pleural effusion | Respiratory | 1.00 | 0.98 | 1.03 | 323091 | 6607 | 316484 |
| 619.3 | Noninflammatory disorders of cervix | Genitourinary | 0.99 | 0.96 | 1.03 | 281393 | 2845 | 278548 |
| 523 | Gingival and periodontal diseases | Digestive | 1.01 | 0.93 | 1.10 | 312187 | 529 | 311658 |
| 530.2 | Esophageal bleeding (varices/hemorrhage) | Digestive | 1.01 | 0.96 | 1.06 | 289525 | 1722 | 287803 |
| 426.24 | Atrioventricular block, complete | Circulatory System | 1.01 | 0.93 | 1.10 | 300112 | 591 | 299521 |
| 557.1 | Celiac disease | Digestive | 1.01 | 0.96 | 1.05 | 260290 | 1894 | 258396 |
| 378.2 | Nystagmus and other irregular eye movements | Sense Organs | 1.02 | 0.89 | 1.17 | 288407 | 215 | 288192 |
| 781 | Symptoms involving nervous and musculoskeletal systems | Symptoms | 1.00 | 0.98 | 1.01 | 328238 | 22180 | 306058 |
| 348.9 | Other conditions of brain, NOS | Neurological | 0.99 | 0.89 | 1.09 | 286559 | 407 | 286152 |

| | | | | | | | | |
|---|---|---|---|---|---|---|---|---|
| 293.1 | Swelling, mass, or lump in head and neck [Space-occupying lesion, intracranial NOS] | Mental Disorders | 0.99 | 0.93 | 1.06 | 326044 | 925 | 325119 |
| 701 | Other hypertrophic and atrophic conditions of skin | Dermatologic | 0.99 | 0.95 | 1.04 | 324891 | 1827 | 323064 |
| 333 | Extrapyramidal disease and abnormal movement disorders | Neurological | 1.01 | 0.96 | 1.05 | 288187 | 2035 | 286152 |
| 379 | Other disorders of eye | Sense Organs | 1.00 | 0.99 | 1.01 | 328190 | 39998 | 288192 |
| 729.1 | Rheumatism, unspecified and fibrositis | Musculoskeletal | 1.01 | 0.92 | 1.12 | 305341 | 387 | 304954 |
| 555.1 | Regional enteritis | Digestive | 0.99 | 0.95 | 1.04 | 260201 | 1805 | 258396 |
| 300.12 | Agorophobia, social phobia, and panic disorder | Mental Disorders | 1.01 | 0.94 | 1.09 | 283642 | 744 | 282898 |
| 646 | Other complications of pregnancy NEC | Pregnancy Complications | 1.01 | 0.96 | 1.05 | 328240 | 2396 | 325844 |
| 218.2 | Other benign neoplasm of uterus | Neoplasms | 0.99 | 0.89 | 1.09 | 308143 | 363 | 307780 |
| 592.1 | Cystitis | Genitourinary | 1.01 | 0.96 | 1.05 | 299089 | 2054 | 297035 |
| 990 | Effects radiation NOS | Injuries & Poisonings | 1.00 | 0.96 | 1.03 | 325633 | 3244 | 322389 |
| 211 | Benign neoplasm of other parts of digestive system | Neoplasms | 1.00 | 0.97 | 1.02 | 319683 | 5375 | 314308 |
| 285.2 | Anemia of chronic disease | Hematopoietic | 1.02 | 0.91 | 1.14 | 309194 | 294 | 308900 |
| 634 | Miscarriage; stillbirth | Pregnancy Complications | 1.00 | 0.97 | 1.04 | 324534 | 4569 | 319965 |
| 735.2 | Acquired toe deformities | Musculoskeletal | 1.01 | 0.96 | 1.05 | 318913 | 2226 | 316687 |
| 458.9 | Hypotension NOS | Circulatory System | 1.00 | 0.96 | 1.03 | 197817 | 3638 | 194179 |
| 70.9 | Hepatitis NOS | Infectious Diseases | 1.01 | 0.92 | 1.11 | 322658 | 452 | 322206 |
| 381.1 | Otitis media | Sense Organs | 0.99 | 0.94 | 1.05 | 325296 | 1153 | 324143 |
| 596.5 | Functional disorders of bladder | Genitourinary | 1.01 | 0.96 | 1.06 | 315318 | 1682 | 313636 |
| 573.9 | Abnormal serum enzyme levels | Digestive | 0.98 | 0.86 | 1.12 | 318561 | 230 | 318331 |
| 221 | Benign neoplasm of other female genital organs | Neoplasms | 0.98 | 0.86 | 1.12 | 295974 | 223 | 295751 |
| 368.9 | Subjective visual disturbances | Sense Organs | 1.01 | 0.93 | 1.09 | 325515 | 633 | 324882 |
| 627.4 | Premenopausal menorrhagia | Genitourinary | 0.99 | 0.88 | 1.10 | 296563 | 332 | 296231 |
| 277.4 | Disorders of bilirubin excretion | Endocrine/Metabolic | 0.99 | 0.89 | 1.09 | 283300 | 373 | 282927 |
| 520 | Disorders of tooth development | Digestive | 0.99 | 0.90 | 1.08 | 312128 | 470 | 311658 |
| 531.1 | Hemorrhage from gastrointestinal ulcer | Digestive | 0.99 | 0.92 | 1.07 | 321204 | 655 | 320549 |
| 333.1 | Essential tremor | Neurological | 1.02 | 0.89 | 1.16 | 286370 | 218 | 286152 |
| 288 | Diseases of white blood cells | Hematopoietic | 1.01 | 0.93 | 1.09 | 320928 | 610 | 320318 |
| 596 | Other disorders of bladder | Genitourinary | 1.00 | 0.97 | 1.02 | 321385 | 7749 | 313636 |
| 751.11 | Congenital anomalies of female genital organs | Congenital Anomalies | 1.01 | 0.92 | 1.11 | 326445 | 442 | 326003 |
| 145.2 | Cancer of tongue | Neoplasms | 1.01 | 0.91 | 1.13 | 326366 | 325 | 326041 |
| 716.1 | Unspecified polyarthropathy or polyarthritis | Musculoskeletal | 1.00 | 0.96 | 1.03 | 277426 | 3530 | 273896 |
| 626 | Disorders of menstruation and other abnormal bleeding from female genital tract | Genitourinary | 1.00 | 0.97 | 1.04 | 300178 | 3947 | 296231 |
| 751.2 | Congenital anomalies of urinary system | Congenital Anomalies | 1.01 | 0.94 | 1.08 | 326908 | 905 | 326003 |
| 835 | Internal derangement of knee | Injuries & Poisonings | 1.00 | 0.97 | 1.03 | 326012 | 4239 | 321773 |
| 351 | Other peripheral nerve disorders | Neurological | 1.00 | 0.98 | 1.02 | 325903 | 12892 | 313011 |
| 536.8 | Dyspepsia and other specified disorders of function of stomach | Digestive | 1.02 | 0.90 | 1.15 | 296758 | 247 | 296511 |
| 747.1 | Cardiac congenital anomalies | Congenital Anomalies | 1.02 | 0.89 | 1.16 | 325565 | 211 | 325354 |

| | | | | | | | | | |
|---|---|---|---|---|---|---|---|---|---|
| 706 | Diseases of sebaceous glands | Dermatologic | 1.01 | 0.93 | 1.10 | 318889 | 595 | 318294 |
| 696.41 | Psoriasis vulgaris | Dermatologic | 0.99 | 0.95 | 1.04 | 315655 | 1723 | 313932 |
| 724.2 | Disorders of coccyx | Musculoskeletal | 1.01 | 0.90 | 1.14 | 311226 | 282 | 310944 |
| 470 | Septal Deviations/Turbinate Hypertrophy | Respiratory | 1.00 | 0.98 | 1.03 | 313771 | 4821 | 308950 |
| 426.3 | Bundle branch block | Circulatory System | 0.99 | 0.88 | 1.11 | 299812 | 291 | 299521 |
| 79 | Viral infection | Infectious Diseases | 1.00 | 0.97 | 1.03 | 326439 | 4233 | 322206 |
| 287.31 | Primary thrombocytopenia | Hematopoietic | 0.99 | 0.90 | 1.09 | 325925 | 428 | 325497 |
| 379.3 | Aphakia and other disorders of lens | Sense Organs | 1.00 | 0.99 | 1.02 | 308920 | 20728 | 288192 |
| 622.1 | Polyp of corpus uteri | Genitourinary | 1.00 | 0.98 | 1.03 | 323490 | 8183 | 315307 |
| 614.3 | Pelvic inflammatory disease (PID) | Genitourinary | 1.01 | 0.89 | 1.15 | 319048 | 249 | 318799 |
| 368.4 | Visual field defects | Sense Organs | 0.99 | 0.89 | 1.10 | 325215 | 333 | 324882 |
| 798.1 | Chronic fatigue syndrome | Symptoms | 0.99 | 0.91 | 1.07 | 325335 | 606 | 324729 |
| 705.8 | Hyperhidrosis | Dermatologic | 1.01 | 0.93 | 1.09 | 318896 | 602 | 318294 |
| 165 | Cancer within the respiratory system | Neoplasms | 1.00 | 0.97 | 1.04 | 328223 | 2954 | 325269 |
| 433.31 | Transient cerebral ischemia | Circulatory System | 1.00 | 0.96 | 1.05 | 320281 | 2174 | 318107 |
| 41.4 | E. coli | Infectious Diseases | 1.00 | 0.96 | 1.03 | 315432 | 2800 | 312632 |
| 740.11 | Osteoarthrosis, localized, primary | Musculoskeletal | 1.00 | 0.98 | 1.02 | 315552 | 9227 | 306325 |
| 276.41 | Acidosis | Endocrine/Metabolic | 0.99 | 0.94 | 1.06 | 321700 | 1056 | 320644 |
| 735 | Acquired foot deformities | Musculoskeletal | 0.99 | 0.88 | 1.11 | 316986 | 299 | 316687 |
| 394 | Rheumatic disease of the heart valves | Circulatory System | 1.01 | 0.94 | 1.07 | 322471 | 943 | 321528 |
| 198.6 | Secondary malignancy of bone | Neoplasms | 1.00 | 0.95 | 1.04 | 231505 | 2193 | 229312 |
| 619.2 | Disorders of uterus, NEC | Genitourinary | 1.00 | 0.97 | 1.04 | 281939 | 3391 | 278548 |
| 227.2 | Benign neoplasm of parathyroid gland | Neoplasms | 1.01 | 0.91 | 1.12 | 327032 | 390 | 326642 |
| 275.3 | Disorders of magnesium metabolism | Endocrine/Metabolic | 0.99 | 0.89 | 1.10 | 326410 | 375 | 326035 |
| 350.2 | Abnormality of gait | Neurological | 1.00 | 0.96 | 1.06 | 327077 | 1633 | 325444 |
| 317 | Alcohol-related disorders | Mental Disorders | 1.00 | 0.97 | 1.02 | 303529 | 5860 | 297669 |
| 215 | Other benign neoplasm of connective and other soft tissue | Neoplasms | 0.99 | 0.94 | 1.05 | 321804 | 1128 | 320676 |
| 655 | Known or suspected fetal abnormality affecting management of mother | Pregnancy Complications | 1.00 | 0.97 | 1.04 | 328240 | 4575 | 323665 |
| 366 | Cataract | Sense Organs | 1.00 | 0.99 | 1.02 | 323825 | 16441 | 307384 |
| 345.3 | Convulsions | Neurological | 1.00 | 0.96 | 1.05 | 288463 | 2311 | 286152 |
| 599 | Other symptoms/disorders or the urinary system | Genitourinary | 1.00 | 0.99 | 1.01 | 324256 | 103829 | 220427 |
| 602 | Other disorders of prostate | Genitourinary | 0.99 | 0.94 | 1.05 | 308765 | 1372 | 307393 |
| 694.2 | Other dyschromia | Dermatologic | 1.01 | 0.94 | 1.08 | 322549 | 800 | 321749 |
| 250.22 | Type 2 diabetes with renal manifestations | Endocrine/Metabolic | 1.01 | 0.88 | 1.16 | 307698 | 208 | 307490 |
| 290 | Delirium dementia and amnestic and other cognitive disorders | Mental Disorders | 1.01 | 0.92 | 1.10 | 321950 | 480 | 321470 |
| 736 | Other acquired deformities of limbs | Musculoskeletal | 1.01 | 0.90 | 1.13 | 316998 | 311 | 316687 |
| 568 | Other disorders of peritoneum | Digestive | 1.00 | 0.97 | 1.03 | 250464 | 3804 | 246660 |
| 276.14 | Hypopotassemia | Endocrine/Metabolic | 1.00 | 0.95 | 1.06 | 322115 | 1471 | 320644 |

| | | | | | | | | | |
|---|---|---|---|---|---|---|---|---|---|
| 296.1 | Bipolar | Mental Disorders | 0.99 | 0.94 | 1.06 | 284019 | 1121 | 282898 |
| 610.4 | Benign neoplasm of breast | Genitourinary | 1.00 | 0.95 | 1.06 | 322359 | 1486 | 320873 |
| 724.1 | Disorders of sacrum | Musculoskeletal | 1.01 | 0.90 | 1.14 | 311225 | 281 | 310944 |
| 276.5 | Hypovolemia | Endocrine/Metabolic | 1.00 | 0.97 | 1.04 | 323539 | 2895 | 320644 |
| 720 | Spinal stenosis | Musculoskeletal | 1.01 | 0.93 | 1.08 | 311636 | 692 | 310944 |
| 428.2 | Heart failure NOS | Circulatory System | 1.00 | 0.97 | 1.03 | 326367 | 4334 | 322033 |
| 696.4 | Psoriasis | Dermatologic | 1.00 | 0.95 | 1.05 | 315554 | 1622 | 313932 |
| 610.1 | Cystic mastopathy | Genitourinary | 0.99 | 0.93 | 1.06 | 321832 | 959 | 320873 |
| 289 | Other diseases of blood and blood-forming organs | Hematopoietic | 1.00 | 0.97 | 1.03 | 325300 | 4982 | 320318 |
| 625.1 | Dyspareunia | Genitourinary | 1.00 | 0.94 | 1.06 | 319824 | 1155 | 318669 |
| 339 | Other headache syndromes | Neurological | 1.00 | 0.98 | 1.02 | 325888 | 7938 | 317950 |
| 573.3 | Hepatomegaly | Digestive | 0.99 | 0.89 | 1.11 | 318641 | 310 | 318331 |
| 627 | Menopausal and postmenopausal disorders | Genitourinary | 1.01 | 0.94 | 1.08 | 297078 | 847 | 296231 |
| 278.1 | Obesity | Endocrine/Metabolic | 1.00 | 0.98 | 1.02 | 328110 | 11143 | 316967 |
| 427.42 | Cardiac arrest | Circulatory System | 1.00 | 0.94 | 1.07 | 300470 | 949 | 299521 |
| 709.7 | Unspecified diffuse connective tissue disease | Dermatologic | 1.00 | 0.99 | 1.01 | 324635 | 82669 | 241966 |
| 807 | Fracture of ribs | Injuries & Poisonings | 0.99 | 0.87 | 1.13 | 323780 | 223 | 323557 |
| 571 | Chronic liver disease and cirrhosis | Digestive | 1.01 | 0.92 | 1.10 | 318871 | 540 | 318331 |
| 512.1 | Wheezing | Respiratory | 0.99 | 0.87 | 1.13 | 314499 | 231 | 314268 |
| 386.9 | Dizziness and giddiness (Light-headedness and vertigo) | Sense Organs | 1.00 | 0.97 | 1.03 | 326388 | 4739 | 321649 |
| 727 | Other disorders of synovium, tendon, and bursa | Musculoskeletal | 0.99 | 0.93 | 1.07 | 305708 | 754 | 304954 |
| 619 | Noninflammatory female genital disorders | Genitourinary | 1.00 | 0.99 | 1.01 | 328106 | 49558 | 278548 |
| 509.1 | Respiratory failure | Respiratory | 1.00 | 0.95 | 1.04 | 318564 | 2080 | 316484 |
| 474.1 | Acute tonsillitis | Respiratory | 0.99 | 0.91 | 1.08 | 309515 | 565 | 308950 |
| 458.2 | Iatrogenic hypotension | Circulatory System | 1.01 | 0.89 | 1.15 | 194417 | 238 | 194179 |
| 681 | Superficial cellulitis and abscess | Dermatologic | 1.01 | 0.93 | 1.08 | 317135 | 728 | 316407 |
| 724.9 | Other unspecified back disorders | Musculoskeletal | 1.00 | 0.99 | 1.02 | 328029 | 17085 | 310944 |
| 531.3 | Duodenal ulcer | Digestive | 1.00 | 0.96 | 1.03 | 323644 | 3095 | 320549 |
| 70.4 | Chronic hepatitis | Infectious Diseases | 1.01 | 0.90 | 1.13 | 322496 | 290 | 322206 |
| 528.11 | Stomatitis and mucositis (ulcerative) | Digestive | 0.99 | 0.90 | 1.10 | 322945 | 411 | 322534 |
| 287.3 | Thrombocytopenia | Hematopoietic | 1.00 | 0.95 | 1.06 | 326768 | 1271 | 325497 |
| 859 | Complication due to other implant and internal device | Injuries & Poisonings | 1.00 | 0.96 | 1.03 | 317302 | 3338 | 313964 |
| 474 | Acute and chronic tonsillitis | Respiratory | 1.00 | 0.93 | 1.09 | 309554 | 604 | 308950 |
| 601.12 | Chronic prostatitis | Genitourinary | 1.00 | 0.93 | 1.06 | 308341 | 948 | 307393 |
| 610.3 | Fibrosclerosis of breast | Genitourinary | 0.99 | 0.89 | 1.11 | 321176 | 303 | 320873 |
| 771 | Musculoskeletal symptoms referable to limbs | Symptoms | 1.00 | 0.94 | 1.07 | 327405 | 891 | 326514 |
| 296.22 | Major depressive disorder | Mental Disorders | 1.01 | 0.91 | 1.11 | 283282 | 384 | 282898 |
| 465 | Acute upper respiratory infections of multiple or unspecified sites | Respiratory | 1.00 | 0.96 | 1.03 | 328232 | 3053 | 325179 |

| | | | | | | | | |
|---|---|---|---|---|---|---|---|---|
| 395.2 | Nonrheumatic aortic valve disorders | Circulatory System | 1.01 | 0.89 | 1.14 | 321775 | 247 | 321528 |
| 560.2 | Impaction of intestine | Digestive | 1.01 | 0.89 | 1.13 | 258667 | 271 | 258396 |
| 269 | Proteinuria | Endocrine/Metabolic | 0.99 | 0.90 | 1.10 | 327850 | 355 | 327495 |
| 610 | Benign mammary dysplasias | Genitourinary | 1.01 | 0.88 | 1.16 | 321081 | 208 | 320873 |
| 750.21 | Congenital anomalies of intestine | Congenital Anomalies | 0.99 | 0.87 | 1.13 | 326230 | 227 | 326003 |
| 728.7 | Fasciitis | Musculoskeletal | 1.01 | 0.88 | 1.15 | 305167 | 213 | 304954 |
| 647.1 | Infections of genitourinary tract during pregnancy | Pregnancy Complications | 0.99 | 0.88 | 1.12 | 328128 | 284 | 327844 |
| 958 | Certain early complications of trauma or procedure | Injuries & Poisonings | 1.01 | 0.91 | 1.11 | 328118 | 381 | 327737 |
| 614.54 | Abscess or ulceration of vulva | Genitourinary | 1.01 | 0.89 | 1.13 | 319079 | 280 | 318799 |
| 454.11 | Varicose veins of lower extremity, symptomtic | Circulatory System | 1.00 | 0.93 | 1.08 | 288365 | 652 | 287713 |
| 149.1 | Cancer of oropharynx | Neoplasms | 1.01 | 0.89 | 1.14 | 326297 | 256 | 326041 |
| 575.2 | Obstruction of bile duct | Digestive | 1.00 | 0.94 | 1.08 | 311135 | 812 | 310323 |
| 735.3 | Hallux valgus (Bunion) | Musculoskeletal | 1.00 | 0.98 | 1.03 | 323592 | 6905 | 316687 |
| 572 | Ascites (non malignant) | Digestive | 1.00 | 0.95 | 1.05 | 319925 | 1594 | 318331 |
| 441 | Vascular insufficiency of intestine | Circulatory System | 1.01 | 0.90 | 1.13 | 319657 | 297 | 319360 |
| 441.1 | Acute vascular insufficiency of intestine | Circulatory System | 1.01 | 0.89 | 1.13 | 319631 | 271 | 319360 |
| 78 | Viral warts & HPV | Infectious Diseases | 1.00 | 0.94 | 1.06 | 323218 | 1012 | 322206 |
| 332 | Parkinson's disease | Neurological | 1.00 | 0.95 | 1.06 | 287334 | 1182 | 286152 |
| 473 | Diseases of the larynx and vocal cords | Respiratory | 1.00 | 0.95 | 1.05 | 310564 | 1614 | 308950 |
| 255.21 | Glucocorticoid deficiency | Endocrine/Metabolic | 1.00 | 0.91 | 1.11 | 324917 | 372 | 324545 |
| 315 | Develomental delays and disorders | Mental Disorders | 1.00 | 0.92 | 1.08 | 326992 | 549 | 326443 |
| 594.2 | Calculus of lower urinary tract | Genitourinary | 1.00 | 0.94 | 1.07 | 320761 | 830 | 319931 |
| 331.1 | Hydrocephalus | Neurological | 1.00 | 0.91 | 1.09 | 286652 | 500 | 286152 |
| 961.1 | Poisoning/allergy of sulfonamides | Injuries & Poisonings | 1.00 | 0.93 | 1.07 | 301101 | 890 | 300211 |
| 530 | Diseases of esophagus | Digestive | 1.00 | 0.93 | 1.07 | 288604 | 801 | 287803 |
| 426.91 | Cardiac pacemaker in situ | Circulatory System | 1.00 | 0.96 | 1.04 | 301837 | 2316 | 299521 |
| 433.5 | Cerebral aneurysm | Circulatory System | 1.00 | 0.91 | 1.11 | 318505 | 398 | 318107 |
| 359.2 | Myopathy | Neurological | 1.00 | 0.91 | 1.09 | 326450 | 498 | 325952 |
| 530.9 | Heartburn | Digestive | 1.00 | 0.96 | 1.05 | 289911 | 2108 | 287803 |
| 528.5 | Diseases of lips | Digestive | 1.00 | 0.92 | 1.08 | 323215 | 681 | 322534 |
| 483 | Acute bronchitis and bronchiolitis | Respiratory | 0.99 | 0.87 | 1.14 | 317889 | 200 | 317689 |
| 530.12 | Ulcer of esophagus | Digestive | 1.00 | 0.97 | 1.03 | 293151 | 5348 | 287803 |
| 202.2 | Non-Hodgkins lymphoma | Neoplasms | 1.00 | 0.97 | 1.04 | 327055 | 3532 | 323523 |
| 300.13 | Phobia | Mental Disorders | 1.00 | 0.91 | 1.09 | 283412 | 514 | 282898 |
| 250.24 | Type 2 diabetes with neurological manifestations | Endocrine/Metabolic | 1.00 | 0.93 | 1.09 | 308102 | 612 | 307490 |
| 280.1 | Iron deficiency anemias, unspecified or not due to blood loss | Hematopoietic | 1.00 | 0.98 | 1.02 | 316402 | 7502 | 308900 |
| 569 | Other disorders of intestine | Digestive | 1.00 | 0.99 | 1.01 | 326148 | 79488 | 246660 |
| 240 | Simple and unspecified goiter | Endocrine/Metabolic | 1.00 | 0.93 | 1.08 | 310951 | 622 | 310329 |

| | | | | | | | | |
|---|---|---|---|---|---|---|---|---|
| 938.2 | Chronic dermatitis due to solar radiation | Injuries & Poisonings | 1.00 | 0.89 | 1.13 | 322170 | 264 | 321906 |
| 965.3 | Salicylates causing adverse effects in therapeutic use | Injuries & Poisonings | 1.00 | 0.91 | 1.11 | 300612 | 401 | 300211 |
| 496.3 | Bronchiectasis | Respiratory | 1.00 | 0.95 | 1.05 | 296077 | 1885 | 294192 |
| 274.21 | Chondrocalcinosis | Endocrine/Metabolic | 1.00 | 0.89 | 1.13 | 326552 | 290 | 326262 |
| 389.2 | Conductive hearing loss | Sense Organs | 1.00 | 0.91 | 1.10 | 318629 | 435 | 318194 |
| 613.7 | Other signs and symptoms in breast | Genitourinary | 1.00 | 0.93 | 1.08 | 323169 | 751 | 322418 |
| 809 | Fracture of unspecified bones | Injuries & Poisonings | 1.00 | 0.91 | 1.11 | 323967 | 410 | 323557 |
| 286.7 | Other and unspecified coagulation defects | Hematopoietic | 1.00 | 0.91 | 1.11 | 325902 | 405 | 325497 |
| 280.2 | Iron deficiency anemia secondary to blood loss (chronic) | Hematopoietic | 1.00 | 0.92 | 1.10 | 309376 | 476 | 308900 |
| 695.3 | Rosacea | Dermatologic | 1.00 | 0.89 | 1.11 | 322068 | 319 | 321749 |
| 204.4 | Multiple myeloma | Neoplasms | 1.00 | 0.92 | 1.08 | 324097 | 574 | 323523 |
| 561 | Symptoms involving digestive system | Digestive | 1.00 | 0.98 | 1.02 | 272941 | 14545 | 258396 |
| 512.7 | Shortness of breath | Respiratory | 1.00 | 0.97 | 1.03 | 320384 | 6116 | 314268 |
| 574.12 | Cholelithiasis with other cholecystitis | Digestive | 1.00 | 0.97 | 1.03 | 315816 | 5493 | 310323 |
| 189.11 | Malignant neoplasm of kidney, except pelvis | Neoplasms | 1.00 | 0.94 | 1.06 | 324997 | 1035 | 323962 |
| 965 | Poisoning by analgesics, antipyretics, and antirheumatics | Injuries & Poisonings | 1.00 | 0.98 | 1.03 | 306293 | 6082 | 300211 |
| 288.11 | Neutropenia | Hematopoietic | 1.00 | 0.97 | 1.04 | 323644 | 3326 | 320318 |
| 716.9 | Arthropathy NOS | Musculoskeletal | 1.00 | 0.99 | 1.01 | 327719 | 53823 | 273896 |
| 627.1 | Postmenopausal bleeding | Genitourinary | 1.00 | 0.98 | 1.02 | 305697 | 9466 | 296231 |
| 349 | Other and unspecified disorders of the nervous system | Neurological | 1.00 | 0.99 | 1.01 | 326855 | 40703 | 286152 |
| 761 | Cervicalgia | Symptoms | 1.00 | 0.94 | 1.07 | 328240 | 1005 | 327235 |
| 627.3 | Postmenopausal atrophic vaginitis | Genitourinary | 1.00 | 0.94 | 1.06 | 297359 | 1128 | 296231 |
| 323.8 | Encephalitis, non-infectious | Neurological | 1.00 | 0.93 | 1.08 | 327771 | 633 | 327138 |
| 836 | Traumatic arthropathy | Injuries & Poisonings | 1.00 | 0.88 | 1.14 | 322016 | 243 | 321773 |
| 418 | Nonspecific chest pain | Circulatory System | 1.00 | 0.99 | 1.01 | 326032 | 30212 | 295820 |
| 854 | Complications of cardiac/vascular device, implant, and graft | Injuries & Poisonings | 1.00 | 0.96 | 1.05 | 315807 | 1843 | 313964 |
| 695.9 | Unspecified erythematous condition | Dermatologic | 1.00 | 0.91 | 1.09 | 322226 | 477 | 321749 |
| 8.52 | Intestinal infection due to C. difficile | Infectious Diseases | 1.00 | 0.93 | 1.08 | 319727 | 648 | 319079 |
| 622.2 | Mucous polyp of cervix | Genitourinary | 1.00 | 0.97 | 1.03 | 318829 | 3522 | 315307 |
| 681.2 | Cellulitis and abscess of face/neck | Dermatologic | 1.00 | 0.92 | 1.09 | 316936 | 529 | 316407 |
| 433.21 | Cerebral artery occlusion, with cerebral infarction | Circulatory System | 1.00 | 0.95 | 1.05 | 319641 | 1534 | 318107 |
| 626.13 | Irregular menstrual cycle | Genitourinary | 1.00 | 0.96 | 1.05 | 298189 | 1958 | 296231 |
| 81 | Infection/inflammation of internal prosthetic device; implant; and graft | Infectious Diseases | 1.00 | 0.96 | 1.04 | 323993 | 2514 | 321479 |
| 577.2 | Chronic pancreatitis | Digestive | 1.00 | 0.92 | 1.09 | 326456 | 536 | 325920 |
| 594.8 | Renal colic | Genitourinary | 1.00 | 0.96 | 1.04 | 322062 | 2131 | 319931 |
| 478 | Throat pain | Respiratory | 1.00 | 0.90 | 1.10 | 309337 | 387 | 308950 |
| 574.11 | Cholelithiasis with acute cholecystitis | Digestive | 1.00 | 0.95 | 1.05 | 311836 | 1513 | 310323 |
| 289.4 | Lymphadenitis | Hematopoietic | 1.00 | 0.96 | 1.04 | 323033 | 2715 | 320318 |

| Supplementary Table 14. Phenome-wide association study (PheWAS) results for the calcium channel blocker (CCB) genetic risk score when using single-nucleotide polymorphisms from the systolic blood pressure genome-wide association study that did not correct for antihypertensive medication use or adjust for body mass index. | | | | | | | | |
|---|---|---|---|---|---|---|---|---|
| Phecode | Trait | Category | OR | Low 95% CI | Upp 95% CI | Total sample size | Cases | Controls |
| 401.1 | Essential hypertension | Circulatory System | 0.96 | 0.95 | 0.97 | 327983 | 79235 | 248748 |
| 401 | Hypertension | Circulatory System | 0.96 | 0.95 | 0.97 | 328239 | 79491 | 248748 |
| 459.9 | Circulatory disease NEC | Circulatory System | 0.98 | 0.97 | 0.98 | 327928 | 133749 | 194179 |
| 411.2 | Myocardial infarction | Circulatory System | 0.96 | 0.95 | 0.98 | 307658 | 11969 | 295689 |
| 411.41 | Aneurysm and dissection of heart | Circulatory System | 0.87 | 0.80 | 0.93 | 296410 | 721 | 295689 |
| 411.8 | Other chronic ischemic heart disease, unspecified | Circulatory System | 0.98 | 0.97 | 0.99 | 326969 | 31280 | 295689 |
| 411 | Ischemic Heart Disease | Circulatory System | 0.98 | 0.97 | 0.99 | 327032 | 31343 | 295689 |
| 411.4 | Coronary atherosclerosis | Circulatory System | 0.98 | 0.97 | 0.99 | 327242 | 31553 | 295689 |
| 960 | Poisoning by antibiotics | Injuries & Poisonings | 0.95 | 0.92 | 0.98 | 303739 | 3528 | 300211 |
| 427.2 | Atrial fibrillation and flutter | Circulatory System | 0.97 | 0.96 | 0.99 | 314573 | 15052 | 299521 |
| 473 | Diseases of the larynx and vocal cords | Respiratory | 0.93 | 0.88 | 0.97 | 310564 | 1614 | 308950 |
| 411.3 | Angina pectoris | Circulatory System | 0.98 | 0.96 | 0.99 | 312048 | 16359 | 295689 |
| 592.13 | Chronic interstitial cystitis | Genitourinary | 0.84 | 0.74 | 0.95 | 297289 | 254 | 297035 |
| 562.1 | Diverticulosis | Digestive | 1.02 | 1.01 | 1.03 | 286281 | 27885 | 258396 |
| 555 | Inflammatory bowel disease and other gastroenteritis and colitis | Digestive | 1.02 | 1.01 | 1.03 | 278195 | 19799 | 258396 |
| 428.1 | Congestive heart failure (CHF) NOS | Circulatory System | 0.94 | 0.90 | 0.99 | 324086 | 2053 | 322033 |
| 180.3 | Cervical intraepithelial neoplasia [CIN] [Cervical dysplasia] | Neoplasms | 1.06 | 1.01 | 1.10 | 299736 | 2153 | 297583 |
| 558 | Noninfectious gastroenteritis | Digestive | 1.02 | 1.00 | 1.03 | 278260 | 19864 | 258396 |
| 8.5 | Bacterial enteritis | Infectious Diseases | 1.06 | 1.01 | 1.10 | 321188 | 2109 | 319079 |
| 573 | Other disorders of liver | Digestive | 1.04 | 1.01 | 1.07 | 323533 | 5202 | 318331 |
| 240 | Simple and unspecified goiter | Endocrine/Metabolic | 0.90 | 0.83 | 0.97 | 310951 | 622 | 310329 |
| 703 | Diseases of nail, NOS | Dermatologic | 0.87 | 0.79 | 0.97 | 321795 | 366 | 321429 |
| 428.2 | Heart failure NOS | Circulatory System | 0.96 | 0.93 | 0.99 | 326367 | 4334 | 322033 |
| 375 | Disorders of lacrimal system | Sense Organs | 1.07 | 1.02 | 1.12 | 289852 | 1660 | 288192 |
| 433.12 | Cerebral atherosclerosis | Circulatory System | 1.18 | 1.04 | 1.34 | 318345 | 238 | 318107 |
| 574 | Cholelithiasis and cholecystitis | Digestive | 0.88 | 0.80 | 0.97 | 310726 | 403 | 310323 |
| 601.11 | Acute prostatitis | Genitourinary | 1.17 | 1.03 | 1.32 | 307653 | 260 | 307393 |
| 250.41 | Impaired fasting glucose | Endocrine/Metabolic | 1.16 | 1.03 | 1.31 | 307758 | 268 | 307490 |
| 751.11 | Congenital anomalies of female genital organs | Congenital Anomalies | 0.89 | 0.81 | 0.98 | 326445 | 442 | 326003 |
| 642.1 | Preeclampsia and eclampsia | Pregnancy Complications | 0.88 | 0.79 | 0.98 | 327385 | 335 | 327050 |
| 426.31 | Right bundle branch block | Circulatory System | 0.94 | 0.90 | 0.99 | 301094 | 1573 | 299521 |
| 426.3 | Bundle branch block | Circulatory System | 1.15 | 1.02 | 1.29 | 299812 | 291 | 299521 |
| 304 | Adjustment reaction | Mental Disorders | 1.14 | 1.02 | 1.27 | 283221 | 323 | 282898 |
| 601.1 | Prostatitis | Genitourinary | 1.10 | 1.01 | 1.18 | 308058 | 665 | 307393 |
| 361.1 | Retinal detachment with retinal defect | Sense Organs | 0.94 | 0.89 | 0.99 | 318238 | 1456 | 316782 |

| | | | | | | | | | |
|---|---|---|---|---|---|---|---|---|---|
| 348.7 | Coma | Neurological | 1.12 | 1.02 | 1.24 | 286531 | 379 | 286152 |
| 636 | Early or threatened labor; hemorrhage in early pregnancy | Pregnancy Complications | 1.07 | 1.01 | 1.13 | 321227 | 1262 | 319965 |
| 751.1 | Congenital anomalies of genital organs | Congenital Anomalies | 0.92 | 0.85 | 0.99 | 326709 | 706 | 326003 |
| 525 | Other diseases of the teeth and supporting structures | Digestive | 1.04 | 1.01 | 1.08 | 314357 | 2699 | 311658 |
| 634.1 | Missed abortion/Hydatidiform mole | Pregnancy Complications | 1.07 | 1.01 | 1.13 | 321168 | 1203 | 319965 |
| 722.1 | Displacement of intervertebral disc | Musculoskeletal | 1.10 | 1.01 | 1.20 | 311460 | 516 | 310944 |
| 608 | Other disorders of male genital organs | Genitourinary | 1.02 | 1.00 | 1.03 | 327998 | 20092 | 307906 |
| 686.3 | Pilonidal cyst | Dermatologic | 1.09 | 1.01 | 1.18 | 317024 | 617 | 316407 |
| 627.4 | Premenopausal menorrhagia | Genitourinary | 0.89 | 0.80 | 0.99 | 296563 | 332 | 296231 |
| 614.32 | Chronic inflammatory pelvic disease | Genitourinary | 0.90 | 0.82 | 0.99 | 319224 | 425 | 318799 |
| 416 | Cardiomegaly | Circulatory System | 0.96 | 0.92 | 1.00 | 324157 | 2610 | 321547 |
| 696.42 | Psoriatic arthropathy | Dermatologic | 1.08 | 1.01 | 1.16 | 314672 | 740 | 313932 |
| 371.1 | Uveitis, noninfectious or NOS | Sense Organs | 1.10 | 1.01 | 1.20 | 318886 | 498 | 318388 |
| 480.11 | Pneumococcal pneumonia | Respiratory | 1.03 | 1.00 | 1.05 | 323680 | 5991 | 317689 |
| 395.1 | Nonrheumatic mitral valve disorders | Circulatory System | 0.96 | 0.93 | 1.00 | 324497 | 2969 | 321528 |
| 220 | Benign neoplasm of ovary | Neoplasms | 1.06 | 1.00 | 1.11 | 297546 | 1490 | 296056 |
| 703.1 | Ingrowing nail | Dermatologic | 0.94 | 0.88 | 1.00 | 322439 | 1010 | 321429 |
| 747.11 | Cardiac shunt/ heart septal defect | Congenital Anomalies | 0.92 | 0.85 | 0.99 | 325935 | 581 | 325354 |
| 571.5 | Other chronic nonalcoholic liver disease | Digestive | 1.05 | 1.00 | 1.10 | 320004 | 1673 | 318331 |
| 199 | Neoplasm of uncertain behavior | Neoplasms | 0.94 | 0.89 | 1.00 | 230468 | 1156 | 229312 |
| 316 | Substance addiction and disorders | Mental Disorders | 1.11 | 1.00 | 1.23 | 298042 | 373 | 297669 |
| 513.4 | Hyperventilation | Respiratory | 1.13 | 1.00 | 1.27 | 327904 | 280 | 327624 |
| 292.1 | Aphasia/speech disturbance | Mental Disorders | 0.95 | 0.90 | 1.00 | 323019 | 1549 | 321470 |
| 646 | Other complications of pregnancy NEC | Pregnancy Complications | 1.04 | 1.00 | 1.09 | 328240 | 2396 | 325844 |
| 977 | Personal history of allergy to medicinal agents | Injuries & Poisonings | 0.88 | 0.78 | 1.00 | 300449 | 238 | 300211 |
| 394.2 | Mitral valve disease | Circulatory System | 0.97 | 0.93 | 1.00 | 324603 | 3075 | 321528 |
| 474 | Acute and chronic tonsillitis | Respiratory | 1.08 | 1.00 | 1.17 | 309554 | 604 | 308950 |
| 371 | Inflammation of the eye | Sense Organs | 0.91 | 0.83 | 1.00 | 318796 | 408 | 318388 |
| 604 | Disorders of penis | Genitourinary | 1.07 | 1.00 | 1.15 | 308641 | 735 | 307906 |
| 426.32 | Left bundle branch block | Circulatory System | 0.96 | 0.91 | 1.00 | 301302 | 1781 | 299521 |
| 601.12 | Chronic prostatitis | Genitourinary | 1.06 | 1.00 | 1.13 | 308341 | 948 | 307393 |
| 70.4 | Chronic hepatitis | Infectious Diseases | 1.12 | 0.99 | 1.25 | 322496 | 290 | 322206 |
| 372 | Disorders of conjunctiva | Sense Organs | 0.94 | 0.89 | 1.00 | 319431 | 1043 | 318388 |
| 250.24 | Type 2 diabetes with neurological manifestations | Endocrine/Metabolic | 0.93 | 0.86 | 1.00 | 308102 | 612 | 307490 |
| 727.5 | Rupture of synovium | Musculoskeletal | 1.10 | 0.99 | 1.23 | 305298 | 344 | 304954 |
| 90 | Sexually transmitted infections (not HIV or hepatitis) | Infectious Diseases | 1.11 | 0.99 | 1.24 | 328239 | 298 | 327941 |
| 478 | Throat pain | Respiratory | 0.91 | 0.83 | 1.01 | 309337 | 387 | 308950 |
| 473.3 | Paralysis/spasm of vocal cords or larynx | Respiratory | 0.90 | 0.79 | 1.01 | 309217 | 267 | 308950 |

| | | | | | | | | | |
|---|---|---|---|---|---|---|---|---|---|
| 272.1 | Hyperlipidemia | Endocrine/Metabolic | 0.98 | 0.95 | 1.00 | 296728 | 5231 | 291497 |
| 345 | Epilepsy, recurrent seizures, convulsions | Neurological | 1.03 | 1.00 | 1.07 | 289613 | 3461 | 286152 |
| 276.5 | Hypovolemia | Endocrine/Metabolic | 1.03 | 1.00 | 1.07 | 323539 | 2895 | 320644 |
| 635.3 | Placenta previa and abruptio placenta | Pregnancy Complications | 1.05 | 1.00 | 1.11 | 321301 | 1336 | 319965 |
| 429.3 | Symptoms involving cardiovascular system | Circulatory System | 0.88 | 0.77 | 1.01 | 322241 | 208 | 322033 |
| 850 | Hemorrhage or hematoma complicating a procedure | Injuries & Poisonings | 0.98 | 0.95 | 1.00 | 319328 | 5364 | 313964 |
| 38.2 | Gram positive septicemia | Infectious Diseases | 0.92 | 0.85 | 1.01 | 313123 | 491 | 312632 |
| 475 | Chronic sinusitis | Respiratory | 1.03 | 1.00 | 1.08 | 311580 | 2630 | 308950 |
| 750 | Digestive congenital anomalies | Congenital Anomalies | 0.94 | 0.87 | 1.01 | 326705 | 702 | 326003 |
| 756 | Other congenital musculoskeletal anomalies | Congenital Anomalies | 0.94 | 0.88 | 1.01 | 328123 | 812 | 327311 |
| 598 | Abnormal findings on examination of urine | Genitourinary | 1.03 | 1.00 | 1.07 | 328240 | 3386 | 324854 |
| 427.9 | Palpitations | Circulatory System | 0.97 | 0.94 | 1.00 | 303480 | 3959 | 299521 |
| 331 | Other cerebral degenerations | Neurological | 1.12 | 0.99 | 1.27 | 286392 | 240 | 286152 |
| 726.3 | Bursitis | Musculoskeletal | 1.08 | 0.99 | 1.17 | 305497 | 543 | 304954 |
| 854 | Complications of cardiac/vascular device, implant, and graft | Injuries & Poisonings | 0.96 | 0.92 | 1.01 | 315807 | 1843 | 313964 |
| 333.1 | Essential tremor | Neurological | 0.89 | 0.78 | 1.02 | 286370 | 218 | 286152 |
| 578 | Gastrointestinal hemorrhage | Digestive | 1.11 | 0.98 | 1.24 | 303679 | 285 | 303394 |
| 574.1 | Cholelithiasis | Digestive | 1.02 | 1.00 | 1.04 | 319629 | 9306 | 310323 |
| 870.4 | Open wound of nose and sinus | Injuries & Poisonings | 1.11 | 0.98 | 1.27 | 319969 | 238 | 319731 |
| 555.2 | Ulcerative colitis | Digestive | 1.03 | 0.99 | 1.07 | 261550 | 3154 | 258396 |
| 530.2 | Esophageal bleeding (varices/hemorrhage) | Digestive | 1.04 | 0.99 | 1.09 | 289525 | 1722 | 287803 |
| 741.4 | Joint effusions | Musculoskeletal | 0.91 | 0.81 | 1.02 | 310925 | 283 | 310642 |
| 458.9 | Hypotension NOS | Circulatory System | 0.97 | 0.94 | 1.00 | 197817 | 3638 | 194179 |
| 477 | Epistaxis or throat hemorrhage | Respiratory | 0.97 | 0.93 | 1.01 | 311469 | 2519 | 308950 |
| 250 | Diabetes mellitus | Endocrine/Metabolic | 1.01 | 1.00 | 1.03 | 327730 | 20240 | 307490 |
| 727 | Other disorders of synovium, tendon, and bursa | Musculoskeletal | 1.06 | 0.99 | 1.14 | 305708 | 754 | 304954 |
| 440 | Atherosclerosis | Circulatory System | 0.89 | 0.78 | 1.02 | 319564 | 204 | 319360 |
| 571 | Chronic liver disease and cirrhosis | Digestive | 1.07 | 0.99 | 1.17 | 318871 | 540 | 318331 |
| 414 | Other forms of chronic heart disease | Circulatory System | 0.96 | 0.92 | 1.01 | 297457 | 1768 | 295689 |
| 740.12 | Osteoarthrosis, localized, secondary | Musculoskeletal | 0.90 | 0.80 | 1.02 | 306589 | 264 | 306325 |
| 442.8 | Aneurysm of other specified artery | Circulatory System | 0.89 | 0.78 | 1.02 | 319570 | 210 | 319360 |
| 496.21 | Obstructive chronic bronchitis | Respiratory | 0.97 | 0.93 | 1.01 | 296921 | 2729 | 294192 |
| 189.21 | Malignant neoplasm of bladder | Neoplasms | 1.04 | 0.99 | 1.08 | 326150 | 2188 | 323962 |
| 274.21 | Chondrocalcinosis | Endocrine/Metabolic | 1.10 | 0.98 | 1.23 | 326552 | 290 | 326262 |
| 382 | Otalgia | Sense Organs | 1.10 | 0.98 | 1.22 | 324458 | 315 | 324143 |
| 202 | Cancer of other lymphoid, histiocytic tissue | Neoplasms | 0.95 | 0.90 | 1.01 | 324661 | 1138 | 323523 |
| 728.71 | Contracture of palmar fascia [Dupuytren's disease] | Musculoskeletal | 0.97 | 0.93 | 1.01 | 307403 | 2449 | 304954 |
| 250.2 | Type 2 diabetes | Endocrine/Metabolic | 1.01 | 1.00 | 1.03 | 326603 | 19113 | 307490 |

| | | | | | | | | |
|---|---|---|---|---|---|---|---|---|
| 378.1 | Strabismus (not specified as paralytic) | Sense Organs | 1.05 | 0.99 | 1.12 | 289139 | 947 | 288192 |
| 41 | Bacterial infection NOS | Infectious Diseases | 0.98 | 0.97 | 1.00 | 323384 | 10752 | 312632 |
| 724.2 | Disorders of coccyx | Musculoskeletal | 0.91 | 0.81 | 1.02 | 311226 | 282 | 310944 |
| 613 | Other nonmalignant breast conditions | Genitourinary | 0.92 | 0.83 | 1.02 | 322786 | 368 | 322418 |
| 800.1 | Fracture of neck of femur | Injuries & Poisonings | 0.90 | 0.80 | 1.03 | 323796 | 239 | 323557 |
| 218.2 | Other benign neoplasm of uterus | Neoplasms | 1.09 | 0.98 | 1.20 | 308143 | 363 | 307780 |
| 669 | Complications of labor and delivery NEC | Pregnancy Complications | 1.02 | 1.00 | 1.04 | 328240 | 9534 | 318706 |
| 729 | Other disorders of soft tissues | Musculoskeletal | 1.01 | 1.00 | 1.02 | 327478 | 22524 | 304954 |
| 442.1 | Aortic aneurysm | Circulatory System | 0.94 | 0.87 | 1.02 | 319946 | 586 | 319360 |
| 426.24 | Atrioventricular block, complete | Circulatory System | 0.94 | 0.87 | 1.02 | 300112 | 591 | 299521 |
| 681 | Superficial cellulitis and abscess | Dermatologic | 0.95 | 0.88 | 1.02 | 317135 | 728 | 316407 |
| 452 | Other venous embolism and thrombosis | Circulatory System | 0.94 | 0.86 | 1.02 | 288267 | 554 | 287713 |
| 724.1 | Disorders of sacrum | Musculoskeletal | 0.91 | 0.81 | 1.03 | 311225 | 281 | 310944 |
| 686.1 | Carbuncle and furuncle | Dermatologic | 1.03 | 0.99 | 1.07 | 318829 | 2422 | 316407 |
| 378.5 | Paralytic strabismus | Sense Organs | 0.92 | 0.82 | 1.03 | 288479 | 287 | 288192 |
| 374 | Other disorders of eyelids | Sense Organs | 0.97 | 0.94 | 1.01 | 321593 | 3205 | 318388 |
| 508 | Pulmonary collapse; interstitial and compensatory emphysema | Respiratory | 1.03 | 0.99 | 1.08 | 318570 | 2086 | 316484 |
| 681.7 | Cellulitis and abscess of trunk | Dermatologic | 1.06 | 0.98 | 1.15 | 317030 | 623 | 316407 |
| 389.4 | Tinnitus | Sense Organs | 1.07 | 0.98 | 1.16 | 318735 | 541 | 318194 |
| 290.1 | Dementias | Mental Disorders | 1.06 | 0.98 | 1.14 | 322121 | 651 | 321470 |
| 381.11 | Suppurative and unspecified otitis media | Sense Organs | 0.95 | 0.89 | 1.02 | 324993 | 850 | 324143 |
| 78 | Viral warts & HPV | Infectious Diseases | 1.05 | 0.98 | 1.11 | 323218 | 1012 | 322206 |
| 626.8 | Infertility, female | Genitourinary | 0.96 | 0.91 | 1.01 | 297638 | 1407 | 296231 |
| 375.2 | Epiphora | Sense Organs | 1.05 | 0.98 | 1.12 | 289094 | 902 | 288192 |
| 747 | Cardiac and circulatory congenital anomalies | Congenital Anomalies | 0.96 | 0.91 | 1.01 | 326576 | 1222 | 325354 |
| 300.1 | Anxiety disorder | Mental Disorders | 0.98 | 0.95 | 1.01 | 288368 | 5470 | 282898 |
| 870.3 | Other open wound of head and face | Injuries & Poisonings | 1.03 | 0.99 | 1.06 | 322782 | 3051 | 319731 |
| 450 | Noninfectious disorders of lymphatic channels | Circulatory System | 0.95 | 0.88 | 1.02 | 328240 | 717 | 327523 |
| 172.2 | Other non-epithelial cancer of skin | Neoplasms | 1.01 | 1.00 | 1.03 | 325462 | 11242 | 314220 |
| 577 | Diseases of pancreas | Digestive | 1.06 | 0.98 | 1.15 | 326478 | 558 | 325920 |
| 800.3 | Fracture of tibia and fibula | Injuries & Poisonings | 0.94 | 0.86 | 1.02 | 324092 | 535 | 323557 |
| 317 | Alcohol-related disorders | Mental Disorders | 1.02 | 0.99 | 1.05 | 303529 | 5860 | 297669 |
| 385.3 | Cholesteatoma | Sense Organs | 1.06 | 0.98 | 1.15 | 324732 | 589 | 324143 |
| 962.3 | Hormones and synthetic substitutes causing adverse effects in therapeutic use | Injuries & Poisonings | 1.08 | 0.97 | 1.19 | 300575 | 364 | 300211 |
| 519.8 | Other diseases of respiratory system, NEC | Respiratory | 1.02 | 0.99 | 1.04 | 274529 | 9082 | 265447 |
| 324 | Other CNS infection and poliomyelitis | Neurological | 1.09 | 0.97 | 1.22 | 327426 | 288 | 327138 |
| 215 | Other benign neoplasm of connective and other soft tissue | Neoplasms | 1.04 | 0.98 | 1.11 | 321804 | 1128 | 320676 |
| 198.6 | Secondary malignancy of bone | Neoplasms | 1.03 | 0.99 | 1.08 | 231505 | 2193 | 229312 |

| | | | | | | | | | |
|---|---|---|---|---|---|---|---|---|---|
| 474.1 | Acute tonsillitis | Respiratory | 0.94 | 0.87 | 1.02 | 309515 | 565 | 308950 |
| 425.1 | Primary/intrinsic cardiomyopathies | Circulatory System | 0.96 | 0.90 | 1.02 | 326036 | 1053 | 324983 |
| 149.1 | Cancer of oropharynx | Neoplasms | 1.09 | 0.97 | 1.23 | 326297 | 256 | 326041 |
| 345.11 | Generalized convulsive epilepsy | Neurological | 1.07 | 0.98 | 1.17 | 286617 | 465 | 286152 |
| 80 | Postoperative infection | Infectious Diseases | 1.02 | 0.99 | 1.05 | 326101 | 4622 | 321479 |
| 149.4 | Cancer of larynx | Neoplasms | 0.92 | 0.81 | 1.03 | 326307 | 266 | 326041 |
| 241.1 | Nontoxic uninodular goiter | Endocrine/Metabolic | 1.06 | 0.98 | 1.16 | 310840 | 511 | 310329 |
| 446.5 | Giant cell arteritis | Circulatory System | 0.93 | 0.84 | 1.03 | 319750 | 390 | 319360 |
| 394.7 | Disease of tricuspid valve | Circulatory System | 0.96 | 0.90 | 1.02 | 322614 | 1086 | 321528 |
| 600 | Hyperplasia of prostate | Genitourinary | 1.01 | 0.99 | 1.03 | 318965 | 11572 | 307393 |
| 496.2 | Chronic bronchitis | Respiratory | 1.08 | 0.97 | 1.22 | 294485 | 293 | 294192 |
| 250.1 | Type 1 diabetes | Endocrine/Metabolic | 1.03 | 0.99 | 1.07 | 310189 | 2699 | 307490 |
| 348.2 | Cerebral edema and compression of brain | Neurological | 1.09 | 0.96 | 1.23 | 286412 | 260 | 286152 |
| 507 | Pleurisy; pleural effusion | Respiratory | 0.98 | 0.96 | 1.01 | 323091 | 6607 | 316484 |
| 938.2 | Chronic dermatitis due to solar radiation | Injuries & Poisonings | 0.92 | 0.81 | 1.04 | 322170 | 264 | 321906 |
| 700 | Corns and callosities | Dermatologic | 0.92 | 0.82 | 1.04 | 323352 | 288 | 323064 |
| 480.5 | Bronchopneumonia and lung abscess | Respiratory | 0.93 | 0.85 | 1.03 | 318077 | 388 | 317689 |
| 208 | Benign neoplasm of colon | Neoplasms | 0.99 | 0.98 | 1.00 | 325437 | 20827 | 304610 |
| 189.11 | Malignant neoplasm of kidney, except pelvis | Neoplasms | 0.96 | 0.90 | 1.02 | 324997 | 1035 | 323962 |
| 526.1 | Cysts of the jaws | Digestive | 1.08 | 0.97 | 1.20 | 311993 | 335 | 311658 |
| 750.21 | Congenital anomalies of intestine | Congenital Anomalies | 0.91 | 0.80 | 1.04 | 326230 | 227 | 326003 |
| 594.3 | Calculus of ureter | Genitourinary | 0.97 | 0.94 | 1.01 | 322471 | 2540 | 319931 |
| 798 | Malaise and fatigue | Symptoms | 1.02 | 0.99 | 1.06 | 327695 | 2966 | 324729 |
| 593 | Hematuria | Genitourinary | 1.01 | 1.00 | 1.03 | 313795 | 16760 | 297035 |
| 755.1 | Congenital deformities of feet | Congenital Anomalies | 0.91 | 0.80 | 1.04 | 327531 | 220 | 327311 |
| 394 | Rheumatic disease of the heart valves | Circulatory System | 0.96 | 0.90 | 1.02 | 322471 | 943 | 321528 |
| 614.51 | Cervicitis and endocervicitis | Genitourinary | 0.96 | 0.91 | 1.02 | 320048 | 1249 | 318799 |
| 577.1 | Acute pancreatitis | Digestive | 1.03 | 0.98 | 1.09 | 327437 | 1517 | 325920 |
| 333 | Extrapyramidal disease and abnormal movement disorders | Neurological | 0.97 | 0.93 | 1.01 | 288187 | 2035 | 286152 |
| 624.1 | Dystrophy of female genital tract | Genitourinary | 0.92 | 0.81 | 1.04 | 318909 | 240 | 318669 |
| 735 | Acquired foot deformities | Musculoskeletal | 1.08 | 0.96 | 1.21 | 316986 | 299 | 316687 |
| 801.1 | Fracture of foot | Injuries & Poisonings | 0.94 | 0.85 | 1.03 | 323975 | 418 | 323557 |
| 528 | Diseases of the oral soft tissues, excluding lesions specific for gingiva and tongue | Digestive | 0.97 | 0.93 | 1.01 | 324593 | 2059 | 322534 |
| 571.8 | Liver abscess and sequelae of chronic liver disease | Digestive | 1.06 | 0.97 | 1.15 | 318904 | 573 | 318331 |
| 946 | Anaphylactic shock NOS | Injuries & Poisonings | 0.95 | 0.87 | 1.03 | 322450 | 544 | 321906 |
| 350.2 | Abnormality of gait | Neurological | 0.97 | 0.92 | 1.02 | 327077 | 1633 | 325444 |
| 859 | Complication due to other implant and internal device | Injuries & Poisonings | 0.98 | 0.94 | 1.01 | 317302 | 3338 | 313964 |
| 379.5 | Disorders of iris and ciliary body | Sense Organs | 1.07 | 0.97 | 1.17 | 288604 | 412 | 288192 |

| | | | | | | | | |
|---|---|---|---|---|---|---|---|---|
| 738.4 | Acquired spondylolisthesis | Musculoskeletal | 1.09 | 0.96 | 1.23 | 316942 | 255 | 316687 |
| 858 | Complication of internal orthopedic device | Injuries & Poisonings | 0.98 | 0.94 | 1.01 | 317147 | 3183 | 313964 |
| 702.2 | Seborrheic keratosis | Dermatologic | 1.02 | 0.99 | 1.06 | 325769 | 3169 | 322600 |
| 386.3 | Labyrinthitis | Sense Organs | 0.95 | 0.89 | 1.02 | 322440 | 791 | 321649 |
| 573.5 | Jaundice (not of newborn) | Digestive | 1.04 | 0.98 | 1.11 | 319269 | 938 | 318331 |
| 958 | Certain early complications of trauma or procedure | Injuries & Poisonings | 1.07 | 0.97 | 1.18 | 328118 | 381 | 327737 |
| 590 | Pyelonephritis | Genitourinary | 1.02 | 0.99 | 1.05 | 300960 | 3925 | 297035 |
| 642 | Hypertension complicating pregnancy, childbirth, and the puerperium | Pregnancy Complications | 0.96 | 0.90 | 1.02 | 328039 | 989 | 327050 |
| 969 | Poisoning by psychotropic agents | Injuries & Poisonings | 1.03 | 0.99 | 1.08 | 302207 | 1996 | 300211 |
| 70 | Viral hepatitis | Infectious Diseases | 1.05 | 0.98 | 1.13 | 322928 | 722 | 322206 |
| 613.1 | Inflammatory disease of breast | Genitourinary | 0.95 | 0.89 | 1.03 | 323156 | 738 | 322418 |
| 530.9 | Heartburn | Digestive | 0.97 | 0.93 | 1.01 | 289911 | 2108 | 287803 |
| 801 | Fracture of ankle and foot | Injuries & Poisonings | 0.92 | 0.82 | 1.04 | 323808 | 251 | 323557 |
| 287.3 | Thrombocytopenia | Hematopoietic | 0.96 | 0.91 | 1.02 | 326768 | 1271 | 325497 |
| 575.8 | Other disorders of biliary tract | Digestive | 0.96 | 0.91 | 1.02 | 311372 | 1049 | 310323 |
| 528.11 | Stomatitis and mucositis (ulcerative) | Digestive | 1.06 | 0.97 | 1.17 | 322945 | 411 | 322534 |
| 574.3 | Cholecystitis without cholelithiasis | Digestive | 1.02 | 0.99 | 1.06 | 313141 | 2818 | 310323 |
| 695.3 | Rosacea | Dermatologic | 0.93 | 0.84 | 1.04 | 322068 | 319 | 321749 |
| 359.2 | Myopathy | Neurological | 0.95 | 0.87 | 1.03 | 326450 | 498 | 325952 |
| 352.2 | Facial nerve disorders [CN7] | Neurological | 0.96 | 0.90 | 1.02 | 313911 | 900 | 313011 |
| 636.2 | Early onset of delivery | Pregnancy Complications | 0.95 | 0.88 | 1.03 | 320594 | 629 | 319965 |
| 286.7 | Other and unspecified coagulation defects | Hematopoietic | 1.06 | 0.97 | 1.17 | 325902 | 405 | 325497 |
| 578.8 | Hemorrhage of rectum and anus | Digestive | 0.99 | 0.97 | 1.01 | 317220 | 13826 | 303394 |
| 614.33 | Pelvic inflammatory disease, NOS | Genitourinary | 0.98 | 0.96 | 1.01 | 324944 | 6145 | 318799 |
| 627 | Menopausal and postmenopausal disorders | Genitourinary | 1.04 | 0.98 | 1.12 | 297078 | 847 | 296231 |
| 949 | Allergies, other | Injuries & Poisonings | 1.06 | 0.97 | 1.17 | 322326 | 420 | 321906 |
| 112 | Candidiasis | Infectious Diseases | 0.97 | 0.93 | 1.02 | 327650 | 2150 | 325500 |
| 819 | Skull and face fracture and other intercranial injury | Injuries & Poisonings | 1.03 | 0.98 | 1.09 | 328056 | 1517 | 326539 |
| 429.2 | Abnormal function study of cardiovascular system | Circulatory System | 0.95 | 0.87 | 1.03 | 322577 | 544 | 322033 |
| 564.9 | Personal history of diseases of digestive system | Digestive | 1.01 | 0.99 | 1.03 | 274320 | 15924 | 258396 |
| 587 | Kidney replaced by transpant | Genitourinary | 0.94 | 0.85 | 1.04 | 314477 | 398 | 314079 |
| 426.91 | Cardiac pacemaker in situ | Circulatory System | 0.97 | 0.94 | 1.02 | 301837 | 2316 | 299521 |
| 530.12 | Ulcer of esophagus | Digestive | 0.98 | 0.96 | 1.01 | 293151 | 5348 | 287803 |
| 597.1 | Urethral stricture (not specified as infectious) | Genitourinary | 1.02 | 0.99 | 1.06 | 317079 | 3443 | 313636 |
| 79 | Viral infection | Infectious Diseases | 1.02 | 0.99 | 1.05 | 326439 | 4233 | 322206 |
| 276.14 | Hypopotassemia | Endocrine/Metabolic | 0.97 | 0.92 | 1.02 | 322115 | 1471 | 320644 |
| 611.3 | Lump or mass in breast | Genitourinary | 1.03 | 0.98 | 1.08 | 322461 | 1588 | 320873 |
| 610.1 | Cystic mastopathy | Genitourinary | 1.04 | 0.98 | 1.11 | 321832 | 959 | 320873 |

| | | | | | | | | | |
|---|---|---|---|---|---|---|---|---|---|
| 269 | Proteinuria | Endocrine/Metabolic | 1.07 | 0.96 | 1.18 | 327850 | 355 | 327495 |
| 573.3 | Hepatomegaly | Digestive | 1.07 | 0.96 | 1.20 | 318641 | 310 | 318331 |
| 362 | Other retinal disorders | Sense Organs | 0.96 | 0.90 | 1.02 | 316825 | 973 | 315852 |
| 292.4 | Altered mental status | Mental Disorders | 1.03 | 0.98 | 1.07 | 323742 | 2272 | 321470 |
| 560.3 | Peritoneal or intestinal adhesions | Digestive | 1.04 | 0.97 | 1.11 | 259257 | 861 | 258396 |
| 250.11 | Type 1 diabetes with ketoacidosis | Endocrine/Metabolic | 1.08 | 0.95 | 1.22 | 307745 | 255 | 307490 |
| 550.5 | Ventral hernia | Digestive | 1.02 | 0.99 | 1.05 | 283371 | 3582 | 279789 |
| 202.21 | Nodular lymphoma | Neoplasms | 1.06 | 0.96 | 1.17 | 323913 | 390 | 323523 |
| 989 | Toxic effect of other substances, chiefly nonmedicinal as to source | Injuries & Poisonings | 1.03 | 0.98 | 1.09 | 328136 | 1438 | 326698 |
| 345.1 | Epilepsy | Neurological | 1.07 | 0.96 | 1.19 | 286483 | 331 | 286152 |
| 379.2 | Disorders of vitreous body | Sense Organs | 1.03 | 0.98 | 1.09 | 289599 | 1407 | 288192 |
| 297.2 | Suicide or self-inflicted injury | Mental Disorders | 1.02 | 0.98 | 1.06 | 285655 | 2757 | 282898 |
| 380.4 | Impacted cerumen | Sense Organs | 0.94 | 0.85 | 1.04 | 327106 | 354 | 326752 |
| 281.13 | Folate-deficiency anemia | Hematopoietic | 1.08 | 0.94 | 1.24 | 309101 | 201 | 308900 |
| 250.42 | Other abnormal glucose | Endocrine/Metabolic | 0.95 | 0.86 | 1.04 | 307927 | 437 | 307490 |
| 519.2 | Respiratory complications | Respiratory | 0.94 | 0.84 | 1.05 | 265743 | 296 | 265447 |
| 333.4 | Torsion dystonia | Neurological | 0.93 | 0.83 | 1.05 | 286417 | 265 | 286152 |
| 353 | Nerve root and plexus disorders | Neurological | 1.04 | 0.97 | 1.10 | 314005 | 994 | 313011 |
| 585.31 | Renal dialysis | Genitourinary | 0.95 | 0.87 | 1.04 | 314603 | 524 | 314079 |
| 550.1 | Inguinal hernia | Digestive | 0.99 | 0.97 | 1.01 | 296174 | 16385 | 279789 |
| 157 | Pancreatic cancer | Neoplasms | 1.05 | 0.97 | 1.13 | 312933 | 602 | 312331 |
| 716.2 | Unspecified monoarthritis | Musculoskeletal | 1.01 | 0.99 | 1.03 | 289797 | 15901 | 273896 |
| 338.2 | Chronic pain | Neurological | 0.95 | 0.86 | 1.04 | 327830 | 404 | 327426 |
| 411.9 | Other acute and subacute forms of ischemic heart disease | Circulatory System | 0.97 | 0.91 | 1.02 | 296883 | 1194 | 295689 |
| 483 | Acute bronchitis and bronchiolitis | Respiratory | 1.08 | 0.94 | 1.24 | 317889 | 200 | 317689 |
| 622 | Polyp of female genital organs | Genitourinary | 0.95 | 0.87 | 1.04 | 315794 | 487 | 315307 |
| 556 | Ulceration of the lower GI tract | Digestive | 0.95 | 0.86 | 1.04 | 258836 | 440 | 258396 |
| 579.2 | Splenomegaly | Digestive | 1.05 | 0.96 | 1.16 | 303837 | 443 | 303394 |
| 577.2 | Chronic pancreatitis | Digestive | 1.05 | 0.96 | 1.14 | 326456 | 536 | 325920 |
| 433.3 | Cerebral ischemia | Circulatory System | 0.96 | 0.90 | 1.03 | 319041 | 934 | 318107 |
| 415.21 | Primary pulmonary hypertension | Circulatory System | 1.05 | 0.96 | 1.15 | 322013 | 466 | 321547 |
| 440.9 | Atherosclerosis of aorta | Circulatory System | 0.93 | 0.81 | 1.06 | 319562 | 202 | 319360 |
| 979 | Adverse drug events and drug allergies | Injuries & Poisonings | 0.96 | 0.89 | 1.03 | 300927 | 716 | 300211 |
| 276.11 | Hyperosmolality and/or hypernatremia | Endocrine/Metabolic | 0.93 | 0.82 | 1.06 | 320891 | 247 | 320644 |
| 653 | Problems associated with amniotic cavity and membranes | Pregnancy Complications | 0.97 | 0.92 | 1.02 | 328119 | 1523 | 326596 |
| 465 | Acute upper respiratory infections of multiple or unspecified sites | Respiratory | 0.98 | 0.95 | 1.02 | 328232 | 3053 | 325179 |
| 530.14 | Reflux esophagitis | Digestive | 0.99 | 0.97 | 1.01 | 298696 | 10893 | 287803 |
| 427.5 | Arrhythmia (cardiac) NOS | Circulatory System | 0.97 | 0.91 | 1.03 | 300469 | 948 | 299521 |

| | | | | | | | | |
|---|---|---|---|---|---|---|---|---|
| 441 | Vascular insufficiency of intestine | Circulatory System | 0.94 | 0.84 | 1.05 | 319657 | 297 | 319360 |
| 368.1 | Amblyopia | Sense Organs | 0.95 | 0.88 | 1.04 | 325423 | 541 | 324882 |
| 200.1 | Polycythemia vera | Neoplasms | 0.95 | 0.86 | 1.05 | 318136 | 404 | 317732 |
| 476 | Allergic rhinitis | Respiratory | 1.03 | 0.97 | 1.10 | 310025 | 1075 | 308950 |
| 961 | Poisoning by other anti-infectives | Injuries & Poisonings | 0.95 | 0.87 | 1.04 | 300647 | 436 | 300211 |
| 621 | Endometrial hyperplasia | Genitourinary | 1.03 | 0.97 | 1.09 | 316461 | 1154 | 315307 |
| 352.1 | Trigeminal nerve disorders [CN5] | Neurological | 0.95 | 0.87 | 1.04 | 313473 | 462 | 313011 |
| 526.41 | Temporomandibular joint disorder, unspecified | Digestive | 1.07 | 0.94 | 1.22 | 311886 | 228 | 311658 |
| 823 | Fracture of tibia and fibula | Injuries & Poisonings | 0.94 | 0.85 | 1.05 | 322362 | 342 | 322020 |
| 681.2 | Cellulitis and abscess of face/neck | Dermatologic | 0.96 | 0.88 | 1.04 | 316936 | 529 | 316407 |
| 497 | Bronchitis | Respiratory | 1.04 | 0.96 | 1.13 | 294834 | 642 | 294192 |
| 599 | Other symptoms/disorders or the urinary system | Genitourinary | 1.00 | 1.00 | 1.01 | 324256 | 103829 | 220427 |
| 601.8 | Other inflammatory disorders of male genital organs | Genitourinary | 0.94 | 0.84 | 1.05 | 307698 | 305 | 307393 |
| 615 | Endometriosis | Genitourinary | 0.98 | 0.95 | 1.01 | 322888 | 4089 | 318799 |
| 555.1 | Regional enteritis | Digestive | 1.02 | 0.98 | 1.07 | 260201 | 1805 | 258396 |
| 596.1 | Bladder neck obstruction | Genitourinary | 1.02 | 0.98 | 1.07 | 315665 | 2029 | 313636 |
| 599.3 | Dysuria | Genitourinary | 1.03 | 0.97 | 1.09 | 221636 | 1209 | 220427 |
| 573.7 | Abnormal results of function study of liver | Digestive | 1.02 | 0.98 | 1.05 | 321896 | 3565 | 318331 |
| 689 | Disorder of skin and subcutaneous tissue NOS | Dermatologic | 1.01 | 1.00 | 1.02 | 328240 | 42194 | 286046 |
| 655 | Known or suspected fetal abnormality affecting management of mother | Pregnancy Complications | 1.02 | 0.99 | 1.05 | 328240 | 4575 | 323665 |
| 255.21 | Glucocorticoid deficiency | Endocrine/Metabolic | 1.05 | 0.95 | 1.17 | 324917 | 372 | 324545 |
| 395.6 | Heart valve replaced | Circulatory System | 0.97 | 0.93 | 1.02 | 323056 | 1528 | 321528 |
| 751.21 | Cystic kidney disease | Congenital Anomalies | 0.95 | 0.87 | 1.05 | 326461 | 458 | 326003 |
| 367.2 | Astigmatism | Sense Organs | 1.07 | 0.94 | 1.23 | 325964 | 210 | 325754 |
| 334.2 | Anterior horn cell disease | Neurological | 1.07 | 0.94 | 1.22 | 286376 | 224 | 286152 |
| 853 | Complication of colostomy or enterostomy | Injuries & Poisonings | 1.05 | 0.96 | 1.14 | 314478 | 514 | 313964 |
| 331.9 | Cerebral degeneration, unspecified | Neurological | 1.05 | 0.95 | 1.16 | 286569 | 417 | 286152 |
| 442.11 | Abdominal aortic aneurysm | Circulatory System | 0.97 | 0.91 | 1.03 | 320257 | 897 | 319360 |
| 574.12 | Cholelithiasis with other cholecystitis | Digestive | 0.99 | 0.96 | 1.01 | 315816 | 5493 | 310323 |
| 559 | Ileostomy status | Digestive | 1.02 | 0.98 | 1.08 | 260069 | 1673 | 258396 |
| 276.6 | Fluid overload | Endocrine/Metabolic | 0.95 | 0.87 | 1.04 | 321117 | 473 | 320644 |
| 226 | Benign neoplasm of thyroid glands | Neoplasms | 1.06 | 0.94 | 1.20 | 326908 | 266 | 326642 |
| 564 | Functional digestive disorders | Digestive | 0.97 | 0.91 | 1.03 | 259379 | 983 | 258396 |
| 481 | Influenza | Respiratory | 1.01 | 0.99 | 1.03 | 327706 | 10017 | 317689 |
| 578.1 | Hematemesis | Digestive | 0.98 | 0.94 | 1.02 | 305428 | 2034 | 303394 |
| 427.41 | Ventricular fibrillation and flutter | Circulatory System | 1.06 | 0.95 | 1.17 | 299866 | 345 | 299521 |
| 187.2 | Malignant neoplasm of testis | Neoplasms | 0.99 | 0.96 | 1.01 | 323790 | 4572 | 319218 |
| 41.4 | E. coli | Infectious Diseases | 0.98 | 0.95 | 1.02 | 315432 | 2800 | 312632 |

| | | | | | | | | |
|---|---|---|---|---|---|---|---|---|
| 368.4 | Visual field defects | Sense Organs | 0.95 | 0.85 | 1.05 | 325215 | 333 | 324882 |
| 155 | Cancer of liver and intrahepatic bile duct | Neoplasms | 1.07 | 0.94 | 1.21 | 312576 | 245 | 312331 |
| 726 | Peripheral enthesopathies and allied syndromes | Musculoskeletal | 1.01 | 0.99 | 1.03 | 313722 | 8768 | 304954 |
| 530 | Diseases of esophagus | Digestive | 1.04 | 0.97 | 1.11 | 288604 | 801 | 287803 |
| 334 | Degenerative disease of the spinal cord | Neurological | 1.03 | 0.98 | 1.08 | 287631 | 1479 | 286152 |
| 430.3 | Subdural hemorrhage | Circulatory System | 0.94 | 0.84 | 1.06 | 318378 | 271 | 318107 |
| 580.32 | Nephritis and nephropathy with pathological lesion | Genitourinary | 0.97 | 0.91 | 1.03 | 315157 | 1078 | 314079 |
| 514 | Abnormal findings examination of lungs | Respiratory | 1.02 | 0.98 | 1.06 | 328212 | 2577 | 325635 |
| 571.81 | Portal hypertension | Digestive | 1.04 | 0.96 | 1.13 | 318900 | 569 | 318331 |
| 427.6 | Premature beats | Circulatory System | 0.96 | 0.87 | 1.05 | 299979 | 458 | 299521 |
| 433.31 | Transient cerebral ischemia | Circulatory System | 0.98 | 0.94 | 1.02 | 320281 | 2174 | 318107 |
| 523.32 | Chronic periodontitis | Digestive | 1.04 | 0.96 | 1.13 | 312248 | 590 | 311658 |
| 458 | Hypotension | Circulatory System | 0.97 | 0.91 | 1.03 | 195260 | 1081 | 194179 |
| 172.11 | Melanomas of skin | Neoplasms | 1.02 | 0.98 | 1.06 | 316943 | 2723 | 314220 |
| 170.2 | Cancer of connective tissue | Neoplasms | 0.96 | 0.90 | 1.04 | 328122 | 734 | 327388 |
| 458.1 | Orthostatic hypotension | Circulatory System | 0.97 | 0.92 | 1.03 | 195553 | 1374 | 194179 |
| 198.1 | Secondary malignancy of lymph nodes | Neoplasms | 1.01 | 0.99 | 1.04 | 234815 | 5503 | 229312 |
| 565.1 | Anal and rectal polyp | Digestive | 0.99 | 0.97 | 1.01 | 254329 | 7669 | 246660 |
| 614.52 | Vaginitis and vulvovaginitis | Genitourinary | 0.95 | 0.87 | 1.05 | 319219 | 420 | 318799 |
| 614.3 | Pelvic inflammatory disease (PID) | Genitourinary | 0.94 | 0.83 | 1.07 | 319048 | 249 | 318799 |
| 172.3 | Carcinoma in situ of skin | Neoplasms | 1.04 | 0.96 | 1.12 | 314884 | 664 | 314220 |
| 117 | Mycoses | Infectious Diseases | 0.98 | 0.95 | 1.02 | 328235 | 2735 | 325500 |
| 614 | Inflammatory diseases of female pelvic organs | Genitourinary | 0.95 | 0.86 | 1.05 | 319173 | 374 | 318799 |
| 560.1 | Paralytic ileus | Digestive | 0.96 | 0.88 | 1.04 | 258916 | 520 | 258396 |
| 704 | Diseases of hair and hair follicles | Dermatologic | 1.01 | 0.99 | 1.04 | 326692 | 5263 | 321429 |
| 348.8 | Encephalopathy, not elsewhere classified | Neurological | 1.07 | 0.93 | 1.23 | 286356 | 204 | 286152 |
| 626.13 | Irregular menstrual cycle | Genitourinary | 1.02 | 0.98 | 1.07 | 298189 | 1958 | 296231 |
| 380.1 | Otitis externa | Sense Organs | 1.04 | 0.96 | 1.13 | 327284 | 532 | 326752 |
| 961.1 | Poisoning/allergy of sulfonamides | Injuries & Poisonings | 0.97 | 0.91 | 1.03 | 301101 | 890 | 300211 |
| 317.11 | Alcoholic liver damage | Mental Disorders | 1.03 | 0.97 | 1.10 | 298534 | 865 | 297669 |
| 579.8 | Nonspecific abnormal findings in stool contents | Digestive | 1.02 | 0.98 | 1.07 | 305134 | 1740 | 303394 |
| 503 | Pulmonary congestion and hypostasis | Respiratory | 0.96 | 0.88 | 1.05 | 316959 | 475 | 316484 |
| 362.2 | Degeneration of macula and posterior pole of retina | Sense Organs | 0.98 | 0.94 | 1.02 | 318055 | 2203 | 315852 |
| 605 | Erectile dysfunction [ED] | Genitourinary | 1.06 | 0.94 | 1.19 | 308197 | 291 | 307906 |
| 446.9 | Arteritis NOS | Circulatory System | 0.94 | 0.83 | 1.07 | 319605 | 245 | 319360 |
| 872 | Traumatic amputation | Injuries & Poisonings | 0.96 | 0.88 | 1.05 | 320213 | 482 | 319731 |
| 557.1 | Celiac disease | Digestive | 0.98 | 0.94 | 1.02 | 260290 | 1894 | 258396 |
| 716.1 | Unspecified polyarthropathy or polyarthritis | Musculoskeletal | 1.02 | 0.98 | 1.05 | 277426 | 3530 | 273896 |

| | | | | | | | | | |
|---|---|---|---|---|---|---|---|---|---|
| 362.29 | Macular degeneration (senile) of retina NOS | Sense Organs | 0.98 | 0.94 | 1.02 | 318052 | 2200 | 315852 |
| 568.1 | Peritoneal adhesions (postoperative) (postinfection) | Digestive | 0.98 | 0.95 | 1.02 | 249757 | 3097 | 246660 |
| 747.13 | Congenital anomalies of great vessels | Congenital Anomalies | 0.98 | 0.93 | 1.02 | 327177 | 1823 | 325354 |
| 251.1 | Hypoglycemia | Endocrine/Metabolic | 1.03 | 0.97 | 1.10 | 305952 | 977 | 304975 |
| 193 | Thyroid cancer | Neoplasms | 0.95 | 0.86 | 1.06 | 327009 | 367 | 326642 |
| 535.1 | Acute gastritis | Digestive | 0.97 | 0.92 | 1.03 | 297704 | 1193 | 296511 |
| 709.2 | Sicca syndrome | Dermatologic | 1.04 | 0.96 | 1.13 | 242486 | 520 | 241966 |
| 610.2 | Fibroadenosis of breast | Genitourinary | 0.95 | 0.85 | 1.06 | 321189 | 316 | 320873 |
| 830 | Dislocation | Injuries & Poisonings | 1.02 | 0.98 | 1.06 | 323927 | 2154 | 321773 |
| 182 | Malignant neoplasm of uterus | Neoplasms | 1.03 | 0.97 | 1.08 | 298948 | 1303 | 297645 |
| 312 | Conduct disorders | Mental Disorders | 1.03 | 0.97 | 1.09 | 327638 | 1195 | 326443 |
| 742.8 | Articular cartilage disorder | Musculoskeletal | 1.04 | 0.96 | 1.12 | 311297 | 655 | 310642 |
| 599.5 | Frequency of urination and polyuria | Genitourinary | 0.99 | 0.96 | 1.02 | 224574 | 4147 | 220427 |
| 386.1 | Meniere's disease | Sense Organs | 0.96 | 0.89 | 1.04 | 322213 | 564 | 321649 |
| 578.2 | Blood in stool | Digestive | 1.02 | 0.98 | 1.06 | 306143 | 2749 | 303394 |
| 242 | Thyrotoxicosis with or without goiter | Endocrine/Metabolic | 0.98 | 0.93 | 1.03 | 311794 | 1465 | 310329 |
| 636.3 | Hemorrhage in early pregnancy | Pregnancy Complications | 1.03 | 0.97 | 1.09 | 321078 | 1113 | 319965 |
| 625 | Pain and other symptoms associated with female genital organs | Genitourinary | 0.98 | 0.94 | 1.02 | 320718 | 2049 | 318669 |
| 447 | Other disorders of arteries and arterioles | Circulatory System | 0.96 | 0.89 | 1.05 | 319916 | 556 | 319360 |
| 626 | Disorders of menstruation and other abnormal bleeding from female genital tract | Genitourinary | 1.01 | 0.98 | 1.05 | 300178 | 3947 | 296231 |
| 292 | Neurological disorders | Mental Disorders | 1.04 | 0.95 | 1.14 | 321919 | 449 | 321470 |
| 619.1 | Noninflammatory disorders of ovary, fallopian tube, and broad ligament | Genitourinary | 0.97 | 0.90 | 1.04 | 279282 | 734 | 278548 |
| 569 | Other disorders of intestine | Digestive | 1.00 | 1.00 | 1.01 | 326148 | 79488 | 246660 |
| 369 | Infection of the eye | Sense Organs | 1.06 | 0.94 | 1.20 | 318642 | 254 | 318388 |
| 418.1 | Precordial pain | Circulatory System | 0.99 | 0.95 | 1.02 | 299506 | 3686 | 295820 |
| 941 | Adverse reaction to serum or vaccine | Injuries & Poisonings | 0.94 | 0.83 | 1.07 | 322128 | 222 | 321906 |
| 614.5 | Inflammatory disease of cervix, vagina, and vulva | Genitourinary | 1.05 | 0.94 | 1.17 | 319133 | 334 | 318799 |
| 241.2 | Nontoxic multinodular goiter | Endocrine/Metabolic | 0.97 | 0.90 | 1.04 | 311043 | 714 | 310329 |
| 697 | Sarcoidosis | Dermatologic | 1.04 | 0.96 | 1.13 | 322309 | 560 | 321749 |
| 510 | Other diseases of lung | Respiratory | 0.97 | 0.90 | 1.04 | 328200 | 770 | 327430 |
| 618 | Genital prolapse | Genitourinary | 0.97 | 0.89 | 1.04 | 316862 | 620 | 316242 |
| 965.3 | Salicylates causing adverse effects in therapeutic use | Injuries & Poisonings | 0.96 | 0.87 | 1.06 | 300612 | 401 | 300211 |
| 263 | Other nutritional deficiency | Endocrine/Metabolic | 0.98 | 0.93 | 1.03 | 327337 | 1551 | 325786 |
| 331.1 | Hydrocephalus | Neurological | 0.96 | 0.88 | 1.05 | 286652 | 500 | 286152 |
| 261.2 | Vitamin B-complex deficiencies | Endocrine/Metabolic | 0.97 | 0.90 | 1.04 | 326559 | 773 | 325786 |
| 528.5 | Diseases of lips | Digestive | 1.03 | 0.96 | 1.11 | 323215 | 681 | 322534 |
| 695.7 | Prurigo and Lichen | Dermatologic | 0.97 | 0.90 | 1.04 | 322541 | 792 | 321749 |
| 383 | Otosclerosis | Sense Organs | 1.05 | 0.94 | 1.17 | 324473 | 330 | 324143 |

| | | | | | | | | | |
|---|---|---|---|---|---|---|---|---|---|
| 158 | Neoplasm of unspecified nature of digestive system | Neoplasms | 0.97 | 0.92 | 1.03 | 313429 | 1098 | 312331 |
| 575.2 | Obstruction of bile duct | Digestive | 1.03 | 0.96 | 1.10 | 311135 | 812 | 310323 |
| 560.2 | Impaction of intestine | Digestive | 1.05 | 0.94 | 1.19 | 258667 | 271 | 258396 |
| 610.8 | Other specified benign mammary dysplasias | Genitourinary | 1.03 | 0.96 | 1.11 | 321576 | 703 | 320873 |
| 785 | Abdominal pain | Symptoms | 1.00 | 0.99 | 1.01 | 328240 | 42311 | 285929 |
| 603 | Other disorders of testis | Genitourinary | 1.03 | 0.97 | 1.09 | 308895 | 989 | 307906 |
| 295.1 | Schizophrenia | Mental Disorders | 1.03 | 0.96 | 1.12 | 283509 | 611 | 282898 |
| 451.2 | Phlebitis and thrombophlebitis of lower extremities | Circulatory System | 1.01 | 0.98 | 1.05 | 291357 | 3644 | 287713 |
| 530.1 | Esophagitis, GERD and related diseases | Digestive | 0.99 | 0.97 | 1.01 | 297342 | 9539 | 287803 |
| 592.12 | Chronic cystitis | Genitourinary | 1.03 | 0.96 | 1.10 | 297948 | 913 | 297035 |
| 296.2 | Depression | Mental Disorders | 1.01 | 0.99 | 1.03 | 295043 | 12145 | 282898 |
| 427.11 | Paroxysmal supraventricular tachycardia | Circulatory System | 0.98 | 0.94 | 1.02 | 301952 | 2431 | 299521 |
| 737.3 | Kyphoscoliosis and scoliosis | Musculoskeletal | 0.96 | 0.87 | 1.06 | 317058 | 371 | 316687 |
| 681.3 | Cellulitis and abscess of arm/hand | Dermatologic | 0.99 | 0.96 | 1.01 | 322177 | 5770 | 316407 |
| 427.4 | Cardiac arrest and ventricular fibrillation | Circulatory System | 1.05 | 0.94 | 1.16 | 299869 | 348 | 299521 |
| 374.1 | Ectropion or entropion | Sense Organs | 1.03 | 0.97 | 1.09 | 319480 | 1092 | 318388 |
| 740.2 | Osteoarthrosis, generalized | Musculoskeletal | 0.97 | 0.89 | 1.05 | 306914 | 589 | 306325 |
| 395.2 | Nonrheumatic aortic valve disorders | Circulatory System | 0.95 | 0.84 | 1.07 | 321775 | 247 | 321528 |
| 275.1 | Disorders of iron metabolism | Hematopoietic | 1.03 | 0.96 | 1.11 | 326735 | 700 | 326035 |
| 451 | Phlebitis and thrombophlebitis | Circulatory System | 0.96 | 0.87 | 1.06 | 288110 | 397 | 287713 |
| 966 | Poisoning by anticonvulsants and anti-Parkinsonism drugs | Injuries & Poisonings | 1.04 | 0.95 | 1.13 | 300747 | 536 | 300211 |
| 420.3 | Endocarditis | Circulatory System | 0.97 | 0.90 | 1.04 | 325668 | 685 | 324983 |
| 8 | Intestinal infection | Infectious Diseases | 1.01 | 0.99 | 1.03 | 328240 | 9161 | 319079 |
| 727.1 | Synovitis and tenosynovitis | Musculoskeletal | 1.03 | 0.96 | 1.11 | 305641 | 687 | 304954 |
| 426.9 | Cardiac pacemaker/device in situ | Circulatory System | 1.05 | 0.93 | 1.20 | 299758 | 237 | 299521 |
| 394.3 | Aortic valve disease | Circulatory System | 0.98 | 0.93 | 1.03 | 322836 | 1308 | 321528 |
| 201 | Hodgkin's disease | Neoplasms | 0.95 | 0.85 | 1.07 | 323794 | 271 | 323523 |
| 261.4 | Vitamin D deficiency | Endocrine/Metabolic | 0.96 | 0.87 | 1.06 | 326194 | 408 | 325786 |
| 800 | Fracture of lower limb | Injuries & Poisonings | 0.97 | 0.90 | 1.04 | 324256 | 699 | 323557 |
| 151 | Cancer of stomach | Neoplasms | 1.03 | 0.95 | 1.12 | 312905 | 574 | 312331 |
| 480 | Pneumonia | Respiratory | 0.99 | 0.96 | 1.02 | 321603 | 3914 | 317689 |
| 184.11 | Malignant neoplasm of ovary | Neoplasms | 0.99 | 0.96 | 1.02 | 310562 | 5009 | 305553 |
| 306.9 | Tension headache | Mental Disorders | 0.96 | 0.85 | 1.07 | 283203 | 305 | 282898 |
| 292.3 | Memory loss | Mental Disorders | 0.97 | 0.90 | 1.04 | 322208 | 738 | 321470 |
| 454 | Varicose veins | Circulatory System | 1.02 | 0.97 | 1.07 | 289316 | 1603 | 287713 |
| 766 | Neuralgia, neuritis, and radiculitis NOS | Symptoms | 1.05 | 0.93 | 1.17 | 326987 | 304 | 326683 |
| 341 | Other demyelinating diseases of central nervous system | Neurological | 1.02 | 0.97 | 1.07 | 287764 | 1612 | 286152 |
| 296.1 | Bipolar | Mental Disorders | 1.02 | 0.97 | 1.08 | 284019 | 1121 | 282898 |

| | | | | | | | | | |
|---|---|---|---|---|---|---|---|---|---|
| 623 | Hypertrophy of female genital organs | Genitourinary | 1.02 | 0.97 | 1.08 | 316579 | 1272 | 315307 |
| 286.12 | Congenital deficiency of other clotting factors (including factor VII) | Hematopoietic | 0.96 | 0.86 | 1.07 | 325800 | 303 | 325497 |
| 296 | Mood disorders | Mental Disorders | 1.01 | 0.99 | 1.02 | 295853 | 12955 | 282898 |
| 965 | Poisoning by analgesics, antipyretics, and antirheumatics | Injuries & Poisonings | 0.99 | 0.97 | 1.02 | 306293 | 6082 | 300211 |
| 221 | Benign neoplasm of other female genital organs | Neoplasms | 0.95 | 0.83 | 1.08 | 295974 | 223 | 295751 |
| 512.2 | Painful respiration | Respiratory | 1.05 | 0.92 | 1.20 | 314496 | 228 | 314268 |
| 290.2 | Delirium due to conditions classified elsewhere | Mental Disorders | 0.97 | 0.90 | 1.05 | 322151 | 681 | 321470 |
| 136 | Other infectious and parasitic diseases | Infectious Diseases | 1.04 | 0.94 | 1.14 | 327982 | 428 | 327554 |
| 715.2 | Ankylosing spondylitis | Musculoskeletal | 0.96 | 0.87 | 1.06 | 317637 | 384 | 317253 |
| 367.9 | Blindness and low vision | Sense Organs | 0.97 | 0.91 | 1.05 | 326510 | 756 | 325754 |
| 512.7 | Shortness of breath | Respiratory | 1.01 | 0.98 | 1.04 | 320384 | 6116 | 314268 |
| 695.9 | Unspecified erythematous condition | Dermatologic | 0.97 | 0.88 | 1.06 | 322226 | 477 | 321749 |
| 550.3 | Femoral hernia | Digestive | 1.03 | 0.95 | 1.11 | 280442 | 653 | 279789 |
| 622.1 | Polyp of corpus uteri | Genitourinary | 1.01 | 0.99 | 1.03 | 323490 | 8183 | 315307 |
| 285 | Other anemias | Hematopoietic | 1.01 | 0.99 | 1.03 | 320744 | 11844 | 308900 |
| 736.2 | Acquired deformities of finger | Musculoskeletal | 0.95 | 0.84 | 1.08 | 316940 | 253 | 316687 |
| 626.1 | Irregular menstrual cycle/bleeding | Genitourinary | 1.01 | 0.98 | 1.05 | 299824 | 3593 | 296231 |
| 520 | Disorders of tooth development | Digestive | 0.97 | 0.88 | 1.06 | 312128 | 470 | 311658 |
| 550.2 | Diaphragmatic hernia | Digestive | 1.00 | 0.99 | 1.02 | 307453 | 27664 | 279789 |
| 371.3 | Inflammation of eyelids | Sense Organs | 0.99 | 0.95 | 1.03 | 320786 | 2398 | 318388 |
| 200 | Myeloproliferative disease | Neoplasms | 0.97 | 0.90 | 1.05 | 324202 | 679 | 323523 |
| 289.5 | Diseases of spleen | Hematopoietic | 0.97 | 0.89 | 1.06 | 320846 | 528 | 320318 |
| 262 | Mineral deficiency NEC | Endocrine/Metabolic | 1.05 | 0.92 | 1.19 | 326027 | 241 | 325786 |
| 560.4 | Other intestinal obstruction | Digestive | 1.01 | 0.98 | 1.05 | 261839 | 3443 | 258396 |
| 564.1 | Irritable Bowel Syndrome | Digestive | 1.01 | 0.98 | 1.04 | 264057 | 5661 | 258396 |
| 916 | Contusion | Injuries & Poisonings | 0.98 | 0.93 | 1.03 | 328240 | 1478 | 326762 |
| 686 | Other local infections of skin and subcutaneous tissue | Dermatologic | 1.01 | 0.99 | 1.03 | 327410 | 11003 | 316407 |
| 427.7 | Tachycardia NOS | Circulatory System | 1.01 | 0.97 | 1.06 | 301787 | 2266 | 299521 |
| 595 | Hydronephrosis | Genitourinary | 1.02 | 0.97 | 1.06 | 321941 | 2010 | 319931 |
| 809 | Fracture of unspecified bones | Injuries & Poisonings | 0.97 | 0.88 | 1.06 | 323967 | 410 | 323557 |
| 361 | Retinal detachments and defects | Sense Organs | 1.02 | 0.97 | 1.06 | 319011 | 2229 | 316782 |
| 364 | Corneal opacity and other disorders of cornea | Sense Organs | 1.05 | 0.92 | 1.19 | 317009 | 227 | 316782 |
| 277 | Other disorders of metabolism | Endocrine/Metabolic | 1.00 | 0.99 | 1.01 | 328230 | 45303 | 282927 |
| 218.1 | Uterine leiomyoma | Neoplasms | 0.99 | 0.97 | 1.01 | 318346 | 10566 | 307780 |
| 189.2 | Cancer of bladder | Neoplasms | 1.02 | 0.96 | 1.10 | 324798 | 836 | 323962 |
| 153.2 | Colon cancer | Neoplasms | 0.99 | 0.95 | 1.02 | 304402 | 3122 | 301280 |
| 327.3 | Sleep apnea | Neurological | 1.01 | 0.98 | 1.04 | 327419 | 4699 | 322720 |
| 681.5 | Cellulitis and abscess of leg, except foot | Dermatologic | 0.99 | 0.97 | 1.02 | 322184 | 5777 | 316407 |

| | | | | | | | | |
|---|---|---|---|---|---|---|---|---|
| 189.4 | Malignant neoplasm of other urinary organs | Neoplasms | 1.01 | 0.98 | 1.05 | 327185 | 3223 | 323962 |
| 389.2 | Conductive hearing loss | Sense Organs | 0.97 | 0.88 | 1.06 | 318629 | 435 | 318194 |
| 960.2 | Allergy/adverse effect of penicillin | Injuries & Poisonings | 0.99 | 0.98 | 1.01 | 316894 | 16683 | 300211 |
| 613.8 | Other specified disorders of breast | Genitourinary | 0.96 | 0.87 | 1.07 | 322773 | 355 | 322418 |
| 342 | Hemiplegia | Neurological | 0.98 | 0.93 | 1.03 | 287677 | 1525 | 286152 |
| 474.2 | Chronic tonsillitis and adenoiditis | Respiratory | 1.02 | 0.96 | 1.08 | 310135 | 1185 | 308950 |
| 681.6 | Cellulitis and abscess of foot, toe | Dermatologic | 0.99 | 0.97 | 1.02 | 322141 | 5734 | 316407 |
| 771 | Musculoskeletal symptoms referable to limbs | Symptoms | 1.02 | 0.96 | 1.09 | 327405 | 891 | 326514 |
| 389.1 | Sensorineural hearing loss | Sense Organs | 0.97 | 0.88 | 1.06 | 318658 | 464 | 318194 |
| 344 | Other paralytic syndromes | Neurological | 0.97 | 0.90 | 1.05 | 286841 | 689 | 286152 |
| 706.2 | Sebaceous cyst | Dermatologic | 0.99 | 0.97 | 1.01 | 327401 | 9107 | 318294 |
| 790.6 | Other abnormal blood chemistry | Symptoms | 0.99 | 0.97 | 1.02 | 328029 | 6529 | 321500 |
| 180.1 | Cervical cancer | Neoplasms | 0.99 | 0.96 | 1.02 | 302137 | 4554 | 297583 |
| 696.4 | Psoriasis | Dermatologic | 1.02 | 0.97 | 1.07 | 315554 | 1622 | 313932 |
| 70.9 | Hepatitis NOS | Infectious Diseases | 1.03 | 0.94 | 1.13 | 322658 | 452 | 322206 |
| 242.1 | Graves' disease | Endocrine/Metabolic | 0.97 | 0.88 | 1.06 | 310789 | 460 | 310329 |
| 293 | Symptoms involving head and neck | Mental Disorders | 1.01 | 0.97 | 1.06 | 327323 | 2204 | 325119 |
| 276.13 | Hyperpotassemia | Endocrine/Metabolic | 0.98 | 0.92 | 1.04 | 321623 | 979 | 320644 |
| 557 | Intestinal malabsorption (non-celiac) | Digestive | 1.04 | 0.93 | 1.17 | 258690 | 294 | 258396 |
| 327 | Sleep disorders | Neurological | 1.02 | 0.96 | 1.09 | 323754 | 1034 | 322720 |
| 745 | Pain in joint | Musculoskeletal | 0.98 | 0.94 | 1.03 | 328240 | 1940 | 326300 |
| 217.1 | Nevus, non-neoplastic | Neoplasms | 0.97 | 0.90 | 1.05 | 320346 | 597 | 319749 |
| 627.1 | Postmenopausal bleeding | Genitourinary | 1.01 | 0.99 | 1.03 | 305697 | 9466 | 296231 |
| 204.21 | Myeloid leukemia, acute | Neoplasms | 0.96 | 0.86 | 1.08 | 323842 | 319 | 323523 |
| 473.4 | Voice disturbance | Respiratory | 0.98 | 0.92 | 1.04 | 310051 | 1101 | 308950 |
| 614.54 | Abscess or ulceration of vulva | Genitourinary | 1.04 | 0.93 | 1.17 | 319079 | 280 | 318799 |
| 528.6 | Leukoplakia of oral mucosa | Digestive | 0.96 | 0.86 | 1.08 | 322846 | 312 | 322534 |
| 736 | Other acquired deformities of limbs | Musculoskeletal | 1.04 | 0.93 | 1.16 | 316998 | 311 | 316687 |
| 198 | Secondary malignant neoplasm | Neoplasms | 1.02 | 0.96 | 1.08 | 230431 | 1119 | 229312 |
| 752 | Nervous system congenital anomalies | Congenital Anomalies | 0.96 | 0.86 | 1.08 | 327963 | 288 | 327675 |
| 535.2 | Atrophic gastritis | Digestive | 1.03 | 0.93 | 1.15 | 296869 | 358 | 296511 |
| 722 | Intervertebral disc disorders | Musculoskeletal | 1.01 | 0.98 | 1.03 | 316907 | 5963 | 310944 |
| 153.3 | Malignant neoplasm of rectum, rectosigmoid junction, and anus | Neoplasms | 1.01 | 0.97 | 1.06 | 303397 | 2117 | 301280 |
| 191 | Malignant and unknown neoplasms of brain and nervous system | Neoplasms | 0.96 | 0.84 | 1.09 | 326662 | 215 | 326447 |
| 228 | Hemangioma and lymphangioma, any site | Neoplasms | 0.98 | 0.94 | 1.03 | 328240 | 1635 | 326605 |
| 599.9 | Other abnormality of urination | Genitourinary | 1.01 | 0.97 | 1.06 | 222371 | 1944 | 220427 |
| 320 | Meningitis | Neurological | 1.03 | 0.94 | 1.13 | 327577 | 439 | 327138 |
| 573.9 | Abnormal serum enzyme levels | Digestive | 0.96 | 0.84 | 1.09 | 318561 | 230 | 318331 |

| | | | | | | | | |
|---|---|---|---|---|---|---|---|---|
| 275.3 | Disorders of magnesium metabolism | Endocrine/Metabolic | 0.97 | 0.87 | 1.07 | 326410 | 375 | 326035 |
| 764 | Sciatica | Symptoms | 0.98 | 0.93 | 1.04 | 327944 | 1261 | 326683 |
| 528.7 | Sialolithiasis | Digestive | 0.96 | 0.86 | 1.08 | 322847 | 313 | 322534 |
| 569.2 | Gastrointestinal complications | Digestive | 0.97 | 0.89 | 1.06 | 247132 | 472 | 246660 |
| 70.3 | Viral hepatitis C | Infectious Diseases | 1.03 | 0.93 | 1.15 | 322549 | 343 | 322206 |
| 649.1 | Diabetes or abnormal glucose tolerance complicating pregnancy | Pregnancy Complications | 1.04 | 0.91 | 1.20 | 328127 | 208 | 327919 |
| 386.2 | Peripheral or central vertigo | Sense Organs | 1.03 | 0.93 | 1.14 | 322036 | 387 | 321649 |
| 619.3 | Noninflammatory disorders of cervix | Genitourinary | 0.99 | 0.95 | 1.03 | 281393 | 2845 | 278548 |
| 385.5 | Tympanosclerosis and middle ear disease related to otitis media | Sense Organs | 0.96 | 0.85 | 1.09 | 324379 | 236 | 324143 |
| 624.2 | Atrophy of female genital tract | Genitourinary | 0.97 | 0.89 | 1.07 | 319111 | 442 | 318669 |
| 292.6 | Hallucinations | Mental Disorders | 0.97 | 0.87 | 1.08 | 321793 | 323 | 321470 |
| 601.4 | Balanoposthitis | Genitourinary | 0.96 | 0.86 | 1.08 | 307689 | 296 | 307393 |
| 444.1 | Arterial embolism and thrombosis of lower extremity artery | Circulatory System | 1.03 | 0.95 | 1.11 | 319930 | 570 | 319360 |
| 803.3 | Fracture of clavicle or scapula | Injuries & Poisonings | 0.97 | 0.86 | 1.08 | 323857 | 300 | 323557 |
| 626.14 | Irregular menstrual bleeding | Genitourinary | 1.01 | 0.98 | 1.04 | 300177 | 3946 | 296231 |
| 281.11 | Pernicious anemia | Hematopoietic | 1.02 | 0.95 | 1.10 | 309650 | 750 | 308900 |
| 729.1 | Rheumatism, unspecified and fibrositis | Musculoskeletal | 1.03 | 0.93 | 1.14 | 305341 | 387 | 304954 |
| 189 | Cancer of urinary organs (incl. kidney and bladder) | Neoplasms | 1.01 | 0.98 | 1.05 | 326965 | 3003 | 323962 |
| 781 | Symptoms involving nervous and musculoskeletal systems | Symptoms | 1.00 | 0.99 | 1.02 | 328238 | 22180 | 306058 |
| 722.9 | Other and unspecified disc disorder | Musculoskeletal | 1.01 | 0.98 | 1.04 | 314894 | 3950 | 310944 |
| 165.1 | Cancer of bronchus; lung | Neoplasms | 1.01 | 0.97 | 1.06 | 327450 | 2181 | 325269 |
| 471 | Nasal polyps | Respiratory | 0.99 | 0.96 | 1.02 | 312342 | 3392 | 308950 |
| 396 | Abnormal heart sounds | Circulatory System | 1.02 | 0.96 | 1.08 | 322603 | 1075 | 321528 |
| 388 | Other disorders of ear | Sense Organs | 0.99 | 0.97 | 1.01 | 328080 | 9886 | 318194 |
| 272.9 | Unspecified disorder of lipoid metabolism | Endocrine/Metabolic | 0.96 | 0.84 | 1.10 | 291721 | 224 | 291497 |
| 594.1 | Calculus of kidney | Genitourinary | 1.01 | 0.98 | 1.05 | 323265 | 3334 | 319931 |
| 81 | Infection/inflammation of internal prosthetic device; implant; and graft | Infectious Diseases | 0.99 | 0.95 | 1.03 | 323993 | 2514 | 321479 |
| 625.1 | Dyspareunia | Genitourinary | 1.02 | 0.96 | 1.08 | 319824 | 1155 | 318669 |
| 278.1 | Obesity | Endocrine/Metabolic | 0.99 | 0.98 | 1.01 | 328110 | 11143 | 316967 |
| 302 | Sexual and gender identity disorders | Mental Disorders | 0.97 | 0.87 | 1.08 | 283238 | 340 | 282898 |
| 735.21 | Hammer toe (acquired) | Musculoskeletal | 0.99 | 0.94 | 1.03 | 318701 | 2014 | 316687 |
| 250.22 | Type 2 diabetes with renal manifestations | Endocrine/Metabolic | 1.04 | 0.91 | 1.19 | 307698 | 208 | 307490 |
| 427.12 | Paroxysmal ventricular tachycardia | Circulatory System | 1.02 | 0.96 | 1.08 | 300490 | 969 | 299521 |
| 575.6 | Cholesterolosis of gallbladder | Digestive | 0.97 | 0.89 | 1.07 | 310789 | 466 | 310323 |
| 443.9 | Peripheral vascular disease, unspecified | Circulatory System | 1.01 | 0.97 | 1.05 | 321969 | 2609 | 319360 |
| 195 | Cancer, suspected or other | Neoplasms | 1.02 | 0.96 | 1.09 | 230227 | 915 | 229312 |
| 433.1 | Occlusion and stenosis of precerebral arteries | Circulatory System | 1.02 | 0.96 | 1.08 | 319149 | 1042 | 318107 |
| 315 | Develomental delays and disorders | Mental Disorders | 1.02 | 0.94 | 1.11 | 326992 | 549 | 326443 |

| | | | | | | | | | |
|---|---|---|---|---|---|---|---|---|---|
| 427.42 | Cardiac arrest | Circulatory System | 0.98 | 0.92 | 1.05 | 300470 | 949 | 299521 |
| 198.2 | Secondary malignancy of respiratory organs | Neoplasms | 0.99 | 0.95 | 1.03 | 231589 | 2277 | 229312 |
| 8.6 | Viral Enteritis | Infectious Diseases | 0.98 | 0.92 | 1.05 | 319924 | 845 | 319079 |
| 531 | Peptic ulcer (excl. esophageal) | Digestive | 1.03 | 0.92 | 1.16 | 320833 | 284 | 320549 |
| 374.3 | Ptosis of eyelid | Sense Organs | 1.01 | 0.97 | 1.06 | 320284 | 1896 | 318388 |
| 454.11 | Varicose veins of lower extremity, symptomtic | Circulatory System | 1.02 | 0.95 | 1.10 | 288365 | 652 | 287713 |
| 586.4 | Stricture/obstruction of ureter | Genitourinary | 0.98 | 0.92 | 1.05 | 315018 | 939 | 314079 |
| 184.1 | Malignant neoplasm of ovary and other uterine adnexa | Neoplasms | 1.01 | 0.97 | 1.05 | 308106 | 2553 | 305553 |
| 335 | Multiple sclerosis | Neurological | 1.01 | 0.96 | 1.07 | 287531 | 1379 | 286152 |
| 721.1 | Spondylosis without myelopathy | Musculoskeletal | 1.02 | 0.96 | 1.08 | 312014 | 1070 | 310944 |
| 415 | Pulmonary heart disease | Circulatory System | 1.01 | 0.98 | 1.04 | 325300 | 3753 | 321547 |
| 695.42 | Systemic lupus erythematosus | Dermatologic | 1.03 | 0.93 | 1.14 | 320784 | 363 | 320421 |
| 751.2 | Congenital anomalies of urinary system | Congenital Anomalies | 0.98 | 0.92 | 1.05 | 326908 | 905 | 326003 |
| 751.12 | Congenital anomalies of male genital organs | Congenital Anomalies | 0.97 | 0.85 | 1.10 | 326231 | 228 | 326003 |
| 366 | Cataract | Sense Organs | 1.00 | 0.98 | 1.01 | 323825 | 16441 | 307384 |
| 381.1 | Otitis media | Sense Organs | 1.02 | 0.96 | 1.08 | 325296 | 1153 | 324143 |
| 276.41 | Acidosis | Endocrine/Metabolic | 1.02 | 0.96 | 1.08 | 321700 | 1056 | 320644 |
| 175 | Acquired absence of breast | Neoplasms | 1.01 | 0.97 | 1.05 | 305907 | 2516 | 303391 |
| 448 | Disease of capillaries | Circulatory System | 0.99 | 0.97 | 1.02 | 326841 | 7481 | 319360 |
| 519 | Other diseases of respiratory system, not elsewhere classified | Respiratory | 1.00 | 0.99 | 1.01 | 327615 | 62168 | 265447 |
| 496.3 | Bronchiectasis | Respiratory | 0.99 | 0.94 | 1.03 | 296077 | 1885 | 294192 |
| 761 | Cervicalgia | Symptoms | 0.98 | 0.93 | 1.05 | 328240 | 1005 | 327235 |
| 610.3 | Fibrosclerosis of breast | Genitourinary | 0.97 | 0.87 | 1.09 | 321176 | 303 | 320873 |
| 789 | Nausea and vomiting | Symptoms | 1.00 | 0.98 | 1.01 | 328240 | 11983 | 316257 |
| 225.1 | Benign neoplasm of brain, cranial nerves, meninges | Neoplasms | 1.02 | 0.95 | 1.09 | 327267 | 820 | 326447 |
| 747.1 | Cardiac congenital anomalies | Congenital Anomalies | 0.97 | 0.84 | 1.10 | 325565 | 211 | 325354 |
| 531.2 | Gastric ulcer | Digestive | 1.01 | 0.98 | 1.04 | 324777 | 4228 | 320549 |
| 964.1 | Anticoagulants causing adverse effects | Injuries & Poisonings | 1.03 | 0.91 | 1.17 | 300470 | 259 | 300211 |
| 990 | Effects radiation NOS | Injuries & Poisonings | 1.01 | 0.97 | 1.04 | 325633 | 3244 | 322389 |
| 870.1 | Open wound or laceration of eye or eyelid | Injuries & Poisonings | 0.97 | 0.86 | 1.09 | 320012 | 281 | 319731 |
| 871 | Open wounds of extremities | Injuries & Poisonings | 0.99 | 0.96 | 1.02 | 323873 | 4142 | 319731 |
| 594.2 | Calculus of lower urinary tract | Genitourinary | 0.98 | 0.92 | 1.05 | 320761 | 830 | 319931 |
| 610 | Benign mammary dysplasias | Genitourinary | 1.03 | 0.90 | 1.18 | 321081 | 208 | 320873 |
| 41.1 | Staphylococcus infections | Infectious Diseases | 1.01 | 0.97 | 1.04 | 315832 | 3200 | 312632 |
| 577.3 | Cyst and pseudocyst of pancreas | Digestive | 0.97 | 0.88 | 1.08 | 326295 | 375 | 325920 |
| 290.11 | Alzheimer's disease | Mental Disorders | 1.02 | 0.93 | 1.13 | 321872 | 402 | 321470 |
| 368.2 | Diplopia and disorders of binocular vision | Sense Organs | 1.02 | 0.95 | 1.09 | 325635 | 753 | 324882 |
| 791 | Gangrene | Symptoms | 0.98 | 0.90 | 1.06 | 328240 | 579 | 327661 |

| | | | | | | | | |
|---|---|---|---|---|---|---|---|---|
| 496.1 | Emphysema | Respiratory | 0.99 | 0.94 | 1.04 | 295918 | 1726 | 294192 |
| 585.2 | Renal failure NOS | Genitourinary | 1.01 | 0.98 | 1.03 | 322824 | 8745 | 314079 |
| 380 | Disorders of external ear | Sense Organs | 0.99 | 0.94 | 1.04 | 328227 | 1475 | 326752 |
| 54 | Herpes simplex | Infectious Diseases | 1.03 | 0.90 | 1.18 | 322425 | 219 | 322206 |
| 574.11 | Cholelithiasis with acute cholecystitis | Digestive | 0.99 | 0.94 | 1.04 | 311836 | 1513 | 310323 |
| 532 | Dysphagia | Digestive | 0.99 | 0.97 | 1.02 | 294356 | 6553 | 287803 |
| 622.2 | Mucous polyp of cervix | Genitourinary | 0.99 | 0.96 | 1.03 | 318829 | 3522 | 315307 |
| 747.12 | Valvular heart disease/ heart chambers | Congenital Anomalies | 0.97 | 0.85 | 1.10 | 325592 | 238 | 325354 |
| 227.3 | Benign neoplasm of pituitary gland and craniopharyngeal duct (pouch) | Neoplasms | 0.97 | 0.87 | 1.09 | 326970 | 328 | 326642 |
| 596.5 | Functional disorders of bladder | Genitourinary | 1.01 | 0.96 | 1.06 | 315318 | 1682 | 313636 |
| 317.1 | Alcoholism | Mental Disorders | 0.99 | 0.97 | 1.02 | 306366 | 8697 | 297669 |
| 187.1 | Malignant neoplasm of unspecified male genital organ | Neoplasms | 0.99 | 0.97 | 1.02 | 327699 | 8481 | 319218 |
| 614.1 | Pelvic peritoneal adhesions, female (postoperative) (postinfection) | Genitourinary | 0.99 | 0.95 | 1.03 | 321370 | 2571 | 318799 |
| 512.9 | Other dyspnea | Respiratory | 1.02 | 0.95 | 1.08 | 315211 | 943 | 314268 |
| 288 | Diseases of white blood cells | Hematopoietic | 0.98 | 0.91 | 1.06 | 320928 | 610 | 320318 |
| 705.8 | Hyperhidrosis | Dermatologic | 1.02 | 0.94 | 1.10 | 318896 | 602 | 318294 |
| 526 | Diseases of the jaws | Digestive | 1.00 | 0.99 | 1.02 | 327645 | 15987 | 311658 |
| 212 | Benign neoplasm of respiratory and intrathoracic organs | Neoplasms | 1.02 | 0.92 | 1.14 | 328240 | 355 | 327885 |
| 210 | Benign neoplasm of lip, oral cavity, and pharynx | Neoplasms | 0.99 | 0.93 | 1.05 | 327034 | 993 | 326041 |
| 537 | Other disorders of stomach and duodenum | Digestive | 1.01 | 0.97 | 1.04 | 299994 | 3483 | 296511 |
| 740.11 | Osteoarthrosis, localized, primary | Musculoskeletal | 1.00 | 0.97 | 1.02 | 315552 | 9227 | 306325 |
| 447.1 | Stricture of artery | Circulatory System | 0.99 | 0.92 | 1.05 | 320246 | 886 | 319360 |
| 526.9 | Jaw disease NOS | Digestive | 1.00 | 0.99 | 1.02 | 327620 | 15962 | 311658 |
| 229 | Benign neoplasm of unspecified sites | Neoplasms | 1.00 | 0.99 | 1.01 | 328240 | 46165 | 282075 |
| 604.1 | Redundant prepuce and phimosis/BXO | Genitourinary | 1.01 | 0.97 | 1.05 | 310716 | 2810 | 307906 |
| 202.2 | Non-Hodgkins lymphoma | Neoplasms | 0.99 | 0.96 | 1.03 | 327055 | 3532 | 323523 |
| 938 | Dermatitis due to solar radiation | Injuries & Poisonings | 0.99 | 0.95 | 1.03 | 324444 | 2538 | 321906 |
| 433.5 | Cerebral aneurysm | Circulatory System | 1.02 | 0.93 | 1.13 | 318505 | 398 | 318107 |
| 379.3 | Aphakia and other disorders of lens | Sense Organs | 1.00 | 0.98 | 1.01 | 308920 | 20728 | 288192 |
| 674 | Other complications of the puerperium NEC | Pregnancy Complications | 0.97 | 0.87 | 1.10 | 328015 | 280 | 327735 |
| 706 | Diseases of sebaceous glands | Dermatologic | 0.98 | 0.91 | 1.06 | 318889 | 595 | 318294 |
| 250.6 | Polyneuropathy in diabetes | Endocrine/Metabolic | 0.98 | 0.88 | 1.08 | 307871 | 381 | 307490 |
| 613.9 | Breast disorder NOS | Genitourinary | 1.01 | 0.98 | 1.03 | 327937 | 5519 | 322418 |
| 714 | Rheumatoid arthritis and other inflammatory polyarthropathies | Musculoskeletal | 1.00 | 0.98 | 1.03 | 325905 | 8652 | 317253 |
| 440.2 | Atherosclerosis of the extremities | Circulatory System | 1.02 | 0.94 | 1.11 | 319912 | 552 | 319360 |
| 741 | Symptoms and disorders of the joints | Musculoskeletal | 1.00 | 0.99 | 1.02 | 327531 | 16889 | 310642 |
| 628 | Ovarian cyst | Genitourinary | 0.99 | 0.97 | 1.02 | 301051 | 4820 | 296231 |
| 345.12 | Partial epilepsy | Neurological | 0.98 | 0.87 | 1.09 | 286450 | 298 | 286152 |

| | | | | | | | | |
|---|---|---|---|---|---|---|---|---|
| 728.7 | Fasciitis | Musculoskeletal | 0.97 | 0.85 | 1.11 | 305167 | 213 | 304954 |
| 594 | Urinary calculus | Genitourinary | 1.01 | 0.98 | 1.03 | 326897 | 6966 | 319931 |
| 289 | Other diseases of blood and blood-forming organs | Hematopoietic | 0.99 | 0.97 | 1.02 | 325300 | 4982 | 320318 |
| 350.3 | Lack of coordination | Neurological | 1.02 | 0.92 | 1.13 | 325804 | 360 | 325444 |
| 735.2 | Acquired toe deformities | Musculoskeletal | 1.01 | 0.97 | 1.05 | 318913 | 2226 | 316687 |
| 717 | Polymyalgia Rheumatica | Musculoskeletal | 0.99 | 0.93 | 1.05 | 328240 | 1147 | 327093 |
| 300.12 | Agorophobia, social phobia, and panic disorder | Mental Disorders | 0.98 | 0.92 | 1.06 | 283642 | 744 | 282898 |
| 465.2 | Acute pharyngitis | Respiratory | 0.99 | 0.92 | 1.05 | 326053 | 874 | 325179 |
| 285.22 | Anemia in neoplastic disease | Hematopoietic | 0.98 | 0.89 | 1.08 | 309337 | 437 | 308900 |
| 939 | Atopic/contact dermatitis due to other or unspecified | Dermatologic | 1.01 | 0.97 | 1.05 | 324060 | 2154 | 321906 |
| 366.2 | Senile cataract | Sense Organs | 1.00 | 0.97 | 1.02 | 315842 | 8458 | 307384 |
| 368 | Visual disturbances | Sense Organs | 1.01 | 0.95 | 1.07 | 325963 | 1081 | 324882 |
| 496 | Chronic airway obstruction | Respiratory | 1.00 | 0.97 | 1.02 | 301827 | 7635 | 294192 |
| 698 | Pruritus and related conditions | Dermatologic | 1.01 | 0.95 | 1.09 | 328240 | 799 | 327441 |
| 420.21 | Acute pericarditis | Circulatory System | 0.97 | 0.86 | 1.11 | 325211 | 228 | 324983 |
| 191.11 | Cancer of brain | Neoplasms | 0.98 | 0.90 | 1.07 | 326945 | 498 | 326447 |
| 635.2 | Antepartum hemorrhage, abruptio placentae, and placenta previa | Pregnancy Complications | 0.99 | 0.92 | 1.05 | 320901 | 936 | 319965 |
| 555.21 | Ulcerative colitis (chronic) | Digestive | 0.98 | 0.91 | 1.07 | 258967 | 571 | 258396 |
| 386.9 | Dizziness and giddiness (Light-headedness and vertigo) | Sense Organs | 1.01 | 0.98 | 1.03 | 326388 | 4739 | 321649 |
| 318 | Tobacco use disorder | Mental Disorders | 1.00 | 0.99 | 1.02 | 318172 | 20503 | 297669 |
| 174.1 | Breast cancer [female] | Neoplasms | 1.01 | 0.97 | 1.05 | 306711 | 2287 | 304424 |
| 798.1 | Chronic fatigue syndrome | Symptoms | 0.98 | 0.91 | 1.07 | 325335 | 606 | 324729 |
| 149 | Cancer of larynx, pharynx, nasal cavities | Neoplasms | 1.01 | 0.95 | 1.08 | 326986 | 945 | 326041 |
| 568 | Other disorders of peritoneum | Digestive | 0.99 | 0.96 | 1.03 | 250464 | 3804 | 246660 |
| 276.12 | Hyposmolality and/or hyponatremia | Endocrine/Metabolic | 1.01 | 0.96 | 1.06 | 322496 | 1852 | 320644 |
| 681.1 | Cellulitis and abscess of fingers/toes | Dermatologic | 1.02 | 0.94 | 1.10 | 316989 | 582 | 316407 |
| 585.3 | Chronic renal failure [CKD] | Genitourinary | 1.01 | 0.97 | 1.05 | 316664 | 2585 | 314079 |
| 280.2 | Iron deficiency anemia secondary to blood loss (chronic) | Hematopoietic | 1.02 | 0.93 | 1.11 | 309376 | 476 | 308900 |
| 426.21 | First degree AV block | Circulatory System | 0.99 | 0.93 | 1.05 | 300526 | 1005 | 299521 |
| 751.22 | Other specified congenital anomalies of kidney | Congenital Anomalies | 0.98 | 0.88 | 1.09 | 326353 | 350 | 326003 |
| 715 | Other inflammatory spondylopathies | Musculoskeletal | 1.02 | 0.91 | 1.15 | 317524 | 271 | 317253 |
| 420.2 | Pericarditis | Circulatory System | 0.99 | 0.93 | 1.05 | 326070 | 1087 | 324983 |
| 360.2 | Progressive myopia | Sense Organs | 1.02 | 0.90 | 1.16 | 317032 | 250 | 316782 |
| 592.1 | Cystitis | Genitourinary | 1.01 | 0.97 | 1.05 | 299089 | 2054 | 297035 |
| 306 | Other mental disorder | Mental Disorders | 1.00 | 0.99 | 1.01 | 312303 | 29405 | 282898 |
| 244.4 | Hypothyroidism NOS | Endocrine/Metabolic | 1.00 | 0.99 | 1.02 | 324759 | 14430 | 310329 |
| 618.6 | Vaginal enterocele, congenital or acquired | Genitourinary | 0.99 | 0.91 | 1.06 | 316909 | 667 | 316242 |
| 540 | Appendiceal conditions | Digestive | 1.01 | 0.97 | 1.04 | 328240 | 3861 | 324379 |

| | | | | | | | | |
|---|---|---|---|---|---|---|---|---|
| 512.1 | Wheezing | Respiratory | 0.98 | 0.86 | 1.11 | 314499 | 231 | 314268 |
| 602 | Other disorders of prostate | Genitourinary | 1.01 | 0.96 | 1.06 | 308765 | 1372 | 307393 |
| 580.2 | Nephrotic syndrome without mention of glomerulonephritis | Genitourinary | 0.98 | 0.90 | 1.07 | 314575 | 496 | 314079 |
| 285.2 | Anemia of chronic disease | Hematopoietic | 0.98 | 0.87 | 1.10 | 309194 | 294 | 308900 |
| 470 | Septal Deviations/Turbinate Hypertrophy | Respiratory | 0.99 | 0.97 | 1.02 | 313771 | 4821 | 308950 |
| 281 | Other deficiency anemia | Hematopoietic | 1.00 | 0.98 | 1.03 | 317687 | 8787 | 308900 |
| 454.1 | Varicose veins of lower extremity | Circulatory System | 1.00 | 0.98 | 1.02 | 299275 | 11562 | 287713 |
| 384.4 | Perforation of tympanic membrane | Sense Organs | 0.99 | 0.93 | 1.05 | 325167 | 1024 | 324143 |
| 967 | Adverse effects of sedatives or other central nervous system depressants and anesthetics | Injuries & Poisonings | 0.99 | 0.91 | 1.07 | 300790 | 579 | 300211 |
| 301 | Personality disorders | Mental Disorders | 1.02 | 0.93 | 1.12 | 283330 | 432 | 282898 |
| 743.9 | Osteopenia or other disorder of bone and cartilage | Musculoskeletal | 0.99 | 0.96 | 1.03 | 328188 | 3989 | 324199 |
| 634 | Miscarriage; stillbirth | Pregnancy Complications | 0.99 | 0.96 | 1.03 | 324534 | 4569 | 319965 |
| 41.2 | Streptococcus infection | Infectious Diseases | 1.01 | 0.96 | 1.06 | 314280 | 1648 | 312632 |
| 835 | Internal derangement of knee | Injuries & Poisonings | 1.01 | 0.98 | 1.04 | 326012 | 4239 | 321773 |
| 150 | Cancer of esophagus | Neoplasms | 1.01 | 0.94 | 1.09 | 313058 | 727 | 312331 |
| 965.2 | Antirheumatics causing adverse effects in therapeutic use | Injuries & Poisonings | 0.99 | 0.90 | 1.08 | 300710 | 499 | 300211 |
| 185 | Cancer of prostate | Neoplasms | 1.00 | 0.97 | 1.02 | 315856 | 8463 | 307393 |
| 300.13 | Phobia | Mental Disorders | 0.99 | 0.90 | 1.07 | 283412 | 514 | 282898 |
| 594.8 | Renal colic | Genitourinary | 1.01 | 0.97 | 1.05 | 322062 | 2131 | 319931 |
| 464 | Acute sinusitis | Respiratory | 1.02 | 0.89 | 1.17 | 325387 | 208 | 325179 |
| 218 | Benign neoplasm of uterus | Neoplasms | 0.98 | 0.89 | 1.09 | 308168 | 388 | 307780 |
| 732 | Osteochondropathies | Musculoskeletal | 1.02 | 0.90 | 1.17 | 315651 | 219 | 315432 |
| 907 | Injuries to the nervous system | Injuries & Poisonings | 0.99 | 0.95 | 1.04 | 328240 | 1845 | 326395 |
| 579 | Other symptoms involving abdomen and pelvis | Digestive | 1.01 | 0.95 | 1.07 | 304580 | 1186 | 303394 |
| 870 | Open wounds of head; neck; and trunk | Injuries & Poisonings | 1.01 | 0.94 | 1.10 | 320327 | 596 | 319731 |
| 531.3 | Duodenal ulcer | Digestive | 0.99 | 0.96 | 1.03 | 323644 | 3095 | 320549 |
| 627.2 | Symptomatic menopause | Genitourinary | 1.02 | 0.89 | 1.17 | 296434 | 203 | 296231 |
| 857 | Mechanical complication of unspecified genitourinary device, implant, and graft | Injuries & Poisonings | 0.99 | 0.94 | 1.05 | 315248 | 1284 | 313964 |
| 618.1 | Prolapse of vaginal walls | Genitourinary | 1.00 | 0.98 | 1.03 | 323726 | 7484 | 316242 |
| 561 | Symptoms involving digestive system | Digestive | 1.00 | 0.98 | 1.01 | 272941 | 14545 | 258396 |
| 530.3 | Stricture and stenosis of esophagus | Digestive | 1.01 | 0.96 | 1.05 | 289757 | 1954 | 287803 |
| 536.8 | Dyspepsia and other specified disorders of function of stomach | Digestive | 1.02 | 0.90 | 1.16 | 296758 | 247 | 296511 |
| 433.2 | Occlusion of cerebral arteries | Circulatory System | 0.99 | 0.96 | 1.03 | 321087 | 2980 | 318107 |
| 349 | Other and unspecified disorders of the nervous system | Neurological | 1.00 | 0.99 | 1.01 | 326855 | 40703 | 286152 |
| 211 | Benign neoplasm of other parts of digestive system | Neoplasms | 1.00 | 0.98 | 1.03 | 319683 | 5375 | 314308 |
| 771.1 | Swelling of limb | Symptoms | 0.99 | 0.92 | 1.06 | 327356 | 842 | 326514 |
| 716.9 | Arthropathy NOS | Musculoskeletal | 1.00 | 0.99 | 1.01 | 327719 | 53823 | 273896 |
| 365 | Glaucoma | Sense Organs | 1.00 | 0.98 | 1.03 | 321340 | 4558 | 316782 |

| | | | | | | | | |
|---|---|---|---|---|---|---|---|---|
| 389 | Hearing loss | Sense Organs | 0.99 | 0.96 | 1.03 | 321364 | 3170 | 318194 |
| 357 | Inflammatory and toxic neuropathy | Neurological | 0.99 | 0.94 | 1.04 | 327375 | 1423 | 325952 |
| 687 | Symptoms affecting skin | Dermatologic | 0.98 | 0.86 | 1.12 | 323032 | 218 | 322814 |
| 694.2 | Other dyschromia | Dermatologic | 1.01 | 0.94 | 1.08 | 322549 | 800 | 321749 |
| 350.1 | Abnormal involuntary movements | Neurological | 1.01 | 0.95 | 1.08 | 326371 | 927 | 325444 |
| 586 | Other disorders of the kidney and ureters | Genitourinary | 0.99 | 0.96 | 1.03 | 317490 | 3411 | 314079 |
| 280.1 | Iron deficiency anemias, unspecified or not due to blood loss | Hematopoietic | 1.00 | 0.98 | 1.03 | 316402 | 7502 | 308900 |
| 726.1 | Enthesopathy | Musculoskeletal | 1.00 | 0.98 | 1.03 | 311657 | 6703 | 304954 |
| 479 | Other upper respiratory disease | Respiratory | 1.00 | 0.98 | 1.01 | 324241 | 15291 | 308950 |
| 724.9 | Other unspecified back disorders | Musculoskeletal | 1.00 | 0.98 | 1.01 | 328029 | 17085 | 310944 |
| 597 | Other disorders of urethra and urinary tract | Genitourinary | 0.99 | 0.93 | 1.06 | 314510 | 874 | 313636 |
| 535.6 | Duodenitis | Digestive | 1.00 | 0.98 | 1.03 | 304405 | 7894 | 296511 |
| 411.1 | Unstable angina (intermediate coronary syndrome) | Circulatory System | 1.00 | 0.97 | 1.02 | 301011 | 5322 | 295689 |
| 284 | Aplastic anemia | Hematopoietic | 1.00 | 0.99 | 1.02 | 321659 | 12759 | 308900 |
| 293.1 | Swelling, mass, or lump in head and neck [Space-occupying lesion, intracranial NOS] | Mental Disorders | 1.01 | 0.95 | 1.08 | 326044 | 925 | 325119 |
| 701.2 | Scar conditions and fibrosis of skin | Dermatologic | 0.99 | 0.96 | 1.03 | 325471 | 2407 | 323064 |
| 500.2 | Pneumoconiosis | Respiratory | 1.01 | 0.95 | 1.07 | 317485 | 1001 | 316484 |
| 727.4 | Ganglion and cyst of synovium, tendon, and bursa | Musculoskeletal | 0.99 | 0.96 | 1.03 | 307936 | 2982 | 304954 |
| 300 | Anxiety, phobic and dissociative disorders | Mental Disorders | 0.99 | 0.89 | 1.09 | 283261 | 363 | 282898 |
| 386 | Vertiginous syndromes and other disorders of vestibular system | Sense Organs | 0.99 | 0.95 | 1.04 | 323761 | 2112 | 321649 |
| 722.6 | Degeneration of intervertebral disc | Musculoskeletal | 1.01 | 0.97 | 1.04 | 313866 | 2922 | 310944 |
| 433.21 | Cerebral artery occlusion, with cerebral infarction | Circulatory System | 0.99 | 0.94 | 1.04 | 319641 | 1534 | 318107 |
| 840 | Sprains and strains | Injuries & Poisonings | 0.99 | 0.90 | 1.08 | 328201 | 481 | 327720 |
| 529.1 | Glossitis | Digestive | 1.02 | 0.91 | 1.14 | 322838 | 304 | 322534 |
| 626.2 | Dysmenorrhea | Genitourinary | 1.01 | 0.96 | 1.06 | 297910 | 1679 | 296231 |
| 367.8 | Hypermetropia | Sense Organs | 0.98 | 0.87 | 1.11 | 325995 | 241 | 325754 |
| 378.2 | Nystagmus and other irregular eye movements | Sense Organs | 0.98 | 0.86 | 1.12 | 288407 | 215 | 288192 |
| 426.4 | Anomalous atrioventricular excitation | Circulatory System | 1.02 | 0.90 | 1.15 | 299761 | 240 | 299521 |
| 288.11 | Neutropenia | Hematopoietic | 1.00 | 0.96 | 1.03 | 323644 | 3326 | 320318 |
| 575.1 | Cholangitis | Digestive | 0.99 | 0.91 | 1.08 | 310820 | 497 | 310323 |
| 8.52 | Intestinal infection due to C. difficile | Infectious Diseases | 0.99 | 0.92 | 1.07 | 319727 | 648 | 319079 |
| 572 | Ascites (non malignant) | Digestive | 1.01 | 0.96 | 1.06 | 319925 | 1594 | 318331 |
| 726.2 | Synoviopathy | Musculoskeletal | 0.99 | 0.89 | 1.10 | 305285 | 331 | 304954 |
| 634.3 | Ectopic pregnancy | Pregnancy Complications | 1.01 | 0.91 | 1.13 | 320307 | 342 | 319965 |
| 585.1 | Acute renal failure | Genitourinary | 1.00 | 0.98 | 1.03 | 318744 | 4665 | 314079 |
| 289.4 | Lymphadenitis | Hematopoietic | 1.00 | 0.97 | 1.04 | 323033 | 2715 | 320318 |
| 516.1 | Hemoptysis | Respiratory | 1.01 | 0.96 | 1.05 | 328154 | 2103 | 326051 |
| 836 | Traumatic arthropathy | Injuries & Poisonings | 1.02 | 0.90 | 1.15 | 322016 | 243 | 321773 |

| | | | | | | | | |
|---|---|---|---|---|---|---|---|---|
| 289.3 | Personal history of diseases of blood and blood-forming organs | Hematopoietic | 1.01 | 0.91 | 1.14 | 320615 | 297 | 320318 |
| 804 | Fracture of hand or wrist | Injuries & Poisonings | 1.01 | 0.94 | 1.09 | 324232 | 675 | 323557 |
| 197 | Chemotherapy | Neoplasms | 1.00 | 0.99 | 1.02 | 251774 | 22462 | 229312 |
| 504 | Other alveolar and parietoalveolar pneumonopathy | Respiratory | 1.01 | 0.91 | 1.13 | 316793 | 309 | 316484 |
| 509.8 | Dependence on respirator [Ventilator] or supplemental oxygen | Respiratory | 1.01 | 0.92 | 1.12 | 316885 | 401 | 316484 |
| 574.2 | Calculus of bile duct | Digestive | 1.00 | 0.96 | 1.03 | 313011 | 2688 | 310323 |
| 216 | Benign neoplasm of skin | Neoplasms | 1.00 | 0.98 | 1.02 | 327614 | 7865 | 319749 |
| 285.1 | Acute posthemorrhagic anemia | Hematopoietic | 1.01 | 0.90 | 1.14 | 309167 | 267 | 308900 |
| 556.1 | Ulceration of intestine | Digestive | 0.99 | 0.92 | 1.07 | 259087 | 691 | 258396 |
| 612.2 | Hypertrophy of breast (Gynecomastia) | Genitourinary | 0.99 | 0.93 | 1.06 | 321732 | 859 | 320873 |
| 502 | Postinflammatory pulmonary fibrosis | Respiratory | 0.99 | 0.93 | 1.06 | 317367 | 883 | 316484 |
| 743.11 | Osteoporosis NOS | Musculoskeletal | 1.00 | 0.97 | 1.04 | 327235 | 3036 | 324199 |
| 495.2 | Asthma with exacerbation | Respiratory | 1.01 | 0.90 | 1.14 | 294453 | 261 | 294192 |
| 289.8 | Polycythemia vera, secondary | Hematopoietic | 1.01 | 0.90 | 1.14 | 320039 | 291 | 319748 |
| 614.4 | Inflammatory diseases of uterus, except cervix | Genitourinary | 0.99 | 0.88 | 1.10 | 319107 | 308 | 318799 |
| 735.3 | Hallux valgus (Bunion) | Musculoskeletal | 1.00 | 0.98 | 1.03 | 323592 | 6905 | 316687 |
| 803 | Fracture of upper limb | Injuries & Poisonings | 1.01 | 0.92 | 1.11 | 323990 | 433 | 323557 |
| 805 | Fracture of vertebral column without mention of spinal cord injury | Injuries & Poisonings | 0.99 | 0.89 | 1.10 | 323890 | 333 | 323557 |
| 512.8 | Cough | Respiratory | 1.00 | 0.97 | 1.04 | 317232 | 2964 | 314268 |
| 599.4 | Urinary incontinence | Genitourinary | 1.00 | 0.98 | 1.02 | 229423 | 8996 | 220427 |
| 427.8 | Sinoatrial node dysfunction (Bradycardia) | Circulatory System | 1.01 | 0.92 | 1.11 | 299940 | 419 | 299521 |
| 303.3 | Psychogenic disorder | Mental Disorders | 1.01 | 0.94 | 1.08 | 283739 | 841 | 282898 |
| 530.7 | Gastroesophageal laceration-hemorrhage syndrome | Digestive | 1.01 | 0.91 | 1.13 | 288136 | 333 | 287803 |
| 195.1 | Malignant neoplasm, other | Neoplasms | 1.00 | 0.99 | 1.01 | 320138 | 90826 | 229312 |
| 702.1 | Actinic keratosis | Dermatologic | 1.00 | 0.97 | 1.04 | 325264 | 2664 | 322600 |
| 714.1 | Rheumatoid arthritis | Musculoskeletal | 1.00 | 0.96 | 1.05 | 319560 | 2307 | 317253 |
| 701 | Other hypertrophic and atrophic conditions of skin | Dermatologic | 1.00 | 0.95 | 1.04 | 324891 | 1827 | 323064 |
| 522 | Diseases of pulp and periapical tissues | Digestive | 1.01 | 0.93 | 1.09 | 312297 | 639 | 311658 |
| 250.7 | Diabetic retinopathy | Endocrine/Metabolic | 0.99 | 0.94 | 1.05 | 317232 | 1380 | 315852 |
| 610.4 | Benign neoplasm of breast | Genitourinary | 1.01 | 0.96 | 1.06 | 322359 | 1486 | 320873 |
| 619.5 | Noninflammatory disorders of vulva and perineum | Genitourinary | 1.01 | 0.95 | 1.06 | 279927 | 1379 | 278548 |
| 345.3 | Convulsions | Neurological | 1.00 | 0.96 | 1.05 | 288463 | 2311 | 286152 |
| 323.8 | Encephalitis, non-infectious | Neurological | 0.99 | 0.92 | 1.07 | 327771 | 633 | 327138 |
| 227.2 | Benign neoplasm of parathyroid gland | Neoplasms | 0.99 | 0.90 | 1.09 | 327032 | 390 | 326642 |
| 260.6 | Anorexia | Endocrine/Metabolic | 1.01 | 0.94 | 1.08 | 326636 | 850 | 325786 |
| 204 | Leukemia | Neoplasms | 0.99 | 0.90 | 1.09 | 323984 | 461 | 323523 |
| 619.2 | Disorders of uterus, NEC | Genitourinary | 1.00 | 0.97 | 1.04 | 281939 | 3391 | 278548 |
| 272.11 | Hypercholesterolemia | Endocrine/Metabolic | 1.00 | 0.99 | 1.01 | 325499 | 34002 | 291497 |

| | | | | | | | | | |
|---|---|---|---|---|---|---|---|---|---|
| 578.9 | Hemorrhage of gastrointestinal tract | Digestive | 1.00 | 0.97 | 1.02 | 308849 | 5455 | 303394 |
| 523 | Gingival and periodontal diseases | Digestive | 0.99 | 0.91 | 1.08 | 312187 | 529 | 311658 |
| 965.1 | Opiates and related narcotics causing adverse effects in therapeutic use | Injuries & Poisonings | 0.99 | 0.94 | 1.05 | 301372 | 1161 | 300211 |
| 377 | Disorders of optic nerve and visual pathways | Sense Organs | 1.01 | 0.92 | 1.11 | 288628 | 436 | 288192 |
| 721.8 | Other allied disorders of spine | Musculoskeletal | 1.00 | 0.96 | 1.03 | 314171 | 3227 | 310944 |
| 627.3 | Postmenopausal atrophic vaginitis | Genitourinary | 1.01 | 0.95 | 1.07 | 297359 | 1128 | 296231 |
| 737 | Curvature of spine | Musculoskeletal | 1.01 | 0.94 | 1.08 | 317430 | 743 | 316687 |
| 287.31 | Primary thrombocytopenia | Hematopoietic | 1.01 | 0.92 | 1.11 | 325925 | 428 | 325497 |
| 619.4 | Noninflammatory disorders of vagina | Genitourinary | 1.00 | 0.96 | 1.05 | 280328 | 1780 | 278548 |
| 256.4 | Polycystic ovaries | Endocrine/Metabolic | 0.99 | 0.87 | 1.13 | 324764 | 219 | 324545 |
| 246 | Other disorders of thyroid | Endocrine/Metabolic | 1.00 | 0.98 | 1.01 | 327738 | 17409 | 310329 |
| 687.4 | Disturbance of skin sensation | Dermatologic | 1.00 | 0.96 | 1.03 | 325793 | 2979 | 322814 |
| 509.2 | Respiratory insufficiency | Respiratory | 1.00 | 0.96 | 1.05 | 318272 | 1788 | 316484 |
| 721 | Spondylosis and allied disorders | Musculoskeletal | 1.01 | 0.94 | 1.07 | 311869 | 925 | 310944 |
| 618.2 | Uterine/Uterovaginal prolapse | Genitourinary | 1.00 | 0.98 | 1.03 | 321713 | 5471 | 316242 |
| 250.23 | Type 2 diabetes with ophthalmic manifestations | Endocrine/Metabolic | 1.00 | 0.95 | 1.06 | 308834 | 1344 | 307490 |
| 521.1 | Dental caries | Digestive | 1.00 | 0.96 | 1.03 | 314912 | 3254 | 311658 |
| 506 | Empyema and pneumothorax | Respiratory | 0.99 | 0.94 | 1.05 | 317630 | 1146 | 316484 |
| 165 | Cancer within the respiratory system | Neoplasms | 1.00 | 0.96 | 1.03 | 328223 | 2954 | 325269 |
| 523.31 | Acute periodontitis | Digestive | 1.01 | 0.94 | 1.08 | 312371 | 713 | 311658 |
| 687.1 | Rash and other nonspecific skin eruption | Dermatologic | 1.00 | 0.96 | 1.05 | 325032 | 2218 | 322814 |
| 599.2 | Retention of urine | Genitourinary | 1.00 | 0.97 | 1.02 | 227284 | 6857 | 220427 |
| 540.11 | Acute appendicitis | Digestive | 1.00 | 0.97 | 1.04 | 327037 | 2658 | 324379 |
| 947 | Urticaria | Injuries & Poisonings | 0.99 | 0.90 | 1.10 | 322293 | 387 | 321906 |
| 701.5 | Abnormal granulation tissue | Dermatologic | 0.99 | 0.91 | 1.09 | 323543 | 479 | 323064 |
| 696.41 | Psoriasis vulgaris | Dermatologic | 1.00 | 0.96 | 1.05 | 315655 | 1723 | 313932 |
| 738 | Other acquired musculoskeletal deformity | Musculoskeletal | 0.99 | 0.90 | 1.09 | 317104 | 417 | 316687 |
| 362.4 | Retinal vascular changes and abnomalities | Sense Organs | 0.99 | 0.93 | 1.06 | 316733 | 881 | 315852 |
| 340 | Migraine | Neurological | 1.00 | 0.96 | 1.03 | 320723 | 2773 | 317950 |
| 198.4 | Secondary malignant neoplasm of liver | Neoplasms | 1.00 | 0.96 | 1.04 | 232009 | 2697 | 229312 |
| 159 | Malignant neoplasm of other and ill-defined sites within the digestive organs and peritoneum | Neoplasms | 1.00 | 0.98 | 1.03 | 317620 | 5289 | 312331 |
| 458.2 | Iatrogenic hypotension | Circulatory System | 1.01 | 0.89 | 1.15 | 194417 | 238 | 194179 |
| 379 | Other disorders of eye | Sense Organs | 1.00 | 0.99 | 1.01 | 328190 | 39998 | 288192 |
| 290 | Delirium dementia and amnestic and other cognitive disorders | Mental Disorders | 1.01 | 0.92 | 1.10 | 321950 | 480 | 321470 |
| 575.9 | Nonspecific abnormal findings on radiological and other examination of biliary tract | Digestive | 1.01 | 0.91 | 1.12 | 310690 | 367 | 310323 |
| 760 | Back pain | Symptoms | 1.00 | 0.97 | 1.02 | 328240 | 5923 | 322317 |
| 567 | Peritonitis and retroperitoneal infections | Digestive | 1.00 | 0.94 | 1.07 | 247550 | 890 | 246660 |
| 782.3 | Edema | Symptoms | 1.00 | 0.96 | 1.05 | 328067 | 1715 | 326352 |

| | | | | | | | | | |
|---|---|---|---|---|---|---|---|---|---|
| 994.2 | Sepsis | Injuries & Poisonings | 1.00 | 0.96 | 1.03 | 328240 | 2885 | 325355 |
| 530.5 | Disorders of esophageal motility | Digestive | 1.01 | 0.93 | 1.09 | 288452 | 649 | 287803 |
| 596 | Other disorders of bladder | Genitourinary | 1.00 | 0.98 | 1.02 | 321385 | 7749 | 313636 |
| 214.1 | Lipoma of skin and subcutaneous tissue | Neoplasms | 1.00 | 0.97 | 1.03 | 325432 | 4756 | 320676 |
| 337 | Disorders of the autonomic nervous system | Neurological | 1.01 | 0.88 | 1.16 | 286359 | 207 | 286152 |
| 204.4 | Multiple myeloma | Neoplasms | 0.99 | 0.92 | 1.08 | 324097 | 574 | 323523 |
| 472 | Chronic pharyngitis and nasopharyngitis | Respiratory | 1.00 | 0.93 | 1.06 | 309897 | 947 | 308950 |
| 613.7 | Other signs and symptoms in breast | Genitourinary | 1.00 | 0.94 | 1.08 | 323169 | 751 | 322418 |
| 586.2 | Cyst of kidney, acquired | Genitourinary | 1.00 | 0.94 | 1.05 | 315372 | 1293 | 314079 |
| 274.1 | Gout | Endocrine/Metabolic | 1.00 | 0.95 | 1.05 | 327930 | 1668 | 326262 |
| 957 | Injury to other and unspecified nerves | Injuries & Poisonings | 0.99 | 0.88 | 1.12 | 328157 | 275 | 327882 |
| 530.11 | GERD | Digestive | 1.00 | 0.98 | 1.02 | 302292 | 14489 | 287803 |
| 418 | Nonspecific chest pain | Circulatory System | 1.00 | 0.99 | 1.01 | 326032 | 30212 | 295820 |
| 647.1 | Infections of genitourinary tract during pregnancy | Pregnancy Complications | 0.99 | 0.88 | 1.12 | 328128 | 284 | 327844 |
| 367.1 | Myopia | Sense Organs | 1.00 | 0.94 | 1.05 | 327025 | 1271 | 325754 |
| 701.4 | Keloid scar | Dermatologic | 1.01 | 0.89 | 1.15 | 323292 | 228 | 323064 |
| 279.7 | Other immunological findings | Endocrine/Metabolic | 0.99 | 0.88 | 1.12 | 327978 | 264 | 327714 |
| 38 | Septicemia | Infectious Diseases | 1.00 | 0.97 | 1.04 | 315677 | 3045 | 312632 |
| 522.5 | Periapical abscess | Digestive | 1.00 | 0.94 | 1.05 | 312904 | 1246 | 311658 |
| 245.21 | Chronic lymphocytic thyroiditis | Endocrine/Metabolic | 0.99 | 0.87 | 1.13 | 310548 | 219 | 310329 |
| 803.2 | Fracture of radius and ulna | Injuries & Poisonings | 1.00 | 0.93 | 1.06 | 324441 | 884 | 323557 |
| 740.9 | Osteoarthrosis NOS | Musculoskeletal | 1.00 | 0.97 | 1.03 | 310757 | 4432 | 306325 |
| 244.1 | Secondary hypothyroidism | Endocrine/Metabolic | 1.00 | 0.94 | 1.06 | 311484 | 1155 | 310329 |
| 174.11 | Malignant neoplasm of female breast | Neoplasms | 1.00 | 0.98 | 1.02 | 316838 | 12414 | 304424 |
| 531.1 | Hemorrhage from gastrointestinal ulcer | Digestive | 1.00 | 0.93 | 1.08 | 321204 | 655 | 320549 |
| 686.4 | Pyogenic granuloma | Dermatologic | 0.99 | 0.90 | 1.10 | 316787 | 380 | 316407 |
| 38.1 | Gram negative septicemia | Infectious Diseases | 1.00 | 0.94 | 1.07 | 313487 | 855 | 312632 |
| 480.1 | Bacterial pneumonia | Respiratory | 1.00 | 0.94 | 1.07 | 318628 | 939 | 317689 |
| 788 | Syncope and collapse | Symptoms | 1.00 | 0.98 | 1.02 | 328240 | 9372 | 318868 |
| 626.12 | Excessive or frequent menstruation | Genitourinary | 1.00 | 0.98 | 1.02 | 306272 | 10041 | 296231 |
| 783 | Fever of unknown origin | Symptoms | 1.00 | 0.97 | 1.03 | 328240 | 4111 | 324129 |
| 540.1 | Appendicitis | Digestive | 1.00 | 0.94 | 1.08 | 325174 | 795 | 324379 |
| 340.1 | Migrain with aura | Neurological | 0.99 | 0.87 | 1.13 | 318167 | 217 | 317950 |
| 759 | Other and unspecified congenital anomalies | Congenital Anomalies | 1.00 | 0.93 | 1.08 | 327249 | 679 | 326570 |
| 565 | Anal and rectal conditions | Digestive | 1.00 | 0.98 | 1.02 | 255321 | 8661 | 246660 |
| 351 | Other peripheral nerve disorders | Neurological | 1.00 | 0.98 | 1.02 | 325903 | 12892 | 313011 |
| 707 | Chronic ulcer of skin | Dermatologic | 1.00 | 0.94 | 1.05 | 327959 | 1260 | 326699 |
| 198.3 | Secondary malignant neoplasm of digestive systems | Neoplasms | 1.00 | 0.95 | 1.05 | 230877 | 1565 | 229312 |

| | | | | | | | | | |
|---|---|---|---|---|---|---|---|---|---|
| 291.8 | Alteration of consciousness | Mental Disorders | 1.00 | 0.91 | 1.10 | 321904 | 434 | 321470 |
| 512 | Other symptoms of respiratory system | Respiratory | 1.00 | 0.97 | 1.03 | 319772 | 5504 | 314268 |
| 742.9 | Other derangement of joint | Musculoskeletal | 1.00 | 0.90 | 1.10 | 311042 | 400 | 310642 |
| 723 | Other disorders of cervical region | Musculoskeletal | 1.00 | 0.90 | 1.10 | 311345 | 401 | 310944 |
| 323 | Encephalitis | Neurological | 1.00 | 0.93 | 1.07 | 327999 | 861 | 327138 |
| 550.4 | Umbilical hernia | Digestive | 1.00 | 0.97 | 1.03 | 283693 | 3904 | 279789 |
| 740.1 | Osteoarthritis; localized | Musculoskeletal | 1.00 | 0.98 | 1.02 | 315797 | 9472 | 306325 |
| 204.12 | Lymphoid leukemia, chronic | Neoplasms | 1.00 | 0.91 | 1.09 | 324030 | 507 | 323523 |
| 184.2 | Cancer of other female genital organs | Neoplasms | 1.00 | 0.89 | 1.11 | 305876 | 323 | 305553 |
| 509.1 | Respiratory failure | Respiratory | 1.00 | 0.96 | 1.04 | 318564 | 2080 | 316484 |
| 214 | Lipoma | Neoplasms | 1.00 | 0.96 | 1.05 | 322495 | 1819 | 320676 |
| 365.11 | Primary open angle glaucoma | Sense Organs | 1.00 | 0.94 | 1.06 | 317838 | 1056 | 316782 |
| 443.1 | Raynaud's syndrome | Circulatory System | 1.00 | 0.95 | 1.06 | 320525 | 1165 | 319360 |
| 427.3 | Other specified cardiac dysrhythmias | Circulatory System | 1.00 | 0.97 | 1.04 | 302836 | 3315 | 299521 |
| 619 | Noninflammatory female genital disorders | Genitourinary | 1.00 | 0.99 | 1.01 | 328106 | 49558 | 278548 |
| 53 | Herpes zoster | Infectious Diseases | 1.00 | 0.90 | 1.12 | 322547 | 341 | 322206 |
| 381.9 | Otorrhea | Sense Organs | 1.00 | 0.89 | 1.13 | 324420 | 277 | 324143 |
| 202.24 | Large cell lymphoma | Neoplasms | 1.00 | 0.92 | 1.09 | 324112 | 589 | 323523 |
| 364.5 | Corneal dystrophy | Sense Organs | 1.00 | 0.88 | 1.15 | 316998 | 216 | 316782 |
| 441.1 | Acute vascular insufficiency of intestine | Circulatory System | 1.00 | 0.89 | 1.13 | 319631 | 271 | 319360 |
| 803.1 | Fracture of humerus | Injuries & Poisonings | 1.00 | 0.90 | 1.12 | 323850 | 293 | 323557 |
| 365.2 | Primary angle-closure glaucoma | Sense Organs | 1.00 | 0.93 | 1.07 | 317491 | 709 | 316782 |
| 296.22 | Major depressive disorder | Mental Disorders | 1.00 | 0.91 | 1.11 | 283282 | 384 | 282898 |
| 720 | Spinal stenosis | Musculoskeletal | 1.00 | 0.93 | 1.07 | 311636 | 692 | 310944 |
| 531.4 | Peptic ulcer, site unspecified | Digestive | 1.00 | 0.91 | 1.10 | 320998 | 449 | 320549 |
| 529 | Diseases and other conditions of the tongue | Digestive | 1.00 | 0.94 | 1.06 | 323485 | 951 | 322534 |
| 427 | Cardiac dysrhythmias | Circulatory System | 1.00 | 0.89 | 1.11 | 299844 | 323 | 299521 |
| 752.11 | Spina bifida | Congenital Anomalies | 1.00 | 0.87 | 1.14 | 327886 | 211 | 327675 |
| 430.2 | Intracerebral hemorrhage | Circulatory System | 1.00 | 0.93 | 1.08 | 318830 | 723 | 318107 |
| 368.9 | Subjective visual disturbances | Sense Organs | 1.00 | 0.93 | 1.08 | 325515 | 633 | 324882 |
| 443.7 | Peripheral angiopathy in diseases classified elsewhere | Circulatory System | 1.00 | 0.91 | 1.10 | 319775 | 415 | 319360 |
| 749 | Congenital anomalies of face and neck | Congenital Anomalies | 1.00 | 0.89 | 1.13 | 328137 | 271 | 327866 |
| 707.1 | Decubitus ulcer | Dermatologic | 1.00 | 0.90 | 1.11 | 327025 | 326 | 326699 |
| 275.5 | Disorders of calcium/phosphorus metabolism | Endocrine/Metabolic | 1.00 | 0.94 | 1.06 | 327144 | 1109 | 326035 |
| 348.9 | Other conditions of brain, NOS | Neurological | 1.00 | 0.91 | 1.10 | 286559 | 407 | 286152 |
| 536 | Disorders of function of stomach | Digestive | 1.00 | 0.89 | 1.13 | 296768 | 257 | 296511 |
| 426.23 | Second degree AV block | Circulatory System | 1.00 | 0.91 | 1.10 | 299982 | 461 | 299521 |
| 571.6 | Primary biliary cirrhosis | Digestive | 1.00 | 0.89 | 1.13 | 318589 | 258 | 318331 |

| | | | | | | | | | |
|---|---|---|---|---|---|---|---|---|---|
| 213 | Benign neoplasm of bone and articular cartilage | Neoplasms | 1.00 | 0.89 | 1.11 | 277528 | 322 | 277206 |
| 535.8 | Other specified gastritis | Digestive | 1.00 | 0.98 | 1.02 | 304926 | 8415 | 296511 |
| 807 | Fracture of ribs | Injuries & Poisonings | 1.00 | 0.88 | 1.14 | 323780 | 223 | 323557 |
| 433.8 | Late effects of cerebrovascular disease | Circulatory System | 1.00 | 0.95 | 1.06 | 319384 | 1277 | 318107 |
| 444 | Arterial embolism and thrombosis | Circulatory System | 1.00 | 0.91 | 1.10 | 319794 | 434 | 319360 |
| 735.23 | Hallux rigidus | Musculoskeletal | 1.00 | 0.95 | 1.05 | 318243 | 1556 | 316687 |
| 614.53 | Cyst or abscess of Bartholin's gland | Genitourinary | 1.00 | 0.93 | 1.07 | 319602 | 803 | 318799 |
| 252.1 | Hyperparathyroidism | Endocrine/Metabolic | 1.00 | 0.93 | 1.07 | 325359 | 814 | 324545 |
| 624.9 | stress incontinence, female | Genitourinary | 1.00 | 0.97 | 1.03 | 324769 | 6100 | 318669 |
| 550 | Abdominal hernia | Digestive | 1.00 | 0.99 | 1.01 | 328240 | 48451 | 279789 |
| 332 | Parkinson's disease | Neurological | 1.00 | 0.94 | 1.06 | 287334 | 1182 | 286152 |
| 519.9 | Symptoms involving respiratory system and other chest symptoms | Respiratory | 1.00 | 0.90 | 1.12 | 265755 | 308 | 265447 |
| 430.1 | Subarachnoid hemorrhage | Circulatory System | 1.00 | 0.93 | 1.07 | 318931 | 824 | 318107 |
| 535 | Gastritis and duodenitis | Digestive | 1.00 | 0.99 | 1.02 | 314259 | 17748 | 296511 |
| 455 | Hemorrhoids | Circulatory System | 1.00 | 0.99 | 1.01 | 312410 | 24697 | 287713 |
| 339 | Other headache syndromes | Neurological | 1.00 | 0.98 | 1.02 | 325888 | 7938 | 317950 |
| 520.2 | Disturbances in tooth eruption | Digestive | 1.00 | 0.96 | 1.04 | 314095 | 2437 | 311658 |
| 384 | Other disorders of tympanic membrane | Sense Organs | 1.00 | 0.91 | 1.10 | 324573 | 430 | 324143 |
| 575.7 | Other disorders of gallbladder | Digestive | 1.00 | 0.95 | 1.05 | 311719 | 1396 | 310323 |
| 709.7 | Unspecified diffuse connective tissue disease | Dermatologic | 1.00 | 0.99 | 1.01 | 324635 | 82669 | 241966 |
| 524.3 | Anomalies of tooth position/malocclusion | Digestive | 1.00 | 0.89 | 1.12 | 311953 | 295 | 311658 |
| 433 | Cerebrovascular disease | Circulatory System | 1.00 | 0.98 | 1.02 | 326753 | 8646 | 318107 |
| 338.1 | Acute pain | Neurological | 1.00 | 0.91 | 1.10 | 327846 | 420 | 327426 |
| 618.5 | Prolapse of vaginal vault after hysterectomy | Genitourinary | 1.00 | 0.92 | 1.09 | 316743 | 501 | 316242 |
| 733 | Other disorders of bone and cartilage | Musculoskeletal | 1.00 | 0.92 | 1.08 | 316001 | 569 | 315432 |
| 527.2 | Sialoadenitis | Digestive | 1.00 | 0.90 | 1.11 | 322908 | 374 | 322534 |
| 369.5 | Conjunctivitis, infectious | Sense Organs | 1.00 | 0.88 | 1.14 | 318612 | 224 | 318388 |
| 174 | Breast cancer | Neoplasms | 1.00 | 0.89 | 1.12 | 304712 | 288 | 304424 |
| 253.2 | Pituitary hypofunction | Endocrine/Metabolic | 1.00 | 0.89 | 1.12 | 324826 | 281 | 324545 |
| 495 | Asthma | Respiratory | 1.00 | 0.99 | 1.01 | 320971 | 26779 | 294192 |
| 603.1 | Hydrocele | Genitourinary | 1.00 | 0.95 | 1.05 | 309308 | 1402 | 307906 |
| 145.2 | Cancer of tongue | Neoplasms | 1.00 | 0.90 | 1.11 | 326366 | 325 | 326041 |
| 277.4 | Disorders of bilirubin excretion | Endocrine/Metabolic | 1.00 | 0.90 | 1.11 | 283300 | 373 | 282927 |
| 198.5 | Secondary malignancy of brain/spine | Neoplasms | 1.00 | 0.93 | 1.07 | 230113 | 801 | 229312 |
| 591 | Urinary tract infection | Genitourinary | 1.00 | 0.98 | 1.02 | 309711 | 12676 | 297035 |
| 501 | Pneumonitis due to inhalation of food or vomitus | Respiratory | 1.00 | 0.92 | 1.08 | 317076 | 592 | 316484 |
| 401.22 | Hypertensive chronic kidney disease | Circulatory System | 1.00 | 0.95 | 1.05 | 250343 | 1595 | 248748 |
| 292.2 | Mild cognitive impairment | Mental Disorders | 1.00 | 0.87 | 1.15 | 321672 | 202 | 321470 |

Supplementary Table 15. Phenome-wide association study (PheWAS) results for the beta-blocker (BB) genetic risk score after excluding potentially pleiotropic single-nucleotide polymorphisms identified by their secondary associations in PhenoScanner.

| Phecode | Trait | Category | OR | Low 95% CI | Upp 95% CI | Total sample size | Cases | Controls |
|---|---|---|---|---|---|---|---|---|
| 401 | Hypertension | Circulatory System | 0.97 | 0.96 | 0.97 | 328239 | 79491 | 248748 |
| 401.1 | Essential hypertension | Circulatory System | 0.97 | 0.96 | 0.97 | 327983 | 79235 | 248748 |
| 459.9 | Circulatory disease NEC | Circulatory System | 0.98 | 0.97 | 0.99 | 327928 | 133749 | 194179 |
| 916 | Contusion | Injuries & Poisonings | 0.89 | 0.85 | 0.94 | 328240 | 1478 | 326762 |
| 395.1 | Nonrheumatic mitral valve disorders | Circulatory System | 0.94 | 0.91 | 0.97 | 324497 | 2969 | 321528 |
| 394.2 | Mitral valve disease | Circulatory System | 0.94 | 0.91 | 0.98 | 324603 | 3075 | 321528 |
| 427 | Cardiac dysrhythmias | Circulatory System | 0.85 | 0.77 | 0.94 | 299844 | 323 | 299521 |
| 250 | Diabetes mellitus | Endocrine/Metabolic | 1.02 | 1.01 | 1.04 | 327730 | 20240 | 307490 |
| 550.3 | Femoral hernia | Digestive | 0.89 | 0.83 | 0.96 | 280442 | 653 | 279789 |
| 289.3 | Personal history of diseases of blood and blood-forming organs | Hematopoietic | 0.85 | 0.76 | 0.95 | 320615 | 297 | 320318 |
| 528 | Diseases of the oral soft tissues, excluding lesions specific for gingiva and tongue | Digestive | 0.94 | 0.90 | 0.98 | 324593 | 2059 | 322534 |
| 250.2 | Type 2 diabetes | Endocrine/Metabolic | 1.02 | 1.01 | 1.04 | 326603 | 19113 | 307490 |
| 371.1 | Uveitis, noninfectious or NOS | Sense Organs | 1.14 | 1.04 | 1.25 | 318886 | 498 | 318388 |
| 427.2 | Atrial fibrillation and flutter | Circulatory System | 0.98 | 0.96 | 0.99 | 314573 | 15052 | 299521 |
| 433.3 | Cerebral ischemia | Circulatory System | 0.92 | 0.86 | 0.98 | 319041 | 934 | 318107 |
| 586.2 | Cyst of kidney, acquired | Genitourinary | 0.93 | 0.88 | 0.98 | 315372 | 1293 | 314079 |
| 571.6 | Primary biliary cirrhosis | Digestive | 1.19 | 1.04 | 1.35 | 318589 | 258 | 318331 |
| 613.9 | Breast disorder NOS | Genitourinary | 1.04 | 1.01 | 1.06 | 327937 | 5519 | 322418 |
| 260.6 | Anorexia | Endocrine/Metabolic | 0.92 | 0.86 | 0.98 | 326636 | 850 | 325786 |
| 634.3 | Ectopic pregnancy | Pregnancy Complications | 0.88 | 0.79 | 0.97 | 320307 | 342 | 319965 |
| 702.1 | Actinic keratosis | Dermatologic | 1.05 | 1.01 | 1.09 | 325264 | 2664 | 322600 |
| 382 | Otalgia | Sense Organs | 0.87 | 0.79 | 0.97 | 324458 | 315 | 324143 |
| 704 | Diseases of hair and hair follicles | Dermatologic | 1.04 | 1.01 | 1.06 | 326692 | 5263 | 321429 |
| 738.4 | Acquired spondylolisthesis | Musculoskeletal | 1.18 | 1.03 | 1.34 | 316942 | 255 | 316687 |
| 149.4 | Cancer of larynx | Neoplasms | 0.87 | 0.77 | 0.97 | 326307 | 266 | 326041 |
| 623 | Hypertrophy of female genital organs | Genitourinary | 1.07 | 1.01 | 1.14 | 316579 | 1272 | 315307 |
| 613.1 | Inflammatory disease of breast | Genitourinary | 1.10 | 1.02 | 1.18 | 323156 | 738 | 322418 |
| 444.1 | Arterial embolism and thrombosis of lower extremity artery | Circulatory System | 0.91 | 0.84 | 0.98 | 319930 | 570 | 319360 |
| 649.1 | Diabetes or abnormal glucose tolerance complicating pregnancy | Pregnancy Complications | 1.20 | 1.03 | 1.38 | 328127 | 208 | 327919 |
| 411.8 | Other chronic ischemic heart disease, unspecified | Circulatory System | 0.99 | 0.97 | 1.00 | 326969 | 31280 | 295689 |
| 411 | Ischemic Heart Disease | Circulatory System | 0.99 | 0.97 | 1.00 | 327032 | 31343 | 295689 |
| 586 | Other disorders of the kidney and ureters | Genitourinary | 0.96 | 0.93 | 0.99 | 317490 | 3411 | 314079 |
| 8 | Intestinal infection | Infectious Diseases | 0.98 | 0.96 | 1.00 | 328240 | 9161 | 319079 |
| 276.12 | Hyposmolality and/or hyponatremia | Endocrine/Metabolic | 0.95 | 0.91 | 0.99 | 322496 | 1852 | 320644 |
| 411.4 | Coronary atherosclerosis | Circulatory System | 0.99 | 0.97 | 1.00 | 327242 | 31553 | 295689 |

| | | | | | | | | | |
|---|---|---|---|---|---|---|---|---|---|
| 225.1 | Benign neoplasm of brain, cranial nerves, meninges | Neoplasms | 0.92 | 0.86 | 0.99 | 327267 | 820 | 326447 |
| 53 | Herpes zoster | Infectious Diseases | 0.89 | 0.80 | 0.98 | 322547 | 341 | 322206 |
| 703 | Diseases of nail, NOS | Dermatologic | 1.13 | 1.02 | 1.26 | 321795 | 366 | 321429 |
| 189.21 | Malignant neoplasm of bladder | Neoplasms | 0.95 | 0.92 | 0.99 | 326150 | 2188 | 323962 |
| 447.1 | Stricture of artery | Circulatory System | 0.93 | 0.87 | 0.99 | 320246 | 886 | 319360 |
| 427.9 | Palpitations | Circulatory System | 0.97 | 0.94 | 1.00 | 303480 | 3959 | 299521 |
| 977 | Personal history of allergy to medicinal agents | Injuries & Poisonings | 1.16 | 1.01 | 1.33 | 300449 | 238 | 300211 |
| 396 | Abnormal heart sounds | Circulatory System | 1.07 | 1.01 | 1.14 | 322603 | 1075 | 321528 |
| 446.9 | Arteritis NOS | Circulatory System | 0.88 | 0.78 | 0.99 | 319605 | 245 | 319360 |
| 809 | Fracture of unspecified bones | Injuries & Poisonings | 0.90 | 0.82 | 0.99 | 323967 | 410 | 323557 |
| 159 | Malignant neoplasm of other and ill-defined sites within the digestive organs and peritoneum | Neoplasms | 0.97 | 0.95 | 1.00 | 317620 | 5289 | 312331 |
| 440.2 | Atherosclerosis of the extremities | Circulatory System | 0.92 | 0.84 | 0.99 | 319912 | 552 | 319360 |
| 348.8 | Encephalopathy, not elsewhere classified | Neurological | 1.17 | 1.01 | 1.35 | 286356 | 204 | 286152 |
| 555.2 | Ulcerative colitis | Digestive | 0.96 | 0.93 | 1.00 | 261550 | 3154 | 258396 |
| 747.13 | Congenital anomalies of great vessels | Congenital Anomalies | 0.95 | 0.91 | 1.00 | 327177 | 1823 | 325354 |
| 204.4 | Multiple myeloma | Neoplasms | 0.92 | 0.85 | 1.00 | 324097 | 574 | 323523 |
| 427.4 | Cardiac arrest and ventricular fibrillation | Circulatory System | 0.90 | 0.81 | 1.00 | 299869 | 348 | 299521 |
| 747.12 | Valvular heart disease/ heart chambers | Congenital Anomalies | 0.88 | 0.78 | 1.00 | 325592 | 238 | 325354 |
| 840 | Sprains and strains | Injuries & Poisonings | 1.10 | 1.00 | 1.21 | 328201 | 481 | 327720 |
| 380 | Disorders of external ear | Sense Organs | 0.95 | 0.90 | 1.00 | 328227 | 1475 | 326752 |
| 523 | Gingival and periodontal diseases | Digestive | 0.92 | 0.85 | 1.00 | 312187 | 529 | 311658 |
| 441.1 | Acute vascular insufficiency of intestine | Circulatory System | 1.13 | 1.00 | 1.28 | 319631 | 271 | 319360 |
| 262 | Mineral deficiency NEC | Endocrine/Metabolic | 1.14 | 1.00 | 1.30 | 326027 | 241 | 325786 |
| 448 | Disease of capillaries | Circulatory System | 0.98 | 0.96 | 1.00 | 326841 | 7481 | 319360 |
| 151 | Cancer of stomach | Neoplasms | 0.92 | 0.85 | 1.00 | 312905 | 574 | 312331 |
| 524.3 | Anomalies of tooth position/malocclusion | Digestive | 0.90 | 0.80 | 1.00 | 311953 | 295 | 311658 |
| 158 | Neoplasm of unspecified nature of digestive system | Neoplasms | 1.06 | 1.00 | 1.13 | 313429 | 1098 | 312331 |
| 427.41 | Ventricular fibrillation and flutter | Circulatory System | 0.91 | 0.82 | 1.00 | 299866 | 345 | 299521 |
| 756 | Other congenital musculoskeletal anomalies | Congenital Anomalies | 1.07 | 1.00 | 1.15 | 328123 | 812 | 327311 |
| 642.1 | Preeclampsia and eclampsia | Pregnancy Complications | 0.91 | 0.82 | 1.00 | 327385 | 335 | 327050 |
| 567 | Peritonitis and retroperitoneal infections | Digestive | 0.94 | 0.88 | 1.00 | 247550 | 890 | 246660 |
| 471 | Nasal polyps | Respiratory | 1.03 | 1.00 | 1.07 | 312342 | 3392 | 308950 |
| 519.8 | Other diseases of respiratory system, NEC | Respiratory | 1.02 | 1.00 | 1.04 | 274529 | 9082 | 265447 |
| 706.2 | Sebaceous cyst | Dermatologic | 1.02 | 1.00 | 1.04 | 327401 | 9107 | 318294 |
| 755.1 | Congenital deformities of feet | Congenital Anomalies | 1.14 | 0.99 | 1.31 | 327531 | 220 | 327311 |
| 290.2 | Delirium due to conditions classified elsewhere | Mental Disorders | 0.93 | 0.87 | 1.00 | 322151 | 681 | 321470 |
| 367.9 | Blindness and low vision | Sense Organs | 1.07 | 1.00 | 1.15 | 326510 | 756 | 325754 |
| 317.11 | Alcoholic liver damage | Mental Disorders | 1.07 | 1.00 | 1.14 | 298534 | 865 | 297669 |

| | | | | | | | | |
|---|---|---|---|---|---|---|---|---|
| 594.1 | Calculus of kidney | Genitourinary | 0.97 | 0.94 | 1.00 | 323265 | 3334 | 319931 |
| 300.13 | Phobia | Mental Disorders | 0.92 | 0.85 | 1.00 | 283412 | 514 | 282898 |
| 458.2 | Iatrogenic hypotension | Circulatory System | 0.89 | 0.79 | 1.01 | 194417 | 238 | 194179 |
| 426.3 | Bundle branch block | Circulatory System | 0.90 | 0.81 | 1.01 | 299812 | 291 | 299521 |
| 740.2 | Osteoarthrosis, generalized | Musculoskeletal | 0.93 | 0.86 | 1.01 | 306914 | 589 | 306325 |
| 627.1 | Postmenopausal bleeding | Genitourinary | 1.02 | 1.00 | 1.04 | 305697 | 9466 | 296231 |
| 276.5 | Hypovolemia | Endocrine/Metabolic | 0.97 | 0.93 | 1.00 | 323539 | 2895 | 320644 |
| 202.21 | Nodular lymphoma | Neoplasms | 0.91 | 0.83 | 1.01 | 323913 | 390 | 323523 |
| 364 | Corneal opacity and other disorders of cornea | Sense Organs | 1.14 | 0.99 | 1.30 | 317009 | 227 | 316782 |
| 585.2 | Renal failure NOS | Genitourinary | 0.98 | 0.96 | 1.00 | 322824 | 8745 | 314079 |
| 577.1 | Acute pancreatitis | Digestive | 0.95 | 0.91 | 1.00 | 327437 | 1517 | 325920 |
| 857 | Mechanical complication of unspecified genitourinary device, implant, and graft | Injuries & Poisonings | 0.95 | 0.90 | 1.00 | 315248 | 1284 | 313964 |
| 426.32 | Left bundle branch block | Circulatory System | 0.96 | 0.92 | 1.00 | 301302 | 1781 | 299521 |
| 783 | Fever of unknown origin | Symptoms | 1.03 | 1.00 | 1.06 | 328240 | 4111 | 324129 |
| 277 | Other disorders of metabolism | Endocrine/Metabolic | 0.99 | 0.98 | 1.00 | 328230 | 45303 | 282927 |
| 585.3 | Chronic renal failure [CKD] | Genitourinary | 0.97 | 0.93 | 1.00 | 316664 | 2585 | 314079 |
| 291.8 | Alteration of consciousness | Mental Disorders | 1.09 | 0.99 | 1.20 | 321904 | 434 | 321470 |
| 191 | Malignant and unknown neoplasms of brain and nervous system | Neoplasms | 0.89 | 0.78 | 1.01 | 326662 | 215 | 326447 |
| 193 | Thyroid cancer | Neoplasms | 0.91 | 0.83 | 1.01 | 327009 | 367 | 326642 |
| 189.4 | Malignant neoplasm of other urinary organs | Neoplasms | 0.97 | 0.94 | 1.00 | 327185 | 3223 | 323962 |
| 208 | Benign neoplasm of colon | Neoplasms | 0.99 | 0.97 | 1.00 | 325437 | 20827 | 304610 |
| 561 | Symptoms involving digestive system | Digestive | 1.02 | 1.00 | 1.03 | 272941 | 14545 | 258396 |
| 369.5 | Conjunctivitis, infectious | Sense Organs | 0.89 | 0.79 | 1.01 | 318612 | 224 | 318388 |
| 210 | Benign neoplasm of lip, oral cavity, and pharynx | Neoplasms | 1.06 | 0.99 | 1.13 | 327034 | 993 | 326041 |
| 575.1 | Cholangitis | Digestive | 1.08 | 0.99 | 1.19 | 310820 | 497 | 310323 |
| 695.9 | Unspecified erythematous condition | Dermatologic | 1.09 | 0.99 | 1.19 | 322226 | 477 | 321749 |
| 389.2 | Conductive hearing loss | Sense Organs | 1.09 | 0.99 | 1.20 | 318629 | 435 | 318194 |
| 872 | Traumatic amputation | Injuries & Poisonings | 1.08 | 0.99 | 1.19 | 320213 | 482 | 319731 |
| 415.21 | Primary pulmonary hypertension | Circulatory System | 0.93 | 0.85 | 1.01 | 322013 | 466 | 321547 |
| 722.9 | Other and unspecified disc disorder | Musculoskeletal | 1.03 | 0.99 | 1.06 | 314894 | 3950 | 310944 |
| 8.6 | Viral Enteritis | Infectious Diseases | 0.95 | 0.89 | 1.01 | 319924 | 845 | 319079 |
| 585.31 | Renal dialysis | Genitourinary | 0.93 | 0.86 | 1.01 | 314603 | 524 | 314079 |
| 564.1 | Irritable Bowel Syndrome | Digestive | 1.02 | 1.00 | 1.05 | 264057 | 5661 | 258396 |
| 687.4 | Disturbance of skin sensation | Dermatologic | 0.97 | 0.94 | 1.01 | 325793 | 2979 | 322814 |
| 695.42 | Systemic lupus erythematosus | Dermatologic | 0.92 | 0.83 | 1.02 | 320784 | 363 | 320421 |
| 477 | Epistaxis or throat hemorrhage | Respiratory | 0.97 | 0.93 | 1.01 | 311469 | 2519 | 308950 |
| 475 | Chronic sinusitis | Respiratory | 1.03 | 0.99 | 1.07 | 311580 | 2630 | 308950 |
| 198.2 | Secondary malignancy of respiratory organs | Neoplasms | 1.04 | 0.99 | 1.08 | 231589 | 2277 | 229312 |

| | | | | | | | | |
|---|---|---|---|---|---|---|---|---|
| 201 | Hodgkin's disease | Neoplasms | 1.11 | 0.98 | 1.25 | 323794 | 271 | 323523 |
| 337 | Disorders of the autonomic nervous system | Neurological | 0.90 | 0.79 | 1.02 | 286359 | 207 | 286152 |
| 728.71 | Contracture of palmar fascia [Dupuytren's disease] | Musculoskeletal | 0.97 | 0.93 | 1.01 | 307403 | 2449 | 304954 |
| 572 | Ascites (non malignant) | Digestive | 1.04 | 0.99 | 1.10 | 319925 | 1594 | 318331 |
| 427.3 | Other specified cardiac dysrhythmias | Circulatory System | 0.97 | 0.94 | 1.01 | 302836 | 3315 | 299521 |
| 295.1 | Schizophrenia | Mental Disorders | 1.07 | 0.98 | 1.16 | 283509 | 611 | 282898 |
| 737 | Curvature of spine | Musculoskeletal | 1.06 | 0.99 | 1.14 | 317430 | 743 | 316687 |
| 411.2 | Myocardial infarction | Circulatory System | 0.99 | 0.97 | 1.00 | 307658 | 11969 | 295689 |
| 180.3 | Cervical intraepithelial neoplasia [CIN] [Cervical dysplasia] | Neoplasms | 0.97 | 0.93 | 1.01 | 299736 | 2153 | 297583 |
| 727.4 | Ganglion and cyst of synovium, tendon, and bursa | Musculoskeletal | 0.97 | 0.94 | 1.01 | 307936 | 2982 | 304954 |
| 473.3 | Paralysis/spasm of vocal cords or larynx | Respiratory | 1.10 | 0.97 | 1.25 | 309217 | 267 | 308950 |
| 338.2 | Chronic pain | Neurological | 1.08 | 0.98 | 1.20 | 327830 | 404 | 327426 |
| 344 | Other paralytic syndromes | Neurological | 0.94 | 0.88 | 1.02 | 286841 | 689 | 286152 |
| 781 | Symptoms involving nervous and musculoskeletal systems | Symptoms | 0.99 | 0.98 | 1.00 | 328238 | 22180 | 306058 |
| 626.13 | Irregular menstrual cycle | Genitourinary | 1.04 | 0.99 | 1.08 | 298189 | 1958 | 296231 |
| 443.9 | Peripheral vascular disease, unspecified | Circulatory System | 0.97 | 0.93 | 1.01 | 321969 | 2609 | 319360 |
| 360.2 | Progressive myopia | Sense Organs | 0.91 | 0.81 | 1.03 | 317032 | 250 | 316782 |
| 695.3 | Rosacea | Dermatologic | 0.92 | 0.83 | 1.02 | 322068 | 319 | 321749 |
| 275.5 | Disorders of calcium/phosphorus metabolism | Endocrine/Metabolic | 0.96 | 0.90 | 1.01 | 327144 | 1109 | 326035 |
| 559 | Ileostomy status | Digestive | 0.96 | 0.92 | 1.01 | 260069 | 1673 | 258396 |
| 520.2 | Disturbances in tooth eruption | Digestive | 1.03 | 0.99 | 1.07 | 314095 | 2437 | 311658 |
| 681 | Superficial cellulitis and abscess | Dermatologic | 0.95 | 0.88 | 1.02 | 317135 | 728 | 316407 |
| 681.5 | Cellulitis and abscess of leg, except foot | Dermatologic | 1.02 | 0.99 | 1.05 | 322184 | 5777 | 316407 |
| 804 | Fracture of hand or wrist | Injuries & Poisonings | 1.06 | 0.98 | 1.15 | 324232 | 675 | 323557 |
| 345.3 | Convulsions | Neurological | 1.03 | 0.99 | 1.08 | 288463 | 2311 | 286152 |
| 681.6 | Cellulitis and abscess of foot, toe | Dermatologic | 1.02 | 0.99 | 1.05 | 322141 | 5734 | 316407 |
| 348.9 | Other conditions of brain, NOS | Neurological | 0.93 | 0.85 | 1.02 | 286559 | 407 | 286152 |
| 433.8 | Late effects of cerebrovascular disease | Circulatory System | 0.96 | 0.91 | 1.01 | 319384 | 1277 | 318107 |
| 747 | Cardiac and circulatory congenital anomalies | Congenital Anomalies | 0.96 | 0.91 | 1.01 | 326576 | 1222 | 325354 |
| 250.11 | Type 1 diabetes with ketoacidosis | Endocrine/Metabolic | 0.91 | 0.81 | 1.03 | 307745 | 255 | 307490 |
| 850 | Hemorrhage or hematoma complicating a procedure | Injuries & Poisonings | 0.98 | 0.95 | 1.01 | 319328 | 5364 | 313964 |
| 297.2 | Suicide or self-inflicted injury | Mental Disorders | 1.03 | 0.99 | 1.07 | 285655 | 2757 | 282898 |
| 306 | Other mental disorder | Mental Disorders | 0.99 | 0.98 | 1.00 | 312303 | 29405 | 282898 |
| 374 | Other disorders of eyelids | Sense Organs | 0.97 | 0.94 | 1.01 | 321593 | 3205 | 318388 |
| 689 | Disorder of skin and subcutaneous tissue NOS | Dermatologic | 1.01 | 1.00 | 1.02 | 328240 | 42194 | 286046 |
| 8.5 | Bacterial enteritis | Infectious Diseases | 0.97 | 0.93 | 1.01 | 321188 | 2109 | 319079 |
| 729 | Other disorders of soft tissues | Musculoskeletal | 0.99 | 0.98 | 1.00 | 327478 | 22524 | 304954 |
| 365.2 | Primary angle-closure glaucoma | Sense Organs | 0.95 | 0.88 | 1.02 | 317491 | 709 | 316782 |

| | | | | | | | | | |
|---|---|---|---|---|---|---|---|---|---|
| 727.5 | Rupture of synovium | Musculoskeletal | 1.09 | 0.97 | 1.21 | 305298 | 344 | 304954 |
| 647.1 | Infections of genitourinary tract during pregnancy | Pregnancy Complications | 1.09 | 0.97 | 1.23 | 328128 | 284 | 327844 |
| 414 | Other forms of chronic heart disease | Circulatory System | 0.97 | 0.92 | 1.01 | 297457 | 1768 | 295689 |
| 562.1 | Diverticulosis | Digestive | 0.99 | 0.98 | 1.00 | 286281 | 27885 | 258396 |
| 569.2 | Gastrointestinal complications | Digestive | 1.07 | 0.98 | 1.18 | 247132 | 472 | 246660 |
| 364.5 | Corneal dystrophy | Sense Organs | 1.11 | 0.96 | 1.27 | 316998 | 216 | 316782 |
| 362.4 | Retinal vascular changes and abnomalities | Sense Organs | 1.05 | 0.98 | 1.12 | 316733 | 881 | 315852 |
| 276.14 | Hypopotassemia | Endocrine/Metabolic | 0.96 | 0.92 | 1.01 | 322115 | 1471 | 320644 |
| 351 | Other peripheral nerve disorders | Neurological | 0.99 | 0.97 | 1.00 | 325903 | 12892 | 313011 |
| 681.3 | Cellulitis and abscess of arm/hand | Dermatologic | 1.02 | 0.99 | 1.05 | 322177 | 5770 | 316407 |
| 394 | Rheumatic disease of the heart valves | Circulatory System | 0.96 | 0.90 | 1.02 | 322471 | 943 | 321528 |
| 858 | Complication of internal orthopedic device | Injuries & Poisonings | 1.03 | 0.99 | 1.06 | 317147 | 3183 | 313964 |
| 293 | Symptoms involving head and neck | Mental Disorders | 1.03 | 0.99 | 1.08 | 327323 | 2204 | 325119 |
| 386.2 | Peripheral or central vertigo | Sense Organs | 0.93 | 0.85 | 1.03 | 322036 | 387 | 321649 |
| 577.2 | Chronic pancreatitis | Digestive | 1.06 | 0.98 | 1.16 | 326456 | 536 | 325920 |
| 596.1 | Bladder neck obstruction | Genitourinary | 0.97 | 0.93 | 1.01 | 315665 | 2029 | 313636 |
| 411.9 | Other acute and subacute forms of ischemic heart disease | Circulatory System | 0.96 | 0.91 | 1.02 | 296883 | 1194 | 295689 |
| 386.9 | Dizziness and giddiness (Light-headedness and vertigo) | Sense Organs | 0.98 | 0.95 | 1.01 | 326388 | 4739 | 321649 |
| 674 | Other complications of the puerperium NEC | Pregnancy Complications | 1.09 | 0.97 | 1.23 | 328015 | 280 | 327735 |
| 585.1 | Acute renal failure | Genitourinary | 0.98 | 0.95 | 1.01 | 318744 | 4665 | 314079 |
| 574.2 | Calculus of bile duct | Digestive | 1.03 | 0.99 | 1.07 | 313011 | 2688 | 310323 |
| 622.2 | Mucous polyp of cervix | Genitourinary | 1.02 | 0.99 | 1.06 | 318829 | 3522 | 315307 |
| 569 | Other disorders of intestine | Digestive | 0.99 | 0.99 | 1.00 | 326148 | 79488 | 246660 |
| 715 | Other inflammatory spondylopathies | Musculoskeletal | 0.92 | 0.82 | 1.04 | 317524 | 271 | 317253 |
| 70.4 | Chronic hepatitis | Infectious Diseases | 1.09 | 0.96 | 1.22 | 322496 | 290 | 322206 |
| 530.2 | Esophageal bleeding (varices/hemorrhage) | Digestive | 1.03 | 0.99 | 1.09 | 289525 | 1722 | 287803 |
| 427.5 | Arrhythmia (cardiac) NOS | Circulatory System | 1.05 | 0.98 | 1.12 | 300469 | 948 | 299521 |
| 967 | Adverse effects of sedatives or other central nervous system depressants and anesthetics | Injuries & Poisonings | 0.95 | 0.87 | 1.02 | 300790 | 579 | 300211 |
| 564.9 | Personal history of diseases of digestive system | Digestive | 0.99 | 0.97 | 1.00 | 274320 | 15924 | 258396 |
| 599.5 | Frequency of urination and polyuria | Genitourinary | 0.98 | 0.95 | 1.01 | 224574 | 4147 | 220427 |
| 362.2 | Degeneration of macula and posterior pole of retina | Sense Organs | 0.97 | 0.93 | 1.01 | 318055 | 2203 | 315852 |
| 803.2 | Fracture of radius and ulna | Injuries & Poisonings | 0.96 | 0.90 | 1.02 | 324441 | 884 | 323557 |
| 362.29 | Macular degeneration (senile) of retina NOS | Sense Organs | 0.97 | 0.93 | 1.01 | 318052 | 2200 | 315852 |
| 619.5 | Noninflammatory disorders of vulva and perineum | Genitourinary | 0.96 | 0.92 | 1.02 | 279927 | 1379 | 278548 |
| 681.1 | Cellulitis and abscess of fingers/toes | Dermatologic | 0.95 | 0.87 | 1.03 | 316989 | 582 | 316407 |
| 695.7 | Prurigo and Lichen | Dermatologic | 0.95 | 0.89 | 1.02 | 322541 | 792 | 321749 |
| 605 | Erectile dysfunction [ED] | Genitourinary | 0.93 | 0.83 | 1.04 | 308197 | 291 | 307906 |
| 508 | Pulmonary collapse; interstitial and compensatory emphysema | Respiratory | 0.97 | 0.93 | 1.01 | 318570 | 2086 | 316484 |

| | | | | | | | | |
|---|---|---|---|---|---|---|---|---|
| 535 | Gastritis and duodenitis | Digestive | 1.01 | 0.99 | 1.03 | 314259 | 17748 | 296511 |
| 573.7 | Abnormal results of function study of liver | Digestive | 0.98 | 0.95 | 1.01 | 321896 | 3565 | 318331 |
| 733 | Other disorders of bone and cartilage | Musculoskeletal | 1.06 | 0.97 | 1.15 | 316001 | 569 | 315432 |
| 782.3 | Edema | Symptoms | 0.97 | 0.92 | 1.02 | 328067 | 1715 | 326352 |
| 740.1 | Osteoarthritis; localized | Musculoskeletal | 0.99 | 0.97 | 1.01 | 315797 | 9472 | 306325 |
| 550.2 | Diaphragmatic hernia | Digestive | 0.99 | 0.98 | 1.00 | 307453 | 27664 | 279789 |
| 242 | Thyrotoxicosis with or without goiter | Endocrine/Metabolic | 0.97 | 0.92 | 1.02 | 311794 | 1465 | 310329 |
| 593 | Hematuria | Genitourinary | 1.01 | 0.99 | 1.03 | 313795 | 16760 | 297035 |
| 555.21 | Ulcerative colitis (chronic) | Digestive | 1.06 | 0.97 | 1.15 | 258967 | 571 | 258396 |
| 571 | Chronic liver disease and cirrhosis | Digestive | 1.06 | 0.97 | 1.15 | 318871 | 540 | 318331 |
| 369 | Infection of the eye | Sense Organs | 0.92 | 0.82 | 1.04 | 318642 | 254 | 318388 |
| 241.1 | Nontoxic uninodular goiter | Endocrine/Metabolic | 0.95 | 0.87 | 1.03 | 310840 | 511 | 310329 |
| 701.4 | Keloid scar | Dermatologic | 1.09 | 0.95 | 1.25 | 323292 | 228 | 323064 |
| 626.14 | Irregular menstrual bleeding | Genitourinary | 1.02 | 0.99 | 1.05 | 300177 | 3946 | 296231 |
| 420.2 | Pericarditis | Circulatory System | 1.04 | 0.98 | 1.11 | 326070 | 1087 | 324983 |
| 938 | Dermatitis due to solar radiation | Injuries & Poisonings | 1.03 | 0.99 | 1.07 | 324444 | 2538 | 321906 |
| 747.1 | Cardiac congenital anomalies | Congenital Anomalies | 1.10 | 0.95 | 1.26 | 325565 | 211 | 325354 |
| 198 | Secondary malignant neoplasm | Neoplasms | 0.96 | 0.91 | 1.02 | 230431 | 1119 | 229312 |
| 172.3 | Carcinoma in situ of skin | Neoplasms | 0.95 | 0.88 | 1.03 | 314884 | 664 | 314220 |
| 80 | Postoperative infection | Infectious Diseases | 0.98 | 0.95 | 1.01 | 326101 | 4622 | 321479 |
| 960 | Poisoning by antibiotics | Injuries & Poisonings | 0.98 | 0.95 | 1.01 | 303739 | 3528 | 300211 |
| 636.2 | Early onset of delivery | Pregnancy Complications | 1.05 | 0.97 | 1.14 | 320594 | 629 | 319965 |
| 429.2 | Abnormal function study of cardiovascular system | Circulatory System | 0.95 | 0.87 | 1.03 | 322577 | 544 | 322033 |
| 830 | Dislocation | Injuries & Poisonings | 1.03 | 0.98 | 1.07 | 323927 | 2154 | 321773 |
| 202.2 | Non-Hodgkins lymphoma | Neoplasms | 0.98 | 0.95 | 1.01 | 327055 | 3532 | 323523 |
| 507 | Pleurisy; pleural effusion | Respiratory | 0.98 | 0.96 | 1.01 | 323091 | 6607 | 316484 |
| 250.23 | Type 2 diabetes with ophthalmic manifestations | Endocrine/Metabolic | 1.04 | 0.98 | 1.09 | 308834 | 1344 | 307490 |
| 342 | Hemiplegia | Neurological | 0.97 | 0.92 | 1.02 | 287677 | 1525 | 286152 |
| 357 | Inflammatory and toxic neuropathy | Neurological | 1.03 | 0.98 | 1.09 | 327375 | 1423 | 325952 |
| 720 | Spinal stenosis | Musculoskeletal | 1.05 | 0.97 | 1.13 | 311636 | 692 | 310944 |
| 504 | Other alveolar and parietoalveolar pneumonopathy | Respiratory | 1.08 | 0.96 | 1.21 | 316793 | 309 | 316484 |
| 701.2 | Scar conditions and fibrosis of skin | Dermatologic | 1.03 | 0.99 | 1.07 | 325471 | 2407 | 323064 |
| 70.9 | Hepatitis NOS | Infectious Diseases | 1.06 | 0.97 | 1.17 | 322658 | 452 | 322206 |
| 805 | Fracture of vertebral column without mention of spinal cord injury | Injuries & Poisonings | 0.94 | 0.84 | 1.04 | 323890 | 333 | 323557 |
| 965.1 | Opiates and related narcotics causing adverse effects in therapeutic use | Injuries & Poisonings | 0.96 | 0.91 | 1.02 | 301372 | 1161 | 300211 |
| 871 | Open wounds of extremities | Injuries & Poisonings | 1.02 | 0.99 | 1.05 | 323873 | 4142 | 319731 |
| 303.3 | Psychogenic disorder | Mental Disorders | 1.04 | 0.97 | 1.12 | 283739 | 841 | 282898 |
| 395.6 | Heart valve replaced | Circulatory System | 0.97 | 0.92 | 1.02 | 323056 | 1528 | 321528 |

| | | | | | | | | |
|---|---|---|---|---|---|---|---|---|
| 275.1 | Disorders of iron metabolism | Hematopoietic | 0.95 | 0.89 | 1.03 | 326735 | 700 | 326035 |
| 627.2 | Symptomatic menopause | Genitourinary | 0.92 | 0.80 | 1.05 | 296434 | 203 | 296231 |
| 426.4 | Anomalous atrioventricular excitation | Circulatory System | 0.93 | 0.82 | 1.05 | 299761 | 240 | 299521 |
| 333.1 | Essential tremor | Neurological | 1.09 | 0.95 | 1.25 | 286370 | 218 | 286152 |
| 614.1 | Pelvic peritoneal adhesions, female (postoperative) (postinfection) | Genitourinary | 1.03 | 0.99 | 1.07 | 321370 | 2571 | 318799 |
| 785 | Abdominal pain | Symptoms | 1.01 | 1.00 | 1.02 | 328240 | 42311 | 285929 |
| 636.3 | Hemorrhage in early pregnancy | Pregnancy Complications | 0.96 | 0.91 | 1.02 | 321078 | 1113 | 319965 |
| 859 | Complication due to other implant and internal device | Injuries & Poisonings | 1.02 | 0.99 | 1.06 | 317302 | 3338 | 313964 |
| 372 | Disorders of conjunctiva | Sense Organs | 0.96 | 0.91 | 1.02 | 319431 | 1043 | 318388 |
| 611.3 | Lump or mass in breast | Genitourinary | 1.03 | 0.98 | 1.08 | 322461 | 1588 | 320873 |
| 578 | Gastrointestinal hemorrhage | Digestive | 0.93 | 0.83 | 1.04 | 303679 | 285 | 303394 |
| 170.2 | Cancer of connective tissue | Neoplasms | 1.05 | 0.97 | 1.13 | 328122 | 734 | 327388 |
| 394.7 | Disease of tricuspid valve | Circulatory System | 0.96 | 0.91 | 1.02 | 322614 | 1086 | 321528 |
| 380.4 | Impacted cerumen | Sense Organs | 0.94 | 0.85 | 1.04 | 327106 | 354 | 326752 |
| 276.13 | Hyperpotassemia | Endocrine/Metabolic | 1.04 | 0.98 | 1.11 | 321623 | 979 | 320644 |
| 965.2 | Antirheumatics causing adverse effects in therapeutic use | Injuries & Poisonings | 1.06 | 0.97 | 1.16 | 300710 | 499 | 300211 |
| 578.8 | Hemorrhage of rectum and anus | Digestive | 0.99 | 0.97 | 1.01 | 317220 | 13826 | 303394 |
| 389.4 | Tinnitus | Sense Organs | 0.95 | 0.87 | 1.03 | 318735 | 541 | 318194 |
| 250.1 | Type 1 diabetes | Endocrine/Metabolic | 1.02 | 0.99 | 1.06 | 310189 | 2699 | 307490 |
| 681.2 | Cellulitis and abscess of face/neck | Dermatologic | 0.95 | 0.87 | 1.03 | 316936 | 529 | 316407 |
| 430.2 | Intracerebral hemorrhage | Circulatory System | 0.96 | 0.89 | 1.03 | 318830 | 723 | 318107 |
| 530 | Diseases of esophagus | Digestive | 0.96 | 0.90 | 1.03 | 288604 | 801 | 287803 |
| 274.1 | Gout | Endocrine/Metabolic | 0.97 | 0.93 | 1.02 | 327930 | 1668 | 326262 |
| 751.12 | Congenital anomalies of male genital organs | Congenital Anomalies | 0.93 | 0.82 | 1.05 | 326231 | 228 | 326003 |
| 495.2 | Asthma with exacerbation | Respiratory | 1.08 | 0.95 | 1.22 | 294453 | 261 | 294192 |
| 474.1 | Acute tonsillitis | Respiratory | 1.05 | 0.97 | 1.14 | 309515 | 565 | 308950 |
| 416 | Cardiomegaly | Circulatory System | 0.98 | 0.94 | 1.02 | 324157 | 2610 | 321547 |
| 578.9 | Hemorrhage of gastrointestinal tract | Digestive | 1.02 | 0.99 | 1.04 | 308849 | 5455 | 303394 |
| 596.5 | Functional disorders of bladder | Genitourinary | 1.03 | 0.98 | 1.08 | 315318 | 1682 | 313636 |
| 941 | Adverse reaction to serum or vaccine | Injuries & Poisonings | 1.08 | 0.95 | 1.24 | 322128 | 222 | 321906 |
| 289.5 | Diseases of spleen | Hematopoietic | 1.05 | 0.96 | 1.15 | 320846 | 528 | 320318 |
| 687.1 | Rash and other nonspecific skin eruption | Dermatologic | 1.03 | 0.98 | 1.07 | 325032 | 2218 | 322814 |
| 573 | Other disorders of liver | Digestive | 1.02 | 0.99 | 1.05 | 323533 | 5202 | 318331 |
| 574 | Cholelithiasis and cholecystitis | Digestive | 0.94 | 0.86 | 1.04 | 310726 | 403 | 310323 |
| 761 | Cervicalgia | Symptoms | 0.96 | 0.91 | 1.03 | 328240 | 1005 | 327235 |
| 745 | Pain in joint | Musculoskeletal | 0.97 | 0.93 | 1.02 | 328240 | 1940 | 326300 |
| 870.3 | Other open wound of head and face | Injuries & Poisonings | 1.02 | 0.99 | 1.06 | 322782 | 3051 | 319731 |
| 324 | Other CNS infection and poliomyelitis | Neurological | 0.94 | 0.84 | 1.05 | 327426 | 288 | 327138 |

| | | | | | | | | |
|---|---|---|---|---|---|---|---|---|
| 211 | Benign neoplasm of other parts of digestive system | Neoplasms | 0.98 | 0.96 | 1.01 | 319683 | 5375 | 314308 |
| 521.1 | Dental caries | Digestive | 1.02 | 0.99 | 1.06 | 314912 | 3254 | 311658 |
| 411.41 | Aneurysm and dissection of heart | Circulatory System | 0.96 | 0.89 | 1.03 | 296410 | 721 | 295689 |
| 331.9 | Cerebral degeneration, unspecified | Neurological | 1.06 | 0.96 | 1.17 | 286569 | 417 | 286152 |
| 286.7 | Other and unspecified coagulation defects | Hematopoietic | 1.06 | 0.96 | 1.17 | 325902 | 405 | 325497 |
| 389.1 | Sensorineural hearing loss | Sense Organs | 1.06 | 0.96 | 1.16 | 318658 | 464 | 318194 |
| 184.1 | Malignant neoplasm of ovary and other uterine adnexa | Neoplasms | 0.98 | 0.94 | 1.02 | 308106 | 2553 | 305553 |
| 709.2 | Sicca syndrome | Dermatologic | 0.95 | 0.87 | 1.04 | 242486 | 520 | 241966 |
| 275.3 | Disorders of magnesium metabolism | Endocrine/Metabolic | 0.94 | 0.85 | 1.04 | 326410 | 375 | 326035 |
| 426.9 | Cardiac pacemaker/device in situ | Circulatory System | 1.08 | 0.95 | 1.23 | 299758 | 237 | 299521 |
| 557.1 | Celiac disease | Digestive | 0.97 | 0.93 | 1.02 | 260290 | 1894 | 258396 |
| 742.8 | Articular cartilage disorder | Musculoskeletal | 1.05 | 0.97 | 1.13 | 311297 | 655 | 310642 |
| 595 | Hydronephrosis | Genitourinary | 0.98 | 0.93 | 1.02 | 321941 | 2010 | 319931 |
| 627.3 | Postmenopausal atrophic vaginitis | Genitourinary | 1.03 | 0.97 | 1.10 | 297359 | 1128 | 296231 |
| 136 | Other infectious and parasitic diseases | Infectious Diseases | 0.95 | 0.86 | 1.04 | 327982 | 428 | 327554 |
| 352.2 | Facial nerve disorders [CN7] | Neurological | 0.96 | 0.90 | 1.03 | 313911 | 900 | 313011 |
| 528.6 | Leukoplakia of oral mucosa | Digestive | 1.07 | 0.95 | 1.19 | 322846 | 312 | 322534 |
| 939 | Atopic/contact dermatitis due to other or unspecified | Dermatologic | 1.02 | 0.98 | 1.07 | 324060 | 2154 | 321906 |
| 743.9 | Osteopenia or other disorder of bone and cartilage | Musculoskeletal | 1.02 | 0.99 | 1.05 | 328188 | 3989 | 324199 |
| 614.33 | Pelvic inflammatory disease, NOS | Genitourinary | 1.01 | 0.99 | 1.04 | 324944 | 6145 | 318799 |
| 701.5 | Abnormal granulation tissue | Dermatologic | 0.95 | 0.87 | 1.04 | 323543 | 479 | 323064 |
| 526.41 | Temporomandibular joint disorder, unspecified | Digestive | 1.08 | 0.94 | 1.23 | 311886 | 228 | 311658 |
| 509.1 | Respiratory failure | Respiratory | 1.02 | 0.98 | 1.07 | 318564 | 2080 | 316484 |
| 454 | Varicose veins | Circulatory System | 0.97 | 0.93 | 1.02 | 289316 | 1603 | 287713 |
| 626.8 | Infertility, female | Genitourinary | 0.97 | 0.92 | 1.02 | 297638 | 1407 | 296231 |
| 252.1 | Hyperparathyroidism | Endocrine/Metabolic | 1.04 | 0.97 | 1.11 | 325359 | 814 | 324545 |
| 738 | Other acquired musculoskeletal deformity | Musculoskeletal | 0.95 | 0.86 | 1.04 | 317104 | 417 | 316687 |
| 215 | Other benign neoplasm of connective and other soft tissue | Neoplasms | 0.97 | 0.91 | 1.03 | 321804 | 1128 | 320676 |
| 323 | Encephalitis | Neurological | 1.04 | 0.97 | 1.11 | 327999 | 861 | 327138 |
| 523.32 | Chronic periodontitis | Digestive | 0.96 | 0.88 | 1.04 | 312248 | 590 | 311658 |
| 365 | Glaucoma | Sense Organs | 0.98 | 0.96 | 1.01 | 321340 | 4558 | 316782 |
| 312 | Conduct disorders | Mental Disorders | 1.03 | 0.97 | 1.09 | 327638 | 1195 | 326443 |
| 577.3 | Cyst and pseudocyst of pancreas | Digestive | 1.06 | 0.95 | 1.17 | 326295 | 375 | 325920 |
| 374.1 | Ectropion or entropion | Sense Organs | 0.97 | 0.91 | 1.03 | 319480 | 1092 | 318388 |
| 575.6 | Cholesterolosis of gallbladder | Digestive | 1.05 | 0.96 | 1.15 | 310789 | 466 | 310323 |
| 189 | Cancer of urinary organs (incl. kidney and bladder) | Neoplasms | 0.98 | 0.95 | 1.02 | 326965 | 3003 | 323962 |
| 573.9 | Abnormal serum enzyme levels | Digestive | 1.07 | 0.94 | 1.22 | 318561 | 230 | 318331 |
| 440.9 | Atherosclerosis of aorta | Circulatory System | 1.08 | 0.93 | 1.24 | 319562 | 202 | 319360 |

| | | | | | | | | |
|---|---|---|---|---|---|---|---|---|
| 540.11 | Acute appendicitis | Digestive | 1.02 | 0.98 | 1.06 | 327037 | 2658 | 324379 |
| 452 | Other venous embolism and thrombosis | Circulatory System | 1.04 | 0.96 | 1.14 | 288267 | 554 | 287713 |
| 300.1 | Anxiety disorder | Mental Disorders | 0.99 | 0.96 | 1.01 | 288368 | 5470 | 282898 |
| 579.2 | Splenomegaly | Digestive | 0.95 | 0.87 | 1.05 | 303837 | 443 | 303394 |
| 989 | Toxic effect of other substances, chiefly nonmedicinal as to source | Injuries & Poisonings | 1.03 | 0.97 | 1.08 | 328136 | 1438 | 326698 |
| 388 | Other disorders of ear | Sense Organs | 0.99 | 0.97 | 1.01 | 328080 | 9886 | 318194 |
| 285.1 | Acute posthemorrhagic anemia | Hematopoietic | 0.94 | 0.84 | 1.06 | 309167 | 267 | 308900 |
| 531.3 | Duodenal ulcer | Digestive | 0.98 | 0.95 | 1.02 | 323644 | 3095 | 320549 |
| 732 | Osteochondropathies | Musculoskeletal | 0.94 | 0.82 | 1.07 | 315651 | 219 | 315432 |
| 374.3 | Ptosis of eyelid | Sense Organs | 1.02 | 0.98 | 1.07 | 320284 | 1896 | 318388 |
| 320 | Meningitis | Neurological | 1.05 | 0.95 | 1.16 | 327577 | 439 | 327138 |
| 375.2 | Epiphora | Sense Organs | 0.97 | 0.91 | 1.03 | 289094 | 902 | 288192 |
| 426.21 | First degree AV block | Circulatory System | 0.97 | 0.91 | 1.03 | 300526 | 1005 | 299521 |
| 586.4 | Stricture/obstruction of ureter | Genitourinary | 0.97 | 0.91 | 1.03 | 315018 | 939 | 314079 |
| 788 | Syncope and collapse | Symptoms | 0.99 | 0.97 | 1.01 | 328240 | 9372 | 318868 |
| 618.5 | Prolapse of vaginal vault after hysterectomy | Genitourinary | 1.05 | 0.96 | 1.14 | 316743 | 501 | 316242 |
| 345.1 | Epilepsy | Neurological | 0.95 | 0.85 | 1.05 | 286483 | 331 | 286152 |
| 721.1 | Spondylosis without myelopathy | Musculoskeletal | 0.97 | 0.91 | 1.03 | 312014 | 1070 | 310944 |
| 747.11 | Cardiac shunt/ heart septal defect | Congenital Anomalies | 0.96 | 0.89 | 1.04 | 325935 | 581 | 325354 |
| 433 | Cerebrovascular disease | Circulatory System | 0.99 | 0.97 | 1.01 | 326753 | 8646 | 318107 |
| 430.1 | Subarachnoid hemorrhage | Circulatory System | 1.04 | 0.97 | 1.11 | 318931 | 824 | 318107 |
| 250.42 | Other abnormal glucose | Endocrine/Metabolic | 1.05 | 0.95 | 1.15 | 307927 | 437 | 307490 |
| 696.4 | Psoriasis | Dermatologic | 0.98 | 0.93 | 1.02 | 315554 | 1622 | 313932 |
| 599.3 | Dysuria | Genitourinary | 1.03 | 0.97 | 1.09 | 221636 | 1209 | 220427 |
| 251.1 | Hypoglycemia | Endocrine/Metabolic | 1.03 | 0.97 | 1.10 | 305952 | 977 | 304975 |
| 394.3 | Aortic valve disease | Circulatory System | 0.97 | 0.92 | 1.03 | 322836 | 1308 | 321528 |
| 596 | Other disorders of bladder | Genitourinary | 1.01 | 0.99 | 1.03 | 321385 | 7749 | 313636 |
| 433.31 | Transient cerebral ischemia | Circulatory System | 1.02 | 0.98 | 1.07 | 320281 | 2174 | 318107 |
| 458.1 | Orthostatic hypotension | Circulatory System | 1.03 | 0.97 | 1.08 | 195553 | 1374 | 194179 |
| 279.7 | Other immunological findings | Endocrine/Metabolic | 1.06 | 0.94 | 1.20 | 327978 | 264 | 327714 |
| 771.1 | Swelling of limb | Symptoms | 0.97 | 0.90 | 1.03 | 327356 | 842 | 326514 |
| 751.22 | Other specified congenital anomalies of kidney | Congenital Anomalies | 1.05 | 0.95 | 1.17 | 326353 | 350 | 326003 |
| 735.23 | Hallux rigidus | Musculoskeletal | 1.02 | 0.97 | 1.08 | 318243 | 1556 | 316687 |
| 41.1 | Staphylococcus infections | Infectious Diseases | 1.02 | 0.98 | 1.05 | 315832 | 3200 | 312632 |
| 381.9 | Otorrhea | Sense Organs | 0.95 | 0.84 | 1.06 | 324420 | 277 | 324143 |
| 470 | Septal Deviations/Turbinate Hypertrophy | Respiratory | 0.99 | 0.96 | 1.01 | 313771 | 4821 | 308950 |
| 574.1 | Cholelithiasis | Digestive | 0.99 | 0.97 | 1.01 | 319629 | 9306 | 310323 |
| 411.3 | Angina pectoris | Circulatory System | 0.99 | 0.98 | 1.01 | 312048 | 16359 | 295689 |

| | | | | | | | | |
|---|---|---|---|---|---|---|---|---|
| 304 | Adjustment reaction | Mental Disorders | 1.06 | 0.94 | 1.18 | 283221 | 323 | 282898 |
| 653 | Problems associated with amniotic cavity and membranes | Pregnancy Complications | 0.98 | 0.93 | 1.03 | 328119 | 1523 | 326596 |
| 594 | Urinary calculus | Genitourinary | 0.99 | 0.97 | 1.01 | 326897 | 6966 | 319931 |
| 497 | Bronchitis | Respiratory | 0.96 | 0.89 | 1.04 | 294834 | 642 | 294192 |
| 613.7 | Other signs and symptoms in breast | Genitourinary | 1.04 | 0.96 | 1.11 | 323169 | 751 | 322418 |
| 614.3 | Pelvic inflammatory disease (PID) | Genitourinary | 1.06 | 0.94 | 1.21 | 319048 | 249 | 318799 |
| 184.11 | Malignant neoplasm of ovary | Neoplasms | 0.99 | 0.96 | 1.01 | 310562 | 5009 | 305553 |
| 472 | Chronic pharyngitis and nasopharyngitis | Respiratory | 1.03 | 0.97 | 1.10 | 309897 | 947 | 308950 |
| 686.3 | Pilonidal cyst | Dermatologic | 1.04 | 0.96 | 1.13 | 317024 | 617 | 316407 |
| 296.22 | Major depressive disorder | Mental Disorders | 1.05 | 0.95 | 1.16 | 283282 | 384 | 282898 |
| 301 | Personality disorders | Mental Disorders | 1.05 | 0.95 | 1.15 | 283330 | 432 | 282898 |
| 496.3 | Bronchiectasis | Respiratory | 1.02 | 0.98 | 1.07 | 296077 | 1885 | 294192 |
| 564 | Functional digestive disorders | Digestive | 0.97 | 0.91 | 1.03 | 259379 | 983 | 258396 |
| 726 | Peripheral enthesopathies and allied syndromes | Musculoskeletal | 0.99 | 0.97 | 1.01 | 313722 | 8768 | 304954 |
| 289.4 | Lymphadenitis | Hematopoietic | 0.98 | 0.95 | 1.02 | 323033 | 2715 | 320318 |
| 613.8 | Other specified disorders of breast | Genitourinary | 1.05 | 0.94 | 1.17 | 322773 | 355 | 322418 |
| 513.4 | Hyperventilation | Respiratory | 1.06 | 0.94 | 1.19 | 327904 | 280 | 327624 |
| 604.1 | Redundant prepuce and phimosis/BXO | Genitourinary | 0.98 | 0.95 | 1.02 | 310716 | 2810 | 307906 |
| 578.2 | Blood in stool | Digestive | 1.02 | 0.98 | 1.06 | 306143 | 2749 | 303394 |
| 474.2 | Chronic tonsillitis and adenoiditis | Respiratory | 1.03 | 0.97 | 1.09 | 310135 | 1185 | 308950 |
| 550.1 | Inguinal hernia | Digestive | 1.01 | 0.99 | 1.02 | 296174 | 16385 | 279789 |
| 531 | Peptic ulcer (excl. esophageal) | Digestive | 0.95 | 0.84 | 1.06 | 320833 | 284 | 320549 |
| 575.2 | Obstruction of bile duct | Digestive | 0.97 | 0.90 | 1.04 | 311135 | 812 | 310323 |
| 556.1 | Ulceration of intestine | Digestive | 1.04 | 0.96 | 1.12 | 259087 | 691 | 258396 |
| 696.41 | Psoriasis vulgaris | Dermatologic | 0.98 | 0.93 | 1.03 | 315655 | 1723 | 313932 |
| 242.1 | Graves' disease | Endocrine/Metabolic | 0.96 | 0.88 | 1.05 | 310789 | 460 | 310329 |
| 8.52 | Intestinal infection due to C. difficile | Infectious Diseases | 0.97 | 0.89 | 1.04 | 319727 | 648 | 319079 |
| 255.21 | Glucocorticoid deficiency | Endocrine/Metabolic | 1.05 | 0.95 | 1.16 | 324917 | 372 | 324545 |
| 741.4 | Joint effusions | Musculoskeletal | 0.95 | 0.85 | 1.06 | 310925 | 283 | 310642 |
| 331 | Other cerebral degenerations | Neurological | 0.95 | 0.83 | 1.07 | 286392 | 240 | 286152 |
| 722.6 | Degeneration of intervertebral disc | Musculoskeletal | 0.98 | 0.95 | 1.02 | 313866 | 2922 | 310944 |
| 726.3 | Bursitis | Musculoskeletal | 0.96 | 0.89 | 1.05 | 305497 | 543 | 304954 |
| 187.1 | Malignant neoplasm of unspecified male genital organ | Neoplasms | 1.01 | 0.99 | 1.03 | 327699 | 8481 | 319218 |
| 427.8 | Sinoatrial node dysfunction (Bradycardia) | Circulatory System | 0.96 | 0.87 | 1.05 | 299940 | 419 | 299521 |
| 195 | Cancer, suspected or other | Neoplasms | 1.03 | 0.96 | 1.10 | 230227 | 915 | 229312 |
| 789 | Nausea and vomiting | Symptoms | 0.99 | 0.97 | 1.01 | 328240 | 11983 | 316257 |
| 426.24 | Atrioventricular block, complete | Circulatory System | 0.96 | 0.89 | 1.05 | 300112 | 591 | 299521 |
| 531.4 | Peptic ulcer, site unspecified | Digestive | 0.96 | 0.88 | 1.05 | 320998 | 449 | 320549 |

| | | | | | | | | | |
|---|---|---|---|---|---|---|---|---|---|
| 614.4 | Inflammatory diseases of uterus, except cervix | Genitourinary | 0.95 | 0.85 | 1.06 | 319107 | 308 | 318799 |
| 367.1 | Myopia | Sense Organs | 0.98 | 0.92 | 1.03 | 327025 | 1271 | 325754 |
| 315 | Develomental delays and disorders | Mental Disorders | 0.96 | 0.89 | 1.05 | 326992 | 549 | 326443 |
| 300.12 | Agorophobia, social phobia, and panic disorder | Mental Disorders | 0.97 | 0.90 | 1.04 | 283642 | 744 | 282898 |
| 509.8 | Dependence on respirator [Ventilator] or supplemental oxygen | Respiratory | 1.05 | 0.95 | 1.16 | 316885 | 401 | 316484 |
| 296 | Mood disorders | Mental Disorders | 1.01 | 0.99 | 1.03 | 295853 | 12955 | 282898 |
| 722 | Intervertebral disc disorders | Musculoskeletal | 1.01 | 0.99 | 1.04 | 316907 | 5963 | 310944 |
| 526.9 | Jaw disease NOS | Digestive | 0.99 | 0.98 | 1.01 | 327620 | 15962 | 311658 |
| 771 | Musculoskeletal symptoms referable to limbs | Symptoms | 0.97 | 0.91 | 1.04 | 327405 | 891 | 326514 |
| 153.3 | Malignant neoplasm of rectum, rectosigmoid junction, and anus | Neoplasms | 0.98 | 0.94 | 1.02 | 303397 | 2117 | 301280 |
| 386.3 | Labyrinthitis | Sense Organs | 1.03 | 0.96 | 1.11 | 322440 | 791 | 321649 |
| 717 | Polymyalgia Rheumatica | Musculoskeletal | 0.98 | 0.92 | 1.03 | 328240 | 1147 | 327093 |
| 527.2 | Sialoadenitis | Digestive | 0.96 | 0.87 | 1.06 | 322908 | 374 | 322534 |
| 444 | Arterial embolism and thrombosis | Circulatory System | 0.96 | 0.87 | 1.05 | 319794 | 434 | 319360 |
| 496 | Chronic airway obstruction | Respiratory | 0.99 | 0.97 | 1.01 | 301827 | 7635 | 294192 |
| 218 | Benign neoplasm of uterus | Neoplasms | 1.05 | 0.94 | 1.16 | 308168 | 388 | 307780 |
| 366.2 | Senile cataract | Sense Organs | 0.99 | 0.97 | 1.01 | 315842 | 8458 | 307384 |
| 526 | Diseases of the jaws | Digestive | 0.99 | 0.98 | 1.01 | 327645 | 15987 | 311658 |
| 443.1 | Raynaud's syndrome | Circulatory System | 0.98 | 0.92 | 1.03 | 320525 | 1165 | 319360 |
| 379 | Other disorders of eye | Sense Organs | 1.00 | 0.98 | 1.01 | 328190 | 39998 | 288192 |
| 427.7 | Tachycardia NOS | Circulatory System | 0.98 | 0.94 | 1.02 | 301787 | 2266 | 299521 |
| 749 | Congenital anomalies of face and neck | Congenital Anomalies | 0.95 | 0.85 | 1.07 | 328137 | 271 | 327866 |
| 216 | Benign neoplasm of skin | Neoplasms | 1.01 | 0.99 | 1.03 | 327614 | 7865 | 319749 |
| 703.1 | Ingrowing nail | Dermatologic | 1.03 | 0.96 | 1.09 | 322439 | 1010 | 321429 |
| 447 | Other disorders of arteries and arterioles | Circulatory System | 0.97 | 0.89 | 1.05 | 319916 | 556 | 319360 |
| 760 | Back pain | Symptoms | 0.99 | 0.96 | 1.01 | 328240 | 5923 | 322317 |
| 331.1 | Hydrocephalus | Neurological | 1.04 | 0.95 | 1.14 | 286652 | 500 | 286152 |
| 228 | Hemangioma and lymphangioma, any site | Neoplasms | 1.02 | 0.97 | 1.07 | 328240 | 1635 | 326605 |
| 378.1 | Strabismus (not specified as paralytic) | Sense Organs | 0.97 | 0.91 | 1.04 | 289139 | 947 | 288192 |
| 379.2 | Disorders of vitreous body | Sense Organs | 0.98 | 0.93 | 1.03 | 289599 | 1407 | 288192 |
| 368 | Visual disturbances | Sense Organs | 1.03 | 0.97 | 1.09 | 325963 | 1081 | 324882 |
| 377 | Disorders of optic nerve and visual pathways | Sense Organs | 0.96 | 0.88 | 1.06 | 288628 | 436 | 288192 |
| 949 | Allergies, other | Injuries & Poisonings | 0.96 | 0.87 | 1.06 | 322326 | 420 | 321906 |
| 426.91 | Cardiac pacemaker in situ | Circulatory System | 0.98 | 0.94 | 1.02 | 301837 | 2316 | 299521 |
| 345 | Epilepsy, recurrent seizures, convulsions | Neurological | 0.99 | 0.95 | 1.02 | 289613 | 3461 | 286152 |
| 427.6 | Premature beats | Circulatory System | 1.04 | 0.95 | 1.14 | 299979 | 458 | 299521 |
| 528.11 | Stomatitis and mucositis (ulcerative) | Digestive | 0.96 | 0.87 | 1.06 | 322945 | 411 | 322534 |
| 590 | Pyelonephritis | Genitourinary | 0.99 | 0.96 | 1.02 | 300960 | 3925 | 297035 |

| | | | | | | | | |
|---|---|---|---|---|---|---|---|---|
| 514 | Abnormal findings examination of lungs | Respiratory | 1.02 | 0.98 | 1.06 | 328212 | 2577 | 325635 |
| 433.21 | Cerebral artery occlusion, with cerebral infarction | Circulatory System | 1.02 | 0.97 | 1.07 | 319641 | 1534 | 318107 |
| 870 | Open wounds of head; neck; and trunk | Injuries & Poisonings | 0.97 | 0.89 | 1.05 | 320327 | 596 | 319731 |
| 592.13 | Chronic interstitial cystitis | Genitourinary | 1.05 | 0.93 | 1.19 | 297289 | 254 | 297035 |
| 250.41 | Impaired fasting glucose | Endocrine/Metabolic | 1.05 | 0.93 | 1.19 | 307758 | 268 | 307490 |
| 529 | Diseases and other conditions of the tongue | Digestive | 1.03 | 0.96 | 1.10 | 323485 | 951 | 322534 |
| 172.2 | Other non-epithelial cancer of skin | Neoplasms | 0.99 | 0.97 | 1.01 | 325462 | 11242 | 314220 |
| 182 | Malignant neoplasm of uterus | Neoplasms | 0.98 | 0.93 | 1.03 | 298948 | 1303 | 297645 |
| 306.9 | Tension headache | Mental Disorders | 1.05 | 0.93 | 1.17 | 283203 | 305 | 282898 |
| 610.1 | Cystic mastopathy | Genitourinary | 1.03 | 0.96 | 1.09 | 321832 | 959 | 320873 |
| 619.3 | Noninflammatory disorders of cervix | Genitourinary | 1.02 | 0.98 | 1.05 | 281393 | 2845 | 278548 |
| 285.2 | Anemia of chronic disease | Hematopoietic | 1.05 | 0.93 | 1.18 | 309194 | 294 | 308900 |
| 686.4 | Pyogenic granuloma | Dermatologic | 0.96 | 0.87 | 1.06 | 316787 | 380 | 316407 |
| 476 | Allergic rhinitis | Respiratory | 0.98 | 0.92 | 1.04 | 310025 | 1075 | 308950 |
| 289 | Other diseases of blood and blood-forming organs | Hematopoietic | 1.01 | 0.98 | 1.04 | 325300 | 4982 | 320318 |
| 579.8 | Nonspecific abnormal findings in stool contents | Digestive | 1.02 | 0.97 | 1.07 | 305134 | 1740 | 303394 |
| 728.7 | Fasciitis | Musculoskeletal | 1.05 | 0.92 | 1.21 | 305167 | 213 | 304954 |
| 627 | Menopausal and postmenopausal disorders | Genitourinary | 1.03 | 0.96 | 1.10 | 297078 | 847 | 296231 |
| 290.1 | Dementias | Mental Disorders | 1.03 | 0.95 | 1.11 | 322121 | 651 | 321470 |
| 624.2 | Atrophy of female genital tract | Genitourinary | 0.97 | 0.88 | 1.06 | 319111 | 442 | 318669 |
| 979 | Adverse drug events and drug allergies | Injuries & Poisonings | 1.03 | 0.95 | 1.11 | 300927 | 716 | 300211 |
| 618 | Genital prolapse | Genitourinary | 0.97 | 0.90 | 1.05 | 316862 | 620 | 316242 |
| 227.2 | Benign neoplasm of parathyroid gland | Neoplasms | 1.04 | 0.94 | 1.15 | 327032 | 390 | 326642 |
| 798.1 | Chronic fatigue syndrome | Symptoms | 0.97 | 0.90 | 1.05 | 325335 | 606 | 324729 |
| 418 | Nonspecific chest pain | Circulatory System | 1.00 | 0.98 | 1.01 | 326032 | 30212 | 295820 |
| 246 | Other disorders of thyroid | Endocrine/Metabolic | 0.99 | 0.98 | 1.01 | 327738 | 17409 | 310329 |
| 451 | Phlebitis and thrombophlebitis | Circulatory System | 0.96 | 0.87 | 1.06 | 288110 | 397 | 287713 |
| 287.3 | Thrombocytopenia | Hematopoietic | 1.02 | 0.97 | 1.08 | 326768 | 1271 | 325497 |
| 480 | Pneumonia | Respiratory | 0.99 | 0.96 | 1.02 | 321603 | 3914 | 317689 |
| 619.1 | Noninflammatory disorders of ovary, fallopian tube, and broad ligament | Genitourinary | 0.97 | 0.91 | 1.05 | 279282 | 734 | 278548 |
| 938.2 | Chronic dermatitis due to solar radiation | Injuries & Poisonings | 0.96 | 0.85 | 1.08 | 322170 | 264 | 321906 |
| 117 | Mycoses | Infectious Diseases | 1.01 | 0.98 | 1.05 | 328235 | 2735 | 325500 |
| 530.3 | Stricture and stenosis of esophagus | Digestive | 0.98 | 0.94 | 1.03 | 289757 | 1954 | 287803 |
| 368.9 | Subjective visual disturbances | Sense Organs | 0.97 | 0.90 | 1.05 | 325515 | 633 | 324882 |
| 531.1 | Hemorrhage from gastrointestinal ulcer | Digestive | 0.97 | 0.90 | 1.05 | 321204 | 655 | 320549 |
| 496.2 | Chronic bronchitis | Respiratory | 0.96 | 0.86 | 1.07 | 294485 | 293 | 294192 |
| 626.12 | Excessive or frequent menstruation | Genitourinary | 1.01 | 0.99 | 1.03 | 306272 | 10041 | 296231 |
| 512.1 | Wheezing | Respiratory | 1.05 | 0.92 | 1.20 | 314499 | 231 | 314268 |

| | | | | | | | | | |
|---|---|---|---|---|---|---|---|---|---|
| 800.3 | Fracture of tibia and fibula | Injuries & Poisonings | 1.03 | 0.95 | 1.12 | 324092 | 535 | 323557 |
| 599 | Other symptoms/disorders or the urinary system | Genitourinary | 1.00 | 1.00 | 1.01 | 324256 | 103829 | 220427 |
| 350.2 | Abnormality of gait | Neurological | 1.02 | 0.97 | 1.07 | 327077 | 1633 | 325444 |
| 751.1 | Congenital anomalies of genital organs | Congenital Anomalies | 0.97 | 0.91 | 1.05 | 326709 | 706 | 326003 |
| 379.5 | Disorders of iris and ciliary body | Sense Organs | 1.04 | 0.94 | 1.14 | 288604 | 412 | 288192 |
| 803 | Fracture of upper limb | Injuries & Poisonings | 0.97 | 0.88 | 1.06 | 323990 | 433 | 323557 |
| 790.6 | Other abnormal blood chemistry | Symptoms | 0.99 | 0.97 | 1.02 | 328029 | 6529 | 321500 |
| 687 | Symptoms affecting skin | Dermatologic | 0.95 | 0.84 | 1.09 | 323032 | 218 | 322814 |
| 702.2 | Seborrheic keratosis | Dermatologic | 0.99 | 0.95 | 1.02 | 325769 | 3169 | 322600 |
| 200.1 | Polycythemia vera | Neoplasms | 1.04 | 0.94 | 1.14 | 318136 | 404 | 317732 |
| 198.6 | Secondary malignancy of bone | Neoplasms | 1.02 | 0.97 | 1.06 | 231505 | 2193 | 229312 |
| 333.4 | Torsion dystonia | Neurological | 1.04 | 0.92 | 1.18 | 286417 | 265 | 286152 |
| 735.2 | Acquired toe deformities | Musculoskeletal | 1.01 | 0.97 | 1.06 | 318913 | 2226 | 316687 |
| 446.5 | Giant cell arteritis | Circulatory System | 1.04 | 0.94 | 1.15 | 319750 | 390 | 319360 |
| 256.4 | Polycystic ovaries | Endocrine/Metabolic | 1.05 | 0.92 | 1.20 | 324764 | 219 | 324545 |
| 557 | Intestinal malabsorption (non-celiac) | Digestive | 1.04 | 0.93 | 1.17 | 258690 | 294 | 258396 |
| 90 | Sexually transmitted infections (not HIV or hepatitis) | Infectious Diseases | 0.96 | 0.86 | 1.08 | 328239 | 298 | 327941 |
| 618.6 | Vaginal enterocele, congenital or acquired | Genitourinary | 1.03 | 0.95 | 1.11 | 316909 | 667 | 316242 |
| 218.1 | Uterine leiomyoma | Neoplasms | 1.01 | 0.99 | 1.03 | 318346 | 10566 | 307780 |
| 801 | Fracture of ankle and foot | Injuries & Poisonings | 0.96 | 0.85 | 1.08 | 323808 | 251 | 323557 |
| 379.3 | Aphakia and other disorders of lens | Sense Organs | 1.00 | 0.98 | 1.01 | 308920 | 20728 | 288192 |
| 528.5 | Diseases of lips | Digestive | 0.97 | 0.90 | 1.05 | 323215 | 681 | 322534 |
| 580.32 | Nephritis and nephropathy with pathological lesion | Genitourinary | 0.98 | 0.92 | 1.04 | 315157 | 1078 | 314079 |
| 800 | Fracture of lower limb | Injuries & Poisonings | 1.03 | 0.95 | 1.11 | 324256 | 699 | 323557 |
| 250.7 | Diabetic retinopathy | Endocrine/Metabolic | 1.02 | 0.97 | 1.07 | 317232 | 1380 | 315852 |
| 592.12 | Chronic cystitis | Genitourinary | 0.98 | 0.92 | 1.04 | 297948 | 913 | 297035 |
| 618.1 | Prolapse of vaginal walls | Genitourinary | 0.99 | 0.97 | 1.02 | 323726 | 7484 | 316242 |
| 285 | Other anemias | Hematopoietic | 1.01 | 0.99 | 1.02 | 320744 | 11844 | 308900 |
| 592.1 | Cystitis | Genitourinary | 1.01 | 0.97 | 1.06 | 299089 | 2054 | 297035 |
| 333 | Extrapyramidal disease and abnormal movement disorders | Neurological | 1.01 | 0.97 | 1.06 | 288187 | 2035 | 286152 |
| 598 | Abnormal findings on examination of urine | Genitourinary | 0.99 | 0.96 | 1.02 | 328240 | 3386 | 324854 |
| 571.81 | Portal hypertension | Digestive | 1.03 | 0.95 | 1.12 | 318900 | 569 | 318331 |
| 296.2 | Depression | Mental Disorders | 1.01 | 0.99 | 1.02 | 295043 | 12145 | 282898 |
| 240 | Simple and unspecified goiter | Endocrine/Metabolic | 1.03 | 0.95 | 1.11 | 310951 | 622 | 310329 |
| 375 | Disorders of lacrimal system | Sense Organs | 0.98 | 0.94 | 1.03 | 289852 | 1660 | 288192 |
| 512.8 | Cough | Respiratory | 1.01 | 0.98 | 1.05 | 317232 | 2964 | 314268 |
| 415 | Pulmonary heart disease | Circulatory System | 1.01 | 0.98 | 1.04 | 325300 | 3753 | 321547 |
| 54 | Herpes simplex | Infectious Diseases | 0.96 | 0.84 | 1.09 | 322425 | 219 | 322206 |

| | | | | | | | | |
|---|---|---|---|---|---|---|---|---|
| 614.53 | Cyst or abscess of Bartholin's gland | Genitourinary | 1.02 | 0.95 | 1.10 | 319602 | 803 | 318799 |
| 626.1 | Irregular menstrual cycle/bleeding | Genitourinary | 1.01 | 0.98 | 1.05 | 299824 | 3593 | 296231 |
| 290 | Delirium dementia and amnestic and other cognitive disorders | Mental Disorders | 1.03 | 0.94 | 1.13 | 321950 | 480 | 321470 |
| 359.2 | Myopathy | Neurological | 1.03 | 0.94 | 1.13 | 326450 | 498 | 325952 |
| 714.1 | Rheumatoid arthritis | Musculoskeletal | 1.01 | 0.97 | 1.06 | 319560 | 2307 | 317253 |
| 327 | Sleep disorders | Neurological | 0.98 | 0.92 | 1.04 | 323754 | 1034 | 322720 |
| 292.2 | Mild cognitive impairment | Mental Disorders | 1.05 | 0.91 | 1.20 | 321672 | 202 | 321470 |
| 722.1 | Displacement of intervertebral disc | Musculoskeletal | 0.97 | 0.89 | 1.06 | 311460 | 516 | 310944 |
| 340 | Migraine | Neurological | 1.01 | 0.97 | 1.05 | 320723 | 2773 | 317950 |
| 187.2 | Malignant neoplasm of testis | Neoplasms | 0.99 | 0.96 | 1.02 | 323790 | 4572 | 319218 |
| 577 | Diseases of pancreas | Digestive | 0.97 | 0.90 | 1.06 | 326478 | 558 | 325920 |
| 365.11 | Primary open angle glaucoma | Sense Organs | 0.98 | 0.92 | 1.04 | 317838 | 1056 | 316782 |
| 707.1 | Decubitus ulcer | Dermatologic | 1.04 | 0.93 | 1.16 | 327025 | 326 | 326699 |
| 386.1 | Meniere's disease | Sense Organs | 1.03 | 0.94 | 1.12 | 322213 | 564 | 321649 |
| 568.1 | Peritoneal adhesions (postoperative) (postinfection) | Digestive | 1.01 | 0.98 | 1.05 | 249757 | 3097 | 246660 |
| 565.1 | Anal and rectal polyp | Digestive | 0.99 | 0.97 | 1.02 | 254329 | 7669 | 246660 |
| 185 | Cancer of prostate | Neoplasms | 1.01 | 0.98 | 1.03 | 315856 | 8463 | 307393 |
| 721 | Spondylosis and allied disorders | Musculoskeletal | 1.02 | 0.96 | 1.09 | 311869 | 925 | 310944 |
| 614.5 | Inflammatory disease of cervix, vagina, and vulva | Genitourinary | 0.97 | 0.87 | 1.08 | 319133 | 334 | 318799 |
| 411.1 | Unstable angina (intermediate coronary syndrome) | Circulatory System | 1.01 | 0.98 | 1.04 | 301011 | 5322 | 295689 |
| 280.1 | Iron deficiency anemias, unspecified or not due to blood loss | Hematopoietic | 1.01 | 0.98 | 1.03 | 316402 | 7502 | 308900 |
| 79 | Viral infection | Infectious Diseases | 0.99 | 0.96 | 1.02 | 326439 | 4233 | 322206 |
| 807 | Fracture of ribs | Injuries & Poisonings | 0.96 | 0.84 | 1.09 | 323780 | 223 | 323557 |
| 458.9 | Hypotension NOS | Circulatory System | 1.01 | 0.98 | 1.04 | 197817 | 3638 | 194179 |
| 290.11 | Alzheimer's disease | Mental Disorders | 1.03 | 0.93 | 1.14 | 321872 | 402 | 321470 |
| 958 | Certain early complications of trauma or procedure | Injuries & Poisonings | 0.97 | 0.88 | 1.07 | 328118 | 381 | 327737 |
| 614 | Inflammatory diseases of female pelvic organs | Genitourinary | 1.03 | 0.93 | 1.15 | 319173 | 374 | 318799 |
| 302 | Sexual and gender identity disorders | Mental Disorders | 0.97 | 0.87 | 1.08 | 283238 | 340 | 282898 |
| 425.1 | Primary/intrinsic cardiomyopathies | Circulatory System | 0.98 | 0.92 | 1.04 | 326036 | 1053 | 324983 |
| 389 | Hearing loss | Sense Organs | 0.99 | 0.96 | 1.02 | 321364 | 3170 | 318194 |
| 350.3 | Lack of coordination | Neurological | 0.97 | 0.87 | 1.07 | 325804 | 360 | 325444 |
| 371 | Inflammation of the eye | Sense Organs | 1.03 | 0.93 | 1.14 | 318796 | 408 | 318388 |
| 368.2 | Diplopia and disorders of binocular vision | Sense Organs | 1.02 | 0.95 | 1.10 | 325635 | 753 | 324882 |
| 627.4 | Premenopausal menorrhagia | Genitourinary | 0.97 | 0.87 | 1.08 | 296563 | 332 | 296231 |
| 530.9 | Heartburn | Digestive | 1.01 | 0.97 | 1.06 | 289911 | 2108 | 287803 |
| 615 | Endometriosis | Genitourinary | 1.01 | 0.98 | 1.04 | 322888 | 4089 | 318799 |
| 836 | Traumatic arthropathy | Injuries & Poisonings | 1.04 | 0.91 | 1.18 | 322016 | 243 | 321773 |
| 327.3 | Sleep apnea | Neurological | 0.99 | 0.96 | 1.02 | 327419 | 4699 | 322720 |

| | | | | | | | | |
|---|---|---|---|---|---|---|---|---|
| 112 | Candidiasis | Infectious Diseases | 1.01 | 0.97 | 1.06 | 327650 | 2150 | 325500 |
| 245.21 | Chronic lymphocytic thyroiditis | Endocrine/Metabolic | 0.96 | 0.84 | 1.10 | 310548 | 219 | 310329 |
| 994.2 | Sepsis | Injuries & Poisonings | 1.01 | 0.97 | 1.05 | 328240 | 2885 | 325355 |
| 520 | Disorders of tooth development | Digestive | 1.03 | 0.94 | 1.13 | 312128 | 470 | 311658 |
| 395.2 | Nonrheumatic aortic valve disorders | Circulatory System | 0.97 | 0.85 | 1.09 | 321775 | 247 | 321528 |
| 420.21 | Acute pericarditis | Circulatory System | 0.96 | 0.85 | 1.10 | 325211 | 228 | 324983 |
| 353 | Nerve root and plexus disorders | Neurological | 0.98 | 0.92 | 1.05 | 314005 | 994 | 313011 |
| 752.11 | Spina bifida | Congenital Anomalies | 1.04 | 0.91 | 1.19 | 327886 | 211 | 327675 |
| 743.11 | Osteoporosis NOS | Musculoskeletal | 1.01 | 0.97 | 1.05 | 327235 | 3036 | 324199 |
| 198.3 | Secondary malignant neoplasm of digestive systems | Neoplasms | 0.99 | 0.94 | 1.04 | 230877 | 1565 | 229312 |
| 229 | Benign neoplasm of unspecified sites | Neoplasms | 1.00 | 0.99 | 1.01 | 328240 | 46165 | 282075 |
| 621 | Endometrial hyperplasia | Genitourinary | 1.02 | 0.96 | 1.08 | 316461 | 1154 | 315307 |
| 870.4 | Open wound of nose and sinus | Injuries & Poisonings | 0.97 | 0.85 | 1.09 | 319969 | 238 | 319731 |
| 550 | Abdominal hernia | Digestive | 1.00 | 0.99 | 1.01 | 328240 | 48451 | 279789 |
| 573.5 | Jaundice (not of newborn) | Digestive | 0.98 | 0.92 | 1.05 | 319269 | 938 | 318331 |
| 214 | Lipoma | Neoplasms | 1.01 | 0.97 | 1.06 | 322495 | 1819 | 320676 |
| 284 | Aplastic anemia | Hematopoietic | 1.00 | 0.99 | 1.02 | 321659 | 12759 | 308900 |
| 735 | Acquired foot deformities | Musculoskeletal | 0.97 | 0.87 | 1.09 | 316986 | 299 | 316687 |
| 823 | Fracture of tibia and fibula | Injuries & Poisonings | 1.03 | 0.93 | 1.15 | 322362 | 342 | 322020 |
| 726.2 | Synoviopathy | Musculoskeletal | 0.97 | 0.87 | 1.08 | 305285 | 331 | 304954 |
| 361 | Retinal detachments and defects | Sense Organs | 1.01 | 0.97 | 1.05 | 319011 | 2229 | 316782 |
| 602 | Other disorders of prostate | Genitourinary | 0.99 | 0.93 | 1.04 | 308765 | 1372 | 307393 |
| 348.2 | Cerebral edema and compression of brain | Neurological | 1.03 | 0.91 | 1.17 | 286412 | 260 | 286152 |
| 512.2 | Painful respiration | Respiratory | 0.97 | 0.85 | 1.10 | 314496 | 228 | 314268 |
| 217.1 | Nevus, non-neoplastic | Neoplasms | 1.02 | 0.94 | 1.11 | 320346 | 597 | 319749 |
| 362 | Other retinal disorders | Sense Organs | 1.02 | 0.95 | 1.08 | 316825 | 973 | 315852 |
| 300 | Anxiety, phobic and dissociative disorders | Mental Disorders | 0.97 | 0.88 | 1.08 | 283261 | 363 | 282898 |
| 512.7 | Shortness of breath | Respiratory | 0.99 | 0.97 | 1.02 | 320384 | 6116 | 314268 |
| 614.51 | Cervicitis and endocervicitis | Genitourinary | 1.02 | 0.96 | 1.07 | 320048 | 1249 | 318799 |
| 150 | Cancer of esophagus | Neoplasms | 0.98 | 0.91 | 1.05 | 313058 | 727 | 312331 |
| 480.11 | Pneumococcal pneumonia | Respiratory | 0.99 | 0.97 | 1.02 | 323680 | 5991 | 317689 |
| 440 | Atherosclerosis | Circulatory System | 1.04 | 0.90 | 1.19 | 319564 | 204 | 319360 |
| 292.1 | Aphasia/speech disturbance | Mental Disorders | 1.01 | 0.96 | 1.07 | 323019 | 1549 | 321470 |
| 286.12 | Congenital deficiency of other clotting factors (including factor VII) | Hematopoietic | 1.03 | 0.92 | 1.16 | 325800 | 303 | 325497 |
| 601.1 | Prostatitis | Genitourinary | 1.02 | 0.94 | 1.10 | 308058 | 665 | 307393 |
| 428.2 | Heart failure NOS | Circulatory System | 0.99 | 0.96 | 1.02 | 326367 | 4334 | 322033 |
| 740.11 | Osteoarthrosis, localized, primary | Musculoskeletal | 1.01 | 0.98 | 1.03 | 315552 | 9227 | 306325 |
| 610 | Benign mammary dysplasias | Genitourinary | 1.04 | 0.90 | 1.19 | 321081 | 208 | 320873 |

| | | | | | | | | |
|---|---|---|---|---|---|---|---|---|
| 613 | Other nonmalignant breast conditions | Genitourinary | 1.03 | 0.93 | 1.14 | 322786 | 368 | 322418 |
| 496.21 | Obstructive chronic bronchitis | Respiratory | 0.99 | 0.95 | 1.03 | 296921 | 2729 | 294192 |
| 174.11 | Malignant neoplasm of female breast | Neoplasms | 1.00 | 0.99 | 1.02 | 316838 | 12414 | 304424 |
| 496.1 | Emphysema | Respiratory | 0.99 | 0.94 | 1.04 | 295918 | 1726 | 294192 |
| 750 | Digestive congenital anomalies | Congenital Anomalies | 0.98 | 0.91 | 1.06 | 326705 | 702 | 326003 |
| 635.3 | Placenta previa and abruptio placenta | Pregnancy Complications | 0.99 | 0.93 | 1.04 | 321301 | 1336 | 319965 |
| 292.4 | Altered mental status | Mental Disorders | 1.01 | 0.97 | 1.05 | 323742 | 2272 | 321470 |
| 624.1 | Dystrophy of female genital tract | Genitourinary | 0.97 | 0.85 | 1.10 | 318909 | 240 | 318669 |
| 574.12 | Cholelithiasis with other cholecystitis | Digestive | 0.99 | 0.97 | 1.02 | 315816 | 5493 | 310323 |
| 752 | Nervous system congenital anomalies | Congenital Anomalies | 1.03 | 0.92 | 1.16 | 327963 | 288 | 327675 |
| 442.11 | Abdominal aortic aneurysm | Circulatory System | 0.98 | 0.92 | 1.05 | 320257 | 897 | 319360 |
| 727.1 | Synovitis and tenosynovitis | Musculoskeletal | 1.02 | 0.94 | 1.10 | 305641 | 687 | 304954 |
| 427.42 | Cardiac arrest | Circulatory System | 1.02 | 0.95 | 1.08 | 300470 | 949 | 299521 |
| 947 | Urticaria | Injuries & Poisonings | 0.98 | 0.88 | 1.08 | 322293 | 387 | 321906 |
| 512.9 | Other dyspnea | Respiratory | 1.02 | 0.95 | 1.08 | 315211 | 943 | 314268 |
| 269 | Proteinuria | Endocrine/Metabolic | 1.03 | 0.92 | 1.14 | 327850 | 355 | 327495 |
| 961 | Poisoning by other anti-infectives | Injuries & Poisonings | 1.02 | 0.93 | 1.13 | 300647 | 436 | 300211 |
| 289.8 | Polycythemia vera, secondary | Hematopoietic | 1.03 | 0.92 | 1.16 | 320039 | 291 | 319748 |
| 442.8 | Aneurysm of other specified artery | Circulatory System | 1.03 | 0.90 | 1.19 | 319570 | 210 | 319360 |
| 608 | Other disorders of male genital organs | Genitourinary | 1.00 | 0.98 | 1.01 | 327998 | 20092 | 307906 |
| 555 | Inflammatory bowel disease and other gastroenteritis and colitis | Digestive | 1.00 | 0.99 | 1.02 | 278195 | 19799 | 258396 |
| 371.3 | Inflammation of eyelids | Sense Organs | 0.99 | 0.95 | 1.03 | 320786 | 2398 | 318388 |
| 332 | Parkinson's disease | Neurological | 1.01 | 0.96 | 1.07 | 287334 | 1182 | 286152 |
| 532 | Dysphagia | Digestive | 0.99 | 0.97 | 1.02 | 294356 | 6553 | 287803 |
| 429.3 | Symptoms involving cardiovascular system | Circulatory System | 0.97 | 0.85 | 1.11 | 322241 | 208 | 322033 |
| 70.3 | Viral hepatitis C | Infectious Diseases | 0.97 | 0.88 | 1.08 | 322549 | 343 | 322206 |
| 854 | Complications of cardiac/vascular device, implant, and graft | Injuries & Poisonings | 0.99 | 0.94 | 1.04 | 315807 | 1843 | 313964 |
| 474 | Acute and chronic tonsillitis | Respiratory | 0.98 | 0.91 | 1.06 | 309554 | 604 | 308950 |
| 558 | Noninfectious gastroenteritis | Digestive | 1.00 | 0.99 | 1.02 | 278260 | 19864 | 258396 |
| 180.1 | Cervical cancer | Neoplasms | 0.99 | 0.96 | 1.02 | 302137 | 4554 | 297583 |
| 766 | Neuralgia, neuritis, and radiculitis NOS | Symptoms | 1.03 | 0.92 | 1.15 | 326987 | 304 | 326683 |
| 990 | Effects radiation NOS | Injuries & Poisonings | 1.01 | 0.97 | 1.04 | 325633 | 3244 | 322389 |
| 625.1 | Dyspareunia | Genitourinary | 1.01 | 0.96 | 1.07 | 319824 | 1155 | 318669 |
| 530.11 | GERD | Digestive | 1.00 | 0.98 | 1.01 | 302292 | 14489 | 287803 |
| 288.11 | Neutropenia | Hematopoietic | 1.01 | 0.97 | 1.04 | 323644 | 3326 | 320318 |
| 478 | Throat pain | Respiratory | 1.02 | 0.93 | 1.13 | 309337 | 387 | 308950 |
| 384.4 | Perforation of tympanic membrane | Sense Organs | 1.01 | 0.95 | 1.08 | 325167 | 1024 | 324143 |
| 803.1 | Fracture of humerus | Injuries & Poisonings | 1.03 | 0.91 | 1.15 | 323850 | 293 | 323557 |

| | | | | | | | | |
|---|---|---|---|---|---|---|---|---|
| 366 | Cataract | Sense Organs | 1.00 | 0.98 | 1.01 | 323825 | 16441 | 307384 |
| 706 | Diseases of sebaceous glands | Dermatologic | 1.02 | 0.94 | 1.11 | 318889 | 595 | 318294 |
| 378.2 | Nystagmus and other irregular eye movements | Sense Organs | 1.03 | 0.90 | 1.18 | 288407 | 215 | 288192 |
| 272.9 | Unspecified disorder of lipoid metabolism | Endocrine/Metabolic | 0.97 | 0.85 | 1.11 | 291721 | 224 | 291497 |
| 480.1 | Bacterial pneumonia | Respiratory | 1.01 | 0.95 | 1.08 | 318628 | 939 | 317689 |
| 293.1 | Swelling, mass, or lump in head and neck [Space-occupying lesion, intracranial NOS] | Mental Disorders | 0.99 | 0.92 | 1.05 | 326044 | 925 | 325119 |
| 578.1 | Hematemesis | Digestive | 0.99 | 0.95 | 1.03 | 305428 | 2034 | 303394 |
| 522 | Diseases of pulp and periapical tissues | Digestive | 0.98 | 0.91 | 1.06 | 312297 | 639 | 311658 |
| 853 | Complication of colostomy or enterostomy | Injuries & Poisonings | 0.98 | 0.90 | 1.07 | 314478 | 514 | 313964 |
| 536.8 | Dyspepsia and other specified disorders of function of stomach | Digestive | 0.97 | 0.86 | 1.10 | 296758 | 247 | 296511 |
| 345.12 | Partial epilepsy | Neurological | 1.03 | 0.91 | 1.15 | 286450 | 298 | 286152 |
| 348.7 | Coma | Neurological | 1.02 | 0.92 | 1.13 | 286531 | 379 | 286152 |
| 227.3 | Benign neoplasm of pituitary gland and craniopharyngeal duct (pouch) | Neoplasms | 1.02 | 0.92 | 1.14 | 326970 | 328 | 326642 |
| 530.7 | Gastroesophageal laceration-hemorrhage syndrome | Digestive | 0.98 | 0.88 | 1.09 | 288136 | 333 | 287803 |
| 740.12 | Osteoarthrosis, localized, secondary | Musculoskeletal | 1.03 | 0.91 | 1.16 | 306589 | 264 | 306325 |
| 510 | Other diseases of lung | Respiratory | 0.98 | 0.92 | 1.06 | 328200 | 770 | 327430 |
| 536 | Disorders of function of stomach | Digestive | 0.97 | 0.86 | 1.10 | 296768 | 257 | 296511 |
| 535.6 | Duodenitis | Digestive | 1.00 | 0.97 | 1.02 | 304405 | 7894 | 296511 |
| 480.5 | Bronchopneumonia and lung abscess | Respiratory | 1.02 | 0.92 | 1.13 | 318077 | 388 | 317689 |
| 501 | Pneumonitis due to inhalation of food or vomitus | Respiratory | 0.98 | 0.91 | 1.07 | 317076 | 592 | 316484 |
| 636 | Early or threatened labor; hemorrhage in early pregnancy | Pregnancy Complications | 1.01 | 0.96 | 1.07 | 321227 | 1262 | 319965 |
| 38 | Septicemia | Infectious Diseases | 1.01 | 0.97 | 1.04 | 315677 | 3045 | 312632 |
| 350.1 | Abnormal involuntary movements | Neurological | 0.99 | 0.93 | 1.05 | 326371 | 927 | 325444 |
| 41.2 | Streptococcus infection | Infectious Diseases | 1.01 | 0.96 | 1.06 | 314280 | 1648 | 312632 |
| 198.5 | Secondary malignancy of brain/spine | Neoplasms | 1.01 | 0.95 | 1.09 | 230113 | 801 | 229312 |
| 736 | Other acquired deformities of limbs | Musculoskeletal | 0.98 | 0.88 | 1.09 | 316998 | 311 | 316687 |
| 274.21 | Chondrocalcinosis | Endocrine/Metabolic | 0.98 | 0.87 | 1.10 | 326552 | 290 | 326262 |
| 964.1 | Anticoagulants causing adverse effects | Injuries & Poisonings | 1.03 | 0.91 | 1.16 | 300470 | 259 | 300211 |
| 292.3 | Memory loss | Mental Disorders | 0.99 | 0.92 | 1.06 | 322208 | 738 | 321470 |
| 174 | Breast cancer | Neoplasms | 1.02 | 0.91 | 1.15 | 304712 | 288 | 304424 |
| 535.2 | Atrophic gastritis | Digestive | 1.02 | 0.92 | 1.13 | 296869 | 358 | 296511 |
| 519.2 | Respiratory complications | Respiratory | 1.02 | 0.91 | 1.15 | 265743 | 296 | 265447 |
| 556 | Ulceration of the lower GI tract | Digestive | 0.98 | 0.89 | 1.08 | 258836 | 440 | 258396 |
| 202.24 | Large cell lymphoma | Neoplasms | 1.02 | 0.94 | 1.10 | 324112 | 589 | 323523 |
| 614.54 | Abscess or ulceration of vulva | Genitourinary | 0.98 | 0.87 | 1.10 | 319079 | 280 | 318799 |
| 742.9 | Other derangement of joint | Musculoskeletal | 1.02 | 0.92 | 1.13 | 311042 | 400 | 310642 |
| 70 | Viral hepatitis | Infectious Diseases | 0.99 | 0.92 | 1.06 | 322928 | 722 | 322206 |
| 418.1 | Precordial pain | Circulatory System | 0.99 | 0.96 | 1.03 | 299506 | 3686 | 295820 |

| | | | | | | | | |
|---|---|---|---|---|---|---|---|---|
| 287.31 | Primary thrombocytopenia | Hematopoietic | 1.02 | 0.93 | 1.12 | 325925 | 428 | 325497 |
| 189.2 | Cancer of bladder | Neoplasms | 0.99 | 0.92 | 1.06 | 324798 | 836 | 323962 |
| 574.11 | Cholelithiasis with acute cholecystitis | Digestive | 1.01 | 0.96 | 1.06 | 311836 | 1513 | 310323 |
| 721.8 | Other allied disorders of spine | Musculoskeletal | 1.01 | 0.97 | 1.04 | 314171 | 3227 | 310944 |
| 199 | Neoplasm of uncertain behavior | Neoplasms | 0.99 | 0.93 | 1.05 | 230468 | 1156 | 229312 |
| 568 | Other disorders of peritoneum | Digestive | 0.99 | 0.96 | 1.03 | 250464 | 3804 | 246660 |
| 226 | Benign neoplasm of thyroid glands | Neoplasms | 0.98 | 0.87 | 1.10 | 326908 | 266 | 326642 |
| 961.1 | Poisoning/allergy of sulfonamides | Injuries & Poisonings | 0.99 | 0.92 | 1.06 | 301101 | 890 | 300211 |
| 368.4 | Visual field defects | Sense Organs | 0.98 | 0.88 | 1.09 | 325215 | 333 | 324882 |
| 433.1 | Occlusion and stenosis of precerebral arteries | Circulatory System | 0.99 | 0.93 | 1.05 | 319149 | 1042 | 318107 |
| 78 | Viral warts & HPV | Infectious Diseases | 0.99 | 0.93 | 1.05 | 323218 | 1012 | 322206 |
| 610.2 | Fibroadenosis of breast | Genitourinary | 1.02 | 0.91 | 1.14 | 321189 | 316 | 320873 |
| 819 | Skull and face fracture and other intercranial injury | Injuries & Poisonings | 0.99 | 0.94 | 1.04 | 328056 | 1517 | 326539 |
| 575.7 | Other disorders of gallbladder | Digestive | 1.01 | 0.96 | 1.06 | 311719 | 1396 | 310323 |
| 610.4 | Benign neoplasm of breast | Genitourinary | 1.01 | 0.96 | 1.06 | 322359 | 1486 | 320873 |
| 189.11 | Malignant neoplasm of kidney, except pelvis | Neoplasms | 0.99 | 0.93 | 1.05 | 324997 | 1035 | 323962 |
| 292 | Neurological disorders | Mental Disorders | 0.98 | 0.90 | 1.08 | 321919 | 449 | 321470 |
| 519 | Other diseases of respiratory system, not elsewhere classified | Respiratory | 1.00 | 0.99 | 1.01 | 327615 | 62168 | 265447 |
| 465.2 | Acute pharyngitis | Respiratory | 0.99 | 0.92 | 1.06 | 326053 | 874 | 325179 |
| 966 | Poisoning by anticonvulsants and anti-Parkinsonism drugs | Injuries & Poisonings | 1.02 | 0.93 | 1.11 | 300747 | 536 | 300211 |
| 579 | Other symptoms involving abdomen and pelvis | Digestive | 1.01 | 0.95 | 1.07 | 304580 | 1186 | 303394 |
| 535.8 | Other specified gastritis | Digestive | 1.00 | 0.97 | 1.02 | 304926 | 8415 | 296511 |
| 655 | Known or suspected fetal abnormality affecting management of mother | Pregnancy Complications | 0.99 | 0.96 | 1.03 | 328240 | 4575 | 323665 |
| 560.1 | Paralytic ileus | Digestive | 1.02 | 0.93 | 1.11 | 258916 | 520 | 258396 |
| 200 | Myeloproliferative disease | Neoplasms | 1.01 | 0.94 | 1.09 | 324202 | 679 | 323523 |
| 198.1 | Secondary malignancy of lymph nodes | Neoplasms | 1.00 | 0.98 | 1.03 | 234815 | 5503 | 229312 |
| 427.11 | Paroxysmal supraventricular tachycardia | Circulatory System | 1.01 | 0.97 | 1.05 | 301952 | 2431 | 299521 |
| 197 | Chemotherapy | Neoplasms | 1.00 | 0.98 | 1.01 | 251774 | 22462 | 229312 |
| 571.8 | Liver abscess and sequelae of chronic liver disease | Digestive | 1.01 | 0.93 | 1.10 | 318904 | 573 | 318331 |
| 619.2 | Disorders of uterus, NEC | Genitourinary | 1.01 | 0.97 | 1.04 | 281939 | 3391 | 278548 |
| 614.32 | Chronic inflammatory pelvic disease | Genitourinary | 1.02 | 0.92 | 1.12 | 319224 | 425 | 318799 |
| 430.3 | Subdural hemorrhage | Circulatory System | 0.98 | 0.87 | 1.10 | 318378 | 271 | 318107 |
| 184.2 | Cancer of other female genital organs | Neoplasms | 0.98 | 0.88 | 1.10 | 305876 | 323 | 305553 |
| 433.2 | Occlusion of cerebral arteries | Circulatory System | 0.99 | 0.96 | 1.03 | 321087 | 2980 | 318107 |
| 574.3 | Cholecystitis without cholelithiasis | Digestive | 0.99 | 0.96 | 1.03 | 313141 | 2818 | 310323 |
| 634 | Miscarriage; stillbirth | Pregnancy Complications | 0.99 | 0.96 | 1.03 | 324534 | 4569 | 319965 |
| 317 | Alcohol-related disorders | Mental Disorders | 1.00 | 0.98 | 1.03 | 303529 | 5860 | 297669 |
| 610.8 | Other specified benign mammary dysplasias | Genitourinary | 1.01 | 0.94 | 1.09 | 321576 | 703 | 320873 |

| | | | | | | | | |
|---|---|---|---|---|---|---|---|---|
| 175 | Acquired absence of breast | Neoplasms | 1.01 | 0.97 | 1.05 | 305907 | 2516 | 303391 |
| 573.3 | Hepatomegaly | Digestive | 0.98 | 0.88 | 1.10 | 318641 | 310 | 318331 |
| 705.8 | Hyperhidrosis | Dermatologic | 0.99 | 0.91 | 1.07 | 318896 | 602 | 318294 |
| 195.1 | Malignant neoplasm, other | Neoplasms | 1.00 | 0.99 | 1.01 | 320138 | 90826 | 229312 |
| 281 | Other deficiency anemia | Hematopoietic | 1.00 | 0.98 | 1.03 | 317687 | 8787 | 308900 |
| 367.2 | Astigmatism | Sense Organs | 1.02 | 0.89 | 1.17 | 325964 | 210 | 325754 |
| 296.1 | Bipolar | Mental Disorders | 0.99 | 0.93 | 1.05 | 284019 | 1121 | 282898 |
| 870.1 | Open wound or laceration of eye or eyelid | Injuries & Poisonings | 0.98 | 0.87 | 1.10 | 320012 | 281 | 319731 |
| 174.1 | Breast cancer [female] | Neoplasms | 0.99 | 0.95 | 1.04 | 306711 | 2287 | 304424 |
| 724.9 | Other unspecified back disorders | Musculoskeletal | 1.00 | 0.99 | 1.02 | 328029 | 17085 | 310944 |
| 339 | Other headache syndromes | Neurological | 1.00 | 0.98 | 1.03 | 325888 | 7938 | 317950 |
| 737.3 | Kyphoscoliosis and scoliosis | Musculoskeletal | 0.98 | 0.89 | 1.09 | 317058 | 371 | 316687 |
| 550.4 | Umbilical hernia | Digestive | 1.00 | 0.96 | 1.03 | 283693 | 3904 | 279789 |
| 218.2 | Other benign neoplasm of uterus | Neoplasms | 0.98 | 0.89 | 1.09 | 308143 | 363 | 307780 |
| 41 | Bacterial infection NOS | Infectious Diseases | 1.00 | 0.98 | 1.02 | 323384 | 10752 | 312632 |
| 420.3 | Endocarditis | Circulatory System | 0.99 | 0.92 | 1.07 | 325668 | 685 | 324983 |
| 506 | Empyema and pneumothorax | Respiratory | 0.99 | 0.94 | 1.05 | 317630 | 1146 | 316484 |
| 618.2 | Uterine/Uterovaginal prolapse | Genitourinary | 1.00 | 0.98 | 1.03 | 321713 | 5471 | 316242 |
| 601.8 | Other inflammatory disorders of male genital organs | Genitourinary | 0.98 | 0.88 | 1.10 | 307698 | 305 | 307393 |
| 599.2 | Retention of urine | Genitourinary | 1.00 | 0.97 | 1.02 | 227284 | 6857 | 220427 |
| 530.14 | Reflux esophagitis | Digestive | 1.00 | 0.98 | 1.02 | 298696 | 10893 | 287803 |
| 696.42 | Psoriatic arthropathy | Dermatologic | 0.99 | 0.92 | 1.06 | 314672 | 740 | 313932 |
| 473.4 | Voice disturbance | Respiratory | 0.99 | 0.93 | 1.05 | 310051 | 1101 | 308950 |
| 280.2 | Iron deficiency anemia secondary to blood loss (chronic) | Hematopoietic | 1.01 | 0.93 | 1.11 | 309376 | 476 | 308900 |
| 426.23 | Second degree AV block | Circulatory System | 0.99 | 0.90 | 1.08 | 299982 | 461 | 299521 |
| 550.5 | Ventral hernia | Digestive | 1.00 | 0.96 | 1.03 | 283371 | 3582 | 279789 |
| 385.5 | Tympanosclerosis and middle ear disease related to otitis media | Sense Organs | 0.98 | 0.86 | 1.11 | 324379 | 236 | 324143 |
| 509.2 | Respiratory insufficiency | Respiratory | 1.01 | 0.96 | 1.06 | 318272 | 1788 | 316484 |
| 204 | Leukemia | Neoplasms | 0.99 | 0.90 | 1.08 | 323984 | 461 | 323523 |
| 250.24 | Type 2 diabetes with neurological manifestations | Endocrine/Metabolic | 1.01 | 0.93 | 1.10 | 308102 | 612 | 307490 |
| 694.2 | Other dyschromia | Dermatologic | 0.99 | 0.92 | 1.06 | 322549 | 800 | 321749 |
| 601.11 | Acute prostatitis | Genitourinary | 1.02 | 0.90 | 1.15 | 307653 | 260 | 307393 |
| 537 | Other disorders of stomach and duodenum | Digestive | 1.00 | 0.97 | 1.04 | 299994 | 3483 | 296511 |
| 735.21 | Hammer toe (acquired) | Musculoskeletal | 1.01 | 0.96 | 1.05 | 318701 | 2014 | 316687 |
| 740.9 | Osteoarthrosis NOS | Musculoskeletal | 1.00 | 0.97 | 1.03 | 310757 | 4432 | 306325 |
| 625 | Pain and other symptoms associated with female genital organs | Genitourinary | 1.01 | 0.96 | 1.05 | 320718 | 2049 | 318669 |
| 443.7 | Peripheral angiopathy in diseases classified elsewhere | Circulatory System | 0.99 | 0.90 | 1.09 | 319775 | 415 | 319360 |
| 716.2 | Unspecified monoarthritis | Musculoskeletal | 1.00 | 0.98 | 1.01 | 289797 | 15901 | 273896 |

| | | | | | | | | | |
|---|---|---|---|---|---|---|---|---|---|
| 288 | Diseases of white blood cells | Hematopoietic | 0.99 | 0.91 | 1.07 | 320928 | 610 | 320318 |
| 340.1 | Migrain with aura | Neurological | 0.98 | 0.86 | 1.12 | 318167 | 217 | 317950 |
| 634.1 | Missed abortion/Hydatidiform mole | Pregnancy Complications | 1.01 | 0.95 | 1.07 | 321168 | 1203 | 319965 |
| 381.1 | Otitis media | Sense Organs | 0.99 | 0.94 | 1.05 | 325296 | 1153 | 324143 |
| 378.5 | Paralytic strabismus | Sense Organs | 0.98 | 0.88 | 1.11 | 288479 | 287 | 288192 |
| 597.1 | Urethral stricture (not specified as infectious) | Genitourinary | 1.00 | 0.97 | 1.04 | 317079 | 3443 | 313636 |
| 495 | Asthma | Respiratory | 1.00 | 0.99 | 1.01 | 320971 | 26779 | 294192 |
| 450 | Noninfectious disorders of lymphatic channels | Circulatory System | 1.01 | 0.94 | 1.09 | 328240 | 717 | 327523 |
| 594.8 | Renal colic | Genitourinary | 1.01 | 0.96 | 1.05 | 322062 | 2131 | 319931 |
| 729.1 | Rheumatism, unspecified and fibrositis | Musculoskeletal | 1.01 | 0.92 | 1.12 | 305341 | 387 | 304954 |
| 292.6 | Hallucinations | Mental Disorders | 0.99 | 0.88 | 1.10 | 321793 | 323 | 321470 |
| 352.1 | Trigeminal nerve disorders [CN5] | Neurological | 0.99 | 0.90 | 1.08 | 313473 | 462 | 313011 |
| 597 | Other disorders of urethra and urinary tract | Genitourinary | 0.99 | 0.93 | 1.06 | 314510 | 874 | 313636 |
| 962.3 | Hormones and synthetic substitutes causing adverse effects in therapeutic use | Injuries & Poisonings | 0.99 | 0.89 | 1.09 | 300575 | 364 | 300211 |
| 272.1 | Hyperlipidemia | Endocrine/Metabolic | 1.00 | 0.97 | 1.02 | 296728 | 5231 | 291497 |
| 716.1 | Unspecified polyarthropathy or polyarthritis | Musculoskeletal | 1.00 | 0.97 | 1.04 | 277426 | 3530 | 273896 |
| 764 | Sciatica | Symptoms | 0.99 | 0.94 | 1.05 | 327944 | 1261 | 326683 |
| 442.1 | Aortic aneurysm | Circulatory System | 0.99 | 0.91 | 1.07 | 319946 | 586 | 319360 |
| 751.2 | Congenital anomalies of urinary system | Congenital Anomalies | 1.01 | 0.94 | 1.08 | 326908 | 905 | 326003 |
| 277.4 | Disorders of bilirubin excretion | Endocrine/Metabolic | 0.99 | 0.89 | 1.09 | 283300 | 373 | 282927 |
| 250.6 | Polyneuropathy in diabetes | Endocrine/Metabolic | 1.01 | 0.92 | 1.12 | 307871 | 381 | 307490 |
| 213 | Benign neoplasm of bone and articular cartilage | Neoplasms | 0.99 | 0.88 | 1.10 | 277528 | 322 | 277206 |
| 626 | Disorders of menstruation and other abnormal bleeding from female genital tract | Genitourinary | 1.00 | 0.97 | 1.04 | 300178 | 3947 | 296231 |
| 642 | Hypertension complicating pregnancy, childbirth, and the puerperium | Pregnancy Complications | 1.01 | 0.95 | 1.07 | 328039 | 989 | 327050 |
| 500.2 | Pneumoconiosis | Respiratory | 0.99 | 0.93 | 1.06 | 317485 | 1001 | 316484 |
| 261.4 | Vitamin D deficiency | Endocrine/Metabolic | 0.99 | 0.90 | 1.09 | 326194 | 408 | 325786 |
| 458 | Hypotension | Circulatory System | 1.01 | 0.95 | 1.07 | 195260 | 1081 | 194179 |
| 165.1 | Cancer of bronchus; lung | Neoplasms | 1.00 | 0.95 | 1.04 | 327450 | 2181 | 325269 |
| 965 | Poisoning by analgesics, antipyretics, and antirheumatics | Injuries & Poisonings | 1.00 | 0.97 | 1.02 | 306293 | 6082 | 300211 |
| 153.2 | Colon cancer | Neoplasms | 1.00 | 0.96 | 1.03 | 304402 | 3122 | 301280 |
| 759 | Other and unspecified congenital anomalies | Congenital Anomalies | 1.01 | 0.94 | 1.09 | 327249 | 679 | 326570 |
| 791 | Gangrene | Symptoms | 1.01 | 0.93 | 1.10 | 328240 | 579 | 327661 |
| 272.11 | Hypercholesterolemia | Endocrine/Metabolic | 1.00 | 0.99 | 1.01 | 325499 | 34002 | 291497 |
| 345.11 | Generalized convulsive epilepsy | Neurological | 1.01 | 0.92 | 1.11 | 286617 | 465 | 286152 |
| 575.9 | Nonspecific abnormal findings on radiological and other examination of biliary tract | Digestive | 0.99 | 0.89 | 1.10 | 310690 | 367 | 310323 |
| 560.4 | Other intestinal obstruction | Digestive | 1.00 | 0.97 | 1.04 | 261839 | 3443 | 258396 |
| 204.12 | Lymphoid leukemia, chronic | Neoplasms | 0.99 | 0.91 | 1.08 | 324030 | 507 | 323523 |
| 172.11 | Melanomas of skin | Neoplasms | 1.00 | 0.97 | 1.04 | 316943 | 2723 | 314220 |

| | | | | | | | | |
|---|---|---|---|---|---|---|---|---|
| 367.8 | Hypermetropia | Sense Organs | 1.01 | 0.89 | 1.15 | 325995 | 241 | 325754 |
| 149.1 | Cancer of oropharynx | Neoplasms | 1.01 | 0.90 | 1.15 | 326297 | 256 | 326041 |
| 907 | Injuries to the nervous system | Injuries & Poisonings | 1.00 | 0.95 | 1.04 | 328240 | 1845 | 326395 |
| 801.1 | Fracture of foot | Injuries & Poisonings | 0.99 | 0.90 | 1.09 | 323975 | 418 | 323557 |
| 960.2 | Allergy/adverse effect of penicillin | Injuries & Poisonings | 1.00 | 0.99 | 1.02 | 316894 | 16683 | 300211 |
| 244.4 | Hypothyroidism NOS | Endocrine/Metabolic | 1.00 | 0.98 | 1.02 | 324759 | 14430 | 310329 |
| 481 | Influenza | Respiratory | 1.00 | 0.98 | 1.02 | 327706 | 10017 | 317689 |
| 433.5 | Cerebral aneurysm | Circulatory System | 1.01 | 0.91 | 1.12 | 318505 | 398 | 318107 |
| 594.2 | Calculus of lower urinary tract | Genitourinary | 0.99 | 0.93 | 1.06 | 320761 | 830 | 319931 |
| 383 | Otosclerosis | Sense Organs | 0.99 | 0.89 | 1.10 | 324473 | 330 | 324143 |
| 965.3 | Salicylates causing adverse effects in therapeutic use | Injuries & Poisonings | 1.01 | 0.91 | 1.11 | 300612 | 401 | 300211 |
| 198.4 | Secondary malignant neoplasm of liver | Neoplasms | 1.00 | 0.96 | 1.04 | 232009 | 2697 | 229312 |
| 276.11 | Hyperosmolality and/or hypernatremia | Endocrine/Metabolic | 1.01 | 0.89 | 1.15 | 320891 | 247 | 320644 |
| 622.1 | Polyp of corpus uteri | Genitourinary | 1.00 | 0.98 | 1.02 | 323490 | 8183 | 315307 |
| 250.22 | Type 2 diabetes with renal manifestations | Endocrine/Metabolic | 1.01 | 0.88 | 1.16 | 307698 | 208 | 307490 |
| 946 | Anaphylactic shock NOS | Injuries & Poisonings | 0.99 | 0.91 | 1.08 | 322450 | 544 | 321906 |
| 835 | Internal derangement of knee | Injuries & Poisonings | 1.00 | 0.97 | 1.03 | 326012 | 4239 | 321773 |
| 451.2 | Phlebitis and thrombophlebitis of lower extremities | Circulatory System | 1.00 | 0.96 | 1.03 | 291357 | 3644 | 287713 |
| 628 | Ovarian cyst | Genitourinary | 1.00 | 0.97 | 1.03 | 301051 | 4820 | 296231 |
| 751.21 | Cystic kidney disease | Congenital Anomalies | 1.01 | 0.92 | 1.11 | 326461 | 458 | 326003 |
| 528.7 | Sialolithiasis | Digestive | 1.01 | 0.90 | 1.13 | 322847 | 313 | 322534 |
| 619.4 | Noninflammatory disorders of vagina | Genitourinary | 1.00 | 0.95 | 1.04 | 280328 | 1780 | 278548 |
| 580.2 | Nephrotic syndrome without mention of glomerulonephritis | Genitourinary | 1.01 | 0.92 | 1.10 | 314575 | 496 | 314079 |
| 281.11 | Pernicious anemia | Hematopoietic | 0.99 | 0.92 | 1.07 | 309650 | 750 | 308900 |
| 594.3 | Calculus of ureter | Genitourinary | 1.00 | 0.96 | 1.04 | 322471 | 2540 | 319931 |
| 323.8 | Encephalitis, non-infectious | Neurological | 1.01 | 0.93 | 1.09 | 327771 | 633 | 327138 |
| 454.1 | Varicose veins of lower extremity | Circulatory System | 1.00 | 0.98 | 1.02 | 299275 | 11562 | 287713 |
| 338.1 | Acute pain | Neurological | 1.01 | 0.92 | 1.11 | 327846 | 420 | 327426 |
| 619 | Noninflammatory female genital disorders | Genitourinary | 1.00 | 0.99 | 1.01 | 328106 | 49558 | 278548 |
| 401.22 | Hypertensive chronic kidney disease | Circulatory System | 1.00 | 0.95 | 1.05 | 250343 | 1595 | 248748 |
| 285.22 | Anemia in neoplastic disease | Hematopoietic | 0.99 | 0.90 | 1.09 | 309337 | 437 | 308900 |
| 555.1 | Regional enteritis | Digestive | 1.00 | 0.95 | 1.04 | 260201 | 1805 | 258396 |
| 385.3 | Cholesteatoma | Sense Organs | 1.01 | 0.93 | 1.09 | 324732 | 589 | 324143 |
| 604 | Disorders of penis | Genitourinary | 1.01 | 0.94 | 1.08 | 308641 | 735 | 307906 |
| 600 | Hyperplasia of prostate | Genitourinary | 1.00 | 0.98 | 1.02 | 318965 | 11572 | 307393 |
| 750.21 | Congenital anomalies of intestine | Congenital Anomalies | 0.99 | 0.87 | 1.13 | 326230 | 227 | 326003 |
| 603 | Other disorders of testis | Genitourinary | 0.99 | 0.93 | 1.06 | 308895 | 989 | 307906 |
| 220 | Benign neoplasm of ovary | Neoplasms | 1.00 | 0.95 | 1.06 | 297546 | 1490 | 296056 |

| | | | | | | | | |
|---|---|---|---|---|---|---|---|---|
| 575.8 | Other disorders of biliary tract | Digestive | 1.01 | 0.95 | 1.07 | 311372 | 1049 | 310323 |
| 502 | Postinflammatory pulmonary fibrosis | Respiratory | 0.99 | 0.93 | 1.06 | 317367 | 883 | 316484 |
| 601.12 | Chronic prostatitis | Genitourinary | 0.99 | 0.93 | 1.06 | 308341 | 948 | 307393 |
| 381.11 | Suppurative and unspecified otitis media | Sense Organs | 1.01 | 0.94 | 1.08 | 324993 | 850 | 324143 |
| 531.2 | Gastric ulcer | Digestive | 1.00 | 0.97 | 1.03 | 324777 | 4228 | 320549 |
| 571.5 | Other chronic nonalcoholic liver disease | Digestive | 1.00 | 0.96 | 1.05 | 320004 | 1673 | 318331 |
| 512 | Other symptoms of respiratory system | Respiratory | 1.00 | 0.97 | 1.02 | 319772 | 5504 | 314268 |
| 157 | Pancreatic cancer | Neoplasms | 1.01 | 0.93 | 1.09 | 312933 | 602 | 312331 |
| 276.41 | Acidosis | Endocrine/Metabolic | 1.00 | 0.94 | 1.06 | 321700 | 1056 | 320644 |
| 341 | Other demyelinating diseases of central nervous system | Neurological | 1.00 | 0.95 | 1.05 | 287764 | 1612 | 286152 |
| 715.2 | Ankylosing spondylitis | Musculoskeletal | 0.99 | 0.90 | 1.10 | 317637 | 384 | 317253 |
| 560.3 | Peritoneal or intestinal adhesions | Digestive | 1.01 | 0.94 | 1.08 | 259257 | 861 | 258396 |
| 81 | Infection/inflammation of internal prosthetic device; implant; and graft | Infectious Diseases | 1.00 | 0.96 | 1.04 | 323993 | 2514 | 321479 |
| 525 | Other diseases of the teeth and supporting structures | Digestive | 1.00 | 0.96 | 1.04 | 314357 | 2699 | 311658 |
| 697 | Sarcoidosis | Dermatologic | 0.99 | 0.91 | 1.08 | 322309 | 560 | 321749 |
| 368.1 | Amblyopia | Sense Organs | 1.01 | 0.92 | 1.10 | 325423 | 541 | 324882 |
| 709.7 | Unspecified diffuse connective tissue disease | Dermatologic | 1.00 | 0.99 | 1.01 | 324635 | 82669 | 241966 |
| 736.2 | Acquired deformities of finger | Musculoskeletal | 0.99 | 0.88 | 1.12 | 316940 | 253 | 316687 |
| 253.2 | Pituitary hypofunction | Endocrine/Metabolic | 1.01 | 0.90 | 1.13 | 324826 | 281 | 324545 |
| 591 | Urinary tract infection | Genitourinary | 1.00 | 0.98 | 1.02 | 309711 | 12676 | 297035 |
| 622 | Polyp of female genital organs | Genitourinary | 1.01 | 0.92 | 1.10 | 315794 | 487 | 315307 |
| 483 | Acute bronchitis and bronchiolitis | Respiratory | 1.01 | 0.88 | 1.16 | 317889 | 200 | 317689 |
| 669 | Complications of labor and delivery NEC | Pregnancy Complications | 1.00 | 0.98 | 1.03 | 328240 | 9534 | 318706 |
| 433.12 | Cerebral atherosclerosis | Circulatory System | 0.99 | 0.87 | 1.13 | 318345 | 238 | 318107 |
| 526.1 | Cysts of the jaws | Digestive | 0.99 | 0.89 | 1.11 | 311993 | 335 | 311658 |
| 454.11 | Varicose veins of lower extremity, symptomtic | Circulatory System | 1.01 | 0.93 | 1.09 | 288365 | 652 | 287713 |
| 726.1 | Enthesopathy | Musculoskeletal | 1.00 | 0.97 | 1.02 | 311657 | 6703 | 304954 |
| 751.11 | Congenital anomalies of female genital organs | Congenital Anomalies | 0.99 | 0.91 | 1.09 | 326445 | 442 | 326003 |
| 957 | Injury to other and unspecified nerves | Injuries & Poisonings | 0.99 | 0.88 | 1.12 | 328157 | 275 | 327882 |
| 800.1 | Fracture of neck of femur | Injuries & Poisonings | 0.99 | 0.87 | 1.13 | 323796 | 239 | 323557 |
| 380.1 | Otitis externa | Sense Organs | 0.99 | 0.91 | 1.08 | 327284 | 532 | 326752 |
| 191.11 | Cancer of brain | Neoplasms | 1.01 | 0.92 | 1.10 | 326945 | 498 | 326447 |
| 221 | Benign neoplasm of other female genital organs | Neoplasms | 0.99 | 0.87 | 1.13 | 295974 | 223 | 295751 |
| 603.1 | Hydrocele | Genitourinary | 1.00 | 0.95 | 1.06 | 309308 | 1402 | 307906 |
| 455 | Hemorrhoids | Circulatory System | 1.00 | 0.99 | 1.01 | 312410 | 24697 | 287713 |
| 560.2 | Impaction of intestine | Digestive | 0.99 | 0.88 | 1.12 | 258667 | 271 | 258396 |
| 530.12 | Ulcer of esophagus | Digestive | 1.00 | 0.97 | 1.03 | 293151 | 5348 | 287803 |
| 522.5 | Periapical abscess | Digestive | 1.00 | 0.95 | 1.06 | 312904 | 1246 | 311658 |

| | | | | | | | | | |
|---|---|---|---|---|---|---|---|---|---|
| 741 | Symptoms and disorders of the joints | Musculoskeletal | 1.00 | 0.99 | 1.02 | 327531 | 16889 | 310642 |
| 41.4 | E. coli | Infectious Diseases | 1.00 | 0.97 | 1.04 | 315432 | 2800 | 312632 |
| 334 | Degenerative disease of the spinal cord | Neurological | 1.00 | 0.95 | 1.06 | 287631 | 1479 | 286152 |
| 281.13 | Folate-deficiency anemia | Hematopoietic | 0.99 | 0.86 | 1.14 | 309101 | 201 | 308900 |
| 202 | Cancer of other lymphoid, histiocytic tissue | Neoplasms | 1.00 | 0.95 | 1.06 | 324661 | 1138 | 323523 |
| 349 | Other and unspecified disorders of the nervous system | Neurological | 1.00 | 0.99 | 1.01 | 326855 | 40703 | 286152 |
| 276.6 | Fluid overload | Endocrine/Metabolic | 1.01 | 0.92 | 1.10 | 321117 | 473 | 320644 |
| 145.2 | Cancer of tongue | Neoplasms | 1.01 | 0.90 | 1.12 | 326366 | 325 | 326041 |
| 149 | Cancer of larynx, pharynx, nasal cavities | Neoplasms | 1.00 | 0.94 | 1.07 | 326986 | 945 | 326041 |
| 316 | Substance addiction and disorders | Mental Disorders | 1.01 | 0.91 | 1.11 | 298042 | 373 | 297669 |
| 523.31 | Acute periodontitis | Digestive | 1.00 | 0.93 | 1.07 | 312371 | 713 | 311658 |
| 614.52 | Vaginitis and vulvovaginitis | Genitourinary | 1.01 | 0.91 | 1.11 | 319219 | 420 | 318799 |
| 635.2 | Antepartum hemorrhage, abruptio placentae, and placenta previa | Pregnancy Complications | 1.00 | 0.93 | 1.06 | 320901 | 936 | 319965 |
| 386 | Vertiginous syndromes and other disorders of vestibular system | Sense Organs | 1.00 | 0.96 | 1.05 | 323761 | 2112 | 321649 |
| 278.1 | Obesity | Endocrine/Metabolic | 1.00 | 0.98 | 1.02 | 328110 | 11143 | 316967 |
| 735.3 | Hallux valgus (Bunion) | Musculoskeletal | 1.00 | 0.97 | 1.02 | 323592 | 6905 | 316687 |
| 727 | Other disorders of synovium, tendon, and bursa | Musculoskeletal | 1.00 | 0.93 | 1.08 | 305708 | 754 | 304954 |
| 601.4 | Balanoposthitis | Genitourinary | 0.99 | 0.89 | 1.11 | 307689 | 296 | 307393 |
| 599.9 | Other abnormality of urination | Genitourinary | 1.00 | 0.96 | 1.05 | 222371 | 1944 | 220427 |
| 428.1 | Congestive heart failure (CHF) NOS | Circulatory System | 1.00 | 0.96 | 1.04 | 324086 | 2053 | 322033 |
| 646 | Other complications of pregnancy NEC | Pregnancy Complications | 1.00 | 0.96 | 1.04 | 328240 | 2396 | 325844 |
| 465 | Acute upper respiratory infections of multiple or unspecified sites | Respiratory | 1.00 | 0.97 | 1.04 | 328232 | 3053 | 325179 |
| 503 | Pulmonary congestion and hypostasis | Respiratory | 1.00 | 0.92 | 1.10 | 316959 | 475 | 316484 |
| 214.1 | Lipoma of skin and subcutaneous tissue | Neoplasms | 1.00 | 0.97 | 1.03 | 325432 | 4756 | 320676 |
| 241.2 | Nontoxic multinodular goiter | Endocrine/Metabolic | 1.00 | 0.93 | 1.07 | 311043 | 714 | 310329 |
| 317.1 | Alcoholism | Mental Disorders | 1.00 | 0.98 | 1.02 | 306366 | 8697 | 297669 |
| 535.1 | Acute gastritis | Digestive | 1.00 | 0.95 | 1.06 | 297704 | 1193 | 296511 |
| 519.9 | Symptoms involving respiratory system and other chest symptoms | Respiratory | 1.00 | 0.89 | 1.11 | 265755 | 308 | 265447 |
| 969 | Poisoning by psychotropic agents | Injuries & Poisonings | 1.00 | 0.96 | 1.05 | 302207 | 1996 | 300211 |
| 473 | Diseases of the larynx and vocal cords | Respiratory | 1.00 | 0.95 | 1.05 | 310564 | 1614 | 308950 |
| 155 | Cancer of liver and intrahepatic bile duct | Neoplasms | 1.00 | 0.88 | 1.13 | 312576 | 245 | 312331 |
| 530.1 | Esophagitis, GERD and related diseases | Digestive | 1.00 | 0.98 | 1.02 | 297342 | 9539 | 287803 |
| 334.2 | Anterior horn cell disease | Neurological | 1.00 | 0.88 | 1.15 | 286376 | 224 | 286152 |
| 361.1 | Retinal detachment with retinal defect | Sense Organs | 1.00 | 0.95 | 1.05 | 318238 | 1456 | 316782 |
| 464 | Acute sinusitis | Respiratory | 1.00 | 0.87 | 1.14 | 325387 | 208 | 325179 |
| 686.1 | Carbuncle and furuncle | Dermatologic | 1.00 | 0.96 | 1.04 | 318829 | 2422 | 316407 |
| 681.7 | Cellulitis and abscess of trunk | Dermatologic | 1.00 | 0.93 | 1.09 | 317030 | 623 | 316407 |
| 700 | Corns and callosities | Dermatologic | 1.00 | 0.89 | 1.12 | 323352 | 288 | 323064 |

| | | | | | | | | |
|---|---|---|---|---|---|---|---|---|
| 707 | Chronic ulcer of skin | Dermatologic | 1.00 | 0.95 | 1.06 | 327959 | 1260 | 326699 |
| 530.5 | Disorders of esophageal motility | Digestive | 1.00 | 0.92 | 1.08 | 288452 | 649 | 287803 |
| 565 | Anal and rectal conditions | Digestive | 1.00 | 0.98 | 1.02 | 255321 | 8661 | 246660 |
| 701 | Other hypertrophic and atrophic conditions of skin | Dermatologic | 1.00 | 0.96 | 1.05 | 324891 | 1827 | 323064 |
| 529.1 | Glossitis | Digestive | 1.00 | 0.89 | 1.12 | 322838 | 304 | 322534 |
| 204.21 | Myeloid leukemia, acute | Neoplasms | 1.00 | 0.90 | 1.12 | 323842 | 319 | 323523 |
| 723 | Other disorders of cervical region | Musculoskeletal | 1.00 | 0.91 | 1.11 | 311345 | 401 | 310944 |
| 38.1 | Gram negative septicemia | Infectious Diseases | 1.00 | 0.94 | 1.07 | 313487 | 855 | 312632 |
| 798 | Malaise and fatigue | Symptoms | 1.00 | 0.97 | 1.04 | 327695 | 2966 | 324729 |
| 516.1 | Hemoptysis | Respiratory | 1.00 | 0.96 | 1.04 | 328154 | 2103 | 326051 |
| 599.4 | Urinary incontinence | Genitourinary | 1.00 | 0.98 | 1.02 | 229423 | 8996 | 220427 |
| 479 | Other upper respiratory disease | Respiratory | 1.00 | 0.98 | 1.02 | 324241 | 15291 | 308950 |
| 540.1 | Appendicitis | Digestive | 1.00 | 0.93 | 1.07 | 325174 | 795 | 324379 |
| 244.1 | Secondary hypothyroidism | Endocrine/Metabolic | 1.00 | 0.94 | 1.06 | 311484 | 1155 | 310329 |
| 212 | Benign neoplasm of respiratory and intrathoracic organs | Neoplasms | 1.00 | 0.90 | 1.11 | 328240 | 355 | 327885 |
| 803.3 | Fracture of clavicle or scapula | Injuries & Poisonings | 1.00 | 0.89 | 1.12 | 323857 | 300 | 323557 |
| 263 | Other nutritional deficiency | Endocrine/Metabolic | 1.00 | 0.95 | 1.05 | 327337 | 1551 | 325786 |
| 724.1 | Disorders of sacrum | Musculoskeletal | 1.00 | 0.89 | 1.12 | 311225 | 281 | 310944 |
| 686 | Other local infections of skin and subcutaneous tissue | Dermatologic | 1.00 | 0.98 | 1.02 | 327410 | 11003 | 316407 |
| 384 | Other disorders of tympanic membrane | Sense Organs | 1.00 | 0.91 | 1.10 | 324573 | 430 | 324143 |
| 612.2 | Hypertrophy of breast (Gynecomastia) | Genitourinary | 1.00 | 0.94 | 1.07 | 321732 | 859 | 320873 |
| 38.2 | Gram positive septicemia | Infectious Diseases | 1.00 | 0.92 | 1.09 | 313123 | 491 | 312632 |
| 724.2 | Disorders of coccyx | Musculoskeletal | 1.00 | 0.89 | 1.13 | 311226 | 282 | 310944 |
| 716.9 | Arthropathy NOS | Musculoskeletal | 1.00 | 0.99 | 1.01 | 327719 | 53823 | 273896 |
| 540 | Appendiceal conditions | Digestive | 1.00 | 0.97 | 1.03 | 328240 | 3861 | 324379 |
| 335 | Multiple sclerosis | Neurological | 1.00 | 0.95 | 1.06 | 287531 | 1379 | 286152 |
| 426.31 | Right bundle branch block | Circulatory System | 1.00 | 0.95 | 1.05 | 301094 | 1573 | 299521 |
| 698 | Pruritus and related conditions | Dermatologic | 1.00 | 0.93 | 1.07 | 328240 | 799 | 327441 |
| 165 | Cancer within the respiratory system | Neoplasms | 1.00 | 0.96 | 1.04 | 328223 | 2954 | 325269 |
| 427.12 | Paroxysmal ventricular tachycardia | Circulatory System | 1.00 | 0.94 | 1.06 | 300490 | 969 | 299521 |
| 610.3 | Fibrosclerosis of breast | Genitourinary | 1.00 | 0.89 | 1.12 | 321176 | 303 | 320873 |
| 441 | Vascular insufficiency of intestine | Circulatory System | 1.00 | 0.89 | 1.12 | 319657 | 297 | 319360 |
| 318 | Tobacco use disorder | Mental Disorders | 1.00 | 0.99 | 1.01 | 318172 | 20503 | 297669 |
| 626.2 | Dysmenorrhea | Genitourinary | 1.00 | 0.95 | 1.05 | 297910 | 1679 | 296231 |
| 587 | Kidney replaced by transpant | Genitourinary | 1.00 | 0.91 | 1.10 | 314477 | 398 | 314079 |
| 261.2 | Vitamin B-complex deficiencies | Endocrine/Metabolic | 1.00 | 0.93 | 1.07 | 326559 | 773 | 325786 |
| 624.9 | stress incontinence, female | Genitourinary | 1.00 | 0.97 | 1.03 | 324769 | 6100 | 318669 |
| 714 | Rheumatoid arthritis and other inflammatory polyarthropathies | Musculoskeletal | 1.00 | 0.98 | 1.02 | 325905 | 8652 | 317253 |

| Supplementary Table 16. Phenome-wide association study (PheWAS) results for the calcium channel blocker (CCB) genetic risk score after excluding potentially pleiotropic single-nucleotide polymorphisms identified by their secondary associations in PhenoScanner. | | | | | | | | |
|---|---|---|---|---|---|---|---|---|
| Phecode | Trait | Category | OR | Low 95% CI | Upp 95% CI | Total sample size | Cases | Controls |
| 401.1 | Essential hypertension | Circulatory System | 0.95 | 0.94 | 0.96 | 327983 | 79235 | 248748 |
| 401 | Hypertension | Circulatory System | 0.95 | 0.94 | 0.96 | 328239 | 79491 | 248748 |
| 459.9 | Circulatory disease NEC | Circulatory System | 0.97 | 0.96 | 0.98 | 327928 | 133749 | 194179 |
| 411 | Ischemic Heart Disease | Circulatory System | 0.97 | 0.96 | 0.99 | 327032 | 31343 | 295689 |
| 411.4 | Coronary atherosclerosis | Circulatory System | 0.97 | 0.96 | 0.99 | 327242 | 31553 | 295689 |
| 411.8 | Other chronic ischemic heart disease, unspecified | Circulatory System | 0.97 | 0.96 | 0.99 | 326969 | 31280 | 295689 |
| 411.2 | Myocardial infarction | Circulatory System | 0.96 | 0.95 | 0.98 | 307658 | 11969 | 295689 |
| 427.2 | Atrial fibrillation and flutter | Circulatory System | 0.97 | 0.95 | 0.98 | 314573 | 15052 | 299521 |
| 562.1 | Diverticulosis | Digestive | 1.02 | 1.01 | 1.04 | 286281 | 27885 | 258396 |
| 411.41 | Aneurysm and dissection of heart | Circulatory System | 0.87 | 0.81 | 0.94 | 296410 | 721 | 295689 |
| 411.3 | Angina pectoris | Circulatory System | 0.97 | 0.96 | 0.99 | 312048 | 16359 | 295689 |
| 716.2 | Unspecified monoarthritis | Musculoskeletal | 1.02 | 1.01 | 1.04 | 289797 | 15901 | 273896 |
| 416 | Cardiomegaly | Circulatory System | 0.94 | 0.91 | 0.98 | 324157 | 2610 | 321547 |
| 395.1 | Nonrheumatic mitral valve disorders | Circulatory System | 0.95 | 0.92 | 0.99 | 324497 | 2969 | 321528 |
| 428.1 | Congestive heart failure (CHF) NOS | Circulatory System | 0.94 | 0.90 | 0.98 | 324086 | 2053 | 322033 |
| 751.11 | Congenital anomalies of female genital organs | Congenital Anomalies | 0.88 | 0.80 | 0.97 | 326445 | 442 | 326003 |
| 686.3 | Pilonidal cyst | Dermatologic | 1.11 | 1.03 | 1.20 | 317024 | 617 | 316407 |
| 38.2 | Gram positive septicemia | Infectious Diseases | 0.89 | 0.81 | 0.97 | 313123 | 491 | 312632 |
| 977 | Personal history of allergy to medicinal agents | Injuries & Poisonings | 0.84 | 0.74 | 0.96 | 300449 | 238 | 300211 |
| 503 | Pulmonary congestion and hypostasis | Respiratory | 0.89 | 0.81 | 0.97 | 316959 | 475 | 316484 |
| 428.2 | Heart failure NOS | Circulatory System | 0.96 | 0.93 | 0.99 | 326367 | 4334 | 322033 |
| 240 | Simple and unspecified goiter | Endocrine/Metabolic | 0.90 | 0.83 | 0.98 | 310951 | 622 | 310329 |
| 946 | Anaphylactic shock NOS | Injuries & Poisonings | 0.90 | 0.82 | 0.98 | 322450 | 544 | 321906 |
| 476 | Allergic rhinitis | Respiratory | 1.08 | 1.01 | 1.14 | 310025 | 1075 | 308950 |
| 333.1 | Essential tremor | Neurological | 0.85 | 0.74 | 0.97 | 286370 | 218 | 286152 |
| 352.2 | Facial nerve disorders [CN7] | Neurological | 0.92 | 0.86 | 0.98 | 313911 | 900 | 313011 |
| 634.1 | Missed abortion/Hydatidiform mole | Pregnancy Complications | 1.07 | 1.01 | 1.14 | 321168 | 1203 | 319965 |
| 200.1 | Polycythemia vera | Neoplasms | 0.89 | 0.80 | 0.98 | 318136 | 404 | 317732 |
| 574 | Cholelithiasis and cholecystitis | Digestive | 0.89 | 0.81 | 0.98 | 310726 | 403 | 310323 |
| 741.4 | Joint effusions | Musculoskeletal | 0.87 | 0.77 | 0.98 | 310925 | 283 | 310642 |
| 220 | Benign neoplasm of ovary | Neoplasms | 1.06 | 1.01 | 1.12 | 297546 | 1490 | 296056 |
| 394.2 | Mitral valve disease | Circulatory System | 0.96 | 0.93 | 0.99 | 324603 | 3075 | 321528 |
| 411.9 | Other acute and subacute forms of ischemic heart disease | Circulatory System | 0.94 | 0.88 | 0.99 | 296883 | 1194 | 295689 |
| 381.11 | Suppurative and unspecified otitis media | Sense Organs | 0.92 | 0.86 | 0.99 | 324993 | 850 | 324143 |
| 375 | Disorders of lacrimal system | Sense Organs | 1.06 | 1.01 | 1.11 | 289852 | 1660 | 288192 |

| | | | | | | | | |
|---|---|---|---|---|---|---|---|---|
| 555 | Inflammatory bowel disease and other gastroenteritis and colitis | Digestive | 1.02 | 1.00 | 1.03 | 278195 | 19799 | 258396 |
| 574.12 | Cholelithiasis with other cholecystitis | Digestive | 0.97 | 0.95 | 1.00 | 315816 | 5493 | 310323 |
| 426.24 | Atrioventricular block, complete | Circulatory System | 0.92 | 0.85 | 1.00 | 300112 | 591 | 299521 |
| 751.1 | Congenital anomalies of genital organs | Congenital Anomalies | 0.92 | 0.86 | 1.00 | 326709 | 706 | 326003 |
| 378.5 | Paralytic strabismus | Sense Organs | 0.88 | 0.79 | 0.99 | 288479 | 287 | 288192 |
| 558 | Noninfectious gastroenteritis | Digestive | 1.02 | 1.00 | 1.03 | 278260 | 19864 | 258396 |
| 564.9 | Personal history of diseases of digestive system | Digestive | 1.02 | 1.00 | 1.03 | 274320 | 15924 | 258396 |
| 513.4 | Hyperventilation | Respiratory | 1.13 | 1.00 | 1.27 | 327904 | 280 | 327624 |
| 560.4 | Other intestinal obstruction | Digestive | 1.04 | 1.00 | 1.07 | 261839 | 3443 | 258396 |
| 850 | Hemorrhage or hematoma complicating a procedure | Injuries & Poisonings | 0.97 | 0.95 | 1.00 | 319328 | 5364 | 313964 |
| 304 | Adjustment reaction | Mental Disorders | 1.12 | 1.00 | 1.25 | 283221 | 323 | 282898 |
| 574.3 | Cholecystitis without cholelithiasis | Digestive | 1.04 | 1.00 | 1.08 | 313141 | 2818 | 310323 |
| 250 | Diabetes mellitus | Endocrine/Metabolic | 1.01 | 1.00 | 1.03 | 327730 | 20240 | 307490 |
| 170.2 | Cancer of connective tissue | Neoplasms | 0.93 | 0.86 | 1.00 | 328122 | 734 | 327388 |
| 286.7 | Other and unspecified coagulation defects | Hematopoietic | 1.10 | 1.00 | 1.22 | 325902 | 405 | 325497 |
| 365 | Glaucoma | Sense Organs | 1.03 | 1.00 | 1.06 | 321340 | 4558 | 316782 |
| 250.2 | Type 2 diabetes | Endocrine/Metabolic | 1.02 | 1.00 | 1.03 | 326603 | 19113 | 307490 |
| 380.4 | Impacted cerumen | Sense Organs | 0.90 | 0.81 | 1.00 | 327106 | 354 | 326752 |
| 415.21 | Primary pulmonary hypertension | Circulatory System | 1.10 | 1.00 | 1.20 | 322013 | 466 | 321547 |
| 429.3 | Symptoms involving cardiovascular system | Circulatory System | 0.87 | 0.76 | 1.00 | 322241 | 208 | 322033 |
| 335 | Multiple sclerosis | Neurological | 1.05 | 1.00 | 1.11 | 287531 | 1379 | 286152 |
| 960 | Poisoning by antibiotics | Injuries & Poisonings | 0.97 | 0.94 | 1.00 | 303739 | 3528 | 300211 |
| 560.3 | Peritoneal or intestinal adhesions | Digestive | 1.07 | 1.00 | 1.14 | 259257 | 861 | 258396 |
| 478 | Throat pain | Respiratory | 0.90 | 0.82 | 1.00 | 309337 | 387 | 308950 |
| 414 | Other forms of chronic heart disease | Circulatory System | 0.95 | 0.91 | 1.00 | 297457 | 1768 | 295689 |
| 375.2 | Epiphora | Sense Organs | 1.07 | 1.00 | 1.14 | 289094 | 902 | 288192 |
| 750 | Digestive congenital anomalies | Congenital Anomalies | 0.93 | 0.86 | 1.00 | 326705 | 702 | 326003 |
| 601.11 | Acute prostatitis | Genitourinary | 1.13 | 1.00 | 1.27 | 307653 | 260 | 307393 |
| 989 | Toxic effect of other substances, chiefly nonmedicinal as to source | Injuries & Poisonings | 1.05 | 1.00 | 1.11 | 328136 | 1438 | 326698 |
| 250.41 | Impaired fasting glucose | Endocrine/Metabolic | 1.12 | 1.00 | 1.27 | 307758 | 268 | 307490 |
| 442.8 | Aneurysm of other specified artery | Circulatory System | 0.88 | 0.76 | 1.00 | 319570 | 210 | 319360 |
| 681 | Superficial cellulitis and abscess | Dermatologic | 0.93 | 0.87 | 1.00 | 317135 | 728 | 316407 |
| 735 | Acquired foot deformities | Musculoskeletal | 1.11 | 1.00 | 1.25 | 316986 | 299 | 316687 |
| 388 | Other disorders of ear | Sense Organs | 0.98 | 0.96 | 1.00 | 328080 | 9886 | 318194 |
| 726 | Peripheral enthesopathies and allied syndromes | Musculoskeletal | 1.02 | 1.00 | 1.04 | 313722 | 8768 | 304954 |
| 530.12 | Ulcer of esophagus | Digestive | 0.97 | 0.95 | 1.00 | 293151 | 5348 | 287803 |
| 594.3 | Calculus of ureter | Genitourinary | 0.96 | 0.93 | 1.00 | 322471 | 2540 | 319931 |
| 215 | Other benign neoplasm of connective and other soft tissue | Neoplasms | 1.06 | 1.00 | 1.12 | 321804 | 1128 | 320676 |

| | | | | | | | | |
|---|---|---|---|---|---|---|---|---|
| 507 | Pleurisy; pleural effusion | Respiratory | 0.98 | 0.95 | 1.00 | 323091 | 6607 | 316484 |
| 642.1 | Preeclampsia and eclampsia | Pregnancy Complications | 0.90 | 0.81 | 1.01 | 327385 | 335 | 327050 |
| 452 | Other venous embolism and thrombosis | Circulatory System | 0.92 | 0.85 | 1.01 | 288267 | 554 | 287713 |
| 745 | Pain in joint | Musculoskeletal | 0.96 | 0.92 | 1.00 | 328240 | 1940 | 326300 |
| 800.1 | Fracture of neck of femur | Injuries & Poisonings | 0.89 | 0.78 | 1.01 | 323796 | 239 | 323557 |
| 380 | Disorders of external ear | Sense Organs | 0.95 | 0.91 | 1.00 | 328227 | 1475 | 326752 |
| 70.9 | Hepatitis NOS | Infectious Diseases | 1.09 | 0.99 | 1.19 | 322658 | 452 | 322206 |
| 736.2 | Acquired deformities of finger | Musculoskeletal | 0.89 | 0.79 | 1.01 | 316940 | 253 | 316687 |
| 250.42 | Other abnormal glucose | Endocrine/Metabolic | 0.92 | 0.83 | 1.01 | 307927 | 437 | 307490 |
| 716.9 | Arthropathy NOS | Musculoskeletal | 1.01 | 1.00 | 1.02 | 327719 | 53823 | 273896 |
| 180.1 | Cervical cancer | Neoplasms | 0.97 | 0.95 | 1.00 | 302137 | 4554 | 297583 |
| 277 | Other disorders of metabolism | Endocrine/Metabolic | 0.99 | 0.98 | 1.00 | 328230 | 45303 | 282927 |
| 350.2 | Abnormality of gait | Neurological | 0.96 | 0.91 | 1.00 | 327077 | 1633 | 325444 |
| 709.7 | Unspecified diffuse connective tissue disease | Dermatologic | 1.01 | 1.00 | 1.02 | 324635 | 82669 | 241966 |
| 389.2 | Conductive hearing loss | Sense Organs | 0.92 | 0.84 | 1.01 | 318629 | 435 | 318194 |
| 360.2 | Progressive myopia | Sense Organs | 1.11 | 0.99 | 1.26 | 317032 | 250 | 316782 |
| 379.2 | Disorders of vitreous body | Sense Organs | 1.05 | 0.99 | 1.10 | 289599 | 1407 | 288192 |
| 458.9 | Hypotension NOS | Circulatory System | 0.97 | 0.94 | 1.00 | 197817 | 3638 | 194179 |
| 341 | Other demyelinating diseases of central nervous system | Neurological | 1.04 | 0.99 | 1.10 | 287764 | 1612 | 286152 |
| 601.1 | Prostatitis | Genitourinary | 1.07 | 0.99 | 1.15 | 308058 | 665 | 307393 |
| 272.1 | Hyperlipidemia | Endocrine/Metabolic | 0.98 | 0.95 | 1.00 | 296728 | 5231 | 291497 |
| 371 | Inflammation of the eye | Sense Organs | 0.92 | 0.83 | 1.01 | 318796 | 408 | 318388 |
| 333 | Extrapyramidal disease and abnormal movement disorders | Neurological | 0.96 | 0.92 | 1.01 | 288187 | 2035 | 286152 |
| 202.24 | Large cell lymphoma | Neoplasms | 1.07 | 0.99 | 1.16 | 324112 | 589 | 323523 |
| 244.1 | Secondary hypothyroidism | Endocrine/Metabolic | 0.95 | 0.90 | 1.01 | 311484 | 1155 | 310329 |
| 368.1 | Amblyopia | Sense Organs | 0.93 | 0.85 | 1.01 | 325423 | 541 | 324882 |
| 425.1 | Primary/intrinsic cardiomyopathies | Circulatory System | 0.95 | 0.89 | 1.01 | 326036 | 1053 | 324983 |
| 798 | Malaise and fatigue | Symptoms | 1.03 | 0.99 | 1.07 | 327695 | 2966 | 324729 |
| 857 | Mechanical complication of unspecified genitourinary device, implant, and graft | Injuries & Poisonings | 0.95 | 0.90 | 1.01 | 315248 | 1284 | 313964 |
| 907 | Injuries to the nervous system | Injuries & Poisonings | 0.96 | 0.92 | 1.01 | 328240 | 1845 | 326395 |
| 642 | Hypertension complicating pregnancy, childbirth, and the puerperium | Pregnancy Complications | 0.95 | 0.89 | 1.01 | 328039 | 989 | 327050 |
| 700 | Corns and callosities | Dermatologic | 0.91 | 0.81 | 1.02 | 323352 | 288 | 323064 |
| 681.2 | Cellulitis and abscess of face/neck | Dermatologic | 0.93 | 0.85 | 1.01 | 316936 | 529 | 316407 |
| 601.12 | Chronic prostatitis | Genitourinary | 1.05 | 0.99 | 1.12 | 308341 | 948 | 307393 |
| 473 | Diseases of the larynx and vocal cords | Respiratory | 0.96 | 0.91 | 1.01 | 310564 | 1614 | 308950 |
| 433.12 | Cerebral atherosclerosis | Circulatory System | 1.11 | 0.98 | 1.26 | 318345 | 238 | 318107 |
| 703 | Diseases of nail, NOS | Dermatologic | 0.92 | 0.83 | 1.02 | 321795 | 366 | 321429 |
| 528.6 | Leukoplakia of oral mucosa | Digestive | 0.91 | 0.81 | 1.02 | 322846 | 312 | 322534 |

| | | | | | | | | |
|---|---|---|---|---|---|---|---|---|
| 324 | Other CNS infection and poliomyelitis | Neurological | 1.10 | 0.98 | 1.23 | 327426 | 288 | 327138 |
| 528 | Diseases of the oral soft tissues, excluding lesions specific for gingiva and tongue | Digestive | 0.96 | 0.92 | 1.01 | 324593 | 2059 | 322534 |
| 510 | Other diseases of lung | Respiratory | 0.94 | 0.88 | 1.01 | 328200 | 770 | 327430 |
| 451.2 | Phlebitis and thrombophlebitis of lower extremities | Circulatory System | 1.03 | 0.99 | 1.06 | 291357 | 3644 | 287713 |
| 187.2 | Malignant neoplasm of testis | Neoplasms | 0.98 | 0.95 | 1.01 | 323790 | 4572 | 319218 |
| 184.11 | Malignant neoplasm of ovary | Neoplasms | 0.98 | 0.95 | 1.01 | 310562 | 5009 | 305553 |
| 636 | Early or threatened labor; hemorrhage in early pregnancy | Pregnancy Complications | 1.05 | 0.99 | 1.11 | 321227 | 1262 | 319965 |
| 426.31 | Right bundle branch block | Circulatory System | 0.96 | 0.91 | 1.01 | 301094 | 1573 | 299521 |
| 961.1 | Poisoning/allergy of sulfonamides | Injuries & Poisonings | 0.95 | 0.89 | 1.01 | 301101 | 890 | 300211 |
| 334 | Degenerative disease of the spinal cord | Neurological | 1.04 | 0.99 | 1.10 | 287631 | 1479 | 286152 |
| 151 | Cancer of stomach | Neoplasms | 1.07 | 0.98 | 1.16 | 312905 | 574 | 312331 |
| 157 | Pancreatic cancer | Neoplasms | 1.07 | 0.98 | 1.15 | 312933 | 602 | 312331 |
| 198.6 | Secondary malignancy of bone | Neoplasms | 1.03 | 0.99 | 1.08 | 231505 | 2193 | 229312 |
| 916 | Contusion | Injuries & Poisonings | 0.96 | 0.91 | 1.01 | 328240 | 1478 | 326762 |
| 465 | Acute upper respiratory infections of multiple or unspecified sites | Respiratory | 0.97 | 0.94 | 1.01 | 328232 | 3053 | 325179 |
| 221 | Benign neoplasm of other female genital organs | Neoplasms | 0.90 | 0.79 | 1.03 | 295974 | 223 | 295751 |
| 480.5 | Bronchopneumonia and lung abscess | Respiratory | 0.92 | 0.84 | 1.02 | 318077 | 388 | 317689 |
| 530.2 | Esophageal bleeding (varices/hemorrhage) | Digestive | 1.04 | 0.99 | 1.09 | 289525 | 1722 | 287803 |
| 191.11 | Cancer of brain | Neoplasms | 0.93 | 0.85 | 1.02 | 326945 | 498 | 326447 |
| 525 | Other diseases of the teeth and supporting structures | Digestive | 1.03 | 0.99 | 1.07 | 314357 | 2699 | 311658 |
| 345.12 | Partial epilepsy | Neurological | 0.91 | 0.81 | 1.03 | 286450 | 298 | 286152 |
| 702.2 | Seborrheic keratosis | Dermatologic | 1.03 | 0.99 | 1.06 | 325769 | 3169 | 322600 |
| 614.54 | Abscess or ulceration of vulva | Genitourinary | 1.09 | 0.97 | 1.23 | 319079 | 280 | 318799 |
| 593 | Hematuria | Genitourinary | 1.01 | 1.00 | 1.03 | 313795 | 16760 | 297035 |
| 292.2 | Mild cognitive impairment | Mental Disorders | 1.11 | 0.97 | 1.28 | 321672 | 202 | 321470 |
| 573.3 | Hepatomegaly | Digestive | 1.09 | 0.98 | 1.22 | 318641 | 310 | 318331 |
| 474 | Acute and chronic tonsillitis | Respiratory | 1.06 | 0.98 | 1.15 | 309554 | 604 | 308950 |
| 477 | Epistaxis or throat hemorrhage | Respiratory | 0.97 | 0.93 | 1.01 | 311469 | 2519 | 308950 |
| 578.8 | Hemorrhage of rectum and anus | Digestive | 0.99 | 0.97 | 1.00 | 317220 | 13826 | 303394 |
| 418.1 | Precordial pain | Circulatory System | 0.98 | 0.94 | 1.01 | 299506 | 3686 | 295820 |
| 378.1 | Strabismus (not specified as paralytic) | Sense Organs | 1.05 | 0.99 | 1.12 | 289139 | 947 | 288192 |
| 371.1 | Uveitis, noninfectious or NOS | Sense Organs | 1.07 | 0.98 | 1.17 | 318886 | 498 | 318388 |
| 149.4 | Cancer of larynx | Neoplasms | 0.91 | 0.81 | 1.03 | 326307 | 266 | 326041 |
| 722.1 | Displacement of intervertebral disc | Musculoskeletal | 1.07 | 0.98 | 1.16 | 311460 | 516 | 310944 |
| 395.6 | Heart valve replaced | Circulatory System | 0.96 | 0.91 | 1.01 | 323056 | 1528 | 321528 |
| 722.6 | Degeneration of intervertebral disc | Musculoskeletal | 1.03 | 0.99 | 1.07 | 313866 | 2922 | 310944 |
| 480.11 | Pneumococcal pneumonia | Respiratory | 1.02 | 0.99 | 1.05 | 323680 | 5991 | 317689 |
| 41 | Bacterial infection NOS | Infectious Diseases | 0.99 | 0.97 | 1.00 | 323384 | 10752 | 312632 |

| | | | | | | | | |
|---|---|---|---|---|---|---|---|---|
| 426.3 | Bundle branch block | Circulatory System | 1.09 | 0.97 | 1.22 | 299812 | 291 | 299521 |
| 962.3 | Hormones and synthetic substitutes causing adverse effects in therapeutic use | Injuries & Poisonings | 1.08 | 0.97 | 1.20 | 300575 | 364 | 300211 |
| 729 | Other disorders of soft tissues | Musculoskeletal | 1.01 | 1.00 | 1.02 | 327478 | 22524 | 304954 |
| 717 | Polymyalgia Rheumatica | Musculoskeletal | 1.04 | 0.99 | 1.11 | 328240 | 1147 | 327093 |
| 394 | Rheumatic disease of the heart valves | Circulatory System | 0.95 | 0.89 | 1.02 | 322471 | 943 | 321528 |
| 444.1 | Arterial embolism and thrombosis of lower extremity artery | Circulatory System | 1.06 | 0.98 | 1.15 | 319930 | 570 | 319360 |
| 619.3 | Noninflammatory disorders of cervix | Genitourinary | 0.97 | 0.94 | 1.01 | 281393 | 2845 | 278548 |
| 728.71 | Contracture of palmar fascia [Dupuytren's disease] | Musculoskeletal | 0.97 | 0.93 | 1.01 | 307403 | 2449 | 304954 |
| 475 | Chronic sinusitis | Respiratory | 1.03 | 0.99 | 1.07 | 311580 | 2630 | 308950 |
| 280.2 | Iron deficiency anemia secondary to blood loss (chronic) | Hematopoietic | 1.07 | 0.98 | 1.17 | 309376 | 476 | 308900 |
| 722.9 | Other and unspecified disc disorder | Musculoskeletal | 1.02 | 0.99 | 1.06 | 314894 | 3950 | 310944 |
| 250.24 | Type 2 diabetes with neurological manifestations | Endocrine/Metabolic | 0.94 | 0.87 | 1.02 | 308102 | 612 | 307490 |
| 979 | Adverse drug events and drug allergies | Injuries & Poisonings | 0.95 | 0.88 | 1.02 | 300927 | 716 | 300211 |
| 441 | Vascular insufficiency of intestine | Circulatory System | 0.92 | 0.82 | 1.03 | 319657 | 297 | 319360 |
| 174.11 | Malignant neoplasm of female breast | Neoplasms | 0.99 | 0.97 | 1.01 | 316838 | 12414 | 304424 |
| 367.2 | Astigmatism | Sense Organs | 1.10 | 0.96 | 1.26 | 325964 | 210 | 325754 |
| 442.1 | Aortic aneurysm | Circulatory System | 0.94 | 0.87 | 1.02 | 319946 | 586 | 319360 |
| 627.4 | Premenopausal menorrhagia | Genitourinary | 0.92 | 0.83 | 1.03 | 296563 | 332 | 296231 |
| 386.9 | Dizziness and giddiness (Light-headedness and vertigo) | Sense Organs | 0.98 | 0.95 | 1.01 | 326388 | 4739 | 321649 |
| 696.42 | Psoriatic arthropathy | Dermatologic | 1.05 | 0.98 | 1.13 | 314672 | 740 | 313932 |
| 374 | Other disorders of eyelids | Sense Organs | 0.98 | 0.94 | 1.01 | 321593 | 3205 | 318388 |
| 573 | Other disorders of liver | Digestive | 1.02 | 0.99 | 1.05 | 323533 | 5202 | 318331 |
| 939 | Atopic/contact dermatitis due to other or unspecified | Dermatologic | 1.03 | 0.99 | 1.08 | 324060 | 2154 | 321906 |
| 8.6 | Viral Enteritis | Infectious Diseases | 0.95 | 0.89 | 1.02 | 319924 | 845 | 319079 |
| 211 | Benign neoplasm of other parts of digestive system | Neoplasms | 1.02 | 0.99 | 1.05 | 319683 | 5375 | 314308 |
| 969 | Poisoning by psychotropic agents | Injuries & Poisonings | 1.03 | 0.99 | 1.08 | 302207 | 1996 | 300211 |
| 306 | Other mental disorder | Mental Disorders | 0.99 | 0.98 | 1.00 | 312303 | 29405 | 282898 |
| 255.21 | Glucocorticoid deficiency | Endocrine/Metabolic | 1.07 | 0.97 | 1.19 | 324917 | 372 | 324545 |
| 458 | Hypotension | Circulatory System | 0.96 | 0.90 | 1.02 | 195260 | 1081 | 194179 |
| 389 | Hearing loss | Sense Organs | 0.98 | 0.94 | 1.01 | 321364 | 3170 | 318194 |
| 473.3 | Paralysis/spasm of vocal cords or larynx | Respiratory | 0.92 | 0.81 | 1.04 | 309217 | 267 | 308950 |
| 202.21 | Nodular lymphoma | Neoplasms | 1.07 | 0.97 | 1.18 | 323913 | 390 | 323523 |
| 508 | Pulmonary collapse; interstitial and compensatory emphysema | Respiratory | 1.03 | 0.99 | 1.08 | 318570 | 2086 | 316484 |
| 550.2 | Diaphragmatic hernia | Digestive | 1.01 | 1.00 | 1.02 | 307453 | 27664 | 279789 |
| 8.5 | Bacterial enteritis | Infectious Diseases | 1.03 | 0.99 | 1.08 | 321188 | 2109 | 319079 |
| 276.5 | Hypovolemia | Endocrine/Metabolic | 1.03 | 0.99 | 1.06 | 323539 | 2895 | 320644 |
| 374.1 | Ectropion or entropion | Sense Organs | 1.04 | 0.98 | 1.11 | 319480 | 1092 | 318388 |
| 427.12 | Paroxysmal ventricular tachycardia | Circulatory System | 1.04 | 0.98 | 1.11 | 300490 | 969 | 299521 |

| | | | | | | | | | |
|---|---|---|---|---|---|---|---|---|---|
| 440.9 | Atherosclerosis of aorta | Circulatory System | 0.91 | 0.79 | 1.05 | 319562 | 202 | 319360 |
| 217.1 | Nevus, non-neoplastic | Neoplasms | 0.95 | 0.87 | 1.03 | 320346 | 597 | 319749 |
| 41.4 | E. coli | Infectious Diseases | 0.98 | 0.94 | 1.01 | 315432 | 2800 | 312632 |
| 519 | Other diseases of respiratory system, not elsewhere classified | Respiratory | 0.99 | 0.99 | 1.00 | 327615 | 62168 | 265447 |
| 727.5 | Rupture of synovium | Musculoskeletal | 1.07 | 0.97 | 1.19 | 305298 | 344 | 304954 |
| 859 | Complication due to other implant and internal device | Injuries & Poisonings | 0.98 | 0.94 | 1.01 | 317302 | 3338 | 313964 |
| 681.7 | Cellulitis and abscess of trunk | Dermatologic | 1.05 | 0.97 | 1.14 | 317030 | 623 | 316407 |
| 695.3 | Rosacea | Dermatologic | 0.93 | 0.83 | 1.04 | 322068 | 319 | 321749 |
| 571.5 | Other chronic nonalcoholic liver disease | Digestive | 1.03 | 0.98 | 1.08 | 320004 | 1673 | 318331 |
| 361 | Retinal detachments and defects | Sense Organs | 1.03 | 0.99 | 1.07 | 319011 | 2229 | 316782 |
| 509.8 | Dependence on respirator [Ventilator] or supplemental oxygen | Respiratory | 1.07 | 0.97 | 1.18 | 316885 | 401 | 316484 |
| 331 | Other cerebral degenerations | Neurological | 1.09 | 0.96 | 1.23 | 286392 | 240 | 286152 |
| 497 | Bronchitis | Respiratory | 1.05 | 0.97 | 1.14 | 294834 | 642 | 294192 |
| 726.3 | Bursitis | Musculoskeletal | 1.06 | 0.97 | 1.15 | 305497 | 543 | 304954 |
| 281.13 | Folate-deficiency anemia | Hematopoietic | 1.09 | 0.95 | 1.26 | 309101 | 201 | 308900 |
| 578 | Gastrointestinal hemorrhage | Digestive | 1.08 | 0.96 | 1.21 | 303679 | 285 | 303394 |
| 626.1 | Irregular menstrual cycle/bleeding | Genitourinary | 1.02 | 0.99 | 1.06 | 299824 | 3593 | 296231 |
| 689 | Disorder of skin and subcutaneous tissue NOS | Dermatologic | 1.01 | 1.00 | 1.02 | 328240 | 42194 | 286046 |
| 193 | Thyroid cancer | Neoplasms | 0.93 | 0.84 | 1.04 | 327009 | 367 | 326642 |
| 242 | Thyrotoxicosis with or without goiter | Endocrine/Metabolic | 0.97 | 0.92 | 1.02 | 311794 | 1465 | 310329 |
| 614.33 | Pelvic inflammatory disease, NOS | Genitourinary | 0.98 | 0.96 | 1.01 | 324944 | 6145 | 318799 |
| 530.5 | Disorders of esophageal motility | Digestive | 1.05 | 0.97 | 1.14 | 288452 | 649 | 287803 |
| 790.6 | Other abnormal blood chemistry | Symptoms | 0.98 | 0.96 | 1.01 | 328029 | 6529 | 321500 |
| 199 | Neoplasm of uncertain behavior | Neoplasms | 0.96 | 0.91 | 1.02 | 230468 | 1156 | 229312 |
| 597 | Other disorders of urethra and urinary tract | Genitourinary | 0.96 | 0.90 | 1.02 | 314510 | 874 | 313636 |
| 450 | Noninfectious disorders of lymphatic channels | Circulatory System | 0.95 | 0.89 | 1.03 | 328240 | 717 | 327523 |
| 433.3 | Cerebral ischemia | Circulatory System | 0.96 | 0.90 | 1.02 | 319041 | 934 | 318107 |
| 290.2 | Delirium due to conditions classified elsewhere | Mental Disorders | 0.95 | 0.88 | 1.03 | 322151 | 681 | 321470 |
| 695.42 | Systemic lupus erythematosus | Dermatologic | 1.07 | 0.96 | 1.18 | 320784 | 363 | 320421 |
| 226 | Benign neoplasm of thyroid glands | Neoplasms | 1.08 | 0.96 | 1.22 | 326908 | 266 | 326642 |
| 528.11 | Stomatitis and mucositis (ulcerative) | Digestive | 1.06 | 0.96 | 1.17 | 322945 | 411 | 322534 |
| 575.8 | Other disorders of biliary tract | Digestive | 0.96 | 0.91 | 1.02 | 311372 | 1049 | 310323 |
| 938.2 | Chronic dermatitis due to solar radiation | Injuries & Poisonings | 0.93 | 0.82 | 1.05 | 322170 | 264 | 321906 |
| 276.14 | Hypopotassemia | Endocrine/Metabolic | 0.97 | 0.92 | 1.02 | 322115 | 1471 | 320644 |
| 634 | Miscarriage; stillbirth | Pregnancy Complications | 0.98 | 0.95 | 1.01 | 324534 | 4569 | 319965 |
| 195 | Cancer, suspected or other | Neoplasms | 1.04 | 0.98 | 1.11 | 230227 | 915 | 229312 |
| 348.8 | Encephalopathy, not elsewhere classified | Neurological | 1.09 | 0.95 | 1.25 | 286356 | 204 | 286152 |
| 361.1 | Retinal detachment with retinal defect | Sense Organs | 0.97 | 0.92 | 1.02 | 318238 | 1456 | 316782 |

| | | | | | | | | | |
|---|---|---|---|---|---|---|---|---|---|
| 803.1 | Fracture of humerus | Injuries & Poisonings | 0.93 | 0.83 | 1.05 | 323850 | 293 | 323557 |
| 752 | Nervous system congenital anomalies | Congenital Anomalies | 0.93 | 0.83 | 1.05 | 327963 | 288 | 327675 |
| 870.4 | Open wound of nose and sinus | Injuries & Poisonings | 1.08 | 0.95 | 1.23 | 319969 | 238 | 319731 |
| 364.5 | Corneal dystrophy | Sense Organs | 1.08 | 0.95 | 1.24 | 316998 | 216 | 316782 |
| 281.11 | Pernicious anemia | Hematopoietic | 0.96 | 0.89 | 1.03 | 309650 | 750 | 308900 |
| 530 | Diseases of esophagus | Digestive | 1.04 | 0.97 | 1.12 | 288604 | 801 | 287803 |
| 647.1 | Infections of genitourinary tract during pregnancy | Pregnancy Complications | 1.07 | 0.95 | 1.21 | 328128 | 284 | 327844 |
| 465.2 | Acute pharyngitis | Respiratory | 0.96 | 0.90 | 1.03 | 326053 | 874 | 325179 |
| 372 | Disorders of conjunctiva | Sense Organs | 0.96 | 0.91 | 1.02 | 319431 | 1043 | 318388 |
| 618.2 | Uterine/Uterovaginal prolapse | Genitourinary | 1.02 | 0.99 | 1.04 | 321713 | 5471 | 316242 |
| 345.1 | Epilepsy | Neurological | 1.07 | 0.96 | 1.19 | 286483 | 331 | 286152 |
| 965.1 | Opiates and related narcotics causing adverse effects in therapeutic use | Injuries & Poisonings | 1.04 | 0.98 | 1.10 | 301372 | 1161 | 300211 |
| 286.12 | Congenital deficiency of other clotting factors (including factor VII) | Hematopoietic | 0.93 | 0.83 | 1.05 | 325800 | 303 | 325497 |
| 743.9 | Osteopenia or other disorder of bone and cartilage | Musculoskeletal | 0.98 | 0.95 | 1.01 | 328188 | 3989 | 324199 |
| 574.1 | Cholelithiasis | Digestive | 1.01 | 0.99 | 1.03 | 319629 | 9306 | 310323 |
| 702.1 | Actinic keratosis | Dermatologic | 1.02 | 0.98 | 1.06 | 325264 | 2664 | 322600 |
| 646 | Other complications of pregnancy NEC | Pregnancy Complications | 1.03 | 0.98 | 1.07 | 328240 | 2396 | 325844 |
| 610.8 | Other specified benign mammary dysplasias | Genitourinary | 1.05 | 0.97 | 1.13 | 321576 | 703 | 320873 |
| 735.3 | Hallux valgus (Bunion) | Musculoskeletal | 1.01 | 0.99 | 1.04 | 323592 | 6905 | 316687 |
| 458.1 | Orthostatic hypotension | Circulatory System | 0.97 | 0.92 | 1.02 | 195553 | 1374 | 194179 |
| 624.9 | stress incontinence, female | Genitourinary | 1.02 | 0.99 | 1.04 | 324769 | 6100 | 318669 |
| 967 | Adverse effects of sedatives or other central nervous system depressants and anesthetics | Injuries & Poisonings | 0.95 | 0.88 | 1.03 | 300790 | 579 | 300211 |
| 426.23 | Second degree AV block | Circulatory System | 1.06 | 0.96 | 1.16 | 299982 | 461 | 299521 |
| 442.11 | Abdominal aortic aneurysm | Circulatory System | 0.96 | 0.90 | 1.03 | 320257 | 897 | 319360 |
| 316 | Substance addiction and disorders | Mental Disorders | 1.06 | 0.96 | 1.17 | 298042 | 373 | 297669 |
| 740.12 | Osteoarthrosis, localized, secondary | Musculoskeletal | 0.93 | 0.82 | 1.05 | 306589 | 264 | 306325 |
| 365.11 | Primary open angle glaucoma | Sense Organs | 1.04 | 0.98 | 1.10 | 317838 | 1056 | 316782 |
| 727.1 | Synovitis and tenosynovitis | Musculoskeletal | 1.04 | 0.97 | 1.13 | 305641 | 687 | 304954 |
| 200 | Myeloproliferative disease | Neoplasms | 1.04 | 0.97 | 1.13 | 324202 | 679 | 323523 |
| 585.1 | Acute renal failure | Genitourinary | 0.98 | 0.95 | 1.01 | 318744 | 4665 | 314079 |
| 556 | Ulceration of the lower GI tract | Digestive | 0.95 | 0.86 | 1.04 | 258836 | 440 | 258396 |
| 427.41 | Ventricular fibrillation and flutter | Circulatory System | 1.06 | 0.96 | 1.18 | 299866 | 345 | 299521 |
| 627.3 | Postmenopausal atrophic vaginitis | Genitourinary | 1.03 | 0.98 | 1.10 | 297359 | 1128 | 296231 |
| 278.1 | Obesity | Endocrine/Metabolic | 0.99 | 0.97 | 1.01 | 328110 | 11143 | 316967 |
| 246 | Other disorders of thyroid | Endocrine/Metabolic | 0.99 | 0.98 | 1.01 | 327738 | 17409 | 310329 |
| 504 | Other alveolar and parietoalveolar pneumonopathy | Respiratory | 0.94 | 0.84 | 1.05 | 316793 | 309 | 316484 |
| 337 | Disorders of the autonomic nervous system | Neurological | 1.08 | 0.94 | 1.24 | 286359 | 207 | 286152 |
| 218.1 | Uterine leiomyoma | Neoplasms | 0.99 | 0.97 | 1.01 | 318346 | 10566 | 307780 |

| | | | | | | | | | |
|---|---|---|---|---|---|---|---|---|---|
| 180.3 | Cervical intraepithelial neoplasia [CIN] [Cervical dysplasia] | Neoplasms | 1.02 | 0.98 | 1.07 | 299736 | 2153 | 297583 |
| 526.1 | Cysts of the jaws | Digestive | 1.06 | 0.96 | 1.18 | 311993 | 335 | 311658 |
| 512.7 | Shortness of breath | Respiratory | 1.01 | 0.99 | 1.04 | 320384 | 6116 | 314268 |
| 704 | Diseases of hair and hair follicles | Dermatologic | 1.02 | 0.99 | 1.04 | 326692 | 5263 | 321429 |
| 184.1 | Malignant neoplasm of ovary and other uterine adnexa | Neoplasms | 0.98 | 0.94 | 1.02 | 308106 | 2553 | 305553 |
| 560.1 | Paralytic ileus | Digestive | 0.95 | 0.87 | 1.04 | 258916 | 520 | 258396 |
| 297.2 | Suicide or self-inflicted injury | Mental Disorders | 1.02 | 0.98 | 1.06 | 285655 | 2757 | 282898 |
| 213 | Benign neoplasm of bone and articular cartilage | Neoplasms | 1.06 | 0.95 | 1.19 | 277528 | 322 | 277206 |
| 526.41 | Temporomandibular joint disorder, unspecified | Digestive | 1.08 | 0.95 | 1.22 | 311886 | 228 | 311658 |
| 292 | Neurological disorders | Mental Disorders | 1.05 | 0.96 | 1.16 | 321919 | 449 | 321470 |
| 724.1 | Disorders of sacrum | Musculoskeletal | 0.94 | 0.83 | 1.05 | 311225 | 281 | 310944 |
| 202 | Cancer of other lymphoid, histiocytic tissue | Neoplasms | 0.97 | 0.91 | 1.03 | 324661 | 1138 | 323523 |
| 272.9 | Unspecified disorder of lipoid metabolism | Endocrine/Metabolic | 0.93 | 0.81 | 1.06 | 291721 | 224 | 291497 |
| 559 | Ileostomy status | Digestive | 1.03 | 0.98 | 1.08 | 260069 | 1673 | 258396 |
| 686.1 | Carbuncle and furuncle | Dermatologic | 1.02 | 0.98 | 1.06 | 318829 | 2422 | 316407 |
| 70.4 | Chronic hepatitis | Infectious Diseases | 1.07 | 0.95 | 1.20 | 322496 | 290 | 322206 |
| 724.2 | Disorders of coccyx | Musculoskeletal | 0.94 | 0.83 | 1.05 | 311226 | 282 | 310944 |
| 569 | Other disorders of intestine | Digestive | 1.00 | 1.00 | 1.01 | 326148 | 79488 | 246660 |
| 145.2 | Cancer of tongue | Neoplasms | 0.94 | 0.84 | 1.05 | 326366 | 325 | 326041 |
| 590 | Pyelonephritis | Genitourinary | 1.02 | 0.99 | 1.05 | 300960 | 3925 | 297035 |
| 272.11 | Hypercholesterolemia | Endocrine/Metabolic | 0.99 | 0.98 | 1.01 | 325499 | 34002 | 291497 |
| 598 | Abnormal findings on examination of urine | Genitourinary | 1.02 | 0.98 | 1.05 | 328240 | 3386 | 324854 |
| 571.6 | Primary biliary cirrhosis | Digestive | 0.93 | 0.83 | 1.06 | 318589 | 258 | 318331 |
| 172.2 | Other non-epithelial cancer of skin | Neoplasms | 1.01 | 0.99 | 1.03 | 325462 | 11242 | 314220 |
| 636.3 | Hemorrhage in early pregnancy | Pregnancy Complications | 1.03 | 0.97 | 1.10 | 321078 | 1113 | 319965 |
| 579 | Other symptoms involving abdomen and pelvis | Digestive | 0.97 | 0.92 | 1.03 | 304580 | 1186 | 303394 |
| 274.1 | Gout | Endocrine/Metabolic | 0.97 | 0.93 | 1.02 | 327930 | 1668 | 326262 |
| 592.13 | Chronic interstitial cystitis | Genitourinary | 0.93 | 0.83 | 1.06 | 297289 | 254 | 297035 |
| 530.1 | Esophagitis, GERD and related diseases | Digestive | 0.99 | 0.97 | 1.01 | 297342 | 9539 | 287803 |
| 276.6 | Fluid overload | Endocrine/Metabolic | 0.95 | 0.87 | 1.04 | 321117 | 473 | 320644 |
| 858 | Complication of internal orthopedic device | Injuries & Poisonings | 0.98 | 0.95 | 1.02 | 317147 | 3183 | 313964 |
| 771 | Musculoskeletal symptoms referable to limbs | Symptoms | 1.04 | 0.97 | 1.11 | 327405 | 891 | 326514 |
| 371.3 | Inflammation of eyelids | Sense Organs | 0.98 | 0.94 | 1.02 | 320786 | 2398 | 318388 |
| 78 | Viral warts & HPV | Infectious Diseases | 1.03 | 0.97 | 1.10 | 323218 | 1012 | 322206 |
| 540.1 | Appendicitis | Digestive | 1.04 | 0.97 | 1.11 | 325174 | 795 | 324379 |
| 367.1 | Myopia | Sense Organs | 1.03 | 0.97 | 1.09 | 327025 | 1271 | 325754 |
| 618.1 | Prolapse of vaginal walls | Genitourinary | 1.01 | 0.99 | 1.04 | 323726 | 7484 | 316242 |
| 854 | Complications of cardiac/vascular device, implant, and graft | Injuries & Poisonings | 0.98 | 0.93 | 1.02 | 315807 | 1843 | 313964 |

| | | | | | | | | |
|---|---|---|---|---|---|---|---|---|
| 250.7 | Diabetic retinopathy | Endocrine/Metabolic | 0.97 | 0.92 | 1.03 | 317232 | 1380 | 315852 |
| 519.8 | Other diseases of respiratory system, NEC | Respiratory | 1.01 | 0.99 | 1.03 | 274529 | 9082 | 265447 |
| 614.51 | Cervicitis and endocervicitis | Genitourinary | 0.97 | 0.92 | 1.03 | 320048 | 1249 | 318799 |
| 535.2 | Atrophic gastritis | Digestive | 1.06 | 0.95 | 1.17 | 296869 | 358 | 296511 |
| 625 | Pain and other symptoms associated with female genital organs | Genitourinary | 0.98 | 0.93 | 1.02 | 320718 | 2049 | 318669 |
| 597.1 | Urethral stricture (not specified as infectious) | Genitourinary | 1.02 | 0.98 | 1.05 | 317079 | 3443 | 313636 |
| 317.1 | Alcoholism | Mental Disorders | 0.99 | 0.97 | 1.01 | 306366 | 8697 | 297669 |
| 994.2 | Sepsis | Injuries & Poisonings | 0.98 | 0.95 | 1.02 | 328240 | 2885 | 325355 |
| 557 | Intestinal malabsorption (non-celiac) | Digestive | 1.06 | 0.95 | 1.19 | 258690 | 294 | 258396 |
| 655 | Known or suspected fetal abnormality affecting management of mother | Pregnancy Complications | 1.02 | 0.99 | 1.05 | 328240 | 4575 | 323665 |
| 427.11 | Paroxysmal supraventricular tachycardia | Circulatory System | 0.98 | 0.94 | 1.02 | 301952 | 2431 | 299521 |
| 296.1 | Bipolar | Mental Disorders | 1.03 | 0.97 | 1.09 | 284019 | 1121 | 282898 |
| 728.7 | Fasciitis | Musculoskeletal | 0.93 | 0.81 | 1.07 | 305167 | 213 | 304954 |
| 870.1 | Open wound or laceration of eye or eyelid | Injuries & Poisonings | 0.94 | 0.84 | 1.06 | 320012 | 281 | 319731 |
| 727 | Other disorders of synovium, tendon, and bursa | Musculoskeletal | 1.04 | 0.97 | 1.12 | 305708 | 754 | 304954 |
| 289.3 | Personal history of diseases of blood and blood-forming organs | Hematopoietic | 0.94 | 0.84 | 1.06 | 320615 | 297 | 320318 |
| 514 | Abnormal findings examination of lungs | Respiratory | 1.02 | 0.98 | 1.06 | 328212 | 2577 | 325635 |
| 455 | Hemorrhoids | Circulatory System | 0.99 | 0.98 | 1.01 | 312410 | 24697 | 287713 |
| 153.2 | Colon cancer | Neoplasms | 0.98 | 0.95 | 1.02 | 304402 | 3122 | 301280 |
| 172.3 | Carcinoma in situ of skin | Neoplasms | 1.04 | 0.96 | 1.12 | 314884 | 664 | 314220 |
| 751.22 | Other specified congenital anomalies of kidney | Congenital Anomalies | 0.95 | 0.85 | 1.05 | 326353 | 350 | 326003 |
| 516.1 | Hemoptysis | Respiratory | 1.02 | 0.98 | 1.07 | 328154 | 2103 | 326051 |
| 458.2 | Iatrogenic hypotension | Circulatory System | 0.94 | 0.82 | 1.06 | 194417 | 238 | 194179 |
| 225.1 | Benign neoplasm of brain, cranial nerves, meninges | Neoplasms | 1.04 | 0.97 | 1.11 | 327267 | 820 | 326447 |
| 427.4 | Cardiac arrest and ventricular fibrillation | Circulatory System | 1.06 | 0.95 | 1.17 | 299869 | 348 | 299521 |
| 613.1 | Inflammatory disease of breast | Genitourinary | 0.96 | 0.90 | 1.04 | 323156 | 738 | 322418 |
| 208 | Benign neoplasm of colon | Neoplasms | 0.99 | 0.98 | 1.01 | 325437 | 20827 | 304610 |
| 506 | Empyema and pneumothorax | Respiratory | 0.97 | 0.92 | 1.03 | 317630 | 1146 | 316484 |
| 496.21 | Obstructive chronic bronchitis | Respiratory | 0.98 | 0.94 | 1.02 | 296921 | 2729 | 294192 |
| 276.41 | Acidosis | Endocrine/Metabolic | 0.97 | 0.91 | 1.03 | 321700 | 1056 | 320644 |
| 741 | Symptoms and disorders of the joints | Musculoskeletal | 1.01 | 0.99 | 1.02 | 327531 | 16889 | 310642 |
| 696.4 | Psoriasis | Dermatologic | 1.02 | 0.98 | 1.08 | 315554 | 1622 | 313932 |
| 70 | Viral hepatitis | Infectious Diseases | 1.04 | 0.96 | 1.12 | 322928 | 722 | 322206 |
| 737.3 | Kyphoscoliosis and scoliosis | Musculoskeletal | 0.95 | 0.86 | 1.05 | 317058 | 371 | 316687 |
| 187.1 | Malignant neoplasm of unspecified male genital organ | Neoplasms | 0.99 | 0.97 | 1.01 | 327699 | 8481 | 319218 |
| 153.3 | Malignant neoplasm of rectum, rectosigmoid junction, and anus | Neoplasms | 0.98 | 0.94 | 1.02 | 303397 | 2117 | 301280 |
| 443.1 | Raynaud's syndrome | Circulatory System | 1.03 | 0.97 | 1.09 | 320525 | 1165 | 319360 |
| 740.11 | Osteoarthrosis, localized, primary | Musculoskeletal | 1.01 | 0.99 | 1.03 | 315552 | 9227 | 306325 |

| | | | | | | | | |
|---|---|---|---|---|---|---|---|---|
| 359.2 | Myopathy | Neurological | 0.96 | 0.88 | 1.05 | 326450 | 498 | 325952 |
| 751.2 | Congenital anomalies of urinary system | Congenital Anomalies | 0.97 | 0.91 | 1.03 | 326908 | 905 | 326003 |
| 724.9 | Other unspecified back disorders | Musculoskeletal | 1.01 | 0.99 | 1.02 | 328029 | 17085 | 310944 |
| 189 | Cancer of urinary organs (incl. kidney and bladder) | Neoplasms | 1.02 | 0.98 | 1.06 | 326965 | 3003 | 323962 |
| 350.3 | Lack of coordination | Neurological | 1.05 | 0.95 | 1.17 | 325804 | 360 | 325444 |
| 604 | Disorders of penis | Genitourinary | 1.04 | 0.96 | 1.11 | 308641 | 735 | 307906 |
| 512.1 | Wheezing | Respiratory | 1.07 | 0.94 | 1.21 | 314499 | 231 | 314268 |
| 496 | Chronic airway obstruction | Respiratory | 0.99 | 0.97 | 1.01 | 301827 | 7635 | 294192 |
| 594.2 | Calculus of lower urinary tract | Genitourinary | 0.97 | 0.90 | 1.04 | 320761 | 830 | 319931 |
| 714.1 | Rheumatoid arthritis | Musculoskeletal | 1.02 | 0.98 | 1.06 | 319560 | 2307 | 317253 |
| 573.5 | Jaundice (not of newborn) | Digestive | 1.03 | 0.97 | 1.10 | 319269 | 938 | 318331 |
| 352.1 | Trigeminal nerve disorders [CN5] | Neurological | 0.96 | 0.87 | 1.05 | 313473 | 462 | 313011 |
| 789 | Nausea and vomiting | Symptoms | 0.99 | 0.97 | 1.01 | 328240 | 11983 | 316257 |
| 608 | Other disorders of male genital organs | Genitourinary | 1.01 | 0.99 | 1.02 | 327998 | 20092 | 307906 |
| 136 | Other infectious and parasitic diseases | Infectious Diseases | 1.05 | 0.95 | 1.15 | 327982 | 428 | 327554 |
| 275.1 | Disorders of iron metabolism | Hematopoietic | 1.04 | 0.96 | 1.12 | 326735 | 700 | 326035 |
| 564.1 | Irritable Bowel Syndrome | Digestive | 1.01 | 0.99 | 1.04 | 264057 | 5661 | 258396 |
| 836 | Traumatic arthropathy | Injuries & Poisonings | 1.06 | 0.94 | 1.20 | 322016 | 243 | 321773 |
| 738 | Other acquired musculoskeletal deformity | Musculoskeletal | 0.95 | 0.87 | 1.05 | 317104 | 417 | 316687 |
| 362.2 | Degeneration of macula and posterior pole of retina | Sense Organs | 0.98 | 0.94 | 1.02 | 318055 | 2203 | 315852 |
| 362.29 | Macular degeneration (senile) of retina NOS | Sense Organs | 0.98 | 0.94 | 1.02 | 318052 | 2200 | 315852 |
| 353 | Nerve root and plexus disorders | Neurological | 1.03 | 0.97 | 1.10 | 314005 | 994 | 313011 |
| 480 | Pneumonia | Respiratory | 0.99 | 0.95 | 1.02 | 321603 | 3914 | 317689 |
| 618 | Genital prolapse | Genitourinary | 0.96 | 0.89 | 1.04 | 316862 | 620 | 316242 |
| 384.4 | Perforation of tympanic membrane | Sense Organs | 0.97 | 0.91 | 1.03 | 325167 | 1024 | 324143 |
| 613.8 | Other specified disorders of breast | Genitourinary | 0.95 | 0.86 | 1.06 | 322773 | 355 | 322418 |
| 327 | Sleep disorders | Neurological | 1.03 | 0.97 | 1.09 | 323754 | 1034 | 322720 |
| 716.1 | Unspecified polyarthropathy or polyarthritis | Musculoskeletal | 1.02 | 0.98 | 1.05 | 277426 | 3530 | 273896 |
| 364 | Corneal opacity and other disorders of cornea | Sense Organs | 1.06 | 0.93 | 1.21 | 317009 | 227 | 316782 |
| 427.7 | Tachycardia NOS | Circulatory System | 1.02 | 0.98 | 1.06 | 301787 | 2266 | 299521 |
| 427.5 | Arrhythmia (cardiac) NOS | Circulatory System | 0.97 | 0.91 | 1.03 | 300469 | 948 | 299521 |
| 578.1 | Hematemesis | Digestive | 0.98 | 0.94 | 1.02 | 305428 | 2034 | 303394 |
| 599.9 | Other abnormality of urination | Genitourinary | 1.02 | 0.98 | 1.07 | 222371 | 1944 | 220427 |
| 292.3 | Memory loss | Mental Disorders | 0.97 | 0.90 | 1.04 | 322208 | 738 | 321470 |
| 274.21 | Chondrocalcinosis | Endocrine/Metabolic | 1.05 | 0.94 | 1.18 | 326552 | 290 | 326262 |
| 250.23 | Type 2 diabetes with ophthalmic manifestations | Endocrine/Metabolic | 0.98 | 0.92 | 1.03 | 308834 | 1344 | 307490 |
| 619.4 | Noninflammatory disorders of vagina | Genitourinary | 1.02 | 0.97 | 1.07 | 280328 | 1780 | 278548 |
| 715 | Other inflammatory spondylopathies | Musculoskeletal | 1.06 | 0.94 | 1.19 | 317524 | 271 | 317253 |

| | | | | | | | | | |
|---|---|---|---|---|---|---|---|---|---|
| 602 | Other disorders of prostate | Genitourinary | 0.98 | 0.92 | 1.03 | 308765 | 1372 | 307393 |
| 736 | Other acquired deformities of limbs | Musculoskeletal | 1.05 | 0.94 | 1.18 | 316998 | 311 | 316687 |
| 430.2 | Intracerebral hemorrhage | Circulatory System | 0.97 | 0.90 | 1.04 | 318830 | 723 | 318107 |
| 681.3 | Cellulitis and abscess of arm/hand | Dermatologic | 0.99 | 0.96 | 1.01 | 322177 | 5770 | 316407 |
| 276.11 | Hyperosmolality and/or hypernatremia | Endocrine/Metabolic | 1.06 | 0.93 | 1.20 | 320891 | 247 | 320644 |
| 269 | Proteinuria | Endocrine/Metabolic | 1.05 | 0.94 | 1.16 | 327850 | 355 | 327495 |
| 290.1 | Dementias | Mental Disorders | 1.03 | 0.96 | 1.12 | 322121 | 651 | 321470 |
| 550 | Abdominal hernia | Digestive | 1.00 | 0.99 | 1.01 | 328240 | 48451 | 279789 |
| 433.5 | Cerebral aneurysm | Circulatory System | 0.96 | 0.87 | 1.06 | 318505 | 398 | 318107 |
| 185 | Cancer of prostate | Neoplasms | 0.99 | 0.97 | 1.01 | 315856 | 8463 | 307393 |
| 512 | Other symptoms of respiratory system | Respiratory | 0.99 | 0.96 | 1.02 | 319772 | 5504 | 314268 |
| 801 | Fracture of ankle and foot | Injuries & Poisonings | 0.95 | 0.84 | 1.07 | 323808 | 251 | 323557 |
| 614.32 | Chronic inflammatory pelvic disease | Genitourinary | 0.96 | 0.87 | 1.06 | 319224 | 425 | 318799 |
| 512.8 | Cough | Respiratory | 1.02 | 0.98 | 1.05 | 317232 | 2964 | 314268 |
| 596.5 | Functional disorders of bladder | Genitourinary | 1.02 | 0.97 | 1.07 | 315318 | 1682 | 313636 |
| 81 | Infection/inflammation of internal prosthetic device; implant; and graft | Infectious Diseases | 0.98 | 0.95 | 1.02 | 323993 | 2514 | 321479 |
| 218.2 | Other benign neoplasm of uterus | Neoplasms | 1.05 | 0.94 | 1.16 | 308143 | 363 | 307780 |
| 681.6 | Cellulitis and abscess of foot, toe | Dermatologic | 0.99 | 0.96 | 1.02 | 322141 | 5734 | 316407 |
| 599.4 | Urinary incontinence | Genitourinary | 1.01 | 0.99 | 1.03 | 229423 | 8996 | 220427 |
| 433.21 | Cerebral artery occlusion, with cerebral infarction | Circulatory System | 0.98 | 0.93 | 1.03 | 319641 | 1534 | 318107 |
| 627.2 | Symptomatic menopause | Genitourinary | 1.06 | 0.92 | 1.22 | 296434 | 203 | 296231 |
| 38 | Septicemia | Infectious Diseases | 0.98 | 0.95 | 1.02 | 315677 | 3045 | 312632 |
| 344 | Other paralytic syndromes | Neurological | 0.97 | 0.90 | 1.04 | 286841 | 689 | 286152 |
| 433.31 | Transient cerebral ischemia | Circulatory System | 0.98 | 0.94 | 1.02 | 320281 | 2174 | 318107 |
| 429.2 | Abnormal function study of cardiovascular system | Circulatory System | 0.97 | 0.89 | 1.05 | 322577 | 544 | 322033 |
| 369 | Infection of the eye | Sense Organs | 0.95 | 0.84 | 1.08 | 318642 | 254 | 318388 |
| 289.5 | Diseases of spleen | Hematopoietic | 0.97 | 0.89 | 1.05 | 320846 | 528 | 320318 |
| 381.9 | Otorrhea | Sense Organs | 0.95 | 0.85 | 1.07 | 324420 | 277 | 324143 |
| 415 | Pulmonary heart disease | Circulatory System | 1.01 | 0.98 | 1.05 | 325300 | 3753 | 321547 |
| 635.3 | Placenta previa and abruptio placenta | Pregnancy Complications | 1.02 | 0.97 | 1.08 | 321301 | 1336 | 319965 |
| 681.5 | Cellulitis and abscess of leg, except foot | Dermatologic | 0.99 | 0.96 | 1.02 | 322184 | 5777 | 316407 |
| 202.2 | Non-Hodgkins lymphoma | Neoplasms | 1.01 | 0.98 | 1.05 | 327055 | 3532 | 323523 |
| 697 | Sarcoidosis | Dermatologic | 1.03 | 0.95 | 1.12 | 322309 | 560 | 321749 |
| 327.3 | Sleep apnea | Neurological | 1.01 | 0.98 | 1.04 | 327419 | 4699 | 322720 |
| 214.1 | Lipoma of skin and subcutaneous tissue | Neoplasms | 0.99 | 0.96 | 1.02 | 325432 | 4756 | 320676 |
| 601.8 | Other inflammatory disorders of male genital organs | Genitourinary | 0.96 | 0.85 | 1.07 | 307698 | 305 | 307393 |
| 90 | Sexually transmitted infections (not HIV or hepatitis) | Infectious Diseases | 1.05 | 0.93 | 1.17 | 328239 | 298 | 327941 |
| 871 | Open wounds of extremities | Injuries & Poisonings | 0.99 | 0.96 | 1.02 | 323873 | 4142 | 319731 |

| | | | | | | | | |
|---|---|---|---|---|---|---|---|---|
| 426.4 | Anomalous atrioventricular excitation | Circulatory System | 1.05 | 0.93 | 1.19 | 299761 | 240 | 299521 |
| 149.1 | Cancer of oropharynx | Neoplasms | 1.05 | 0.93 | 1.19 | 326297 | 256 | 326041 |
| 722 | Intervertebral disc disorders | Musculoskeletal | 1.01 | 0.98 | 1.04 | 316907 | 5963 | 310944 |
| 574.2 | Calculus of bile duct | Digestive | 1.02 | 0.98 | 1.05 | 313011 | 2688 | 310323 |
| 275.3 | Disorders of magnesium metabolism | Endocrine/Metabolic | 0.96 | 0.87 | 1.06 | 326410 | 375 | 326035 |
| 446.5 | Giant cell arteritis | Circulatory System | 0.96 | 0.87 | 1.06 | 319750 | 390 | 319360 |
| 389.4 | Tinnitus | Sense Organs | 1.03 | 0.95 | 1.13 | 318735 | 541 | 318194 |
| 292.1 | Aphasia/speech disturbance | Mental Disorders | 0.98 | 0.93 | 1.03 | 323019 | 1549 | 321470 |
| 801.1 | Fracture of foot | Injuries & Poisonings | 0.96 | 0.87 | 1.06 | 323975 | 418 | 323557 |
| 394.3 | Aortic valve disease | Circulatory System | 0.98 | 0.93 | 1.03 | 322836 | 1308 | 321528 |
| 159 | Malignant neoplasm of other and ill-defined sites within the digestive organs and peritoneum | Neoplasms | 0.99 | 0.96 | 1.02 | 317620 | 5289 | 312331 |
| 362 | Other retinal disorders | Sense Organs | 0.98 | 0.92 | 1.04 | 316825 | 973 | 315852 |
| 348.7 | Coma | Neurological | 1.04 | 0.94 | 1.15 | 286531 | 379 | 286152 |
| 586.4 | Stricture/obstruction of ureter | Genitourinary | 0.98 | 0.91 | 1.04 | 315018 | 939 | 314079 |
| 368.4 | Visual field defects | Sense Organs | 0.96 | 0.86 | 1.07 | 325215 | 333 | 324882 |
| 805 | Fracture of vertebral column without mention of spinal cord injury | Injuries & Poisonings | 0.96 | 0.86 | 1.07 | 323890 | 333 | 323557 |
| 300.12 | Agorophobia, social phobia, and panic disorder | Mental Disorders | 0.97 | 0.90 | 1.05 | 283642 | 744 | 282898 |
| 687 | Symptoms affecting skin | Dermatologic | 0.95 | 0.83 | 1.09 | 323032 | 218 | 322814 |
| 585.2 | Renal failure NOS | Genitourinary | 0.99 | 0.97 | 1.01 | 322824 | 8745 | 314079 |
| 454.1 | Varicose veins of lower extremity | Circulatory System | 1.01 | 0.99 | 1.03 | 299275 | 11562 | 287713 |
| 599.3 | Dysuria | Genitourinary | 1.02 | 0.97 | 1.08 | 221636 | 1209 | 220427 |
| 386.3 | Labyrinthitis | Sense Organs | 0.97 | 0.91 | 1.04 | 322440 | 791 | 321649 |
| 262 | Mineral deficiency NEC | Endocrine/Metabolic | 1.05 | 0.92 | 1.19 | 326027 | 241 | 325786 |
| 150 | Cancer of esophagus | Neoplasms | 1.03 | 0.96 | 1.11 | 313058 | 727 | 312331 |
| 444 | Arterial embolism and thrombosis | Circulatory System | 0.97 | 0.88 | 1.06 | 319794 | 434 | 319360 |
| 348.2 | Cerebral edema and compression of brain | Neurological | 1.05 | 0.93 | 1.18 | 286412 | 260 | 286152 |
| 622.2 | Mucous polyp of cervix | Genitourinary | 0.99 | 0.95 | 1.02 | 318829 | 3522 | 315307 |
| 279.7 | Other immunological findings | Endocrine/Metabolic | 0.96 | 0.85 | 1.08 | 327978 | 264 | 327714 |
| 195.1 | Malignant neoplasm, other | Neoplasms | 1.00 | 0.99 | 1.00 | 320138 | 90826 | 229312 |
| 555.1 | Regional enteritis | Digestive | 1.02 | 0.97 | 1.07 | 260201 | 1805 | 258396 |
| 613 | Other nonmalignant breast conditions | Genitourinary | 0.96 | 0.87 | 1.07 | 322786 | 368 | 322418 |
| 331.1 | Hydrocephalus | Neurological | 0.97 | 0.89 | 1.06 | 286652 | 500 | 286152 |
| 394.7 | Disease of tricuspid valve | Circulatory System | 0.98 | 0.92 | 1.04 | 322614 | 1086 | 321528 |
| 189.21 | Malignant neoplasm of bladder | Neoplasms | 1.02 | 0.97 | 1.06 | 326150 | 2188 | 323962 |
| 38.1 | Gram negative septicemia | Infectious Diseases | 1.02 | 0.96 | 1.10 | 313487 | 855 | 312632 |
| 172.11 | Melanomas of skin | Neoplasms | 1.01 | 0.98 | 1.05 | 316943 | 2723 | 314220 |
| 579.8 | Nonspecific abnormal findings in stool contents | Digestive | 1.02 | 0.97 | 1.07 | 305134 | 1740 | 303394 |
| 292.4 | Altered mental status | Mental Disorders | 1.02 | 0.97 | 1.06 | 323742 | 2272 | 321470 |

| | | | | | | | | | |
|---|---|---|---|---|---|---|---|---|---|
| 276.13 | Hyperpotassemia | Endocrine/Metabolic | 0.98 | 0.92 | 1.04 | 321623 | 979 | 320644 |
| 427.9 | Palpitations | Circulatory System | 0.99 | 0.96 | 1.02 | 303480 | 3959 | 299521 |
| 430.1 | Subarachnoid hemorrhage | Circulatory System | 1.02 | 0.96 | 1.10 | 318931 | 824 | 318107 |
| 550.4 | Umbilical hernia | Digestive | 1.01 | 0.98 | 1.04 | 283693 | 3904 | 279789 |
| 586 | Other disorders of the kidney and ureters | Genitourinary | 0.99 | 0.95 | 1.02 | 317490 | 3411 | 314079 |
| 626.8 | Infertility, female | Genitourinary | 0.98 | 0.93 | 1.03 | 297638 | 1407 | 296231 |
| 79 | Viral infection | Infectious Diseases | 1.01 | 0.98 | 1.04 | 326439 | 4233 | 322206 |
| 441.1 | Acute vascular insufficiency of intestine | Circulatory System | 1.04 | 0.93 | 1.18 | 319631 | 271 | 319360 |
| 614.53 | Cyst or abscess of Bartholin's gland | Genitourinary | 0.98 | 0.91 | 1.05 | 319602 | 803 | 318799 |
| 622.1 | Polyp of corpus uteri | Genitourinary | 1.01 | 0.99 | 1.03 | 323490 | 8183 | 315307 |
| 698 | Pruritus and related conditions | Dermatologic | 1.02 | 0.96 | 1.10 | 328240 | 799 | 327441 |
| 853 | Complication of colostomy or enterostomy | Injuries & Poisonings | 1.03 | 0.95 | 1.12 | 314478 | 514 | 313964 |
| 293 | Symptoms involving head and neck | Mental Disorders | 1.01 | 0.97 | 1.06 | 327323 | 2204 | 325119 |
| 747.13 | Congenital anomalies of great vessels | Congenital Anomalies | 0.98 | 0.94 | 1.03 | 327177 | 1823 | 325354 |
| 550.5 | Ventral hernia | Digestive | 1.01 | 0.98 | 1.05 | 283371 | 3582 | 279789 |
| 509.1 | Respiratory failure | Respiratory | 0.99 | 0.94 | 1.03 | 318564 | 2080 | 316484 |
| 557.1 | Celiac disease | Digestive | 0.98 | 0.94 | 1.03 | 260290 | 1894 | 258396 |
| 531.3 | Duodenal ulcer | Digestive | 0.99 | 0.95 | 1.02 | 323644 | 3095 | 320549 |
| 715.2 | Ankylosing spondylitis | Musculoskeletal | 0.97 | 0.87 | 1.07 | 317637 | 384 | 317253 |
| 338.1 | Acute pain | Neurological | 0.97 | 0.88 | 1.07 | 327846 | 420 | 327426 |
| 536.8 | Dyspepsia and other specified disorders of function of stomach | Digestive | 1.04 | 0.92 | 1.18 | 296758 | 247 | 296511 |
| 495.2 | Asthma with exacerbation | Respiratory | 1.04 | 0.92 | 1.18 | 294453 | 261 | 294192 |
| 420.2 | Pericarditis | Circulatory System | 0.98 | 0.92 | 1.04 | 326070 | 1087 | 324983 |
| 540 | Appendiceal conditions | Digestive | 1.01 | 0.98 | 1.04 | 328240 | 3861 | 324379 |
| 743.11 | Osteoporosis NOS | Musculoskeletal | 0.99 | 0.95 | 1.02 | 327235 | 3036 | 324199 |
| 242.1 | Graves' disease | Endocrine/Metabolic | 0.97 | 0.88 | 1.06 | 310789 | 460 | 310329 |
| 382 | Otalgia | Sense Organs | 1.04 | 0.93 | 1.16 | 324458 | 315 | 324143 |
| 210 | Benign neoplasm of lip, oral cavity, and pharynx | Neoplasms | 1.02 | 0.96 | 1.09 | 327034 | 993 | 326041 |
| 800.3 | Fracture of tibia and fibula | Injuries & Poisonings | 0.97 | 0.89 | 1.06 | 324092 | 535 | 323557 |
| 612.2 | Hypertrophy of breast (Gynecomastia) | Genitourinary | 1.02 | 0.96 | 1.09 | 321732 | 859 | 320873 |
| 289.4 | Lymphadenitis | Hematopoietic | 0.99 | 0.95 | 1.03 | 323033 | 2715 | 320318 |
| 740.9 | Osteoarthrosis NOS | Musculoskeletal | 1.01 | 0.98 | 1.04 | 310757 | 4432 | 306325 |
| 300.13 | Phobia | Mental Disorders | 1.03 | 0.94 | 1.12 | 283412 | 514 | 282898 |
| 749 | Congenital anomalies of face and neck | Congenital Anomalies | 0.96 | 0.85 | 1.08 | 328137 | 271 | 327866 |
| 747.11 | Cardiac shunt/ heart septal defect | Congenital Anomalies | 0.97 | 0.90 | 1.06 | 325935 | 581 | 325354 |
| 384 | Other disorders of tympanic membrane | Sense Organs | 0.97 | 0.88 | 1.07 | 324573 | 430 | 324143 |
| 627 | Menopausal and postmenopausal disorders | Genitourinary | 1.02 | 0.96 | 1.09 | 297078 | 847 | 296231 |
| 577.3 | Cyst and pseudocyst of pancreas | Digestive | 0.97 | 0.87 | 1.07 | 326295 | 375 | 325920 |

| | | | | | | | | |
|---|---|---|---|---|---|---|---|---|
| 523.32 | Chronic periodontitis | Digestive | 1.03 | 0.95 | 1.11 | 312248 | 590 | 311658 |
| 614.5 | Inflammatory disease of cervix, vagina, and vulva | Genitourinary | 1.04 | 0.93 | 1.15 | 319133 | 334 | 318799 |
| 701.5 | Abnormal granulation tissue | Dermatologic | 1.03 | 0.94 | 1.13 | 323543 | 479 | 323064 |
| 591 | Urinary tract infection | Genitourinary | 1.01 | 0.99 | 1.02 | 309711 | 12676 | 297035 |
| 323 | Encephalitis | Neurological | 1.02 | 0.96 | 1.09 | 327999 | 861 | 327138 |
| 614.1 | Pelvic peritoneal adhesions, female (postoperative) (postinfection) | Genitourinary | 0.99 | 0.95 | 1.03 | 321370 | 2571 | 318799 |
| 634.3 | Ectopic pregnancy | Pregnancy Complications | 1.04 | 0.93 | 1.15 | 320307 | 342 | 319965 |
| 426.32 | Left bundle branch block | Circulatory System | 0.99 | 0.94 | 1.03 | 301302 | 1781 | 299521 |
| 260.6 | Anorexia | Endocrine/Metabolic | 1.02 | 0.96 | 1.09 | 326636 | 850 | 325786 |
| 573.7 | Abnormal results of function study of liver | Digestive | 1.01 | 0.98 | 1.04 | 321896 | 3565 | 318331 |
| 285.22 | Anemia in neoplastic disease | Hematopoietic | 0.97 | 0.88 | 1.07 | 309337 | 437 | 308900 |
| 819 | Skull and face fracture and other intercranial injury | Injuries & Poisonings | 1.02 | 0.97 | 1.07 | 328056 | 1517 | 326539 |
| 703.1 | Ingrowing nail | Dermatologic | 0.98 | 0.92 | 1.04 | 322439 | 1010 | 321429 |
| 750.21 | Congenital anomalies of intestine | Congenital Anomalies | 0.96 | 0.84 | 1.09 | 326230 | 227 | 326003 |
| 964.1 | Anticoagulants causing adverse effects | Injuries & Poisonings | 1.04 | 0.92 | 1.17 | 300470 | 259 | 300211 |
| 301 | Personality disorders | Mental Disorders | 1.03 | 0.94 | 1.13 | 283330 | 432 | 282898 |
| 579.2 | Splenomegaly | Digestive | 1.03 | 0.94 | 1.13 | 303837 | 443 | 303394 |
| 687.1 | Rash and other nonspecific skin eruption | Dermatologic | 1.01 | 0.97 | 1.06 | 325032 | 2218 | 322814 |
| 619.2 | Disorders of uterus, NEC | Genitourinary | 1.01 | 0.98 | 1.05 | 281939 | 3391 | 278548 |
| 965.3 | Salicylates causing adverse effects in therapeutic use | Injuries & Poisonings | 0.97 | 0.88 | 1.07 | 300612 | 401 | 300211 |
| 531.4 | Peptic ulcer, site unspecified | Digestive | 0.97 | 0.88 | 1.07 | 320998 | 449 | 320549 |
| 201 | Hodgkin's disease | Neoplasms | 0.96 | 0.85 | 1.09 | 323794 | 271 | 323523 |
| 443.7 | Peripheral angiopathy in diseases classified elsewhere | Circulatory System | 1.03 | 0.94 | 1.13 | 319775 | 415 | 319360 |
| 528.5 | Diseases of lips | Digestive | 1.02 | 0.95 | 1.10 | 323215 | 681 | 322534 |
| 155 | Cancer of liver and intrahepatic bile duct | Neoplasms | 1.04 | 0.92 | 1.18 | 312576 | 245 | 312331 |
| 451 | Phlebitis and thrombophlebitis | Circulatory System | 0.97 | 0.88 | 1.07 | 288110 | 397 | 287713 |
| 338.2 | Chronic pain | Neurological | 1.03 | 0.93 | 1.14 | 327830 | 404 | 327426 |
| 496.3 | Bronchiectasis | Respiratory | 0.99 | 0.94 | 1.03 | 296077 | 1885 | 294192 |
| 614.52 | Vaginitis and vulvovaginitis | Genitourinary | 0.97 | 0.88 | 1.07 | 319219 | 420 | 318799 |
| 284 | Aplastic anemia | Hematopoietic | 0.99 | 0.98 | 1.01 | 321659 | 12759 | 308900 |
| 189.11 | Malignant neoplasm of kidney, except pelvis | Neoplasms | 0.98 | 0.92 | 1.04 | 324997 | 1035 | 323962 |
| 333.4 | Torsion dystonia | Neurological | 0.96 | 0.85 | 1.09 | 286417 | 265 | 286152 |
| 740.2 | Osteoarthrosis, generalized | Musculoskeletal | 0.98 | 0.90 | 1.06 | 306914 | 589 | 306325 |
| 522 | Diseases of pulp and periapical tissues | Digestive | 1.02 | 0.95 | 1.11 | 312297 | 639 | 311658 |
| 427.42 | Cardiac arrest | Circulatory System | 0.98 | 0.92 | 1.05 | 300470 | 949 | 299521 |
| 714 | Rheumatoid arthritis and other inflammatory polyarthropathies | Musculoskeletal | 1.01 | 0.99 | 1.03 | 325905 | 8652 | 317253 |
| 550.1 | Inguinal hernia | Digestive | 1.00 | 0.98 | 1.01 | 296174 | 16385 | 279789 |
| 870 | Open wounds of head; neck; and trunk | Injuries & Poisonings | 0.98 | 0.90 | 1.06 | 320327 | 596 | 319731 |

| | | | | | | | | |
|---|---|---|---|---|---|---|---|---|
| 674 | Other complications of the puerperium NEC | Pregnancy Complications | 1.04 | 0.92 | 1.17 | 328015 | 280 | 327735 |
| 585.3 | Chronic renal failure [CKD] | Genitourinary | 1.01 | 0.97 | 1.05 | 316664 | 2585 | 314079 |
| 625.1 | Dyspareunia | Genitourinary | 1.02 | 0.96 | 1.08 | 319824 | 1155 | 318669 |
| 340 | Migraine | Neurological | 1.01 | 0.97 | 1.05 | 320723 | 2773 | 317950 |
| 474.1 | Acute tonsillitis | Respiratory | 0.98 | 0.90 | 1.06 | 309515 | 565 | 308950 |
| 430.3 | Subdural hemorrhage | Circulatory System | 0.97 | 0.86 | 1.09 | 318378 | 271 | 318107 |
| 275.5 | Disorders of calcium/phosphorus metabolism | Endocrine/Metabolic | 1.02 | 0.96 | 1.08 | 327144 | 1109 | 326035 |
| 531.1 | Hemorrhage from gastrointestinal ulcer | Digestive | 0.98 | 0.91 | 1.06 | 321204 | 655 | 320549 |
| 626 | Disorders of menstruation and other abnormal bleeding from female genital tract | Genitourinary | 1.01 | 0.98 | 1.04 | 300178 | 3947 | 296231 |
| 536 | Disorders of function of stomach | Digestive | 1.04 | 0.92 | 1.17 | 296768 | 257 | 296511 |
| 614 | Inflammatory diseases of female pelvic organs | Genitourinary | 1.03 | 0.93 | 1.14 | 319173 | 374 | 318799 |
| 694.2 | Other dyschromia | Dermatologic | 0.98 | 0.91 | 1.05 | 322549 | 800 | 321749 |
| 447 | Other disorders of arteries and arterioles | Circulatory System | 1.02 | 0.94 | 1.11 | 319916 | 556 | 319360 |
| 735.2 | Acquired toe deformities | Musculoskeletal | 1.01 | 0.97 | 1.06 | 318913 | 2226 | 316687 |
| 426.9 | Cardiac pacemaker/device in situ | Circulatory System | 1.04 | 0.91 | 1.18 | 299758 | 237 | 299521 |
| 610.3 | Fibrosclerosis of breast | Genitourinary | 0.97 | 0.86 | 1.08 | 321176 | 303 | 320873 |
| 531.2 | Gastric ulcer | Digestive | 1.01 | 0.98 | 1.04 | 324777 | 4228 | 320549 |
| 244.4 | Hypothyroidism NOS | Endocrine/Metabolic | 1.00 | 0.98 | 1.01 | 324759 | 14430 | 310329 |
| 823 | Fracture of tibia and fibula | Injuries & Poisonings | 0.97 | 0.87 | 1.08 | 322362 | 342 | 322020 |
| 752.11 | Spina bifida | Congenital Anomalies | 0.96 | 0.84 | 1.10 | 327886 | 211 | 327675 |
| 705.8 | Hyperhidrosis | Dermatologic | 1.02 | 0.94 | 1.11 | 318896 | 602 | 318294 |
| 723 | Other disorders of cervical region | Musculoskeletal | 1.03 | 0.93 | 1.13 | 311345 | 401 | 310944 |
| 385.3 | Cholesteatoma | Sense Organs | 1.02 | 0.94 | 1.11 | 324732 | 589 | 324143 |
| 433.1 | Occlusion and stenosis of precerebral arteries | Circulatory System | 0.98 | 0.92 | 1.04 | 319149 | 1042 | 318107 |
| 729.1 | Rheumatism, unspecified and fibrositis | Musculoskeletal | 1.03 | 0.93 | 1.14 | 305341 | 387 | 304954 |
| 383 | Otosclerosis | Sense Organs | 1.03 | 0.93 | 1.15 | 324473 | 330 | 324143 |
| 835 | Internal derangement of knee | Injuries & Poisonings | 1.01 | 0.98 | 1.04 | 326012 | 4239 | 321773 |
| 575.7 | Other disorders of gallbladder | Digestive | 1.01 | 0.96 | 1.07 | 311719 | 1396 | 310323 |
| 198.1 | Secondary malignancy of lymph nodes | Neoplasms | 0.99 | 0.97 | 1.02 | 234815 | 5503 | 229312 |
| 348.9 | Other conditions of brain, NOS | Neurological | 1.03 | 0.93 | 1.13 | 286559 | 407 | 286152 |
| 621 | Endometrial hyperplasia | Genitourinary | 1.02 | 0.96 | 1.08 | 316461 | 1154 | 315307 |
| 303.3 | Psychogenic disorder | Mental Disorders | 1.02 | 0.95 | 1.09 | 283739 | 841 | 282898 |
| 368.2 | Diplopia and disorders of binocular vision | Sense Organs | 1.02 | 0.95 | 1.10 | 325635 | 753 | 324882 |
| 182 | Malignant neoplasm of uterus | Neoplasms | 0.99 | 0.93 | 1.04 | 298948 | 1303 | 297645 |
| 603.1 | Hydrocele | Genitourinary | 1.01 | 0.96 | 1.07 | 309308 | 1402 | 307906 |
| 624.1 | Dystrophy of female genital tract | Genitourinary | 0.97 | 0.85 | 1.10 | 318909 | 240 | 318669 |
| 345.3 | Convulsions | Neurological | 0.99 | 0.95 | 1.03 | 288463 | 2311 | 286152 |
| 575.9 | Nonspecific abnormal findings on radiological and other examination of biliary tract | Digestive | 1.03 | 0.93 | 1.14 | 310690 | 367 | 310323 |

| | | | | | | | | |
|---|---|---|---|---|---|---|---|---|
| 669 | Complications of labor and delivery NEC | Pregnancy Complications | 1.01 | 0.98 | 1.03 | 328240 | 9534 | 318706 |
| 323.8 | Encephalitis, non-infectious | Neurological | 1.02 | 0.94 | 1.10 | 327771 | 633 | 327138 |
| 184.2 | Cancer of other female genital organs | Neoplasms | 0.97 | 0.87 | 1.08 | 305876 | 323 | 305553 |
| 696.41 | Psoriasis vulgaris | Dermatologic | 1.01 | 0.97 | 1.06 | 315655 | 1723 | 313932 |
| 500.2 | Pneumoconiosis | Respiratory | 1.02 | 0.96 | 1.08 | 317485 | 1001 | 316484 |
| 427.6 | Premature beats | Circulatory System | 1.02 | 0.93 | 1.12 | 299979 | 458 | 299521 |
| 706 | Diseases of sebaceous glands | Dermatologic | 0.98 | 0.90 | 1.06 | 318889 | 595 | 318294 |
| 726.2 | Synoviopathy | Musculoskeletal | 0.97 | 0.87 | 1.08 | 305285 | 331 | 304954 |
| 340.1 | Migrain with aura | Neurological | 1.03 | 0.91 | 1.18 | 318167 | 217 | 317950 |
| 395.2 | Nonrheumatic aortic valve disorders | Circulatory System | 0.97 | 0.85 | 1.10 | 321775 | 247 | 321528 |
| 653 | Problems associated with amniotic cavity and membranes | Pregnancy Complications | 1.01 | 0.96 | 1.07 | 328119 | 1523 | 326596 |
| 174 | Breast cancer | Neoplasms | 1.03 | 0.92 | 1.16 | 304712 | 288 | 304424 |
| 958 | Certain early complications of trauma or procedure | Injuries & Poisonings | 1.03 | 0.93 | 1.13 | 328118 | 381 | 327737 |
| 440.2 | Atherosclerosis of the extremities | Circulatory System | 1.02 | 0.94 | 1.11 | 319912 | 552 | 319360 |
| 530.7 | Gastroesophageal laceration-hemorrhage syndrome | Digestive | 1.03 | 0.92 | 1.14 | 288136 | 333 | 287803 |
| 709.2 | Sicca syndrome | Dermatologic | 1.02 | 0.94 | 1.11 | 242486 | 520 | 241966 |
| 519.9 | Symptoms involving respiratory system and other chest symptoms | Respiratory | 0.97 | 0.87 | 1.09 | 265755 | 308 | 265447 |
| 626.12 | Excessive or frequent menstruation | Genitourinary | 1.00 | 0.97 | 1.02 | 306272 | 10041 | 296231 |
| 523.31 | Acute periodontitis | Digestive | 0.98 | 0.91 | 1.06 | 312371 | 713 | 311658 |
| 480.1 | Bacterial pneumonia | Respiratory | 0.98 | 0.92 | 1.05 | 318628 | 939 | 317689 |
| 624.2 | Atrophy of female genital tract | Genitourinary | 1.02 | 0.93 | 1.12 | 319111 | 442 | 318669 |
| 334.2 | Anterior horn cell disease | Neurological | 1.03 | 0.91 | 1.18 | 286376 | 224 | 286152 |
| 532 | Dysphagia | Digestive | 0.99 | 0.97 | 1.02 | 294356 | 6553 | 287803 |
| 720 | Spinal stenosis | Musculoskeletal | 0.98 | 0.91 | 1.06 | 311636 | 692 | 310944 |
| 433.8 | Late effects of cerebrovascular disease | Circulatory System | 0.99 | 0.93 | 1.04 | 319384 | 1277 | 318107 |
| 426.91 | Cardiac pacemaker in situ | Circulatory System | 0.99 | 0.95 | 1.03 | 301837 | 2316 | 299521 |
| 735.21 | Hammer toe (acquired) | Musculoskeletal | 1.01 | 0.97 | 1.06 | 318701 | 2014 | 316687 |
| 214 | Lipoma | Neoplasms | 0.99 | 0.94 | 1.04 | 322495 | 1819 | 320676 |
| 379.5 | Disorders of iris and ciliary body | Sense Organs | 1.02 | 0.93 | 1.13 | 288604 | 412 | 288192 |
| 293.1 | Swelling, mass, or lump in head and neck [Space-occupying lesion, intracranial NOS] | Mental Disorders | 1.02 | 0.95 | 1.08 | 326044 | 925 | 325119 |
| 564 | Functional digestive disorders | Digestive | 0.99 | 0.93 | 1.05 | 259379 | 983 | 258396 |
| 613.9 | Breast disorder NOS | Genitourinary | 0.99 | 0.97 | 1.02 | 327937 | 5519 | 322418 |
| 961 | Poisoning by other anti-infectives | Injuries & Poisonings | 0.98 | 0.89 | 1.08 | 300647 | 436 | 300211 |
| 761 | Cervicalgia | Symptoms | 0.99 | 0.93 | 1.05 | 328240 | 1005 | 327235 |
| 872 | Traumatic amputation | Injuries & Poisonings | 0.98 | 0.90 | 1.07 | 320213 | 482 | 319731 |
| 447.1 | Stricture of artery | Circulatory System | 0.98 | 0.92 | 1.05 | 320246 | 886 | 319360 |
| 263 | Other nutritional deficiency | Endocrine/Metabolic | 0.99 | 0.94 | 1.04 | 327337 | 1551 | 325786 |
| 594.1 | Calculus of kidney | Genitourinary | 0.99 | 0.96 | 1.03 | 323265 | 3334 | 319931 |

| | | | | | | | | |
|---|---|---|---|---|---|---|---|---|
| 957 | Injury to other and unspecified nerves | Injuries & Poisonings | 1.03 | 0.91 | 1.16 | 328157 | 275 | 327882 |
| 771.1 | Swelling of limb | Symptoms | 0.98 | 0.92 | 1.05 | 327356 | 842 | 326514 |
| 446.9 | Arteritis NOS | Circulatory System | 1.03 | 0.91 | 1.17 | 319605 | 245 | 319360 |
| 165.1 | Cancer of bronchus; lung | Neoplasms | 1.01 | 0.97 | 1.05 | 327450 | 2181 | 325269 |
| 302 | Sexual and gender identity disorders | Mental Disorders | 1.02 | 0.92 | 1.14 | 283238 | 340 | 282898 |
| 312 | Conduct disorders | Mental Disorders | 1.01 | 0.96 | 1.07 | 327638 | 1195 | 326443 |
| 555.2 | Ulcerative colitis | Digestive | 1.01 | 0.97 | 1.04 | 261550 | 3154 | 258396 |
| 512.2 | Painful respiration | Respiratory | 1.03 | 0.90 | 1.17 | 314496 | 228 | 314268 |
| 568.1 | Peritoneal adhesions (postoperative) (postinfection) | Digestive | 0.99 | 0.96 | 1.03 | 249757 | 3097 | 246660 |
| 440 | Atherosclerosis | Circulatory System | 0.97 | 0.84 | 1.11 | 319564 | 204 | 319360 |
| 512.9 | Other dyspnea | Respiratory | 0.99 | 0.92 | 1.05 | 315211 | 943 | 314268 |
| 575.6 | Cholesterolosis of gallbladder | Digestive | 0.98 | 0.89 | 1.07 | 310789 | 466 | 310323 |
| 613.7 | Other signs and symptoms in breast | Genitourinary | 0.98 | 0.92 | 1.06 | 323169 | 751 | 322418 |
| 158 | Neoplasm of unspecified nature of digestive system | Neoplasms | 0.99 | 0.93 | 1.05 | 313429 | 1098 | 312331 |
| 764 | Sciatica | Symptoms | 0.99 | 0.93 | 1.04 | 327944 | 1261 | 326683 |
| 755.1 | Congenital deformities of feet | Congenital Anomalies | 1.03 | 0.90 | 1.17 | 327531 | 220 | 327311 |
| 577 | Diseases of pancreas | Digestive | 1.02 | 0.94 | 1.11 | 326478 | 558 | 325920 |
| 296.2 | Depression | Mental Disorders | 1.00 | 0.98 | 1.01 | 295043 | 12145 | 282898 |
| 287.3 | Thrombocytopenia | Hematopoietic | 0.99 | 0.93 | 1.04 | 326768 | 1271 | 325497 |
| 803.3 | Fracture of clavicle or scapula | Injuries & Poisonings | 0.98 | 0.87 | 1.09 | 323857 | 300 | 323557 |
| 276.12 | Hyposmolality and/or hyponatremia | Endocrine/Metabolic | 1.01 | 0.96 | 1.06 | 322496 | 1852 | 320644 |
| 53 | Herpes zoster | Infectious Diseases | 1.02 | 0.92 | 1.14 | 322547 | 341 | 322206 |
| 965 | Poisoning by analgesics, antipyretics, and antirheumatics | Injuries & Poisonings | 1.01 | 0.98 | 1.03 | 306293 | 6082 | 300211 |
| 726.1 | Enthesopathy | Musculoskeletal | 1.01 | 0.98 | 1.03 | 311657 | 6703 | 304954 |
| 586.2 | Cyst of kidney, acquired | Genitourinary | 0.99 | 0.94 | 1.04 | 315372 | 1293 | 314079 |
| 362.4 | Retinal vascular changes and abnomalities | Sense Organs | 0.99 | 0.92 | 1.05 | 316733 | 881 | 315852 |
| 381.1 | Otitis media | Sense Organs | 1.01 | 0.95 | 1.07 | 325296 | 1153 | 324143 |
| 261.4 | Vitamin D deficiency | Endocrine/Metabolic | 1.02 | 0.93 | 1.12 | 326194 | 408 | 325786 |
| 592.1 | Cystitis | Genitourinary | 0.99 | 0.95 | 1.04 | 299089 | 2054 | 297035 |
| 788 | Syncope and collapse | Symptoms | 1.00 | 0.98 | 1.02 | 328240 | 9372 | 318868 |
| 578.9 | Hemorrhage of gastrointestinal tract | Digestive | 1.01 | 0.98 | 1.03 | 308849 | 5455 | 303394 |
| 530.14 | Reflux esophagitis | Digestive | 1.00 | 0.98 | 1.02 | 298696 | 10893 | 287803 |
| 369.5 | Conjunctivitis, infectious | Sense Organs | 1.03 | 0.90 | 1.17 | 318612 | 224 | 318388 |
| 385.5 | Tympanosclerosis and middle ear disease related to otitis media | Sense Organs | 0.98 | 0.86 | 1.11 | 324379 | 236 | 324143 |
| 574.11 | Cholelithiasis with acute cholecystitis | Digestive | 0.99 | 0.94 | 1.04 | 311836 | 1513 | 310323 |
| 433 | Cerebrovascular disease | Circulatory System | 1.00 | 0.97 | 1.02 | 326753 | 8646 | 318107 |
| 8 | Intestinal infection | Infectious Diseases | 1.00 | 0.98 | 1.03 | 328240 | 9161 | 319079 |
| 198.5 | Secondary malignancy of brain/spine | Neoplasms | 0.99 | 0.92 | 1.06 | 230113 | 801 | 229312 |

| | | | | | | | | |
|---|---|---|---|---|---|---|---|---|
| 367.8 | Hypermetropia | Sense Organs | 1.02 | 0.90 | 1.16 | 325995 | 241 | 325754 |
| 619.1 | Noninflammatory disorders of ovary, fallopian tube, and broad ligament | Genitourinary | 0.99 | 0.92 | 1.06 | 279282 | 734 | 278548 |
| 610.4 | Benign neoplasm of breast | Genitourinary | 1.01 | 0.96 | 1.06 | 322359 | 1486 | 320873 |
| 701.4 | Keloid scar | Dermatologic | 1.02 | 0.90 | 1.17 | 323292 | 228 | 323064 |
| 345.11 | Generalized convulsive epilepsy | Neurological | 1.02 | 0.93 | 1.11 | 286617 | 465 | 286152 |
| 317 | Alcohol-related disorders | Mental Disorders | 1.00 | 0.97 | 1.02 | 303529 | 5860 | 297669 |
| 501 | Pneumonitis due to inhalation of food or vomitus | Respiratory | 1.02 | 0.94 | 1.10 | 317076 | 592 | 316484 |
| 427.8 | Sinoatrial node dysfunction (Bradycardia) | Circulatory System | 0.98 | 0.89 | 1.08 | 299940 | 419 | 299521 |
| 594 | Urinary calculus | Genitourinary | 1.00 | 0.97 | 1.02 | 326897 | 6966 | 319931 |
| 601.4 | Balanoposthitis | Genitourinary | 1.02 | 0.91 | 1.14 | 307689 | 296 | 307393 |
| 840 | Sprains and strains | Injuries & Poisonings | 1.02 | 0.93 | 1.11 | 328201 | 481 | 327720 |
| 291.8 | Alteration of consciousness | Mental Disorders | 1.02 | 0.93 | 1.12 | 321904 | 434 | 321470 |
| 580.2 | Nephrotic syndrome without mention of glomerulonephritis | Genitourinary | 0.98 | 0.90 | 1.08 | 314575 | 496 | 314079 |
| 635.2 | Antepartum hemorrhage, abruptio placentae, and placenta previa | Pregnancy Complications | 1.01 | 0.95 | 1.08 | 320901 | 936 | 319965 |
| 306.9 | Tension headache | Mental Disorders | 1.02 | 0.91 | 1.14 | 283203 | 305 | 282898 |
| 426.21 | First degree AV block | Circulatory System | 1.01 | 0.95 | 1.08 | 300526 | 1005 | 299521 |
| 740.1 | Osteoarthritis; localized | Musculoskeletal | 1.00 | 0.98 | 1.02 | 315797 | 9472 | 306325 |
| 585.31 | Renal dialysis | Genitourinary | 0.98 | 0.90 | 1.07 | 314603 | 524 | 314079 |
| 803 | Fracture of upper limb | Injuries & Poisonings | 0.98 | 0.89 | 1.08 | 323990 | 433 | 323557 |
| 649.1 | Diabetes or abnormal glucose tolerance complicating pregnancy | Pregnancy Complications | 0.98 | 0.85 | 1.12 | 328127 | 208 | 327919 |
| 296 | Mood disorders | Mental Disorders | 1.00 | 0.98 | 1.01 | 295853 | 12955 | 282898 |
| 250.1 | Type 1 diabetes | Endocrine/Metabolic | 1.01 | 0.97 | 1.05 | 310189 | 2699 | 307490 |
| 614.4 | Inflammatory diseases of uterus, except cervix | Genitourinary | 1.02 | 0.91 | 1.14 | 319107 | 308 | 318799 |
| 261.2 | Vitamin B-complex deficiencies | Endocrine/Metabolic | 0.99 | 0.92 | 1.06 | 326559 | 773 | 325786 |
| 701.2 | Scar conditions and fibrosis of skin | Dermatologic | 0.99 | 0.95 | 1.03 | 325471 | 2407 | 323064 |
| 250.22 | Type 2 diabetes with renal manifestations | Endocrine/Metabolic | 1.02 | 0.89 | 1.17 | 307698 | 208 | 307490 |
| 204 | Leukemia | Neoplasms | 1.02 | 0.93 | 1.11 | 323984 | 461 | 323523 |
| 742.9 | Other derangement of joint | Musculoskeletal | 1.02 | 0.92 | 1.12 | 311042 | 400 | 310642 |
| 565.1 | Anal and rectal polyp | Digestive | 1.00 | 0.97 | 1.02 | 254329 | 7669 | 246660 |
| 520.2 | Disturbances in tooth eruption | Digestive | 1.01 | 0.97 | 1.05 | 314095 | 2437 | 311658 |
| 483 | Acute bronchitis and bronchiolitis | Respiratory | 1.02 | 0.89 | 1.18 | 317889 | 200 | 317689 |
| 751.12 | Congenital anomalies of male genital organs | Congenital Anomalies | 1.02 | 0.90 | 1.16 | 326231 | 228 | 326003 |
| 396 | Abnormal heart sounds | Circulatory System | 1.01 | 0.95 | 1.07 | 322603 | 1075 | 321528 |
| 596.1 | Bladder neck obstruction | Genitourinary | 1.01 | 0.96 | 1.05 | 315665 | 2029 | 313636 |
| 520 | Disorders of tooth development | Digestive | 1.01 | 0.93 | 1.11 | 312128 | 470 | 311658 |
| 342 | Hemiplegia | Neurological | 1.01 | 0.96 | 1.06 | 287677 | 1525 | 286152 |
| 747.12 | Valvular heart disease/ heart chambers | Congenital Anomalies | 0.98 | 0.86 | 1.11 | 325592 | 238 | 325354 |
| 250.11 | Type 1 diabetes with ketoacidosis | Endocrine/Metabolic | 0.98 | 0.87 | 1.11 | 307745 | 255 | 307490 |

| | | | | | | | | |
|---|---|---|---|---|---|---|---|---|
| 427 | Cardiac dysrhythmias | Circulatory System | 0.98 | 0.88 | 1.10 | 299844 | 323 | 299521 |
| 695.9 | Unspecified erythematous condition | Dermatologic | 0.99 | 0.90 | 1.08 | 322226 | 477 | 321749 |
| 615 | Endometriosis | Genitourinary | 1.00 | 0.96 | 1.03 | 322888 | 4089 | 318799 |
| 636.2 | Early onset of delivery | Pregnancy Complications | 0.99 | 0.91 | 1.07 | 320594 | 629 | 319965 |
| 949 | Allergies, other | Injuries & Poisonings | 1.02 | 0.92 | 1.12 | 322326 | 420 | 321906 |
| 610 | Benign mammary dysplasias | Genitourinary | 1.02 | 0.89 | 1.17 | 321081 | 208 | 320873 |
| 535.6 | Duodenitis | Digestive | 1.00 | 0.98 | 1.03 | 304405 | 7894 | 296511 |
| 941 | Adverse reaction to serum or vaccine | Injuries & Poisonings | 1.02 | 0.89 | 1.16 | 322128 | 222 | 321906 |
| 622 | Polyp of female genital organs | Genitourinary | 0.99 | 0.90 | 1.08 | 315794 | 487 | 315307 |
| 367.9 | Blindness and low vision | Sense Organs | 0.99 | 0.92 | 1.06 | 326510 | 756 | 325754 |
| 212 | Benign neoplasm of respiratory and intrathoracic organs | Neoplasms | 1.02 | 0.92 | 1.13 | 328240 | 355 | 327885 |
| 721.1 | Spondylosis without myelopathy | Musculoskeletal | 1.01 | 0.95 | 1.07 | 312014 | 1070 | 310944 |
| 550.3 | Femoral hernia | Digestive | 1.01 | 0.94 | 1.09 | 280442 | 653 | 279789 |
| 379 | Other disorders of eye | Sense Organs | 1.00 | 0.99 | 1.01 | 328190 | 39998 | 288192 |
| 54 | Herpes simplex | Infectious Diseases | 1.02 | 0.89 | 1.16 | 322425 | 219 | 322206 |
| 626.14 | Irregular menstrual bleeding | Genitourinary | 1.00 | 0.96 | 1.03 | 300177 | 3946 | 296231 |
| 572 | Ascites (non malignant) | Digestive | 1.01 | 0.96 | 1.06 | 319925 | 1594 | 318331 |
| 464 | Acute sinusitis | Respiratory | 1.02 | 0.89 | 1.17 | 325387 | 208 | 325179 |
| 537 | Other disorders of stomach and duodenum | Digestive | 1.00 | 0.96 | 1.03 | 299994 | 3483 | 296511 |
| 386.1 | Meniere's disease | Sense Organs | 0.99 | 0.91 | 1.07 | 322213 | 564 | 321649 |
| 471 | Nasal polyps | Respiratory | 1.00 | 0.96 | 1.03 | 312342 | 3392 | 308950 |
| 528.7 | Sialolithiasis | Digestive | 0.98 | 0.88 | 1.10 | 322847 | 313 | 322534 |
| 227.2 | Benign neoplasm of parathyroid gland | Neoplasms | 0.99 | 0.89 | 1.09 | 327032 | 390 | 326642 |
| 530.9 | Heartburn | Digestive | 0.99 | 0.95 | 1.04 | 289911 | 2108 | 287803 |
| 300.1 | Anxiety disorder | Mental Disorders | 1.00 | 0.97 | 1.02 | 288368 | 5470 | 282898 |
| 357 | Inflammatory and toxic neuropathy | Neurological | 1.01 | 0.96 | 1.06 | 327375 | 1423 | 325952 |
| 401.22 | Hypertensive chronic kidney disease | Circulatory System | 0.99 | 0.94 | 1.04 | 250343 | 1595 | 248748 |
| 560.2 | Impaction of intestine | Digestive | 1.02 | 0.90 | 1.15 | 258667 | 271 | 258396 |
| 807 | Fracture of ribs | Injuries & Poisonings | 1.02 | 0.89 | 1.16 | 323780 | 223 | 323557 |
| 756 | Other congenital musculoskeletal anomalies | Congenital Anomalies | 0.99 | 0.92 | 1.06 | 328123 | 812 | 327311 |
| 241.1 | Nontoxic uninodular goiter | Endocrine/Metabolic | 1.01 | 0.93 | 1.10 | 310840 | 511 | 310329 |
| 830 | Dislocation | Injuries & Poisonings | 0.99 | 0.95 | 1.04 | 323927 | 2154 | 321773 |
| 189.4 | Malignant neoplasm of other urinary organs | Neoplasms | 1.00 | 0.97 | 1.04 | 327185 | 3223 | 323962 |
| 578.2 | Blood in stool | Digestive | 1.01 | 0.97 | 1.04 | 306143 | 2749 | 303394 |
| 285.2 | Anemia of chronic disease | Hematopoietic | 0.98 | 0.88 | 1.10 | 309194 | 294 | 308900 |
| 295.1 | Schizophrenia | Mental Disorders | 0.99 | 0.91 | 1.07 | 283509 | 611 | 282898 |
| 568 | Other disorders of peritoneum | Digestive | 1.00 | 0.96 | 1.03 | 250464 | 3804 | 246660 |
| 803.2 | Fracture of radius and ulna | Injuries & Poisonings | 0.99 | 0.93 | 1.06 | 324441 | 884 | 323557 |

| | | | | | | | | |
|---|---|---|---|---|---|---|---|---|
| 707 | Chronic ulcer of skin | Dermatologic | 1.01 | 0.95 | 1.07 | 327959 | 1260 | 326699 |
| 751.21 | Cystic kidney disease | Congenital Anomalies | 0.99 | 0.90 | 1.08 | 326461 | 458 | 326003 |
| 611.3 | Lump or mass in breast | Genitourinary | 0.99 | 0.95 | 1.04 | 322461 | 1588 | 320873 |
| 571.81 | Portal hypertension | Digestive | 1.01 | 0.93 | 1.10 | 318900 | 569 | 318331 |
| 695.7 | Prurigo and Lichen | Dermatologic | 0.99 | 0.92 | 1.06 | 322541 | 792 | 321749 |
| 618.5 | Prolapse of vaginal vault after hysterectomy | Genitourinary | 1.01 | 0.93 | 1.10 | 316743 | 501 | 316242 |
| 433.2 | Occlusion of cerebral arteries | Circulatory System | 1.00 | 0.96 | 1.03 | 321087 | 2980 | 318107 |
| 960.2 | Allergy/adverse effect of penicillin | Injuries & Poisonings | 1.00 | 0.98 | 1.01 | 316894 | 16683 | 300211 |
| 112 | Candidiasis | Infectious Diseases | 1.01 | 0.96 | 1.05 | 327650 | 2150 | 325500 |
| 595 | Hydronephrosis | Genitourinary | 0.99 | 0.95 | 1.04 | 321941 | 2010 | 319931 |
| 938 | Dermatitis due to solar radiation | Injuries & Poisonings | 1.00 | 0.97 | 1.05 | 324444 | 2538 | 321906 |
| 288 | Diseases of white blood cells | Hematopoietic | 0.99 | 0.91 | 1.07 | 320928 | 610 | 320318 |
| 600 | Hyperplasia of prostate | Genitourinary | 1.00 | 0.98 | 1.02 | 318965 | 11572 | 307393 |
| 531 | Peptic ulcer (excl. esophageal) | Digestive | 0.99 | 0.88 | 1.11 | 320833 | 284 | 320549 |
| 191 | Malignant and unknown neoplasms of brain and nervous system | Neoplasms | 0.98 | 0.86 | 1.13 | 326662 | 215 | 326447 |
| 389.1 | Sensorineural hearing loss | Sense Organs | 1.01 | 0.92 | 1.11 | 318658 | 464 | 318194 |
| 759 | Other and unspecified congenital anomalies | Congenital Anomalies | 0.99 | 0.92 | 1.07 | 327249 | 679 | 326570 |
| 747 | Cardiac and circulatory congenital anomalies | Congenital Anomalies | 1.01 | 0.95 | 1.07 | 326576 | 1222 | 325354 |
| 285 | Other anemias | Hematopoietic | 1.00 | 0.98 | 1.02 | 320744 | 11844 | 308900 |
| 41.1 | Staphylococcus infections | Infectious Diseases | 1.00 | 0.97 | 1.04 | 315832 | 3200 | 312632 |
| 339 | Other headache syndromes | Neurological | 1.00 | 0.98 | 1.02 | 325888 | 7938 | 317950 |
| 70.3 | Viral hepatitis C | Infectious Diseases | 1.01 | 0.91 | 1.13 | 322549 | 343 | 322206 |
| 809 | Fracture of unspecified bones | Injuries & Poisonings | 0.99 | 0.90 | 1.09 | 323967 | 410 | 323557 |
| 502 | Postinflammatory pulmonary fibrosis | Respiratory | 0.99 | 0.93 | 1.06 | 317367 | 883 | 316484 |
| 575.2 | Obstruction of bile duct | Digestive | 0.99 | 0.93 | 1.06 | 311135 | 812 | 310323 |
| 386.2 | Peripheral or central vertigo | Sense Organs | 1.01 | 0.92 | 1.12 | 322036 | 387 | 321649 |
| 686 | Other local infections of skin and subcutaneous tissue | Dermatologic | 1.00 | 0.98 | 1.02 | 327410 | 11003 | 316407 |
| 245.21 | Chronic lymphocytic thyroiditis | Endocrine/Metabolic | 0.98 | 0.86 | 1.12 | 310548 | 219 | 310329 |
| 481 | Influenza | Respiratory | 1.00 | 0.98 | 1.02 | 327706 | 10017 | 317689 |
| 577.1 | Acute pancreatitis | Digestive | 0.99 | 0.95 | 1.05 | 327437 | 1517 | 325920 |
| 253.2 | Pituitary hypofunction | Endocrine/Metabolic | 0.99 | 0.88 | 1.11 | 324826 | 281 | 324545 |
| 727.4 | Ganglion and cyst of synovium, tendon, and bursa | Musculoskeletal | 1.00 | 0.96 | 1.03 | 307936 | 2982 | 304954 |
| 592.12 | Chronic cystitis | Genitourinary | 1.01 | 0.94 | 1.08 | 297948 | 913 | 297035 |
| 742.8 | Articular cartilage disorder | Musculoskeletal | 1.01 | 0.93 | 1.09 | 311297 | 655 | 310642 |
| 870.3 | Other open wound of head and face | Injuries & Poisonings | 1.00 | 0.97 | 1.04 | 322782 | 3051 | 319731 |
| 529 | Diseases and other conditions of the tongue | Digestive | 0.99 | 0.93 | 1.06 | 323485 | 951 | 322534 |
| 418 | Nonspecific chest pain | Circulatory System | 1.00 | 0.99 | 1.01 | 326032 | 30212 | 295820 |
| 610.2 | Fibroadenosis of breast | Genitourinary | 0.99 | 0.88 | 1.10 | 321189 | 316 | 320873 |

| | | | | | | | | |
|---|---|---|---|---|---|---|---|---|
| 216 | Benign neoplasm of skin | Neoplasms | 1.00 | 0.98 | 1.03 | 327614 | 7865 | 319749 |
| 448 | Disease of capillaries | Circulatory System | 1.00 | 0.97 | 1.02 | 326841 | 7481 | 319360 |
| 738.4 | Acquired spondylolisthesis | Musculoskeletal | 1.01 | 0.90 | 1.15 | 316942 | 255 | 316687 |
| 379.3 | Aphakia and other disorders of lens | Sense Organs | 1.00 | 0.98 | 1.01 | 308920 | 20728 | 288192 |
| 368.9 | Subjective visual disturbances | Sense Organs | 1.01 | 0.93 | 1.09 | 325515 | 633 | 324882 |
| 618.6 | Vaginal enterocele, congenital or acquired | Genitourinary | 1.01 | 0.93 | 1.09 | 316909 | 667 | 316242 |
| 965.2 | Antirheumatics causing adverse effects in therapeutic use | Injuries & Poisonings | 0.99 | 0.91 | 1.08 | 300710 | 499 | 300211 |
| 290.11 | Alzheimer's disease | Mental Disorders | 1.01 | 0.92 | 1.11 | 321872 | 402 | 321470 |
| 366 | Cataract | Sense Organs | 1.00 | 0.98 | 1.01 | 323825 | 16441 | 307384 |
| 785 | Abdominal pain | Symptoms | 1.00 | 0.99 | 1.01 | 328240 | 42311 | 285929 |
| 573.9 | Abnormal serum enzyme levels | Digestive | 0.99 | 0.87 | 1.12 | 318561 | 230 | 318331 |
| 800 | Fracture of lower limb | Injuries & Poisonings | 1.01 | 0.94 | 1.09 | 324256 | 699 | 323557 |
| 197 | Chemotherapy | Neoplasms | 1.00 | 0.98 | 1.01 | 251774 | 22462 | 229312 |
| 288.11 | Neutropenia | Hematopoietic | 1.00 | 0.96 | 1.03 | 323644 | 3326 | 320318 |
| 380.1 | Otitis externa | Sense Organs | 1.01 | 0.93 | 1.10 | 327284 | 532 | 326752 |
| 454.11 | Varicose veins of lower extremity, symptomtic | Circulatory System | 0.99 | 0.92 | 1.07 | 288365 | 652 | 287713 |
| 626.13 | Irregular menstrual cycle | Genitourinary | 1.00 | 0.96 | 1.05 | 298189 | 1958 | 296231 |
| 317.11 | Alcoholic liver damage | Mental Disorders | 1.01 | 0.94 | 1.08 | 298534 | 865 | 297669 |
| 218 | Benign neoplasm of uterus | Neoplasms | 0.99 | 0.90 | 1.09 | 308168 | 388 | 307780 |
| 374.3 | Ptosis of eyelid | Sense Organs | 1.00 | 0.95 | 1.04 | 320284 | 1896 | 318388 |
| 701 | Other hypertrophic and atrophic conditions of skin | Dermatologic | 1.00 | 0.95 | 1.04 | 324891 | 1827 | 323064 |
| 470 | Septal Deviations/Turbinate Hypertrophy | Respiratory | 1.00 | 0.97 | 1.03 | 313771 | 4821 | 308950 |
| 296.22 | Major depressive disorder | Mental Disorders | 1.01 | 0.91 | 1.12 | 283282 | 384 | 282898 |
| 737 | Curvature of spine | Musculoskeletal | 0.99 | 0.92 | 1.07 | 317430 | 743 | 316687 |
| 189.2 | Cancer of bladder | Neoplasms | 0.99 | 0.93 | 1.06 | 324798 | 836 | 323962 |
| 627.1 | Postmenopausal bleeding | Genitourinary | 1.00 | 0.98 | 1.02 | 305697 | 9466 | 296231 |
| 117 | Mycoses | Infectious Diseases | 1.00 | 0.97 | 1.04 | 328235 | 2735 | 325500 |
| 256.4 | Polycystic ovaries | Endocrine/Metabolic | 0.99 | 0.86 | 1.13 | 324764 | 219 | 324545 |
| 250.6 | Polyneuropathy in diabetes | Endocrine/Metabolic | 1.01 | 0.91 | 1.12 | 307871 | 381 | 307490 |
| 524.3 | Anomalies of tooth position/malocclusion | Digestive | 1.01 | 0.90 | 1.13 | 311953 | 295 | 311658 |
| 733 | Other disorders of bone and cartilage | Musculoskeletal | 0.99 | 0.91 | 1.08 | 316001 | 569 | 315432 |
| 571 | Chronic liver disease and cirrhosis | Digestive | 1.01 | 0.93 | 1.10 | 318871 | 540 | 318331 |
| 174.1 | Breast cancer [female] | Neoplasms | 1.00 | 0.96 | 1.04 | 306711 | 2287 | 304424 |
| 300 | Anxiety, phobic and dissociative disorders | Mental Disorders | 0.99 | 0.89 | 1.10 | 283261 | 363 | 282898 |
| 990 | Effects radiation NOS | Injuries & Poisonings | 1.00 | 0.97 | 1.04 | 325633 | 3244 | 322389 |
| 567 | Peritonitis and retroperitoneal infections | Digestive | 0.99 | 0.93 | 1.06 | 247550 | 890 | 246660 |
| 454 | Varicose veins | Circulatory System | 1.00 | 0.96 | 1.05 | 289316 | 1603 | 287713 |
| 522.5 | Periapical abscess | Digestive | 1.00 | 0.94 | 1.05 | 312904 | 1246 | 311658 |

| | | | | | | | | |
|---|---|---|---|---|---|---|---|---|
| 747.1 | Cardiac congenital anomalies | Congenital Anomalies | 1.01 | 0.88 | 1.16 | 325565 | 211 | 325354 |
| 721 | Spondylosis and allied disorders | Musculoskeletal | 0.99 | 0.93 | 1.06 | 311869 | 925 | 310944 |
| 377 | Disorders of optic nerve and visual pathways | Sense Organs | 1.01 | 0.92 | 1.11 | 288628 | 436 | 288192 |
| 577.2 | Chronic pancreatitis | Digestive | 0.99 | 0.91 | 1.08 | 326456 | 536 | 325920 |
| 349 | Other and unspecified disorders of the nervous system | Neurological | 1.00 | 0.99 | 1.01 | 326855 | 40703 | 286152 |
| 681.1 | Cellulitis and abscess of fingers/toes | Dermatologic | 1.01 | 0.93 | 1.09 | 316989 | 582 | 316407 |
| 721.8 | Other allied disorders of spine | Musculoskeletal | 1.00 | 0.96 | 1.03 | 314171 | 3227 | 310944 |
| 318 | Tobacco use disorder | Mental Disorders | 1.00 | 0.99 | 1.02 | 318172 | 20503 | 297669 |
| 626.2 | Dysmenorrhea | Genitourinary | 1.00 | 0.96 | 1.05 | 297910 | 1679 | 296231 |
| 798.1 | Chronic fatigue syndrome | Symptoms | 1.01 | 0.93 | 1.09 | 325335 | 606 | 324729 |
| 420.21 | Acute pericarditis | Circulatory System | 0.99 | 0.87 | 1.13 | 325211 | 228 | 324983 |
| 474.2 | Chronic tonsillitis and adenoiditis | Respiratory | 1.00 | 0.95 | 1.06 | 310135 | 1185 | 308950 |
| 315 | Develomental delays and disorders | Mental Disorders | 0.99 | 0.91 | 1.08 | 326992 | 549 | 326443 |
| 569.2 | Gastrointestinal complications | Digestive | 0.99 | 0.91 | 1.09 | 247132 | 472 | 246660 |
| 526 | Diseases of the jaws | Digestive | 1.00 | 0.99 | 1.02 | 327645 | 15987 | 311658 |
| 523 | Gingival and periodontal diseases | Digestive | 1.01 | 0.92 | 1.10 | 312187 | 529 | 311658 |
| 420.3 | Endocarditis | Circulatory System | 1.01 | 0.93 | 1.08 | 325668 | 685 | 324983 |
| 365.2 | Primary angle-closure glaucoma | Sense Organs | 1.01 | 0.93 | 1.08 | 317491 | 709 | 316782 |
| 605 | Erectile dysfunction [ED] | Genitourinary | 0.99 | 0.88 | 1.11 | 308197 | 291 | 307906 |
| 41.2 | Streptococcus infection | Infectious Diseases | 1.00 | 0.96 | 1.05 | 314280 | 1648 | 312632 |
| 555.21 | Ulcerative colitis (chronic) | Digestive | 0.99 | 0.92 | 1.08 | 258967 | 571 | 258396 |
| 804 | Fracture of hand or wrist | Injuries & Poisonings | 0.99 | 0.92 | 1.07 | 324232 | 675 | 323557 |
| 575.1 | Cholangitis | Digestive | 1.01 | 0.92 | 1.10 | 310820 | 497 | 310323 |
| 496.1 | Emphysema | Respiratory | 1.00 | 0.96 | 1.05 | 295918 | 1726 | 294192 |
| 287.31 | Primary thrombocytopenia | Hematopoietic | 1.01 | 0.91 | 1.11 | 325925 | 428 | 325497 |
| 565 | Anal and rectal conditions | Digestive | 1.00 | 0.98 | 1.02 | 255321 | 8661 | 246660 |
| 527.2 | Sialoadenitis | Digestive | 1.01 | 0.91 | 1.11 | 322908 | 374 | 322534 |
| 526.9 | Jaw disease NOS | Digestive | 1.00 | 0.99 | 1.02 | 327620 | 15962 | 311658 |
| 782.3 | Edema | Symptoms | 1.00 | 0.96 | 1.05 | 328067 | 1715 | 326352 |
| 443.9 | Peripheral vascular disease, unspecified | Circulatory System | 1.00 | 0.96 | 1.04 | 321969 | 2609 | 319360 |
| 791 | Gangrene | Symptoms | 1.00 | 0.92 | 1.08 | 328240 | 579 | 327661 |
| 766 | Neuralgia, neuritis, and radiculitis NOS | Symptoms | 1.01 | 0.90 | 1.13 | 326987 | 304 | 326683 |
| 198.2 | Secondary malignancy of respiratory organs | Neoplasms | 1.00 | 0.96 | 1.04 | 231589 | 2277 | 229312 |
| 345 | Epilepsy, recurrent seizures, convulsions | Neurological | 1.00 | 0.97 | 1.03 | 289613 | 3461 | 286152 |
| 411.1 | Unstable angina (intermediate coronary syndrome) | Circulatory System | 1.00 | 0.97 | 1.03 | 301011 | 5322 | 295689 |
| 427.3 | Other specified cardiac dysrhythmias | Circulatory System | 1.00 | 0.97 | 1.04 | 302836 | 3315 | 299521 |
| 165 | Cancer within the respiratory system | Neoplasms | 1.00 | 0.96 | 1.04 | 328223 | 2954 | 325269 |
| 619.5 | Noninflammatory disorders of vulva and perineum | Genitourinary | 1.00 | 0.95 | 1.06 | 279927 | 1379 | 278548 |

| | | | | | | | | |
|---|---|---|---|---|---|---|---|---|
| 599.2 | Retention of urine | Genitourinary | 1.00 | 0.98 | 1.03 | 227284 | 6857 | 220427 |
| 594.8 | Renal colic | Genitourinary | 1.00 | 0.96 | 1.04 | 322062 | 2131 | 319931 |
| 351 | Other peripheral nerve disorders | Neurological | 1.00 | 0.98 | 1.02 | 325903 | 12892 | 313011 |
| 706.2 | Sebaceous cyst | Dermatologic | 1.00 | 0.98 | 1.02 | 327401 | 9107 | 318294 |
| 599 | Other symptoms/disorders or the urinary system | Genitourinary | 1.00 | 0.99 | 1.01 | 324256 | 103829 | 220427 |
| 587 | Kidney replaced by transpant | Genitourinary | 1.01 | 0.91 | 1.11 | 314477 | 398 | 314079 |
| 623 | Hypertrophy of female genital organs | Genitourinary | 1.00 | 0.95 | 1.06 | 316579 | 1272 | 315307 |
| 509.2 | Respiratory insufficiency | Respiratory | 1.00 | 0.95 | 1.05 | 318272 | 1788 | 316484 |
| 227.3 | Benign neoplasm of pituitary gland and craniopharyngeal duct (pouch) | Neoplasms | 1.01 | 0.90 | 1.12 | 326970 | 328 | 326642 |
| 614.3 | Pelvic inflammatory disease (PID) | Genitourinary | 0.99 | 0.88 | 1.13 | 319048 | 249 | 318799 |
| 386 | Vertiginous syndromes and other disorders of vestibular system | Sense Organs | 1.00 | 0.96 | 1.05 | 323761 | 2112 | 321649 |
| 198 | Secondary malignant neoplasm | Neoplasms | 1.00 | 0.94 | 1.06 | 230431 | 1119 | 229312 |
| 556.1 | Ulceration of intestine | Digestive | 1.00 | 0.93 | 1.08 | 259087 | 691 | 258396 |
| 604.1 | Redundant prepuce and phimosis/BXO | Genitourinary | 1.00 | 0.96 | 1.04 | 310716 | 2810 | 307906 |
| 535.8 | Other specified gastritis | Digestive | 1.00 | 0.98 | 1.02 | 304926 | 8415 | 296511 |
| 735.23 | Hallux rigidus | Musculoskeletal | 1.00 | 0.95 | 1.05 | 318243 | 1556 | 316687 |
| 561 | Symptoms involving digestive system | Digestive | 1.00 | 0.98 | 1.02 | 272941 | 14545 | 258396 |
| 781 | Symptoms involving nervous and musculoskeletal systems | Symptoms | 1.00 | 0.99 | 1.01 | 328238 | 22180 | 306058 |
| 229 | Benign neoplasm of unspecified sites | Neoplasms | 1.00 | 0.99 | 1.01 | 328240 | 46165 | 282075 |
| 472 | Chronic pharyngitis and nasopharyngitis | Respiratory | 1.00 | 0.94 | 1.06 | 309897 | 947 | 308950 |
| 366.2 | Senile cataract | Sense Organs | 1.00 | 0.98 | 1.02 | 315842 | 8458 | 307384 |
| 599.5 | Frequency of urination and polyuria | Genitourinary | 1.00 | 0.97 | 1.03 | 224574 | 4147 | 220427 |
| 198.3 | Secondary malignant neoplasm of digestive systems | Neoplasms | 1.00 | 0.95 | 1.05 | 230877 | 1565 | 229312 |
| 204.12 | Lymphoid leukemia, chronic | Neoplasms | 1.00 | 0.91 | 1.09 | 324030 | 507 | 323523 |
| 783 | Fever of unknown origin | Symptoms | 1.00 | 0.97 | 1.03 | 328240 | 4111 | 324129 |
| 760 | Back pain | Symptoms | 1.00 | 0.98 | 1.03 | 328240 | 5923 | 322317 |
| 281 | Other deficiency anemia | Hematopoietic | 1.00 | 0.98 | 1.02 | 317687 | 8787 | 308900 |
| 596 | Other disorders of bladder | Genitourinary | 1.00 | 0.98 | 1.02 | 321385 | 7749 | 313636 |
| 289.8 | Polycythemia vera, secondary | Hematopoietic | 1.00 | 0.89 | 1.12 | 320039 | 291 | 319748 |
| 603 | Other disorders of testis | Genitourinary | 1.00 | 0.94 | 1.07 | 308895 | 989 | 307906 |
| 378.2 | Nystagmus and other irregular eye movements | Sense Organs | 1.00 | 0.88 | 1.15 | 288407 | 215 | 288192 |
| 947 | Urticaria | Injuries & Poisonings | 1.00 | 0.90 | 1.10 | 322293 | 387 | 321906 |
| 495 | Asthma | Respiratory | 1.00 | 0.99 | 1.01 | 320971 | 26779 | 294192 |
| 628 | Ovarian cyst | Genitourinary | 1.00 | 0.97 | 1.03 | 301051 | 4820 | 296231 |
| 8.52 | Intestinal infection due to C. difficile | Infectious Diseases | 1.00 | 0.92 | 1.08 | 319727 | 648 | 319079 |
| 521.1 | Dental caries | Digestive | 1.00 | 0.97 | 1.04 | 314912 | 3254 | 311658 |
| 292.6 | Hallucinations | Mental Disorders | 1.00 | 0.90 | 1.12 | 321793 | 323 | 321470 |
| 496.2 | Chronic bronchitis | Respiratory | 1.00 | 0.89 | 1.12 | 294485 | 293 | 294192 |

| | | | | | | | | |
|---|---|---|---|---|---|---|---|---|
| 251.1 | Hypoglycemia | Endocrine/Metabolic | 1.00 | 0.94 | 1.06 | 305952 | 977 | 304975 |
| 80 | Postoperative infection | Infectious Diseases | 1.00 | 0.97 | 1.03 | 326101 | 4622 | 321479 |
| 285.1 | Acute posthemorrhagic anemia | Hematopoietic | 1.00 | 0.88 | 1.12 | 309167 | 267 | 308900 |
| 529.1 | Glossitis | Digestive | 1.00 | 0.89 | 1.12 | 322838 | 304 | 322534 |
| 687.4 | Disturbance of skin sensation | Dermatologic | 1.00 | 0.96 | 1.04 | 325793 | 2979 | 322814 |
| 198.4 | Secondary malignant neoplasm of liver | Neoplasms | 1.00 | 0.96 | 1.04 | 232009 | 2697 | 229312 |
| 204.21 | Myeloid leukemia, acute | Neoplasms | 1.00 | 0.90 | 1.12 | 323842 | 319 | 323523 |
| 966 | Poisoning by anticonvulsants and anti-Parkinsonism drugs | Injuries & Poisonings | 1.00 | 0.92 | 1.09 | 300747 | 536 | 300211 |
| 280.1 | Iron deficiency anemias, unspecified or not due to blood loss | Hematopoietic | 1.00 | 0.98 | 1.02 | 316402 | 7502 | 308900 |
| 580.32 | Nephritis and nephropathy with pathological lesion | Genitourinary | 1.00 | 0.94 | 1.06 | 315157 | 1078 | 314079 |
| 350.1 | Abnormal involuntary movements | Neurological | 1.00 | 0.94 | 1.07 | 326371 | 927 | 325444 |
| 332 | Parkinson's disease | Neurological | 1.00 | 0.94 | 1.06 | 287334 | 1182 | 286152 |
| 530.3 | Stricture and stenosis of esophagus | Digestive | 1.00 | 0.96 | 1.05 | 289757 | 1954 | 287803 |
| 290 | Delirium dementia and amnestic and other cognitive disorders | Mental Disorders | 1.00 | 0.92 | 1.10 | 321950 | 480 | 321470 |
| 204.4 | Multiple myeloma | Neoplasms | 1.00 | 0.92 | 1.09 | 324097 | 574 | 323523 |
| 277.4 | Disorders of bilirubin excretion | Endocrine/Metabolic | 1.00 | 0.90 | 1.11 | 283300 | 373 | 282927 |
| 241.2 | Nontoxic multinodular goiter | Endocrine/Metabolic | 1.00 | 0.93 | 1.08 | 311043 | 714 | 310329 |
| 479 | Other upper respiratory disease | Respiratory | 1.00 | 0.98 | 1.02 | 324241 | 15291 | 308950 |
| 535 | Gastritis and duodenitis | Digestive | 1.00 | 0.98 | 1.02 | 314259 | 17748 | 296511 |
| 732 | Osteochondropathies | Musculoskeletal | 1.00 | 0.87 | 1.14 | 315651 | 219 | 315432 |
| 368 | Visual disturbances | Sense Organs | 1.00 | 0.94 | 1.06 | 325963 | 1081 | 324882 |
| 473.4 | Voice disturbance | Respiratory | 1.00 | 0.94 | 1.06 | 310051 | 1101 | 308950 |
| 331.9 | Cerebral degeneration, unspecified | Neurological | 1.00 | 0.91 | 1.10 | 286569 | 417 | 286152 |
| 289 | Other diseases of blood and blood-forming organs | Hematopoietic | 1.00 | 0.97 | 1.03 | 325300 | 4982 | 320318 |
| 610.1 | Cystic mastopathy | Genitourinary | 1.00 | 0.94 | 1.07 | 321832 | 959 | 320873 |
| 175 | Acquired absence of breast | Neoplasms | 1.00 | 0.96 | 1.04 | 305907 | 2516 | 303391 |
| 535.1 | Acute gastritis | Digestive | 1.00 | 0.94 | 1.06 | 297704 | 1193 | 296511 |
| 320 | Meningitis | Neurological | 1.00 | 0.91 | 1.10 | 327577 | 439 | 327138 |
| 619 | Noninflammatory female genital disorders | Genitourinary | 1.00 | 0.99 | 1.01 | 328106 | 49558 | 278548 |
| 252.1 | Hyperparathyroidism | Endocrine/Metabolic | 1.00 | 0.93 | 1.07 | 325359 | 814 | 324545 |
| 707.1 | Decubitus ulcer | Dermatologic | 1.00 | 0.90 | 1.12 | 327025 | 326 | 326699 |
| 571.8 | Liver abscess and sequelae of chronic liver disease | Digestive | 1.00 | 0.92 | 1.09 | 318904 | 573 | 318331 |
| 228 | Hemangioma and lymphangioma, any site | Neoplasms | 1.00 | 0.95 | 1.05 | 328240 | 1635 | 326605 |
| 149 | Cancer of larynx, pharynx, nasal cavities | Neoplasms | 1.00 | 0.94 | 1.07 | 326986 | 945 | 326041 |
| 530.11 | GERD | Digestive | 1.00 | 0.98 | 1.02 | 302292 | 14489 | 287803 |
| 540.11 | Acute appendicitis | Digestive | 1.00 | 0.96 | 1.04 | 327037 | 2658 | 324379 |
| 686.4 | Pyogenic granuloma | Dermatologic | 1.00 | 0.90 | 1.11 | 316787 | 380 | 316407 |
| 519.2 | Respiratory complications | Respiratory | 1.00 | 0.89 | 1.12 | 265743 | 296 | 265447 |

| Supplementary Table 17. Descriptive characteristics of the UK Biobank and Vanderbilt University Biobank (BioVU) cohorts. | | | | | | | |
|---|---|---|---|---|---|---|---|
| Cohort | Total number | Age, years | Sex, female | BMI | SBP, mmHg | DBP, mmHg | Current smoker |
| UK Biobank | 424,439 | 56.8 (8.0) | 229,239 (54.0%) | 27.4 (4.8) | 138.1 (18.6) | 82.2 (10.13) | 43,928 (10.4%) |
| BioVU | 45,517 | 60.8 (20.9 | 25,148 (61.7%) | 28.7 (7.7) | 124.5 (13.2) | 71.3 (8.8) | 13,701 (30.1%) |

Mean (standard deviation) is given for continuous variable and number (%) for categorical variables. BMI: Body Mass Index, SBP: Systolic Blood Pressure, DBP: Diastolic Blood Pressure.

| Supplementary Table 18. Cox regression results for the association between antihypertensive drug classes and risk of diverticulosis. | | | | | | |
|---|---|---|---|---|---|---|
| Antihypertensive class | N | Diverticulosis cases | Hazard Ratio | Lower 95% CI | Upper 95% CI | P |
| ACEI | 7210 | 162 | 1.00 | 0.79 | 1.26 | 0.99 |
| ARB | 4021 | 111 | 1.11 | 0.86 | 1.43 | 0.42 |
| BB | 6908 | 191 | 1.14 | 0.91 | 1.42 | 0.24 |
| CCB | 6756 | 180 | 1.09 | 0.88 | 1.35 | 0.43 |
| Dihydropyridine CCB | 5961 | 142 | 1.01 | 0.80 | 1.28 | 0.91 |
| Non-dihydropyridine CCB | 782 | 37 | 1.49 | 1.03 | 2.14 | 0.03 |
| Two drug classes | 18488 | 465 | 0.95 | 0.78 | 1.15 | 0.61 |
| Three of more drug classes | 5741 | 162 | 1.02 | 0.81 | 1.28 | 0.90 |

Thiazide diuretics are modelled as the reference category (N=5501, Diverticulosis cases=138). The analysis of any CCB subclass was performed in a separate model. ACEI: angiotensin-converting-enzyme inhibitor; ARB: angiotensin receptor blocker; BB: beta-blocker; CCB: calcium channel blocker.

Supplemental Figures

Supplementary Figure 1. Individual ratio method Mendelian randomization estimates for the analysis of beta-blockers and coronary heart disease risk.

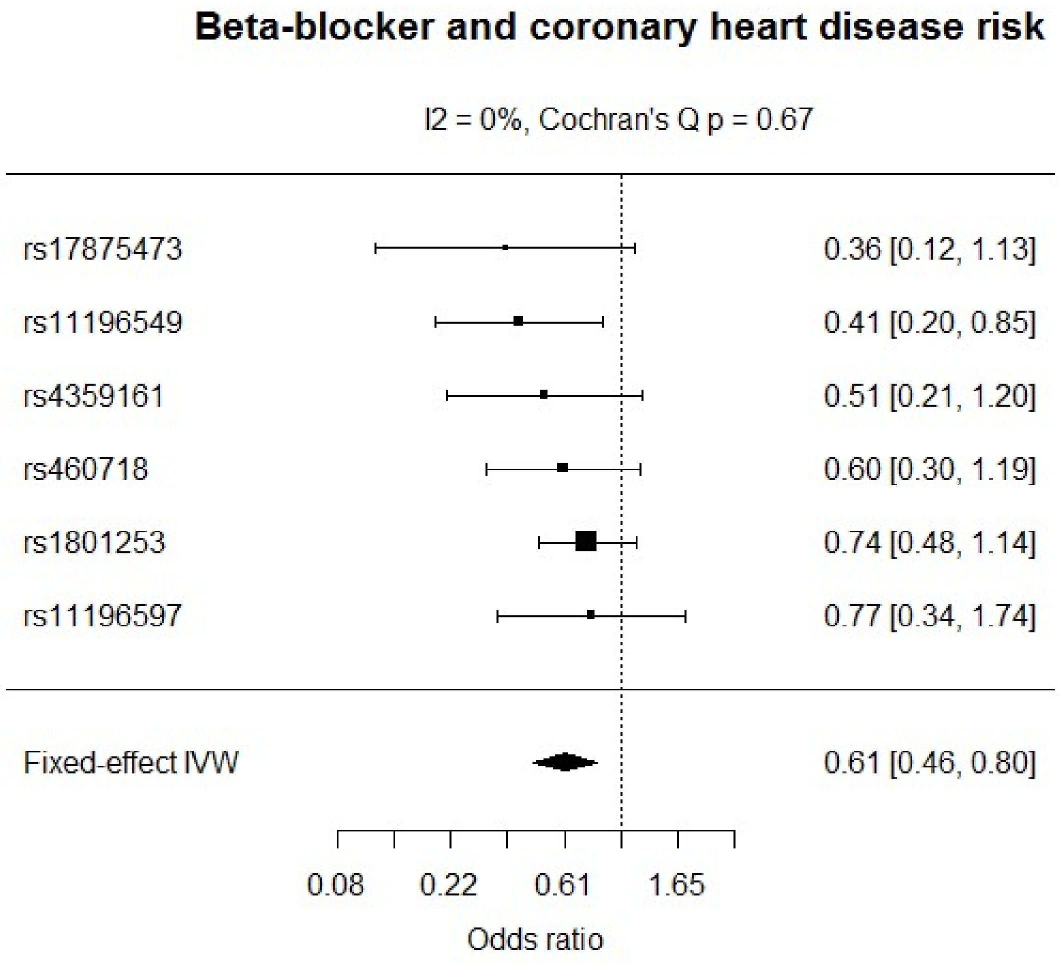


IVW: inverse-variance weighted.

Supplementary Figure 2. Individual ratio method Mendelian randomization estimates for the analysis of beta-blockers and stroke risk.

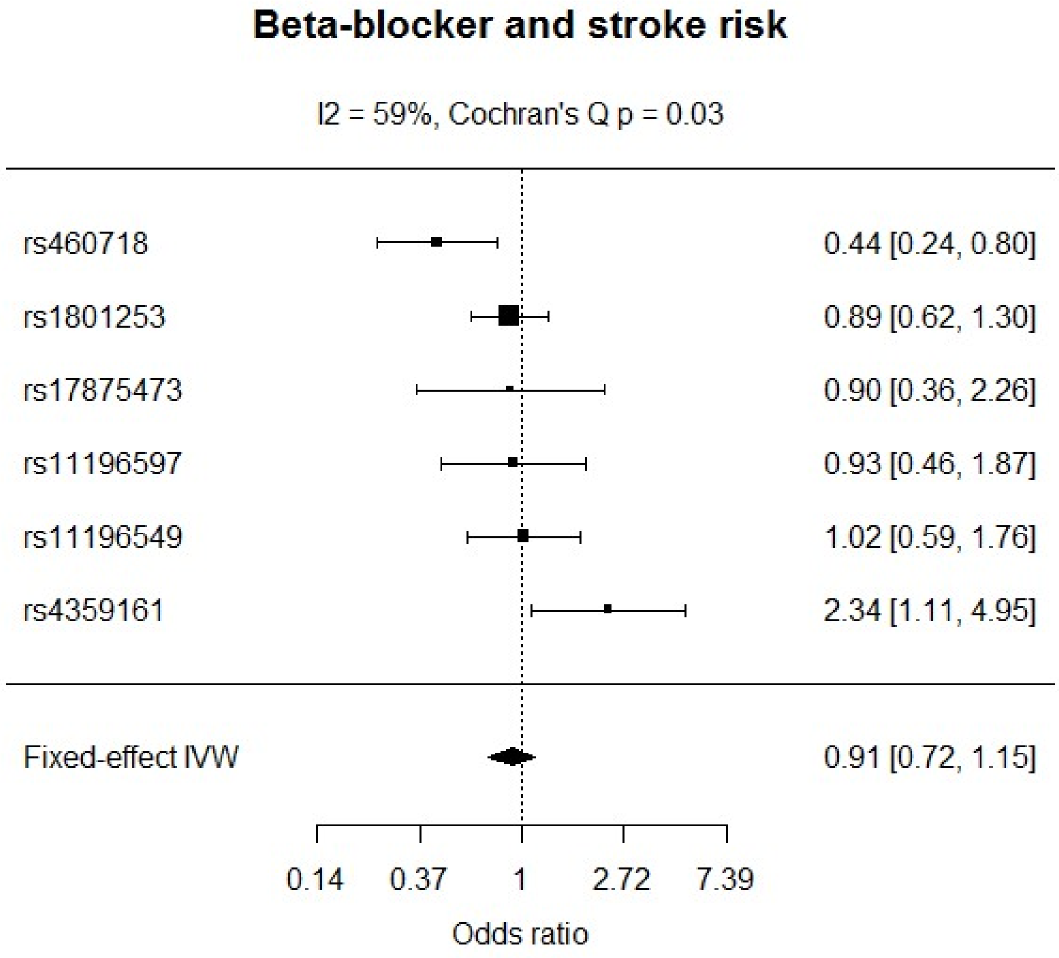


IVW: inverse-variance weighted.

Supplementary Figure 3. Individual ratio method Mendelian randomization estimates for the analysis of calcium channel blockers and coronary heart disease risk.

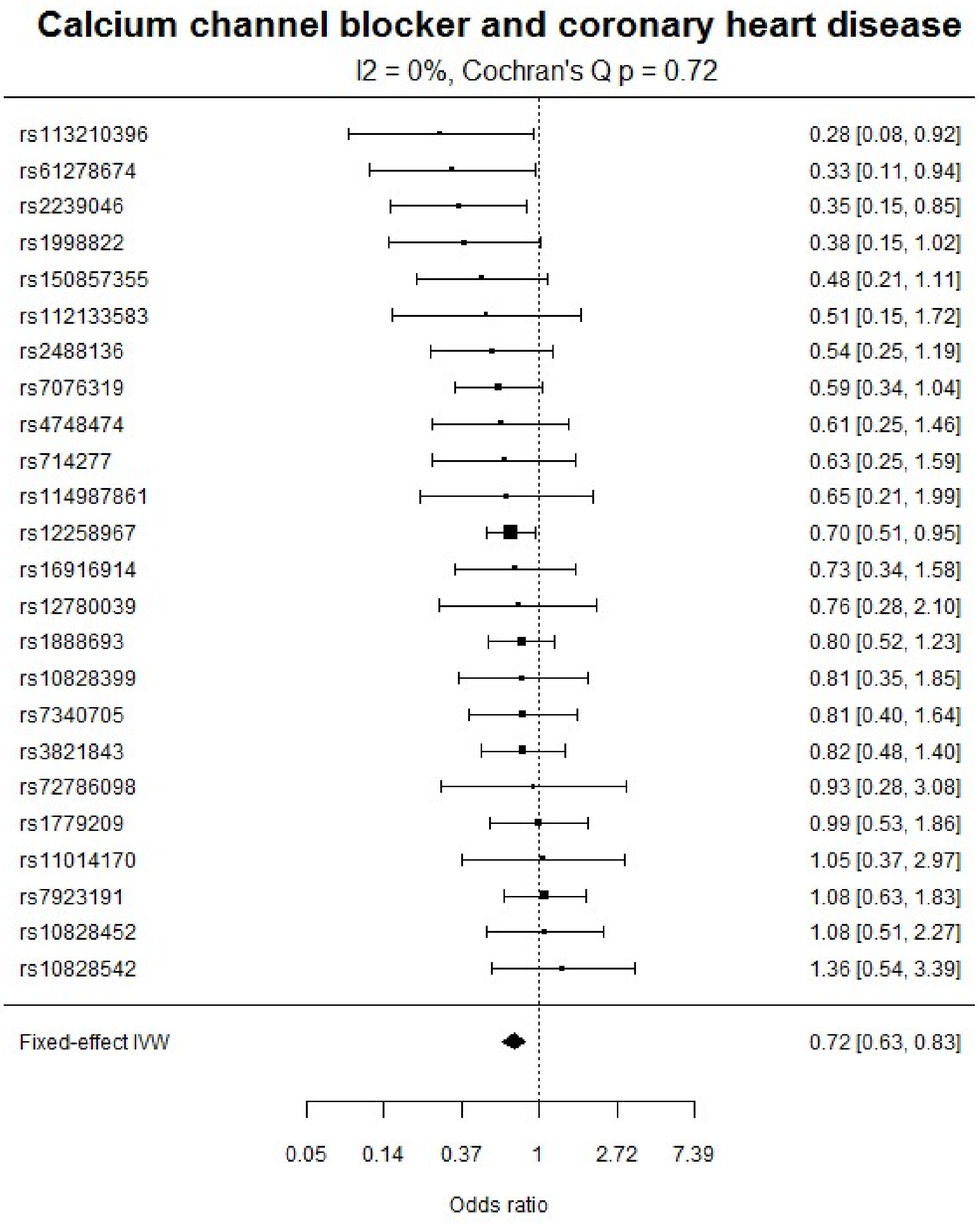


IVW: inverse-variance weighted.

Supplementary Figure 4. Individual ratio method Mendelian randomization estimates for the analysis of calcium channel blockers and stroke risk.

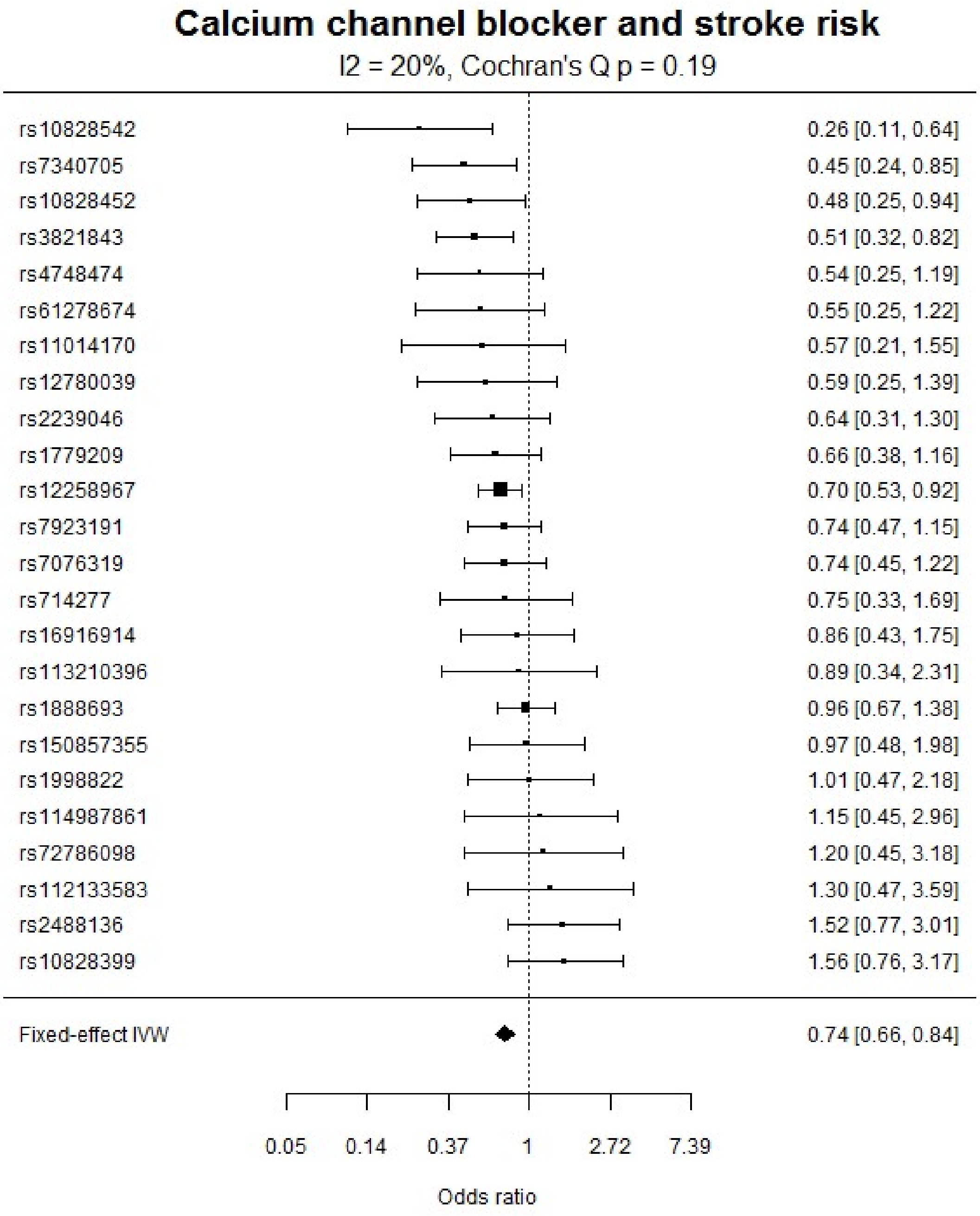


IVW: inverse-variance weighted.

Supplementary Figure 5. Mendelian randomization (MR) sensitivity analyses for the analysis of beta-blockers and coronary heart disease risk.

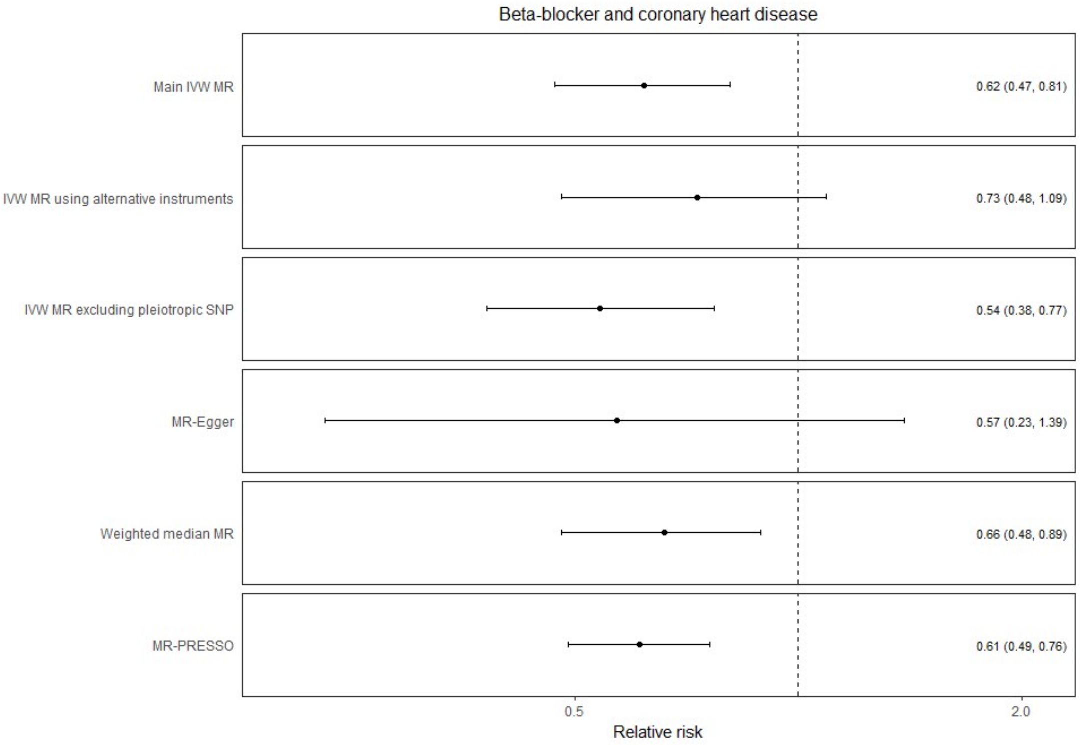


IVW: inverse-variance weighted; SNP: single-nucleotide polymorphism.

Supplementary Figure 6. Mendelian randomization sensitivity analyses for the analysis of beta-blockers and stroke risk.

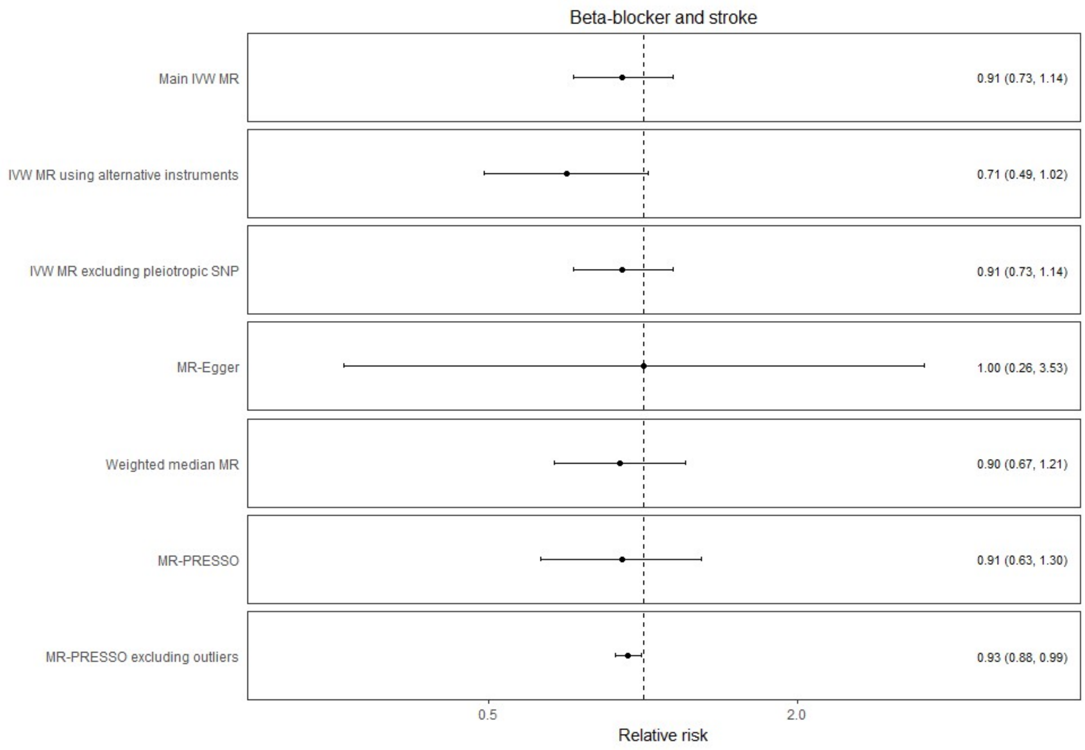


IVW: inverse-variance weighted; SNP: single-nucleotide polymorphism.

Supplementary Figure 7. Mendelian randomization sensitivity analyses for the analysis of calcium channel blockers and coronary heart disease risk.

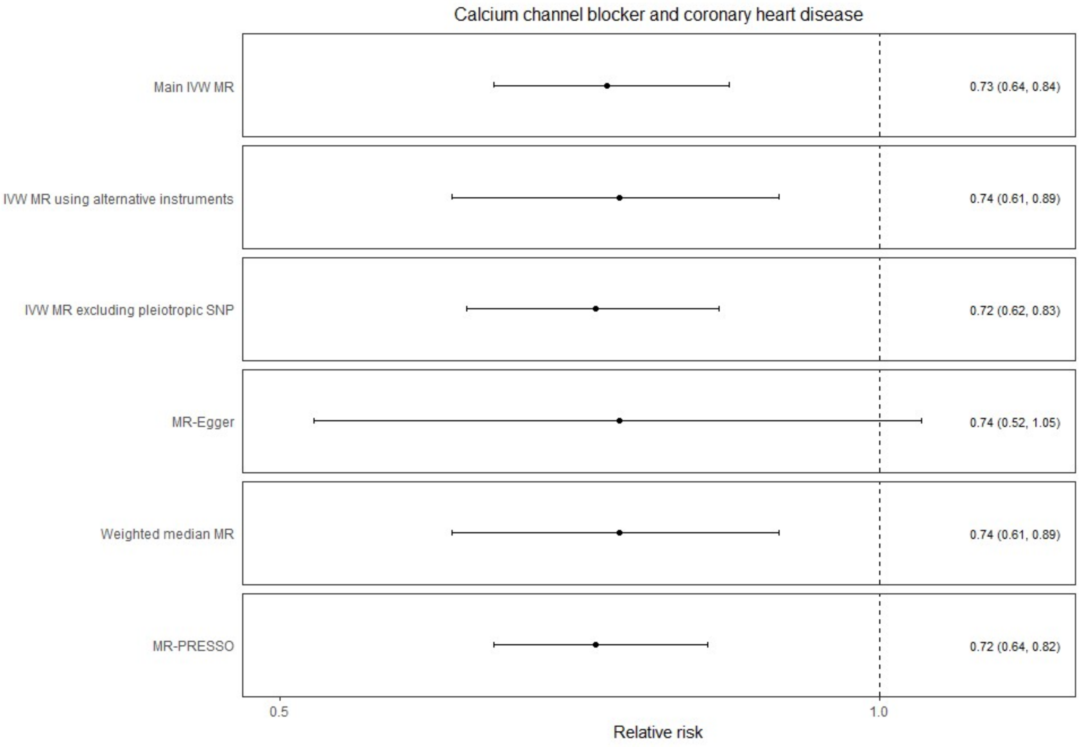


IVW: inverse-variance weighted; SNP: single-nucleotide polymorphism.

Supplementary Figure 8. Mendelian randomization sensitivity analyses for the analysis of calcium channel blockers and stroke risk.

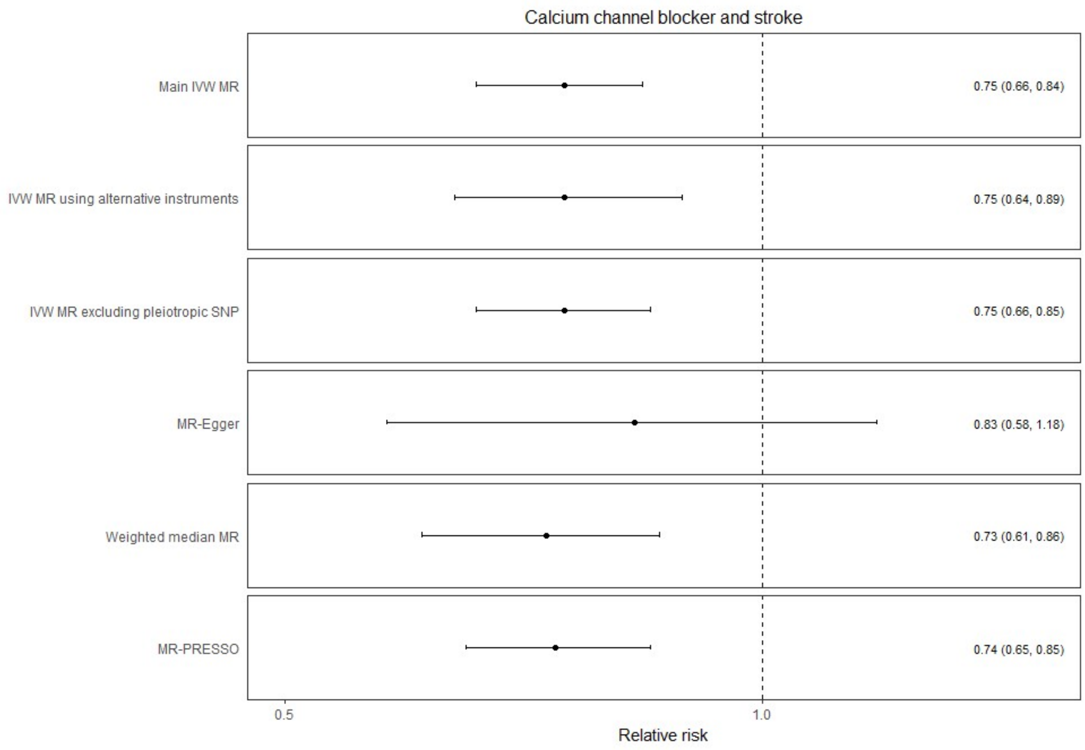


IVW: inverse-variance weighted; SNP: single-nucleotide polymorphism.

Supplementary Figure 9. Permutation analysis randomly sampling 24 systolic blood pressure (SBP) single-nucleotide polymorphisms (SNPs) and investigating association of the resultant standardized genetic risk score (GRS) with diverticulosis risk 1,000 times.

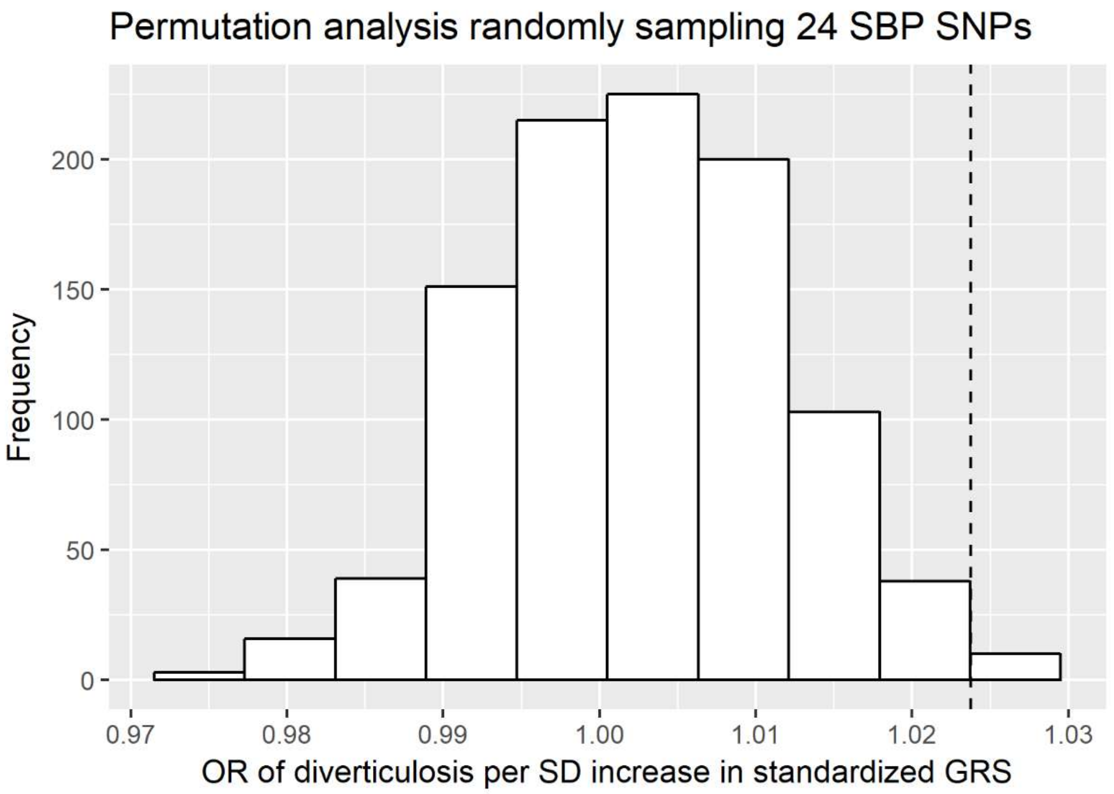


The dashed line represents the result obtained in the main phenome-wide association study analysis using the standardized calcium channel blocker GRS. OR: odds ratio; SD: standard deviation.

# Supplemental References

1. Burgess S, Butterworth A and Thompson SG. Mendelian randomization analysis with multiple genetic variants using summarized data. *Genet Epidemiol*. 2013;37:658-665.

2. Wright JM, Musini VM and Gill R. First-line drugs for hypertension. *Cochrane Database Syst Rev*. 2018;4:CD001841.

3. Ettehad D, Emdin CA, Kiran A, Anderson SG, Callender T, Emberson J, Chalmers J, Rodgers A and Rahimi K. Blood pressure lowering for prevention of cardiovascular disease and death: a systematic review and meta-analysis. *Lancet*. 2016;387:957-967.

4. Burgess S, Bowden J, Fall T, Ingelsson E and Thompson SG. Sensitivity Analyses for Robust Causal Inference from Mendelian Randomization Analyses with Multiple Genetic Variants. *Epidemiology*. 2017;28:30-42.

5. Bowden J, Davey Smith G, Haycock PC and Burgess S. Consistent Estimation in Mendelian Randomization with Some Invalid Instruments Using a Weighted Median Estimator. *Genet Epidemiol*. 2016;40:304-314.

6. Bowden J, Davey Smith G and Burgess S. Mendelian randomization with invalid instruments: effect estimation and bias detection through Egger regression. *Int J Epidemiol*. 2015;44:512-25.

7. Verbanck M, Chen CY, Neale B and Do R. Detection of widespread horizontal pleiotropy in causal relationships inferred from Mendelian randomization between complex traits and diseases. *Nat Genet*. 2018;50:693-698.

8. Evangelou E, Warren HR, Mosen-Ansorena D, Mifsud B, Pazoki R, Gao H, Ntritsos G, Dimou N, Cabrera CP, Karaman I, Ng FL, Evangelou M, Witkowska K, Tzanis E, Hellwege JN, Giri A, Velez Edwards DR, Sun YV, Cho K, Gaziano JM, Wilson PWF, Tsao PS, Kovesdy CP, Esko T, Magi R, Milani L, Almgren P, Boutin T, Debette S, Ding J, Giulianini F, Holliday EG, Jackson AU, Li-Gao R, Lin WY, Luan J, Mangino M, Oldmeadow C, Prins BP, Qian Y, Sargurupremraj M, Shah N, Surendran P, Theriault S, Verweij N, Willems SM, Zhao JH, Amouyel P, Connell J, de Mutsert R, Doney ASF, Farrall M, Menni C, Morris AD, Noordam R, Pare G, Poulter NR, Shields DC, Stanton A, Thom S, Abecasis G, Amin N,

Arking DE, Ayers KL, Barbieri CM, Batini C, Bis JC, Blake T, Bochud M, Boehnke M, Boerwinkle E, Boomsma DI, Bottinger EP, Braund PS, Brumat M, Campbell A, Campbell H, Chakravarti A, Chambers JC, Chauhan G, Ciullo M, Cocca M, Collins F, Cordell HJ, Davies G, Borst MH, Geus EJ, Deary IJ, Deelen J, Del Greco MF, Demirkale CY, Dorr M, Ehret GB, Elosua R, Enroth S, Erzurumluoglu AM, Ferreira T, Franberg M, Franco OH, Gandin I, Gasparini P, Giedraitis V, Gieger C, Girotto G, Goel A, Gow AJ, Gudnason V, Guo X, Gyllensten U, Hamsten A, Harris TB, Harris SE, Hartman CA, Havulinna AS, Hicks AA, Hofer E, Hofman A, Hottenga JJ, Huffman JE, Hwang SJ, Ingelsson E, James A, Jansen R, Jarvelin MR, Joehanes R, Johansson A, Johnson AD, Joshi PK, Jousilahti P, Jukema JW, Jula A, Kahonen M, Kathiresan S, Keavney BD, Khaw KT, Knekt P, Knight J, Kolcic I, Kooner JS, Koskinen S, Kristiansson K, Kutalik Z, Laan M, Larson M, Launer LJ, Lehne B, Lehtimaki T, Liewald DCM, Lin L, Lind L, Lindgren CM, Liu Y, Loos RJF, Lopez LM, Lu Y, Lyytikainen LP, Mahajan A, Mamasoula C, Marrugat J, Marten J, Milaneschi Y, Morgan A, Morris AP, Morrison AC, Munson PJ, Nalls MA, Nandakumar P, Nelson CP, Niiranen T, Nolte IM, Nutile T, Oldehinkel AJ, Oostra BA, O'Reilly PF, Org E, Padmanabhan S, Palmas W, Palotie A, Pattie A, Penninx B, Perola M, Peters A, Polasek O, Pramstaller PP, Nguyen QT, Raitakari OT, Ren M, Rettig R, Rice K, Ridker PM, Ried JS, Riese H, Ripatti S, Robino A, Rose LM, Rotter JI, Rudan I, Ruggiero D, Saba Y, Sala CF, Salomaa V, Samani NJ, Sarin AP, Schmidt R, Schmidt H, Shrine N, Siscovick D, Smith AV, Snieder H, Sober S, Sorice R, Starr JM, Stott DJ, Strachan DP, Strawbridge RJ, Sundstrom J, Swertz MA, Taylor KD, Teumer A, Tobin MD, Tomaszewski M, Toniolo D, Traglia M, Trompet S, Tuomilehto J, Tzourio C, Uitterlinden AG, Vaez A, van der Most PJ, van Duijn CM, Vergnaud AC, Verwoert GC, Vitart V, Volker U, Vollenweider P, Vuckovic D, Watkins H, Wild SH, Willemsen G, Wilson JF, Wright AF, Yao J, Zemunik T, Zhang W, Attia JR, Butterworth AS, Chasman DI, Conen D, Cucca F, Danesh J, Hayward C, Howson JMM, Laakso M, Lakatta EG, Langenberg C, Melander O, Mook-Kanamori DO, Palmer CNA, Risch L, Scott RA, Scott RJ, Sever P, Spector TD, van der Harst P, Wareham NJ, Zeggini E, Levy D, Munroe PB, Newton-Cheh C, Brown MJ, Metspalu A, Hung AM, O'Donnell CJ, Edwards TL, Million Veteran P, Psaty BM, Tzoulaki I, Barnes MR, Wain LV,

Elliott P and Caulfield MJ. Genetic analysis of over 1 million people identifies 535 new loci associated with blood pressure traits. *Nat Genet*. 2018;50:1412–1425.

9. Neale Lab. Rapid GWAS of thousands of phenotypes for 337,000 samples in the UK Biobank. Published 19 July 2017. http://www.nealelab.is/blog/2017/7/19/rapid-gwas-of-thousands-of-phenotypes-for-337000-samples-in-the-uk-biobank. Accessed 30 March 2018.

10. Staley JR, Blackshaw J, Kamat MA, Ellis S, Surendran P, Sun BB, Paul DS, Freitag D, Burgess S, Danesh J, Young R and Butterworth AS. PhenoScanner: a database of human genotype-phenotype associations. *Bioinformatics*. 2016;32:3207-3209.
